# Oil palm (*Elaeis guineensis *Jacq.) tissue culture ESTs: Identifying genes associated with callogenesis and embryogenesis

**DOI:** 10.1186/1471-2229-8-62

**Published:** 2008-05-29

**Authors:** Eng-Ti L Low, Halimah Alias, Soo-Heong Boon, Elyana M Shariff, Chi-Yee A Tan, Leslie CL Ooi, Suan-Choo Cheah, Abdul-Rahim Raha, Kiew-Lian Wan, Rajinder Singh

**Affiliations:** 1 Advanced Biotechnology and Breeding Centre, Biology Division, Malaysian Palm Oil Board (MPOB), 6, Persiaran Institusi, Bandar Baru Bangi, 43000 Kajang, Selangor DE, Malaysia; 2 Faculty of Biotechnology and Biomolecular Sciences, Universiti Putra Malaysia, 43300 UPM Serdang, Selangor DE, Malaysia; 3 Malaysia Genome Institute, Heliks Emas Block, UKM-MTDC Smart Technology Centre, Universiti Kebangsaan Malaysia, 43600 UKM Bangi, Selangor DE, Malaysia; 4 School of Biosciences and Biotechnology, Faculty of Science and Technology, Universiti Kebangsaan Malaysia, 43600 UKM Bangi, Selangor DE, Malaysia; 5 Asiatic Centre for Genome Technology Sdn Bhd (ACGT), Lot L3-I-1, Enterprise 4, Technology Park Malaysia, 57000 Kuala Lumpur, Malaysia; 6 Myagri Associates Sdn. Bhd., 25-2, Jalan Seri Putra 1/2, Bandar Seri Putra Bangi, 43000 Kajang, Selangor DE, Malaysia; 7 Thermo Fisher Scientific, 3, Jalan Sepadu 25/123, Taman Perindustrian Axis, Seksyen 25, 40400 Shah Alam, Selangor Darul Ehsan, Malaysia

## Abstract

**Background:**

Oil palm (*Elaeis guineensis *Jacq.) is one of the most important oil bearing crops in the world. However, genetic improvement of oil palm through conventional breeding is extremely slow and costly, as the breeding cycle can take up to 10 years. This has brought about interest in vegetative propagation of oil palm. Since the introduction of oil palm tissue culture in the 1970s, clonal propagation has proven to be useful, not only in producing uniform planting materials, but also in the development of the genetic engineering programme. Despite considerable progress in improving the tissue culture techniques, the callusing and embryogenesis rates from proliferating callus cultures remain very low. Thus, understanding the gene diversity and expression profiles in oil palm tissue culture is critical in increasing the efficiency of these processes.

**Results:**

A total of 12 standard cDNA libraries, representing three main developmental stages in oil palm tissue culture, were generated in this study. Random sequencing of clones from these cDNA libraries generated 17,599 expressed sequence tags (ESTs). The ESTs were analysed, annotated and assembled to generate 9,584 putative unigenes distributed in 3,268 consensi and 6,316 singletons. These unigenes were assigned putative functions based on similarity and gene ontology annotations. Cluster analysis, which surveyed the relatedness of each library based on the abundance of ESTs in each consensus, revealed that lipid transfer proteins were highly expressed in embryogenic tissues. A glutathione S-transferase was found to be highly expressed in non-embryogenic callus. Further analysis of the unigenes identified 648 non-redundant simple sequence repeats and 211 putative full-length open reading frames.

**Conclusion:**

This study has provided an overview of genes expressed during oil palm tissue culture. Candidate genes with expression that are modulated during tissue culture were identified. However, in order to confirm whether these genes are suitable as early markers for embryogenesis, the genes need to be tested on earlier stages of tissue culture and a wider range of genotypes. This collection of ESTs is an important resource for genetic and genome analyses of the oil palm, particularly during tissue culture development.

## Background

The oil palm belongs to the family Palmaceae and the genus *Elaeis*. There are two important species in the genus *Elaeis*, *E. guineensis *and *E. oleifera*. This diploid monocotyledon is persistent in the dominance of the single vegetative apex, hence producing no adventitious or auxiliary shoots. Prior to the advent of tissue culture, there was no known reliable method for vegetative propagation of oil palm [1]. This makes tissue culture an important aspect in the development of the oil palm industry [2], especially in the generation of superior and uniform oil palm planting materials. Pioneering work in oil palm tissue culture was carried out by Staritsky [3] and Rabechault *et al*. [4].

The use of tissue culture was predicted to improve oil production and this was later confirmed by significant increases in yield (up to 30%) compared to commercial *Dura *× *Pisifera *(D × P) seedlings in large-scale field trials [5,6]. Oil palm tissue culture is predominantly initiated from young leaves in callus induction media. The callus, which is nodular in appearance forms along the cut edges where the veins are exposed [7]. Some of the callus remains compact and nodular and undergoes embryogenesis [7,8]. The process would eventually lead to somatic embryo formation and maturation, shoot regeneration, rooting and finally the recovery of new viable plantlets. However, the callus could also form into soft, granular and translucent tissues, which do not have any embryogenic potential [7]. These tissues are defined as non-embryogenic callus and would remain as callus without much hope of generating new plantlets.

The formation of callus and somatic embryos remains one of the major bottlenecks in oil palm tissue culture. The rate of callogenesis of oil palm explants remains low, at about 19% [9]. It was also reported by Wooi [10] that the average rate of embryogenesis in leaf derived callus ranged from 3% to 6%. Despite the economic importance of oil palm tissue culture, little is known about the chemical characteristics and molecular changes associated with callogenesis and embryogenesis in oil palm.

In plants, numerous approaches have been used to understand the complexity of gene expression and interaction. One of these approaches is the expressed sequence tag (EST). Since its introduction, the technique has proven to be a rapid and efficient way of obtaining information on gene diversity and mRNA expression patterns from a wide variety of tissues, cell types or developmental stages [11-13]. The effectiveness of the technique to identify oil palm genes was demonstrated when Jouannic *et al*. [14] reported the generation of 2,411 ESTs from male and female inflorescences, shoot apices and zygotic embryos. More recently, Ho *et al*. [15] also identified 14,537 ESTs from oil palm zygotic embryos, suspension cells, shoot apical meristems, young flowers, mature flowers and roots.

In the past few years, there has been an increasing interest in the application of the EST approach to understand the molecular mechanisms associated with tissue culture. For example, in *Cichorium intybus*, 2,348 ESTs were isolated from tissue culture samples, of which 33 differentially expressed genes were identified in embryogenic and non-embryogenic genotypes [16]. Efforts to gain insights into somatic embryogenesis have also led to the identification of genes abundantly expressed during somatic embryogenesis [17,18], embryo-specific genes [19] and a glycoprotein that was secreted specifically by non-embryogenic callus [20]. A number of embryogenesis related genes, such as Late-Embryogenic Abundant (LEA), Somatic Embryogenesis Receptor Kinase (SERK) Agamous-like 15 (AGL15), Baby Boom (BBM), Leafy Cotyledon 1 (LEC1), Fusca3 (FUS3) and Leafy Cotyledon 2 (LEC2), which are expressed during both zygotic and somatic embryogenesis, have also been identified [21].

In order to obtain an understanding of the genes expressed during the oil palm tissue culture, a large set of oil palm ESTs was generated from three major stages in the development of oil palm tissue culture. The analysis of this EST collection allowed for the identification of candidate genes, which are relevant in somatic embryogenesis.

## Results

### Characteristics of cDNA libraries

A total of 12 cDNA libraries were generated from leaf-derived embryogenic callus (EC), non-embryogenic callus (NEC) and embryoid (EMB). In order to produce useful sequencing reads, all 12 libraries were size-fractionated and directionally cloned prior to single-pass sequencing. A small-scale quality assessment was performed for all the libraries prior to commencement of large-scale sequencing (Table 1). The titers of all 12 amplified libraries ranged from 1.22 × 10^9 ^to 6.10 × 10^9 ^pfu/ml and the percentage of recombinant clones observed were between 54.8% and 97.0%. Insert size of these libraries ranged from 250 bp to 2,300 bp, with an average of about 1 kb.

**Table 1:** Characteristics of 12 oil palm cDNA libraries

**Developmental stage**	**Library name**	**Titer of amplified library (pfu/ml)**	**Percentage of recombinant clones (%)**	**Average insert size (kb)**
NEC	CA	1.38 × 10^9^	88.5	1.2
	CB	2.96 × 10^9^	85.1	0.7
	CNH	1.22 × 10^9^	93.5	1.2
	CNI	5.56 × 10^9^	93.3	0.7
	CNL	2.83 × 10^9^	93.8	0.8
	CNN	4.64 × 10^9^	86.7	0.8

EC	CEM	1.53 × 10^9^	93.5	0.6
	CEO	6.10 × 10^9^	87.1	1.0

EMB	EA	2.94 × 10^9^	54.8	0.7
	EB	4.86 × 10^9^	91.6	0.4
	EN	2.93 × 10^9^	97.0	2.1
	EO	2.23 × 10^9^	90.5	1.4

### EST generation

A total of 20,949 5'-end reads were generated from the 12 cDNA libraries, yielding a total of 17,599 high-quality sequences with an average edited length of 465 bases. In order to facilitate further analysis, the sequences generated from the 12 cDNA libraries were grouped accordingly into three different developmental stages, which are embryogenic callus (EC), non-embryogenic callus (NEC), and embryoid (EMB) (Table 2). The overall sequencing success rate was approximately 84%. The most number of ESTs were generated from the EMB libraries followed by NEC libraries and the EC libraries. The EST sequences generated in this study were deposited in GenBank under the accession nos. EY396120–EY413718.

**Table 2:** Summary of EST sequencing

	**EC**		**NEC**		**EMB**		**All**	
				
	**No.**	**%**	**No.**	**%**	**No.**	**%**	**No.**	**%**
Total clones sequenced	3,472		7,825		9,652		20,949	
Clean ESTs for assembly	2,716		6,501		8,382		17,599	
Average EST length (base)	348		493		482		465	
No. of consensi	396		1207		1391		3,268	
No. ESTs within consensus	990	36	3,988	61	4,371	52	11,283	64
No. of singletons	1,726	64	2,513	39	4,011	48	6,316	36
Unique sequences^a^	2,122	78	3,720	57	5,402	64	9,584	54

^a^Calculated as the sum of the number of consensi and singletons within a library.

Only 57.6% of the 17,599 ESTs were assigned putative identification using the blastx analysis. Matches with an E-value ≤ 10^-10 ^were assumed to have significant similarity to known sequences in the GenBank non-redundant protein database [22,23]. There was a large percentage of ESTs that shared no significant similarity to sequences in GenBank. This was probably due to the shorter average length of these sequences (389 bases) compared to the ESTs showing significant similarities with the GenBank entries (521 bases).

A total of 3.1% (311/10,138) of the ESTs with significant amino acid sequence similarities were found to be similar to previously identified and characterized oil palm genes. These genes, which include metallothionein-like protein [24], translationally controlled tumor protein and glyceraldehyde 3-phosphate dehydrogenase are listed in Table 3. Based on the comparison with known oil palm sequences, it is clear that genes encoding metallothionein-like protein were the most abundant, representing 37.6% (117/311) of the total ESTs with significant matches to oil palm genes. Metallothionein-like proteins are genes that have been isolated from higher plants including monocot (wheat, maize) and dicot (*Arabidopsis*) [25-27]. These genes play a role in heavy metal detoxification, especially in respect to cadmium, copper and zinc [28]. In oil palm, it was reported that the class I, type 3 metallothionein-like genes, MT3-A and MT3-B, when expressed as a fusion protein of glutathione-S-transferase/MT3-A, possess a strong binding affinity for zinc [24].

**Table 3:** Frequency of ESTs with significant similarities to *Elaeis *sequences

**Database accession no.**	**Putative identity**	**EST frequency**	**Organism**
gb|AAP13098|	1-aminocyclopropane-1-carboxylic acid oxidase	1	*E. guineensis*
gb|AAK74073|	Eukaryotic translation initiation factor 4A-1	1	*E. oleifera*
gb|AAF69015|	Glutelin	1	*E. guineensis*
gb|ABB72844|	NAC protein 1 splice variant 2	1	*E. guineensis*
gb|AAO26314|	Protein disulphide isomerase	1	*E. guineensis*
gb|AAO26315|	6-phosphogluconolactonase	1	*E. guineensis*
gb|AAO26312|	Receptor-like protein kinase	1	*E. guineensis*
gb|AAS72302|	Sodium sulfate symporterarsenite permease	1	*E. oleifera*
gb|AAK28402|	7S globulin	2	*E. guineensis*
gb|AAF60173|	Actin depolymerizing factor	2	*E. guineensis*
gb|AAF60172|	Dehydrin-like protein	2	*E. guineensis*
gb|AAM33419|	Delta-9-stearoyl-acyl-carrier protein desaturase	2	*E. guineensis*
gb|AAL76994|	RNA binding protein	2	*E. oleifera*
gb|AAK72126|	S-adenosyl methionine synthetase	2	*E. oleifera*
gb|AAK97632|	40S ribosomal protein S15	6	*E. oleifera*
gb|ABD66069|	EMZ08	6	*E. guineensis*
gb|AAG27431|	QM-like protein	12	*E. guineensis*
gb|AAT45848|	Actine	13	*E. guineensis*
gb|AAT45847|	Elongation factor 1-alpha 1	16	*E. guineensis*
gb|AAN52490|	Defensin EGAD1	20	*E. guineensis*
gb|ABB72846|	Glyceraldehyde 3-phosphate Dehydrogenase	35	*E. guineensis*
gb|AAQ87663|	Translationally controlled tumor protein	66	*E. guineensis*
emb|CAB5258|	Metallothionein-like protein	117	*E. guineensis*

### Clustering of ESTs

In order to assess the rate of gene discovery in each library, StackPACK analysis [29] was carried out and the unigenes (total number of consensi and singletons) within each library were calculated. The proportion of sequences that were unique within each library was not similar and ranged from 57% to 78% (Table 2). The wide range observed was probably due to the fact that the EC library was not deeply sampled. With the exception of the EC library, the number of ESTs that could be clustered into consensus were quite similar, which is 61% and 52% in NEC and EMB libraries respectively. This suggests that the transcripts were distributed almost equally within these libraries.

To further identify unique sequences and remove redundancies between libraries, all ESTs were clustered. Cluster analysis of the 17,599 ESTs revealed 9,584 unigenes. The analysis formed 3,268 consensus sequences (representing 11,283 or 64% of the total ESTs), with an average consensus length of 666 bases. The remaining 6,316 sequences were singletons, with an average length of 449 bases. Of these 9,584 unigenes, 5,299 showed significant similarity to known sequences in GenBank non-redundant protein database at an E-value cut off of 10^-10^. The remaining 4,285 (44.7%) did not show significant similarity to any known sequences in the database. The assembly results of the libraries are provided in Additional File 1.

**Figure 1:**
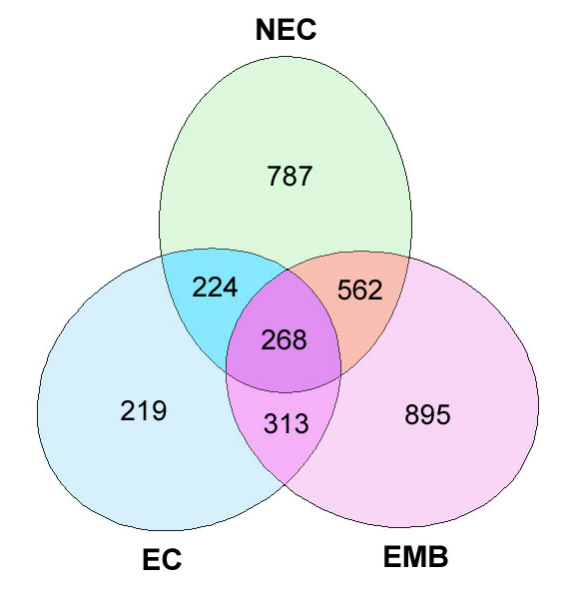
**Distribution of consensi in cDNA libraries**. A total of 3,268 consensi were examined for their distribution among all three cDNA libraries.

Another major advantage of sequencing ESTs from multiple libraries is the ability to identify genes that are putatively transcribed specifically within a certain tissue or during a particular developmental phase. This study revealed that 10,602 sequences (60%) were unique to one of the three tissues sampled (Table 4). As expected, several transcripts were widely expressed in all three tissues sampled. A total of 268 different consensi containing ESTs derived from all three tissues were identified. These transcripts may represent genes, such as those involved in housekeeping, which are common in these tissues (Figure 1 and Table 5). The most abundant transcript detected was putatively identified as ribosomal protein S3 (336 ESTs; cn0037), followed by metallothionein-like protein (117 ESTs; cn3069) and a hypothetical protein (109 ESTs; cn0752). The high percentage of ribosomal proteins identified is expected, as ribosomal proteins are fundamental proteins for living systems and function as intermediary for protein translation.

**Table 4:** Identification of sequences specific to a single tissue

**Tissue**	**Tissue-specific sequences**		**Tissue-specific unigenes**		
		
	**No.**^a^	**%**^b^	**No. consensi**	**No. singletons**	**Total**
EC	1,454	53	219	1,101	1,320
NEC	3,771	58	787	1,872	2,659
EMB	5,377	64	895	3,343	4,238
Total	10,602	60	1,901	6,316	8,217

^a ^The number of tissue-specific sequences was determined by adding the individual sequences in the consensi and singletons that were detected only in a single tissue.

^b ^The percentage of tissue-specific sequences was calculated based on the total number of sequences used for an assembly within that tissue.

**Table 5:** Putative identity of the 20 most abundant sequences present in all three tissues

**Consensus ID**	**No. of ESTs**	**Putative identity**
cn0037	336	Ribosomal protein S3
cn3069	117	Metallothionein-like protein
cn0752	109	Hypothetical protein
cn3128	69	Ribosomal protein L16
cn0925	63	Translationally controlled tumor protein
cn3268	53	Hypothetical protein
cn0959	45	No significant similarity
cn2506	41	Unknown protein
cn2303	40	Endo-1,3;1,4-beta-D-glucanase
cn0086	40	Type 2 metallothionein-like protein
cn2135	32	No significant similarity
cn2719	32	Ubiquitin-conjugating enzyme
cn2943	31	1-Cys peroxiredoxin
cn2451	28	Glyceraldehyde 3-phosphate dehydrogenase
cn1149	27	Translation initiation factor
cn2134	25	No significant similarity
cn0773	22	Non-symbiotic hemoglobin class 1
cn2138	22	Pathogenesis-related protein 4
cn2330	21	No significant similarity
cn1371	21	Sucrose synthase 1

### Protein coding regions

The sequences were also analyzed to identify the base preference of *Elaeis guineensis*, which can be useful for predicting the coding regions of the oil palm genome. This was achieved by identifying full-length open reading frames (ORFs) in the unigene dataset. Homology search results of the 9,584 unigenes using blastx were used to identify unigenes with relatively high similarity (score > 200) to known genes and having an in-frame start and stop codon position similar to the GenBank sequence. Selecting oil palm sequences that had a start and stop codon at a position similar to the protein sequence in GenBank resulted in a more stringent and accurate dataset of full-length ORFs.

A total of 272 putative ORFs were identified, and subsequently translated into amino acid sequences. The amino acid sequences were resubmitted for homology search to confirm the full-length ORFs. Based on the resubmission results, 211 amino acid sequences were identified as putative full-length ORFs. These sequences had a start codon similar to the GenBank sequences, in-frame stop codon and 3 untranslated region (UTR), indicating that the amino acid sequence was translated in the right reading frame. The full-length ORFs were also deposited in GenBank under the accession nos. EU284816–EU285026. The codon usage table for full-length *Elaeis guineensis *ORFs was subsequently generated using CODONW. The oil palm (*Elaeis guineensis*) codon usage table containing 44,372 codons is shown in Table 6. The codon usage table shows that the GC content of the predicted coding region (48.4%) was higher than the predicted 3 UTR (37.8%) and that the GC frequency at the third position is 51.5%. One of the prominent features is that TGA is the preferred stop codon, as it appears in 42.2% of the sequences.

**Table 6:** Codon usage in *Elaeis guineensis*

		Second Letter																	
				
		U				C				A				G					
					
First Letter	U	UUU	Phe	803	0.90	UCU	Ser	590	1.34	UAU	Tyr	733	1.04	UGU	Cys	242	0.76	U	Third Letter
		UUC	Phe	980	1.10	UCC	Ser	468	1.06	UAC	Tyr	683	0.96	UGC	Cys	391	1.24	C	
		UUA	Leu	244	0.38	UCA	Ser	490	1.11	UAA	STOP	65	0.92	UGA	STOP	89	1.27	A	
		UUG	Leu	745	1.17	UCG	Ser	260	0.59	UAG	STOP	57	0.81	UGG	Trp	456	1.00	G	
					
	C	CUU	Leu	988	1.55	CCU	Pro	667	1.36	CAU	His	546	1.14	CGU	Arg	417	0.96	U	
		CUC	Leu	817	1.28	CCC	Pro	459	0.94	CAC	His	411	0.86	CGC	Arg	409	0.94	C	
		CUA	Leu	251	0.39	CCA	Pro	605	1.24	CAA	Gln	552	0.72	CGA	Arg	236	0.54	A	
		CUG	Leu	775	1.22	CCG	Pro	228	0.47	CAG	Gln	981	1.28	CGG	Arg	330	0.76	G	
					
	A	AUU	Ile	1007	1.20	ACU	Thr	654	1.24	AAU	Asn	878	1.00	AGU	Ser	348	0.79	U	
		AUC	Ile	1087	1.29	ACC	Thr	669	1.27	AAC	Asn	885	1.00	AGC	Ser	495	1.12	C	
		AUA	Ile	431	0.51	ACA	Thr	501	0.95	AAA	Lys	1072	0.56	AGA	Arg	503	1.15	A	
		AUG	Met	1078	1.00	ACG	Thr	279	0.53	AAG	Lys	2735	1.44	AGG	Arg	718	1.65	G	
					
	G	GUU	Val	1038	1.30	GCU	Ala	1200	1.38	GAU	Asp	1433	1.24	GGU	Gly	876	1.15	U	
		GUC	Val	822	1.03	GCC	Ala	893	1.03	GAC	Asp	886	0.76	GGC	Gly	676	0.89	C	
		GUA	Val	340	0.42	GCA	Ala	971	1.12	GAA	Glu	1184	0.78	GGA	Gly	884	1.16	A	
		GUG	Val	1001	1.25	GCG	Ala	416	0.48	GAG	Glu	1834	1.22	GGG	Gly	610	0.80	G	

Codon usage was calculated from 211 ORFs containing 44,372 codons.

### EST-derived simple sequence repeat (SSR) markers

Data mining of 9,584 unigenes assembled from 17,599 ESTs identified 648 non-redundant (NR) SSRs in 586 unigenes. The unigenes represented about 5.0 Mb of genic sequences. A total of 56 sequences contained more than one SSR. The NR EST-derived SSRs were represented by mono-, di-, tri-, tetra- and pentanucleotide repeat motifs. This corresponds to an overall SSR density of approximately one SSR per 7.7 kb or an SSR-containing sequence in 6.1% of the NR EST sequences. About 3.4% of the SSRs identified were compound SSRs, which are defined as two neighboring repeats that are located less than 10 nucleotides apart in a single sequence. The frequencies of the SSR motifs identified from 9,584 unigenes are summarized in Table 7.

**Table 7:** Frequency of non-redundant gene-derived SSRs

**SSR motifs**	**Number of repeats**												**Total**
		
	**5**	**6**	**7**	**8**	**9**	**10**	**11**	**12**	**13**	**14**	**15**	**>15**	
A/T	-	-	-	-	-	27	11	6	1	2	4	8	59
C/G	-	-	-	-	-	7	5	1	3	1	1	2	20
AC/GT	-	-	13	5	8	4	1		1	2	1		35
AG/CT	-	-	61	50	41	11	11	6	6	6	8	15	215
AT	-	-	15	10	14	4	5	7	1		2	10	68
CG	-	-		1									1
AAC/GTT	3	4	1										8
AAG/CTT	23	13	9	4	4		1						54
AAT/ATT	9	7	3		3	1	1	1				1	26
ACC/GGT	7	7								1			15
ACG/CTG	11	5	2	1									19
ACT/ATG	12	3	4	1	2	1							23
AGC/CGT	12	2	6	2									22
AGG/CCT	20	7	1	1	1	1							31
AGT/ATC	4	4		1									9
CCG/CGG	14	6	3	3	1								27
AAAG/CTTT	1	1											2
AAAT/ATTT	3	1											4
AACC/GGTT		1											1
ACAT/ATGT	2	2				1							5
ACGG/CCTG	1												1
AGAT/ATCT				1									1
AATAT/ATATT	1												1
ACCAT/ATGGT	1												1
													
N	-	-	-	-	-	34	16	7	4	3	5	10	79
NN	-	-	89	66	63	19	17	13	8	8	11	25	319
NNN	115	58	29	13	11	3	2	1		1		1	234
NNNN	7	5		1		1							14
NNNNN	2												2
													
Total													648

Non-redundant genes were derived from 9,584 unigenes assembled from 17,599 ESTs.

**Figure 2:**
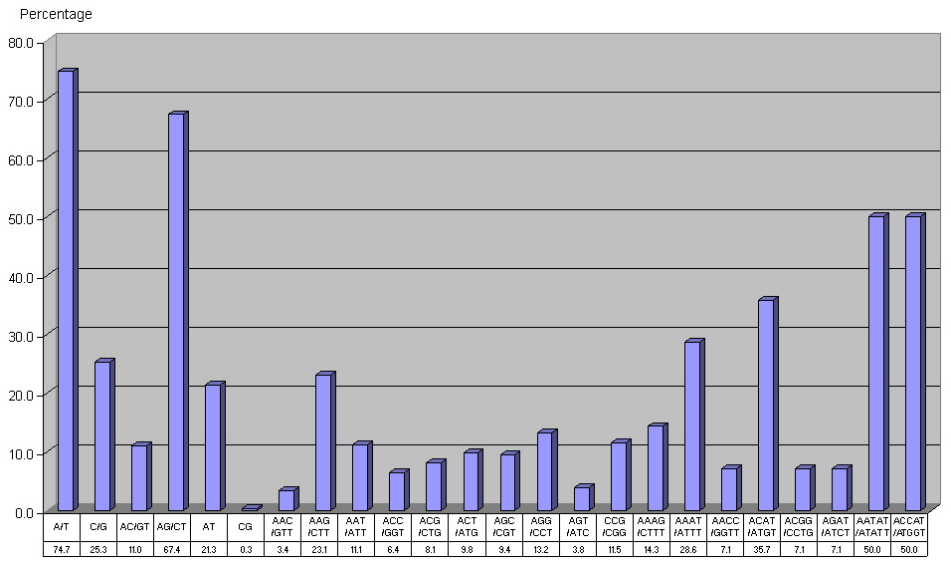
The percentage distribution of the different SSR motifs (mono-, di-, tri-, tetra- and pentanucleotide).

Based on the distribution of SSR motifs, AG/CT motifs represented the most abundant dinucleotide repeat motif. These motifs corresponded to about 67% of the dinucleotide repeat motifs, whereas AT (21%), AC/GT (11%) and CG (0.3%) occurred at relatively low abundance. Among the trinucleotide repeats, AAG/CTT (23%) was the most common motif, followed by AGG/CCT (13%), CCG/CGG (11%) and AAT/ATT (11%). The remaining trinucleotide repeat motifs were less abundant (<10%) with the AGT/ATC (4%) repeat motifs being the least abundant. The most abundant tetranucleotide repeat motifs are the ACAT/ATGT (36%) and AAAT/ATTT (29%) motifs. The distribution of the repeat motifs is shown in Figure 2.

Analysis was also carried out on the 211 putative full-length ORFs to mine for SSR sequences and determine the location (UTR or ORF) of the SSRs identified. A total of 18 SSRs were identified in 17 sequences. Two mononucleotide, seven dinucleotide and nine trinucleotide repeats were identified. The mono- and dinucleotide repeats were identified only in the UTR while the trinucleotide repeats were identified in both the ORF and UTR. A higher percentage (66.7%) of the trinucleotide repeats were identified in the ORF region. The distribution of these SSRs in the putative full-length ORFs is shown in Table 8.

**Table 8:** Distribution of SSRs in putative full-length ORFs

**SSR type**	**Unigene ID**	**SSR**	**SSR position**
Mononucleotide	cn0037	(T)10	UTR
	cn0410	(C)11	UTR

Dinucleotide	cn2588	(AG)11	UTR
	cn2703	(CT)7	UTR
	cn2715	(AG)8	UTR
	cn2719	(AG)10	UTR
	pOP-CBP00138	(TC)13	UTR
	pOP-EO05728	(TC)7	UTR
	pOP-EO06516	(GA)8	UTR

Trinucleotide	cn1550	(AGG)5	UTR
	cn2670	(CTT)5	UTR
	cn2782	(TCC)6	UTR
	cn0320	(TGG)5	ORF
	cn0919	(AAG)7	ORF
	cn0919	(GAA)6	ORF
	cn1384	(GCT)7	ORF
	cn2291	(GAT)5	ORF
	cn2437	(CTG)6	ORF

### Gene ontology annotation

Gene ontology (GO) annotations of the 17,599 oil palm ESTs were performed using Blast2GO [30]. The software performed blastx similarity search against the GenBank non-redundant protein database, retrieved GO terms for the top 20 BLAST results and annotated the sequences based on defined criteria. A total of 8,436 ESTs were successfully annotated with GO terms. In order to help improve the number of sequences annotated with GO terms, additional information was obtained for the ESTs using InterProScan. An additional 755 sequences were then annotated. Overall, a total of 9,191 ESTs were annotated with 33,742 GO terms distributed among the three main GO categories, which are Biological Process (9,253), Molecular Function (13,140) and Cellular Component (11,178). The number of ESTs that were represented with GO terms is probably an underestimate, as 47.8% of the ESTs were not annotated. Generally, the sequences with no BLAST hit (3,100) could not be annotated. However 2,205 of the ESTs with BLAST hit also could not be annotated, while an additional 3,103 sequences did not fulfil the selected criteria for annotation. The majority of the 2,205 genes with BLAST hit could not be annotated with GO terms because the sequences were similar to hypothetical or unknown proteins.

The representations of genes across the various GO terms were examined with WEGO [31]. The distribution and percentage of ESTs in each of the GO terms were calculated. A percentage of 100 was defined as the total number of ESTs that were assigned GO terms in a tissue type, *i.e*. NEC, EC or EMB. However, it must be noted that the percentages of the subcategories do not add up to 100% as many of the ESTs are involved in different classes of function and annotated with multiple GO terms. Generally the distribution of the genes based on the GO terms was quite similar in all three tissues.

Approximately 59.8% of the annotations for the ESTs were grouped into the "cellular process" category in the GO main category Biological Process (Table 9). The category includes processes that are carried out at the cellular level such as cell cycle, cell communication and cell development. The second highest category in Biological Process is the "metabolic process" category, which represents 57.1% of the annotations. The category has subcategories that are involved in photosynthesis, metabolism and regulation of metabolic processes. However, both categories have overlapping subcategories, especially those involved in the cellular metabolic process. It is interesting to note that 5.1% of the annotations were grouped into the "response to stimulus" subcategory. In this category, 307 (3.3%) and 248 (2.7%) ESTs were involved in response function towards stress and chemical stimulants respectively. This is not surprising since the tissue culture environment has been suggested to induce the stress-response mechanism [32].

**Table 9:** Gene ontology (GO) functional classification for ESTs generated from NEC, EC and EMB cDNA libraries

**GO term**	**GO ID**	**All tissues**		**NEC**		**EC**		**Emb**	
					
		**No.**	**%**	**No.**	**%**	**No.**	**%**	**No.**	**%**
**Biological Process**									
Reproduction	GO:0000003	29	0.3	8	0.2	2	0.2	19	0.4
Reproductive process	GO:0022414	17	0.2	7	0.2	2	0.2	8	0.2
Immune system process	GO:0002376	22	0.2	1	0.0	2	0.2	19	0.4
Metabolic process	GO:0008152	5247	57.1	1968	58.3	491	55.3	2788	56.6
Cellular process	GO:0009987	5492	59.8	2058	61.0	521	58.7	2913	59.1
Viral reproduction	GO:0016032	4	0.0	2	0.1	-	-	2	0.0
Reproductive process	GO:0022414	17	0.2	7	0.2	2	0.2	8	0.2
Biological adhesion	GO:0022610	28	0.3	7	0.2	-	-	21	0.4
Multicellular organismal process	GO:0032501	52	0.6	8	0.2	6	0.7	38	0.8
Developmental process	GO:0032502	268	2.9	76	2.3	25	2.8	167	3.4
Growth	GO:0040007	2	0.0	1	0.0	-	-	1	0.0
Response to stimulus	GO:0050896	470	5.1	123	3.6	40	4.5	307	6.2
Localization	GO:0051179	1929	21.0	664	19.7	173	19.5	1092	22.2
Establishment of localization	GO:0051234	1925	20.9	661	19.6	173	19.5	1091	22.1
Maintenance of localization	GO:0051235	18	0.2	9	0.3	2	0.2	7	0.1
Multi-organism process	GO:0051704	32	0.3	13	0.4	2	0.2	17	0.3
Biological regulation	GO:0065007	422	4.6	128	3.8	38	4.3	256	5.2

**Molecular Function**									
Motor activity	GO:0003774	20	0.2	7	0.2	1	0.1	12	0.2
Catalytic activity	GO:0003824	3811	41.5	1430	42.4	309	34.8	2072	42.0
Structural molecule activity	GO:0005198	1266	13.8	563	16.7	141	15.9	562	11.4
Transporter activity	GO:0005215	595	6.5	215	6.4	64	7.2	316	6.4
Binding	GO:0005488	4494	48.9	1749	51.8	389	43.8	2356	47.8
Auxiliary transport protein activity	GO:0015457	1	0.0	-	-	-	-	1	0.0
Antioxidant activity	GO:0016209	219	2.4	49	1.5	18	2.0	152	3.1
Enzyme regulator activity	GO:0030234	102	1.1	52	1.5	5	0.6	45	0.9
Transcription regulator activity	GO:0030528	191	2.1	62	1.8	15	1.7	114	2.3
Translation regulator activity	GO:0045182	188	2.0	50	1.5	15	1.7	123	2.5
Nutrient reservoir activity	GO:0045735	15	0.2	1	0.0	-	-	14	0.3
Molecular transducer activity	GO:0060089	135	1.5	58	1.7	9	1.0	68	1.4

**Cellular Component**									
Extracellular region	GO:0005576	73	0.8	14	0.4	8	0.9	51	1.0
Cell	GO:0005623	6845	74.5	2491	73.8	680	76.6	3674	74.5
Virion	GO:0019012	8	0.1	3	0.1	-	-	5	0.1
Extracellular matrix	GO:0031012	1	0.0	-	-	-	-	1	0.0
Membrane-enclosed lumen	GO:0031974	104	1.1	41	1.2	10	1.1	53	1.1
Envelope	GO:0031975	111	1.2	31	0.9	13	1.5	67	1.4
Macromolecular complex	GO:0032991	1871	20.4	761	22.6	207	23.3	903	18.3
Organelle	GO:0043226	5927	64.5	2185	64.8	602	67.8	3140	63.7
Organelle part	GO:0044422	1148	12.5	541	16.0	112	12.6	495	10.0
Extracellular matrix part	GO:0044420	1	0.0	-	-	-	-	1	0.0
Extracellular region part	GO:0044421	5	0.1	-	-	2	0.2	3	0.1
Virion part	GO:0044423	8	0.1	3	0.1	-	-	5	0.1
Cell part	GO:0044464	6845	74.5	2491	73.8	680	76.6	3674	74.5

Based on the distribution of the annotations for this sub-category in the individual tissues, the results also showed that the number of ESTs was lowest in NEC (3.6%) and gradually increased in EC (4.5%) and EMB (6.2%). It is possible that the expression of stress-response genes is necessary to cope and acclimatise to the stress conditions associated with tissue culture, such as mechanical wounding, osmotic shock and hormonal imbalances. The ability to endure stress can inevitably help the proliferation of culture lines into embryoids.

In the Molecular Function main category, 48.9% of the EST annotations were grouped in the binding category (Table 9). They were represented by a number of GO terms involved in binding, with 1,777 and 1,430 ESTs annotated as "nucleic acid binding" and "ion binding" respectively. Another category that has high levels of representation is in "catalytic activity". Specific catalytic activities that have at least 10% of the ESTs associated with the category are hydrolase, transferase and oxidoreductase subcategories, which are represented by 1,173, 1,118 and 982 ESTs respectively. These genes are involved in processes such as in signal transduction, metabolism and post-translational modification.

### In silico screening and northern blot analysis

Digital northern or *in silico *subtraction was performed to identify candidate genes or markers that are specific in either NEC or embryogenic cultures (EC/EMB). The analysis is derived based on the relationship of the frequencies of the ESTs in a particular tissue with the expression of the genes in that tissue [16,33,34]. The analysis allowed the identification of transcripts that could represent genes that trigger the embryogenesis process.

**Figure 3:**
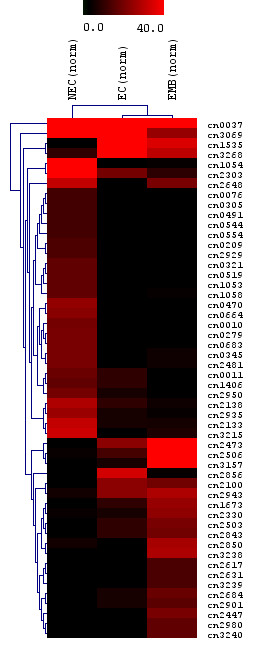
**Hierarchical clustering of normalized EST distribution in a set of 52 consensi**. EC, NEC and EMB represent sequences from the embryogenic callus, non-embryogenic callus and embryoid cDNA libraries respectively. The frequencies of ESTs in a given consensus from each tissue are indicated by increasing intensities of red.

For this purpose, the 3,268 consensi were compiled into a matrix file containing the frequency of ESTs in each consensus sequence corresponding to each of the three tissues (EC, NEC and EMB). Using the Stekel and Falciani statistical test (R test) in IDEG6 [35] with a significance threshold of 0.001, 52 (1.6%) unigenes were identified. The threshold (0.001) allows the selection of differentially expressed genes with almost no false positives [35]. The Stekel and Falciani statistical test was developed to compare gene expression from multiple cDNA libraries [36]. A total of 30 and 22 unigenes were found to be differentially expressed in the NEC and EC/EMB respectively (Table 10). Cluster analysis to determine the relatedness between the tissues based on the abundance of transcript in each consensus, revealed that the NEC samples were distinctly different from EC and EMB cultures (Figure 3).

**Table 10:** Distribution of ESTs in NEC, EC and EMB tissue at a significance threshold of 0.001

		**Distribution of ESTs**			**Distribution of ESTs (Normalized)^b^**			
				
**Unigene ID**	**Putative identity^a^**	**NEC**	**EC**	**EMB**	**NEC**	**EC**	**EMB**	**p-value**
cn0010	No significant similarity	12	0	0	**18.5**	0	0	0.000006
cn0011	No significant similarity	11	2	0	**16.9**	7.4	0	0.000110
cn0037	Ribosomal protein S3	282	20	34	**433.8**	73.6	40.6	0.000000
cn0076	No significant similarity	7	0	0	**10.8**	0	0	0.000938
cn0209	No significant similarity	8	0	0	**12.3**	0	0	0.000346
cn0279	No significant similarity	13	0	0	**19.1**	0	0	0.000002
cn0305	No significant similarity	7	0	0	**10.8**	0	0	0.000938
cn0321	No significant similarity	10	0	0	**15.4**	0	0	0.000047
cn0345	Aspartic proteinase oryzasin 1 precursor	13	0	2	**19.1**	0	2.4	0.000195
cn0470	Cinnamyl alcohol dehydrogenase	15	0	0	**23.1**	0	0	0.000000
cn0491	Zinc finger protein	7	0	0	**10.8**	0	0	0.000938
cn0519	Plastidic aspartate aminotransferase	10	0	0	**15.4**	0	0	0.000047
cn0544	Glutathione S-transferase parA Auxin-regulated protein parA STR246C protein	7	0	0	**10.8**	0	0	0.000938
cn0554	Cationic amino acid transporter	7	0	0	**10.8**	0	0	0.000938
cn0664	Cinnamyl alcohol dehydrogenase	14	0	0	**21.5**	0	0	0.000001
cn0683	No significant similarity	13	0	0	**19.1**	0	0	0.000002
cn1053	Cinnamyl alcohol dehydrogenase	10	0	0	**15.4**	0	0	0.000047
cn1054	Putative cinnamyl alcohol dehydrogenase	31	0	1	**47.7**	0	1.2	0.000000
cn1058	No significant similarity	10	0	1	**15.4**	0	1.2	0.000642
cn1406	Mitogen-activated protein kinase	10	2	0	**15.4**	7.4	0	0.000251
cn2133	No significant similarity	20	1	3	**30.8**	3.7	3.6	0.000018
cn2138	Pathogenesis-related protein 4	18	2	2	**27.7**	7.4	2.4	0.000048
cn2303	Endo-1,3;1,4-beta-D-glucanase	29	5	6	**44.6**	18.4	7.2	0.000009
cn2481	Eukaryotic translation initiation factor 2B, subunit 3	13	0	2	**19.1**	0	2.4	0.000195
cn2648	No significant similarity	20	0	16	**30.8**	0	19.1	0.000861
cn2929	No significant similarity	8	0	0	**12.3**	0	0	0.000346
cn2935	Beta-1, 3-glucanase	16	1	1	**24.6**	3.7	1.2	0.000019
cn2950	No significant similarity	12	1	0	**18.5**	3.7	0	0.000034
cn3069	Metallothionein-like protein	83	14	20	**127.7**	51.5	23.9	0.000000
cn3215	Unknown protein	21	0	4	**32.3**	0	4.8	0.000003
cn1535	PVR3-like protein	0	14	29	0	**51.5**	34.6	0.000000
cn2100	Dehydrin-like protein	0	6	15	0	**22.1**	17.9	0.000057
cn2856	Non-specific lipid transfer protein-C	0	9	1	0	**33.1**	1.2	0.000001
cn2943	1-Cys peroxiredoxin	2	6	23	3.1	**22.1**	**27.4**	0.000314
cn3268	Hypothetical protein	5	23	25	7.7	**84.7**	29.8	0.000000
cn1673	Defensin EGAD1	0	2	20	0	7.4	**23.9**	0.000007
cn2330	No significant similarity	1	1	19	1.5	3.7	**22.7**	0.000127
cn2447	No significant similarity	0	0	16	0	0	**19.1**	0.000007
cn2473	Basic protein 1A, WBP1A lipid transfer protein homolog	0	6	44	0	22.1	**52.5**	0.000000
cn2503	Extensin-like protein	0	2	16	0	7.4	**19.1**	0.000089
cn2506	Unknown protein	1	3	37	1.5	11	**44.1**	0.000000
cn2617	TPA: class III peroxidase 138 precursor	0	0	10	0	0	**11.9**	0.000600
cn2631	Reductase 1	0	0	10	0	0	**11.9**	0.000600
cn2684	Proline-rich SAC51	0	1	14	0	3.7	**16.7**	0.000188
cn2843	Mannoseglucose-specific lectin	0	2	15	0	7.4	**17.9**	0.000165
cn2850	ATP synthase beta subunit	2	0	22	3.1	0	**26.2**	0.000011
cn2901	Histone H3	0	1	12	0	3.7	**14.3**	0.000713
cn2980	Pescadillo-like protein	0	0	13	0	0	**15.5**	0.000065
cn3157	Catalase 2	0	1	43	0	3.7	**51.3**	0.000000
cn3238	Sucrose-synthase 21	0	0	23	0	0	**27.4**	0.000000
cn3239	Mannose-binding lectin Precursor	0	0	10	0	0	**11.9**	0.000600
cn3240	Mannose-binding lectin precursor	0	0	13	0	0	**15.5**	0.000065

^a ^Putative identity was assigned based on BLAST analysis results. No significant similarity represents sequences that do have any similarity to sequences in GenBank at an E-value threshold of 10^-10^.

^b ^Numbers in bold indicate higher expression observed in the specific tissues.

Based on the genes identified, 15 unigenes were represented in NEC only while the ESTs for seven unigenes were present only in EMB. An additional two groups of 15 unigenes that showed a higher expression in NEC and EC/EMB respectively, were also identified. The results showed that lipid transfer protein (cn2473), catalase 2 (cn3157), PVR3-like protein (cn3238), defensin EGAD1 (cn1673) and dehydrin-like protein (cn2100) were among the genes found to be highly expressed in the EC/EMB. The identified transcripts might be involved in somatic embryogenesis initiation, differentiation during somatic embryo development and also in somatic embryo growth, maturation and desiccation. In the NEC libraries, cinnamyl alcohol dehydrogenase, which is involved in lipid biosynthesis, seemed to be abundantly expressed. Stress related genes, such as glutathione S-transferase, pathogenesis-related protein 4, glucanases and metallothionein-like protein were also up-regulated in NEC tissues. A large percentage of genes in the NEC libraries (41%) did not have significant similarity to sequences in GenBank.

**Figure 4:**
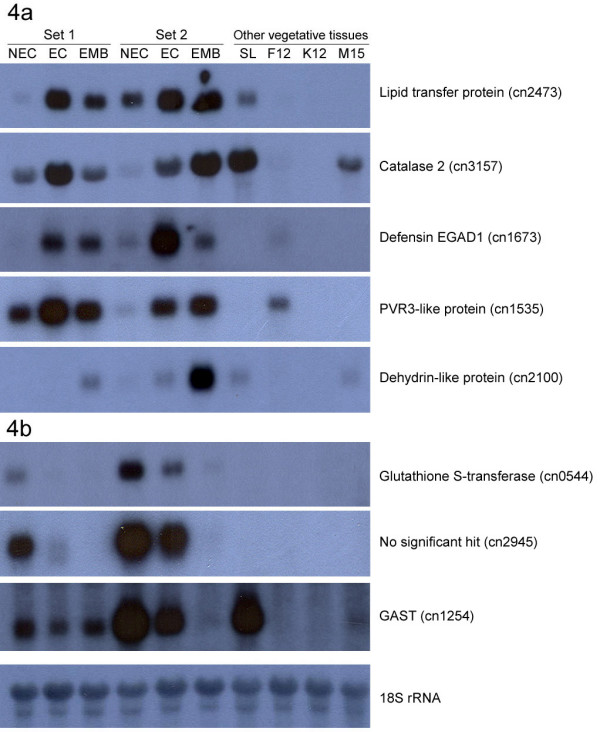
**Northern blot analysis of cDNAs using RNA from various samples**. Figures 4a and 4b are northern blot results performed using somatic embryogenesis and NEC related cDNAs respectively. NEC, non-embryogenic callus; EC, embryogenic callus; EMB, embryoid; SL, spear leaves; F12, flower week-12; K12, kernel week-12; M15, mesocarp week-15. Hybridisation of 18S rRNA showed equal loading of RNA. Each set is represented by one NEC, EC and EMB.

Eight genes were selected for northern blot analysis using RNA extracted from tissue culture and vegetative tissue samples. Based on the digital northern results, five genes that had higher levels of abundance in EC/EMB compared to NEC were selected. One gene, glutathione S-transferase (cn0544) that had a higher level of abundance in NEC compared to EC/EMB in the digital northern results was also selected. The northern blot analysis included an additional two genes that were expressed at low levels in NEC tissues but not in EC/EMB. The two genes were a gibberellin-stimulated transcript (cn1254 having 519 bases) and a transcript with no hit to genes in GenBank (cn2945 having 503 bases). Northern blot analysis was performed and the results are shown in Figure 4. The results showed trends similar to what was observed in the digital northern analysis. The expression for the five EC/EMB enhanced transcripts was lower or not detected in most of the NEC samples when compared to EC and EMB (Figure 4a). However, the lipid transfer protein and dehydrin-like protein were also expressed in the spear leaf while defensin EGAD1 and PVR3-like protein were also found to be expressed in the flower tissue. Catalase 2 expressions were detected at high levels in EC/EMB and spear leaf with a lower expression in NEC and mesocarp tissues. These genes could be important markers to differentiate EC/EMB from NEC.

For the three probes selected for higher abundance in NEC, two transcripts (glutathione S-transferase [cn0544] and a transcript with no hit [cn2945]) were generally confirmed to be more highly expressed in NEC compared to EC/EMB in northern blot analysis (Figure 4b). The expression profile was to some extent genotype dependent, as the difference in expression level was more profound in Set 2 compared to Set 1. However, the expression profile of the last EST (gibberellin-stimulated transcript; GAST) did not correlate with the observation made through *in silico *analysis.

## Discussion

This study aims to provide an insight into the genes expressed during oil palm tissue culture through the generation of 17,599 high-quality ESTs from NEC, EC and EMB tissues. Clustering of the sequences revealed 9,584 unigenes. It is important to note however, that the number of unigenes could be an overestimate as the different contigs could represent different portions of the whole gene. The present EST collection has similarities with the EST data reported by Ho *et al. *[15]. The same oil palm species was used as the starting material and the preliminary data analysis approach employed was also similar. This will enable researchers with various research objectives to perform direct comparisons and study the expression of genes in the various oil palm tissues. However, the EST data reported by Ho *et al*. [15] and our study were derived from different sets of tissues. Thus, as the main focus of this study is to examine the gene expression of oil palm NEC, EC and EMB, no direct comparisons were made between the two EST datasets. The present collection also revealed some prominent features, such as the base preference of oil palm, which were not previously reported in the oil palm EST publications. The ESTs in this study were also mined for putative full-length ORFs and SSR markers.

### Characteristics of the oil palm transcriptome

#### Codon usage

An oil palm base preference would be an important resource to accurately predict protein-coding regions in the oil palm genome. To achieve this, 211 putative full-length ORFs were identified and used to generate the oil palm codon usage table. The codon usage table showed that the GC content (48.4%) was similar to the GC content in *Elaeis guineensis *ESTs as reported by Jouannic *et al. *[14] (49.6%) and Ho *et al. *[15] (about 48.0%), and genes (50.8%) in the Kazusa codon usage database [37]. However, the GC frequency at the third position (51.5%) was slightly lower than previously predicted (56.4%) in the Kazusa codon usage database. The difference could be due to the different dataset size and genes used in this study compared to the Kazusa codon usage database, which only used 56 ORFs. Another reason for the variation could be the difference in the codon preference between gene species, as about 32.0% of the ORFs identified in this study coded for ribosomal proteins or structural constituent of ribosomes while the Kazusa codon usage database had a high percentage of MADS box genes. In fact, Asamizu *et al. *[38] had previously reported that higher organisms showed highly variable codon preference among genes.

The data also revealed that oil palm suppresses the CG dinucleotide in the last two codon positions, where the XCG/XCC ratio is 0.48. This CG suppression has been documented in plants, whereby *Populus*, pea, soybean, potato and spinach have an XCG/XCC ratio of 0.38, 0.51, 0.37, 0.48 and 0.42 respectively [39]. The suppression is possibly due to the high mutation rate of methylated C to T in the CG dinucleotides [39,40].

#### EST-derived SSR markers

The unigene sequences were further mined to identify EST-derived SSR markers. The main benefit of using the non-redundant set of sequences is to provide a more accurate representation of the densities of SSR motifs in the transcribed portions of the genome [41,42]. Kantety *et al. *[43] observed a reduction in redundancy by about 85.0% when the non-redundant sequences were analyzed compared to all the ESTs available in the study. Based on the 9,584 unigenes available, 648 SSRs were identified. The overall density of SSRs (one SSR per 7.7 kb) was similar to the densities reported by Varshney *et al. *[44] in barley (1/7.5 kb) and maize (1/7.5 kb). The density was also similar to the occurrence of one microsatellite motif every 7.73 kb of EST sequence in *Coffea *[41]. However, SSR density in oil palm is lower than those reported in wheat (1/6.2 kb), rye and sorghum (1/5.5 kb) and rice (1/3.9 kb), which are between 1/3.9 kb and 1/6.2 kb [44]. The differences could be due to the different SSR search criteria and software used.

Among the dinucleotide repeat motifs identified, AG/CT repeats were the most common in the dataset. The abundance of the AG and CT repeats has also been reported in *Coffea *[41], barley [45] and wheat [46]. Kantety *et al. *[43] suggested that the high level of occurrence of the GA and CT motifs is due to the high frequency of the translated amino acid products of the motifs. The GA/CT motifs are translated into GAG (Glu), AGA (Arg), CUC (Leu) and UCU (Ser). This is supported by the data in the oil palm codon usage table. These four amino acids occur in 25.3% of the codons analyzed and have a relatively higher frequency than the amino acids produced by the other dinucleotide repeats. The most rare dinucleotide repeat is CG/GC, which is in accordance with reports by Kantety *et al. *[43], Varshney *et al. *[44] and Asp *et al. *[47]. Reports suggest that methylated C has a high mutation rate to T in CG dinucleotides [39,40]. This could explain the reduced occurrence of XCG amino acids resulting from CG repeat motifs.

The AAG/CTT repeat motif is the most frequently occurring oil palm trinucleotide repeat. Kumpatla and Mukhopadhyay [48] reported that the AAG/AGA/GAA/CTT/TTC/TCT repeat motifs were the most predominant repeats in the EST collections of 16 out of the 20 species analyzed. Trinucleotide repeats were also reported as the most abundant SSR repeat class [41,42,44,46,47], as they do not lead to frame shift mutations that would be prone to negative selection [48]. However, in oil palm, the dinucleotide repeats have been identified as the most prevalent repeat class in the EST data. At least two reports had identified dinucleotide repeats as the most prevalent repeat class [48,49]. Studies have shown that dinucleotide repeats are predominantly found in the UTR [41,50]. This might suggest that the SSRs found in the oil palm EST population are mostly in the UTR instead of the coding region, which is why the dinucleotide SSRs are the most prevalent. This hypothesis is supported by SSR analysis of the 211 putative full-length ORFs. Twelve SSRs (two mononucleotide, seven dinucleotide and three trinucleotide repeat motifs) were identified in the UTR while only six repeats (all trinucleotides) were located in the ORF (Table 8). However, this has to be confirmed by determining the location of the UTR and ORF in the remaining SSR-containing sequences.

#### Identification of somatic embryogenesis-related genes

The cDNA libraries were constructed from NEC, EC and EMB as the tissues represented three distinct stages of tissue culture. The ESTs that were identified showed that all the libraries were informative and could provide sequences that are unique to each tissue type. Sequence assembly and digital northerns identified patterns of expression specific to embryogenic and non-embryogenic tissues by examining sequences unique to either NEC or EC/EMB. The technique identified 52 unigenes that were differentially expressed in NEC and EC/EMB. Tissue culture is known to be a complex phenomenon that is affected by a number of factors, such as the culture and media condition, environment and the genotype of the selected palms [7]. The identification of a single gene to differentiate the NEC from EC is thus not likely. Therefore, the expression profile of a combination of genes is necessary to provide a signature that could differentiate these tissues. The availability of such an expression profile would make oil palm tissue culture more viable, especially since the process from explant to field planting can take up to 58 months [7]. Therefore, the collective profile of the 52 genes could be used as a preliminary screen to differentiate NEC from EC. This was obvious from the cluster analysis carried out using these genes, where NEC tissue could be differentiated from EC/EMB. However, further work needs to be carried out to further confirm and reduce the set of genes to a more manageable number to make it viable to screen large number of samples.

Towards this end, the profiles of some of the interesting gene families and genes that could play an important role in embryogenesis were investigated. One of the gene families selected is the lipid transfer protein (LTP) family. Previous studies have demonstrated that LTPs are present in carrot embryogenic cultures [51], grapevines somatic embryos [52], suspension cell cultures [15], microspore-derived embryos [53] and have been implicated in embryogenesis of *Arabidopsis *[54]. The digital northern results showed that three LTP genes were highly redundant and appeared specifically in EC/EMB.

Northern blot analysis of two of these LTPs, cn2473 and cn1535 that shared significant similarity with a wheat LTP [55] and a non-specific LTP respectively, showed that the genes were expressed at very high levels in EC/EMBs and exhibited minimal expression in NECs (Figure 4a). The expression was absent in other vegetative tissues except spear leaf (cn2473) and inflorescence (cn1535). It is interesting to note that the expression pattern of a carrot LTP gene was also similar, whereby its expression was detected in EC/EMB but not in NEC [51]. In addition, cn2473 seemed to be expressed higher in EC compared to EMB. Similar observations were made in cotton [56], where a LTP was found to be highly expressed in subcultured and primary embryogenic callus compared to globular and heart embryos. Zeng *et al*. [56] suggested that the LTPs might facilitate processes such as membrane biosynthesis, cell expansion and polar differentiation that are likely to be limiting factors during somatic embryogenesis. This could explain the abundance of the LTP genes in EC/EMB, especially since embryogenic tissues are actively dividing and differentiating [7]. However, it is important to note that LTPs belong to a diverse family of genes and additional research is necessary to fully characterize the LTPs identified.

Genes that are involved in stress response were also investigated, since the GO classification results showed an increase in the abundance of these genes in EC/EMB. This is in line with previous studies, which have shown that stress can act as a trigger to induce embryogenesis [57-59] and the frequency of stress response genes increased with time during callus development [60]. Three stress response genes *i.e*. catalase 2, defensin EGAD1 and dehydrin-like protein were selected for northern blot analysis (Figure 4a).

The results showed that the expression of catalase 2 is higher in EC/EMB compared with NEC. Papadakis *et al. *[61] showed that totipotent tobacco protoplast had two- and seven-fold lower contents of intra- and extracellular O_2_^- ^and H_2_O_2 _compared to non-totipotent protoplast cells, which suggest that suppression of totipotency was correlated with reduced activity of the cellular antioxidant machinery. As catalases are important components in detoxifying the level of reactive oxygen species and has a high affinity to H_2_O_2_, the expression level observed in the northern blot results seems to support this theory. The results do suggest that the increase in catalase gene expression was probably due to the stresses and reactive compounds generated during tissue culture. Although the effects of the oil palm catalase 2 towards reactive compounds were not tested, detoxification seems to be an important step in achieving embryogenesis.

Northern blot analysis was also performed on EGAD1 and a dehydrin-like protein. The expression profile of the EGAD1 gene was similar to that reported previously by Tregear *et al. *[62] who observed that the transcript was detected at different stages during the *in vitro *process. More importantly, the EGAD1 transcript accumulates in greater quantities in callus cultures initiated from mantled palms, compared to normal palms [62]. In this study, EGAD1 showed significantly high levels of expression in EC and EMB. There was hardly any expression detected in NEC and the other tissues, which indicate that apart from being a marker for somaclonal variation events, it could also be predictive for embryogenesis. The dehydrin-like protein on the other hand, showed stronger signal in EMB tissues, weak signal in EC, but expression was not detected in NEC. This expression profile is in line with the digital northern data reported by Ho *et al. *[15] that showed a dehydrin-like protein expressed at a high level in zygotic embryo, low level in suspension cell culture and was not detected in other tissues. The northern result also showed that the gene appeared to be genotype dependent, as it was more prominent in Set 2 compared to Set 1.

The expression of a gene (cn0544) that had significant similarity with an auxin-regulated glutathione S-transferase (GST) was also investigated. In tissue culture, auxins are important growth regulators that are involved in the initiation of somatic embryogenesis [63]. GSTs are shown to be expressed in cultured leaves of chicory embryogenic cultivar forming somatic embryos [64] and been associated with somatic embryo formation in carrot [65]. However, the digital northern and northern blot results (Figure 4b) showed that the oil palm GST was abundantly present in the NEC library. This is not surprising as GSTs are represented by a large and diverse gene family that can be divided into phi, tau, theta, zeta and lambda classes [66]. The differences in gene expression imply that different GSTs are regulated differently at different stages of tissue culture and various tissues.

Nevertheless, *in silico *EST data analysis and real-time RT-PCR experiments in *Cichorium intybus *showed similar trends to the oil palm GST. The study identified two GSTs preferentially expressed in cultured explants from a non-embryogenic genotype [16]. The results were further supported by the repression of a GST gene in lines that had good callus proliferation [67] and the down-regulation of GST expression in the adaxial side of cotyledons after 14 days in culture [68]. The adaxial side of the cotyledon is the region where somatic embryos develop. However, although the expression of the oil palm GST was higher in NEC in Set 1 and Set 2, the level of GST expression in Set 2 was higher than Set 1. This indicates that the expression level is genotype dependent.

Digital northern analysis was also performed on cn2945, which showed a higher level of expression in NEC compared to EC/EMB. Although the clone had no significant hit, analysis of cn2945 showed that the gene had a signal peptide and transmembrane motif, indicating that it is likely to be translocated across the membrane lipid bilayer. However, the expression profile observed was also genotype dependent, as the difference in expression level was more prominent in Set 2 and not in Set 1.

Overall, the fact that the selected clones (cn1673, cn2100, cn2473, cn3157, cn3238, cn0544 and cn2945) were found to show higher expression in either EC/EMB or NEC suggests a possible association between these genes and embryogenesis at this stage in the tissue culture process. However, the expression of these genes needs further validation, especially on earlier stages of embryogenic tissues such as proembryogenic masses and primary embryogenic callus to determine their applicability in predicting embryogenesis in oil palm.

## Conclusion

The EST data reported here represents an overview of genes expressed during oil palm tissue culture. From the sequencing effort, 9,584 putative unigenes were identified. A total of 211 putative full length ORFs were also identified. This probably represents the most comprehensive list of full-length ORFs reported for oil palm. The base preference of *Elaeis guineensis *was determined to help predict protein-coding regions in the oil palm genome. The EST collection also proved to be a valuable source of SSR markers, as 648 EST-SSRs were identified. The identification of SSRs will go a long way in the development of molecular markers for the purpose of marker-assisted selection in oil palm breeding. The fact that the markers are derived from genic regions increases their usefulness, especially in looking for markers with important economic traits using the candidate gene approach. The work reported here also identified genes that were differentially expressed in NEC and EC/EMB tissues. The expression pattern of some of these genes were confirmed via northern blot analysis, which showed that they could be potential candidates for development as embryogenesis markers, although the expression profiles for some of these genes appear to be genotype dependent. However, if the markers are used together, the predictive power of the genes would increase. The collection of genes are currently being used as molecular markers (either as SSR or restriction fragment length polymorphism [RFLP]) for genetic mapping, where one of the major aim is to identify quantitative trait loci (QTLs) associated with callogenesis and embryogenesis in a mapping population. The most immediate application of the ESTs reported in this study is the development of an oil palm cDNA microarray. The present dataset also effectively compliments the oil palm EST collections reported previously, and contributes to almost half of the current collection of oil palm sequences available in the public databases. Although the combined dataset available currently for oil palm (about 35,000) is not even close to the number of sequences available for some model crops, they nevertheless represent a large enough resource to identify candidate genes for functional studies that will help improve the understanding of the various processes in oil palm and provide the molecular handles to improve important processes, such as oil palm tissue culture.

## Methods

### Plant materials

Two lines of embryogenic callus (EC), three lines of non-embryogenic callus (NEC) and two lines of embryoids (EMB) were used for cDNA library construction. These leaf-derived tissue culture materials were obtained from two laboratories. Data validation was carried out using two sets of tissue culture samples, each set represented by NEC, EC and EMB. In addition to the tissue culture samples, spear leaf, inflorescences frond-12 (female, 5 cm), 12 weeks after antheses (12 WAA) kernel and 15 WAA mesocarp tissues obtained from Malaysian Palm Oil Board (MPOB), Malaysia were also used for the validation experiments. All plant materials were of the *Dura *(D) × *Pisifera *(P) fruit type and stored at -80°C prior to RNA extraction.

### Construction of cDNA libraries

A total of 12 cDNA libraries were constructed from different developmental stages of the tissue culture process: EC, NEC and EMB. Total RNA was isolated using the aqueous phenol extraction method as described by Rochester *et al*. [69]. Poly (A)^+ ^RNA was isolated using oligo-dT cellulose chromatography according to Singh and Cheah [70]. Directional cDNA libraries were constructed using the ZAP-cDNA^® ^Synthesis Kit and the ZAP-cDNA^® ^Gigapack^® ^III Gold Cloning Kit (Stratagene USA) according to the manufacturer's instruction.

### Sequencing of cDNA libraries

For DNA sequencing, phagemids and plasmids were used as templates. Individual recombinant phages were selected randomly for sequencing. Inserts from phages were amplified using T3 and T7 primers. Enzymatic purification was performed on the PCR products by using Shrimp Alkaline Phosphatase (GE Biosciences USA) and Exonuclease 1 (GE Biosciences USA) to clean up excess dNTPs and primers. Plasmid clones were obtained either by single or mass *in vivo *excision using ExAssist^® ^Helper phage as recommended by the manufacturer (Stratagene USA). Plasmid DNA for sequencing was prepared using Wizard^® ^Plus Minipreps DNA Purification System kit (Promega USA). cDNA inserts were sequenced from the 5' end with SK primer using the ABI PRISM™ Ready Reaction BigDye™ Terminator Cycle Sequencing Kit (Applied Biosystems USA). Sequencing was performed on an ABI 377 automated DNA sequencer (Applied Biosystem USA).

### Sequence processing, clustering, BLAST search and annotation

All the sequences were quality checked before clustering and deposited into GenBank. Raw ABI-formatted chromatogram reads were base-called using PHRED (version 0.020425.c) [71,72] with a threshold value of 20. Crossmatch (version 0.990329) and customized Perl scripts were used to trim vectors, adaptors, polyA-ends and low quality nucleotides. ESTs were also filtered for prokaryotic microbe such as *Escherichia coli*. Only high quality ESTs with a minimum of 100 bases and fewer than 4% Ns were retained. Manual processing was also performed to confirm the results.

Multiple sequence alignment, clustering, assembling and the generation of consensus were accomplished by StackPACK [29]. StackPACK contains d2_cluster [73], where sequences were grouped together if there were at least 96% sequence similarity in any window of 150 bases. The loose clusters were then aligned using PHRAP [74] and subsequently, CRAW [75]. The resulting set of consensi and singletons were considered as a set of putative unique genes (unigenes). Homology searches of all cleaned ESTs and assembled consensus sequences were compared to GenBank by using the blastx algorithm. Sequences with blastx E-value > 10^-10 ^were designated as having "no significant similarity".

Digital analysis of gene expression was performed with IDEG6 [35] by using transcript abundance in each consensus gathered from the EST frequency for that consensus in all three tissue types (NEC, EC and EMB). The resultant gene list was clustered in TIGR Multiple Experiment Viewer software (version 3.0).

### In silico identification of simple sequence repeat (SSR) markers

The unigenes assembled from 17,599 ESTs were mined for SSR markers. The polyA and polyT sequences in a 50 bp window at the terminal regions were removed. The unigenes were mined for microsatellite motifs. MISA (*MicroSAtellite identification tool*) search module [76] was used to identify and localize the microsatellite motifs. Sequences were deemed to contain microsatellite motifs if it contains ten, seven and five repeat units of mononucleotides, dinucleotides and higher-order repeats respectively. The Perl scripts provided by the MISA website also generate summary files with information such as the position and type of microsatellites identified, distribution of different repeat type classes and frequencies of identified SSR motifs.

### Assignment of GO terms

The 17,599 ESTs were annotated with gene ontology terms using Blast2GO [30]. The sequences were annotated using blastx search to the GenBank non-redundant database. The top 20 hits were evaluated and blastx results that had an E-value cutoff of 1e^-6 ^and a minimum similarity of 55% were annotated with GO terms. A weightage based on the default Evidence Code Weights was also used to determine the GO terms annotated. Additional information and GO terms were obtained by comparing the sequences to the InterPro database using the InterProScan tool to identify protein signatures [77]. The GO terms were compared and visualized using WEGO [31].

### Northern blot analysis

Total RNAs (20 μg) were separated on a 1.0% (w/v) agarose/formaldehyde gel and transferred to positively charged nylon membranes (Hybond™-N^+^, GE Healthcare Biosciences, UK), by downward capillary transfer with NorthernMax™ kit transfer buffer (Ambion Inc., USA). Specific probes labelled with α^32^P-dCTP were generated by purified PCR amplification of the relevant differentially expressed clones. After a 3-hour prehybridization step performed in ULTRAhyb™ (Ambion Inc., USA) and hybridization in the same solution for overnight at 65°C, the membrane was washed twice in 2× saline sodium phosphate EDTA (SSPE) (0.36 M sodium chloride, 20 mM sodium hydrogen phosphate, 2 mM EDTA) pH 7.4 at room temperature for 5 min and then was washed in 0.1× SSPE at 65°C for 15 min. The blot was exposed at -80°C with an intensifying screen overnight or up to 1 week.

## Authors' contributions

E–TLL, HA, C–YAT and LCLO constructed the cDNA libraries and generated the ESTs. S–HB and EMS were also involved in EST sequencing. HA and E–TLL set-up the sequence analysis system. E–TLL, S–HB, HA and EMS analyzed the ESTs. LCLO maintained the EST clones. S–HB performed the northern blot analysis. E–TLL, HA and S–HB drafted the manuscript. S–CC, K–LW and RS participated in the design of the study. S–CC, A–RR, K–LW and RS supervised and coordinated the study and critically revised the manuscript. All authors read and approved the final manuscript.

## Supplementary Material

Additional file 1 Assembly results of 17,599 ESTs isolated from non-embryogenic callus, embryogenic callus and embryoid cDNA libraries. Click here for file
