# Supplementary material for: Oil palm (Elaeis guineensis Jacq.) tissue culture ESTs: Identifying genes associated with callogenesis and embryogenesis
Source: BMC Plant Biol. 2008 May 29;8:62. doi: 10.1186/1471-2229-8-62 (PMC2442076; doi:10.1186/1471-2229-8-62)
Supplement: Additional file 1 — Assembly results of 17,599 ESTs isolated from non-embryogenic callus, embryogenic callus and embryoid cDNA libraries. [file 1471-2229-8-62-S1.pdf]

Additional File 1: Assembly results of 17,599 ESTs isolated from non-embryogenic callus, embryogenic callus and embryoid cDNA libraries

Cluster ID	Contig ID	Consensus ID	EST ID	Length (nt)
cl0001	ct0001	cn0001	pOP-CEO01278	347
cl0001	ct0001	cn0001	pOP-EO07275_EST_C_1_pSK_SK	654
cl0002	ct0002	cn0002	pOP-CEO01327	290
cl0002	ct0002	cn0002	pOP-CNI01953_EST_C_1_pSK_SK	406
cl0003	ct0003	cn0003	pOP-CNH04151	443
cl0003	ct0003	cn0003	pOP-CNI01754_EST_C_1_pSK_SK	544
cl0004	ct0004	cn0004	pOP-CNH04216	514
cl0004	ct0004	cn0004	pOP-CNH04221	575
cl0005	ct0005	cn0005	pOP-CNH04262	604
cl0005	ct0006	cn0006	pOP-CNH02565_EST_C_1_pSK_SK	542
cl0005	ct0006	cn0006	pOP-CNH03380_EST_C_1_pSK_SK	562
cl0005	ct0006	cn0006	pOP-CNH04379	563
cl0005	ct0006	cn0006	pOP-CNH04778_EST_C_1_pSK_SK	526
cl0006	ct0007	cn0007	pOP-CNH04365	855
cl0006	ct0008	cn0008	pOP-CNH01019_EST_C_1_pSK_SK	342
cl0006	ct0008	cn0008	pOP-CNH01813_EST_C_1_pSK_SK	449
cl0006	ct0008	cn0008	pOP-CNH02537_EST_C_1_pSK_SK	447
cl0006	ct0008	cn0008	pOP-CNH02613_EST_C_1_pSK_SK	673
cl0006	ct0009	cn0009	pOP-CNH02090_EST_C_1_pSK_SK	477
cl0006	ct0009	cn0009	pOP-CNH04237	539
cl0006	ct0009	cn0009	pOP-CNH04313	522
cl0006	ct0009	cn0009	pOP-CNH04549	691
cl0006	ct0009	cn0009	pOP-CNH04605	559
cl0006	ct0010	cn0010	pOP-CNH01104_EST_C_1_pSK_SK	143
cl0006	ct0010	cn0010	pOP-CNH01635_EST_C_1_pSK_SK	446
cl0006	ct0010	cn0010	pOP-CNH02074_EST_C_1_pSK_SK	710
cl0006	ct0010	cn0010	pOP-CNH02191_EST_C_1_pSK_SK	300
cl0006	ct0010	cn0010	pOP-CNH03309_EST_C_1_pSK_SK	365
cl0006	ct0010	cn0010	pOP-CNH03652_EST_C_1_pSK_SK	430
cl0006	ct0010	cn0010	pOP-CNH04175	589
cl0006	ct0010	cn0010	pOP-CNH04397	806
cl0006	ct0010	cn0010	pOP-CNH04419	448
cl0006	ct0010	cn0010	pOP-CNH04882_EST_C_1_pSK_SK	647
cl0006	ct0010	cn0010	pOP-CNH05039_EST_C_1_pSK_SK	447
cl0006	ct0010	cn0010	pOP-CNI02138_EST_C_1_pSK_SK	571
cl0006	ct0011	cn0011	pOP-CEO03450_EST_C_1_pSK_SK	439
cl0006	ct0011	cn0011	pOP-CEO03533_EST_C_1_pSK_SK	712
cl0006	ct0011	cn0011	pOP-CNH01251_EST_C_1_pSK_SK	546
cl0006	ct0011	cn0011	pOP-CNH01312_EST_C_1_pSK_SK	497
cl0006	ct0011	cn0011	pOP-CNH01639_EST_C_1_pSK_SK	569
cl0006	ct0011	cn0011	pOP-CNH01725_EST_C_1_pSK_SK	613
cl0006	ct0011	cn0011	pOP-CNH01809_EST_C_1_pSK_SK	552
cl0006	ct0011	cn0011	pOP-CNH01911_EST_C_1_pSK_SK	536
cl0006	ct0011	cn0011	pOP-CNH01934_EST_C_1_pSK_SK	480
cl0006	ct0011	cn0011	pOP-CNH02585_EST_C_1_pSK_SK	549
cl0006	ct0011	cn0011	pOP-CNH02839_EST_C_1_pSK_SK	645
cl0006	ct0011	cn0011	pOP-CNH04274	561
cl0006	ct0011	cn0011	pOP-CNH04976_EST_C_1_pSK_SK	327
cl0007	ct0012	cn0012	pOP-CNH04222	581
cl0007	ct0012	cn0012	pOP-CNH04390	838
cl0008	ct0013	cn0013	pOP-CNH04398	719
cl0008	ct0013	cn0013	pOP-CNH04404	806

cl0009	ct0014	cn0014	pOP-CEO02083_EST_C_1_pSK_SK	457
cl0009	ct0014	cn0014	pOP-CNH04492	791
cl0010	ct0015	cn0015	pOP-CNH04572	578
cl0010	ct0015	cn0015	pOP-EO02695_EST_C_1_pSK_SK	400
cl0011	ct0016	cn0016	pOP-CNH04449	767
cl0011	ct0016	cn0016	pOP-CNH04591	737
cl0012	ct0017	cn0017	pOP-CNH04604	682
cl0012	ct0017	cn0017	pOP-CNH04630	678
cl0013	ct0018	cn0018	pOP-CNH02538_EST_C_1_pSK_SK	545
cl0013	ct0018	cn0018	pOP-CNH04660	486
cl0014	ct0019	cn0019	pOP-CNH04254	614
cl0014	ct0019	cn0019	pOP-CNH04700	495
cl0015	ct0020	cn0020	pOP-CNH00560_EST_C_1_pSK_SK	645
cl0015	ct0020	cn0020	pOP-EO04624_EST_C_1_pSK_SK	482
cl0016	ct0021	cn0021	pOP-CNH00579_EST_C_1_pSK_SK	643
cl0016	ct0021	cn0021	pOP-EO07386_EST_C_1_pSK_SK	712
cl0017	ct0022	cn0022	pOP-CNH00585_EST_C_1_pSK_SK	586
cl0017	ct0022	cn0022	pOP-CNH04166	521
cl0017	ct0022	cn0022	pOP-CNH04382	606
cl0017	ct0022	cn0022	pOP-CNH04384	606
cl0018	ct0023	cn0023	pOP-CNH00591_EST_C_1_pSK_SK	410
cl0018	ct0023	cn0023	pOP-CNH00601_EST_C_1_pSK_SK	405
cl0019	ct0024	cn0024	pOP-CNH00621_EST_C_1_pSK_SK	602
cl0019	ct0024	cn0024	pOP-CNH00682_EST_C_1_pSK_SK	521
cl0020	ct0025	cn0025	pOP-CNH00789_EST_C_1_pSK_SK	447
cl0020	ct0025	cn0025	pOP-CNH00814_EST_C_1_pSK_SK	504
cl0021	ct0026	cn0026	pOP-CNH00843_EST_C_1_pSK_SK	620
cl0021	ct0026	cn0026	pOP-CNH00865_EST_C_1_pSK_SK	575
cl0022	ct0027	cn0027	pOP-CEO01825_EST_C_1_pSK_SK	139
cl0022	ct0027	cn0027	pOP-CEO03289_EST_C_1_pSK_SK	483
cl0022	ct0027	cn0027	pOP-CNH00922_EST_C_1_pSK_SK	507
cl0022	ct0027	cn0027	pOP-CNH02450_EST_C_1_pSK_SK	537
cl0022	ct0027	cn0027	pOP-CNH04530	343
cl0022	ct0027	cn0027	pOP-EAP01470_EST_C_1_pBSK_SK	629
cl0023	ct0028	cn0028	pOP-CNH01070_EST_C_1_pSK_SK	635
cl0023	ct0028	cn0028	pOP-CNH04443	677
cl0024	ct0029	cn0029	pOP-CEO03756_EST_C_1_pSK_SK	485
cl0024	ct0029	cn0029	pOP-CNH01071_EST_C_1_pSK_SK	732
cl0025	ct0030	cn0030	pOP-CNH01102_EST_C_1_pSK_SK	299
cl0025	ct0030	cn0030	pOP-CNH04553	791
cl0025	ct0030	cn0030	pOP-CNH04609	736
cl0026	ct0031	cn0031	pOP-CNH01147_EST_C_1_pSK_SK	430
cl0026	ct0031	cn0031	pOP-CNH04617	736
cl0027	ct0032	cn0032	pOP-CNH01176_EST_C_1_pSK_SK	293
cl0027	ct0032	cn0032	pOP-CNH04285	289
cl0027	ct0032	cn0032	pOP-CNH04331	286
cl0027	ct0032	cn0032	pOP-CNH04438	288
cl0028	ct0033	cn0033	pOP-CNH00856_EST_C_1_pSK_SK	442
cl0028	ct0033	cn0033	pOP-CNH01193_EST_C_1_pSK_SK	530
cl0028	ct0033	cn0033	pOP-CNH04622	749
cl0029	ct0034	cn0034	pOP-CNH00561_EST_C_1_pSK_SK	628
cl0029	ct0034	cn0035	pOP-CNH01205_EST_C_1_pSK_SK	583
cl0030	ct0035	cn0036	pOP-EO06282_EST_C_1_pSK_SK	293
cl0030	ct0035	cn0036	pOP-EO06941_EST_C_1_pSK_SK	293
cl0030	ct0036	cn0037	pOP-CEM00066_EST_C_1_pSK_SK	404
cl0030	ct0036	cn0037	pOP-CEO00591_EST_C_1_pSK_SK	647

cl0030	ct0036	cn0037	pOP-CEO00719_EST_C_1_pSK_SK	166
cl0030	ct0036	cn0037	pOP-CEO00950_EST_C_1_pSK_SK	704
cl0030	ct0036	cn0037	pOP-CEO01276	719
cl0030	ct0036	cn0037	pOP-CEO01628_EST_C_1_pSK_SK	478
cl0030	ct0036	cn0037	pOP-CEO01669_EST_C_1_pSK_SK	541
cl0030	ct0036	cn0037	pOP-CEO01943_EST_C_1_pSK_SK	346
cl0030	ct0036	cn0037	pOP-CEO02176_EST_C_1_pSK_SK	293
cl0030	ct0036	cn0037	pOP-CEO02523_EST_C_1_pSK_SK	206
cl0030	ct0036	cn0037	pOP-CEO02833_EST_C_1_pSK_SK	532
cl0030	ct0036	cn0037	pOP-CEO02995_EST_C_1_pSK_SK	796
cl0030	ct0036	cn0037	pOP-CEO03067_EST_C_1_pSK_SK	300
cl0030	ct0036	cn0037	pOP-CEO03153_EST_C_1_pSK_SK	346
cl0030	ct0036	cn0037	pOP-CEO03266_EST_C_1_pSK_SK	781
cl0030	ct0036	cn0037	pOP-CEO03299_EST_C_1_pSK_SK	648
cl0030	ct0036	cn0037	pOP-CEO03330_EST_C_1_pSK_SK	270
cl0030	ct0036	cn0037	pOP-CEO03346_EST_C_1_pSK_SK	568
cl0030	ct0036	cn0037	pOP-CEO03384_EST_C_1_pSK_SK	571
cl0030	ct0036	cn0037	pOP-CEO03440_EST_C_1_pSK_SK	638
cl0030	ct0036	cn0037	pOP-CNH00639_EST_C_1_pSK_SK	699
cl0030	ct0036	cn0037	pOP-CNH00652_EST_C_1_pSK_SK	423
cl0030	ct0036	cn0037	pOP-CNH00692_EST_C_1_pSK_SK	463
cl0030	ct0036	cn0037	pOP-CNH00859_EST_C_1_pSK_SK	523
cl0030	ct0036	cn0037	pOP-CNH00871_EST_C_1_pSK_SK	417
cl0030	ct0036	cn0037	pOP-CNH00953_EST_C_1_pSK_SK	643
cl0030	ct0036	cn0037	pOP-CNH00977_EST_C_1_pSK_SK	512
cl0030	ct0036	cn0037	pOP-CNH00999_EST_C_1_pSK_SK	365
cl0030	ct0036	cn0037	pOP-CNH01010_EST_C_1_pSK_SK	327
cl0030	ct0036	cn0037	pOP-CNH01021_EST_C_1_pSK_SK	316
cl0030	ct0036	cn0037	pOP-CNH01051_EST_C_1_pSK_SK	234
cl0030	ct0036	cn0037	pOP-CNH01052_EST_C_1_pSK_SK	593
cl0030	ct0036	cn0037	pOP-CNH01074_EST_C_1_pSK_SK	377
cl0030	ct0036	cn0037	pOP-CNH01078_EST_C_1_pSK_SK	388
cl0030	ct0036	cn0037	pOP-CNH01084_EST_C_1_pSK_SK	263
cl0030	ct0036	cn0037	pOP-CNH01096_EST_C_1_pSK_SK	255
cl0030	ct0036	cn0037	pOP-CNH01101_EST_C_1_pSK_SK	305
cl0030	ct0036	cn0037	pOP-CNH01106_EST_C_1_pSK_SK	294
cl0030	ct0036	cn0037	pOP-CNH01136_EST_C_1_pSK_SK	408
cl0030	ct0036	cn0037	pOP-CNH01141_EST_C_1_pSK_SK	397
cl0030	ct0036	cn0037	pOP-CNH01162_EST_C_1_pSK_SK	418
cl0030	ct0036	cn0037	pOP-CNH01164_EST_C_1_pSK_SK	385
cl0030	ct0036	cn0037	pOP-CNH01168_EST_C_1_pSK_SK	446
cl0030	ct0036	cn0037	pOP-CNH01172_EST_C_1_pSK_SK	469
cl0030	ct0036	cn0037	pOP-CNH01196_EST_C_1_pSK_SK	580
cl0030	ct0036	cn0037	pOP-CNH01203_EST_C_1_pSK_SK	452
cl0030	ct0036	cn0037	pOP-CNH01222_EST_C_1_pSK_SK	554
cl0030	ct0036	cn0037	pOP-CNH01223_EST_C_1_pSK_SK	592
cl0030	ct0036	cn0037	pOP-CNH01228_EST_C_1_pSK_SK	599
cl0030	ct0036	cn0037	pOP-CNH01231_EST_C_1_pSK_SK	313
cl0030	ct0036	cn0037	pOP-CNH01235_EST_C_1_pSK_SK	395
cl0030	ct0036	cn0037	pOP-CNH01241_EST_C_1_pSK_SK	415
cl0030	ct0036	cn0037	pOP-CNH01254_EST_C_1_pSK_SK	515
cl0030	ct0036	cn0037	pOP-CNH01260_EST_C_1_pSK_SK	423
cl0030	ct0036	cn0037	pOP-CNH01268_EST_C_1_pSK_SK	423
cl0030	ct0036	cn0037	pOP-CNH01279_EST_C_1_pSK_SK	734
cl0030	ct0036	cn0037	pOP-CNH01280_EST_C_1_pSK_SK	359
cl0030	ct0036	cn0037	pOP-CNH01285_EST_C_1_pSK_SK	401

cl0030	ct0036	cn0037	pOP-CNH01305_EST_C_1_pSK_SK	414
cl0030	ct0036	cn0037	pOP-CNH01321_EST_C_1_pSK_SK	702
cl0030	ct0036	cn0037	pOP-CNH01333_EST_C_1_pSK_SK	643
cl0030	ct0036	cn0037	pOP-CNH01351_EST_C_1_pSK_SK	572
cl0030	ct0036	cn0037	pOP-CNH01352_EST_C_1_pSK_SK	559
cl0030	ct0036	cn0037	pOP-CNH01360_EST_C_1_pSK_SK	700
cl0030	ct0036	cn0037	pOP-CNH01363_EST_C_1_pSK_SK	599
cl0030	ct0036	cn0037	pOP-CNH01366_EST_C_1_pSK_SK	533
cl0030	ct0036	cn0037	pOP-CNH01397_EST_C_1_pSK_SK	426
cl0030	ct0036	cn0037	pOP-CNH01419_EST_C_1_pSK_SK	524
cl0030	ct0036	cn0037	pOP-CNH01433_EST_C_1_pSK_SK	515
cl0030	ct0036	cn0037	pOP-CNH01435_EST_C_1_pSK_SK	524
cl0030	ct0036	cn0037	pOP-CNH01440_EST_C_1_pSK_SK	504
cl0030	ct0036	cn0037	pOP-CNH01456_EST_C_1_pSK_SK	367
cl0030	ct0036	cn0037	pOP-CNH01465_EST_C_1_pSK_SK	711
cl0030	ct0036	cn0037	pOP-CNH01477_EST_C_1_pSK_SK	603
cl0030	ct0036	cn0037	pOP-CNH01481_EST_C_1_pSK_SK	423
cl0030	ct0036	cn0037	pOP-CNH01495_EST_C_1_pSK_SK	510
cl0030	ct0036	cn0037	pOP-CNH01523_EST_C_1_pSK_SK	423
cl0030	ct0036	cn0037	pOP-CNH01546_EST_C_1_pSK_SK	337
cl0030	ct0036	cn0037	pOP-CNH01547_EST_C_1_pSK_SK	455
cl0030	ct0036	cn0037	pOP-CNH01555_EST_C_1_pSK_SK	638
cl0030	ct0036	cn0037	pOP-CNH01586_EST_C_1_pSK_SK	683
cl0030	ct0036	cn0037	pOP-CNH01627_EST_C_1_pSK_SK	618
cl0030	ct0036	cn0037	pOP-CNH01634_EST_C_1_pSK_SK	614
cl0030	ct0036	cn0037	pOP-CNH01644_EST_C_1_pSK_SK	558
cl0030	ct0036	cn0037	pOP-CNH01660_EST_C_1_pSK_SK	584
cl0030	ct0036	cn0037	pOP-CNH01675_EST_C_1_pSK_SK	684
cl0030	ct0036	cn0037	pOP-CNH01677_EST_C_1_pSK_SK	545
cl0030	ct0036	cn0037	pOP-CNH01680_EST_C_1_pSK_SK	589
cl0030	ct0036	cn0037	pOP-CNH01700_EST_C_1_pSK_SK	557
cl0030	ct0036	cn0037	pOP-CNH01704_EST_C_1_pSK_SK	423
cl0030	ct0036	cn0037	pOP-CNH01729_EST_C_1_pSK_SK	663
cl0030	ct0036	cn0037	pOP-CNH01761_EST_C_1_pSK_SK	509
cl0030	ct0036	cn0037	pOP-CNH01764_EST_C_1_pSK_SK	517
cl0030	ct0036	cn0037	pOP-CNH01776_EST_C_1_pSK_SK	379
cl0030	ct0036	cn0037	pOP-CNH01780_EST_C_1_pSK_SK	423
cl0030	ct0036	cn0037	pOP-CNH01797_EST_C_1_pSK_SK	502
cl0030	ct0036	cn0037	pOP-CNH01810_EST_C_1_pSK_SK	450
cl0030	ct0036	cn0037	pOP-CNH01816_EST_C_1_pSK_SK	461
cl0030	ct0036	cn0037	pOP-CNH01825_EST_C_1_pSK_SK	477
cl0030	ct0036	cn0037	pOP-CNH01830_EST_C_1_pSK_SK	423
cl0030	ct0036	cn0037	pOP-CNH01833_EST_C_1_pSK_SK	469
cl0030	ct0036	cn0037	pOP-CNH01842_EST_C_1_pSK_SK	411
cl0030	ct0036	cn0037	pOP-CNH01844_EST_C_1_pSK_SK	445
cl0030	ct0036	cn0037	pOP-CNH01845_EST_C_1_pSK_SK	512
cl0030	ct0036	cn0037	pOP-CNH01851_EST_C_1_pSK_SK	493
cl0030	ct0036	cn0037	pOP-CNH01863_EST_C_1_pSK_SK	330
cl0030	ct0036	cn0037	pOP-CNH01864_EST_C_1_pSK_SK	423
cl0030	ct0036	cn0037	pOP-CNH01867_EST_C_1_pSK_SK	365
cl0030	ct0036	cn0037	pOP-CNH01871_EST_C_1_pSK_SK	291
cl0030	ct0036	cn0037	pOP-CNH01877_EST_C_1_pSK_SK	200
cl0030	ct0036	cn0037	pOP-CNH01878_EST_C_1_pSK_SK	445
cl0030	ct0036	cn0037	pOP-CNH01900_EST_C_1_pSK_SK	619
cl0030	ct0036	cn0037	pOP-CNH01914_EST_C_1_pSK_SK	645
cl0030	ct0036	cn0037	pOP-CNH01926_EST_C_1_pSK_SK	629

cl0030	ct0036	cn0037	pOP-CNH01935_EST_C_1_pSK_SK	423
cl0030	ct0036	cn0037	pOP-CNH01937_EST_C_1_pSK_SK	600
cl0030	ct0036	cn0037	pOP-CNH01951_EST_C_1_pSK_SK	602
cl0030	ct0036	cn0037	pOP-CNH01972_EST_C_1_pSK_SK	640
cl0030	ct0036	cn0037	pOP-CNH01986_EST_C_1_pSK_SK	395
cl0030	ct0036	cn0037	pOP-CNH02006_EST_C_1_pSK_SK	354
cl0030	ct0036	cn0037	pOP-CNH02009_EST_C_1_pSK_SK	609
cl0030	ct0036	cn0037	pOP-CNH02014_EST_C_1_pSK_SK	424
cl0030	ct0036	cn0037	pOP-CNH02017_EST_C_1_pSK_SK	316
cl0030	ct0036	cn0037	pOP-CNH02028_EST_C_1_pSK_SK	625
cl0030	ct0036	cn0037	pOP-CNH02032_EST_C_1_pSK_SK	603
cl0030	ct0036	cn0037	pOP-CNH02042_EST_C_1_pSK_SK	354
cl0030	ct0036	cn0037	pOP-CNH02050_EST_C_1_pSK_SK	457
cl0030	ct0036	cn0037	pOP-CNH02051_EST_C_1_pSK_SK	423
cl0030	ct0036	cn0037	pOP-CNH02060_EST_C_1_pSK_SK	417
cl0030	ct0036	cn0037	pOP-CNH02077_EST_C_1_pSK_SK	550
cl0030	ct0036	cn0037	pOP-CNH02087_EST_C_1_pSK_SK	423
cl0030	ct0036	cn0037	pOP-CNH02094_EST_C_1_pSK_SK	514
cl0030	ct0036	cn0037	pOP-CNH02095_EST_C_1_pSK_SK	541
cl0030	ct0036	cn0037	pOP-CNH02104_EST_C_1_pSK_SK	291
cl0030	ct0036	cn0037	pOP-CNH02117_EST_C_1_pSK_SK	423
cl0030	ct0036	cn0037	pOP-CNH02127_EST_C_1_pSK_SK	381
cl0030	ct0036	cn0037	pOP-CNH02144_EST_C_1_pSK_SK	491
cl0030	ct0036	cn0037	pOP-CNH02164_EST_C_1_pSK_SK	560
cl0030	ct0036	cn0037	pOP-CNH02165_EST_C_1_pSK_SK	524
cl0030	ct0036	cn0037	pOP-CNH02168_EST_C_1_pSK_SK	371
cl0030	ct0036	cn0037	pOP-CNH02176_EST_C_1_pSK_SK	609
cl0030	ct0036	cn0037	pOP-CNH02258_EST_C_1_pSK_SK	510
cl0030	ct0036	cn0037	pOP-CNH02259_EST_C_1_pSK_SK	562
cl0030	ct0036	cn0037	pOP-CNH02272_EST_C_1_pSK_SK	652
cl0030	ct0036	cn0037	pOP-CNH02276_EST_C_1_pSK_SK	492
cl0030	ct0036	cn0037	pOP-CNH02375_EST_C_1_pSK_SK	407
cl0030	ct0036	cn0037	pOP-CNH02444_EST_C_1_pSK_SK	417
cl0030	ct0036	cn0037	pOP-CNH02457_EST_C_1_pSK_SK	400
cl0030	ct0036	cn0037	pOP-CNH02469_EST_C_1_pSK_SK	393
cl0030	ct0036	cn0037	pOP-CNH02490_EST_C_1_pSK_SK	192
cl0030	ct0036	cn0037	pOP-CNH02515_EST_C_1_pSK_SK	356
cl0030	ct0036	cn0037	pOP-CNH02527_EST_C_1_pSK_SK	477
cl0030	ct0036	cn0037	pOP-CNH02528_EST_C_1_pSK_SK	330
cl0030	ct0036	cn0037	pOP-CNH02532_EST_C_1_pSK_SK	291
cl0030	ct0036	cn0037	pOP-CNH02540_EST_C_1_pSK_SK	539
cl0030	ct0036	cn0037	pOP-CNH02570_EST_C_1_pSK_SK	523
cl0030	ct0036	cn0037	pOP-CNH02573_EST_C_1_pSK_SK	539
cl0030	ct0036	cn0037	pOP-CNH02586_EST_C_1_pSK_SK	559
cl0030	ct0036	cn0037	pOP-CNH02587_EST_C_1_pSK_SK	395
cl0030	ct0036	cn0037	pOP-CNH02610_EST_C_1_pSK_SK	657
cl0030	ct0036	cn0037	pOP-CNH02650_EST_C_1_pSK_SK	348
cl0030	ct0036	cn0037	pOP-CNH02701_EST_C_1_pSK_SK	610
cl0030	ct0036	cn0037	pOP-CNH02723_EST_C_1_pSK_SK	587
cl0030	ct0036	cn0037	pOP-CNH02729_EST_C_1_pSK_SK	712
cl0030	ct0036	cn0037	pOP-CNH02736_EST_C_1_pSK_SK	638
cl0030	ct0036	cn0037	pOP-CNH02748_EST_C_1_pSK_SK	593
cl0030	ct0036	cn0037	pOP-CNH02778_EST_C_1_pSK_SK	577
cl0030	ct0036	cn0037	pOP-CNH02779_EST_C_1_pSK_SK	577
cl0030	ct0036	cn0037	pOP-CNH02781_EST_C_1_pSK_SK	590
cl0030	ct0036	cn0037	pOP-CNH02796_EST_C_1_pSK_SK	514

cl0030	ct0036	cn0037	pOP-CNH02799_EST_C_1_pSK_SK	648
cl0030	ct0036	cn0037	pOP-CNH02821_EST_C_1_pSK_SK	587
cl0030	ct0036	cn0037	pOP-CNH02822_EST_C_1_pSK_SK	423
cl0030	ct0036	cn0037	pOP-CNH02829_EST_C_1_pSK_SK	423
cl0030	ct0036	cn0037	pOP-CNH02837_EST_C_1_pSK_SK	593
cl0030	ct0036	cn0037	pOP-CNH02841_EST_C_1_pSK_SK	594
cl0030	ct0036	cn0037	pOP-CNH02855_EST_C_1_pSK_SK	514
cl0030	ct0036	cn0037	pOP-CNH02886_EST_C_1_pSK_SK	613
cl0030	ct0036	cn0037	pOP-CNH02901_EST_C_1_pSK_SK	423
cl0030	ct0036	cn0037	pOP-CNH02906_EST_C_1_pSK_SK	512
cl0030	ct0036	cn0037	pOP-CNH02962_EST_C_1_pSK_SK	692
cl0030	ct0036	cn0037	pOP-CNH02980_EST_C_1_pSK_SK	423
cl0030	ct0036	cn0037	pOP-CNH02984_EST_C_1_pSK_SK	640
cl0030	ct0036	cn0037	pOP-CNH02998_EST_C_1_pSK_SK	576
cl0030	ct0036	cn0037	pOP-CNH03003_EST_C_1_pSK_SK	566
cl0030	ct0036	cn0037	pOP-CNH03006_EST_C_1_pSK_SK	531
cl0030	ct0036	cn0037	pOP-CNH03012_EST_C_1_pSK_SK	534
cl0030	ct0036	cn0037	pOP-CNH03032_EST_C_1_pSK_SK	609
cl0030	ct0036	cn0037	pOP-CNH03041_EST_C_1_pSK_SK	492
cl0030	ct0036	cn0037	pOP-CNH03045_EST_C_1_pSK_SK	432
cl0030	ct0036	cn0037	pOP-CNH03046_EST_C_1_pSK_SK	320
cl0030	ct0036	cn0037	pOP-CNH03049_EST_C_1_pSK_SK	361
cl0030	ct0036	cn0037	pOP-CNH03065_EST_C_1_pSK_SK	616
cl0030	ct0036	cn0037	pOP-CNH03103_EST_C_1_pSK_SK	367
cl0030	ct0036	cn0037	pOP-CNH03114_EST_C_1_pSK_SK	569
cl0030	ct0036	cn0037	pOP-CNH03119_EST_C_1_pSK_SK	650
cl0030	ct0036	cn0037	pOP-CNH03120_EST_C_1_pSK_SK	577
cl0030	ct0036	cn0037	pOP-CNH03125_EST_C_1_pSK_SK	367
cl0030	ct0036	cn0037	pOP-CNH03131_EST_C_1_pSK_SK	367
cl0030	ct0036	cn0037	pOP-CNH03154_EST_C_1_pSK_SK	419
cl0030	ct0036	cn0037	pOP-CNH03182_EST_C_1_pSK_SK	395
cl0030	ct0036	cn0037	pOP-CNH03187_EST_C_1_pSK_SK	404
cl0030	ct0036	cn0037	pOP-CNH03195_EST_C_1_pSK_SK	395
cl0030	ct0036	cn0037	pOP-CNH03214_EST_C_1_pSK_SK	469
cl0030	ct0036	cn0037	pOP-CNH03227_EST_C_1_pSK_SK	229
cl0030	ct0036	cn0037	pOP-CNH03237_EST_C_1_pSK_SK	438
cl0030	ct0036	cn0037	pOP-CNH03242_EST_C_1_pSK_SK	482
cl0030	ct0036	cn0037	pOP-CNH03243_EST_C_1_pSK_SK	456
cl0030	ct0036	cn0037	pOP-CNH03246_EST_C_1_pSK_SK	508
cl0030	ct0036	cn0037	pOP-CNH03258_EST_C_1_pSK_SK	611
cl0030	ct0036	cn0037	pOP-CNH03259_EST_C_1_pSK_SK	423
cl0030	ct0036	cn0037	pOP-CNH03292_EST_C_1_pSK_SK	576
cl0030	ct0036	cn0037	pOP-CNH03310_EST_C_1_pSK_SK	703
cl0030	ct0036	cn0037	pOP-CNH03338_EST_C_1_pSK_SK	754
cl0030	ct0036	cn0037	pOP-CNH03351_EST_C_1_pSK_SK	423
cl0030	ct0036	cn0037	pOP-CNH03354_EST_C_1_pSK_SK	539
cl0030	ct0036	cn0037	pOP-CNH03416_EST_C_1_pSK_SK	542
cl0030	ct0036	cn0037	pOP-CNH03420_EST_C_1_pSK_SK	530
cl0030	ct0036	cn0037	pOP-CNH03464_EST_C_1_pSK_SK	423
cl0030	ct0036	cn0037	pOP-CNH03508_EST_C_1_pSK_SK	681
cl0030	ct0036	cn0037	pOP-CNH03509_EST_C_1_pSK_SK	675
cl0030	ct0036	cn0037	pOP-CNH03558_EST_C_1_pSK_SK	324
cl0030	ct0036	cn0037	pOP-CNH03586_EST_C_1_pSK_SK	348
cl0030	ct0036	cn0037	pOP-CNH03600_EST_C_1_pSK_SK	423
cl0030	ct0036	cn0037	pOP-CNH03619_EST_C_1_pSK_SK	659
cl0030	ct0036	cn0037	pOP-CNH03641_EST_C_1_pSK_SK	367

cl0030	ct0036	cn0037	pOP-CNH03648_EST_C_1_pSK_SK	397
cl0030	ct0036	cn0037	pOP-CNH03674_EST_C_1_pSK_SK	289
cl0030	ct0036	cn0037	pOP-CNH03684_EST_C_1_pSK_SK	365
cl0030	ct0036	cn0037	pOP-CNH03685_EST_C_1_pSK_SK	365
cl0030	ct0036	cn0037	pOP-CNH03687_EST_C_1_pSK_SK	473
cl0030	ct0036	cn0037	pOP-CNH03690_EST_C_1_pSK_SK	431
cl0030	ct0036	cn0037	pOP-CNH03692_EST_C_1_pSK_SK	365
cl0030	ct0036	cn0037	pOP-CNH03693_EST_C_1_pSK_SK	365
cl0030	ct0036	cn0037	pOP-CNH03715_EST_C_1_pSK_SK	221
cl0030	ct0036	cn0037	pOP-CNH03756_EST_C_1_pSK_SK	404
cl0030	ct0036	cn0037	pOP-CNH03772_EST_C_1_pSK_SK	423
cl0030	ct0036	cn0037	pOP-CNH03795_EST_C_1_pSK_SK	518
cl0030	ct0036	cn0037	pOP-CNH03797_EST_C_1_pSK_SK	559
cl0030	ct0036	cn0037	pOP-CNH03819_EST_C_1_pSK_SK	364
cl0030	ct0036	cn0037	pOP-CNH04138	436
cl0030	ct0036	cn0037	pOP-CNH04141	427
cl0030	ct0036	cn0037	pOP-CNH04182	437
cl0030	ct0036	cn0037	pOP-CNH04184	472
cl0030	ct0036	cn0037	pOP-CNH04194	382
cl0030	ct0036	cn0037	pOP-CNH04201	423
cl0030	ct0036	cn0037	pOP-CNH04205	410
cl0030	ct0036	cn0037	pOP-CNH04234	291
cl0030	ct0036	cn0037	pOP-CNH04266	437
cl0030	ct0036	cn0037	pOP-CNH04275	482
cl0030	ct0036	cn0037	pOP-CNH04291	356
cl0030	ct0036	cn0037	pOP-CNH04320	350
cl0030	ct0036	cn0037	pOP-CNH04333	844
cl0030	ct0036	cn0037	pOP-CNH04348	408
cl0030	ct0036	cn0037	pOP-CNH04350	539
cl0030	ct0036	cn0037	pOP-CNH04358	692
cl0030	ct0036	cn0037	pOP-CNH04363	370
cl0030	ct0036	cn0037	pOP-CNH04369	743
cl0030	ct0036	cn0037	pOP-CNH04374	540
cl0030	ct0036	cn0037	pOP-CNH04377	593
cl0030	ct0036	cn0037	pOP-CNH04380	526
cl0030	ct0036	cn0037	pOP-CNH04385	430
cl0030	ct0036	cn0037	pOP-CNH04423	577
cl0030	ct0036	cn0037	pOP-CNH04426	420
cl0030	ct0036	cn0037	pOP-CNH04437	429
cl0030	ct0036	cn0037	pOP-CNH04450	420
cl0030	ct0036	cn0037	pOP-CNH04483	650
cl0030	ct0036	cn0037	pOP-CNH04501	381
cl0030	ct0036	cn0037	pOP-CNH04523	420
cl0030	ct0036	cn0037	pOP-CNH04535	423
cl0030	ct0036	cn0037	pOP-CNH04578	447
cl0030	ct0036	cn0037	pOP-CNH04613	370
cl0030	ct0036	cn0037	pOP-CNH04661	587
cl0030	ct0036	cn0037	pOP-CNH04678	579
cl0030	ct0036	cn0037	pOP-CNH04686	350
cl0030	ct0036	cn0037	pOP-CNH04689	543
cl0030	ct0036	cn0037	pOP-CNH04696	792
cl0030	ct0036	cn0037	pOP-CNH04698	745
cl0030	ct0036	cn0037	pOP-CNH04701	386
cl0030	ct0036	cn0037	pOP-CNH04703	726
cl0030	ct0036	cn0037	pOP-CNH04713_EST_C_1_pSK_SK	590
cl0030	ct0036	cn0037	pOP-CNH04729_EST_C_1_pSK_SK	426

cl0030	ct0036	cn0037	pOP-CNH04752_EST_C_1_pSK_SK	338
cl0030	ct0036	cn0037	pOP-CNH04756_EST_C_1_pSK_SK	382
cl0030	ct0036	cn0037	pOP-CNH04757_EST_C_1_pSK_SK	423
cl0030	ct0036	cn0037	pOP-CNH04782_EST_C_1_pSK_SK	502
cl0030	ct0036	cn0037	pOP-CNH04800_EST_C_1_pSK_SK	422
cl0030	ct0036	cn0037	pOP-CNH04874_EST_C_1_pSK_SK	782
cl0030	ct0036	cn0037	pOP-CNH04885_EST_C_1_pSK_SK	808
cl0030	ct0036	cn0037	pOP-CNH04914_EST_C_1_pSK_SK	669
cl0030	ct0036	cn0037	pOP-CNH04919_EST_C_1_pSK_SK	704
cl0030	ct0036	cn0037	pOP-CNH04944_EST_C_1_pSK_SK	767
cl0030	ct0036	cn0037	pOP-CNH04959_EST_C_1_pSK_SK	607
cl0030	ct0036	cn0037	pOP-CNH04974_EST_C_1_pSK_SK	723
cl0030	ct0036	cn0037	pOP-CNH04988_EST_C_1_pSK_SK	706
cl0030	ct0036	cn0037	pOP-CNH05042_EST_C_1_pSK_SK	628
cl0030	ct0036	cn0037	pOP-CNHP00107_EST_C_1_pSK_SK	298
cl0030	ct0036	cn0037	pOP-CNHP00257_EST_C_1_pSK_SK	300
cl0030	ct0036	cn0037	pOP-CNHP00290_EST_C_1_pSK_SK	422
cl0030	ct0036	cn0037	pOP-CNI02002_EST_C_1_pSK_SK	533
cl0030	ct0036	cn0037	pOP-CNIP00812_EST_C_1_pSK_SK	690
cl0030	ct0036	cn0037	pOP-CNIP01017_EST_C_1_pSK_SK	611
cl0030	ct0036	cn0037	pOP-EAP00256_EST_C_1_pBSK_SK	265
cl0030	ct0036	cn0037	pOP-EAP00826_EST_C_1_pBSK_SK	528
cl0030	ct0036	cn0037	pOP-EAP00927_EST_C_1_pBSK_SK	407
cl0030	ct0036	cn0037	pOP-EAP01336_EST_C_1_pBSK_SK	615
cl0030	ct0036	cn0037	pOP-EAP01342_EST_C_1_pBSK_SK	438
cl0030	ct0036	cn0037	pOP-EAP01817_EST_C_1_pBSK_SK	577
cl0030	ct0036	cn0037	pOP-EAP03142_EST_C_1_pBSK_SK	138
cl0030	ct0036	cn0037	pOP-EAP03399_EST_C_1_pBSK_SK	551
cl0030	ct0036	cn0037	pOP-EAP03798_EST_C_1_pBSK_SK	617
cl0030	ct0036	cn0037	pOP-EN00266_EST_C_1_pSK_SK	576
cl0030	ct0036	cn0037	pOP-EN00271_EST_C_1_pSK_SK	423
cl0030	ct0036	cn0037	pOP-EN00388_EST_C_1_pSK_SK	399
cl0030	ct0036	cn0037	pOP-EO02704_EST_C_1_pSK_SK	448
cl0030	ct0036	cn0037	pOP-EO03254_EST_C_1_pSK_SK	386
cl0030	ct0036	cn0037	pOP-EO03733_EST_C_1_pSK_SK	513
cl0030	ct0036	cn0037	pOP-EO04287_EST_C_1_pSK_SK	282
cl0030	ct0036	cn0037	pOP-EO05352_EST_C_1_pSK_SK	423
cl0030	ct0036	cn0037	pOP-EO05500_EST_C_1_pSK_SK	390
cl0030	ct0036	cn0037	pOP-EO06294_EST_C_1_pSK_SK	649
cl0030	ct0036	cn0037	pOP-EO07134_EST_C_1_pSK_SK	399
cl0030	ct0036	cn0037	pOP-EO07168_EST_C_1_pSK_SK	284
cl0030	ct0036	cn0037	pOP-EO07192_EST_C_1_pSK_SK	423
cl0030	ct0036	cn0037	pOP-EO07216_EST_C_1_pSK_SK	713
cl0030	ct0036	cn0037	pOP-EO07296_EST_C_1_pSK_SK	812
cl0030	ct0036	cn0037	pOP-EO07405_EST_C_1_pSK_SK	572
cl0030	ct0036	cn0037	pOP-EO07429_EST_C_1_pSK_SK	780
cl0030	ct0036	cn0037	pOP-EO07545_EST_C_1_pSK_SK	406
cl0030	ct0036	cn0037	pOP-EO07723_EST_C_1_pSK_SK	700
cl0030	ct0036	cn0037	pOP-EO07775_EST_C_1_pSK_SK	423
cl0030	ct0036	cn0037	pOP-EO07793_EST_C_1_pSK_SK	470
cl0030	ct0036	cn0037	pOP-EO08106_EST_C_1_pSK_SK	537
cl0030	ct0036	cn0037	pOP-EO08130_EST_C_1_pSK_SK	423
cl0030	ct0036	cn0037	pOP-EO08330_EST_C_1_pSK_SK	517
cl0030	ct0036	cn0037	pOP-EO08347_EST_C_1_pSK_SK	215
cl0031	ct0037	cn0038	pOP-CNH00756_EST_C_1_pSK_SK	527
cl0031	ct0037	cn0038	pOP-CNH01266_EST_C_1_pSK_SK	403

cl0032	ct0038	cn0039	pOP-CNH00996_EST_C_1_pSK_SK	344
cl0032	ct0038	cn0039	pOP-CNH01054_EST_C_1_pSK_SK	474
cl0032	ct0038	cn0039	pOP-CNH01273_EST_C_1_pSK_SK	596
cl0032	ct0038	cn0039	pOP-CNH04229	652
cl0033	ct0039	cn0040	pOP-CNH00961_EST_C_1_pSK_SK	593
cl0033	ct0039	cn0040	pOP-CNH01278_EST_C_1_pSK_SK	463
cl0034	ct0040	cn0041	pOP-CNH00666_EST_C_1_pSK_SK	564
cl0034	ct0040	cn0041	pOP-CNH01283_EST_C_1_pSK_SK	651
cl0035	ct0041	cn0042	pOP-EO05249_EST_C_1_pSK_SK	469
cl0035	ct0042	cn0043	pOP-CNH01299_EST_C_1_pSK_SK	690
cl0036	ct0043	cn0044	pOP-CNH01230_EST_C_1_pSK_SK	472
cl0036	ct0043	cn0044	pOP-CNH01320_EST_C_1_pSK_SK	647
cl0037	ct0044	cn0045	pOP-CNH01323_EST_C_1_pSK_SK	759
cl0037	ct0044	cn0045	pOP-CNH04522	814
cl0038	ct0045	cn0046	pOP-CNH01326_EST_C_1_pSK_SK	653
cl0038	ct0045	cn0046	pOP-CNH04637	694
cl0038	ct0045	cn0046	pOP-EO03451_EST_C_1_pSK_SK	491
cl0039	ct0046	cn0047	pOP-CNH01348_EST_C_1_pSK_SK	415
cl0039	ct0046	cn0047	pOP-CNH04491	632
cl0040	ct0047	cn0048	pOP-CNH01355_EST_C_1_pSK_SK	654
cl0040	ct0047	cn0048	pOP-CNH04235	608
cl0041	ct0048	cn0049	pOP-CNH00935_EST_C_1_pSK_SK	632
cl0041	ct0048	cn0049	pOP-CNH01378_EST_C_1_pSK_SK	743
cl0042	ct0049	cn0050	pOP-CNH01086_EST_C_1_pSK_SK	329
cl0042	ct0049	cn0050	pOP-CNH01347_EST_C_1_pSK_SK	561
cl0042	ct0049	cn0050	pOP-CNH01402_EST_C_1_pSK_SK	466
cl0043	ct0050	cn0051	pOP-CEO00791_EST_C_1_pSK_SK	370
cl0043	ct0050	cn0051	pOP-CNH01407_EST_C_1_pSK_SK	406
cl0043	ct0050	cn0051	pOP-CNI01520_EST_C_1_pSK_SK	622
cl0043	ct0050	cn0051	pOP-EO04821_EST_C_1_pSK_SK	574
cl0044	ct0051	cn0052	pOP-CNH00819_EST_C_1_pSK_SK	469
cl0044	ct0051	cn0052	pOP-CNH01411_EST_C_1_pSK_SK	432
cl0045	ct0052	cn0053	pOP-CNH01026_EST_C_1_pSK_SK	423
cl0045	ct0052	cn0053	pOP-CNH01429_EST_C_1_pSK_SK	562
cl0046	ct0053	cn0054	pOP-CNH00952_EST_C_1_pSK_SK	616
cl0046	ct0053	cn0054	pOP-CNH01438_EST_C_1_pSK_SK	589
cl0047	ct0054	cn0055	pOP-CEO02044_EST_C_1_pSK_SK	285
cl0047	ct0054	cn0055	pOP-CNH01448_EST_C_1_pSK_SK	594
cl0048	ct0055	cn0056	pOP-CNH01466_EST_C_1_pSK_SK	581
cl0048	ct0055	cn0056	pOP-CNH04231	554
cl0049	ct0056	cn0057	pOP-CNH01265_EST_C_1_pSK_SK	435
cl0049	ct0056	cn0057	pOP-CNH01470_EST_C_1_pSK_SK	477
cl0050	ct0057	cn0058	pOP-CNH01027_EST_C_1_pSK_SK	556
cl0050	ct0057	cn0058	pOP-CNH01471_EST_C_1_pSK_SK	478
cl0051	ct0058	cn0059	pOP-CNH00555_EST_C_1_pSK_SK	204
cl0051	ct0058	cn0059	pOP-CNH01488_EST_C_1_pSK_SK	600
cl0052	ct0059	cn0060	pOP-CNH00808_EST_C_1_pSK_SK	600
cl0052	ct0059	cn0060	pOP-CNH01538_EST_C_1_pSK_SK	486
cl0052	ct0059	cn0060	pOP-CNH04411	878
cl0053	ct0060	cn0061	pOP-CNH01542_EST_C_1_pSK_SK	348
cl0053	ct0060	cn0061	pOP-CNH04399	347
cl0054	ct0061	cn0062	pOP-CNH01553_EST_C_1_pSK_SK	640
cl0054	ct0061	cn0062	pOP-CNH04505	749
cl0055	ct0062	cn0063	pOP-CNH01292_EST_C_1_pSK_SK	440
cl0055	ct0062	cn0063	pOP-CNH01563_EST_C_1_pSK_SK	523
cl0056	ct0063	cn0064	pOP-CNH01564_EST_C_1_pSK_SK	630

cl0056	ct0063	cn0064	pOP-CNH04368	641
cl0057	ct0064	cn0065	pOP-CNH00949_EST_C_1_pSK_SK	592
cl0057	ct0064	cn0065	pOP-CNH01565_EST_C_1_pSK_SK	605
cl0058	ct0065	cn0066	pOP-CNH01198_EST_C_1_pSK_SK	701
cl0058	ct0065	cn0066	pOP-CNH01590_EST_C_1_pSK_SK	455
cl0059	ct0066	cn0067	pOP-CNH01588_EST_C_1_pSK_SK	661
cl0059	ct0066	cn0067	pOP-CNH01591_EST_C_1_pSK_SK	485
cl0060	ct0067	cn0068	pOP-CNH01151_EST_C_1_pSK_SK	217
cl0060	ct0067	cn0068	pOP-CNH01612_EST_C_1_pSK_SK	433
cl0061	ct0068	cn0069	pOP-CEO01380_EST_C_1_pSK_SK	465
cl0061	ct0068	cn0069	pOP-CNIP00056_EST_C_1_pSK_SK	449
cl0061	ct0068	cn0069	pOP-EAP03410_EST_C_1_pBSK_SK	301
cl0061	ct0068	cn0069	pOP-EO06594_EST_C_1_pSK_SK	849
cl0061	ct0068	cn0070	pOP-CNH01614_EST_C_1_pSK_SK	606
cl0062	ct0069	cn0071	pOP-CNH01108_EST_C_1_pSK_SK	250
cl0062	ct0069	cn0071	pOP-CNH01647_EST_C_1_pSK_SK	628
cl0063	ct0070	cn0072	pOP-CNH01194_EST_C_1_pSK_SK	524
cl0063	ct0070	cn0072	pOP-CNH01652_EST_C_1_pSK_SK	732
cl0064	ct0071	cn0073	pOP-CNH01371_EST_C_1_pSK_SK	772
cl0064	ct0071	cn0073	pOP-CNH01682_EST_C_1_pSK_SK	591
cl0065	ct0072	cn0074	pOP-CNH01049_EST_C_1_pSK_SK	558
cl0065	ct0072	cn0074	pOP-CNH01705_EST_C_1_pSK_SK	559
cl0065	ct0072	cn0074	pOP-CNH04586	549
cl0066	ct0073	cn0075	pOP-CNH01714_EST_C_1_pSK_SK	508
cl0066	ct0073	cn0075	pOP-CNH04185	581
cl0067	ct0074	cn0076	pOP-CNH00926_EST_C_1_pSK_SK	427
cl0067	ct0074	cn0076	pOP-CNH00976_EST_C_1_pSK_SK	645
cl0067	ct0074	cn0076	pOP-CNH01412_EST_C_1_pSK_SK	414
cl0067	ct0074	cn0076	pOP-CNH01629_EST_C_1_pSK_SK	427
cl0067	ct0074	cn0076	pOP-CNH01716_EST_C_1_pSK_SK	425
cl0067	ct0074	cn0076	pOP-CNH04287	409
cl0067	ct0074	cn0076	pOP-CNH04345	408
cl0068	ct0075	cn0077	pOP-CNH01731_EST_C_1_pSK_SK	642
cl0068	ct0075	cn0078	pOP-CNH01653_EST_C_1_pSK_SK	415
cl0069	ct0076	cn0079	pOP-CNH01375_EST_C_1_pSK_SK	645
cl0069	ct0076	cn0079	pOP-CNH01733_EST_C_1_pSK_SK	569
cl0070	ct0077	cn0080	pOP-CNH01385_EST_C_1_pSK_SK	771
cl0070	ct0077	cn0080	pOP-CNH01742_EST_C_1_pSK_SK	658
cl0071	ct0078	cn0081	pOP-CNH01756_EST_C_1_pSK_SK	501
cl0071	ct0078	cn0081	pOP-CNH04289	368
cl0072	ct0079	cn0082	pOP-CNH01763_EST_C_1_pSK_SK	599
cl0072	ct0079	cn0082	pOP-CNH04706	898
cl0073	ct0080	cn0083	pOP-CNH00602_EST_C_1_pSK_SK	651
cl0073	ct0080	cn0083	pOP-CNH01262_EST_C_1_pSK_SK	478
cl0073	ct0080	cn0083	pOP-CNH01767_EST_C_1_pSK_SK	576
cl0074	ct0081	cn0084	pOP-CNH01475_EST_C_1_pSK_SK	654
cl0074	ct0081	cn0084	pOP-CNH01801_EST_C_1_pSK_SK	587
cl0075	ct0082	cn0085	pOP-CNH01067_EST_C_1_pSK_SK	571
cl0075	ct0082	cn0085	pOP-CNH01817_EST_C_1_pSK_SK	480
cl0075	ct0082	cn0085	pOP-CNH04391	839
cl0075	ct0083	cn0086	pOP-CAP00009_EST_C_1_pBSK_SK	516
cl0075	ct0083	cn0086	pOP-CAP00408_EST_C_1_pBSK_SK	521
cl0075	ct0083	cn0086	pOP-CBP00005_EST_C_1_pBSK_SK	529
cl0075	ct0083	cn0086	pOP-CBP00045_EST_C_1_pBSK_SK	202
cl0075	ct0083	cn0086	pOP-CBP00180_EST_C_1_pBSK_SK	426
cl0075	ct0083	cn0086	pOP-CBP00230_EST_C_1_pBSK_SK	449

cl0075	ct0083	cn0086	pOP-CBP00252_EST_C_1_pBSK_SK	451
cl0075	ct0083	cn0086	pOP-CBP00265_EST_C_1_pBSK_SK	426
cl0075	ct0083	cn0086	pOP-CEM00139_EST_C_1_pSK_SK	216
cl0075	ct0083	cn0086	pOP-CEO01321	180
cl0075	ct0083	cn0086	pOP-CEO01498_EST_C_1_pSK_SK	129
cl0075	ct0083	cn0086	pOP-CEO03226_EST_C_1_pSK_SK	252
cl0075	ct0083	cn0086	pOP-CNH02432_EST_C_1_pSK_SK	262
cl0075	ct0083	cn0086	pOP-CNHP00158_EST_C_1_pSK_SK	303
cl0075	ct0083	cn0086	pOP-CNI01713_EST_C_1_pSK_SK	268
cl0075	ct0083	cn0086	pOP-CNI01874_EST_C_1_pSK_SK	170
cl0075	ct0083	cn0086	pOP-CNIP00198_EST_C_1_pSK_SK	277
cl0075	ct0083	cn0086	pOP-CNIP00215_EST_C_1_pSK_SK	212
cl0075	ct0083	cn0086	pOP-CNIP00482_EST_C_1_pSK_SK	442
cl0075	ct0083	cn0086	pOP-CNIP00520_EST_C_1_pSK_SK	353
cl0075	ct0083	cn0086	pOP-CNIP00817_EST_C_1_pSK_SK	218
cl0075	ct0083	cn0086	pOP-EAP00765_EST_C_1_pBSK_SK	417
cl0075	ct0083	cn0086	pOP-EAP00783_EST_C_1_pBSK_SK	434
cl0075	ct0083	cn0086	pOP-EAP02722_EST_C_1_pBSK_SK	409
cl0075	ct0083	cn0086	pOP-EO02425_EST_C_1_pSK_SK	333
cl0075	ct0083	cn0086	pOP-EO02604_EST_C_1_pSK_SK	373
cl0075	ct0083	cn0086	pOP-EO02675_EST_C_1_pSK_SK	305
cl0075	ct0083	cn0086	pOP-EO02752_EST_C_1_pSK_SK	299
cl0075	ct0083	cn0086	pOP-EO02916_EST_C_1_pSK_SK	382
cl0075	ct0083	cn0086	pOP-EO03410_EST_C_1_pSK_SK	299
cl0075	ct0083	cn0086	pOP-EO03501_EST_C_1_pSK_SK	417
cl0075	ct0083	cn0086	pOP-EO03564_EST_C_1_pSK_SK	319
cl0075	ct0083	cn0086	pOP-EO03603_EST_C_1_pSK_SK	308
cl0075	ct0083	cn0086	pOP-EO03616_EST_C_1_pSK_SK	478
cl0075	ct0083	cn0086	pOP-EO04451_EST_C_1_pSK_SK	506
cl0075	ct0083	cn0086	pOP-EO04921_EST_C_1_pSK_SK	423
cl0075	ct0083	cn0086	pOP-EO05462_EST_C_1_pSK_SK	426
cl0075	ct0083	cn0086	pOP-EO05472_EST_C_1_pSK_SK	507
cl0075	ct0083	cn0086	pOP-EO05986_EST_C_1_pSK_SK	362
cl0075	ct0083	cn0086	pOP-EO08319_EST_C_1_pSK_SK	449
cl0075	ct0083	cn0087	pOP-CEO01079_EST_C_1_pSK_SK	612
cl0076	ct0084	cn0088	pOP-CNH01308_EST_C_1_pSK_SK	400
cl0076	ct0084	cn0088	pOP-CNH01719_EST_C_1_pSK_SK	552
cl0076	ct0084	cn0088	pOP-CNH01820_EST_C_1_pSK_SK	445
cl0076	ct0084	cn0088	pOP-CNH04528	804
cl0077	ct0085	cn0089	pOP-CNH01517_EST_C_1_pSK_SK	502
cl0077	ct0085	cn0089	pOP-CNH01834_EST_C_1_pSK_SK	545
cl0078	ct0086	cn0090	pOP-CNH00709_EST_C_1_pSK_SK	567
cl0078	ct0086	cn0090	pOP-CNH01837_EST_C_1_pSK_SK	490
cl0079	ct0087	cn0091	pOP-CNH01855_EST_C_1_pSK_SK	451
cl0079	ct0087	cn0091	pOP-CNH04581	825
cl0080	ct0088	cn0092	pOP-CNH01868_EST_C_1_pSK_SK	420
cl0080	ct0088	cn0092	pOP-CNH04152	632
cl0080	ct0088	cn0092	pOP-CNH04159	583
cl0081	ct0089	cn0093	pOP-CNH01870_EST_C_1_pSK_SK	397
cl0081	ct0089	cn0093	pOP-CNH04298	556
cl0082	ct0090	cn0094	pOP-CNH01875_EST_C_1_pSK_SK	388
cl0082	ct0090	cn0094	pOP-CNH04667	778
cl0083	ct0091	cn0095	pOP-CNH01545_EST_C_1_pSK_SK	411
cl0083	ct0091	cn0095	pOP-CNH01876_EST_C_1_pSK_SK	348
cl0083	ct0091	cn0095	pOP-EO03592_EST_C_1_pSK_SK	424
cl0084	ct0092	cn0096	pOP-CNH01818_EST_C_1_pSK_SK	548

cl0084	ct0092	cn0096	pOP-CNH01883_EST_C_1_pSK_SK	615
cl0085	ct0093	cn0097	pOP-CNH01890_EST_C_1_pSK_SK	485
cl0085	ct0093	cn0097	pOP-CNH04683	591
cl0086	ct0094	cn0098	pOP-CNH00620_EST_C_1_pSK_SK	524
cl0086	ct0094	cn0098	pOP-CNH01896_EST_C_1_pSK_SK	535
cl0087	ct0095	cn0099	pOP-CNH01917_EST_C_1_pSK_SK	670
cl0087	ct0095	cn0099	pOP-CNH04232	456
cl0088	ct0096	cn0100	pOP-CNH01145_EST_C_1_pSK_SK	500
cl0088	ct0096	cn0100	pOP-CNH01922_EST_C_1_pSK_SK	633
cl0089	ct0097	cn0101	pOP-CNH00992_EST_C_1_pSK_SK	641
cl0089	ct0097	cn0101	pOP-CNH01928_EST_C_1_pSK_SK	774
cl0090	ct0098	cn0102	pOP-CNH01048_EST_C_1_pSK_SK	455
cl0090	ct0098	cn0102	pOP-CNH01936_EST_C_1_pSK_SK	482
cl0091	ct0099	cn0103	pOP-CNH01474_EST_C_1_pSK_SK	578
cl0091	ct0099	cn0103	pOP-CNH01938_EST_C_1_pSK_SK	724
cl0092	ct0100	cn0104	pOP-CNH01695_EST_C_1_pSK_SK	341
cl0092	ct0100	cn0104	pOP-CNH01940_EST_C_1_pSK_SK	537
cl0093	ct0101	cn0105	pOP-CNH01975_EST_C_1_pSK_SK	722
cl0093	ct0102	cn0106	pOP-CNH00672_EST_C_1_pSK_SK	597
cl0093	ct0102	cn0106	pOP-CNH00873_EST_C_1_pSK_SK	627
cl0093	ct0102	cn0106	pOP-CNH04255	604
cl0094	ct0103	cn0107	pOP-CNH01144_EST_C_1_pSK_SK	523
cl0094	ct0103	cn0107	pOP-CNH01987_EST_C_1_pSK_SK	485
cl0095	ct0104	cn0108	pOP-CNH01988_EST_C_1_pSK_SK	471
cl0095	ct0104	cn0108	pOP-CNH04250	489
cl0096	ct0105	cn0109	pOP-CNH02007_EST_C_1_pSK_SK	536
cl0096	ct0105	cn0109	pOP-EO05396_EST_C_1_pSK_SK	539
cl0097	ct0106	cn0110	pOP-CNH01091_EST_C_1_pSK_SK	597
cl0097	ct0106	cn0110	pOP-CNH02034_EST_C_1_pSK_SK	470
cl0098	ct0107	cn0111	pOP-CNH01189_EST_C_1_pSK_SK	612
cl0098	ct0107	cn0111	pOP-CNH02048_EST_C_1_pSK_SK	522
cl0099	ct0108	cn0112	pOP-CNH02053_EST_C_1_pSK_SK	577
cl0099	ct0108	cn0112	pOP-CNH04699	582
cl0100	ct0109	cn0113	pOP-CNH01672_EST_C_1_pSK_SK	657
cl0100	ct0109	cn0113	pOP-CNH01799_EST_C_1_pSK_SK	471
cl0100	ct0109	cn0113	pOP-CNH02072_EST_C_1_pSK_SK	608
cl0100	ct0109	cn0113	pOP-CNH04634	688
cl0101	ct0110	cn0114	pOP-CNH01064_EST_C_1_pSK_SK	606
cl0101	ct0110	cn0114	pOP-CNH02078_EST_C_1_pSK_SK	567
cl0102	ct0111	cn0115	pOP-CNH01706_EST_C_1_pSK_SK	630
cl0102	ct0111	cn0115	pOP-CNH02084_EST_C_1_pSK_SK	606
cl0103	ct0112	cn0116	pOP-CNH01654_EST_C_1_pSK_SK	667
cl0103	ct0112	cn0116	pOP-CNH02089_EST_C_1_pSK_SK	550
cl0104	ct0113	cn0117	pOP-CNH02118_EST_C_1_pSK_SK	432
cl0104	ct0113	cn0117	pOP-CNH04550	862
cl0104	ct0113	cn0117	pOP-CNH04606	805
cl0105	ct0114	cn0118	pOP-CNH01678_EST_C_1_pSK_SK	675
cl0105	ct0114	cn0118	pOP-CNH02122_EST_C_1_pSK_SK	525
cl0106	ct0115	cn0119	pOP-CNH00849_EST_C_1_pSK_SK	552
cl0106	ct0115	cn0119	pOP-CNH02129_EST_C_1_pSK_SK	530
cl0107	ct0116	cn0120	pOP-CNH02133_EST_C_1_pSK_SK	576
cl0107	ct0116	cn0120	pOP-CNH02135_EST_C_1_pSK_SK	639
cl0108	ct0117	cn0121	pOP-CNH01735_EST_C_1_pSK_SK	507
cl0108	ct0117	cn0121	pOP-CNH02139_EST_C_1_pSK_SK	517
cl0109	ct0118	cn0122	pOP-CNH01087_EST_C_1_pSK_SK	457
cl0109	ct0118	cn0122	pOP-CNH02155_EST_C_1_pSK_SK	568

cl0110	ct0119	cn0123	pOP-CNH00951_EST_C_1_pSK_SK	586
cl0110	ct0119	cn0123	pOP-CNH01037_EST_C_1_pSK_SK	205
cl0110	ct0119	cn0123	pOP-CNH02163_EST_C_1_pSK_SK	483
cl0110	ct0119	cn0123	pOP-CNH03290_EST_C_1_pSK_SK	591
cl0110	ct0119	cn0123	pOP-CNH04601	726
cl0111	ct0120	cn0124	pOP-CNH01600_EST_C_1_pSK_SK	445
cl0111	ct0120	cn0124	pOP-CNH02187_EST_C_1_pSK_SK	586
cl0112	ct0121	cn0125	pOP-CNH02190_EST_C_1_pSK_SK	610
cl0112	ct0121	cn0125	pOP-CNH04415	805
cl0113	ct0122	cn0126	pOP-CNH01316_EST_C_1_pSK_SK	819
cl0113	ct0122	cn0126	pOP-CNH02192_EST_C_1_pSK_SK	356
cl0113	ct0122	cn0126	pOP-CNH04302	555
cl0114	ct0123	cn0127	pOP-CNH02201_EST_C_1_pSK_SK	581
cl0114	ct0123	cn0127	pOP-CNH04273	585
cl0115	ct0124	cn0128	pOP-CNH01850_EST_C_1_pSK_SK	562
cl0115	ct0124	cn0128	pOP-CNH02137_EST_C_1_pSK_SK	571
cl0115	ct0124	cn0128	pOP-CNH02205_EST_C_1_pSK_SK	631
cl0116	ct0125	cn0129	pOP-CNH01329_EST_C_1_pSK_SK	235
cl0116	ct0125	cn0129	pOP-CNH02216_EST_C_1_pSK_SK	235
cl0116	ct0125	cn0129	pOP-CNH02220_EST_C_1_pSK_SK	235
cl0117	ct0126	cn0130	pOP-CNH01391_EST_C_1_pSK_SK	699
cl0117	ct0126	cn0130	pOP-CNH02222_EST_C_1_pSK_SK	815
cl0118	ct0127	cn0131	pOP-CNH01377_EST_C_1_pSK_SK	598
cl0118	ct0127	cn0131	pOP-CNH02224_EST_C_1_pSK_SK	729
cl0119	ct0128	cn0132	pOP-CNH01786_EST_C_1_pSK_SK	609
cl0119	ct0128	cn0132	pOP-CNH02241_EST_C_1_pSK_SK	668
cl0120	ct0129	cn0133	pOP-CNH02251_EST_C_1_pSK_SK	591
cl0120	ct0129	cn0133	pOP-CNH04587	602
cl0121	ct0130	cn0134	pOP-CNH02260_EST_C_1_pSK_SK	678
cl0121	ct0130	cn0134	pOP-CNH04310	494
cl0122	ct0131	cn0135	pOP-CNH00938_EST_C_1_pSK_SK	530
cl0122	ct0131	cn0135	pOP-CNH02270_EST_C_1_pSK_SK	714
cl0122	ct0131	cn0135	pOP-CNH04223	567
cl0123	ct0132	cn0136	pOP-CNH02045_EST_C_1_pSK_SK	586
cl0123	ct0132	cn0136	pOP-CNH02288_EST_C_1_pSK_SK	660
cl0124	ct0133	cn0137	pOP-CNH02146_EST_C_1_pSK_SK	571
cl0124	ct0133	cn0137	pOP-CNH02295_EST_C_1_pSK_SK	590
cl0125	ct0134	cn0138	pOP-CNH01611_EST_C_1_pSK_SK	460
cl0125	ct0134	cn0138	pOP-CNH02302_EST_C_1_pSK_SK	668
cl0126	ct0135	cn0139	pOP-CNH02071_EST_C_1_pSK_SK	299
cl0126	ct0135	cn0139	pOP-CNH02316_EST_C_1_pSK_SK	381
cl0127	ct0136	cn0140	pOP-CNH01836_EST_C_1_pSK_SK	581
cl0127	ct0136	cn0140	pOP-CNH02321_EST_C_1_pSK_SK	646
cl0127	ct0136	cn0140	pOP-CNH04278	479
cl0127	ct0136	cn0140	pOP-CNH04279	567
cl0128	ct0137	cn0141	pOP-CNH02254_EST_C_1_pSK_SK	634
cl0128	ct0137	cn0141	pOP-CNH02326_EST_C_1_pSK_SK	555
cl0129	ct0138	cn0142	pOP-CNH02328_EST_C_1_pSK_SK	556
cl0129	ct0138	cn0142	pOP-CNH04681	820
cl0129	ct0138	cn0142	pOP-CNH04690	754
cl0130	ct0139	cn0143	pOP-CNH00611_EST_C_1_pSK_SK	720
cl0130	ct0139	cn0143	pOP-CNH00945_EST_C_1_pSK_SK	524
cl0130	ct0139	cn0143	pOP-CNH02330_EST_C_1_pSK_SK	597
cl0131	ct0140	cn0144	pOP-CNH01997_EST_C_1_pSK_SK	548
cl0131	ct0140	cn0144	pOP-CNH02340_EST_C_1_pSK_SK	621
cl0132	ct0141	cn0145	pOP-CNH00575_EST_C_1_pSK_SK	653

cl0132	ct0141	cn0145	pOP-CNH00718_EST_C_1_pSK_SK	612
cl0132	ct0141	cn0145	pOP-CNH01783_EST_C_1_pSK_SK	456
cl0132	ct0141	cn0145	pOP-CNH02348_EST_C_1_pSK_SK	530
cl0132	ct0141	cn0145	pOP-EO06026_EST_C_1_pSK_SK	580
cl0133	ct0142	cn0146	pOP-CNH01008_EST_C_1_pSK_SK	322
cl0133	ct0142	cn0146	pOP-CNH02370_EST_C_1_pSK_SK	425
cl0134	ct0143	cn0147	pOP-CNH01291_EST_C_1_pSK_SK	484
cl0134	ct0143	cn0147	pOP-CNH02408_EST_C_1_pSK_SK	417
cl0135	ct0144	cn0148	pOP-CNH02366_EST_C_1_pSK_SK	489
cl0135	ct0144	cn0148	pOP-CNH02416_EST_C_1_pSK_SK	540
cl0136	ct0145	cn0149	pOP-CNH02427_EST_C_1_pSK_SK	535
cl0136	ct0145	cn0149	pOP-CNH04559	692
cl0137	ct0146	cn0150	pOP-CNH01595_EST_C_1_pSK_SK	604
cl0137	ct0146	cn0150	pOP-CNH02446_EST_C_1_pSK_SK	479
cl0138	ct0147	cn0151	pOP-CNH02466_EST_C_1_pSK_SK	368
cl0138	ct0147	cn0151	pOP-CNH02483_EST_C_1_pSK_SK	419
cl0139	ct0148	cn0152	pOP-CNH01043_EST_C_1_pSK_SK	474
cl0139	ct0148	cn0152	pOP-CNH02487_EST_C_1_pSK_SK	384
cl0140	ct0149	cn0153	pOP-CNH02422_EST_C_1_pSK_SK	543
cl0140	ct0149	cn0153	pOP-CNH02496_EST_C_1_pSK_SK	516
cl0141	ct0150	cn0154	pOP-CNH02097_EST_C_1_pSK_SK	456
cl0141	ct0150	cn0154	pOP-CNH02503_EST_C_1_pSK_SK	456
cl0142	ct0151	cn0155	pOP-CNH02380_EST_C_1_pSK_SK	452
cl0142	ct0151	cn0155	pOP-CNH02507_EST_C_1_pSK_SK	347
cl0143	ct0152	cn0156	pOP-CNH02318_EST_C_1_pSK_SK	640
cl0143	ct0152	cn0156	pOP-CNH02511_EST_C_1_pSK_SK	537
cl0144	ct0153	cn0157	pOP-CNH00693_EST_C_1_pSK_SK	657
cl0144	ct0153	cn0157	pOP-CNH02441_EST_C_1_pSK_SK	519
cl0144	ct0153	cn0157	pOP-CNH02512_EST_C_1_pSK_SK	516
cl0144	ct0153	cn0157	pOP-CNH02513_EST_C_1_pSK_SK	507
cl0145	ct0154	cn0158	pOP-CNH01572_EST_C_1_pSK_SK	705
cl0145	ct0154	cn0158	pOP-CNH02517_EST_C_1_pSK_SK	481
cl0146	ct0155	cn0159	pOP-CNH00729_EST_C_1_pSK_SK	488
cl0146	ct0155	cn0159	pOP-CNH02526_EST_C_1_pSK_SK	501
cl0147	ct0156	cn0160	pOP-CNH01261_EST_C_1_pSK_SK	436
cl0147	ct0156	cn0160	pOP-CNH02543_EST_C_1_pSK_SK	483
cl0148	ct0157	cn0161	pOP-CNH00925_EST_C_1_pSK_SK	538
cl0148	ct0157	cn0161	pOP-CNH01649_EST_C_1_pSK_SK	538
cl0148	ct0157	cn0161	pOP-CNH01722_EST_C_1_pSK_SK	538
cl0148	ct0157	cn0161	pOP-CNH02547_EST_C_1_pSK_SK	538
cl0148	ct0157	cn0161	pOP-CNH04276	538
cl0149	ct0158	cn0162	pOP-CNH01436_EST_C_1_pSK_SK	625
cl0149	ct0158	cn0162	pOP-CNH02553_EST_C_1_pSK_SK	544
cl0150	ct0159	cn0163	pOP-CNH01449_EST_C_1_pSK_SK	544
cl0150	ct0159	cn0163	pOP-CNH02557_EST_C_1_pSK_SK	549
cl0151	ct0160	cn0164	pOP-CNH01606_EST_C_1_pSK_SK	546
cl0151	ct0160	cn0164	pOP-CNH02574_EST_C_1_pSK_SK	542
cl0152	ct0161	cn0165	pOP-CNH00805_EST_C_1_pSK_SK	591
cl0152	ct0161	cn0165	pOP-CNH02588_EST_C_1_pSK_SK	594
cl0153	ct0162	cn0166	pOP-CNH01290_EST_C_1_pSK_SK	623
cl0153	ct0162	cn0166	pOP-CNH02594_EST_C_1_pSK_SK	524
cl0154	ct0163	cn0167	pOP-CNH01613_EST_C_1_pSK_SK	558
cl0154	ct0164	cn0168	pOP-CNH02595_EST_C_1_pSK_SK	550
cl0155	ct0165	cn0169	pOP-CNH02603_EST_C_1_pSK_SK	249
cl0155	ct0165	cn0169	pOP-CNH02611_EST_C_1_pSK_SK	561
cl0155	ct0165	cn0169	pOP-CNH04339	712

cl0156	ct0166	cn0170	pOP-CNH00929_EST_C_1_pSK_SK	496
cl0156	ct0166	cn0170	pOP-CNH02625_EST_C_1_pSK_SK	515
cl0157	ct0167	cn0171	pOP-CNH00852_EST_C_1_pSK_SK	615
cl0157	ct0167	cn0171	pOP-CNH01288_EST_C_1_pSK_SK	596
cl0157	ct0167	cn0171	pOP-CNH02634_EST_C_1_pSK_SK	630
cl0158	ct0168	cn0172	pOP-CNH01453_EST_C_1_pSK_SK	585
cl0158	ct0168	cn0172	pOP-CNH02683_EST_C_1_pSK_SK	574
cl0159	ct0169	cn0173	pOP-CNH00998_EST_C_1_pSK_SK	487
cl0159	ct0169	cn0173	pOP-CNH02686_EST_C_1_pSK_SK	647
cl0160	ct0170	cn0174	pOP-CNH02699_EST_C_1_pSK_SK	628
cl0160	ct0170	cn0174	pOP-CNH04413	876
cl0160	ct0170	cn0174	pOP-CNH04577	657
cl0161	ct0171	cn0175	pOP-CNH02703_EST_C_1_pSK_SK	626
cl0161	ct0171	cn0175	pOP-CNH04218	528
cl0162	ct0172	cn0176	pOP-CNH01748_EST_C_1_pSK_SK	522
cl0162	ct0172	cn0176	pOP-CNH02724_EST_C_1_pSK_SK	593
cl0163	ct0173	cn0177	pOP-CNH01925_EST_C_1_pSK_SK	678
cl0163	ct0173	cn0177	pOP-CNH02739_EST_C_1_pSK_SK	624
cl0163	ct0173	cn0177	pOP-CNH04480	672
cl0164	ct0174	cn0178	pOP-CNH02226_EST_C_1_pSK_SK	781
cl0164	ct0174	cn0178	pOP-CNH02752_EST_C_1_pSK_SK	619
cl0165	ct0175	cn0179	pOP-CNH00835_EST_C_1_pSK_SK	581
cl0165	ct0175	cn0179	pOP-CNH02757_EST_C_1_pSK_SK	631
cl0166	ct0176	cn0180	pOP-CNH01219_EST_C_1_pSK_SK	564
cl0166	ct0176	cn0180	pOP-CNH02297_EST_C_1_pSK_SK	606
cl0166	ct0176	cn0180	pOP-CNH02758_EST_C_1_pSK_SK	619
cl0167	ct0177	cn0181	pOP-CNH01747_EST_C_1_pSK_SK	486
cl0167	ct0177	cn0181	pOP-CNH02223_EST_C_1_pSK_SK	723
cl0167	ct0177	cn0181	pOP-CNH02759_EST_C_1_pSK_SK	602
cl0167	ct0177	cn0181	pOP-CNH04532	757
cl0168	ct0178	cn0182	pOP-CNH02765_EST_C_1_pSK_SK	703
cl0168	ct0178	cn0182	pOP-CNH04658	656
cl0169	ct0179	cn0183	pOP-CNH02052_EST_C_1_pSK_SK	547
cl0169	ct0179	cn0183	pOP-CNH02767_EST_C_1_pSK_SK	623
cl0170	ct0180	cn0184	pOP-CNH00837_EST_C_1_pSK_SK	554
cl0170	ct0180	cn0184	pOP-CNH02783_EST_C_1_pSK_SK	613
cl0171	ct0181	cn0185	pOP-CNH00752_EST_C_1_pSK_SK	497
cl0171	ct0181	cn0185	pOP-CNH02785_EST_C_1_pSK_SK	642
cl0172	ct0182	cn0186	pOP-CNH01518_EST_C_1_pSK_SK	523
cl0172	ct0182	cn0186	pOP-CNH01963_EST_C_1_pSK_SK	699
cl0172	ct0182	cn0186	pOP-CNH02787_EST_C_1_pSK_SK	599
cl0172	ct0182	cn0186	pOP-CNH04506	812
cl0173	ct0183	cn0187	pOP-CNH02113_EST_C_1_pSK_SK	527
cl0173	ct0183	cn0187	pOP-CNH02814_EST_C_1_pSK_SK	523
cl0174	ct0184	cn0188	pOP-CNH02671_EST_C_1_pSK_SK	541
cl0174	ct0184	cn0188	pOP-CNH02820_EST_C_1_pSK_SK	600
cl0175	ct0185	cn0189	pOP-CNH01550_EST_C_1_pSK_SK	294
cl0175	ct0185	cn0189	pOP-CNH01741_EST_C_1_pSK_SK	574
cl0175	ct0185	cn0189	pOP-CNH02842_EST_C_1_pSK_SK	682
cl0176	ct0186	cn0190	pOP-CNH00717_EST_C_1_pSK_SK	495
cl0176	ct0186	cn0190	pOP-CNH01918_EST_C_1_pSK_SK	763
cl0176	ct0186	cn0190	pOP-CNH02845_EST_C_1_pSK_SK	594
cl0177	ct0187	cn0191	pOP-CNH00788_EST_C_1_pSK_SK	526
cl0177	ct0187	cn0191	pOP-CNH02849_EST_C_1_pSK_SK	607
cl0178	ct0188	cn0192	pOP-CNH00973_EST_C_1_pSK_SK	615
cl0178	ct0188	cn0192	pOP-CNH01138_EST_C_1_pSK_SK	200

cl0178	ct0188	cn0192	pOP-CNH01796_EST_C_1_pSK_SK	521
cl0178	ct0188	cn0192	pOP-CNH01941_EST_C_1_pSK_SK	696
cl0178	ct0188	cn0192	pOP-CNH02881_EST_C_1_pSK_SK	602
cl0178	ct0188	cn0192	pOP-CNH04325	731
cl0179	ct0189	cn0193	pOP-CNH01578_EST_C_1_pSK_SK	698
cl0179	ct0189	cn0193	pOP-CNH02607_EST_C_1_pSK_SK	610
cl0179	ct0189	cn0193	pOP-CNH02899_EST_C_1_pSK_SK	548
cl0180	ct0190	cn0194	pOP-CNH02919_EST_C_1_pSK_SK	641
cl0180	ct0190	cn0194	pOP-CNH04615	828
cl0181	ct0191	cn0195	pOP-CNH02232_EST_C_1_pSK_SK	735
cl0181	ct0191	cn0195	pOP-CNH02920_EST_C_1_pSK_SK	665
cl0182	ct0192	cn0196	pOP-CNH02474_EST_C_1_pSK_SK	371
cl0182	ct0192	cn0196	pOP-CNH02937_EST_C_1_pSK_SK	601
cl0182	ct0192	cn0196	pOP-CNH04662	819
cl0183	ct0193	cn0197	pOP-CNH00971_EST_C_1_pSK_SK	727
cl0183	ct0193	cn0197	pOP-CNH02945_EST_C_1_pSK_SK	658
cl0184	ct0194	cn0198	pOP-CNH01661_EST_C_1_pSK_SK	659
cl0184	ct0194	cn0198	pOP-CNH02568_EST_C_1_pSK_SK	677
cl0184	ct0194	cn0198	pOP-CNH02966_EST_C_1_pSK_SK	612
cl0185	ct0195	cn0199	pOP-CNH02894_EST_C_1_pSK_SK	497
cl0185	ct0195	cn0199	pOP-CNH02971_EST_C_1_pSK_SK	530
cl0186	ct0196	cn0200	pOP-CNH02985_EST_C_1_pSK_SK	588
cl0186	ct0196	cn0200	pOP-CNH04195	545
cl0186	ct0196	cn0200	pOP-CNH04444	758
cl0187	ct0197	cn0201	pOP-CNH01016_EST_C_1_pSK_SK	227
cl0187	ct0197	cn0201	pOP-CNH02990_EST_C_1_pSK_SK	627
cl0187	ct0197	cn0201	pOP-CNH04314	576
cl0187	ct0197	cn0201	pOP-CNH04497	675
cl0188	ct0198	cn0202	pOP-CNH01249_EST_C_1_pSK_SK	566
cl0188	ct0198	cn0202	pOP-CNH02949_EST_C_1_pSK_SK	643
cl0188	ct0198	cn0202	pOP-CNH03000_EST_C_1_pSK_SK	579
cl0189	ct0199	cn0203	pOP-CNH02658_EST_C_1_pSK_SK	615
cl0189	ct0199	cn0203	pOP-CNH03011_EST_C_1_pSK_SK	541
cl0190	ct0200	cn0204	pOP-CNH02221_EST_C_1_pSK_SK	488
cl0190	ct0200	cn0204	pOP-CNH03022_EST_C_1_pSK_SK	557
cl0191	ct0201	cn0205	pOP-CNH01889_EST_C_1_pSK_SK	652
cl0191	ct0201	cn0205	pOP-CNH01983_EST_C_1_pSK_SK	457
cl0191	ct0201	cn0205	pOP-CNH02056_EST_C_1_pSK_SK	563
cl0191	ct0201	cn0205	pOP-CNH02576_EST_C_1_pSK_SK	539
cl0191	ct0201	cn0205	pOP-CNH03034_EST_C_1_pSK_SK	563
cl0192	ct0202	cn0206	pOP-CNH02399_EST_C_1_pSK_SK	316
cl0192	ct0202	cn0206	pOP-CNH03036_EST_C_1_pSK_SK	646
cl0193	ct0203	cn0207	pOP-CNH01857_EST_C_1_pSK_SK	414
cl0193	ct0203	cn0207	pOP-CNH02944_EST_C_1_pSK_SK	571
cl0193	ct0203	cn0207	pOP-CNH03040_EST_C_1_pSK_SK	608
cl0194	ct0204	cn0208	pOP-CNH01185_EST_C_1_pSK_SK	520
cl0194	ct0204	cn0208	pOP-CNH03052_EST_C_1_pSK_SK	564
cl0195	ct0205	cn0209	pOP-CNH01566_EST_C_1_pSK_SK	715
cl0195	ct0205	cn0209	pOP-CNH01743_EST_C_1_pSK_SK	550
cl0195	ct0205	cn0209	pOP-CNH02037_EST_C_1_pSK_SK	491
cl0195	ct0205	cn0209	pOP-CNH02955_EST_C_1_pSK_SK	630
cl0195	ct0205	cn0209	pOP-CNH03053_EST_C_1_pSK_SK	629
cl0195	ct0205	cn0209	pOP-CNH04134	257
cl0195	ct0205	cn0209	pOP-CNH04576	833
cl0195	ct0205	cn0209	pOP-CNH04671	732
cl0196	ct0206	cn0210	pOP-CNH01255_EST_C_1_pSK_SK	710

cl0196	ct0206	cn0210	pOP-CNH02447_EST_C_1_pSK_SK	464
cl0196	ct0206	cn0210	pOP-CNH02840_EST_C_1_pSK_SK	652
cl0196	ct0206	cn0210	pOP-CNH03062_EST_C_1_pSK_SK	636
cl0197	ct0207	cn0211	pOP-CNH01708_EST_C_1_pSK_SK	449
cl0197	ct0207	cn0211	pOP-CNH03082_EST_C_1_pSK_SK	655
cl0197	ct0207	cn0211	pOP-CNH04354	778
cl0198	ct0208	cn0212	pOP-CNH03087_EST_C_1_pSK_SK	420
cl0198	ct0208	cn0212	pOP-CNH03088_EST_C_1_pSK_SK	193
cl0198	ct0208	cn0212	pOP-EO08099_EST_C_1_pSK_SK	299
cl0199	ct0209	cn0213	pOP-CBP00174_EST_C_1_pBSK_SK	398
cl0199	ct0209	cn0213	pOP-CNH03095_EST_C_1_pSK_SK	470
cl0200	ct0210	cn0214	pOP-CNH01065_EST_C_1_pSK_SK	624
cl0200	ct0210	cn0214	pOP-CNH03099_EST_C_1_pSK_SK	414
cl0201	ct0211	cn0215	pOP-CNH02672_EST_C_1_pSK_SK	704
cl0201	ct0211	cn0215	pOP-CNH02734_EST_C_1_pSK_SK	705
cl0201	ct0211	cn0215	pOP-CNH02861_EST_C_1_pSK_SK	530
cl0201	ct0211	cn0215	pOP-CNH03110_EST_C_1_pSK_SK	663
cl0202	ct0212	cn0216	pOP-CNH02088_EST_C_1_pSK_SK	578
cl0202	ct0212	cn0216	pOP-CNH02915_EST_C_1_pSK_SK	617
cl0202	ct0212	cn0216	pOP-CNH03115_EST_C_1_pSK_SK	533
cl0203	ct0213	cn0217	pOP-CNH02776_EST_C_1_pSK_SK	607
cl0203	ct0213	cn0217	pOP-CNH03122_EST_C_1_pSK_SK	610
cl0204	ct0214	cn0218	pOP-CNH01765_EST_C_1_pSK_SK	458
cl0204	ct0214	cn0218	pOP-CNH03057_EST_C_1_pSK_SK	461
cl0204	ct0214	cn0218	pOP-CNH03123_EST_C_1_pSK_SK	442
cl0205	ct0215	cn0219	pOP-CNH03128_EST_C_1_pSK_SK	513
cl0205	ct0215	cn0219	pOP-CNH04251	563
cl0206	ct0216	cn0220	pOP-CNH03130_EST_C_1_pSK_SK	592
cl0206	ct0216	cn0220	pOP-CNH04554	721
cl0206	ct0216	cn0220	pOP-CNH04610	786
cl0207	ct0217	cn0221	pOP-CNH02620_EST_C_1_pSK_SK	336
cl0207	ct0217	cn0221	pOP-CNH03136_EST_C_1_pSK_SK	569
cl0207	ct0217	cn0221	pOP-CNH04427	803
cl0208	ct0218	cn0222	pOP-CNH02731_EST_C_1_pSK_SK	582
cl0208	ct0218	cn0222	pOP-CNH03138_EST_C_1_pSK_SK	618
cl0209	ct0219	cn0223	pOP-CNH02979_EST_C_1_pSK_SK	520
cl0209	ct0219	cn0223	pOP-CNH03147_EST_C_1_pSK_SK	485
cl0210	ct0220	cn0224	pOP-CNH02208_EST_C_1_pSK_SK	524
cl0210	ct0220	cn0224	pOP-CNH03148_EST_C_1_pSK_SK	578
cl0211	ct0221	cn0225	pOP-CNH02068_EST_C_1_pSK_SK	556
cl0211	ct0221	cn0225	pOP-CNH03170_EST_C_1_pSK_SK	581
cl0211	ct0221	cn0225	pOP-CNH04537	787
cl0212	ct0222	cn0226	pOP-CNH02602_EST_C_1_pSK_SK	700
cl0212	ct0222	cn0226	pOP-CNH03177_EST_C_1_pSK_SK	621
cl0213	ct0223	cn0227	pOP-CNH00777_EST_C_1_pSK_SK	419
cl0213	ct0223	cn0227	pOP-CNH02322_EST_C_1_pSK_SK	558
cl0213	ct0223	cn0227	pOP-CNH02495_EST_C_1_pSK_SK	527
cl0213	ct0223	cn0227	pOP-CNH03184_EST_C_1_pSK_SK	527
cl0214	ct0224	cn0228	pOP-CNH01514_EST_C_1_pSK_SK	347
cl0214	ct0224	cn0228	pOP-CNH01949_EST_C_1_pSK_SK	589
cl0214	ct0224	cn0228	pOP-CNH03002_EST_C_1_pSK_SK	488
cl0214	ct0224	cn0228	pOP-CNH03190_EST_C_1_pSK_SK	468
cl0214	ct0224	cn0228	pOP-CNH03191_EST_C_1_pSK_SK	304
cl0215	ct0225	cn0229	pOP-CNH02362_EST_C_1_pSK_SK	727
cl0215	ct0225	cn0229	pOP-CNH02385_EST_C_1_pSK_SK	539
cl0215	ct0225	cn0229	pOP-CNH03201_EST_C_1_pSK_SK	531

cl0216	ct0226	cn0230	pOP-CNH02633_EST_C_1_pSK_SK	756
cl0216	ct0226	cn0230	pOP-CNH03202_EST_C_1_pSK_SK	543
cl0216	ct0226	cn0230	pOP-CNH04441	796
cl0216	ct0226	cn0231	pOP-CNH04322	445
cl0217	ct0227	cn0232	pOP-CNH03029_EST_C_1_pSK_SK	655
cl0217	ct0227	cn0232	pOP-CNH03044_EST_C_1_pSK_SK	659
cl0217	ct0227	cn0232	pOP-CNH03213_EST_C_1_pSK_SK	716
cl0218	ct0228	cn0233	pOP-CNH02044_EST_C_1_pSK_SK	551
cl0218	ct0228	cn0233	pOP-CNH03215_EST_C_1_pSK_SK	607
cl0219	ct0229	cn0234	pOP-CNH01752_EST_C_1_pSK_SK	523
cl0219	ct0229	cn0234	pOP-CNH03216_EST_C_1_pSK_SK	520
cl0220	ct0230	cn0235	pOP-CNH01245_EST_C_1_pSK_SK	705
cl0220	ct0230	cn0235	pOP-CNH01829_EST_C_1_pSK_SK	646
cl0220	ct0230	cn0235	pOP-CNH03226_EST_C_1_pSK_SK	583
cl0221	ct0231	cn0236	pOP-CNH02935_EST_C_1_pSK_SK	655
cl0221	ct0231	cn0236	pOP-CNH03229_EST_C_1_pSK_SK	634
cl0222	ct0232	cn0237	pOP-CNH02705_EST_C_1_pSK_SK	599
cl0222	ct0232	cn0237	pOP-CNH03234_EST_C_1_pSK_SK	429
cl0223	ct0233	cn0238	pOP-CNH02857_EST_C_1_pSK_SK	389
cl0223	ct0233	cn0238	pOP-CNH03235_EST_C_1_pSK_SK	387
cl0224	ct0234	cn0239	pOP-CNH02105_EST_C_1_pSK_SK	527
cl0224	ct0234	cn0239	pOP-CNH03247_EST_C_1_pSK_SK	564
cl0225	ct0235	cn0240	pOP-CNH02468_EST_C_1_pSK_SK	329
cl0225	ct0235	cn0240	pOP-CNH03260_EST_C_1_pSK_SK	563
cl0226	ct0236	cn0241	pOP-CNH03269_EST_C_1_pSK_SK	495
cl0226	ct0236	cn0241	pOP-EAP01548_EST_C_1_pBSK_SK	494
cl0227	ct0237	cn0242	pOP-CNH02609_EST_C_1_pSK_SK	586
cl0227	ct0237	cn0242	pOP-CNH03274_EST_C_1_pSK_SK	603
cl0228	ct0238	cn0243	pOP-CNH03276_EST_C_1_pSK_SK	616
cl0228	ct0238	cn0243	pOP-CNHP00508_EST_C_1_pSK_SK	594
cl0228	ct0239	cn0244	pOP-CEO03188_EST_C_1_pSK_SK	316
cl0228	ct0239	cn0244	pOP-CNH02166_EST_C_1_pSK_SK	590
cl0228	ct0239	cn0244	pOP-CNH04176	568
cl0228	ct0239	cn0244	pOP-CNH04263	536
cl0228	ct0239	cn0244	pOP-CNH04290	486
cl0228	ct0239	cn0244	pOP-CNI02070_EST_C_1_pSK_SK	393
cl0228	ct0239	cn0244	pOP-CNIP00151_EST_C_1_pSK_SK	545
cl0228	ct0239	cn0244	pOP-EO07857_EST_C_1_pSK_SK	602
cl0229	ct0240	cn0245	pOP-CNH01847_EST_C_1_pSK_SK	504
cl0229	ct0240	cn0245	pOP-CNH03277_EST_C_1_pSK_SK	617
cl0230	ct0241	cn0246	pOP-CNH01587_EST_C_1_pSK_SK	666
cl0230	ct0241	cn0246	pOP-CNH02750_EST_C_1_pSK_SK	605
cl0230	ct0241	cn0246	pOP-CNH03296_EST_C_1_pSK_SK	585
cl0231	ct0242	cn0247	pOP-CNH01166_EST_C_1_pSK_SK	376
cl0231	ct0242	cn0247	pOP-CNH03302_EST_C_1_pSK_SK	707
cl0232	ct0243	cn0248	pOP-CNH02862_EST_C_1_pSK_SK	647
cl0232	ct0243	cn0248	pOP-CNH03305_EST_C_1_pSK_SK	660
cl0233	ct0244	cn0249	pOP-CNH01215_EST_C_1_pSK_SK	445
cl0233	ct0244	cn0249	pOP-CNH03316_EST_C_1_pSK_SK	544
cl0234	ct0245	cn0250	pOP-CNH03320_EST_C_1_pSK_SK	529
cl0234	ct0245	cn0251	pOP-CNH04524	395
cl0235	ct0246	cn0252	pOP-CNH02103_EST_C_1_pSK_SK	575
cl0235	ct0246	cn0252	pOP-CNH02830_EST_C_1_pSK_SK	623
cl0235	ct0246	cn0252	pOP-CNH03321_EST_C_1_pSK_SK	674
cl0236	ct0247	cn0253	pOP-CNH01358_EST_C_1_pSK_SK	643
cl0236	ct0247	cn0253	pOP-CNH03329_EST_C_1_pSK_SK	628

cl0237	ct0248	cn0254	pOP-CNH01063_EST_C_1_pSK_SK	633
cl0237	ct0248	cn0254	pOP-CNH03332_EST_C_1_pSK_SK	734
cl0238	ct0249	cn0255	pOP-CNH02847_EST_C_1_pSK_SK	615
cl0238	ct0249	cn0255	pOP-CNH03340_EST_C_1_pSK_SK	697
cl0239	ct0250	cn0256	pOP-CNH00664_EST_C_1_pSK_SK	281
cl0239	ct0250	cn0256	pOP-CNH03362_EST_C_1_pSK_SK	552
cl0239	ct0250	cn0256	pOP-CNH03370_EST_C_1_pSK_SK	552
cl0239	ct0250	cn0256	pOP-CNH04353	538
cl0240	ct0251	cn0257	pOP-CNH03372_EST_C_1_pSK_SK	621
cl0240	ct0251	cn0257	pOP-CNH04453	796
cl0241	ct0252	cn0258	pOP-CNH03174_EST_C_1_pSK_SK	605
cl0241	ct0252	cn0258	pOP-CNH03373_EST_C_1_pSK_SK	563
cl0241	ct0252	cn0259	pOP-EO05120_EST_C_1_pSK_SK	512
cl0242	ct0253	cn0260	pOP-CNH02649_EST_C_1_pSK_SK	336
cl0242	ct0253	cn0260	pOP-CNH02930_EST_C_1_pSK_SK	386
cl0242	ct0253	cn0260	pOP-CNH03385_EST_C_1_pSK_SK	339
cl0243	ct0254	cn0261	pOP-CNH03169_EST_C_1_pSK_SK	472
cl0243	ct0254	cn0261	pOP-CNH03386_EST_C_1_pSK_SK	426
cl0244	ct0255	cn0262	pOP-CNH01246_EST_C_1_pSK_SK	535
cl0244	ct0255	cn0262	pOP-CNH03388_EST_C_1_pSK_SK	502
cl0245	ct0256	cn0263	pOP-CNH02049_EST_C_1_pSK_SK	553
cl0245	ct0256	cn0263	pOP-CNH02492_EST_C_1_pSK_SK	502
cl0245	ct0256	cn0263	pOP-CNH03394_EST_C_1_pSK_SK	517
cl0246	ct0257	cn0264	pOP-CNH01392_EST_C_1_pSK_SK	725
cl0246	ct0257	cn0264	pOP-CNH03397_EST_C_1_pSK_SK	448
cl0246	ct0257	cn0264	pOP-CNH04640	743
cl0247	ct0258	cn0265	pOP-CNH02691_EST_C_1_pSK_SK	587
cl0247	ct0258	cn0265	pOP-CNH03409_EST_C_1_pSK_SK	536
cl0248	ct0259	cn0266	pOP-CNH02975_EST_C_1_pSK_SK	592
cl0248	ct0260	cn0267	pOP-CNH03411_EST_C_1_pSK_SK	540
cl0249	ct0261	cn0268	pOP-CNH02430_EST_C_1_pSK_SK	542
cl0249	ct0261	cn0268	pOP-CNH03415_EST_C_1_pSK_SK	584
cl0250	ct0262	cn0269	pOP-CNH02281_EST_C_1_pSK_SK	710
cl0250	ct0262	cn0269	pOP-CNH03424_EST_C_1_pSK_SK	505
cl0250	ct0262	cn0269	pOP-CNH04638	698
cl0251	ct0263	cn0270	pOP-CNH01111_EST_C_1_pSK_SK	331
cl0251	ct0263	cn0270	pOP-CNH03432_EST_C_1_pSK_SK	606
cl0251	ct0263	cn0270	pOP-CNH04593	678
cl0252	ct0264	cn0271	pOP-CNH01206_EST_C_1_pSK_SK	506
cl0252	ct0265	cn0272	pOP-CNH01976_EST_C_1_pSK_SK	665
cl0252	ct0265	cn0272	pOP-CNH03291_EST_C_1_pSK_SK	591
cl0252	ct0266	cn0273	pOP-CNH03134_EST_C_1_pSK_SK	430
cl0252	ct0266	cn0273	pOP-CNH03499_EST_C_1_pSK_SK	649
cl0252	ct0266	cn0273	pOP-CNH04416	590
cl0252	ct0267	cn0274	pOP-CNH00787_EST_C_1_pSK_SK	569
cl0252	ct0267	cn0274	pOP-CNH01574_EST_C_1_pSK_SK	583
cl0252	ct0267	cn0275	pOP-CNH04768_EST_C_1_pSK_SK	561
cl0252	ct0267	cn0276	pOP-CNH01605_EST_C_1_pSK_SK	550
cl0252	ct0268	cn0277	pOP-CNH00978_EST_C_1_pSK_SK	589
cl0252	ct0268	cn0277	pOP-CNH01479_EST_C_1_pSK_SK	550
cl0252	ct0268	cn0277	pOP-CNH01881_EST_C_1_pSK_SK	381
cl0252	ct0268	cn0277	pOP-CNH02989_EST_C_1_pSK_SK	670
cl0252	ct0268	cn0277	pOP-CNH03393_EST_C_1_pSK_SK	492
cl0252	ct0268	cn0277	pOP-CNH04652	737
cl0252	ct0268	cn0278	pOP-CNHP00463_EST_C_1_pSK_SK	680
cl0252	ct0269	cn0279	pOP-CNH01469_EST_C_1_pSK_SK	477

cl0252	ct0269	cn0279	pOP-CNH01720_EST_C_1_pSK_SK	594
cl0252	ct0269	cn0279	pOP-CNH01766_EST_C_1_pSK_SK	557
cl0252	ct0269	cn0279	pOP-CNH01885_EST_C_1_pSK_SK	503
cl0252	ct0269	cn0279	pOP-CNH02818_EST_C_1_pSK_SK	257
cl0252	ct0269	cn0279	pOP-CNH03436_EST_C_1_pSK_SK	369
cl0252	ct0269	cn0279	pOP-CNH03438_EST_C_1_pSK_SK	311
cl0252	ct0269	cn0279	pOP-CNH03458_EST_C_1_pSK_SK	526
cl0252	ct0269	cn0279	pOP-CNH04556	683
cl0252	ct0269	cn0279	pOP-CNH04612	685
cl0252	ct0269	cn0279	pOP-CNH04627	733
cl0252	ct0269	cn0279	pOP-CNH04675	720
cl0252	ct0269	cn0279	pOP-CNHP00198_EST_C_1_pSK_SK	685
cl0253	ct0270	cn0280	pOP-CNH00759_EST_C_1_pSK_SK	280
cl0253	ct0270	cn0280	pOP-CNH03451_EST_C_1_pSK_SK	270
cl0254	ct0271	cn0281	pOP-CNH02616_EST_C_1_pSK_SK	337
cl0254	ct0271	cn0281	pOP-CNH03453_EST_C_1_pSK_SK	554
cl0255	ct0272	cn0282	pOP-CNH02994_EST_C_1_pSK_SK	359
cl0255	ct0272	cn0282	pOP-CNH03457_EST_C_1_pSK_SK	441
cl0256	ct0273	cn0283	pOP-CNH02109_EST_C_1_pSK_SK	538
cl0256	ct0273	cn0283	pOP-CNH02346_EST_C_1_pSK_SK	427
cl0256	ct0273	cn0283	pOP-CNH03462_EST_C_1_pSK_SK	482
cl0257	ct0274	cn0284	pOP-CNH01824_EST_C_1_pSK_SK	479
cl0257	ct0274	cn0284	pOP-CNH01888_EST_C_1_pSK_SK	479
cl0257	ct0274	cn0284	pOP-CNH03473_EST_C_1_pSK_SK	396
cl0257	ct0274	cn0284	pOP-CNH04367	818
cl0258	ct0275	cn0285	pOP-CNH01610_EST_C_1_pSK_SK	414
cl0258	ct0275	cn0285	pOP-CNH03478_EST_C_1_pSK_SK	497
cl0258	ct0275	cn0285	pOP-EO02950_EST_C_1_pSK_SK	295
cl0259	ct0276	cn0286	pOP-CNH03481_EST_C_1_pSK_SK	275
cl0259	ct0276	cn0286	pOP-CNH04215	682
cl0260	ct0277	cn0287	pOP-CNH01777_EST_C_1_pSK_SK	443
cl0260	ct0277	cn0287	pOP-CNH03485_EST_C_1_pSK_SK	635
cl0261	ct0278	cn0288	pOP-CNH03486_EST_C_1_pSK_SK	343
cl0261	ct0278	cn0288	pOP-CNH03488_EST_C_1_pSK_SK	544
cl0262	ct0279	cn0289	pOP-CNH02462_EST_C_1_pSK_SK	390
cl0262	ct0279	cn0289	pOP-CNH03262_EST_C_1_pSK_SK	625
cl0262	ct0279	cn0289	pOP-CNH03278_EST_C_1_pSK_SK	575
cl0262	ct0279	cn0289	pOP-CNH03490_EST_C_1_pSK_SK	604
cl0263	ct0280	cn0290	pOP-CNH02674_EST_C_1_pSK_SK	546
cl0263	ct0280	cn0290	pOP-CNH03491_EST_C_1_pSK_SK	420
cl0264	ct0281	cn0291	pOP-CNH01543_EST_C_1_pSK_SK	414
cl0264	ct0281	cn0291	pOP-CNH03496_EST_C_1_pSK_SK	343
cl0265	ct0282	cn0292	pOP-CNH02810_EST_C_1_pSK_SK	508
cl0265	ct0282	cn0292	pOP-CNH03500_EST_C_1_pSK_SK	631
cl0266	ct0283	cn0293	pOP-CNH04204	617
cl0266	ct0283	cn0293	pOP-CNH04376	880
cl0266	ct0284	cn0294	pOP-CNH03511_EST_C_1_pSK_SK	346
cl0266	ct0284	cn0294	pOP-CNH04230	662
cl0266	ct0284	cn0294	pOP-CNH04337	735
cl0267	ct0285	cn0295	pOP-CNH01498_EST_C_1_pSK_SK	620
cl0267	ct0285	cn0295	pOP-CNH01923_EST_C_1_pSK_SK	709
cl0267	ct0285	cn0295	pOP-CNH03516_EST_C_1_pSK_SK	563
cl0268	ct0286	cn0296	pOP-CNH02953_EST_C_1_pSK_SK	634
cl0268	ct0286	cn0296	pOP-CNH03527_EST_C_1_pSK_SK	553
cl0269	ct0287	cn0297	pOP-CNH02467_EST_C_1_pSK_SK	390
cl0269	ct0287	cn0297	pOP-CNH03366_EST_C_1_pSK_SK	672

cl0269	ct0287	cn0297	pOP-CNH03529_EST_C_1_pSK_SK	646
cl0269	ct0287	cn0297	pOP-CNH04641	660
cl0270	ct0288	cn0298	pOP-CNH02080_EST_C_1_pSK_SK	454
cl0270	ct0288	cn0298	pOP-CNH03543_EST_C_1_pSK_SK	340
cl0271	ct0289	cn0299	pOP-CNH01354_EST_C_1_pSK_SK	635
cl0271	ct0289	cn0299	pOP-CNH01609_EST_C_1_pSK_SK	491
cl0271	ct0289	cn0299	pOP-CNH01712_EST_C_1_pSK_SK	494
cl0271	ct0289	cn0299	pOP-CNH03547_EST_C_1_pSK_SK	556
cl0272	ct0290	cn0300	pOP-CNH01373_EST_C_1_pSK_SK	582
cl0272	ct0290	cn0300	pOP-CNH01439_EST_C_1_pSK_SK	349
cl0272	ct0290	cn0300	pOP-CNH02261_EST_C_1_pSK_SK	575
cl0272	ct0290	cn0300	pOP-CNH03553_EST_C_1_pSK_SK	286
cl0272	ct0290	cn0300	pOP-CNH04642	646
cl0273	ct0291	cn0301	pOP-CNH01562_EST_C_1_pSK_SK	696
cl0273	ct0292	cn0302	pOP-CNH01822_EST_C_1_pSK_SK	474
cl0273	ct0292	cn0302	pOP-CNH03163_EST_C_1_pSK_SK	564
cl0273	ct0292	cn0302	pOP-CNH03377_EST_C_1_pSK_SK	565
cl0273	ct0292	cn0302	pOP-CNH03557_EST_C_1_pSK_SK	282
cl0274	ct0293	cn0303	pOP-CNH01388_EST_C_1_pSK_SK	730
cl0274	ct0293	cn0303	pOP-CNH01966_EST_C_1_pSK_SK	684
cl0274	ct0293	cn0303	pOP-CNH03562_EST_C_1_pSK_SK	352
cl0275	ct0294	cn0304	pOP-CNH01865_EST_C_1_pSK_SK	626
cl0275	ct0294	cn0304	pOP-CNH02021_EST_C_1_pSK_SK	603
cl0275	ct0294	cn0304	pOP-CNH03256_EST_C_1_pSK_SK	587
cl0275	ct0294	cn0304	pOP-CNH03566_EST_C_1_pSK_SK	378
cl0276	ct0295	cn0305	pOP-CNH01031_EST_C_1_pSK_SK	414
cl0276	ct0295	cn0305	pOP-CNH01808_EST_C_1_pSK_SK	435
cl0276	ct0295	cn0305	pOP-CNH02952_EST_C_1_pSK_SK	613
cl0276	ct0295	cn0305	pOP-CNH03492_EST_C_1_pSK_SK	585
cl0276	ct0295	cn0305	pOP-CNH03567_EST_C_1_pSK_SK	296
cl0276	ct0295	cn0305	pOP-CNH05000_EST_C_1_pSK_SK	683
cl0276	ct0295	cn0305	pOP-CNI01599_EST_C_1_pSK_SK	539
cl0277	ct0296	cn0306	pOP-CNH02123_EST_C_1_pSK_SK	480
cl0277	ct0296	cn0306	pOP-CNH03574_EST_C_1_pSK_SK	348
cl0278	ct0297	cn0307	pOP-CNH01152_EST_C_1_pSK_SK	373
cl0278	ct0297	cn0307	pOP-CNH03576_EST_C_1_pSK_SK	341
cl0279	ct0298	cn0308	pOP-CNH01947_EST_C_1_pSK_SK	775
cl0279	ct0298	cn0308	pOP-CNH03422_EST_C_1_pSK_SK	465
cl0279	ct0298	cn0308	pOP-CNH03579_EST_C_1_pSK_SK	347
cl0279	ct0298	cn0308	pOP-CNH04142	456
cl0279	ct0298	cn0308	pOP-CNH04422	468
cl0280	ct0299	cn0309	pOP-CNH03166_EST_C_1_pSK_SK	569
cl0280	ct0299	cn0309	pOP-CNH03387_EST_C_1_pSK_SK	525
cl0280	ct0299	cn0309	pOP-CNH03583_EST_C_1_pSK_SK	352
cl0281	ct0300	cn0310	pOP-CNH02136_EST_C_1_pSK_SK	485
cl0281	ct0300	cn0310	pOP-CNH02653_EST_C_1_pSK_SK	643
cl0281	ct0300	cn0310	pOP-CNH03588_EST_C_1_pSK_SK	350
cl0281	ct0300	cn0310	pOP-CNH04370	794
cl0282	ct0301	cn0311	pOP-CNH01759_EST_C_1_pSK_SK	557
cl0282	ct0301	cn0311	pOP-CNH03592_EST_C_1_pSK_SK	351
cl0283	ct0302	cn0312	pOP-CNH03330_EST_C_1_pSK_SK	710
cl0283	ct0303	cn0313	pOP-CNH01541_EST_C_1_pSK_SK	602
cl0283	ct0303	cn0313	pOP-CNH03599_EST_C_1_pSK_SK	351
cl0284	ct0304	cn0314	pOP-CNH03090_EST_C_1_pSK_SK	638
cl0284	ct0304	cn0314	pOP-CNH03604_EST_C_1_pSK_SK	349
cl0284	ct0304	cn0314	pOP-CNH04507	768

cl0285	ct0305	cn0315	pOP-CNH00997_EST_C_1_pSK_SK	402
cl0285	ct0305	cn0315	pOP-CNH03607_EST_C_1_pSK_SK	355
cl0286	ct0306	cn0316	pOP-CNH00743_EST_C_1_pSK_SK	420
cl0286	ct0306	cn0316	pOP-CNH03606_EST_C_1_pSK_SK	467
cl0286	ct0306	cn0316	pOP-CNH03608_EST_C_1_pSK_SK	347
cl0287	ct0307	cn0317	pOP-CNH03004_EST_C_1_pSK_SK	539
cl0287	ct0307	cn0317	pOP-CNH03153_EST_C_1_pSK_SK	568
cl0287	ct0307	cn0317	pOP-CNH03613_EST_C_1_pSK_SK	584
cl0287	ct0307	cn0317	pOP-CNH04412	771
cl0288	ct0308	cn0318	pOP-CNH03598_EST_C_1_pSK_SK	512
cl0288	ct0308	cn0318	pOP-CNH03614_EST_C_1_pSK_SK	564
cl0289	ct0309	cn0319	pOP-CNH03589_EST_C_1_pSK_SK	407
cl0289	ct0309	cn0319	pOP-CNH03623_EST_C_1_pSK_SK	404
cl0289	ct0309	cn0319	pOP-CNH03630_EST_C_1_pSK_SK	681
cl0290	ct0310	cn0320	pOP-CNH01592_EST_C_1_pSK_SK	446
cl0290	ct0310	cn0320	pOP-CNH03631_EST_C_1_pSK_SK	632
cl0290	ct0310	cn0320	pOP-EO02357_EST_C_1_pSK_SK	630
cl0291	ct0311	cn0321	pOP-CNH01422_EST_C_1_pSK_SK	608
cl0291	ct0311	cn0321	pOP-CNH02257_EST_C_1_pSK_SK	648
cl0291	ct0311	cn0321	pOP-CNH02394_EST_C_1_pSK_SK	540
cl0291	ct0311	cn0321	pOP-CNH02521_EST_C_1_pSK_SK	509
cl0291	ct0311	cn0321	pOP-CNH03515_EST_C_1_pSK_SK	334
cl0291	ct0311	cn0321	pOP-CNH03632_EST_C_1_pSK_SK	624
cl0291	ct0311	cn0321	pOP-CNH04360	852
cl0291	ct0311	cn0321	pOP-CNH04705	764
cl0291	ct0311	cn0321	pOP-CNH04728_EST_C_1_pSK_SK	628
cl0291	ct0311	cn0321	pOP-CNH04742_EST_C_1_pSK_SK	492
cl0292	ct0312	cn0322	pOP-CNH02791_EST_C_1_pSK_SK	618
cl0292	ct0312	cn0322	pOP-CNH02809_EST_C_1_pSK_SK	643
cl0292	ct0312	cn0322	pOP-CNH03637_EST_C_1_pSK_SK	348
cl0293	ct0313	cn0323	pOP-CNH02996_EST_C_1_pSK_SK	571
cl0293	ct0313	cn0323	pOP-CNH03096_EST_C_1_pSK_SK	603
cl0293	ct0313	cn0323	pOP-CNH03638_EST_C_1_pSK_SK	686
cl0294	ct0314	cn0324	pOP-CNH03643_EST_C_1_pSK_SK	688
cl0294	ct0314	cn0325	pOP-CNH02905_EST_C_1_pSK_SK	497
cl0295	ct0315	cn0326	pOP-CNH00920_EST_C_1_pSK_SK	727
cl0295	ct0315	cn0326	pOP-CNH01832_EST_C_1_pSK_SK	583
cl0295	ct0315	cn0326	pOP-CNH01895_EST_C_1_pSK_SK	675
cl0295	ct0315	cn0326	pOP-CNH02534_EST_C_1_pSK_SK	453
cl0295	ct0315	cn0326	pOP-CNH03085_EST_C_1_pSK_SK	717
cl0295	ct0315	cn0326	pOP-CNH03651_EST_C_1_pSK_SK	485
cl0296	ct0316	cn0327	pOP-CNH02559_EST_C_1_pSK_SK	544
cl0296	ct0316	cn0327	pOP-CNH02560_EST_C_1_pSK_SK	556
cl0296	ct0316	cn0327	pOP-CNH03653_EST_C_1_pSK_SK	251
cl0297	ct0317	cn0328	pOP-CNH01880_EST_C_1_pSK_SK	416
cl0297	ct0317	cn0328	pOP-CNH03659_EST_C_1_pSK_SK	480
cl0298	ct0318	cn0329	pOP-CNH01971_EST_C_1_pSK_SK	577
cl0298	ct0318	cn0329	pOP-CNH03660_EST_C_1_pSK_SK	483
cl0299	ct0319	cn0330	pOP-CNH02070_EST_C_1_pSK_SK	612
cl0299	ct0319	cn0330	pOP-CNH03668_EST_C_1_pSK_SK	382
cl0299	ct0319	cn0330	pOP-CNH04425	704
cl0299	ct0319	cn0330	pOP-CNH04666	753
cl0300	ct0320	cn0331	pOP-CNH03568_EST_C_1_pSK_SK	349
cl0300	ct0320	cn0331	pOP-CNH03597_EST_C_1_pSK_SK	345
cl0300	ct0320	cn0331	pOP-CNH03672_EST_C_1_pSK_SK	494
cl0301	ct0321	cn0332	pOP-CNH00596_EST_C_1_pSK_SK	666

cl0301	ct0321	cn0332	pOP-CNH02011_EST_C_1_pSK_SK	628
cl0301	ct0321	cn0332	pOP-CNH02464_EST_C_1_pSK_SK	460
cl0301	ct0321	cn0332	pOP-CNH03678_EST_C_1_pSK_SK	375
cl0301	ct0321	cn0332	pOP-CNH03680_EST_C_1_pSK_SK	374
cl0302	ct0322	cn0333	pOP-CNH03326_EST_C_1_pSK_SK	536
cl0302	ct0322	cn0333	pOP-CNH03681_EST_C_1_pSK_SK	305
cl0302	ct0322	cn0333	pOP-CNH04381	535
cl0303	ct0323	cn0334	pOP-CNH03679_EST_C_1_pSK_SK	320
cl0303	ct0323	cn0334	pOP-CNH03683_EST_C_1_pSK_SK	326
cl0304	ct0324	cn0335	pOP-CNH03686_EST_C_1_pSK_SK	568
cl0304	ct0324	cn0335	pOP-CNH03688_EST_C_1_pSK_SK	422
cl0305	ct0325	cn0336	pOP-CNH02015_EST_C_1_pSK_SK	512
cl0305	ct0325	cn0336	pOP-CNH03697_EST_C_1_pSK_SK	486
cl0305	ct0325	cn0336	pOP-CNH03698_EST_C_1_pSK_SK	486
cl0306	ct0326	cn0337	pOP-CNH03164_EST_C_1_pSK_SK	675
cl0306	ct0326	cn0337	pOP-CNH03699_EST_C_1_pSK_SK	483
cl0306	ct0326	cn0337	pOP-CNH04294	142
cl0306	ct0326	cn0337	pOP-CNH04548	759
cl0306	ct0326	cn0337	pOP-CNH04566	746
cl0307	ct0327	cn0338	pOP-CNH03695_EST_C_1_pSK_SK	500
cl0307	ct0327	cn0338	pOP-CNH03696_EST_C_1_pSK_SK	460
cl0307	ct0327	cn0338	pOP-CNH03701_EST_C_1_pSK_SK	401
cl0308	ct0328	cn0339	pOP-CNH01771_EST_C_1_pSK_SK	521
cl0308	ct0328	cn0339	pOP-CNH03308_EST_C_1_pSK_SK	581
cl0308	ct0328	cn0339	pOP-CNH03705_EST_C_1_pSK_SK	581
cl0308	ct0328	cn0339	pOP-CNH03707_EST_C_1_pSK_SK	380
cl0309	ct0329	cn0340	pOP-CNH01651_EST_C_1_pSK_SK	649
cl0309	ct0329	cn0340	pOP-CNH03577_EST_C_1_pSK_SK	479
cl0309	ct0329	cn0340	pOP-CNH03636_EST_C_1_pSK_SK	675
cl0309	ct0329	cn0340	pOP-CNH03718_EST_C_1_pSK_SK	405
cl0310	ct0330	cn0341	pOP-CNH03546_EST_C_1_pSK_SK	467
cl0310	ct0330	cn0341	pOP-CNH03720_EST_C_1_pSK_SK	282
cl0310	ct0330	cn0341	pOP-CNH03721_EST_C_1_pSK_SK	282
cl0310	ct0330	cn0341	pOP-CNI01739_EST_C_1_pSK_SK	103
cl0311	ct0331	cn0342	pOP-CNH03722_EST_C_1_pSK_SK	495
cl0311	ct0331	cn0342	pOP-CNH03723_EST_C_1_pSK_SK	398
cl0312	ct0332	cn0343	pOP-CNH03452_EST_C_1_pSK_SK	598
cl0312	ct0332	cn0343	pOP-CNH03601_EST_C_1_pSK_SK	529
cl0312	ct0332	cn0343	pOP-CNH03731_EST_C_1_pSK_SK	163
cl0312	ct0332	cn0343	pOP-CNH03732_EST_C_1_pSK_SK	517
cl0312	ct0332	cn0343	pOP-CNH04657	607
cl0313	ct0333	cn0344	pOP-CNH01132_EST_C_1_pSK_SK	460
cl0313	ct0333	cn0344	pOP-CNH01760_EST_C_1_pSK_SK	421
cl0313	ct0333	cn0344	pOP-CNH02140_EST_C_1_pSK_SK	539
cl0313	ct0333	cn0344	pOP-CNH02152_EST_C_1_pSK_SK	439
cl0313	ct0333	cn0344	pOP-CNH03733_EST_C_1_pSK_SK	490
cl0314	ct0334	cn0345	pOP-CNH00663_EST_C_1_pSK_SK	366
cl0314	ct0334	cn0345	pOP-CNH00784_EST_C_1_pSK_SK	392
cl0314	ct0334	cn0345	pOP-CNH01331_EST_C_1_pSK_SK	698
cl0314	ct0334	cn0345	pOP-CNH01499_EST_C_1_pSK_SK	600
cl0314	ct0334	cn0345	pOP-CNH01736_EST_C_1_pSK_SK	574
cl0314	ct0334	cn0345	pOP-CNH02397_EST_C_1_pSK_SK	519
cl0314	ct0334	cn0345	pOP-CNH02940_EST_C_1_pSK_SK	592
cl0314	ct0334	cn0345	pOP-CNH03217_EST_C_1_pSK_SK	367
cl0314	ct0334	cn0345	pOP-CNH03734_EST_C_1_pSK_SK	401
cl0314	ct0334	cn0345	pOP-CNH03735_EST_C_1_pSK_SK	401

cl0314	ct0334	cn0345	pOP-CNHP00045_EST_C_1_pSK_SK	572
cl0314	ct0334	cn0345	pOP-CNI02247_EST_C_1_pSK_SK	561
cl0314	ct0334	cn0345	pOP-CNIP00835_EST_C_1_pSK_SK	580
cl0314	ct0334	cn0345	pOP-EO06995_EST_C_1_pSK_SK	682
cl0314	ct0334	cn0345	pOP-EO07770_EST_C_1_pSK_SK	764
cl0315	ct0335	cn0346	pOP-CNH01171_EST_C_1_pSK_SK	444
cl0315	ct0335	cn0346	pOP-CNH03341_EST_C_1_pSK_SK	598
cl0315	ct0335	cn0346	pOP-CNH03737_EST_C_1_pSK_SK	298
cl0315	ct0335	cn0346	pOP-CNH03738_EST_C_1_pSK_SK	364
cl0316	ct0336	cn0347	pOP-CNH02912_EST_C_1_pSK_SK	626
cl0316	ct0336	cn0347	pOP-CNH03554_EST_C_1_pSK_SK	493
cl0316	ct0336	cn0347	pOP-CNH03658_EST_C_1_pSK_SK	343
cl0316	ct0336	cn0347	pOP-CNH03739_EST_C_1_pSK_SK	312
cl0317	ct0337	cn0348	pOP-CNH03741_EST_C_1_pSK_SK	401
cl0317	ct0337	cn0348	pOP-CNH03742_EST_C_1_pSK_SK	226
cl0318	ct0338	cn0349	pOP-CNH01774_EST_C_1_pSK_SK	506
cl0318	ct0338	cn0349	pOP-CNH02147_EST_C_1_pSK_SK	581
cl0318	ct0338	cn0349	pOP-CNH03744_EST_C_1_pSK_SK	577
cl0319	ct0339	cn0350	pOP-CNH01361_EST_C_1_pSK_SK	590
cl0319	ct0339	cn0350	pOP-CNH02558_EST_C_1_pSK_SK	499
cl0319	ct0339	cn0350	pOP-CNH02566_EST_C_1_pSK_SK	530
cl0319	ct0339	cn0350	pOP-CNH02680_EST_C_1_pSK_SK	636
cl0319	ct0339	cn0350	pOP-CNH03749_EST_C_1_pSK_SK	465
cl0320	ct0340	cn0351	pOP-CNH00775_EST_C_1_pSK_SK	368
cl0320	ct0340	cn0351	pOP-CNH03753_EST_C_1_pSK_SK	485
cl0320	ct0340	cn0351	pOP-CNH04417	826
cl0321	ct0341	cn0352	pOP-CNH01383_EST_C_1_pSK_SK	702
cl0321	ct0341	cn0352	pOP-CNH03760_EST_C_1_pSK_SK	541
cl0321	ct0341	cn0352	pOP-CNH04485	696
cl0322	ct0342	cn0353	pOP-CNH03765_EST_C_1_pSK_SK	344
cl0322	ct0342	cn0353	pOP-CNH04564	513
cl0323	ct0343	cn0354	pOP-CNH03764_EST_C_1_pSK_SK	526
cl0323	ct0343	cn0354	pOP-CNH03768_EST_C_1_pSK_SK	413
cl0324	ct0344	cn0355	pOP-CNH03783_EST_C_1_pSK_SK	545
cl0324	ct0344	cn0355	pOP-CNH04493	828
cl0325	ct0345	cn0356	pOP-CNH03782_EST_C_1_pSK_SK	546
cl0325	ct0345	cn0356	pOP-CNH03784_EST_C_1_pSK_SK	528
cl0326	ct0346	cn0357	pOP-CNH03781_EST_C_1_pSK_SK	558
cl0326	ct0346	cn0357	pOP-CNH03785_EST_C_1_pSK_SK	546
cl0327	ct0347	cn0358	pOP-CNH02119_EST_C_1_pSK_SK	466
cl0327	ct0347	cn0358	pOP-CNH03791_EST_C_1_pSK_SK	521
cl0328	ct0348	cn0359	pOP-CNH02054_EST_C_1_pSK_SK	508
cl0328	ct0348	cn0359	pOP-CNH03801_EST_C_1_pSK_SK	502
cl0329	ct0349	cn0360	pOP-CNH02323_EST_C_1_pSK_SK	503
cl0329	ct0349	cn0360	pOP-CNH03804_EST_C_1_pSK_SK	583
cl0330	ct0350	cn0361	pOP-CNH01175_EST_C_1_pSK_SK	496
cl0330	ct0350	cn0361	pOP-CNH03810_EST_C_1_pSK_SK	498
cl0331	ct0351	cn0362	pOP-CNH03060_EST_C_1_pSK_SK	489
cl0331	ct0351	cn0362	pOP-CNH03817_EST_C_1_pSK_SK	476
cl0332	ct0352	cn0363	pOP-CNH00931_EST_C_1_pSK_SK	407
cl0332	ct0352	cn0363	pOP-CNH02738_EST_C_1_pSK_SK	601
cl0332	ct0352	cn0363	pOP-CNH03818_EST_C_1_pSK_SK	334
cl0333	ct0353	cn0364	pOP-CNH03582_EST_C_1_pSK_SK	350
cl0333	ct0353	cn0364	pOP-CNH04714_EST_C_1_pSK_SK	476
cl0334	ct0354	cn0365	pOP-CNH03186_EST_C_1_pSK_SK	543
cl0334	ct0354	cn0365	pOP-CNH04243	573

cl0334	ct0354	cn0365	pOP-CNH04722_EST_C_1_pSK_SK	365
cl0335	ct0355	cn0366	pOP-CNH01364_EST_C_1_pSK_SK	629
cl0335	ct0355	cn0366	pOP-CNH04418	804
cl0335	ct0355	cn0366	pOP-CNH04737_EST_C_1_pSK_SK	502
cl0336	ct0356	cn0367	pOP-CNH01153_EST_C_1_pSK_SK	326
cl0336	ct0356	cn0367	pOP-CNH01956_EST_C_1_pSK_SK	326
cl0336	ct0356	cn0367	pOP-CNH02038_EST_C_1_pSK_SK	548
cl0336	ct0356	cn0367	pOP-CNH02853_EST_C_1_pSK_SK	588
cl0336	ct0356	cn0367	pOP-CNH04746_EST_C_1_pSK_SK	325
cl0337	ct0357	cn0368	pOP-CNH01950_EST_C_1_pSK_SK	425
cl0337	ct0357	cn0368	pOP-CNH04747_EST_C_1_pSK_SK	406
cl0338	ct0358	cn0369	pOP-CNH02264_EST_C_1_pSK_SK	648
cl0338	ct0358	cn0369	pOP-CNH04753_EST_C_1_pSK_SK	564
cl0339	ct0359	cn0370	pOP-CNH02404_EST_C_1_pSK_SK	478
cl0339	ct0359	cn0370	pOP-CNH02510_EST_C_1_pSK_SK	472
cl0339	ct0359	cn0370	pOP-CNH03605_EST_C_1_pSK_SK	320
cl0339	ct0359	cn0370	pOP-CNH04484	769
cl0339	ct0359	cn0370	pOP-CNH04754_EST_C_1_pSK_SK	488
cl0340	ct0360	cn0371	pOP-CNH02265_EST_C_1_pSK_SK	571
cl0340	ct0360	cn0371	pOP-CNH04135	484
cl0340	ct0360	cn0371	pOP-CNH04766_EST_C_1_pSK_SK	548
cl0341	ct0361	cn0372	pOP-CNH02452_EST_C_1_pSK_SK	454
cl0341	ct0361	cn0372	pOP-CNH04776_EST_C_1_pSK_SK	598
cl0342	ct0362	cn0373	pOP-CNH01711_EST_C_1_pSK_SK	339
cl0342	ct0362	cn0373	pOP-CNH04786_EST_C_1_pSK_SK	339
cl0343	ct0363	cn0374	pOP-CNH04202	578
cl0343	ct0363	cn0374	pOP-CNH04792_EST_C_1_pSK_SK	588
cl0344	ct0364	cn0375	pOP-CNH04226	531
cl0344	ct0364	cn0375	pOP-CNH04793_EST_C_1_pSK_SK	582
cl0345	ct0365	cn0376	pOP-CNH03076_EST_C_1_pSK_SK	497
cl0345	ct0365	cn0376	pOP-CNH04511	496
cl0345	ct0365	cn0376	pOP-CNH04518	497
cl0345	ct0365	cn0376	pOP-CNH04796_EST_C_1_pSK_SK	513
cl0346	ct0366	cn0377	pOP-CNH01325_EST_C_1_pSK_SK	725
cl0346	ct0366	cn0377	pOP-CNH04807_EST_C_1_pSK_SK	588
cl0347	ct0367	cn0378	pOP-CNH01872_EST_C_1_pSK_SK	449
cl0347	ct0367	cn0378	pOP-CNH04875_EST_C_1_pSK_SK	830
cl0348	ct0368	cn0379	pOP-CNH02383_EST_C_1_pSK_SK	461
cl0348	ct0368	cn0379	pOP-CNH04877_EST_C_1_pSK_SK	763
cl0349	ct0369	cn0380	pOP-CNH01036_EST_C_1_pSK_SK	234
cl0349	ct0369	cn0380	pOP-CNH04880_EST_C_1_pSK_SK	819
cl0350	ct0370	cn0381	pOP-CNH01046_EST_C_1_pSK_SK	661
cl0350	ct0370	cn0381	pOP-CNH01191_EST_C_1_pSK_SK	550
cl0350	ct0370	cn0381	pOP-CNH01376_EST_C_1_pSK_SK	617
cl0350	ct0370	cn0381	pOP-CNH04881_EST_C_1_pSK_SK	696
cl0351	ct0371	cn0382	pOP-CNH02864_EST_C_1_pSK_SK	643
cl0351	ct0371	cn0382	pOP-CNH03425_EST_C_1_pSK_SK	574
cl0351	ct0371	cn0382	pOP-CNH03774_EST_C_1_pSK_SK	494
cl0351	ct0371	cn0382	pOP-CNH04883_EST_C_1_pSK_SK	312
cl0352	ct0372	cn0383	pOP-CNH01130_EST_C_1_pSK_SK	222
cl0352	ct0372	cn0383	pOP-CNH01450_EST_C_1_pSK_SK	432
cl0352	ct0372	cn0383	pOP-CNH02655_EST_C_1_pSK_SK	480
cl0352	ct0372	cn0383	pOP-CNH04884_EST_C_1_pSK_SK	528
cl0353	ct0373	cn0384	pOP-CNH01187_EST_C_1_pSK_SK	605
cl0353	ct0373	cn0384	pOP-CNH04900_EST_C_1_pSK_SK	440
cl0354	ct0374	cn0385	pOP-CNH01502_EST_C_1_pSK_SK	535

cl0354	ct0374	cn0385	pOP-CNH04905_EST_C_1_pSK_SK	771
cl0355	ct0375	cn0386	pOP-CNH02310_EST_C_1_pSK_SK	524
cl0355	ct0375	cn0386	pOP-CNH04203	581
cl0355	ct0375	cn0386	pOP-CNH04906_EST_C_1_pSK_SK	677
cl0356	ct0376	cn0387	pOP-CNH00858_EST_C_1_pSK_SK	544
cl0356	ct0376	cn0387	pOP-CNH02336_EST_C_1_pSK_SK	504
cl0356	ct0376	cn0387	pOP-CNH04394	528
cl0356	ct0376	cn0387	pOP-CNH04909_EST_C_1_pSK_SK	562
cl0357	ct0377	cn0388	pOP-CNH03293_EST_C_1_pSK_SK	548
cl0357	ct0377	cn0388	pOP-CNH04145	620
cl0357	ct0377	cn0388	pOP-CNH04351	640
cl0357	ct0377	cn0388	pOP-CNH04915_EST_C_1_pSK_SK	713
cl0358	ct0378	cn0389	pOP-CNH03038_EST_C_1_pSK_SK	536
cl0358	ct0378	cn0389	pOP-CNH04551	856
cl0358	ct0378	cn0389	pOP-CNH04607	861
cl0358	ct0378	cn0389	pOP-CNH04923_EST_C_1_pSK_SK	756
cl0359	ct0379	cn0390	pOP-CNH01749_EST_C_1_pSK_SK	543
cl0359	ct0379	cn0390	pOP-CNH04928_EST_C_1_pSK_SK	755
cl0360	ct0380	cn0391	pOP-CNH01999_EST_C_1_pSK_SK	512
cl0360	ct0380	cn0391	pOP-CNH04936_EST_C_1_pSK_SK	759
cl0361	ct0381	cn0392	pOP-CNH04219	260
cl0361	ct0381	cn0392	pOP-CNH04949_EST_C_1_pSK_SK	742
cl0362	ct0382	cn0393	pOP-CNH04253	322
cl0362	ct0382	cn0393	pOP-CNH04951_EST_C_1_pSK_SK	745
cl0363	ct0383	cn0394	pOP-CNH00845_EST_C_1_pSK_SK	593
cl0363	ct0383	cn0394	pOP-CNH00956_EST_C_1_pSK_SK	631
cl0363	ct0383	cn0394	pOP-CNH02914_EST_C_1_pSK_SK	604
cl0363	ct0383	cn0394	pOP-CNH04953_EST_C_1_pSK_SK	745
cl0364	ct0384	cn0395	pOP-CNH01169_EST_C_1_pSK_SK	545
cl0364	ct0384	cn0395	pOP-CNH01552_EST_C_1_pSK_SK	492
cl0364	ct0384	cn0395	pOP-CNH02033_EST_C_1_pSK_SK	504
cl0364	ct0384	cn0395	pOP-CNH02202_EST_C_1_pSK_SK	550
cl0364	ct0384	cn0395	pOP-CNH03311_EST_C_1_pSK_SK	591
cl0364	ct0384	cn0395	pOP-CNH04955_EST_C_1_pSK_SK	580
cl0365	ct0385	cn0396	pOP-CEO02877_EST_C_1_pSK_SK	503
cl0365	ct0385	cn0396	pOP-CNH04963_EST_C_1_pSK_SK	696
cl0365	ct0385	cn0396	pOP-EO03765_EST_C_1_pSK_SK	464
cl0365	ct0385	cn0396	pOP-EO04313_EST_C_1_pSK_SK	471
cl0366	ct0386	cn0397	pOP-CNH01362_EST_C_1_pSK_SK	657
cl0366	ct0386	cn0397	pOP-CNH02145_EST_C_1_pSK_SK	539
cl0366	ct0386	cn0397	pOP-CNH03208_EST_C_1_pSK_SK	580
cl0366	ct0386	cn0397	pOP-CNH04968_EST_C_1_pSK_SK	711
cl0367	ct0387	cn0398	pOP-CNH04979_EST_C_1_pSK_SK	650
cl0367	ct0387	cn0398	pOP-CNH04980_EST_C_1_pSK_SK	657
cl0368	ct0388	cn0399	pOP-CNH02436_EST_C_1_pSK_SK	524
cl0368	ct0388	cn0399	pOP-CNH04983_EST_C_1_pSK_SK	539
cl0369	ct0389	cn0400	pOP-CNH01093_EST_C_1_pSK_SK	392
cl0369	ct0389	cn0400	pOP-CNH01516_EST_C_1_pSK_SK	455
cl0369	ct0389	cn0400	pOP-CNH02327_EST_C_1_pSK_SK	453
cl0369	ct0389	cn0400	pOP-CNH04558	764
cl0369	ct0389	cn0400	pOP-CNH04989_EST_C_1_pSK_SK	467
cl0370	ct0390	cn0401	pOP-CNH02101_EST_C_1_pSK_SK	356
cl0370	ct0390	cn0401	pOP-CNH03642_EST_C_1_pSK_SK	651
cl0370	ct0390	cn0401	pOP-CNH04990_EST_C_1_pSK_SK	699
cl0371	ct0391	cn0402	pOP-CNH00933_EST_C_1_pSK_SK	481
cl0371	ct0391	cn0402	pOP-CNH05005_EST_C_1_pSK_SK	676

cl0372	ct0392	cn0403	pOP-CNH03575_EST_C_1_pSK_SK	339
cl0372	ct0392	cn0403	pOP-CNH05008_EST_C_1_pSK_SK	710
cl0373	ct0393	cn0404	pOP-CNH04421	749
cl0373	ct0393	cn0404	pOP-CNH05032_EST_C_1_pSK_SK	748
cl0374	ct0394	cn0405	pOP-CNH02271_EST_C_1_pSK_SK	695
cl0374	ct0394	cn0405	pOP-CNH05033_EST_C_1_pSK_SK	209
cl0375	ct0395	cn0406	pOP-CNH02186_EST_C_1_pSK_SK	524
cl0375	ct0395	cn0406	pOP-CNH04442	684
cl0375	ct0395	cn0406	pOP-CNH04745_EST_C_1_pSK_SK	573
cl0375	ct0395	cn0406	pOP-CNH05027_EST_C_1_pSK_SK	656
cl0375	ct0395	cn0406	pOP-CNH05035_EST_C_1_pSK_SK	675
cl0376	ct0396	cn0407	pOP-CNH05028_EST_C_1_pSK_SK	674
cl0376	ct0396	cn0407	pOP-CNH05036_EST_C_1_pSK_SK	674
cl0377	ct0397	cn0408	pOP-CNH01467_EST_C_1_pSK_SK	349
cl0377	ct0397	cn0408	pOP-CNH03189_EST_C_1_pSK_SK	453
cl0377	ct0397	cn0408	pOP-CNH05044_EST_C_1_pSK_SK	537
cl0378	ct0398	cn0409	pOP-CNH04942_EST_C_1_pSK_SK	782
cl0378	ct0398	cn0409	pOP-CNH05051_EST_C_1_pSK_SK	762
cl0379	ct0399	cn0410	pOP-CNH02509_EST_C_1_pSK_SK	351
cl0379	ct0399	cn0410	pOP-CNH04347	769
cl0379	ct0399	cn0410	pOP-CNH05054_EST_C_1_pSK_SK	671
cl0380	ct0400	cn0411	pOP-CNH03412_EST_C_1_pSK_SK	529
cl0380	ct0400	cn0411	pOP-CNH05060_EST_C_1_pSK_SK	629
cl0381	ct0401	cn0412	pOP-CNH02039_EST_C_1_pSK_SK	596
cl0381	ct0401	cn0412	pOP-CNH04727_EST_C_1_pSK_SK	429
cl0381	ct0401	cn0412	pOP-CNH05063_EST_C_1_pSK_SK	511
cl0382	ct0402	cn0413	pOP-CNH02274_EST_C_1_pSK_SK	666
cl0382	ct0402	cn0413	pOP-CNH04245	542
cl0382	ct0402	cn0413	pOP-CNH05065_EST_C_1_pSK_SK	618
cl0383	ct0403	cn0414	pOP-CNH01604_EST_C_1_pSK_SK	446
cl0383	ct0403	cn0414	pOP-CNH05068_EST_C_1_pSK_SK	630
cl0384	ct0404	cn0415	pOP-CNH03124_EST_C_1_pSK_SK	617
cl0384	ct0404	cn0415	pOP-CNH05079_EST_C_1_pSK_SK	623
cl0385	ct0405	cn0416	pOP-CNH01492_EST_C_1_pSK_SK	644
cl0385	ct0405	cn0416	pOP-CNH05080_EST_C_1_pSK_SK	606
cl0386	ct0406	cn0417	pOP-CNH04288	547
cl0386	ct0406	cn0417	pOP-CNH05081_EST_C_1_pSK_SK	574
cl0387	ct0407	cn0418	pOP-CNH01244_EST_C_1_pSK_SK	427
cl0387	ct0407	cn0418	pOP-CNH02869_EST_C_1_pSK_SK	430
cl0387	ct0407	cn0418	pOP-CNH05025_EST_C_1_pSK_SK	636
cl0387	ct0407	cn0418	pOP-CNH05084_EST_C_1_pSK_SK	694
cl0388	ct0408	cn0419	pOP-CNH02180_EST_C_1_pSK_SK	495
cl0388	ct0408	cn0419	pOP-CNH03510_EST_C_1_pSK_SK	316
cl0388	ct0408	cn0419	pOP-CNH04199	527
cl0388	ct0408	cn0419	pOP-CNH04328	493
cl0388	ct0408	cn0419	pOP-CNHP00025_EST_C_1_pSK_SK	468
cl0389	ct0409	cn0420	pOP-CNH01356_EST_C_1_pSK_SK	389
cl0389	ct0409	cn0420	pOP-CNH03625_EST_C_1_pSK_SK	649
cl0389	ct0409	cn0420	pOP-CNHP00028_EST_C_1_pSK_SK	486
cl0390	ct0410	cn0421	pOP-CNH01625_EST_C_1_pSK_SK	671
cl0390	ct0410	cn0421	pOP-CNH01632_EST_C_1_pSK_SK	658
cl0390	ct0410	cn0421	pOP-CNH02296_EST_C_1_pSK_SK	621
cl0390	ct0410	cn0422	pOP-CNHP00033_EST_C_1_pSK_SK	645
cl0391	ct0411	cn0423	pOP-CNH00839_EST_C_1_pSK_SK	520
cl0391	ct0411	cn0423	pOP-CNHP00036_EST_C_1_pSK_SK	597
cl0391	ct0411	cn0423	pOP-EO02685_EST_C_1_pSK_SK	268

cl0392	ct0412	cn0424	pOP-CNH03042_EST_C_1_pSK_SK	525
cl0392	ct0412	cn0424	pOP-CNHP00048_EST_C_1_pSK_SK	635
cl0393	ct0413	cn0425	pOP-CNH00954_EST_C_1_pSK_SK	600
cl0393	ct0413	cn0425	pOP-CNHP00050_EST_C_1_pSK_SK	619
cl0394	ct0414	cn0426	pOP-CNH03811_EST_C_1_pSK_SK	377
cl0394	ct0414	cn0426	pOP-CNHP00064_EST_C_1_pSK_SK	517
cl0395	ct0415	cn0427	pOP-CNH00934_EST_C_1_pSK_SK	408
cl0395	ct0415	cn0427	pOP-CNH01802_EST_C_1_pSK_SK	509
cl0395	ct0415	cn0427	pOP-CNH02771_EST_C_1_pSK_SK	592
cl0395	ct0415	cn0427	pOP-CNH03066_EST_C_1_pSK_SK	660
cl0395	ct0415	cn0427	pOP-CNHP00067_EST_C_1_pSK_SK	571
cl0396	ct0416	cn0428	pOP-CNH01188_EST_C_1_pSK_SK	587
cl0396	ct0416	cn0428	pOP-CNHP00080_EST_C_1_pSK_SK	673
cl0397	ct0417	cn0429	pOP-CNHP00087_EST_C_1_pSK_SK	632
cl0397	ct0418	cn0430	pOP-CNH03503_EST_C_1_pSK_SK	563
cl0398	ct0419	cn0431	pOP-CNH00772_EST_C_1_pSK_SK	432
cl0398	ct0419	cn0431	pOP-CNH00854_EST_C_1_pSK_SK	532
cl0398	ct0419	cn0431	pOP-CNHP00089_EST_C_1_pSK_SK	373
cl0399	ct0420	cn0432	pOP-CNH00658_EST_C_1_pSK_SK	621
cl0399	ct0420	cn0432	pOP-CNHP00094_EST_C_1_pSK_SK	273
cl0400	ct0421	cn0433	pOP-CNH01849_EST_C_1_pSK_SK	512
cl0400	ct0421	cn0433	pOP-CNH02289_EST_C_1_pSK_SK	671
cl0400	ct0421	cn0433	pOP-CNH03025_EST_C_1_pSK_SK	460
cl0400	ct0421	cn0433	pOP-CNHP00102_EST_C_1_pSK_SK	568
cl0401	ct0422	cn0434	pOP-CNH01534_EST_C_1_pSK_SK	593
cl0401	ct0422	cn0434	pOP-CNH01567_EST_C_1_pSK_SK	594
cl0401	ct0422	cn0434	pOP-CNH04676	814
cl0401	ct0422	cn0434	pOP-CNHP00109_EST_C_1_pSK_SK	526
cl0402	ct0423	cn0435	pOP-CNH00616_EST_C_1_pSK_SK	727
cl0402	ct0423	cn0435	pOP-CNHP00125_EST_C_1_pSK_SK	647
cl0403	ct0424	cn0436	pOP-CNH00846_EST_C_1_pSK_SK	628
cl0403	ct0424	cn0436	pOP-CNHP00135_EST_C_1_pSK_SK	516
cl0404	ct0425	cn0437	pOP-CNH02306_EST_C_1_pSK_SK	478
cl0404	ct0425	cn0437	pOP-CNHP00140_EST_C_1_pSK_SK	499
cl0405	ct0426	cn0438	pOP-CNH00869_EST_C_1_pSK_SK	684
cl0405	ct0426	cn0438	pOP-CNH03800_EST_C_1_pSK_SK	474
cl0405	ct0426	cn0438	pOP-CNHP00141_EST_C_1_pSK_SK	647
cl0406	ct0427	cn0439	pOP-CNH00649_EST_C_1_pSK_SK	512
cl0406	ct0427	cn0439	pOP-CNH03640_EST_C_1_pSK_SK	628
cl0406	ct0427	cn0439	pOP-CNHP00145_EST_C_1_pSK_SK	617
cl0407	ct0428	cn0440	pOP-CNH01154_EST_C_1_pSK_SK	408
cl0407	ct0428	cn0440	pOP-CNHP00155_EST_C_1_pSK_SK	329
cl0408	ct0429	cn0441	pOP-CNH01327_EST_C_1_pSK_SK	484
cl0408	ct0429	cn0441	pOP-CNHP00159_EST_C_1_pSK_SK	456
cl0409	ct0430	cn0442	pOP-CNH04432	575
cl0409	ct0430	cn0442	pOP-CNHP00175_EST_C_1_pSK_SK	499
cl0410	ct0431	cn0443	pOP-CNH01615_EST_C_1_pSK_SK	411
cl0410	ct0431	cn0443	pOP-CNHP00209_EST_C_1_pSK_SK	475
cl0411	ct0432	cn0444	pOP-CNH01142_EST_C_1_pSK_SK	418
cl0411	ct0432	cn0444	pOP-CNH01775_EST_C_1_pSK_SK	549
cl0411	ct0432	cn0444	pOP-CNHP00216_EST_C_1_pSK_SK	450
cl0412	ct0433	cn0445	pOP-CNH00809_EST_C_1_pSK_SK	569
cl0412	ct0434	cn0446	pOP-CNH04545	791
cl0412	ct0434	cn0446	pOP-CNHP00217_EST_C_1_pSK_SK	523
cl0413	ct0435	cn0447	pOP-CNH04982_EST_C_1_pSK_SK	693
cl0413	ct0435	cn0447	pOP-CNHP00218_EST_C_1_pSK_SK	444

cl0414	ct0436	cn0448	pOP-CNH03578_EST_C_1_pSK_SK	359
cl0414	ct0436	cn0448	pOP-CNHP00245_EST_C_1_pSK_SK	373
cl0415	ct0437	cn0449	pOP-CNH03402_EST_C_1_pSK_SK	567
cl0415	ct0437	cn0449	pOP-CNH04299	480
cl0415	ct0437	cn0449	pOP-CNH04430	767
cl0415	ct0437	cn0449	pOP-CNHP00253_EST_C_1_pSK_SK	512
cl0416	ct0438	cn0450	pOP-CNH00760_EST_C_1_pSK_SK	531
cl0416	ct0438	cn0450	pOP-CNH01211_EST_C_1_pSK_SK	620
cl0416	ct0438	cn0450	pOP-CNH01457_EST_C_1_pSK_SK	556
cl0416	ct0438	cn0450	pOP-CNH03793_EST_C_1_pSK_SK	611
cl0416	ct0438	cn0450	pOP-CNHP00256_EST_C_1_pSK_SK	503
cl0416	ct0438	cn0450	pOP-EO05658_EST_C_1_pSK_SK	485
cl0417	ct0439	cn0451	pOP-CNH00817_EST_C_1_pSK_SK	486
cl0417	ct0439	cn0451	pOP-CNHP00266_EST_C_1_pSK_SK	236
cl0418	ct0440	cn0452	pOP-CNH02854_EST_C_1_pSK_SK	536
cl0418	ct0440	cn0452	pOP-CNHP00273_EST_C_1_pSK_SK	513
cl0419	ct0441	cn0453	pOP-CNH00791_EST_C_1_pSK_SK	450
cl0419	ct0441	cn0453	pOP-CNHP00280_EST_C_1_pSK_SK	556
cl0420	ct0442	cn0454	pOP-CNH01641_EST_C_1_pSK_SK	688
cl0420	ct0442	cn0454	pOP-CNHP00310_EST_C_1_pSK_SK	623
cl0420	ct0442	cn0454	pOP-CNHP00312_EST_C_1_pSK_SK	749
cl0421	ct0443	cn0455	pOP-CNH01050_EST_C_1_pSK_SK	535
cl0421	ct0443	cn0455	pOP-CNHP00318_EST_C_1_pSK_SK	540
cl0422	ct0444	cn0456	pOP-CNH03513_EST_C_1_pSK_SK	602
cl0422	ct0444	cn0456	pOP-CNH04171	530
cl0422	ct0444	cn0456	pOP-CNHP00319_EST_C_1_pSK_SK	535
cl0423	ct0445	cn0457	pOP-CNH05007_EST_C_1_pSK_SK	762
cl0423	ct0445	cn0457	pOP-CNHP00320_EST_C_1_pSK_SK	486
cl0424	ct0446	cn0458	pOP-CNH03050_EST_C_1_pSK_SK	569
cl0424	ct0446	cn0458	pOP-CNHP00321_EST_C_1_pSK_SK	719
cl0425	ct0447	cn0459	pOP-CNHP00199_EST_C_1_pSK_SK	183
cl0425	ct0447	cn0459	pOP-CNHP00327_EST_C_1_pSK_SK	700
cl0426	ct0448	cn0460	pOP-CNH01282_EST_C_1_pSK_SK	638
cl0426	ct0448	cn0460	pOP-CNH01657_EST_C_1_pSK_SK	621
cl0426	ct0448	cn0460	pOP-CNH01932_EST_C_1_pSK_SK	673
cl0426	ct0448	cn0460	pOP-CNH03580_EST_C_1_pSK_SK	350
cl0426	ct0448	cn0460	pOP-CNHP00287_EST_C_1_pSK_SK	253
cl0426	ct0448	cn0460	pOP-CNHP00329_EST_C_1_pSK_SK	596
cl0427	ct0449	cn0461	pOP-CNH03596_EST_C_1_pSK_SK	348
cl0427	ct0449	cn0461	pOP-CNHP00331_EST_C_1_pSK_SK	649
cl0428	ct0450	cn0462	pOP-CNH02215_EST_C_1_pSK_SK	770
cl0428	ct0450	cn0462	pOP-CNH04482	754
cl0428	ct0450	cn0462	pOP-CNH04916_EST_C_1_pSK_SK	604
cl0428	ct0450	cn0462	pOP-CNHP00332_EST_C_1_pSK_SK	447
cl0429	ct0451	cn0463	pOP-CNH01785_EST_C_1_pSK_SK	483
cl0429	ct0451	cn0463	pOP-CNHP00333_EST_C_1_pSK_SK	638
cl0430	ct0452	cn0464	pOP-CNH03430_EST_C_1_pSK_SK	512
cl0430	ct0452	cn0464	pOP-CNHP00335_EST_C_1_pSK_SK	685
cl0431	ct0453	cn0465	pOP-CNH01045_EST_C_1_pSK_SK	524
cl0431	ct0453	cn0465	pOP-CNH01559_EST_C_1_pSK_SK	670
cl0431	ct0453	cn0465	pOP-CNH01913_EST_C_1_pSK_SK	531
cl0431	ct0453	cn0465	pOP-CNHP00345_EST_C_1_pSK_SK	696
cl0432	ct0454	cn0466	pOP-CNH01127_EST_C_1_pSK_SK	443
cl0432	ct0454	cn0466	pOP-CNH04293	450
cl0432	ct0454	cn0466	pOP-CNHP00346_EST_C_1_pSK_SK	625
cl0433	ct0455	cn0467	pOP-CNH01806_EST_C_1_pSK_SK	615

cl0433	ct0455	cn0468	pOP-CNH01263_EST_C_1_pSK_SK	516
cl0433	ct0455	cn0468	pOP-CNH02300_EST_C_1_pSK_SK	534
cl0433	ct0455	cn0468	pOP-CNHP00354_EST_C_1_pSK_SK	456
cl0434	ct0456	cn0469	pOP-CNH01483_EST_C_1_pSK_SK	604
cl0434	ct0456	cn0469	pOP-CNH01779_EST_C_1_pSK_SK	491
cl0434	ct0456	cn0469	pOP-CNH01916_EST_C_1_pSK_SK	712
cl0434	ct0456	cn0469	pOP-CNH03220_EST_C_1_pSK_SK	437
cl0434	ct0456	cn0469	pOP-CNHP00370_EST_C_1_pSK_SK	659
cl0435	ct0457	cn0470	pOP-CNH00699_EST_C_1_pSK_SK	654
cl0435	ct0457	cn0470	pOP-CNH00812_EST_C_1_pSK_SK	629
cl0435	ct0457	cn0470	pOP-CNH00816_EST_C_1_pSK_SK	550
cl0435	ct0457	cn0470	pOP-CNH01487_EST_C_1_pSK_SK	541
cl0435	ct0457	cn0470	pOP-CNH01607_EST_C_1_pSK_SK	523
cl0435	ct0457	cn0470	pOP-CNH01929_EST_C_1_pSK_SK	537
cl0435	ct0457	cn0470	pOP-CNH02286_EST_C_1_pSK_SK	665
cl0435	ct0457	cn0470	pOP-CNH03212_EST_C_1_pSK_SK	512
cl0435	ct0457	cn0470	pOP-CNH04395	641
cl0435	ct0457	cn0470	pOP-CNH04733_EST_C_1_pSK_SK	484
cl0435	ct0457	cn0470	pOP-CNHP00066_EST_C_1_pSK_SK	609
cl0435	ct0457	cn0470	pOP-CNHP00380_EST_C_1_pSK_SK	445
cl0435	ct0457	cn0470	pOP-CNHP00530_EST_C_1_pSK_SK	568
cl0435	ct0457	cn0470	pOP-CNI02251_EST_C_1_pSK_SK	735
cl0435	ct0457	cn0470	pOP-CNIP01054_EST_C_1_pSK_SK	218
cl0436	ct0458	cn0471	pOP-CNH01077_EST_C_1_pSK_SK	574
cl0436	ct0458	cn0471	pOP-CNHP00396_EST_C_1_pSK_SK	626
cl0437	ct0459	cn0472	pOP-CNH02392_EST_C_1_pSK_SK	278
cl0437	ct0459	cn0472	pOP-CNHP00402_EST_C_1_pSK_SK	278
cl0438	ct0460	cn0473	pOP-CNH02741_EST_C_1_pSK_SK	570
cl0438	ct0460	cn0473	pOP-CNH03539_EST_C_1_pSK_SK	570
cl0438	ct0460	cn0473	pOP-CNH04736_EST_C_1_pSK_SK	526
cl0438	ct0460	cn0473	pOP-CNHP00409_EST_C_1_pSK_SK	584
cl0439	ct0461	cn0474	pOP-CNH02472_EST_C_1_pSK_SK	398
cl0439	ct0461	cn0474	pOP-CNHP00416_EST_C_1_pSK_SK	718
cl0440	ct0462	cn0475	pOP-CNH01529_EST_C_1_pSK_SK	602
cl0440	ct0462	cn0475	pOP-CNH04659	798
cl0440	ct0462	cn0475	pOP-CNHP00337_EST_C_1_pSK_SK	202
cl0440	ct0462	cn0475	pOP-CNHP00422_EST_C_1_pSK_SK	708
cl0441	ct0463	cn0476	pOP-CNH04635	521
cl0441	ct0463	cn0476	pOP-CNHP00313_EST_C_1_pSK_SK	505
cl0441	ct0463	cn0476	pOP-CNHP00428_EST_C_1_pSK_SK	522
cl0442	ct0464	cn0477	pOP-CNH04178	525
cl0442	ct0464	cn0477	pOP-CNH04787_EST_C_1_pSK_SK	578
cl0442	ct0464	cn0477	pOP-CNHP00434_EST_C_1_pSK_SK	780
cl0443	ct0465	cn0478	pOP-CNH02290_EST_C_1_pSK_SK	530
cl0443	ct0465	cn0478	pOP-CNHP00446_EST_C_1_pSK_SK	606
cl0444	ct0466	cn0479	pOP-CNHP00451_EST_C_1_pSK_SK	473
cl0444	ct0466	cn0479	pOP-EO04378_EST_C_1_pSK_SK	349
cl0445	ct0467	cn0480	pOP-CNH00590_EST_C_1_pSK_SK	646
cl0445	ct0467	cn0480	pOP-CNH01713_EST_C_1_pSK_SK	526
cl0445	ct0467	cn0480	pOP-CNH03518_EST_C_1_pSK_SK	625
cl0445	ct0467	cn0480	pOP-CNHP00457_EST_C_1_pSK_SK	736
cl0446	ct0468	cn0481	pOP-CNH01024_EST_C_1_pSK_SK	319
cl0446	ct0468	cn0481	pOP-CNHP00445_EST_C_1_pSK_SK	685
cl0446	ct0468	cn0481	pOP-CNHP00458_EST_C_1_pSK_SK	678
cl0446	ct0468	cn0481	pOP-EO05798_EST_C_1_pSK_SK	629
cl0447	ct0469	cn0482	pOP-CNH01908_EST_C_1_pSK_SK	595

cl0447	ct0469	cn0482	pOP-CNH03307_EST_C_1_pSK_SK	581
cl0447	ct0469	cn0482	pOP-CNH03755_EST_C_1_pSK_SK	520
cl0447	ct0469	cn0482	pOP-CNHP00461_EST_C_1_pSK_SK	671
cl0448	ct0470	cn0483	pOP-CNH01332_EST_C_1_pSK_SK	663
cl0448	ct0470	cn0483	pOP-CNH03322_EST_C_1_pSK_SK	557
cl0448	ct0470	cn0483	pOP-CNHP00462_EST_C_1_pSK_SK	688
cl0449	ct0471	cn0484	pOP-CNH02197_EST_C_1_pSK_SK	643
cl0449	ct0471	cn0484	pOP-CNHP00465_EST_C_1_pSK_SK	747
cl0450	ct0472	cn0485	pOP-CNHP00132_EST_C_1_pSK_SK	596
cl0450	ct0472	cn0485	pOP-CNHP00299_EST_C_1_pSK_SK	614
cl0450	ct0472	cn0485	pOP-CNHP00470_EST_C_1_pSK_SK	636
cl0451	ct0473	cn0486	pOP-CNH00566_EST_C_1_pSK_SK	750
cl0451	ct0473	cn0486	pOP-CNH03361_EST_C_1_pSK_SK	658
cl0451	ct0473	cn0486	pOP-CNH04282	568
cl0451	ct0473	cn0486	pOP-CNHP00478_EST_C_1_pSK_SK	596
cl0452	ct0474	cn0487	pOP-CNH03673_EST_C_1_pSK_SK	404
cl0452	ct0474	cn0487	pOP-CNHP00484_EST_C_1_pSK_SK	595
cl0453	ct0475	cn0488	pOP-CNH04904_EST_C_1_pSK_SK	693
cl0453	ct0475	cn0488	pOP-CNHP00493_EST_C_1_pSK_SK	608
cl0454	ct0476	cn0489	pOP-CNH01110_EST_C_1_pSK_SK	265
cl0454	ct0476	cn0489	pOP-CNH01195_EST_C_1_pSK_SK	590
cl0454	ct0476	cn0489	pOP-CNH03067_EST_C_1_pSK_SK	597
cl0454	ct0476	cn0489	pOP-CNHP00494_EST_C_1_pSK_SK	605
cl0455	ct0477	cn0490	pOP-CNH01202_EST_C_1_pSK_SK	569
cl0455	ct0477	cn0490	pOP-CNH02055_EST_C_1_pSK_SK	566
cl0455	ct0477	cn0490	pOP-CNH02596_EST_C_1_pSK_SK	566
cl0455	ct0477	cn0490	pOP-CNHP00514_EST_C_1_pSK_SK	569
cl0456	ct0478	cn0491	pOP-CNH01018_EST_C_1_pSK_SK	264
cl0456	ct0478	cn0491	pOP-CNH01349_EST_C_1_pSK_SK	663
cl0456	ct0478	cn0491	pOP-CNH02369_EST_C_1_pSK_SK	445
cl0456	ct0478	cn0491	pOP-CNH03188_EST_C_1_pSK_SK	529
cl0456	ct0478	cn0491	pOP-CNH05013_EST_C_1_pSK_SK	712
cl0456	ct0478	cn0491	pOP-CNHP00103_EST_C_1_pSK_SK	600
cl0456	ct0478	cn0491	pOP-CNHP00526_EST_C_1_pSK_SK	575
cl0456	ct0478	cn0492	pOP-CNHP00228_EST_C_1_pSK_SK	579
cl0457	ct0479	cn0493	pOP-CNH00731_EST_C_1_pSK_SK	599
cl0457	ct0479	cn0493	pOP-CNH02489_EST_C_1_pSK_SK	430
cl0457	ct0479	cn0493	pOP-CNH02493_EST_C_1_pSK_SK	468
cl0457	ct0479	cn0493	pOP-CNHP00092_EST_C_1_pSK_SK	573
cl0457	ct0479	cn0493	pOP-CNHP00535_EST_C_1_pSK_SK	566
cl0458	ct0480	cn0494	pOP-CNH03591_EST_C_1_pSK_SK	503
cl0458	ct0480	cn0494	pOP-CNHP00537_EST_C_1_pSK_SK	578
cl0459	ct0481	cn0495	pOP-CNH03015_EST_C_1_pSK_SK	489
cl0459	ct0481	cn0495	pOP-CNI01089_EST_C_1_pSK_SK	188
cl0460	ct0482	cn0496	pOP-CNH01426_EST_C_1_pSK_SK	465
cl0460	ct0482	cn0496	pOP-CNH03299_EST_C_1_pSK_SK	469
cl0460	ct0482	cn0496	pOP-CNI01123_EST_C_1_pSK_SK	231
cl0460	ct0482	cn0496	pOP-CNI01124_EST_C_1_pSK_SK	231
cl0461	ct0483	cn0497	pOP-CNI01142_EST_C_1_pSK_SK	368
cl0461	ct0483	cn0497	pOP-EAP00696_EST_C_1_pBSK_SK	174
cl0462	ct0484	cn0498	pOP-CNI01145_EST_C_1_pSK_SK	499
cl0462	ct0484	cn0499	pOP-EO03743_EST_C_1_pSK_SK	498
cl0463	ct0485	cn0500	pOP-CNI01155_EST_C_1_pSK_SK	404
cl0463	ct0485	cn0500	pOP-EO02830_EST_C_1_pSK_SK	411
cl0464	ct0486	cn0501	pOP-CNH04136	470
cl0464	ct0486	cn0501	pOP-CNI01178_EST_C_1_pSK_SK	505

cl0465	ct0487	cn0502	pOP-CNI01188_EST_C_1_pSK_SK	216
cl0465	ct0487	cn0502	pOP-CNI01189_EST_C_1_pSK_SK	231
cl0466	ct0488	cn0503	pOP-CNH00645_EST_C_1_pSK_SK	515
cl0466	ct0488	cn0503	pOP-CNI01190_EST_C_1_pSK_SK	435
cl0467	ct0489	cn0504	pOP-CNH02551_EST_C_1_pSK_SK	542
cl0467	ct0489	cn0504	pOP-CNI01204_EST_C_1_pSK_SK	209
cl0468	ct0490	cn0505	pOP-CNH02364_EST_C_1_pSK_SK	658
cl0468	ct0490	cn0505	pOP-CNI01208_EST_C_1_pSK_SK	508
cl0469	ct0491	cn0506	pOP-CNI01135_EST_C_1_pSK_SK	256
cl0469	ct0491	cn0506	pOP-CNI01136_EST_C_1_pSK_SK	226
cl0469	ct0491	cn0506	pOP-CNI01214_EST_C_1_pSK_SK	226
cl0469	ct0491	cn0506	pOP-EO08139_EST_C_1_pSK_SK	486
cl0470	ct0492	cn0507	pOP-CEO00874_EST_C_1_pSK_SK	595
cl0470	ct0492	cn0507	pOP-CNI01237_EST_C_1_pSK_SK	602
cl0471	ct0493	cn0508	pOP-CEO03470_EST_C_1_pSK_SK	339
cl0471	ct0493	cn0508	pOP-CNI01269_EST_C_1_pSK_SK	682
cl0472	ct0494	cn0509	pOP-CNI01285_EST_C_1_pSK_SK	503
cl0472	ct0494	cn0509	pOP-CNI01759_EST_C_1_pSK_SK	609
cl0473	ct0495	cn0510	pOP-CNHP00023_EST_C_1_pSK_SK	360
cl0473	ct0495	cn0510	pOP-CNI01286_EST_C_1_pSK_SK	283
cl0473	ct0496	cn0511	pOP-CNH00963_EST_C_1_pSK_SK	651
cl0473	ct0496	cn0511	pOP-CNH04707	774
cl0473	ct0496	cn0511	pOP-CNH04777_EST_C_1_pSK_SK	581
cl0473	ct0496	cn0511	pOP-CNH04870_EST_C_1_pSK_SK	471
cl0473	ct0496	cn0511	pOP-CNI01702_EST_C_1_pSK_SK	495
cl0473	ct0496	cn0511	pOP-CNIP00407_EST_C_1_pSK_SK	619
cl0473	ct0496	cn0511	pOP-EO02977_EST_C_1_pSK_SK	374
cl0473	ct0496	cn0511	pOP-EO04699_EST_C_1_pSK_SK	521
cl0474	ct0497	cn0512	pOP-CNI01310_EST_C_1_pSK_SK	279
cl0474	ct0497	cn0512	pOP-CNI01311_EST_C_1_pSK_SK	279
cl0475	ct0498	cn0513	pOP-CNH04268	574
cl0475	ct0498	cn0513	pOP-CNI01314_EST_C_1_pSK_SK	435
cl0476	ct0499	cn0514	pOP-CNI01316_EST_C_1_pSK_SK	498
cl0476	ct0499	cn0514	pOP-EO08143_EST_C_1_pSK_SK	550
cl0477	ct0500	cn0515	pOP-CEO03623_EST_C_1_pSK_SK	535
cl0477	ct0500	cn0516	pOP-CNI01330_EST_C_1_pSK_SK	419
cl0478	ct0501	cn0517	pOP-CNH02149_EST_C_1_pSK_SK	437
cl0478	ct0501	cn0517	pOP-CNH02179_EST_C_1_pSK_SK	431
cl0478	ct0501	cn0517	pOP-CNH04406	830
cl0478	ct0501	cn0517	pOP-CNH04798_EST_C_1_pSK_SK	538
cl0478	ct0501	cn0517	pOP-CNI01392_EST_C_1_pSK_SK	542
cl0478	ct0501	cn0517	pOP-EO07482_EST_C_1_pSK_SK	764
cl0478	ct0501	cn0517	pOP-EO07489_EST_C_1_pSK_SK	717
cl0479	ct0502	cn0518	pOP-CNH03350_EST_C_1_pSK_SK	403
cl0479	ct0502	cn0518	pOP-CNI01395_EST_C_1_pSK_SK	519
cl0480	ct0503	cn0519	pOP-CBP00158_EST_C_1_pBSK_SK	591
cl0480	ct0503	cn0519	pOP-CNH01506_EST_C_1_pSK_SK	468
cl0480	ct0503	cn0519	pOP-CNH02631_EST_C_1_pSK_SK	633
cl0480	ct0503	cn0519	pOP-CNH02692_EST_C_1_pSK_SK	529
cl0480	ct0503	cn0519	pOP-CNH04190	557
cl0480	ct0503	cn0519	pOP-CNHP00044_EST_C_1_pSK_SK	630
cl0480	ct0503	cn0519	pOP-CNHP00241_EST_C_1_pSK_SK	528
cl0480	ct0503	cn0519	pOP-CNHP00414_EST_C_1_pSK_SK	640
cl0480	ct0503	cn0519	pOP-CNI01398_EST_C_1_pSK_SK	414
cl0480	ct0503	cn0519	pOP-CNI01399_EST_C_1_pSK_SK	411
cl0481	ct0504	cn0520	pOP-CNI01298_EST_C_1_pSK_SK	579

cl0481	ct0504	cn0520	pOP-CNI01522_EST_C_1_pSK_SK	483
cl0482	ct0505	cn0521	pOP-CBP00241_EST_C_1_pBSK_SK	569
cl0482	ct0505	cn0521	pOP-CNHP00203_EST_C_1_pSK_SK	510
cl0482	ct0505	cn0521	pOP-CNI01490_EST_C_1_pSK_SK	616
cl0482	ct0505	cn0521	pOP-CNI01588_EST_C_1_pSK_SK	230
cl0482	ct0505	cn0521	pOP-EAP01511_EST_C_1_pBSK_SK	609
cl0482	ct0505	cn0521	pOP-EAP05017_EST_C_1_pBSK_SK	425
cl0482	ct0505	cn0521	pOP-EO03514_EST_C_1_pSK_SK	538
cl0482	ct0505	cn0521	pOP-EO07322_EST_C_1_pSK_SK	765
cl0483	ct0506	cn0522	pOP-CNH00799_EST_C_1_pSK_SK	518
cl0483	ct0507	cn0523	pOP-CNH02410_EST_C_1_pSK_SK	526
cl0483	ct0507	cn0523	pOP-CNHP00061_EST_C_1_pSK_SK	602
cl0483	ct0507	cn0523	pOP-CNHP00243_EST_C_1_pSK_SK	459
cl0483	ct0508	cn0524	pOP-CEO02500_EST_C_1_pSK_SK	467
cl0483	ct0508	cn0524	pOP-CNHP00497_EST_C_1_pSK_SK	591
cl0483	ct0508	cn0524	pOP-CNI01603_EST_C_1_pSK_SK	201
cl0483	ct0508	cn0524	pOP-EAP02767_EST_C_1_pBSK_SK	656
cl0483	ct0508	cn0524	pOP-EO04936_EST_C_1_pSK_SK	520
cl0483	ct0508	cn0524	pOP-EO06691_EST_C_1_pSK_SK	530
cl0483	ct0508	cn0524	pOP-EO06732_EST_C_1_pSK_SK	623
cl0484	ct0509	cn0525	pOP-CNI01623_EST_C_1_pSK_SK	188
cl0484	ct0509	cn0525	pOP-EO03179_EST_C_1_pSK_SK	434
cl0485	ct0510	cn0526	pOP-CNI01628_EST_C_1_pSK_SK	272
cl0485	ct0510	cn0526	pOP-CNIP00232_EST_C_1_pSK_SK	185
cl0486	ct0511	cn0527	pOP-CNHP00517_EST_C_1_pSK_SK	575
cl0486	ct0511	cn0527	pOP-CNI01648_EST_C_1_pSK_SK	744
cl0487	ct0512	cn0528	pOP-CNI01653_EST_C_1_pSK_SK	722
cl0487	ct0512	cn0528	pOP-EO03197_EST_C_1_pSK_SK	360
cl0488	ct0513	cn0529	pOP-CNH03796_EST_C_1_pSK_SK	558
cl0488	ct0513	cn0529	pOP-CNH04544	733
cl0488	ct0513	cn0529	pOP-CNI01654_EST_C_1_pSK_SK	498
cl0489	ct0514	cn0530	pOP-CEO02447_EST_C_1_pSK_SK	185
cl0489	ct0514	cn0530	pOP-CNI01657_EST_C_1_pSK_SK	515
cl0490	ct0515	cn0531	pOP-CNH04894_EST_C_1_pSK_SK	732
cl0490	ct0515	cn0531	pOP-CNI01712_EST_C_1_pSK_SK	528
cl0491	ct0516	cn0532	pOP-CNHP00040_EST_C_1_pSK_SK	562
cl0491	ct0516	cn0532	pOP-CNI01716_EST_C_1_pSK_SK	483
cl0492	ct0517	cn0533	pOP-CNH02073_EST_C_1_pSK_SK	466
cl0492	ct0517	cn0533	pOP-CNI01734_EST_C_1_pSK_SK	497
cl0493	ct0518	cn0534	pOP-CNH01056_EST_C_1_pSK_SK	553
cl0493	ct0518	cn0534	pOP-CNH01656_EST_C_1_pSK_SK	678
cl0493	ct0518	cn0534	pOP-CNHP00432_EST_C_1_pSK_SK	739
cl0493	ct0518	cn0534	pOP-CNHP00466_EST_C_1_pSK_SK	523
cl0493	ct0518	cn0534	pOP-CNHP00536_EST_C_1_pSK_SK	622
cl0493	ct0518	cn0535	pOP-CNI01747_EST_C_1_pSK_SK	379
cl0494	ct0519	cn0536	pOP-CNI01763_EST_C_1_pSK_SK	524
cl0494	ct0519	cn0536	pOP-EO04300_EST_C_1_pSK_SK	334
cl0495	ct0520	cn0537	pOP-CEM00211_EST_C_1_pSK_SK	174
cl0495	ct0520	cn0537	pOP-CNI01776_EST_C_1_pSK_SK	446
cl0495	ct0520	cn0537	pOP-CNIP00970_EST_C_1_pSK_SK	380
cl0496	ct0521	cn0538	pOP-CNH02061_EST_C_1_pSK_SK	535
cl0496	ct0521	cn0538	pOP-CNH02992_EST_C_1_pSK_SK	563
cl0496	ct0521	cn0538	pOP-CNI01797_EST_C_1_pSK_SK	548
cl0497	ct0522	cn0539	pOP-CNI01641_EST_C_1_pSK_SK	428
cl0497	ct0522	cn0539	pOP-CNI01802_EST_C_1_pSK_SK	416
cl0498	ct0523	cn0540	pOP-CEO01418_EST_C_1_pSK_SK	342

cl0498	ct0523	cn0540	pOP-CNI01806_EST_C_1_pSK_SK	554
cl0499	ct0524	cn0541	pOP-CNH00600_EST_C_1_pSK_SK	592
cl0499	ct0524	cn0541	pOP-CNH02402_EST_C_1_pSK_SK	469
cl0499	ct0524	cn0541	pOP-CNH02504_EST_C_1_pSK_SK	449
cl0499	ct0524	cn0541	pOP-CNH02524_EST_C_1_pSK_SK	406
cl0499	ct0524	cn0541	pOP-CNH02618_EST_C_1_pSK_SK	339
cl0499	ct0524	cn0541	pOP-CNH04886_EST_C_1_pSK_SK	822
cl0499	ct0524	cn0541	pOP-CNI01822_EST_C_1_pSK_SK	343
cl0499	ct0524	cn0541	pOP-CNI01893_EST_C_1_pSK_SK	486
cl0499	ct0524	cn0541	pOP-CNIP01039_EST_C_1_pSK_SK	786
cl0499	ct0524	cn0541	pOP-CNIP01040_EST_C_1_pSK_SK	625
cl0499	ct0524	cn0541	pOP-EAP00686_EST_C_1_pBSK_SK	219
cl0499	ct0524	cn0541	pOP-EO03932_EST_C_1_pSK_SK	528
cl0499	ct0524	cn0541	pOP-EO04060_EST_C_1_pSK_SK	334
cl0499	ct0524	cn0541	pOP-EO04129_EST_C_1_pSK_SK	504
cl0499	ct0524	cn0541	pOP-EO06214_EST_C_1_pSK_SK	519
cl0499	ct0524	cn0541	pOP-EO06340_EST_C_1_pSK_SK	597
cl0499	ct0524	cn0541	pOP-EO07838_EST_C_1_pSK_SK	751
cl0499	ct0524	cn0542	pOP-CEO01581_EST_C_1_pSK_SK	458
cl0500	ct0525	cn0543	pOP-CNI01826_EST_C_1_pSK_SK	385
cl0500	ct0525	cn0543	pOP-CNIP00084_EST_C_1_pSK_SK	251
cl0500	ct0526	cn0544	pOP-CAP00230_EST_C_1_pBSK_SK	616
cl0500	ct0526	cn0544	pOP-CBP00167_EST_C_1_pBSK_SK	426
cl0500	ct0526	cn0544	pOP-CNI01952_EST_C_1_pSK_SK	520
cl0500	ct0526	cn0544	pOP-CNI01959_EST_C_1_pSK_SK	258
cl0500	ct0526	cn0544	pOP-CNIP00478_EST_C_1_pSK_SK	179
cl0500	ct0526	cn0544	pOP-CNIP00823_EST_C_1_pSK_SK	244
cl0500	ct0526	cn0544	pOP-CNIP01030_EST_C_1_pSK_SK	308
cl0501	ct0527	cn0545	pOP-CNI01498_EST_C_1_pSK_SK	485
cl0501	ct0527	cn0545	pOP-CNI01828_EST_C_1_pSK_SK	472
cl0502	ct0528	cn0546	pOP-CNH03754_EST_C_1_pSK_SK	500
cl0502	ct0528	cn0546	pOP-CNHP00146_EST_C_1_pSK_SK	505
cl0502	ct0528	cn0546	pOP-EO06637_EST_C_1_pSK_SK	656
cl0502	ct0528	cn0546	pOP-EO06786_EST_C_1_pSK_SK	786
cl0502	ct0528	cn0547	pOP-CNI01851_EST_C_1_pSK_SK	520
cl0503	ct0529	cn0548	pOP-CNH00711_EST_C_1_pSK_SK	499
cl0503	ct0529	cn0548	pOP-CNHP00467_EST_C_1_pSK_SK	725
cl0503	ct0529	cn0548	pOP-CNI01861_EST_C_1_pSK_SK	713
cl0503	ct0530	cn0549	pOP-CNH02269_EST_C_1_pSK_SK	674
cl0503	ct0530	cn0549	pOP-CNH04653	674
cl0503	ct0530	cn0549	pOP-CNHP00170_EST_C_1_pSK_SK	311
cl0503	ct0530	cn0549	pOP-CNI01426_EST_C_1_pSK_SK	313
cl0504	ct0531	cn0550	pOP-CNH04424	571
cl0504	ct0531	cn0550	pOP-CNI01253_EST_C_1_pSK_SK	321
cl0504	ct0531	cn0550	pOP-CNI01862_EST_C_1_pSK_SK	208
cl0505	ct0532	cn0551	pOP-CEO01501_EST_C_1_pSK_SK	652
cl0505	ct0532	cn0551	pOP-CNI01878_EST_C_1_pSK_SK	368
cl0506	ct0533	cn0552	pOP-CNH00685_EST_C_1_pSK_SK	421
cl0506	ct0533	cn0552	pOP-CNH02377_EST_C_1_pSK_SK	313
cl0506	ct0533	cn0552	pOP-CNH02412_EST_C_1_pSK_SK	453
cl0506	ct0533	cn0552	pOP-CNH02730_EST_C_1_pSK_SK	480
cl0506	ct0533	cn0552	pOP-CNH03058_EST_C_1_pSK_SK	683
cl0506	ct0533	cn0552	pOP-CNI01881_EST_C_1_pSK_SK	452
cl0506	ct0533	cn0552	pOP-EO06072_EST_C_1_pSK_SK	704
cl0507	ct0534	cn0553	pOP-CEO01670_EST_C_1_pSK_SK	366
cl0507	ct0534	cn0553	pOP-CNI01885_EST_C_1_pSK_SK	118

cl0508	ct0535	cn0554	pOP-CNH02643_EST_C_1_pSK_SK	634
cl0508	ct0535	cn0554	pOP-CNH03145_EST_C_1_pSK_SK	466
cl0508	ct0535	cn0554	pOP-CNH03146_EST_C_1_pSK_SK	633
cl0508	ct0535	cn0554	pOP-CNH03622_EST_C_1_pSK_SK	613
cl0508	ct0535	cn0554	pOP-CNH04233	459
cl0508	ct0535	cn0554	pOP-CNH04799_EST_C_1_pSK_SK	526
cl0508	ct0535	cn0554	pOP-CNI01890_EST_C_1_pSK_SK	434
cl0509	ct0536	cn0555	pOP-CNH00993_EST_C_1_pSK_SK	635
cl0509	ct0536	cn0555	pOP-CNI01487_EST_C_1_pSK_SK	640
cl0509	ct0536	cn0555	pOP-CNI01492_EST_C_1_pSK_SK	535
cl0509	ct0536	cn0555	pOP-CNI01896_EST_C_1_pSK_SK	457
cl0510	ct0537	cn0556	pOP-CNI01418_EST_C_1_pSK_SK	349
cl0510	ct0537	cn0556	pOP-CNI01915_EST_C_1_pSK_SK	448
cl0511	ct0538	cn0557	pOP-CNHP00515_EST_C_1_pSK_SK	609
cl0511	ct0538	cn0557	pOP-CNI01927_EST_C_1_pSK_SK	526
cl0512	ct0539	cn0558	pOP-CNH01314_EST_C_1_pSK_SK	564
cl0512	ct0539	cn0558	pOP-CNI01939_EST_C_1_pSK_SK	266
cl0513	ct0540	cn0559	pOP-CNI01428_EST_C_1_pSK_SK	262
cl0513	ct0540	cn0559	pOP-CNI01947_EST_C_1_pSK_SK	442
cl0513	ct0540	cn0559	pOP-EO08280_EST_C_1_pSK_SK	553
cl0514	ct0541	cn0560	pOP-CNI01760_EST_C_1_pSK_SK	402
cl0514	ct0541	cn0560	pOP-CNI01951_EST_C_1_pSK_SK	516
cl0515	ct0542	cn0561	pOP-CNI01964_EST_C_1_pSK_SK	374
cl0515	ct0542	cn0561	pOP-CNIP00355_EST_C_1_pSK_SK	249
cl0516	ct0543	cn0562	pOP-CNI01969_EST_C_1_pSK_SK	395
cl0516	ct0544	cn0563	pOP-CNH01773_EST_C_1_pSK_SK	519
cl0517	ct0545	cn0564	pOP-CEO03171_EST_C_1_pSK_SK	520
cl0517	ct0545	cn0564	pOP-CNI01972_EST_C_1_pSK_SK	268
cl0518	ct0546	cn0565	pOP-CNH04457	399
cl0518	ct0546	cn0565	pOP-CNI01999_EST_C_1_pSK_SK	638
cl0519	ct0547	cn0566	pOP-CNH02636_EST_C_1_pSK_SK	668
cl0519	ct0547	cn0566	pOP-CNH02798_EST_C_1_pSK_SK	625
cl0519	ct0547	cn0566	pOP-CNI02020_EST_C_1_pSK_SK	417
cl0520	ct0548	cn0567	pOP-CNI02028_EST_C_1_pSK_SK	501
cl0520	ct0548	cn0567	pOP-EO06686_EST_C_1_pSK_SK	676
cl0520	ct0548	cn0567	pOP-EO06737_EST_C_1_pSK_SK	761
cl0521	ct0549	cn0568	pOP-CNH00939_EST_C_1_pSK_SK	556
cl0521	ct0549	cn0568	pOP-CNI01164_EST_C_1_pSK_SK	614
cl0521	ct0549	cn0568	pOP-CNI02036_EST_C_1_pSK_SK	272
cl0521	ct0549	cn0568	pOP-EO03084_EST_C_1_pSK_SK	418
cl0521	ct0549	cn0568	pOP-EO03372_EST_C_1_pSK_SK	391
cl0521	ct0549	cn0568	pOP-EO07093_EST_C_1_pSK_SK	636
cl0522	ct0550	cn0569	pOP-CNI01807_EST_C_1_pSK_SK	100
cl0522	ct0550	cn0569	pOP-CNI02046_EST_C_1_pSK_SK	100
cl0523	ct0551	cn0570	pOP-CNI02047_EST_C_1_pSK_SK	525
cl0523	ct0551	cn0570	pOP-EO05664_EST_C_1_pSK_SK	479
cl0524	ct0552	cn0571	pOP-CEO03463_EST_C_1_pSK_SK	382
cl0524	ct0552	cn0571	pOP-CNI02049_EST_C_1_pSK_SK	401
cl0525	ct0553	cn0572	pOP-CNH03069_EST_C_1_pSK_SK	648
cl0525	ct0553	cn0572	pOP-CNI02063_EST_C_1_pSK_SK	295
cl0525	ct0554	cn0573	pOP-CNH01420_EST_C_1_pSK_SK	555
cl0525	ct0554	cn0573	pOP-CNH03059_EST_C_1_pSK_SK	553
cl0525	ct0554	cn0573	pOP-EAP01829_EST_C_1_pBSK_SK	633
cl0525	ct0554	cn0573	pOP-EAP03278_EST_C_1_pBSK_SK	465
cl0526	ct0555	cn0574	pOP-CNI02066_EST_C_1_pSK_SK	564
cl0526	ct0555	cn0574	pOP-EO06629_EST_C_1_pSK_SK	573

cl0526	ct0555	cn0574	pOP-EO06794_EST_C_1_pSK_SK	570
cl0527	ct0556	cn0575	pOP-CNH03039_EST_C_1_pSK_SK	630
cl0527	ct0557	cn0576	pOP-CNH02749_EST_C_1_pSK_SK	614
cl0527	ct0557	cn0576	pOP-CNH03355_EST_C_1_pSK_SK	660
cl0527	ct0557	cn0576	pOP-CNH03357_EST_C_1_pSK_SK	606
cl0527	ct0557	cn0576	pOP-CNI02073_EST_C_1_pSK_SK	228
cl0528	ct0558	cn0577	pOP-CNH00889_EST_C_1_pSK_SK	523
cl0528	ct0558	cn0577	pOP-CNH02657_EST_C_1_pSK_SK	542
cl0528	ct0558	cn0577	pOP-CNI02075_EST_C_1_pSK_SK	565
cl0529	ct0559	cn0578	pOP-CNI01335_EST_C_1_pSK_SK	708
cl0529	ct0559	cn0578	pOP-CNI02082_EST_C_1_pSK_SK	420
cl0530	ct0560	cn0579	pOP-CNH00989_EST_C_1_pSK_SK	399
cl0530	ct0560	cn0579	pOP-CNI01341_EST_C_1_pSK_SK	550
cl0530	ct0560	cn0579	pOP-CNI01377_EST_C_1_pSK_SK	247
cl0530	ct0560	cn0579	pOP-CNI01704_EST_C_1_pSK_SK	216
cl0530	ct0560	cn0579	pOP-CNI02106_EST_C_1_pSK_SK	346
cl0531	ct0561	cn0580	pOP-CNI01625_EST_C_1_pSK_SK	310
cl0531	ct0561	cn0580	pOP-CNI02119_EST_C_1_pSK_SK	350
cl0532	ct0562	cn0581	pOP-CNI01710_EST_C_1_pSK_SK	369
cl0532	ct0562	cn0581	pOP-CNI01955_EST_C_1_pSK_SK	456
cl0532	ct0562	cn0581	pOP-CNI02121_EST_C_1_pSK_SK	367
cl0533	ct0563	cn0582	pOP-CNI02124_EST_C_1_pSK_SK	260
cl0533	ct0563	cn0582	pOP-EO05464_EST_C_1_pSK_SK	484
cl0534	ct0564	cn0583	pOP-CNH01253_EST_C_1_pSK_SK	529
cl0534	ct0564	cn0583	pOP-CNH02177_EST_C_1_pSK_SK	522
cl0534	ct0564	cn0583	pOP-CNH04801_EST_C_1_pSK_SK	507
cl0534	ct0564	cn0583	pOP-CNI02131_EST_C_1_pSK_SK	624
cl0535	ct0565	cn0584	pOP-CNI02144_EST_C_1_pSK_SK	452
cl0535	ct0565	cn0584	pOP-EO05909_EST_C_1_pSK_SK	402
cl0536	ct0566	cn0585	pOP-CNH00870_EST_C_1_pSK_SK	687
cl0536	ct0566	cn0585	pOP-CNI02148_EST_C_1_pSK_SK	681
cl0537	ct0567	cn0586	pOP-CNH02516_EST_C_1_pSK_SK	461
cl0537	ct0567	cn0586	pOP-CNH03467_EST_C_1_pSK_SK	543
cl0537	ct0567	cn0586	pOP-CNH04780_EST_C_1_pSK_SK	544
cl0537	ct0567	cn0586	pOP-CNI02164_EST_C_1_pSK_SK	422
cl0538	ct0568	cn0587	pOP-CNI01446_EST_C_1_pSK_SK	179
cl0538	ct0568	cn0587	pOP-CNI02180_EST_C_1_pSK_SK	327
cl0539	ct0569	cn0588	pOP-CNI01530_EST_C_1_pSK_SK	416
cl0539	ct0569	cn0588	pOP-CNI02182_EST_C_1_pSK_SK	416
cl0540	ct0570	cn0589	pOP-CNH02788_EST_C_1_pSK_SK	610
cl0540	ct0570	cn0589	pOP-CNH04927_EST_C_1_pSK_SK	688
cl0540	ct0570	cn0589	pOP-CNI02185_EST_C_1_pSK_SK	616
cl0541	ct0571	cn0590	pOP-CEO03660_EST_C_1_pSK_SK	349
cl0541	ct0571	cn0590	pOP-CNI02186_EST_C_1_pSK_SK	552
cl0542	ct0572	cn0591	pOP-CNH01274_EST_C_1_pSK_SK	562
cl0542	ct0572	cn0591	pOP-CNH02698_EST_C_1_pSK_SK	599
cl0542	ct0572	cn0591	pOP-CNI02197_EST_C_1_pSK_SK	504
cl0542	ct0572	cn0591	pOP-EO08516_EST_C_1_pSK_SK	251
cl0543	ct0573	cn0592	pOP-CNH02590_EST_C_1_pSK_SK	488
cl0543	ct0573	cn0592	pOP-CNI02231_EST_C_1_pSK_SK	315
cl0544	ct0574	cn0593	pOP-CNH02961_EST_C_1_pSK_SK	601
cl0544	ct0574	cn0593	pOP-CNI02236_EST_C_1_pSK_SK	508
cl0545	ct0575	cn0594	pOP-CNIP00001_EST_C_1_pSK_SK	451
cl0545	ct0575	cn0594	pOP-EN00142_EST_C_1_pSK_SK	515
cl0545	ct0575	cn0594	pOP-EO05778_EST_C_1_pSK_SK	613
cl0545	ct0575	cn0594	pOP-EO06595_EST_C_1_pSK_SK	728

cl0546	ct0576	cn0595	pOP-CNIP00010_EST_C_1_pSK_SK	440
cl0546	ct0577	cn0596	pOP-CNH02780_EST_C_1_pSK_SK	593
cl0546	ct0577	cn0596	pOP-CNHP00099_EST_C_1_pSK_SK	584
cl0547	ct0578	cn0597	pOP-CNH04693	654
cl0547	ct0578	cn0597	pOP-CNH04912_EST_C_1_pSK_SK	721
cl0547	ct0578	cn0597	pOP-CNIP00014_EST_C_1_pSK_SK	248
cl0548	ct0579	cn0598	pOP-CNI02013_EST_C_1_pSK_SK	207
cl0548	ct0579	cn0598	pOP-CNIP00040_EST_C_1_pSK_SK	440
cl0549	ct0580	cn0599	pOP-CEO01283	322
cl0549	ct0580	cn0599	pOP-CNIP00041_EST_C_1_pSK_SK	449
cl0550	ct0581	cn0600	pOP-CNI01729_EST_C_1_pSK_SK	530
cl0550	ct0581	cn0600	pOP-CNIP00068_EST_C_1_pSK_SK	533
cl0551	ct0582	cn0601	pOP-CNI01817_EST_C_1_pSK_SK	148
cl0551	ct0582	cn0601	pOP-CNIP00074_EST_C_1_pSK_SK	335
cl0552	ct0583	cn0602	pOP-CNI01342_EST_C_1_pSK_SK	373
cl0552	ct0583	cn0602	pOP-CNIP00077_EST_C_1_pSK_SK	579
cl0553	ct0584	cn0603	pOP-CEO02432_EST_C_1_pSK_SK	409
cl0553	ct0584	cn0603	pOP-CNI01612_EST_C_1_pSK_SK	201
cl0553	ct0584	cn0603	pOP-CNIP00100_EST_C_1_pSK_SK	183
cl0554	ct0585	cn0604	pOP-CNIP00135_EST_C_1_pSK_SK	472
cl0554	ct0586	cn0605	pOP-CNH02743_EST_C_1_pSK_SK	640
cl0554	ct0586	cn0605	pOP-CNH04286	486
cl0555	ct0587	cn0606	pOP-CNIP00097_EST_C_1_pSK_SK	529
cl0555	ct0587	cn0606	pOP-CNIP00164_EST_C_1_pSK_SK	264
cl0556	ct0588	cn0607	pOP-CNI01620_EST_C_1_pSK_SK	405
cl0556	ct0588	cn0607	pOP-CNI01692_EST_C_1_pSK_SK	254
cl0556	ct0588	cn0607	pOP-CNI02053_EST_C_1_pSK_SK	237
cl0556	ct0588	cn0607	pOP-CNIP00177_EST_C_1_pSK_SK	490
cl0557	ct0589	cn0608	pOP-CNHP00398_EST_C_1_pSK_SK	655
cl0557	ct0589	cn0608	pOP-CNIP00181_EST_C_1_pSK_SK	505
cl0558	ct0590	cn0609	pOP-CNI01315_EST_C_1_pSK_SK	468
cl0558	ct0590	cn0609	pOP-CNIP00193_EST_C_1_pSK_SK	423
cl0559	ct0591	cn0610	pOP-CNI01101_EST_C_1_pSK_SK	527
cl0559	ct0591	cn0610	pOP-CNI02003_EST_C_1_pSK_SK	599
cl0559	ct0591	cn0610	pOP-CNIP00194_EST_C_1_pSK_SK	444
cl0560	ct0592	cn0611	pOP-CNI01504_EST_C_1_pSK_SK	238
cl0560	ct0592	cn0611	pOP-CNIP00201_EST_C_1_pSK_SK	251
cl0561	ct0593	cn0612	pOP-CNI01081_EST_C_1_pSK_SK	656
cl0561	ct0593	cn0612	pOP-CNIP00205_EST_C_1_pSK_SK	430
cl0562	ct0594	cn0613	pOP-CNI02176_EST_C_1_pSK_SK	371
cl0562	ct0594	cn0613	pOP-CNIP00229_EST_C_1_pSK_SK	396
cl0563	ct0595	cn0614	pOP-CNHP00388_EST_C_1_pSK_SK	564
cl0563	ct0595	cn0615	pOP-CNIP00230_EST_C_1_pSK_SK	530
cl0564	ct0596	cn0616	pOP-CNI01502_EST_C_1_pSK_SK	439
cl0564	ct0596	cn0616	pOP-CNIP00235_EST_C_1_pSK_SK	437
cl0565	ct0597	cn0617	pOP-CEM00130_EST_C_1_pSK_SK	418
cl0565	ct0597	cn0617	pOP-CEO03075_EST_C_1_pSK_SK	400
cl0565	ct0597	cn0617	pOP-CNIP00244_EST_C_1_pSK_SK	267
cl0565	ct0597	cn0617	pOP-CNIP00579_EST_C_1_pSK_SK	391
cl0565	ct0598	cn0618	pOP-CEO01912_EST_C_1_pSK_SK	343
cl0565	ct0598	cn0618	pOP-CEO02929_EST_C_1_pSK_SK	342
cl0565	ct0598	cn0618	pOP-CEO02988_EST_C_1_pSK_SK	710
cl0565	ct0598	cn0618	pOP-CNH03261_EST_C_1_pSK_SK	427
cl0565	ct0598	cn0618	pOP-CNHP00157_EST_C_1_pSK_SK	347
cl0565	ct0598	cn0618	pOP-CNI01077_EST_C_1_pSK_SK	475
cl0565	ct0598	cn0618	pOP-CNI01242_EST_C_1_pSK_SK	364

cl0565	ct0598	cn0618	pOP-CNI01535_EST_C_1_pSK_SK	374
cl0565	ct0598	cn0618	pOP-CNI01633_EST_C_1_pSK_SK	387
cl0565	ct0598	cn0618	pOP-CNI01931_EST_C_1_pSK_SK	475
cl0565	ct0598	cn0618	pOP-CNI02175_EST_C_1_pSK_SK	462
cl0565	ct0598	cn0618	pOP-CNIP00673_EST_C_1_pSK_SK	491
cl0565	ct0598	cn0618	pOP-CNIP00918_EST_C_1_pSK_SK	549
cl0565	ct0598	cn0618	pOP-EAP01137_EST_C_1_pBSK_SK	533
cl0565	ct0598	cn0618	pOP-EAP03361_EST_C_1_pBSK_SK	592
cl0565	ct0598	cn0618	pOP-EAP03663_EST_C_1_pBSK_SK	383
cl0565	ct0598	cn0618	pOP-EO03722_EST_C_1_pSK_SK	352
cl0565	ct0598	cn0619	pOP-CNI01349_EST_C_1_pSK_SK	633
cl0566	ct0599	cn0620	pOP-CNI02179_EST_C_1_pSK_SK	327
cl0566	ct0599	cn0620	pOP-CNIP00271_EST_C_1_pSK_SK	257
cl0567	ct0600	cn0621	pOP-CNI01820_EST_C_1_pSK_SK	346
cl0567	ct0600	cn0621	pOP-CNIP00283_EST_C_1_pSK_SK	260
cl0568	ct0601	cn0622	pOP-CNI01413_EST_C_1_pSK_SK	287
cl0568	ct0601	cn0622	pOP-CNI01414_EST_C_1_pSK_SK	559
cl0568	ct0601	cn0622	pOP-CNIP00289_EST_C_1_pSK_SK	257
cl0569	ct0602	cn0623	pOP-CNH00807_EST_C_1_pSK_SK	666
cl0569	ct0602	cn0623	pOP-CNIP00290_EST_C_1_pSK_SK	344
cl0569	ct0602	cn0624	pOP-CNI01257_EST_C_1_pSK_SK	373
cl0570	ct0603	cn0625	pOP-CNI01974_EST_C_1_pSK_SK	371
cl0570	ct0603	cn0625	pOP-CNIP00313_EST_C_1_pSK_SK	602
cl0571	ct0604	cn0626	pOP-CNH03017_EST_C_1_pSK_SK	546
cl0571	ct0604	cn0626	pOP-CNI01721_EST_C_1_pSK_SK	307
cl0571	ct0604	cn0626	pOP-CNIP00316_EST_C_1_pSK_SK	528
cl0572	ct0605	cn0627	pOP-CNH01532_EST_C_1_pSK_SK	584
cl0572	ct0605	cn0627	pOP-CNIP00323_EST_C_1_pSK_SK	134
cl0573	ct0606	cn0628	pOP-CNI01553_EST_C_1_pSK_SK	358
cl0573	ct0606	cn0628	pOP-CNIP00340_EST_C_1_pSK_SK	428
cl0574	ct0607	cn0629	pOP-CNH04917_EST_C_1_pSK_SK	537
cl0574	ct0607	cn0629	pOP-CNH05050_EST_C_1_pSK_SK	528
cl0574	ct0607	cn0629	pOP-CNIP00348_EST_C_1_pSK_SK	308
cl0575	ct0608	cn0630	pOP-CNI02011_EST_C_1_pSK_SK	382
cl0575	ct0608	cn0630	pOP-CNIP00178_EST_C_1_pSK_SK	209
cl0575	ct0608	cn0630	pOP-CNIP00358_EST_C_1_pSK_SK	371
cl0576	ct0609	cn0631	pOP-CNIP00163_EST_C_1_pSK_SK	324
cl0576	ct0609	cn0631	pOP-CNIP00214_EST_C_1_pSK_SK	324
cl0576	ct0609	cn0631	pOP-CNIP00257_EST_C_1_pSK_SK	324
cl0576	ct0609	cn0631	pOP-CNIP00359_EST_C_1_pSK_SK	504
cl0577	ct0610	cn0632	pOP-CNI01447_EST_C_1_pSK_SK	169
cl0577	ct0610	cn0632	pOP-CNIP00389_EST_C_1_pSK_SK	337
cl0578	ct0611	cn0633	pOP-CNH05019_EST_C_1_pSK_SK	111
cl0578	ct0611	cn0633	pOP-CNIP00390_EST_C_1_pSK_SK	191
cl0579	ct0612	cn0634	pOP-CNI01911_EST_C_1_pSK_SK	267
cl0579	ct0612	cn0634	pOP-CNIP00393_EST_C_1_pSK_SK	377
cl0580	ct0613	cn0635	pOP-CNI01638_EST_C_1_pSK_SK	268
cl0580	ct0613	cn0635	pOP-CNIP00428_EST_C_1_pSK_SK	149
cl0581	ct0614	cn0636	pOP-CNH05078_EST_C_1_pSK_SK	697
cl0581	ct0614	cn0636	pOP-CNIP00460_EST_C_1_pSK_SK	508
cl0582	ct0615	cn0637	pOP-CNH01116_EST_C_1_pSK_SK	271
cl0582	ct0615	cn0637	pOP-CNH03767_EST_C_1_pSK_SK	436
cl0582	ct0615	cn0637	pOP-CNHP00101_EST_C_1_pSK_SK	275
cl0582	ct0615	cn0637	pOP-CNHP00174_EST_C_1_pSK_SK	553
cl0582	ct0615	cn0637	pOP-CNI01703_EST_C_1_pSK_SK	377
cl0582	ct0615	cn0637	pOP-CNIP00471_EST_C_1_pSK_SK	434

cl0582	ct0615	cn0637	pOP-CNIP00878_EST_C_1_pSK_SK	552
cl0582	ct0615	cn0637	pOP-EAP02712_EST_C_1_pBSK_SK	631
cl0582	ct0615	cn0637	pOP-EAP03622_EST_C_1_pBSK_SK	600
cl0582	ct0615	cn0637	pOP-EAP03788_EST_C_1_pBSK_SK	371
cl0583	ct0616	cn0638	pOP-CNH04952_EST_C_1_pSK_SK	518
cl0583	ct0616	cn0638	pOP-CNIP00421_EST_C_1_pSK_SK	421
cl0583	ct0616	cn0638	pOP-CNIP00477_EST_C_1_pSK_SK	290
cl0584	ct0617	cn0639	pOP-CNI01986_EST_C_1_pSK_SK	383
cl0584	ct0617	cn0639	pOP-CNIP00481_EST_C_1_pSK_SK	613
cl0585	ct0618	cn0640	pOP-CEO01323	448
cl0585	ct0618	cn0640	pOP-CNIP00487_EST_C_1_pSK_SK	325
cl0586	ct0619	cn0641	pOP-CNI01364_EST_C_1_pSK_SK	384
cl0586	ct0619	cn0641	pOP-CNIP00511_EST_C_1_pSK_SK	208
cl0587	ct0620	cn0642	pOP-CNI01106_EST_C_1_pSK_SK	399
cl0587	ct0620	cn0642	pOP-CNI01122_EST_C_1_pSK_SK	480
cl0587	ct0620	cn0642	pOP-CNIP00325_EST_C_1_pSK_SK	377
cl0587	ct0620	cn0642	pOP-CNIP00515_EST_C_1_pSK_SK	395
cl0588	ct0621	cn0643	pOP-CNH04324	607
cl0588	ct0621	cn0643	pOP-CNI01172_EST_C_1_pSK_SK	656
cl0588	ct0621	cn0643	pOP-CNIP00516_EST_C_1_pSK_SK	507
cl0589	ct0622	cn0644	pOP-CNI01118_EST_C_1_pSK_SK	411
cl0589	ct0623	cn0645	pOP-CNIP00238_EST_C_1_pSK_SK	248
cl0589	ct0623	cn0645	pOP-CNIP00528_EST_C_1_pSK_SK	374
cl0590	ct0624	cn0646	pOP-CNH03221_EST_C_1_pSK_SK	539
cl0590	ct0624	cn0646	pOP-CNIP00543_EST_C_1_pSK_SK	364
cl0591	ct0625	cn0647	pOP-CNH03048_EST_C_1_pSK_SK	551
cl0591	ct0625	cn0647	pOP-CNIP00575_EST_C_1_pSK_SK	478
cl0592	ct0626	cn0648	pOP-CNHP00389_EST_C_1_pSK_SK	691
cl0592	ct0626	cn0648	pOP-CNIP00582_EST_C_1_pSK_SK	298
cl0593	ct0627	cn0649	pOP-CNIP00418_EST_C_1_pSK_SK	370
cl0593	ct0627	cn0649	pOP-CNIP00591_EST_C_1_pSK_SK	369
cl0594	ct0628	cn0650	pOP-CNH01328_EST_C_1_pSK_SK	564
cl0594	ct0628	cn0650	pOP-CNH02081_EST_C_1_pSK_SK	492
cl0594	ct0628	cn0650	pOP-CNH02606_EST_C_1_pSK_SK	439
cl0594	ct0628	cn0650	pOP-CNH03019_EST_C_1_pSK_SK	555
cl0594	ct0628	cn0650	pOP-CNH03086_EST_C_1_pSK_SK	548
cl0594	ct0628	cn0650	pOP-CNH03334_EST_C_1_pSK_SK	694
cl0594	ct0628	cn0650	pOP-CNH03506_EST_C_1_pSK_SK	427
cl0594	ct0628	cn0650	pOP-CNH04651	701
cl0594	ct0628	cn0650	pOP-CNH04943_EST_C_1_pSK_SK	657
cl0594	ct0628	cn0650	pOP-CNHP00404_EST_C_1_pSK_SK	591
cl0594	ct0628	cn0650	pOP-CNIP00592_EST_C_1_pSK_SK	201
cl0594	ct0628	cn0650	pOP-EAP02128_EST_C_1_pBSK_SK	495
cl0594	ct0628	cn0650	pOP-EO08321_EST_C_1_pSK_SK	463
cl0595	ct0629	cn0651	pOP-CNH02311_EST_C_1_pSK_SK	650
cl0595	ct0629	cn0651	pOP-CNH04447	705
cl0595	ct0629	cn0651	pOP-CNIP00593_EST_C_1_pSK_SK	347
cl0596	ct0630	cn0652	pOP-CNHP00316_EST_C_1_pSK_SK	578
cl0596	ct0630	cn0652	pOP-CNIP00597_EST_C_1_pSK_SK	352
cl0597	ct0631	cn0653	pOP-CNIP00571_EST_C_1_pSK_SK	190
cl0597	ct0631	cn0653	pOP-CNIP00604_EST_C_1_pSK_SK	404
cl0598	ct0632	cn0654	pOP-CNH02120_EST_C_1_pSK_SK	454
cl0598	ct0632	cn0655	pOP-CNH01989_EST_C_1_pSK_SK	446
cl0598	ct0632	cn0655	pOP-CNIP00620_EST_C_1_pSK_SK	431
cl0599	ct0633	cn0656	pOP-CNIP00116_EST_C_1_pSK_SK	446
cl0599	ct0633	cn0656	pOP-CNIP00656_EST_C_1_pSK_SK	374

cl0600	ct0634	cn0657	pOP-CEO02375_EST_C_1_pSK_SK	326
cl0600	ct0634	cn0657	pOP-CEO02376_EST_C_1_pSK_SK	290
cl0600	ct0634	cn0657	pOP-CNH00715_EST_C_1_pSK_SK	581
cl0600	ct0634	cn0657	pOP-CNIP00663_EST_C_1_pSK_SK	430
cl0600	ct0634	cn0657	pOP-EO07863_EST_C_1_pSK_SK	660
cl0601	ct0635	cn0658	pOP-CNIP00681_EST_C_1_pSK_SK	287
cl0601	ct0636	cn0659	pOP-CNH01985_EST_C_1_pSK_SK	567
cl0602	ct0637	cn0660	pOP-CNI01153_EST_C_1_pSK_SK	620
cl0602	ct0637	cn0660	pOP-CNIP00705_EST_C_1_pSK_SK	600
cl0603	ct0638	cn0661	pOP-CNIP00023_EST_C_1_pSK_SK	215
cl0603	ct0638	cn0661	pOP-CNIP00718_EST_C_1_pSK_SK	310
cl0604	ct0639	cn0662	pOP-CNI01567_EST_C_1_pSK_SK	193
cl0604	ct0639	cn0662	pOP-CNIP00721_EST_C_1_pSK_SK	530
cl0605	ct0640	cn0663	pOP-CNI01217_EST_C_1_pSK_SK	644
cl0605	ct0640	cn0663	pOP-CNIP00724_EST_C_1_pSK_SK	342
cl0605	ct0641	cn0664	pOP-CNH01006_EST_C_1_pSK_SK	288
cl0605	ct0641	cn0664	pOP-CNH01133_EST_C_1_pSK_SK	540
cl0605	ct0641	cn0664	pOP-CNH01413_EST_C_1_pSK_SK	530
cl0605	ct0641	cn0664	pOP-CNH02303_EST_C_1_pSK_SK	638
cl0605	ct0641	cn0664	pOP-CNH03077_EST_C_1_pSK_SK	662
cl0605	ct0641	cn0664	pOP-CNH03665_EST_C_1_pSK_SK	435
cl0605	ct0641	cn0664	pOP-CNH03727_EST_C_1_pSK_SK	408
cl0605	ct0641	cn0664	pOP-CNH04172	238
cl0605	ct0641	cn0664	pOP-CNH04211	629
cl0605	ct0641	cn0664	pOP-CNH04446	393
cl0605	ct0641	cn0664	pOP-CNHP00117_EST_C_1_pSK_SK	475
cl0605	ct0641	cn0664	pOP-CNHP00366_EST_C_1_pSK_SK	381
cl0605	ct0641	cn0664	pOP-CNIP00015_EST_C_1_pSK_SK	341
cl0605	ct0641	cn0664	pOP-CNIP00273_EST_C_1_pSK_SK	469
cl0606	ct0642	cn0665	pOP-CNIP00212_EST_C_1_pSK_SK	513
cl0606	ct0642	cn0665	pOP-CNIP00726_EST_C_1_pSK_SK	643
cl0607	ct0643	cn0666	pOP-CNI02090_EST_C_1_pSK_SK	586
cl0607	ct0643	cn0666	pOP-CNIP00739_EST_C_1_pSK_SK	354
cl0608	ct0644	cn0667	pOP-CNH02813_EST_C_1_pSK_SK	531
cl0608	ct0644	cn0667	pOP-CNIP00742_EST_C_1_pSK_SK	562
cl0609	ct0645	cn0668	pOP-CNIP00191_EST_C_1_pSK_SK	408
cl0609	ct0645	cn0668	pOP-CNIP00750_EST_C_1_pSK_SK	261
cl0610	ct0646	cn0669	pOP-CNH00697_EST_C_1_pSK_SK	524
cl0610	ct0646	cn0669	pOP-CNH02367_EST_C_1_pSK_SK	502
cl0610	ct0646	cn0669	pOP-CNH02908_EST_C_1_pSK_SK	511
cl0610	ct0646	cn0669	pOP-CNH03514_EST_C_1_pSK_SK	602
cl0610	ct0646	cn0669	pOP-CNI01238_EST_C_1_pSK_SK	746
cl0610	ct0646	cn0669	pOP-CNIP00434_EST_C_1_pSK_SK	595
cl0610	ct0646	cn0669	pOP-EO03207_EST_C_1_pSK_SK	459
cl0610	ct0646	cn0669	pOP-EO05694_EST_C_1_pSK_SK	440
cl0610	ct0646	cn0669	pOP-EO08375_EST_C_1_pSK_SK	486
cl0610	ct0646	cn0670	pOP-CNIP00754_EST_C_1_pSK_SK	641
cl0611	ct0647	cn0671	pOP-CNI01970_EST_C_1_pSK_SK	493
cl0611	ct0647	cn0671	pOP-CNIP00757_EST_C_1_pSK_SK	166
cl0612	ct0648	cn0672	pOP-CNH01315_EST_C_1_pSK_SK	783
cl0612	ct0648	cn0673	pOP-CNI01370_EST_C_1_pSK_SK	403
cl0612	ct0648	cn0673	pOP-CNIP00760_EST_C_1_pSK_SK	196
cl0613	ct0649	cn0674	pOP-CNH02249_EST_C_1_pSK_SK	678
cl0613	ct0649	cn0674	pOP-CNH04496	739
cl0613	ct0649	cn0674	pOP-CNH04935_EST_C_1_pSK_SK	740
cl0613	ct0649	cn0674	pOP-CNIP00763_EST_C_1_pSK_SK	741

cl0614	ct0650	cn0675	pOP-CNH00969_EST_C_1_pSK_SK	623
cl0614	ct0650	cn0675	pOP-CNH02209_EST_C_1_pSK_SK	630
cl0614	ct0650	cn0675	pOP-CNH04631	813
cl0614	ct0650	cn0675	pOP-CNIP00779_EST_C_1_pSK_SK	321
cl0615	ct0651	cn0676	pOP-CNIP00748_EST_C_1_pSK_SK	558
cl0615	ct0651	cn0676	pOP-CNIP00785_EST_C_1_pSK_SK	668
cl0616	ct0652	cn0677	pOP-CNIP00758_EST_C_1_pSK_SK	306
cl0616	ct0652	cn0677	pOP-CNIP00796_EST_C_1_pSK_SK	306
cl0617	ct0653	cn0678	pOP-CNI01133_EST_C_1_pSK_SK	565
cl0617	ct0653	cn0678	pOP-CNIP00797_EST_C_1_pSK_SK	441
cl0618	ct0654	cn0679	pOP-CNHP00054_EST_C_1_pSK_SK	621
cl0618	ct0654	cn0679	pOP-CNIP00809_EST_C_1_pSK_SK	433
cl0619	ct0655	cn0680	pOP-CNIP00827_EST_C_1_pSK_SK	335
cl0619	ct0656	cn0681	pOP-CNI01785_EST_C_1_pSK_SK	294
cl0619	ct0657	cn0682	pOP-CNH00887_EST_C_1_pSK_SK	476
cl0619	ct0657	cn0682	pOP-CNH00948_EST_C_1_pSK_SK	469
cl0619	ct0657	cn0682	pOP-CNH02040_EST_C_1_pSK_SK	435
cl0619	ct0657	cn0682	pOP-CNH02100_EST_C_1_pSK_SK	561
cl0619	ct0658	cn0683	pOP-CNH01167_EST_C_1_pSK_SK	400
cl0619	ct0658	cn0683	pOP-CNH01473_EST_C_1_pSK_SK	630
cl0619	ct0658	cn0683	pOP-CNH01683_EST_C_1_pSK_SK	630
cl0619	ct0658	cn0683	pOP-CNH01699_EST_C_1_pSK_SK	646
cl0619	ct0658	cn0683	pOP-CNH02764_EST_C_1_pSK_SK	714
cl0619	ct0658	cn0683	pOP-CNH02943_EST_C_1_pSK_SK	538
cl0619	ct0658	cn0683	pOP-CNH03051_EST_C_1_pSK_SK	649
cl0619	ct0658	cn0683	pOP-CNH03156_EST_C_1_pSK_SK	557
cl0619	ct0658	cn0683	pOP-CNH03333_EST_C_1_pSK_SK	643
cl0619	ct0658	cn0683	pOP-CNH03482_EST_C_1_pSK_SK	521
cl0619	ct0658	cn0683	pOP-CNH04433	642
cl0619	ct0658	cn0683	pOP-CNH04494	490
cl0619	ct0658	cn0683	pOP-CNH04618	642
cl0620	ct0659	cn0684	pOP-CNIP00714_EST_C_1_pSK_SK	397
cl0620	ct0659	cn0684	pOP-CNIP00839_EST_C_1_pSK_SK	270
cl0621	ct0660	cn0685	pOP-CNIP00860_EST_C_1_pSK_SK	412
cl0621	ct0660	cn0685	pOP-CNIP00889_EST_C_1_pSK_SK	397
cl0622	ct0661	cn0686	pOP-CNIP00912_EST_C_1_pSK_SK	393
cl0622	ct0661	cn0686	pOP-EO04428_EST_C_1_pSK_SK	481
cl0623	ct0662	cn0687	pOP-CNIP00017_EST_C_1_pSK_SK	417
cl0623	ct0662	cn0687	pOP-CNIP00914_EST_C_1_pSK_SK	242
cl0624	ct0663	cn0688	pOP-EO02116_EST_C_1_pSK_SK	643
cl0624	ct0663	cn0689	pOP-CNIP00928_EST_C_1_pSK_SK	621
cl0624	ct0664	cn0690	pOP-CEO01668_EST_C_1_pSK_SK	590
cl0624	ct0664	cn0690	pOP-CNH04336	757
cl0624	ct0664	cn0691	pOP-EO02206_EST_C_1_pSK_SK	380
cl0624	ct0664	cn0691	pOP-EO02677_EST_C_1_pSK_SK	263
cl0624	ct0665	cn0692	pOP-CEO02383_EST_C_1_pSK_SK	415
cl0624	ct0665	cn0692	pOP-CNI01615_EST_C_1_pSK_SK	498
cl0624	ct0665	cn0692	pOP-CNIP00173_EST_C_1_pSK_SK	498
cl0624	ct0665	cn0692	pOP-EAP00806_EST_C_1_pBSK_SK	438
cl0624	ct0665	cn0692	pOP-EAP00903_EST_C_1_pBSK_SK	369
cl0625	ct0666	cn0693	pOP-CNIP00929_EST_C_1_pSK_SK	699
cl0625	ct0666	cn0693	pOP-EO04855_EST_C_1_pSK_SK	485
cl0626	ct0667	cn0694	pOP-CNI01437_EST_C_1_pSK_SK	477
cl0626	ct0667	cn0694	pOP-CNIP00931_EST_C_1_pSK_SK	398
cl0627	ct0668	cn0695	pOP-CEO01311	474
cl0627	ct0668	cn0695	pOP-CNIP00939_EST_C_1_pSK_SK	184

cl0628	ct0669	cn0696	pOP-CNIP00405_EST_C_1_pSK_SK	427
cl0628	ct0669	cn0696	pOP-CNIP00942_EST_C_1_pSK_SK	574
cl0629	ct0670	cn0697	pOP-CNIP00032_EST_C_1_pSK_SK	514
cl0629	ct0670	cn0697	pOP-CNIP00945_EST_C_1_pSK_SK	514
cl0630	ct0671	cn0698	pOP-CNH02417_EST_C_1_pSK_SK	503
cl0630	ct0671	cn0698	pOP-CNH02801_EST_C_1_pSK_SK	590
cl0630	ct0671	cn0698	pOP-CNIP00952_EST_C_1_pSK_SK	672
cl0631	ct0672	cn0699	pOP-CNIP00664_EST_C_1_pSK_SK	521
cl0631	ct0672	cn0699	pOP-CNIP00957_EST_C_1_pSK_SK	488
cl0632	ct0673	cn0700	pOP-CNIP00470_EST_C_1_pSK_SK	428
cl0632	ct0673	cn0700	pOP-CNIP00961_EST_C_1_pSK_SK	781
cl0633	ct0674	cn0701	pOP-CNH01554_EST_C_1_pSK_SK	517
cl0633	ct0674	cn0701	pOP-CNI01485_EST_C_1_pSK_SK	564
cl0633	ct0674	cn0701	pOP-CNIP00430_EST_C_1_pSK_SK	448
cl0633	ct0674	cn0701	pOP-CNIP00499_EST_C_1_pSK_SK	486
cl0633	ct0674	cn0701	pOP-CNIP00965_EST_C_1_pSK_SK	684
cl0633	ct0674	cn0701	pOP-EO03265_EST_C_1_pSK_SK	424
cl0634	ct0675	cn0702	pOP-CNI01354_EST_C_1_pSK_SK	363
cl0634	ct0675	cn0702	pOP-CNIP00303_EST_C_1_pSK_SK	493
cl0634	ct0675	cn0702	pOP-CNIP00969_EST_C_1_pSK_SK	204
cl0635	ct0676	cn0703	pOP-CNH04503	793
cl0635	ct0677	cn0704	pOP-CNIP00978_EST_C_1_pSK_SK	680
cl0635	ct0678	cn0705	pOP-CNH02170_EST_C_1_pSK_SK	671
cl0636	ct0679	cn0706	pOP-CNIP00527_EST_C_1_pSK_SK	269
cl0636	ct0679	cn0706	pOP-CNIP00996_EST_C_1_pSK_SK	417
cl0637	ct0680	cn0707	pOP-CNH02800_EST_C_1_pSK_SK	624
cl0637	ct0680	cn0707	pOP-CNIP01007_EST_C_1_pSK_SK	581
cl0638	ct0681	cn0708	pOP-CNIP01010_EST_C_1_pSK_SK	137
cl0638	ct0681	cn0708	pOP-EO03164_EST_C_1_pSK_SK	438
cl0639	ct0682	cn0709	pOP-CNIP01011_EST_C_1_pSK_SK	405
cl0639	ct0682	cn0709	pOP-EO08090_EST_C_1_pSK_SK	474
cl0640	ct0683	cn0710	pOP-CNIP01013_EST_C_1_pSK_SK	649
cl0640	ct0684	cn0711	pOP-CNH01582_EST_C_1_pSK_SK	638
cl0641	ct0685	cn0712	pOP-CNH02856_EST_C_1_pSK_SK	630
cl0641	ct0685	cn0712	pOP-CNIP00096_EST_C_1_pSK_SK	691
cl0641	ct0685	cn0713	pOP-CNIP01020_EST_C_1_pSK_SK	622
cl0642	ct0686	cn0714	pOP-CNI01108_EST_C_1_pSK_SK	563
cl0642	ct0686	cn0715	pOP-EAP03631_EST_C_1_pBSK_SK	283
cl0642	ct0687	cn0716	pOP-CNI01985_EST_C_1_pSK_SK	282
cl0642	ct0687	cn0716	pOP-CNIP00634_EST_C_1_pSK_SK	398
cl0642	ct0687	cn0716	pOP-CNIP00734_EST_C_1_pSK_SK	505
cl0642	ct0687	cn0716	pOP-CNIP01022_EST_C_1_pSK_SK	494
cl0642	ct0688	cn0717	pOP-CEO01265	251
cl0642	ct0688	cn0717	pOP-CNI02232_EST_C_1_pSK_SK	669
cl0642	ct0688	cn0717	pOP-EAP00615_EST_C_1_pBSK_SK	275
cl0642	ct0688	cn0717	pOP-EAP03849_EST_C_1_pBSK_SK	589
cl0642	ct0688	cn0717	pOP-EO02781_EST_C_1_pSK_SK	430
cl0642	ct0688	cn0717	pOP-EO04922_EST_C_1_pSK_SK	521
cl0643	ct0689	cn0718	pOP-CNI01478_EST_C_1_pSK_SK	456
cl0643	ct0689	cn0718	pOP-CNIP00440_EST_C_1_pSK_SK	398
cl0643	ct0689	cn0718	pOP-CNIP01033_EST_C_1_pSK_SK	390
cl0644	ct0690	cn0719	pOP-CEO03169_EST_C_1_pSK_SK	316
cl0644	ct0690	cn0719	pOP-CNI01144_EST_C_1_pSK_SK	322
cl0644	ct0690	cn0719	pOP-CNI01912_EST_C_1_pSK_SK	574
cl0644	ct0690	cn0719	pOP-CNIP01042_EST_C_1_pSK_SK	700
cl0644	ct0690	cn0719	pOP-CNIP04089_EST_C_1_pSK_SK	127

cl0644	ct0690	cn0720	pOP-EAP01723_EST_C_1_pBSK_SK	542
cl0645	ct0691	cn0721	pOP-CNIP01055_EST_C_1_pSK_SK	370
cl0645	ct0691	cn0721	pOP-EO04297_EST_C_1_pSK_SK	327
cl0645	ct0691	cn0721	pOP-EO04298_EST_C_1_pSK_SK	131
cl0646	ct0692	cn0722	pOP-CNIP04002_EST_C_1_pSK_SK	610
cl0646	ct0692	cn0723	pOP-CEO01326	256
cl0646	ct0692	cn0723	pOP-CEO01387_EST_C_1_pSK_SK	387
cl0646	ct0692	cn0723	pOP-CEO02651_EST_C_1_pSK_SK	469
cl0646	ct0692	cn0723	pOP-CNI01551_EST_C_1_pSK_SK	394
cl0646	ct0692	cn0723	pOP-EO03550_EST_C_1_pSK_SK	410
cl0646	ct0692	cn0724	pOP-CEO00780_EST_C_1_pSK_SK	334
cl0647	ct0693	cn0725	pOP-CNH01884_EST_C_1_pSK_SK	529
cl0647	ct0693	cn0725	pOP-CNIP04008_EST_C_1_pSK_SK	254
cl0648	ct0694	cn0726	pOP-CNI01177_EST_C_1_pSK_SK	343
cl0648	ct0694	cn0726	pOP-CNIP04010_EST_C_1_pSK_SK	224
cl0649	ct0695	cn0727	pOP-CNIP04016_EST_C_1_pSK_SK	580
cl0649	ct0695	cn0727	pOP-EO05329_EST_C_1_pSK_SK	517
cl0650	ct0696	cn0728	pOP-CNI01236_EST_C_1_pSK_SK	284
cl0650	ct0696	cn0728	pOP-CNIP04031_EST_C_1_pSK_SK	191
cl0651	ct0697	cn0729	pOP-CNH04420	809
cl0651	ct0697	cn0729	pOP-CNIP04047_EST_C_1_pSK_SK	610
cl0652	ct0698	cn0730	pOP-CNH00690_EST_C_1_pSK_SK	531
cl0652	ct0698	cn0730	pOP-CNIP00306_EST_C_1_pSK_SK	413
cl0652	ct0698	cn0730	pOP-CNIP04052_EST_C_1_pSK_SK	454
cl0652	ct0698	cn0730	pOP-EN00526_EST_C_1_pSK_SK	296
cl0653	ct0699	cn0731	pOP-CNIP01074_EST_C_1_pSK_SK	306
cl0653	ct0699	cn0731	pOP-CNIP04055_EST_C_1_pSK_SK	376
cl0654	ct0700	cn0732	pOP-CNI01511_EST_C_1_pSK_SK	449
cl0654	ct0700	cn0732	pOP-CNIP04063_EST_C_1_pSK_SK	436
cl0655	ct0701	cn0733	pOP-CNHP00357_EST_C_1_pSK_SK	651
cl0655	ct0701	cn0733	pOP-CNIP04068_EST_C_1_pSK_SK	507
cl0656	ct0702	cn0734	pOP-CNIP04071_EST_C_1_pSK_SK	345
cl0656	ct0702	cn0734	pOP-CNIP04072_EST_C_1_pSK_SK	345
cl0657	ct0703	cn0735	pOP-CNIP04075_EST_C_1_pSK_SK	308
cl0657	ct0703	cn0735	pOP-EO04247_EST_C_1_pSK_SK	296
cl0658	ct0704	cn0736	pOP-CNIP04078_EST_C_1_pSK_SK	197
cl0658	ct0704	cn0736	pOP-EO08118_EST_C_1_pSK_SK	570
cl0659	ct0705	cn0737	pOP-CEO02693_EST_C_1_pSK_SK	338
cl0659	ct0705	cn0737	pOP-CNLP00003_EST_C_1_pSK_SK	309
cl0660	ct0706	cn0738	pOP-CNIP00218_EST_C_1_pSK_SK	497
cl0660	ct0706	cn0738	pOP-CNLP00015_EST_C_1_pSK_SK	347
cl0661	ct0707	cn0739	pOP-CNHP00214_EST_C_1_pSK_SK	370
cl0661	ct0707	cn0739	pOP-CNLP00016_EST_C_1_pSK_SK	562
cl0662	ct0708	cn0740	pOP-CNNP00002_EST_C_1_pBSK_SK	270
cl0662	ct0708	cn0740	pOP-CNNP00010_EST_C_1_pBSK_SK	270
cl0663	ct0709	cn0741	pOP-CNH03523_EST_C_1_pSK_SK	618
cl0663	ct0709	cn0741	pOP-CNNP00011_EST_C_1_pBSK_SK	300
cl0664	ct0710	cn0742	pOP-CNI01515_EST_C_1_pSK_SK	663
cl0664	ct0710	cn0742	pOP-CNNP00026_EST_C_1_pBSK_SK	491
cl0665	ct0711	cn0743	pOP-CNH00700_EST_C_1_pSK_SK	557
cl0665	ct0711	cn0743	pOP-CNH01395_EST_C_1_pSK_SK	708
cl0665	ct0711	cn0743	pOP-CNH02361_EST_C_1_pSK_SK	590
cl0665	ct0711	cn0743	pOP-CNH02437_EST_C_1_pSK_SK	542
cl0665	ct0711	cn0743	pOP-EAP00071_EST_C_1_pBSK_SK	590
cl0666	ct0712	cn0744	pOP-EAP00249_EST_C_1_pBSK_SK	163
cl0666	ct0712	cn0744	pOP-EAP00250_EST_C_1_pBSK_SK	163

cl0667	ct0713	cn0745	pOP-EO05735_EST_C_1_pSK_SK	519
cl0667	ct0713	cn0746	pOP-EO05926_EST_C_1_pSK_SK	467
cl0667	ct0714	cn0747	pOP-EO03832_EST_C_1_pSK_SK	530
cl0667	ct0714	cn0747	pOP-EO06211_EST_C_1_pSK_SK	626
cl0667	ct0714	cn0747	pOP-EO06492_EST_C_1_pSK_SK	786
cl0667	ct0714	cn0747	pOP-EO07315_EST_C_1_pSK_SK	708
cl0667	ct0715	cn0748	pOP-CEO00592_EST_C_1_pSK_SK	456
cl0667	ct0715	cn0748	pOP-CEO02665_EST_C_1_pSK_SK	524
cl0667	ct0715	cn0748	pOP-CNH02214_EST_C_1_pSK_SK	553
cl0667	ct0715	cn0748	pOP-CNH02896_EST_C_1_pSK_SK	552
cl0667	ct0715	cn0748	pOP-CNIP00924_EST_C_1_pSK_SK	520
cl0667	ct0715	cn0748	pOP-EAP02184_EST_C_1_pBSK_SK	240
cl0667	ct0715	cn0748	pOP-EAP03853_EST_C_1_pBSK_SK	365
cl0667	ct0715	cn0748	pOP-EO07835_EST_C_1_pSK_SK	759
cl0667	ct0716	cn0749	pOP-CEM00080_EST_C_1_pSK_SK	494
cl0667	ct0716	cn0749	pOP-CNH03091_EST_C_1_pSK_SK	525
cl0667	ct0716	cn0749	pOP-CNH03525_EST_C_1_pSK_SK	582
cl0667	ct0716	cn0749	pOP-CNHP00534_EST_C_1_pSK_SK	626
cl0667	ct0716	cn0749	pOP-EAP00255_EST_C_1_pBSK_SK	119
cl0667	ct0716	cn0749	pOP-EAP00328_EST_C_1_pBSK_SK	156
cl0667	ct0716	cn0749	pOP-EAP02298_EST_C_1_pBSK_SK	702
cl0667	ct0716	cn0750	pOP-CEO03527_EST_C_1_pSK_SK	412
cl0667	ct0717	cn0751	pOP-CNI01445_EST_C_1_pSK_SK	121
cl0667	ct0717	cn0751	pOP-CNIP00296_EST_C_1_pSK_SK	225
cl0667	ct0717	cn0751	pOP-EO02524_EST_C_1_pSK_SK	228
cl0667	ct0717	cn0751	pOP-EO02711_EST_C_1_pSK_SK	397
cl0667	ct0717	cn0751	pOP-EO04090_EST_C_1_pSK_SK	375
cl0667	ct0717	cn0751	pOP-EO04216_EST_C_1_pSK_SK	499
cl0667	ct0717	cn0751	pOP-EO06622_EST_C_1_pSK_SK	623
cl0667	ct0717	cn0751	pOP-EO06679_EST_C_1_pSK_SK	708
cl0667	ct0717	cn0751	pOP-EO06744_EST_C_1_pSK_SK	782
cl0667	ct0717	cn0751	pOP-EO06801_EST_C_1_pSK_SK	756
cl0667	ct0717	cn0751	pOP-EO07342_EST_C_1_pSK_SK	722
cl0667	ct0718	cn0752	pOP-CEO01390_EST_C_1_pSK_SK	446
cl0667	ct0718	cn0752	pOP-CEO01612_EST_C_1_pSK_SK	649
cl0667	ct0718	cn0752	pOP-CEO01650_EST_C_1_pSK_SK	205
cl0667	ct0718	cn0752	pOP-CEO01991_EST_C_1_pSK_SK	555
cl0667	ct0718	cn0752	pOP-CEO02870_EST_C_1_pSK_SK	480
cl0667	ct0718	cn0752	pOP-CEO03031_EST_C_1_pSK_SK	428
cl0667	ct0718	cn0752	pOP-CEO03122_EST_C_1_pSK_SK	353
cl0667	ct0718	cn0752	pOP-CEO03601_EST_C_1_pSK_SK	260
cl0667	ct0718	cn0752	pOP-CEO03680_EST_C_1_pSK_SK	281
cl0667	ct0718	cn0752	pOP-CNH00634_EST_C_1_pSK_SK	325
cl0667	ct0718	cn0752	pOP-CNH00815_EST_C_1_pSK_SK	418
cl0667	ct0718	cn0752	pOP-CNH01004_EST_C_1_pSK_SK	285
cl0667	ct0718	cn0752	pOP-CNH01094_EST_C_1_pSK_SK	411
cl0667	ct0718	cn0752	pOP-CNH01131_EST_C_1_pSK_SK	397
cl0667	ct0718	cn0752	pOP-CNH01157_EST_C_1_pSK_SK	391
cl0667	ct0718	cn0752	pOP-CNH01258_EST_C_1_pSK_SK	472
cl0667	ct0718	cn0752	pOP-CNH01463_EST_C_1_pSK_SK	585
cl0667	ct0718	cn0752	pOP-CNH01630_EST_C_1_pSK_SK	521
cl0667	ct0718	cn0752	pOP-CNH01715_EST_C_1_pSK_SK	418
cl0667	ct0718	cn0752	pOP-CNH01788_EST_C_1_pSK_SK	450
cl0667	ct0718	cn0752	pOP-CNH01811_EST_C_1_pSK_SK	484
cl0667	ct0718	cn0752	pOP-CNH01982_EST_C_1_pSK_SK	664
cl0667	ct0718	cn0752	pOP-CNH02106_EST_C_1_pSK_SK	647

cl0667	ct0718	cn0752	pOP-CNH02879_EST_C_1_pSK_SK	576
cl0667	ct0718	cn0752	pOP-CNH02931_EST_C_1_pSK_SK	560
cl0667	ct0718	cn0752	pOP-CNH02941_EST_C_1_pSK_SK	672
cl0667	ct0718	cn0752	pOP-CNH03348_EST_C_1_pSK_SK	636
cl0667	ct0718	cn0752	pOP-CNH03507_EST_C_1_pSK_SK	475
cl0667	ct0718	cn0752	pOP-CNH03594_EST_C_1_pSK_SK	347
cl0667	ct0718	cn0752	pOP-CNH03618_EST_C_1_pSK_SK	540
cl0667	ct0718	cn0752	pOP-CNH03726_EST_C_1_pSK_SK	399
cl0667	ct0718	cn0752	pOP-CNH04174	518
cl0667	ct0718	cn0752	pOP-CNH04239	524
cl0667	ct0718	cn0752	pOP-CNH04303	557
cl0667	ct0718	cn0752	pOP-CNH04327	666
cl0667	ct0718	cn0752	pOP-CNH04364	633
cl0667	ct0718	cn0752	pOP-CNH04366	571
cl0667	ct0718	cn0752	pOP-CNH05071_EST_C_1_pSK_SK	318
cl0667	ct0718	cn0752	pOP-CNI01555_EST_C_1_pSK_SK	547
cl0667	ct0718	cn0752	pOP-CNI01582_EST_C_1_pSK_SK	369
cl0667	ct0718	cn0752	pOP-CNI01863_EST_C_1_pSK_SK	218
cl0667	ct0718	cn0752	pOP-CNI02074_EST_C_1_pSK_SK	464
cl0667	ct0718	cn0752	pOP-CNI02094_EST_C_1_pSK_SK	427
cl0667	ct0718	cn0752	pOP-CNI02112_EST_C_1_pSK_SK	490
cl0667	ct0718	cn0752	pOP-CNI02140_EST_C_1_pSK_SK	245
cl0667	ct0718	cn0752	pOP-EAP00332_EST_C_1_pBSK_SK	211
cl0667	ct0718	cn0752	pOP-EAP01827_EST_C_1_pBSK_SK	388
cl0667	ct0718	cn0752	pOP-EAP03358_EST_C_1_pBSK_SK	376
cl0667	ct0718	cn0752	pOP-EN00128_EST_C_1_pSK_SK	473
cl0667	ct0718	cn0752	pOP-EN00133_EST_C_1_pSK_SK	592
cl0667	ct0718	cn0752	pOP-EN00215_EST_C_1_pSK_SK	457
cl0667	ct0718	cn0752	pOP-EN00272_EST_C_1_pSK_SK	561
cl0667	ct0718	cn0752	pOP-EN00466_EST_C_1_pSK_SK	514
cl0667	ct0718	cn0752	pOP-EN00473_EST_C_1_pSK_SK	517
cl0667	ct0718	cn0752	pOP-EN00524_EST_C_1_pSK_SK	470
cl0667	ct0718	cn0752	pOP-EN00593_EST_C_1_pSK_SK	411
cl0667	ct0718	cn0752	pOP-EN00602_EST_C_1_pSK_SK	538
cl0667	ct0718	cn0752	pOP-EN00670_EST_C_1_pSK_SK	374
cl0667	ct0718	cn0752	pOP-EN00686_EST_C_1_pSK_SK	485
cl0667	ct0718	cn0752	pOP-EN00689_EST_C_1_pSK_SK	536
cl0667	ct0718	cn0752	pOP-EN00818_EST_C_1_pSK_SK	518
cl0667	ct0718	cn0752	pOP-EN00826_EST_C_1_pSK_SK	496
cl0667	ct0718	cn0752	pOP-EN00858_EST_C_1_pSK_SK	539
cl0667	ct0718	cn0752	pOP-EN00897_EST_C_1_pSK_SK	523
cl0667	ct0718	cn0752	pOP-EO02106_EST_C_1_pSK_SK	355
cl0667	ct0718	cn0752	pOP-EO02778_EST_C_1_pSK_SK	409
cl0667	ct0718	cn0752	pOP-EO03598_EST_C_1_pSK_SK	448
cl0667	ct0718	cn0752	pOP-EO04215_EST_C_1_pSK_SK	525
cl0667	ct0718	cn0752	pOP-EO04332_EST_C_1_pSK_SK	346
cl0667	ct0718	cn0752	pOP-EO04351_EST_C_1_pSK_SK	524
cl0667	ct0718	cn0752	pOP-EO04487_EST_C_1_pSK_SK	403
cl0667	ct0718	cn0752	pOP-EO04627_EST_C_1_pSK_SK	536
cl0667	ct0718	cn0752	pOP-EO04829_EST_C_1_pSK_SK	561
cl0667	ct0718	cn0752	pOP-EO05408_EST_C_1_pSK_SK	526
cl0667	ct0718	cn0752	pOP-EO05481_EST_C_1_pSK_SK	524
cl0667	ct0718	cn0752	pOP-EO05483_EST_C_1_pSK_SK	503
cl0667	ct0718	cn0752	pOP-EO05615_EST_C_1_pSK_SK	468
cl0667	ct0718	cn0752	pOP-EO05687_EST_C_1_pSK_SK	473
cl0667	ct0718	cn0752	pOP-EO05800_EST_C_1_pSK_SK	381

cl0667	ct0718	cn0752	pOP-EO05873_EST_C_1_pSK_SK	603
cl0667	ct0718	cn0752	pOP-EO05915_EST_C_1_pSK_SK	572
cl0667	ct0718	cn0752	pOP-EO05991_EST_C_1_pSK_SK	580
cl0667	ct0718	cn0752	pOP-EO06059_EST_C_1_pSK_SK	646
cl0667	ct0718	cn0752	pOP-EO06092_EST_C_1_pSK_SK	529
cl0667	ct0718	cn0752	pOP-EO06434_EST_C_1_pSK_SK	769
cl0667	ct0718	cn0752	pOP-EO06442_EST_C_1_pSK_SK	738
cl0667	ct0718	cn0752	pOP-EO06506_EST_C_1_pSK_SK	715
cl0667	ct0718	cn0752	pOP-EO06542_EST_C_1_pSK_SK	736
cl0667	ct0718	cn0752	pOP-EO06547_EST_C_1_pSK_SK	822
cl0667	ct0718	cn0752	pOP-EO06592_EST_C_1_pSK_SK	802
cl0667	ct0718	cn0752	pOP-EO06644_EST_C_1_pSK_SK	684
cl0667	ct0718	cn0752	pOP-EO06663_EST_C_1_pSK_SK	702
cl0667	ct0718	cn0752	pOP-EO06671_EST_C_1_pSK_SK	763
cl0667	ct0718	cn0752	pOP-EO06752_EST_C_1_pSK_SK	806
cl0667	ct0718	cn0752	pOP-EO06760_EST_C_1_pSK_SK	834
cl0667	ct0718	cn0752	pOP-EO06779_EST_C_1_pSK_SK	847
cl0667	ct0718	cn0752	pOP-EO06823_EST_C_1_pSK_SK	412
cl0667	ct0718	cn0752	pOP-EO06865_EST_C_1_pSK_SK	807
cl0667	ct0718	cn0752	pOP-EO06884_EST_C_1_pSK_SK	758
cl0667	ct0718	cn0752	pOP-EO06979_EST_C_1_pSK_SK	754
cl0667	ct0718	cn0752	pOP-EO07164_EST_C_1_pSK_SK	629
cl0667	ct0718	cn0752	pOP-EO07544_EST_C_1_pSK_SK	787
cl0667	ct0718	cn0752	pOP-EO07669_EST_C_1_pSK_SK	816
cl0667	ct0718	cn0752	pOP-EO07873_EST_C_1_pSK_SK	641
cl0667	ct0718	cn0752	pOP-EO07937_EST_C_1_pSK_SK	675
cl0667	ct0718	cn0752	pOP-EO08383_EST_C_1_pSK_SK	517
cl0667	ct0718	cn0752	pOP-EO08441_EST_C_1_pSK_SK	298
cl0667	ct0718	cn0752	pOP-EO08491_EST_C_1_pSK_SK	145
cl0667	ct0718	cn0752	pOP-EO08532_EST_C_1_pSK_SK	325
cl0667	ct0718	cn0753	pOP-EO06466_EST_C_1_pSK_SK	705
cl0668	ct0719	cn0754	pOP-CEO01605_EST_C_1_pSK_SK	516
cl0668	ct0719	cn0754	pOP-CNH00713_EST_C_1_pSK_SK	596
cl0668	ct0719	cn0754	pOP-CNH04759_EST_C_1_pSK_SK	559
cl0668	ct0719	cn0754	pOP-CNHP00374_EST_C_1_pSK_SK	463
cl0668	ct0719	cn0754	pOP-CNI01376_EST_C_1_pSK_SK	559
cl0668	ct0719	cn0754	pOP-CNNP00016_EST_C_1_pBSK_SK	669
cl0668	ct0719	cn0754	pOP-EAP00275_EST_C_1_pBSK_SK	531
cl0668	ct0719	cn0754	pOP-EN00516_EST_C_1_pSK_SK	462
cl0668	ct0719	cn0754	pOP-EO02350_EST_C_1_pSK_SK	624
cl0668	ct0719	cn0754	pOP-EO03246_EST_C_1_pSK_SK	472
cl0668	ct0719	cn0754	pOP-EO05807_EST_C_1_pSK_SK	694
cl0669	ct0720	cn0755	pOP-CNH00797_EST_C_1_pSK_SK	531
cl0669	ct0720	cn0755	pOP-EAP00331_EST_C_1_pBSK_SK	470
cl0670	ct0721	cn0756	pOP-EAP00350_EST_C_1_pBSK_SK	498
cl0670	ct0721	cn0756	pOP-EAP00351_EST_C_1_pBSK_SK	217
cl0671	ct0722	cn0757	pOP-EAP00373_EST_C_1_pBSK_SK	182
cl0671	ct0722	cn0757	pOP-EAP00374_EST_C_1_pBSK_SK	405
cl0672	ct0723	cn0758	pOP-CNH00955_EST_C_1_pSK_SK	634
cl0672	ct0723	cn0758	pOP-EAP00375_EST_C_1_pBSK_SK	480
cl0673	ct0724	cn0759	pOP-CNH01232_EST_C_1_pSK_SK	528
cl0673	ct0724	cn0759	pOP-CNH04261	404
cl0673	ct0724	cn0759	pOP-CNH04283	500
cl0673	ct0724	cn0759	pOP-EAP00416_EST_C_1_pBSK_SK	259
cl0674	ct0725	cn0760	pOP-EAP00481_EST_C_1_pBSK_SK	166
cl0674	ct0725	cn0760	pOP-EAP00500_EST_C_1_pBSK_SK	541

cl0675	ct0726	cn0761	pOP-CNH02409_EST_C_1_pSK_SK	331
cl0675	ct0726	cn0761	pOP-EAP00507_EST_C_1_pBSK_SK	485
cl0676	ct0727	cn0762	pOP-CEO02540_EST_C_1_pSK_SK	359
cl0676	ct0727	cn0762	pOP-CNH02083_EST_C_1_pSK_SK	491
cl0676	ct0727	cn0762	pOP-CNH05070_EST_C_1_pSK_SK	653
cl0676	ct0727	cn0762	pOP-EAP00166_EST_C_1_pBSK_SK	620
cl0676	ct0727	cn0762	pOP-EAP00509_EST_C_1_pBSK_SK	669
cl0677	ct0728	cn0763	pOP-CNH02062_EST_C_1_pSK_SK	447
cl0677	ct0728	cn0763	pOP-CNH02255_EST_C_1_pSK_SK	705
cl0677	ct0728	cn0763	pOP-EAP00521_EST_C_1_pBSK_SK	581
cl0678	ct0729	cn0764	pOP-CNH02717_EST_C_1_pSK_SK	613
cl0678	ct0729	cn0764	pOP-EAP00534_EST_C_1_pBSK_SK	549
cl0678	ct0729	cn0764	pOP-EO06530_EST_C_1_pSK_SK	617
cl0679	ct0730	cn0765	pOP-CNIP00538_EST_C_1_pSK_SK	325
cl0679	ct0730	cn0765	pOP-EAP00537_EST_C_1_pBSK_SK	409
cl0679	ct0730	cn0766	pOP-CEM00163_EST_C_1_pSK_SK	277
cl0680	ct0731	cn0767	pOP-CNH00942_EST_C_1_pSK_SK	692
cl0680	ct0731	cn0767	pOP-EAP00539_EST_C_1_pBSK_SK	629
cl0681	ct0732	cn0768	pOP-CNH01690_EST_C_1_pSK_SK	748
cl0681	ct0732	cn0768	pOP-CNH01902_EST_C_1_pSK_SK	705
cl0681	ct0732	cn0769	pOP-EAP00540_EST_C_1_pBSK_SK	585
cl0682	ct0733	cn0770	pOP-CEO00635_EST_C_1_pSK_SK	416
cl0682	ct0733	cn0770	pOP-EAP00559_EST_C_1_pBSK_SK	582
cl0682	ct0733	cn0771	pOP-EAP00560_EST_C_1_pBSK_SK	590
cl0682	ct0734	cn0772	pOP-CNH04474	528
cl0682	ct0734	cn0772	pOP-EO03614_EST_C_1_pSK_SK	465
cl0682	ct0734	cn0772	pOP-EO05911_EST_C_1_pSK_SK	589
cl0682	ct0735	cn0773	pOP-CEO00678_EST_C_1_pSK_SK	381
cl0682	ct0735	cn0773	pOP-CEO00695_EST_C_1_pSK_SK	318
cl0682	ct0735	cn0773	pOP-CEO01398_EST_C_1_pSK_SK	272
cl0682	ct0735	cn0773	pOP-CEO01459_EST_C_1_pSK_SK	422
cl0682	ct0735	cn0773	pOP-CEO02277_EST_C_1_pSK_SK	404
cl0682	ct0735	cn0773	pOP-CEO02518_EST_C_1_pSK_SK	149
cl0682	ct0735	cn0773	pOP-CNH02706_EST_C_1_pSK_SK	287
cl0682	ct0735	cn0773	pOP-CNH03238_EST_C_1_pSK_SK	278
cl0682	ct0735	cn0773	pOP-CNIP00933_EST_C_1_pSK_SK	559
cl0682	ct0735	cn0773	pOP-CNIP01029_EST_C_1_pSK_SK	531
cl0682	ct0735	cn0773	pOP-CNLP00009_EST_C_1_pSK_SK	446
cl0682	ct0735	cn0773	pOP-EO02242_EST_C_1_pSK_SK	625
cl0682	ct0735	cn0773	pOP-EO02739_EST_C_1_pSK_SK	446
cl0682	ct0735	cn0773	pOP-EO03348_EST_C_1_pSK_SK	460
cl0682	ct0735	cn0773	pOP-EO04062_EST_C_1_pSK_SK	619
cl0682	ct0735	cn0773	pOP-EO05725_EST_C_1_pSK_SK	463
cl0682	ct0735	cn0773	pOP-EO05831_EST_C_1_pSK_SK	600
cl0682	ct0735	cn0773	pOP-EO05857_EST_C_1_pSK_SK	593
cl0682	ct0735	cn0773	pOP-EO06007_EST_C_1_pSK_SK	612
cl0682	ct0735	cn0773	pOP-EO06921_EST_C_1_pSK_SK	554
cl0682	ct0735	cn0773	pOP-EO07749_EST_C_1_pSK_SK	719
cl0682	ct0735	cn0773	pOP-EO08076_EST_C_1_pSK_SK	575
cl0683	ct0736	cn0774	pOP-EAP00561_EST_C_1_pBSK_SK	120
cl0683	ct0736	cn0774	pOP-EAP00562_EST_C_1_pBSK_SK	357
cl0684	ct0737	cn0775	pOP-EAP00566_EST_C_1_pBSK_SK	148
cl0684	ct0737	cn0775	pOP-EAP00567_EST_C_1_pBSK_SK	493
cl0685	ct0738	cn0776	pOP-CNIP00583_EST_C_1_pSK_SK	222
cl0685	ct0738	cn0776	pOP-EAP00569_EST_C_1_pBSK_SK	493
cl0686	ct0739	cn0777	pOP-EAP00564_EST_C_1_pBSK_SK	224

cl0686	ct0739	cn0777	pOP-EAP00571_EST_C_1_pBSK_SK	223
cl0687	ct0740	cn0778	pOP-EAP00580_EST_C_1_pBSK_SK	564
cl0687	ct0740	cn0778	pOP-EAP00583_EST_C_1_pBSK_SK	587
cl0688	ct0741	cn0779	pOP-EAP00581_EST_C_1_pBSK_SK	598
cl0688	ct0741	cn0779	pOP-EAP00584_EST_C_1_pBSK_SK	554
cl0689	ct0742	cn0780	pOP-EAP00595_EST_C_1_pBSK_SK	613
cl0689	ct0742	cn0780	pOP-EO04896_EST_C_1_pSK_SK	475
cl0690	ct0743	cn0781	pOP-CNH00862_EST_C_1_pSK_SK	668
cl0690	ct0743	cn0781	pOP-CNHP00469_EST_C_1_pSK_SK	672
cl0690	ct0743	cn0781	pOP-EAP00598_EST_C_1_pBSK_SK	384
cl0691	ct0744	cn0782	pOP-CNI01778_EST_C_1_pSK_SK	295
cl0691	ct0744	cn0782	pOP-EAP00613_EST_C_1_pBSK_SK	583
cl0692	ct0745	cn0783	pOP-CNH01640_EST_C_1_pSK_SK	644
cl0692	ct0745	cn0783	pOP-CNH02968_EST_C_1_pSK_SK	647
cl0692	ct0745	cn0783	pOP-EAP00617_EST_C_1_pBSK_SK	283
cl0693	ct0746	cn0784	pOP-CNIP00190_EST_C_1_pSK_SK	359
cl0693	ct0746	cn0784	pOP-EAP00620_EST_C_1_pBSK_SK	649
cl0693	ct0746	cn0784	pOP-EO05587_EST_C_1_pSK_SK	453
cl0693	ct0746	cn0785	pOP-CNH00908_EST_C_1_pSK_SK	485
cl0694	ct0747	cn0786	pOP-EAP00622_EST_C_1_pBSK_SK	576
cl0694	ct0747	cn0786	pOP-EAP00623_EST_C_1_pBSK_SK	657
cl0695	ct0748	cn0787	pOP-CNI01539_EST_C_1_pSK_SK	524
cl0695	ct0748	cn0787	pOP-EAP00632_EST_C_1_pBSK_SK	576
cl0696	ct0749	cn0788	pOP-CNH04901_EST_C_1_pSK_SK	721
cl0696	ct0749	cn0788	pOP-EAP00645_EST_C_1_pBSK_SK	606
cl0696	ct0749	cn0789	pOP-CNHP00178_EST_C_1_pSK_SK	450
cl0697	ct0750	cn0790	pOP-EAP00649_EST_C_1_pBSK_SK	573
cl0697	ct0750	cn0790	pOP-EAP00650_EST_C_1_pBSK_SK	567
cl0698	ct0751	cn0791	pOP-CEO02640_EST_C_1_pSK_SK	524
cl0698	ct0751	cn0791	pOP-CNI02087_EST_C_1_pSK_SK	618
cl0698	ct0751	cn0791	pOP-CNIP00374_EST_C_1_pSK_SK	258
cl0698	ct0751	cn0791	pOP-EAP00663_EST_C_1_pBSK_SK	202
cl0698	ct0751	cn0791	pOP-EAP00664_EST_C_1_pBSK_SK	169
cl0698	ct0751	cn0791	pOP-EO02535_EST_C_1_pSK_SK	529
cl0698	ct0751	cn0791	pOP-EO04292_EST_C_1_pSK_SK	418
cl0698	ct0751	cn0791	pOP-EO04518_EST_C_1_pSK_SK	533
cl0699	ct0752	cn0792	pOP-CNH01939_EST_C_1_pSK_SK	670
cl0699	ct0752	cn0793	pOP-CNH04454	665
cl0699	ct0752	cn0793	pOP-EAP00685_EST_C_1_pBSK_SK	132
cl0700	ct0753	cn0794	pOP-CNH04435	713
cl0700	ct0753	cn0794	pOP-CNI01313_EST_C_1_pSK_SK	181
cl0700	ct0753	cn0794	pOP-CNI01691_EST_C_1_pSK_SK	415
cl0700	ct0753	cn0794	pOP-EAP00689_EST_C_1_pBSK_SK	266
cl0700	ct0753	cn0794	pOP-EO03136_EST_C_1_pSK_SK	348
cl0701	ct0754	cn0795	pOP-CNI01287_EST_C_1_pSK_SK	647
cl0701	ct0754	cn0795	pOP-EAP00692_EST_C_1_pBSK_SK	320
cl0702	ct0755	cn0796	pOP-CNIP00109_EST_C_1_pSK_SK	437
cl0702	ct0755	cn0796	pOP-CNIP00958_EST_C_1_pSK_SK	310
cl0702	ct0755	cn0796	pOP-EAP00702_EST_C_1_pBSK_SK	658
cl0703	ct0756	cn0797	pOP-EAP00708_EST_C_1_pBSK_SK	559
cl0703	ct0756	cn0797	pOP-EO06156_EST_C_1_pSK_SK	535
cl0704	ct0757	cn0798	pOP-EAP00732_EST_C_1_pBSK_SK	650
cl0704	ct0757	cn0798	pOP-EAP00733_EST_C_1_pBSK_SK	172
cl0705	ct0758	cn0799	pOP-EAP00744_EST_C_1_pBSK_SK	632
cl0705	ct0759	cn0800	pOP-CNH01001_EST_C_1_pSK_SK	310
cl0705	ct0759	cn0800	pOP-CNH02702_EST_C_1_pSK_SK	657

cl0706	ct0760	cn0801	pOP-CNHP00464_EST_C_1_pSK_SK	752
cl0706	ct0760	cn0801	pOP-EAP00760_EST_C_1_pBSK_SK	582
cl0706	ct0760	cn0801	pOP-EAP00766_EST_C_1_pBSK_SK	139
cl0707	ct0761	cn0802	pOP-EAP00780_EST_C_1_pBSK_SK	511
cl0707	ct0761	cn0802	pOP-EAP00781_EST_C_1_pBSK_SK	549
cl0708	ct0762	cn0803	pOP-EAP00800_EST_C_1_pBSK_SK	457
cl0708	ct0762	cn0803	pOP-EO08194_EST_C_1_pSK_SK	562
cl0709	ct0763	cn0804	pOP-CNH02624_EST_C_1_pSK_SK	587
cl0709	ct0763	cn0804	pOP-EAP00593_EST_C_1_pBSK_SK	637
cl0709	ct0763	cn0804	pOP-EAP00810_EST_C_1_pBSK_SK	421
cl0710	ct0764	cn0805	pOP-EAP00856_EST_C_1_pBSK_SK	540
cl0710	ct0765	cn0806	pOP-CNH01693_EST_C_1_pSK_SK	616
cl0710	ct0765	cn0806	pOP-CNH02934_EST_C_1_pSK_SK	643
cl0710	ct0765	cn0806	pOP-CNHP00368_EST_C_1_pSK_SK	665
cl0710	ct0765	cn0806	pOP-CNI01247_EST_C_1_pSK_SK	264
cl0711	ct0766	cn0807	pOP-EAP00857_EST_C_1_pBSK_SK	420
cl0711	ct0766	cn0807	pOP-EAP00858_EST_C_1_pBSK_SK	432
cl0712	ct0767	cn0808	pOP-EAP00865_EST_C_1_pBSK_SK	444
cl0712	ct0767	cn0808	pOP-EAP00867_EST_C_1_pBSK_SK	658
cl0713	ct0768	cn0809	pOP-CNH00669_EST_C_1_pSK_SK	502
cl0713	ct0768	cn0809	pOP-CNH00790_EST_C_1_pSK_SK	433
cl0713	ct0768	cn0809	pOP-CNH00923_EST_C_1_pSK_SK	587
cl0713	ct0768	cn0809	pOP-CNH01275_EST_C_1_pSK_SK	519
cl0713	ct0768	cn0809	pOP-CNH01946_EST_C_1_pSK_SK	626
cl0713	ct0768	cn0809	pOP-CNH02342_EST_C_1_pSK_SK	648
cl0713	ct0768	cn0809	pOP-CNH02539_EST_C_1_pSK_SK	519
cl0713	ct0768	cn0809	pOP-CNH02802_EST_C_1_pSK_SK	593
cl0713	ct0768	cn0809	pOP-CNH03802_EST_C_1_pSK_SK	629
cl0713	ct0768	cn0809	pOP-CNH05021_EST_C_1_pSK_SK	657
cl0713	ct0768	cn0809	pOP-CNHP00097_EST_C_1_pSK_SK	398
cl0713	ct0768	cn0809	pOP-EAP00888_EST_C_1_pBSK_SK	222
cl0713	ct0768	cn0809	pOP-EO05272_EST_C_1_pSK_SK	559
cl0714	ct0769	cn0810	pOP-CNIP00868_EST_C_1_pSK_SK	502
cl0714	ct0769	cn0810	pOP-EAP00905_EST_C_1_pBSK_SK	461
cl0714	ct0769	cn0810	pOP-EO06161_EST_C_1_pSK_SK	314
cl0715	ct0770	cn0811	pOP-CEO01302	474
cl0715	ct0770	cn0811	pOP-EAP00915_EST_C_1_pBSK_SK	412
cl0716	ct0771	cn0812	pOP-EAP00894_EST_C_1_pBSK_SK	219
cl0716	ct0771	cn0812	pOP-EAP00933_EST_C_1_pBSK_SK	222
cl0717	ct0772	cn0813	pOP-CNH02890_EST_C_1_pSK_SK	574
cl0717	ct0772	cn0813	pOP-CNHP00122_EST_C_1_pSK_SK	506
cl0717	ct0772	cn0813	pOP-EAP00937_EST_C_1_pBSK_SK	630
cl0718	ct0773	cn0814	pOP-CNIP00956_EST_C_1_pSK_SK	424
cl0718	ct0773	cn0814	pOP-EAP00946_EST_C_1_pBSK_SK	245
cl0719	ct0774	cn0815	pOP-CNH04977_EST_C_1_pSK_SK	740
cl0719	ct0774	cn0815	pOP-EAP01016_EST_C_1_pBSK_SK	605
cl0719	ct0774	cn0815	pOP-EO05849_EST_C_1_pSK_SK	412
cl0720	ct0775	cn0816	pOP-CNI02072_EST_C_1_pSK_SK	185
cl0720	ct0775	cn0816	pOP-EAP01086_EST_C_1_pBSK_SK	139
cl0721	ct0776	cn0817	pOP-CNIP00625_EST_C_1_pSK_SK	393
cl0721	ct0776	cn0817	pOP-EAP01118_EST_C_1_pBSK_SK	593
cl0721	ct0776	cn0817	pOP-EO06108_EST_C_1_pSK_SK	611
cl0722	ct0777	cn0818	pOP-CNI01570_EST_C_1_pSK_SK	242
cl0722	ct0777	cn0818	pOP-EAP01133_EST_C_1_pBSK_SK	332
cl0723	ct0778	cn0819	pOP-CNH01848_EST_C_1_pSK_SK	388
cl0723	ct0778	cn0819	pOP-CNH02338_EST_C_1_pSK_SK	388

cl0723	ct0778	cn0819	pOP-EAP01140_EST_C_1_pBSK_SK	172
cl0724	ct0779	cn0820	pOP-CNH01416_EST_C_1_pSK_SK	571
cl0724	ct0779	cn0820	pOP-CNH02171_EST_C_1_pSK_SK	562
cl0724	ct0779	cn0820	pOP-CNI02214_EST_C_1_pSK_SK	267
cl0724	ct0779	cn0820	pOP-EAP01142_EST_C_1_pBSK_SK	514
cl0725	ct0780	cn0821	pOP-EAP01145_EST_C_1_pBSK_SK	169
cl0725	ct0780	cn0821	pOP-EO03247_EST_C_1_pSK_SK	475
cl0725	ct0780	cn0821	pOP-EO08186_EST_C_1_pSK_SK	504
cl0726	ct0781	cn0822	pOP-EAP00586_EST_C_1_pBSK_SK	325
cl0726	ct0781	cn0822	pOP-EAP01150_EST_C_1_pBSK_SK	525
cl0726	ct0781	cn0822	pOP-EO03175_EST_C_1_pSK_SK	327
cl0727	ct0782	cn0823	pOP-EAP00913_EST_C_1_pBSK_SK	131
cl0727	ct0782	cn0823	pOP-EAP01147_EST_C_1_pBSK_SK	111
cl0727	ct0782	cn0823	pOP-EAP01156_EST_C_1_pBSK_SK	123
cl0728	ct0783	cn0824	pOP-CAP00402_EST_C_1_pBSK_SK	641
cl0728	ct0783	cn0824	pOP-CEO02400_EST_C_1_pSK_SK	228
cl0728	ct0783	cn0824	pOP-EAP01570_EST_C_1_pBSK_SK	478
cl0728	ct0783	cn0824	pOP-EAP01875_EST_C_1_pBSK_SK	600
cl0728	ct0783	cn0824	pOP-EAP02731_EST_C_1_pBSK_SK	532
cl0728	ct0783	cn0824	pOP-EO02352_EST_C_1_pSK_SK	479
cl0728	ct0783	cn0824	pOP-EO04505_EST_C_1_pSK_SK	465
cl0728	ct0783	cn0824	pOP-EO04964_EST_C_1_pSK_SK	519
cl0728	ct0783	cn0824	pOP-EO05008_EST_C_1_pSK_SK	501
cl0728	ct0783	cn0825	pOP-EAP00174_EST_C_1_pBSK_SK	621
cl0728	ct0783	cn0825	pOP-EO02234_EST_C_1_pSK_SK	635
cl0728	ct0783	cn0826	pOP-CNH01520_EST_C_1_pSK_SK	537
cl0728	ct0783	cn0827	pOP-EAP01172_EST_C_1_pBSK_SK	349
cl0729	ct0784	cn0828	pOP-CEO02406_EST_C_1_pSK_SK	569
cl0729	ct0784	cn0828	pOP-CNH02954_EST_C_1_pSK_SK	584
cl0729	ct0784	cn0828	pOP-CNI01855_EST_C_1_pSK_SK	495
cl0729	ct0784	cn0828	pOP-EAP00140_EST_C_1_pBSK_SK	411
cl0729	ct0784	cn0828	pOP-EAP00654_EST_C_1_pBSK_SK	310
cl0729	ct0784	cn0828	pOP-EAP01075_EST_C_1_pBSK_SK	584
cl0729	ct0784	cn0828	pOP-EAP01177_EST_C_1_pBSK_SK	123
cl0729	ct0784	cn0828	pOP-EAP01680_EST_C_1_pBSK_SK	434
cl0729	ct0784	cn0828	pOP-EAP02891_EST_C_1_pBSK_SK	587
cl0729	ct0784	cn0828	pOP-EO02424_EST_C_1_pSK_SK	463
cl0729	ct0784	cn0828	pOP-EO02434_EST_C_1_pSK_SK	463
cl0730	ct0785	cn0829	pOP-EAP00759_EST_C_1_pBSK_SK	389
cl0730	ct0785	cn0829	pOP-EAP00767_EST_C_1_pBSK_SK	251
cl0730	ct0785	cn0829	pOP-EAP01178_EST_C_1_pBSK_SK	370
cl0731	ct0786	cn0830	pOP-CEO02860_EST_C_1_pSK_SK	257
cl0731	ct0786	cn0830	pOP-EAP01194_EST_C_1_pBSK_SK	559
cl0732	ct0787	cn0831	pOP-EAP00541_EST_C_1_pBSK_SK	550
cl0732	ct0787	cn0831	pOP-EAP01196_EST_C_1_pBSK_SK	234
cl0733	ct0788	cn0832	pOP-CNI01783_EST_C_1_pSK_SK	415
cl0733	ct0788	cn0832	pOP-EAP01208_EST_C_1_pBSK_SK	207
cl0734	ct0789	cn0833	pOP-CEO01055_EST_C_1_pSK_SK	415
cl0734	ct0789	cn0833	pOP-CEO01167_EST_C_1_pSK_SK	580
cl0734	ct0789	cn0833	pOP-CEO01836_EST_C_1_pSK_SK	262
cl0734	ct0789	cn0833	pOP-CEO02273_EST_C_1_pSK_SK	294
cl0734	ct0789	cn0833	pOP-CNIP00540_EST_C_1_pSK_SK	298
cl0734	ct0789	cn0833	pOP-CNIP00917_EST_C_1_pSK_SK	129
cl0734	ct0789	cn0833	pOP-EAP00921_EST_C_1_pBSK_SK	130
cl0734	ct0789	cn0833	pOP-EAP00942_EST_C_1_pBSK_SK	111
cl0734	ct0789	cn0833	pOP-EAP01139_EST_C_1_pBSK_SK	654

cl0734	ct0789	cn0833	pOP-EAP01223_EST_C_1_pBSK_SK	116
cl0734	ct0789	cn0833	pOP-EAP01247_EST_C_1_pBSK_SK	245
cl0734	ct0789	cn0833	pOP-EAP01275_EST_C_1_pBSK_SK	314
cl0734	ct0789	cn0833	pOP-EAP01419_EST_C_1_pBSK_SK	266
cl0734	ct0789	cn0833	pOP-EAP03585_EST_C_1_pBSK_SK	259
cl0735	ct0790	cn0834	pOP-CNI01495_EST_C_1_pSK_SK	463
cl0735	ct0790	cn0834	pOP-CNI01556_EST_C_1_pSK_SK	463
cl0735	ct0790	cn0834	pOP-EAP01227_EST_C_1_pBSK_SK	537
cl0736	ct0791	cn0835	pOP-CNHP00439_EST_C_1_pSK_SK	312
cl0736	ct0791	cn0835	pOP-EAP01228_EST_C_1_pBSK_SK	566
cl0737	ct0792	cn0836	pOP-EAP01285_EST_C_1_pBSK_SK	231
cl0737	ct0792	cn0836	pOP-EAP01286_EST_C_1_pBSK_SK	260
cl0738	ct0793	cn0837	pOP-CEO02987_EST_C_1_pSK_SK	459
cl0738	ct0793	cn0837	pOP-EAP01290_EST_C_1_pBSK_SK	141
cl0738	ct0793	cn0838	pOP-CEO02568_EST_C_1_pSK_SK	314
cl0738	ct0794	cn0839	pOP-CEM00048_EST_C_1_pSK_SK	113
cl0738	ct0794	cn0839	pOP-CEO01350	284
cl0738	ct0794	cn0839	pOP-CEO03610_EST_C_1_pSK_SK	390
cl0738	ct0794	cn0839	pOP-CNI01622_EST_C_1_pSK_SK	400
cl0738	ct0794	cn0839	pOP-CNIP01026_EST_C_1_pSK_SK	280
cl0738	ct0794	cn0839	pOP-CNIP01027_EST_C_1_pSK_SK	281
cl0738	ct0794	cn0839	pOP-EAP00546_EST_C_1_pBSK_SK	583
cl0738	ct0794	cn0839	pOP-EAP01068_EST_C_1_pBSK_SK	137
cl0738	ct0794	cn0839	pOP-EAP01166_EST_C_1_pBSK_SK	328
cl0738	ct0794	cn0839	pOP-EAP01370_EST_C_1_pBSK_SK	329
cl0738	ct0794	cn0839	pOP-EAP03615_EST_C_1_pBSK_SK	263
cl0738	ct0794	cn0839	pOP-EAP03659_EST_C_1_pBSK_SK	457
cl0738	ct0794	cn0840	pOP-CEO02122_EST_C_1_pSK_SK	530
cl0738	ct0794	cn0840	pOP-CEO03495_EST_C_1_pSK_SK	241
cl0739	ct0795	cn0841	pOP-CNH00723_EST_C_1_pSK_SK	579
cl0739	ct0795	cn0841	pOP-CNH01389_EST_C_1_pSK_SK	721
cl0739	ct0795	cn0841	pOP-CNH02974_EST_C_1_pSK_SK	466
cl0739	ct0795	cn0841	pOP-CNI01700_EST_C_1_pSK_SK	544
cl0739	ct0795	cn0841	pOP-EAP01303_EST_C_1_pBSK_SK	216
cl0740	ct0796	cn0842	pOP-CNH00766_EST_C_1_pSK_SK	518
cl0740	ct0796	cn0842	pOP-EAP01308_EST_C_1_pBSK_SK	363
cl0741	ct0797	cn0843	pOP-EAP01323_EST_C_1_pBSK_SK	299
cl0741	ct0797	cn0843	pOP-EAP01324_EST_C_1_pBSK_SK	299
cl0742	ct0798	cn0844	pOP-EAP01360_EST_C_1_pBSK_SK	143
cl0742	ct0798	cn0844	pOP-EAP01361_EST_C_1_pBSK_SK	227
cl0743	ct0799	cn0845	pOP-CNI01946_EST_C_1_pSK_SK	281
cl0743	ct0799	cn0845	pOP-EAP01392_EST_C_1_pBSK_SK	249
cl0744	ct0800	cn0846	pOP-CBP00125_EST_C_1_pBSK_SK	533
cl0744	ct0800	cn0846	pOP-CNH00599_EST_C_1_pSK_SK	495
cl0744	ct0800	cn0846	pOP-CNI01798_EST_C_1_pSK_SK	481
cl0744	ct0800	cn0846	pOP-EAP01408_EST_C_1_pBSK_SK	127
cl0744	ct0800	cn0846	pOP-EAP02984_EST_C_1_pBSK_SK	305
cl0744	ct0800	cn0846	pOP-EO02528_EST_C_1_pSK_SK	528
cl0744	ct0800	cn0846	pOP-EO02947_EST_C_1_pSK_SK	389
cl0745	ct0801	cn0847	pOP-CNI01564_EST_C_1_pSK_SK	389
cl0745	ct0801	cn0847	pOP-EAP01412_EST_C_1_pBSK_SK	296
cl0746	ct0802	cn0848	pOP-CNHP00171_EST_C_1_pSK_SK	351
cl0746	ct0802	cn0848	pOP-CNIP00165_EST_C_1_pSK_SK	380
cl0746	ct0802	cn0848	pOP-EAP01424_EST_C_1_pBSK_SK	361
cl0747	ct0803	cn0849	pOP-CNIP00260_EST_C_1_pSK_SK	285
cl0747	ct0803	cn0849	pOP-EAP01427_EST_C_1_pBSK_SK	234

cl0748	ct0804	cn0850	pOP-CNH05073_EST_C_1_pSK_SK	648
cl0748	ct0804	cn0850	pOP-EAP01433_EST_C_1_pBSK_SK	276
cl0749	ct0805	cn0851	pOP-EAP01028_EST_C_1_pBSK_SK	584
cl0749	ct0805	cn0851	pOP-EAP01440_EST_C_1_pBSK_SK	478
cl0750	ct0806	cn0852	pOP-EAP01032_EST_C_1_pBSK_SK	595
cl0750	ct0806	cn0852	pOP-EAP01446_EST_C_1_pBSK_SK	398
cl0751	ct0807	cn0853	pOP-EAP01463_EST_C_1_pBSK_SK	651
cl0751	ct0807	cn0853	pOP-EAP01464_EST_C_1_pBSK_SK	340
cl0752	ct0808	cn0854	pOP-EAP00669_EST_C_1_pBSK_SK	627
cl0752	ct0809	cn0855	pOP-EAP01481_EST_C_1_pBSK_SK	637
cl0752	ct0809	cn0856	pOP-CNHP00339_EST_C_1_pSK_SK	577
cl0752	ct0809	cn0857	pOP-CEO01633_EST_C_1_pSK_SK	475
cl0752	ct0810	cn0858	pOP-CNH01374_EST_C_1_pSK_SK	631
cl0752	ct0810	cn0858	pOP-CNH02151_EST_C_1_pSK_SK	464
cl0752	ct0810	cn0858	pOP-CNH04405	726
cl0752	ct0810	cn0858	pOP-EO03006_EST_C_1_pSK_SK	370
cl0752	ct0810	cn0858	pOP-EO07005_EST_C_1_pSK_SK	581
cl0753	ct0811	cn0859	pOP-CNI01882_EST_C_1_pSK_SK	580
cl0753	ct0811	cn0860	pOP-EAP01488_EST_C_1_pBSK_SK	562
cl0753	ct0811	cn0861	pOP-CNI01646_EST_C_1_pSK_SK	393
cl0754	ct0812	cn0862	pOP-CNI01624_EST_C_1_pSK_SK	310
cl0754	ct0812	cn0862	pOP-EAP01500_EST_C_1_pBSK_SK	587
cl0755	ct0813	cn0863	pOP-CNIP00858_EST_C_1_pSK_SK	296
cl0755	ct0813	cn0863	pOP-EAP01528_EST_C_1_pBSK_SK	564
cl0756	ct0814	cn0864	pOP-CEO03517_EST_C_1_pSK_SK	609
cl0756	ct0814	cn0864	pOP-EAP01529_EST_C_1_pBSK_SK	310
cl0757	ct0815	cn0865	pOP-EAP01540_EST_C_1_pBSK_SK	385
cl0757	ct0815	cn0865	pOP-EO07968_EST_C_1_pSK_SK	479
cl0758	ct0816	cn0866	pOP-CNIP00011_EST_C_1_pSK_SK	357
cl0758	ct0816	cn0866	pOP-EAP01541_EST_C_1_pBSK_SK	601
cl0759	ct0817	cn0867	pOP-EAP00788_EST_C_1_pBSK_SK	413
cl0759	ct0817	cn0867	pOP-EAP01545_EST_C_1_pBSK_SK	600
cl0760	ct0818	cn0868	pOP-EAP01558_EST_C_1_pBSK_SK	666
cl0760	ct0818	cn0869	pOP-EAP01472_EST_C_1_pBSK_SK	658
cl0761	ct0819	cn0870	pOP-EAP01579_EST_C_1_pBSK_SK	446
cl0761	ct0819	cn0870	pOP-EO06556_EST_C_1_pSK_SK	775
cl0762	ct0820	cn0871	pOP-CNI01394_EST_C_1_pSK_SK	324
cl0762	ct0820	cn0871	pOP-EAP01582_EST_C_1_pBSK_SK	358
cl0763	ct0821	cn0872	pOP-EAP01514_EST_C_1_pBSK_SK	613
cl0763	ct0821	cn0872	pOP-EAP01589_EST_C_1_pBSK_SK	387
cl0764	ct0822	cn0873	pOP-EAP01600_EST_C_1_pBSK_SK	206
cl0764	ct0822	cn0873	pOP-EAP01601_EST_C_1_pBSK_SK	509
cl0765	ct0823	cn0874	pOP-CEO01011_EST_C_1_pSK_SK	682
cl0765	ct0823	cn0874	pOP-CNH00593_EST_C_1_pSK_SK	310
cl0765	ct0823	cn0874	pOP-CNHP00495_EST_C_1_pSK_SK	595
cl0765	ct0823	cn0874	pOP-CNI01699_EST_C_1_pSK_SK	714
cl0765	ct0823	cn0874	pOP-EAP01603_EST_C_1_pBSK_SK	429
cl0766	ct0824	cn0875	pOP-EAP00819_EST_C_1_pBSK_SK	437
cl0766	ct0824	cn0875	pOP-EAP01605_EST_C_1_pBSK_SK	528
cl0766	ct0824	cn0875	pOP-EO03176_EST_C_1_pSK_SK	433
cl0767	ct0825	cn0876	pOP-EAP01543_EST_C_1_pBSK_SK	611
cl0767	ct0825	cn0876	pOP-EAP01609_EST_C_1_pBSK_SK	520
cl0768	ct0826	cn0877	pOP-CNI01980_EST_C_1_pSK_SK	395
cl0768	ct0826	cn0877	pOP-EAP01612_EST_C_1_pBSK_SK	470
cl0769	ct0827	cn0878	pOP-CNI01597_EST_C_1_pSK_SK	399
cl0769	ct0827	cn0878	pOP-EAP01614_EST_C_1_pBSK_SK	519

cl0770	ct0828	cn0879	pOP-CNH04402	833
cl0770	ct0828	cn0879	pOP-EAP00218_EST_C_1_pBSK_SK	360
cl0770	ct0828	cn0879	pOP-EAP01626_EST_C_1_pBSK_SK	631
cl0770	ct0828	cn0879	pOP-EO04303_EST_C_1_pSK_SK	450
cl0770	ct0828	cn0880	pOP-CNH05041_EST_C_1_pSK_SK	684
cl0771	ct0829	cn0881	pOP-CNIP00092_EST_C_1_pSK_SK	427
cl0771	ct0829	cn0881	pOP-EAP01662_EST_C_1_pBSK_SK	584
cl0772	ct0830	cn0882	pOP-CNH02425_EST_C_1_pSK_SK	536
cl0772	ct0830	cn0882	pOP-EAP01665_EST_C_1_pBSK_SK	624
cl0773	ct0831	cn0883	pOP-CNIP00473_EST_C_1_pSK_SK	358
cl0773	ct0831	cn0883	pOP-EAP01671_EST_C_1_pBSK_SK	612
cl0774	ct0832	cn0884	pOP-EAP01678_EST_C_1_pBSK_SK	492
cl0774	ct0832	cn0884	pOP-EAP01679_EST_C_1_pBSK_SK	335
cl0775	ct0833	cn0885	pOP-EAP01278_EST_C_1_pBSK_SK	259
cl0775	ct0833	cn0885	pOP-EAP01681_EST_C_1_pBSK_SK	275
cl0776	ct0834	cn0886	pOP-EAP01715_EST_C_1_pBSK_SK	667
cl0776	ct0834	cn0886	pOP-EO06280_EST_C_1_pSK_SK	597
cl0777	ct0835	cn0887	pOP-EAP01720_EST_C_1_pBSK_SK	647
cl0777	ct0835	cn0887	pOP-EAP01721_EST_C_1_pBSK_SK	646
cl0778	ct0836	cn0888	pOP-CNIP00028_EST_C_1_pSK_SK	462
cl0778	ct0836	cn0888	pOP-EAP01722_EST_C_1_pBSK_SK	673
cl0779	ct0837	cn0889	pOP-CNI01390_EST_C_1_pSK_SK	486
cl0779	ct0837	cn0889	pOP-CNI02116_EST_C_1_pSK_SK	470
cl0779	ct0837	cn0889	pOP-EAP01725_EST_C_1_pBSK_SK	701
cl0780	ct0838	cn0890	pOP-EAP01751_EST_C_1_pBSK_SK	625
cl0780	ct0838	cn0890	pOP-EO05570_EST_C_1_pSK_SK	430
cl0781	ct0839	cn0891	pOP-CNH00827_EST_C_1_pSK_SK	351
cl0781	ct0839	cn0891	pOP-CNH03497_EST_C_1_pSK_SK	350
cl0781	ct0839	cn0891	pOP-EAP01786_EST_C_1_pBSK_SK	652
cl0781	ct0839	cn0892	pOP-CNH01384_EST_C_1_pSK_SK	581
cl0782	ct0840	cn0893	pOP-EAP01804_EST_C_1_pBSK_SK	610
cl0782	ct0840	cn0893	pOP-EO02962_EST_C_1_pSK_SK	326
cl0783	ct0841	cn0894	pOP-CNIP00424_EST_C_1_pSK_SK	270
cl0783	ct0841	cn0894	pOP-EAP01808_EST_C_1_pBSK_SK	466
cl0784	ct0842	cn0895	pOP-CNI01338_EST_C_1_pSK_SK	199
cl0784	ct0842	cn0895	pOP-CNI01771_EST_C_1_pSK_SK	468
cl0784	ct0842	cn0895	pOP-CNIP00038_EST_C_1_pSK_SK	415
cl0784	ct0842	cn0895	pOP-CNIP00420_EST_C_1_pSK_SK	468
cl0784	ct0842	cn0895	pOP-EAP01814_EST_C_1_pBSK_SK	351
cl0785	ct0843	cn0896	pOP-CNH04899_EST_C_1_pSK_SK	745
cl0785	ct0843	cn0896	pOP-EAP01820_EST_C_1_pBSK_SK	457
cl0785	ct0843	cn0896	pOP-EAP01821_EST_C_1_pBSK_SK	170
cl0786	ct0844	cn0897	pOP-CNH02644_EST_C_1_pSK_SK	533
cl0786	ct0844	cn0897	pOP-EAP01790_EST_C_1_pBSK_SK	481
cl0786	ct0844	cn0897	pOP-EAP01825_EST_C_1_pBSK_SK	347
cl0786	ct0844	cn0897	pOP-EO05491_EST_C_1_pSK_SK	483
cl0787	ct0845	cn0898	pOP-CNI02117_EST_C_1_pSK_SK	415
cl0787	ct0845	cn0898	pOP-EAP01826_EST_C_1_pBSK_SK	530
cl0788	ct0846	cn0899	pOP-CNHP00055_EST_C_1_pSK_SK	652
cl0788	ct0846	cn0899	pOP-EAP01830_EST_C_1_pBSK_SK	688
cl0789	ct0847	cn0900	pOP-CNH04173	384
cl0789	ct0847	cn0900	pOP-CNHP00539_EST_C_1_pSK_SK	571
cl0789	ct0847	cn0900	pOP-EAP01833_EST_C_1_pBSK_SK	716
cl0789	ct0847	cn0900	pOP-EO07107_EST_C_1_pSK_SK	558
cl0790	ct0848	cn0901	pOP-CNIP00159_EST_C_1_pSK_SK	433
cl0790	ct0848	cn0901	pOP-EAP01854_EST_C_1_pBSK_SK	394

cl0791	ct0849	cn0902	pOP-EAP01857_EST_C_1_pBSK_SK	419
cl0791	ct0849	cn0902	pOP-EO08089_EST_C_1_pSK_SK	551
cl0792	ct0850	cn0903	pOP-CNH01618_EST_C_1_pSK_SK	553
cl0792	ct0850	cn0903	pOP-CNIP00510_EST_C_1_pSK_SK	537
cl0792	ct0850	cn0903	pOP-EAP01843_EST_C_1_pBSK_SK	704
cl0792	ct0850	cn0903	pOP-EAP01889_EST_C_1_pBSK_SK	611
cl0792	ct0850	cn0903	pOP-EO07745_EST_C_1_pSK_SK	696
cl0793	ct0851	cn0904	pOP-EAP00699_EST_C_1_pBSK_SK	549
cl0793	ct0851	cn0904	pOP-EAP01368_EST_C_1_pBSK_SK	224
cl0793	ct0851	cn0904	pOP-EAP01743_EST_C_1_pBSK_SK	434
cl0793	ct0851	cn0904	pOP-EAP01895_EST_C_1_pBSK_SK	367
cl0793	ct0851	cn0904	pOP-EAP01910_EST_C_1_pBSK_SK	312
cl0793	ct0851	cn0904	pOP-EAP02067_EST_C_1_pBSK_SK	365
cl0793	ct0851	cn0904	pOP-EAP02373_EST_C_1_pBSK_SK	582
cl0793	ct0851	cn0904	pOP-EAP03202_EST_C_1_pBSK_SK	343
cl0793	ct0852	cn0905	pOP-CNI01937_EST_C_1_pSK_SK	308
cl0793	ct0852	cn0905	pOP-CNIP00637_EST_C_1_pSK_SK	471
cl0793	ct0852	cn0905	pOP-EAP00123_EST_C_1_pBSK_SK	504
cl0793	ct0852	cn0905	pOP-EAP01331_EST_C_1_pBSK_SK	210
cl0793	ct0852	cn0905	pOP-EAP02061_EST_C_1_pBSK_SK	381
cl0793	ct0852	cn0905	pOP-EAP03162_EST_C_1_pBSK_SK	553
cl0793	ct0852	cn0905	pOP-EO02125_EST_C_1_pSK_SK	495
cl0793	ct0852	cn0905	pOP-EO02235_EST_C_1_pSK_SK	506
cl0793	ct0852	cn0905	pOP-EO02786_EST_C_1_pSK_SK	350
cl0793	ct0852	cn0905	pOP-EO05053_EST_C_1_pSK_SK	512
cl0793	ct0852	cn0905	pOP-EO06113_EST_C_1_pSK_SK	559
cl0794	ct0853	cn0906	pOP-CNI01163_EST_C_1_pSK_SK	420
cl0794	ct0853	cn0906	pOP-CNI01219_EST_C_1_pSK_SK	254
cl0794	ct0853	cn0906	pOP-EAP01944_EST_C_1_pBSK_SK	284
cl0795	ct0854	cn0907	pOP-EAP00517_EST_C_1_pBSK_SK	548
cl0795	ct0854	cn0907	pOP-EAP00519_EST_C_1_pBSK_SK	549
cl0795	ct0854	cn0907	pOP-EAP01948_EST_C_1_pBSK_SK	468
cl0796	ct0855	cn0908	pOP-EAP01193_EST_C_1_pBSK_SK	325
cl0796	ct0855	cn0908	pOP-EAP01952_EST_C_1_pBSK_SK	232
cl0797	ct0856	cn0909	pOP-CNH04694	729
cl0797	ct0856	cn0909	pOP-EAP01373_EST_C_1_pBSK_SK	221
cl0797	ct0856	cn0909	pOP-EAP01968_EST_C_1_pBSK_SK	201
cl0798	ct0857	cn0910	pOP-CNH03371_EST_C_1_pSK_SK	619
cl0798	ct0857	cn0910	pOP-CNH03786_EST_C_1_pSK_SK	460
cl0798	ct0857	cn0910	pOP-CNHP00053_EST_C_1_pSK_SK	619
cl0798	ct0857	cn0910	pOP-EAP00612_EST_C_1_pBSK_SK	577
cl0798	ct0857	cn0910	pOP-EAP02065_EST_C_1_pBSK_SK	352
cl0798	ct0857	cn0910	pOP-EO05691_EST_C_1_pSK_SK	461
cl0799	ct0858	cn0911	pOP-CNI01838_EST_C_1_pSK_SK	151
cl0799	ct0858	cn0911	pOP-EAP02073_EST_C_1_pBSK_SK	297
cl0800	ct0859	cn0912	pOP-EAP02077_EST_C_1_pBSK_SK	451
cl0800	ct0859	cn0912	pOP-EO06954_EST_C_1_pSK_SK	716
cl0801	ct0860	cn0913	pOP-EAP01214_EST_C_1_pBSK_SK	251
cl0801	ct0860	cn0913	pOP-EAP02080_EST_C_1_pBSK_SK	169
cl0802	ct0861	cn0914	pOP-CEO01403_EST_C_1_pSK_SK	416
cl0802	ct0861	cn0914	pOP-CNH01047_EST_C_1_pSK_SK	474
cl0802	ct0861	cn0914	pOP-CNH01920_EST_C_1_pSK_SK	444
cl0802	ct0861	cn0914	pOP-EAP02085_EST_C_1_pBSK_SK	308
cl0802	ct0861	cn0914	pOP-EAP03396_EST_C_1_pBSK_SK	538
cl0802	ct0861	cn0914	pOP-EN00595_EST_C_1_pSK_SK	324
cl0802	ct0861	cn0914	pOP-EO03991_EST_C_1_pSK_SK	530

cl0802	ct0861	cn0914	pOP-EO04167_EST_C_1_pSK_SK	531
cl0802	ct0861	cn0914	pOP-EO05872_EST_C_1_pSK_SK	475
cl0803	ct0862	cn0915	pOP-EAP02086_EST_C_1_pBSK_SK	654
cl0803	ct0862	cn0916	pOP-EAP02087_EST_C_1_pBSK_SK	611
cl0804	ct0863	cn0917	pOP-CEO03194_EST_C_1_pSK_SK	155
cl0804	ct0863	cn0917	pOP-EAP02098_EST_C_1_pBSK_SK	135
cl0805	ct0864	cn0918	pOP-EAP02124_EST_C_1_pBSK_SK	296
cl0805	ct0864	cn0918	pOP-EO05791_EST_C_1_pSK_SK	629
cl0806	ct0865	cn0919	pOP-CNH01394_EST_C_1_pSK_SK	610
cl0806	ct0865	cn0919	pOP-CNHP00419_EST_C_1_pSK_SK	691
cl0806	ct0865	cn0919	pOP-CNI01140_EST_C_1_pSK_SK	426
cl0806	ct0865	cn0919	pOP-EAP02133_EST_C_1_pBSK_SK	615
cl0807	ct0866	cn0920	pOP-CNH03664_EST_C_1_pSK_SK	510
cl0807	ct0866	cn0920	pOP-CNHP00137_EST_C_1_pSK_SK	683
cl0807	ct0866	cn0920	pOP-CNHP00504_EST_C_1_pSK_SK	565
cl0807	ct0866	cn0920	pOP-CNI01836_EST_C_1_pSK_SK	492
cl0807	ct0866	cn0920	pOP-EAP02142_EST_C_1_pBSK_SK	227
cl0808	ct0867	cn0921	pOP-EAP02177_EST_C_1_pBSK_SK	391
cl0808	ct0867	cn0921	pOP-EAP02178_EST_C_1_pBSK_SK	203
cl0809	ct0868	cn0922	pOP-CNH04625	750
cl0809	ct0869	cn0923	pOP-CBP00037_EST_C_1_pBSK_SK	684
cl0809	ct0869	cn0923	pOP-CNH02651_EST_C_1_pSK_SK	633
cl0809	ct0869	cn0923	pOP-CNH03230_EST_C_1_pSK_SK	606
cl0809	ct0869	cn0923	pOP-CNH04270	531
cl0809	ct0869	cn0923	pOP-CNH04664	415
cl0809	ct0869	cn0923	pOP-CNI02206_EST_C_1_pSK_SK	162
cl0809	ct0869	cn0923	pOP-EAP02290_EST_C_1_pBSK_SK	627
cl0809	ct0869	cn0923	pOP-EAP03754_EST_C_1_pBSK_SK	228
cl0809	ct0869	cn0923	pOP-EAP03843_EST_C_1_pBSK_SK	593
cl0809	ct0869	cn0923	pOP-EO02144_EST_C_1_pSK_SK	382
cl0809	ct0869	cn0923	pOP-EO03646_EST_C_1_pSK_SK	384
cl0809	ct0869	cn0923	pOP-EO08389_EST_C_1_pSK_SK	366
cl0809	ct0869	cn0923	pOP-EO08454_EST_C_1_pSK_SK	344
cl0809	ct0869	cn0924	pOP-CEO03453_EST_C_1_pSK_SK	519
cl0809	ct0870	cn0925	pOP-CAP00330_EST_C_1_pBSK_SK	376
cl0809	ct0870	cn0925	pOP-CBP00166_EST_C_1_pBSK_SK	430
cl0809	ct0870	cn0925	pOP-CBP00236_EST_C_1_pBSK_SK	206
cl0809	ct0870	cn0925	pOP-CEM00153_EST_C_1_pSK_SK	141
cl0809	ct0870	cn0925	pOP-CEMP00032_EST_C_1_pSK_SK	252
cl0809	ct0870	cn0925	pOP-CEO01149_EST_C_1_pSK_SK	488
cl0809	ct0870	cn0925	pOP-CEO01356	285
cl0809	ct0870	cn0925	pOP-CEO01389_EST_C_1_pSK_SK	578
cl0809	ct0870	cn0925	pOP-CEO01419_EST_C_1_pSK_SK	173
cl0809	ct0870	cn0925	pOP-CEO01449_EST_C_1_pSK_SK	513
cl0809	ct0870	cn0925	pOP-CEO01734_EST_C_1_pSK_SK	184
cl0809	ct0870	cn0925	pOP-CEO01736_EST_C_1_pSK_SK	179
cl0809	ct0870	cn0925	pOP-CEO01737_EST_C_1_pSK_SK	182
cl0809	ct0870	cn0925	pOP-CEO01930_EST_C_1_pSK_SK	388
cl0809	ct0870	cn0925	pOP-CEO02070_EST_C_1_pSK_SK	170
cl0809	ct0870	cn0925	pOP-CEO03284_EST_C_1_pSK_SK	256
cl0809	ct0870	cn0925	pOP-CEO03426_EST_C_1_pSK_SK	636
cl0809	ct0870	cn0925	pOP-CEO03498_EST_C_1_pSK_SK	594
cl0809	ct0870	cn0925	pOP-CEO03499_EST_C_1_pSK_SK	379
cl0809	ct0870	cn0925	pOP-CEOP00033_EST_C_1_pSK_SK	501
cl0809	ct0870	cn0925	pOP-CNH01264_EST_C_1_pSK_SK	486
cl0809	ct0870	cn0925	pOP-CNH01405_EST_C_1_pSK_SK	554

cl0809	ct0870	cn0925	pOP-CNH02411_EST_C_1_pSK_SK	439
cl0809	ct0870	cn0925	pOP-CNH02420_EST_C_1_pSK_SK	542
cl0809	ct0870	cn0925	pOP-CNH02445_EST_C_1_pSK_SK	464
cl0809	ct0870	cn0925	pOP-CNH02640_EST_C_1_pSK_SK	520
cl0809	ct0870	cn0925	pOP-CNH03581_EST_C_1_pSK_SK	349
cl0809	ct0870	cn0925	pOP-CNH04902_EST_C_1_pSK_SK	540
cl0809	ct0870	cn0925	pOP-CNHP00078_EST_C_1_pSK_SK	520
cl0809	ct0870	cn0925	pOP-CNHP00381_EST_C_1_pSK_SK	517
cl0809	ct0870	cn0925	pOP-CNI01282_EST_C_1_pSK_SK	492
cl0809	ct0870	cn0925	pOP-CNI01468_EST_C_1_pSK_SK	386
cl0809	ct0870	cn0925	pOP-CNI01537_EST_C_1_pSK_SK	501
cl0809	ct0870	cn0925	pOP-CNIP00080_EST_C_1_pSK_SK	629
cl0809	ct0870	cn0925	pOP-CNIP00488_EST_C_1_pSK_SK	129
cl0809	ct0870	cn0925	pOP-CNIP00650_EST_C_1_pSK_SK	558
cl0809	ct0870	cn0925	pOP-CNIP00762_EST_C_1_pSK_SK	622
cl0809	ct0870	cn0925	pOP-CNIP00800_EST_C_1_pSK_SK	376
cl0809	ct0870	cn0925	pOP-CNIP00935_EST_C_1_pSK_SK	465
cl0809	ct0870	cn0925	pOP-EAP00158_EST_C_1_pBSK_SK	390
cl0809	ct0870	cn0925	pOP-EAP00159_EST_C_1_pBSK_SK	386
cl0809	ct0870	cn0925	pOP-EAP00160_EST_C_1_pBSK_SK	369
cl0809	ct0870	cn0925	pOP-EAP00161_EST_C_1_pBSK_SK	391
cl0809	ct0870	cn0925	pOP-EAP00162_EST_C_1_pBSK_SK	186
cl0809	ct0870	cn0925	pOP-EAP00324_EST_C_1_pBSK_SK	157
cl0809	ct0870	cn0925	pOP-EAP00643_EST_C_1_pBSK_SK	492
cl0809	ct0870	cn0925	pOP-EAP00991_EST_C_1_pBSK_SK	486
cl0809	ct0870	cn0925	pOP-EAP01087_EST_C_1_pBSK_SK	153
cl0809	ct0870	cn0925	pOP-EAP01089_EST_C_1_pBSK_SK	540
cl0809	ct0870	cn0925	pOP-EAP01180_EST_C_1_pBSK_SK	235
cl0809	ct0870	cn0925	pOP-EAP01887_EST_C_1_pBSK_SK	413
cl0809	ct0870	cn0925	pOP-EAP01960_EST_C_1_pBSK_SK	431
cl0809	ct0870	cn0925	pOP-EAP02123_EST_C_1_pBSK_SK	391
cl0809	ct0870	cn0925	pOP-EAP02187_EST_C_1_pBSK_SK	200
cl0809	ct0870	cn0925	pOP-EAP02763_EST_C_1_pBSK_SK	501
cl0809	ct0870	cn0925	pOP-EAP02897_EST_C_1_pBSK_SK	574
cl0809	ct0870	cn0925	pOP-EAP03386_EST_C_1_pBSK_SK	486
cl0809	ct0870	cn0925	pOP-EAP03777_EST_C_1_pBSK_SK	307
cl0809	ct0870	cn0925	pOP-EAP03847_EST_C_1_pBSK_SK	435
cl0809	ct0870	cn0925	pOP-EBP03120_EST_C_1_pBSK_M13F	230
cl0809	ct0870	cn0925	pOP-EO02538_EST_C_1_pSK_SK	525
cl0809	ct0870	cn0925	pOP-EO02692_EST_C_1_pSK_SK	446
cl0809	ct0870	cn0925	pOP-EO03829_EST_C_1_pSK_SK	490
cl0810	ct0871	cn0926	pOP-CNH03419_EST_C_1_pSK_SK	512
cl0810	ct0871	cn0926	pOP-EAP00513_EST_C_1_pBSK_SK	588
cl0810	ct0871	cn0926	pOP-EAP01471_EST_C_1_pBSK_SK	598
cl0810	ct0871	cn0926	pOP-EAP01916_EST_C_1_pBSK_SK	534
cl0810	ct0871	cn0926	pOP-EAP02201_EST_C_1_pBSK_SK	229
cl0810	ct0871	cn0926	pOP-EAP02799_EST_C_1_pBSK_SK	407
cl0811	ct0872	cn0927	pOP-CNH01058_EST_C_1_pSK_SK	568
cl0811	ct0872	cn0927	pOP-CNH01508_EST_C_1_pSK_SK	497
cl0811	ct0872	cn0927	pOP-CNH04692	746
cl0811	ct0872	cn0927	pOP-CNHP00072_EST_C_1_pSK_SK	655
cl0811	ct0872	cn0927	pOP-EAP02203_EST_C_1_pBSK_SK	243
cl0811	ct0872	cn0927	pOP-EO03256_EST_C_1_pSK_SK	427
cl0812	ct0873	cn0928	pOP-CEO00806_EST_C_1_pSK_SK	573
cl0812	ct0873	cn0928	pOP-EAP02213_EST_C_1_pBSK_SK	525
cl0813	ct0874	cn0929	pOP-CNI01677_EST_C_1_pSK_SK	536

cl0813	ct0874	cn0929	pOP-CNIP00371_EST_C_1_pSK_SK	509
cl0813	ct0874	cn0929	pOP-EAP02218_EST_C_1_pBSK_SK	218
cl0814	ct0875	cn0930	pOP-EAP02219_EST_C_1_pBSK_SK	537
cl0814	ct0875	cn0930	pOP-EO04647_EST_C_1_pSK_SK	482
cl0815	ct0876	cn0931	pOP-EAP02222_EST_C_1_pBSK_SK	125
cl0815	ct0876	cn0931	pOP-EAP02223_EST_C_1_pBSK_SK	244
cl0816	ct0877	cn0932	pOP-EAP02224_EST_C_1_pBSK_SK	226
cl0816	ct0877	cn0932	pOP-EO04435_EST_C_1_pSK_SK	487
cl0817	ct0878	cn0933	pOP-EAP00482_EST_C_1_pBSK_SK	149
cl0817	ct0878	cn0933	pOP-EAP02232_EST_C_1_pBSK_SK	168
cl0818	ct0879	cn0934	pOP-EAP02235_EST_C_1_pBSK_SK	366
cl0818	ct0879	cn0934	pOP-EAP02245_EST_C_1_pBSK_SK	408
cl0819	ct0880	cn0935	pOP-EAP00690_EST_C_1_pBSK_SK	651
cl0819	ct0880	cn0935	pOP-EAP02246_EST_C_1_pBSK_SK	388
cl0820	ct0881	cn0936	pOP-EAP00251_EST_C_1_pBSK_SK	156
cl0820	ct0881	cn0936	pOP-EAP02254_EST_C_1_pBSK_SK	664
cl0821	ct0882	cn0937	pOP-EAP02259_EST_C_1_pBSK_SK	562
cl0821	ct0883	cn0938	pOP-EO06073_EST_C_1_pSK_SK	430
cl0821	ct0883	cn0939	pOP-CNIP00438_EST_C_1_pSK_SK	305
cl0822	ct0884	cn0940	pOP-CNI01538_EST_C_1_pSK_SK	291
cl0822	ct0884	cn0940	pOP-EAP01312_EST_C_1_pBSK_SK	347
cl0822	ct0884	cn0940	pOP-EAP02279_EST_C_1_pBSK_SK	231
cl0823	ct0885	cn0941	pOP-CNIP00413_EST_C_1_pSK_SK	632
cl0823	ct0885	cn0941	pOP-EAP02280_EST_C_1_pBSK_SK	314
cl0824	ct0886	cn0942	pOP-CNNP00031_EST_C_1_pBSK_SK	270
cl0824	ct0886	cn0942	pOP-EAP02285_EST_C_1_pBSK_SK	236
cl0824	ct0886	cn0942	pOP-EAP02286_EST_C_1_pBSK_SK	288
cl0825	ct0887	cn0943	pOP-EAP01205_EST_C_1_pBSK_SK	600
cl0825	ct0887	cn0943	pOP-EAP02287_EST_C_1_pBSK_SK	548
cl0826	ct0888	cn0944	pOP-EAP02303_EST_C_1_pBSK_SK	728
cl0826	ct0888	cn0944	pOP-EO05722_EST_C_1_pSK_SK	457
cl0827	ct0889	cn0945	pOP-EAP01516_EST_C_1_pBSK_SK	583
cl0827	ct0889	cn0945	pOP-EAP02306_EST_C_1_pBSK_SK	607
cl0828	ct0890	cn0946	pOP-EAP00791_EST_C_1_pBSK_SK	299
cl0828	ct0890	cn0946	pOP-EAP02314_EST_C_1_pBSK_SK	577
cl0829	ct0891	cn0947	pOP-EAP00939_EST_C_1_pBSK_SK	150
cl0829	ct0891	cn0947	pOP-EAP02315_EST_C_1_pBSK_SK	725
cl0830	ct0892	cn0948	pOP-CNH01270_EST_C_1_pSK_SK	556
cl0830	ct0892	cn0948	pOP-CNH03540_EST_C_1_pSK_SK	346
cl0830	ct0892	cn0948	pOP-CNI01128_EST_C_1_pSK_SK	376
cl0830	ct0892	cn0948	pOP-CNI01129_EST_C_1_pSK_SK	281
cl0830	ct0892	cn0948	pOP-CNIP00139_EST_C_1_pSK_SK	564
cl0830	ct0892	cn0948	pOP-EAP02321_EST_C_1_pBSK_SK	754
cl0831	ct0893	cn0949	pOP-CEO03486_EST_C_1_pSK_SK	501
cl0831	ct0893	cn0950	pOP-CEO01834_EST_C_1_pSK_SK	328
cl0831	ct0893	cn0950	pOP-EAP02325_EST_C_1_pBSK_SK	437
cl0831	ct0893	cn0951	pOP-EO05869_EST_C_1_pSK_SK	315
cl0832	ct0894	cn0952	pOP-CNIP00158_EST_C_1_pSK_SK	325
cl0832	ct0894	cn0952	pOP-EAP00323_EST_C_1_pBSK_SK	105
cl0832	ct0894	cn0952	pOP-EAP02327_EST_C_1_pBSK_SK	338
cl0833	ct0895	cn0953	pOP-EAP01943_EST_C_1_pBSK_SK	439
cl0833	ct0895	cn0953	pOP-EAP02328_EST_C_1_pBSK_SK	749
cl0834	ct0896	cn0954	pOP-EAP02334_EST_C_1_pBSK_SK	664
cl0834	ct0896	cn0954	pOP-EO04136_EST_C_1_pSK_SK	490
cl0834	ct0896	cn0954	pOP-EO04174_EST_C_1_pSK_SK	496
cl0835	ct0897	cn0955	pOP-CNI01584_EST_C_1_pSK_SK	283

cl0835	ct0897	cn0955	pOP-EAP00488_EST_C_1_pBSK_SK	550
cl0835	ct0897	cn0955	pOP-EAP00490_EST_C_1_pBSK_SK	535
cl0835	ct0897	cn0955	pOP-EAP02343_EST_C_1_pBSK_SK	460
cl0836	ct0898	cn0956	pOP-EAP00986_EST_C_1_pBSK_SK	282
cl0836	ct0898	cn0956	pOP-EAP02346_EST_C_1_pBSK_SK	319
cl0837	ct0899	cn0957	pOP-CEO02735_EST_C_1_pSK_SK	465
cl0837	ct0899	cn0957	pOP-CNH00571_EST_C_1_pSK_SK	388
cl0837	ct0900	cn0958	pOP-CNH02134_EST_C_1_pSK_SK	402
cl0837	ct0900	cn0958	pOP-CNH03105_EST_C_1_pSK_SK	389
cl0837	ct0900	cn0958	pOP-CNH04140	359
cl0837	ct0900	cn0958	pOP-CNIP00904_EST_C_1_pSK_SK	187
cl0837	ct0900	cn0958	pOP-EAP01896_EST_C_1_pBSK_SK	158
cl0837	ct0901	cn0959	pOP-CEMP00019_EST_C_1_pSK_SK	244
cl0837	ct0901	cn0959	pOP-CEO01692_EST_C_1_pSK_SK	656
cl0837	ct0901	cn0959	pOP-CEO03483_EST_C_1_pSK_SK	472
cl0837	ct0901	cn0959	pOP-CNH00681_EST_C_1_pSK_SK	516
cl0837	ct0901	cn0959	pOP-CNH00758_EST_C_1_pSK_SK	461
cl0837	ct0901	cn0959	pOP-CNH00894_EST_C_1_pSK_SK	480
cl0837	ct0901	cn0959	pOP-CNH01035_EST_C_1_pSK_SK	254
cl0837	ct0901	cn0959	pOP-CNH01044_EST_C_1_pSK_SK	410
cl0837	ct0901	cn0959	pOP-CNH01109_EST_C_1_pSK_SK	277
cl0837	ct0901	cn0959	pOP-CNH01503_EST_C_1_pSK_SK	480
cl0837	ct0901	cn0959	pOP-CNH01597_EST_C_1_pSK_SK	486
cl0837	ct0901	cn0959	pOP-CNH01696_EST_C_1_pSK_SK	363
cl0837	ct0901	cn0959	pOP-CNH02178_EST_C_1_pSK_SK	430
cl0837	ct0901	cn0959	pOP-CNH02225_EST_C_1_pSK_SK	573
cl0837	ct0901	cn0959	pOP-CNH02434_EST_C_1_pSK_SK	441
cl0837	ct0901	cn0959	pOP-CNH02720_EST_C_1_pSK_SK	658
cl0837	ct0901	cn0959	pOP-CNH02777_EST_C_1_pSK_SK	705
cl0837	ct0901	cn0959	pOP-CNH02859_EST_C_1_pSK_SK	532
cl0837	ct0901	cn0959	pOP-CNH03055_EST_C_1_pSK_SK	605
cl0837	ct0901	cn0959	pOP-CNH03109_EST_C_1_pSK_SK	532
cl0837	ct0901	cn0959	pOP-CNH03286_EST_C_1_pSK_SK	633
cl0837	ct0901	cn0959	pOP-CNH03667_EST_C_1_pSK_SK	433
cl0837	ct0901	cn0959	pOP-CNH03777_EST_C_1_pSK_SK	552
cl0837	ct0901	cn0959	pOP-CNH04329	808
cl0837	ct0901	cn0959	pOP-CNH05058_EST_C_1_pSK_SK	505
cl0837	ct0901	cn0959	pOP-CNHP00204_EST_C_1_pSK_SK	554
cl0837	ct0901	cn0959	pOP-CNHP00349_EST_C_1_pSK_SK	568
cl0837	ct0901	cn0959	pOP-CNI01846_EST_C_1_pSK_SK	306
cl0837	ct0901	cn0959	pOP-CNIP00199_EST_C_1_pSK_SK	498
cl0837	ct0901	cn0959	pOP-CNIP00859_EST_C_1_pSK_SK	498
cl0837	ct0901	cn0959	pOP-EAP00653_EST_C_1_pBSK_SK	523
cl0837	ct0901	cn0959	pOP-EAP00825_EST_C_1_pBSK_SK	252
cl0837	ct0901	cn0959	pOP-EAP00926_EST_C_1_pBSK_SK	385
cl0837	ct0901	cn0959	pOP-EAP01096_EST_C_1_pBSK_SK	373
cl0837	ct0901	cn0959	pOP-EAP01309_EST_C_1_pBSK_SK	608
cl0837	ct0901	cn0959	pOP-EAP01811_EST_C_1_pBSK_SK	674
cl0837	ct0901	cn0959	pOP-EAP02350_EST_C_1_pBSK_SK	644
cl0837	ct0901	cn0959	pOP-EAP03144_EST_C_1_pBSK_SK	672
cl0837	ct0901	cn0959	pOP-EAP03835_EST_C_1_pBSK_SK	322
cl0837	ct0901	cn0959	pOP-EN00592_EST_C_1_pSK_SK	528
cl0837	ct0901	cn0959	pOP-EN00772_EST_C_1_pSK_SK	580
cl0837	ct0901	cn0959	pOP-EO02700_EST_C_1_pSK_SK	446
cl0837	ct0901	cn0959	pOP-EO04774_EST_C_1_pSK_SK	525
cl0837	ct0901	cn0959	pOP-EO06198_EST_C_1_pSK_SK	514

cl0837	ct0901	cn0959	pOP-EO06548_EST_C_1_pSK_SK	693
cl0838	ct0902	cn0960	pOP-EAP02175_EST_C_1_pBSK_SK	195
cl0838	ct0902	cn0960	pOP-EAP02353_EST_C_1_pBSK_SK	471
cl0839	ct0903	cn0961	pOP-CNH01601_EST_C_1_pSK_SK	478
cl0839	ct0903	cn0961	pOP-CNIP00277_EST_C_1_pSK_SK	226
cl0839	ct0903	cn0961	pOP-EAP02354_EST_C_1_pBSK_SK	739
cl0840	ct0904	cn0962	pOP-EAP01000_EST_C_1_pBSK_SK	180
cl0840	ct0904	cn0962	pOP-EAP02362_EST_C_1_pBSK_SK	689
cl0841	ct0905	cn0963	pOP-EAP00935_EST_C_1_pBSK_SK	441
cl0841	ct0905	cn0963	pOP-EAP01162_EST_C_1_pBSK_SK	106
cl0841	ct0905	cn0963	pOP-EAP02369_EST_C_1_pBSK_SK	452
cl0841	ct0905	cn0963	pOP-EO02571_EST_C_1_pSK_SK	430
cl0841	ct0905	cn0963	pOP-EO03568_EST_C_1_pSK_SK	539
cl0841	ct0905	cn0964	pOP-EO04913_EST_C_1_pSK_SK	456
cl0842	ct0906	cn0965	pOP-EAP02377_EST_C_1_pBSK_SK	131
cl0842	ct0906	cn0965	pOP-EAP02378_EST_C_1_pBSK_SK	329
cl0843	ct0907	cn0966	pOP-CNIP00317_EST_C_1_pSK_SK	564
cl0843	ct0908	cn0967	pOP-EAP02383_EST_C_1_pBSK_SK	640
cl0844	ct0909	cn0968	pOP-CNI02018_EST_C_1_pSK_SK	377
cl0844	ct0909	cn0968	pOP-CNI02039_EST_C_1_pSK_SK	287
cl0844	ct0909	cn0968	pOP-CNIP00950_EST_C_1_pSK_SK	255
cl0844	ct0909	cn0968	pOP-EAP02394_EST_C_1_pBSK_SK	407
cl0845	ct0910	cn0969	pOP-EAP02397_EST_C_1_pBSK_SK	587
cl0845	ct0910	cn0969	pOP-EO05451_EST_C_1_pSK_SK	471
cl0845	ct0910	cn0970	pOP-CNH01128_EST_C_1_pSK_SK	540
cl0846	ct0911	cn0971	pOP-EAP02341_EST_C_1_pBSK_SK	539
cl0846	ct0911	cn0971	pOP-EAP02730_EST_C_1_pBSK_SK	451
cl0846	ct0911	cn0971	pOP-EO08370_EST_C_1_pSK_SK	426
cl0847	ct0912	cn0972	pOP-EAP01914_EST_C_1_pBSK_SK	366
cl0847	ct0912	cn0972	pOP-EAP02732_EST_C_1_pBSK_SK	312
cl0848	ct0913	cn0973	pOP-EAP00860_EST_C_1_pBSK_SK	373
cl0848	ct0913	cn0973	pOP-EAP02744_EST_C_1_pBSK_SK	324
cl0849	ct0914	cn0974	pOP-EAP02241_EST_C_1_pBSK_SK	377
cl0849	ct0914	cn0974	pOP-EAP02746_EST_C_1_pBSK_SK	332
cl0850	ct0915	cn0975	pOP-CNHP00161_EST_C_1_pSK_SK	440
cl0850	ct0915	cn0975	pOP-EAP02762_EST_C_1_pBSK_SK	458
cl0851	ct0916	cn0976	pOP-EAP02355_EST_C_1_pBSK_SK	729
cl0851	ct0916	cn0976	pOP-EAP02772_EST_C_1_pBSK_SK	425
cl0852	ct0917	cn0977	pOP-EAP02781_EST_C_1_pBSK_SK	308
cl0852	ct0917	cn0977	pOP-EO05459_EST_C_1_pSK_SK	484
cl0853	ct0918	cn0978	pOP-EAP00678_EST_C_1_pBSK_SK	532
cl0853	ct0918	cn0978	pOP-EAP02797_EST_C_1_pBSK_SK	480
cl0854	ct0919	cn0979	pOP-CNI01777_EST_C_1_pSK_SK	157
cl0854	ct0919	cn0979	pOP-CNI01805_EST_C_1_pSK_SK	153
cl0854	ct0919	cn0979	pOP-EAP02801_EST_C_1_pBSK_SK	234
cl0855	ct0920	cn0980	pOP-EAP01591_EST_C_1_pBSK_SK	417
cl0855	ct0920	cn0980	pOP-EAP02803_EST_C_1_pBSK_SK	384
cl0856	ct0921	cn0981	pOP-CNH02354_EST_C_1_pSK_SK	584
cl0856	ct0921	cn0981	pOP-EAP02804_EST_C_1_pBSK_SK	387
cl0857	ct0922	cn0982	pOP-CNIP00855_EST_C_1_pSK_SK	273
cl0857	ct0922	cn0982	pOP-EAP02811_EST_C_1_pBSK_SK	339
cl0858	ct0923	cn0983	pOP-EAP01013_EST_C_1_pBSK_SK	200
cl0858	ct0923	cn0983	pOP-EAP02814_EST_C_1_pBSK_SK	441
cl0859	ct0924	cn0984	pOP-CNH01404_EST_C_1_pSK_SK	625
cl0859	ct0924	cn0984	pOP-EAP02818_EST_C_1_pBSK_SK	556
cl0859	ct0924	cn0985	pOP-CNHP00093_EST_C_1_pSK_SK	648

cl0860	ct0925	cn0986	pOP-EAP02822_EST_C_1_pBSK_SK	468
cl0860	ct0925	cn0986	pOP-EO07028_EST_C_1_pSK_SK	637
cl0861	ct0926	cn0987	pOP-CNHP00244_EST_C_1_pSK_SK	593
cl0861	ct0926	cn0988	pOP-EAP02823_EST_C_1_pBSK_SK	428
cl0862	ct0927	cn0989	pOP-EAP02831_EST_C_1_pBSK_SK	738
cl0862	ct0928	cn0990	pOP-EAP00551_EST_C_1_pBSK_SK	618
cl0863	ct0929	cn0991	pOP-EAP00795_EST_C_1_pBSK_SK	448
cl0863	ct0929	cn0991	pOP-EAP02833_EST_C_1_pBSK_SK	520
cl0864	ct0930	cn0992	pOP-CNH00811_EST_C_1_pSK_SK	632
cl0864	ct0930	cn0992	pOP-CNH01558_EST_C_1_pSK_SK	660
cl0864	ct0930	cn0992	pOP-EAP02834_EST_C_1_pBSK_SK	617
cl0865	ct0931	cn0993	pOP-EAP01644_EST_C_1_pBSK_SK	632
cl0865	ct0931	cn0993	pOP-EAP02837_EST_C_1_pBSK_SK	684
cl0866	ct0932	cn0994	pOP-EAP02838_EST_C_1_pBSK_SK	621
cl0866	ct0932	cn0994	pOP-EO06348_EST_C_1_pSK_SK	479
cl0867	ct0933	cn0995	pOP-CNH05056_EST_C_1_pSK_SK	329
cl0867	ct0933	cn0995	pOP-EAP02840_EST_C_1_pBSK_SK	619
cl0868	ct0934	cn0996	pOP-EAP00590_EST_C_1_pBSK_SK	580
cl0868	ct0934	cn0996	pOP-EAP02845_EST_C_1_pBSK_SK	596
cl0869	ct0935	cn0997	pOP-EAP00431_EST_C_1_pBSK_SK	305
cl0869	ct0935	cn0997	pOP-EAP02856_EST_C_1_pBSK_SK	361
cl0870	ct0936	cn0998	pOP-EAP02029_EST_C_1_pBSK_SK	120
cl0870	ct0936	cn0998	pOP-EAP02873_EST_C_1_pBSK_SK	560
cl0871	ct0937	cn0999	pOP-EAP00823_EST_C_1_pBSK_SK	391
cl0871	ct0937	cn0999	pOP-EAP01045_EST_C_1_pBSK_SK	448
cl0871	ct0937	cn0999	pOP-EAP01350_EST_C_1_pBSK_SK	401
cl0871	ct0937	cn0999	pOP-EAP02874_EST_C_1_pBSK_SK	409
cl0872	ct0938	cn1000	pOP-EAP01048_EST_C_1_pBSK_SK	494
cl0872	ct0938	cn1000	pOP-EAP02877_EST_C_1_pBSK_SK	452
cl0873	ct0939	cn1001	pOP-EAP01023_EST_C_1_pBSK_SK	487
cl0873	ct0939	cn1001	pOP-EAP01049_EST_C_1_pBSK_SK	357
cl0873	ct0939	cn1001	pOP-EAP02878_EST_C_1_pBSK_SK	334
cl0874	ct0940	cn1002	pOP-EAP01051_EST_C_1_pBSK_SK	190
cl0874	ct0940	cn1002	pOP-EAP02880_EST_C_1_pBSK_SK	143
cl0875	ct0941	cn1003	pOP-EAP01061_EST_C_1_pBSK_SK	539
cl0875	ct0941	cn1003	pOP-EAP02884_EST_C_1_pBSK_SK	509
cl0876	ct0942	cn1004	pOP-EAP01065_EST_C_1_pBSK_SK	458
cl0876	ct0942	cn1004	pOP-EAP02885_EST_C_1_pBSK_SK	608
cl0877	ct0943	cn1005	pOP-EAP01069_EST_C_1_pBSK_SK	387
cl0877	ct0943	cn1005	pOP-EAP02887_EST_C_1_pBSK_SK	361
cl0878	ct0944	cn1006	pOP-EAP01070_EST_C_1_pBSK_SK	244
cl0878	ct0944	cn1006	pOP-EAP01398_EST_C_1_pBSK_SK	150
cl0878	ct0944	cn1006	pOP-EAP02888_EST_C_1_pBSK_SK	220
cl0879	ct0945	cn1007	pOP-EAP01073_EST_C_1_pBSK_SK	414
cl0879	ct0945	cn1007	pOP-EAP02890_EST_C_1_pBSK_SK	380
cl0880	ct0946	cn1008	pOP-EAP01084_EST_C_1_pBSK_SK	425
cl0880	ct0946	cn1008	pOP-EAP02895_EST_C_1_pBSK_SK	398
cl0881	ct0947	cn1009	pOP-EAP01088_EST_C_1_pBSK_SK	242
cl0881	ct0947	cn1009	pOP-EAP02896_EST_C_1_pBSK_SK	211
cl0881	ct0947	cn1009	pOP-EO04710_EST_C_1_pSK_SK	467
cl0882	ct0948	cn1010	pOP-EAP01105_EST_C_1_pBSK_SK	237
cl0882	ct0948	cn1010	pOP-EAP02812_EST_C_1_pBSK_SK	303
cl0882	ct0948	cn1010	pOP-EAP02900_EST_C_1_pBSK_SK	220
cl0883	ct0949	cn1011	pOP-CNIP00004_EST_C_1_pSK_SK	635
cl0883	ct0949	cn1011	pOP-EAP02915_EST_C_1_pBSK_SK	663
cl0884	ct0950	cn1012	pOP-EAP00310_EST_C_1_pBSK_SK	471

cl0884	ct0950	cn1012	pOP-EAP01873_EST_C_1_pBSK_SK	472
cl0884	ct0950	cn1012	pOP-EAP02923_EST_C_1_pBSK_SK	652
cl0885	ct0951	cn1013	pOP-EAP00563_EST_C_1_pBSK_SK	279
cl0885	ct0952	cn1014	pOP-EAP02924_EST_C_1_pBSK_SK	456
cl0886	ct0953	cn1015	pOP-CNI02181_EST_C_1_pSK_SK	210
cl0886	ct0953	cn1015	pOP-EAP02930_EST_C_1_pBSK_SK	441
cl0887	ct0954	cn1016	pOP-EAP01871_EST_C_1_pBSK_SK	428
cl0887	ct0954	cn1016	pOP-EAP02933_EST_C_1_pBSK_SK	603
cl0888	ct0955	cn1017	pOP-CNIP00824_EST_C_1_pSK_SK	519
cl0888	ct0955	cn1017	pOP-EAP02937_EST_C_1_pBSK_SK	548
cl0889	ct0956	cn1018	pOP-CNH01393_EST_C_1_pSK_SK	483
cl0889	ct0956	cn1018	pOP-CNHP00116_EST_C_1_pSK_SK	529
cl0889	ct0956	cn1018	pOP-CNIP00384_EST_C_1_pSK_SK	304
cl0889	ct0956	cn1018	pOP-EAP02941_EST_C_1_pBSK_SK	381
cl0890	ct0957	cn1019	pOP-EAP01067_EST_C_1_pBSK_SK	520
cl0890	ct0957	cn1019	pOP-EAP02886_EST_C_1_pBSK_SK	674
cl0890	ct0957	cn1019	pOP-EAP02947_EST_C_1_pBSK_SK	701
cl0891	ct0958	cn1020	pOP-CNIP00494_EST_C_1_pSK_SK	480
cl0891	ct0959	cn1021	pOP-EAP02949_EST_C_1_pBSK_SK	658
cl0891	ct0959	cn1021	pOP-EO05531_EST_C_1_pSK_SK	452
cl0892	ct0960	cn1022	pOP-EAP01318_EST_C_1_pBSK_SK	307
cl0892	ct0960	cn1022	pOP-EAP02387_EST_C_1_pBSK_SK	498
cl0892	ct0960	cn1022	pOP-EAP02951_EST_C_1_pBSK_SK	448
cl0893	ct0961	cn1023	pOP-CNIP00030_EST_C_1_pSK_SK	409
cl0893	ct0961	cn1023	pOP-EAP02966_EST_C_1_pBSK_SK	253
cl0894	ct0962	cn1024	pOP-CNH00747_EST_C_1_pSK_SK	372
cl0894	ct0962	cn1024	pOP-CNH04623	744
cl0894	ct0962	cn1024	pOP-CNH04644	684
cl0894	ct0962	cn1024	pOP-CNH04896_EST_C_1_pSK_SK	760
cl0894	ct0962	cn1024	pOP-CNI01462_EST_C_1_pSK_SK	652
cl0894	ct0962	cn1024	pOP-EAP02967_EST_C_1_pBSK_SK	425
cl0894	ct0962	cn1024	pOP-EO06128_EST_C_1_pSK_SK	697
cl0895	ct0963	cn1025	pOP-CNIP00902_EST_C_1_pSK_SK	526
cl0895	ct0963	cn1025	pOP-EAP02974_EST_C_1_pBSK_SK	484
cl0896	ct0964	cn1026	pOP-CEO01624_EST_C_1_pSK_SK	412
cl0896	ct0964	cn1026	pOP-CNH04509	747
cl0896	ct0964	cn1026	pOP-CNI01579_EST_C_1_pSK_SK	370
cl0896	ct0965	cn1027	pOP-CEM00099_EST_C_1_pSK_SK	459
cl0896	ct0965	cn1027	pOP-CNH02895_EST_C_1_pSK_SK	604
cl0896	ct0965	cn1027	pOP-EAP02978_EST_C_1_pBSK_SK	548
cl0897	ct0966	cn1028	pOP-CNHP00081_EST_C_1_pSK_SK	578
cl0897	ct0966	cn1028	pOP-EAP02979_EST_C_1_pBSK_SK	367
cl0898	ct0967	cn1029	pOP-EAP00691_EST_C_1_pBSK_SK	526
cl0898	ct0967	cn1029	pOP-EAP02171_EST_C_1_pBSK_SK	573
cl0898	ct0967	cn1029	pOP-EAP02985_EST_C_1_pBSK_SK	428
cl0899	ct0968	cn1030	pOP-CNH02737_EST_C_1_pSK_SK	669
cl0899	ct0968	cn1030	pOP-CNH04242	549
cl0899	ct0968	cn1030	pOP-EAP02992_EST_C_1_pBSK_SK	554
cl0900	ct0969	cn1031	pOP-EAP00111_EST_C_1_pBSK_SK	499
cl0900	ct0969	cn1031	pOP-EAP02998_EST_C_1_pBSK_SK	423
cl0901	ct0970	cn1032	pOP-CNH01105_EST_C_1_pSK_SK	286
cl0901	ct0970	cn1032	pOP-EAP03114_EST_C_1_pBSK_SK	553
cl0902	ct0971	cn1033	pOP-EAP02938_EST_C_1_pBSK_SK	551
cl0902	ct0971	cn1033	pOP-EAP03117_EST_C_1_pBSK_SK	511
cl0903	ct0972	cn1034	pOP-EAP00859_EST_C_1_pBSK_SK	630
cl0903	ct0972	cn1034	pOP-EAP03123_EST_C_1_pBSK_SK	649

cl0904	ct0973	cn1035	pOP-CNH00782_EST_C_1_pSK_SK	219
cl0904	ct0973	cn1035	pOP-EAP02105_EST_C_1_pBSK_SK	402
cl0904	ct0973	cn1035	pOP-EAP03124_EST_C_1_pBSK_SK	379
cl0905	ct0974	cn1036	pOP-CNIP04026_EST_C_1_pSK_SK	412
cl0905	ct0974	cn1036	pOP-EAP03129_EST_C_1_pBSK_SK	252
cl0906	ct0975	cn1037	pOP-CEO01295	416
cl0906	ct0975	cn1037	pOP-EAP03146_EST_C_1_pBSK_SK	599
cl0906	ct0975	cn1037	pOP-EO03641_EST_C_1_pSK_SK	473
cl0906	ct0975	cn1037	pOP-EO04038_EST_C_1_pSK_SK	449
cl0906	ct0975	cn1037	pOP-EO04133_EST_C_1_pSK_SK	345
cl0906	ct0975	cn1037	pOP-EO07190_EST_C_1_pSK_SK	686
cl0907	ct0976	cn1038	pOP-CNI02192_EST_C_1_pSK_SK	355
cl0907	ct0976	cn1038	pOP-EAP03148_EST_C_1_pBSK_SK	559
cl0908	ct0977	cn1039	pOP-EAP00283_EST_C_1_pBSK_SK	521
cl0908	ct0977	cn1039	pOP-EAP03151_EST_C_1_pBSK_SK	545
cl0909	ct0978	cn1040	pOP-EAP00714_EST_C_1_pBSK_SK	578
cl0909	ct0978	cn1040	pOP-EAP01951_EST_C_1_pBSK_SK	612
cl0909	ct0978	cn1040	pOP-EAP02714_EST_C_1_pBSK_SK	585
cl0909	ct0978	cn1040	pOP-EAP03119_EST_C_1_pBSK_SK	692
cl0909	ct0978	cn1040	pOP-EAP03156_EST_C_1_pBSK_SK	566
cl0910	ct0979	cn1041	pOP-CNI01102_EST_C_1_pSK_SK	413
cl0910	ct0979	cn1041	pOP-EAP03159_EST_C_1_pBSK_SK	484
cl0911	ct0980	cn1042	pOP-CNI01218_EST_C_1_pSK_SK	230
cl0911	ct0980	cn1042	pOP-EAP03164_EST_C_1_pBSK_SK	379
cl0912	ct0981	cn1043	pOP-CNH01090_EST_C_1_pSK_SK	256
cl0912	ct0981	cn1043	pOP-CNH01907_EST_C_1_pSK_SK	702
cl0912	ct0981	cn1043	pOP-CNH02319_EST_C_1_pSK_SK	491
cl0912	ct0981	cn1043	pOP-CNH04590	155
cl0912	ct0981	cn1043	pOP-CNHP00424_EST_C_1_pSK_SK	604
cl0912	ct0981	cn1043	pOP-EAP03172_EST_C_1_pBSK_SK	488
cl0913	ct0982	cn1044	pOP-CNH00573_EST_C_1_pSK_SK	619
cl0913	ct0982	cn1044	pOP-CNH00626_EST_C_1_pSK_SK	473
cl0913	ct0982	cn1044	pOP-CNH01793_EST_C_1_pSK_SK	475
cl0913	ct0982	cn1044	pOP-EAP03174_EST_C_1_pBSK_SK	501
cl0914	ct0983	cn1045	pOP-CNH00618_EST_C_1_pSK_SK	686
cl0914	ct0983	cn1045	pOP-CNHP00188_EST_C_1_pSK_SK	667
cl0914	ct0983	cn1045	pOP-EAP03178_EST_C_1_pBSK_SK	455
cl0915	ct0984	cn1046	pOP-CNIP00814_EST_C_1_pSK_SK	209
cl0915	ct0984	cn1046	pOP-EAP01905_EST_C_1_pBSK_SK	592
cl0915	ct0984	cn1046	pOP-EAP03179_EST_C_1_pBSK_SK	546
cl0916	ct0985	cn1047	pOP-CNI01743_EST_C_1_pSK_SK	468
cl0916	ct0985	cn1047	pOP-EAP01478_EST_C_1_pBSK_SK	613
cl0916	ct0985	cn1047	pOP-EAP03186_EST_C_1_pBSK_SK	578
cl0917	ct0986	cn1048	pOP-CNH00886_EST_C_1_pSK_SK	485
cl0917	ct0986	cn1048	pOP-CNH02279_EST_C_1_pSK_SK	644
cl0917	ct0986	cn1048	pOP-CNIP04081_EST_C_1_pSK_SK	489
cl0917	ct0986	cn1048	pOP-EAP03188_EST_C_1_pBSK_SK	670
cl0918	ct0987	cn1049	pOP-CNI01840_EST_C_1_pSK_SK	275
cl0918	ct0987	cn1049	pOP-EAP02148_EST_C_1_pBSK_SK	153
cl0918	ct0987	cn1049	pOP-EAP03194_EST_C_1_pBSK_SK	230
cl0919	ct0988	cn1050	pOP-EAP01739_EST_C_1_pBSK_SK	570
cl0919	ct0988	cn1050	pOP-EAP03195_EST_C_1_pBSK_SK	646
cl0920	ct0989	cn1051	pOP-EAP00682_EST_C_1_pBSK_SK	348
cl0920	ct0989	cn1051	pOP-EAP03196_EST_C_1_pBSK_SK	610
cl0920	ct0989	cn1051	pOP-EO07958_EST_C_1_pSK_SK	632
cl0921	ct0990	cn1052	pOP-CNI02005_EST_C_1_pSK_SK	526

cl0921	ct0990	cn1052	pOP-EAP01619_EST_C_1_pBSK_SK	388
cl0921	ct0990	cn1052	pOP-EAP02917_EST_C_1_pBSK_SK	695
cl0921	ct0990	cn1052	pOP-EAP03200_EST_C_1_pBSK_SK	623
cl0922	ct0991	cn1053	pOP-CNH00578_EST_C_1_pSK_SK	687
cl0922	ct0991	cn1053	pOP-CNH00710_EST_C_1_pSK_SK	457
cl0922	ct0991	cn1053	pOP-CNH01324_EST_C_1_pSK_SK	742
cl0922	ct0991	cn1053	pOP-CNH01584_EST_C_1_pSK_SK	645
cl0922	ct0991	cn1053	pOP-CNH01994_EST_C_1_pSK_SK	539
cl0922	ct0991	cn1053	pOP-CNH03205_EST_C_1_pSK_SK	681
cl0922	ct0991	cn1053	pOP-CNH04139	554
cl0922	ct0991	cn1053	pOP-CNH04147	555
cl0922	ct0991	cn1053	pOP-CNH04907_EST_C_1_pSK_SK	602
cl0922	ct0991	cn1053	pOP-CNH05010_EST_C_1_pSK_SK	755
cl0922	ct0992	cn1054	pOP-CNH00851_EST_C_1_pSK_SK	674
cl0922	ct0992	cn1054	pOP-CNH00912_EST_C_1_pSK_SK	571
cl0922	ct0992	cn1054	pOP-CNH00936_EST_C_1_pSK_SK	571
cl0922	ct0992	cn1054	pOP-CNH01593_EST_C_1_pSK_SK	427
cl0922	ct0992	cn1054	pOP-CNH01622_EST_C_1_pSK_SK	526
cl0922	ct0992	cn1054	pOP-CNH02010_EST_C_1_pSK_SK	531
cl0922	ct0992	cn1054	pOP-CNH02200_EST_C_1_pSK_SK	291
cl0922	ct0992	cn1054	pOP-CNH02213_EST_C_1_pSK_SK	450
cl0922	ct0992	cn1054	pOP-CNH02305_EST_C_1_pSK_SK	628
cl0922	ct0992	cn1054	pOP-CNH02523_EST_C_1_pSK_SK	354
cl0922	ct0992	cn1054	pOP-CNH02967_EST_C_1_pSK_SK	602
cl0922	ct0992	cn1054	pOP-CNH03218_EST_C_1_pSK_SK	545
cl0922	ct0992	cn1054	pOP-CNH03520_EST_C_1_pSK_SK	671
cl0922	ct0992	cn1054	pOP-CNH03526_EST_C_1_pSK_SK	601
cl0922	ct0992	cn1054	pOP-CNH03585_EST_C_1_pSK_SK	500
cl0922	ct0992	cn1054	pOP-CNH03627_EST_C_1_pSK_SK	634
cl0922	ct0992	cn1054	pOP-CNH03789_EST_C_1_pSK_SK	542
cl0922	ct0992	cn1054	pOP-CNH04536	852
cl0922	ct0992	cn1054	pOP-CNH04538	768
cl0922	ct0992	cn1054	pOP-CNH04668	662
cl0922	ct0992	cn1054	pOP-CNH04688	751
cl0922	ct0992	cn1054	pOP-CNH04695	666
cl0922	ct0992	cn1054	pOP-CNH05064_EST_C_1_pSK_SK	652
cl0922	ct0992	cn1054	pOP-CNHP00153_EST_C_1_pSK_SK	357
cl0922	ct0992	cn1054	pOP-CNHP00234_EST_C_1_pSK_SK	317
cl0922	ct0992	cn1054	pOP-CNHP00325_EST_C_1_pSK_SK	694
cl0922	ct0992	cn1054	pOP-CNHP00365_EST_C_1_pSK_SK	352
cl0922	ct0992	cn1054	pOP-CNHP00503_EST_C_1_pSK_SK	603
cl0922	ct0992	cn1054	pOP-CNI01961_EST_C_1_pSK_SK	310
cl0922	ct0992	cn1054	pOP-CNIP00530_EST_C_1_pSK_SK	241
cl0922	ct0992	cn1054	pOP-CNIP00686_EST_C_1_pSK_SK	346
cl0922	ct0992	cn1054	pOP-EAP03204_EST_C_1_pBSK_SK	252
cl0922	ct0992	cn1055	pOP-CNI01151_EST_C_1_pSK_SK	525
cl0923	ct0993	cn1056	pOP-CNIP00007_EST_C_1_pSK_SK	548
cl0923	ct0993	cn1056	pOP-CNIP00955_EST_C_1_pSK_SK	562
cl0923	ct0993	cn1056	pOP-EAP03211_EST_C_1_pBSK_SK	307
cl0924	ct0994	cn1057	pOP-EAP01111_EST_C_1_pBSK_SK	262
cl0924	ct0994	cn1057	pOP-EAP03213_EST_C_1_pBSK_SK	513
cl0925	ct0995	cn1058	pOP-CNH00740_EST_C_1_pSK_SK	385
cl0925	ct0995	cn1058	pOP-CNH01522_EST_C_1_pSK_SK	540
cl0925	ct0995	cn1058	pOP-CNH01869_EST_C_1_pSK_SK	409
cl0925	ct0995	cn1058	pOP-CNH02148_EST_C_1_pSK_SK	520
cl0925	ct0995	cn1058	pOP-CNH02407_EST_C_1_pSK_SK	454

cl0925	ct0995	cn1058	pOP-CNH04628	704
cl0925	ct0995	cn1058	pOP-CNHP00049_EST_C_1_pSK_SK	577
cl0925	ct0995	cn1058	pOP-CNI01327_EST_C_1_pSK_SK	387
cl0925	ct0995	cn1058	pOP-CNIP00688_EST_C_1_pSK_SK	596
cl0925	ct0995	cn1058	pOP-CNIP00992_EST_C_1_pSK_SK	382
cl0925	ct0995	cn1058	pOP-EAP03218_EST_C_1_pBSK_SK	624
cl0926	ct0996	cn1059	pOP-EAP03219_EST_C_1_pBSK_SK	570
cl0926	ct0996	cn1060	pOP-CEO01080_EST_C_1_pSK_SK	407
cl0927	ct0997	cn1061	pOP-CNI01372_EST_C_1_pSK_SK	291
cl0927	ct0997	cn1061	pOP-CNIP00612_EST_C_1_pSK_SK	544
cl0927	ct0997	cn1061	pOP-EAP03235_EST_C_1_pBSK_SK	639
cl0928	ct0998	cn1062	pOP-CNI01514_EST_C_1_pSK_SK	446
cl0928	ct0998	cn1062	pOP-EAP03237_EST_C_1_pBSK_SK	431
cl0929	ct0999	cn1063	pOP-EAP00799_EST_C_1_pBSK_SK	411
cl0929	ct0999	cn1063	pOP-EAP03242_EST_C_1_pBSK_SK	386
cl0930	ct1000	cn1064	pOP-CNIP00182_EST_C_1_pSK_SK	502
cl0930	ct1000	cn1064	pOP-EAP03262_EST_C_1_pBSK_SK	489
cl0930	ct1000	cn1064	pOP-EN00494_EST_C_1_pSK_SK	509
cl0931	ct1001	cn1065	pOP-CNH03027_EST_C_1_pSK_SK	536
cl0931	ct1001	cn1065	pOP-CNHP00249_EST_C_1_pSK_SK	518
cl0931	ct1001	cn1065	pOP-CNIP00042_EST_C_1_pSK_SK	451
cl0931	ct1001	cn1065	pOP-EAP00526_EST_C_1_pBSK_SK	267
cl0931	ct1001	cn1065	pOP-EAP01003_EST_C_1_pBSK_SK	181
cl0931	ct1001	cn1065	pOP-EAP01627_EST_C_1_pBSK_SK	380
cl0931	ct1001	cn1065	pOP-EAP01732_EST_C_1_pBSK_SK	492
cl0931	ct1001	cn1065	pOP-EAP02060_EST_C_1_pBSK_SK	381
cl0931	ct1001	cn1065	pOP-EAP02844_EST_C_1_pBSK_SK	632
cl0931	ct1001	cn1065	pOP-EAP03270_EST_C_1_pBSK_SK	150
cl0931	ct1001	cn1065	pOP-EO02755_EST_C_1_pSK_SK	443
cl0931	ct1001	cn1066	pOP-CEO00896_EST_C_1_pSK_SK	544
cl0932	ct1002	cn1067	pOP-EAP02301_EST_C_1_pBSK_SK	524
cl0932	ct1002	cn1067	pOP-EAP03299_EST_C_1_pBSK_SK	502
cl0933	ct1003	cn1068	pOP-CNHP00506_EST_C_1_pSK_SK	607
cl0933	ct1003	cn1068	pOP-EAP03305_EST_C_1_pBSK_SK	625
cl0934	ct1004	cn1069	pOP-EAP01706_EST_C_1_pBSK_SK	224
cl0934	ct1004	cn1069	pOP-EAP03307_EST_C_1_pBSK_SK	588
cl0935	ct1005	cn1070	pOP-CEO01268	599
cl0935	ct1005	cn1070	pOP-EAP03308_EST_C_1_pBSK_SK	486
cl0936	ct1006	cn1071	pOP-EAP03311_EST_C_1_pBSK_SK	627
cl0936	ct1006	cn1071	pOP-EO05162_EST_C_1_pSK_SK	484
cl0937	ct1007	cn1072	pOP-CNH01434_EST_C_1_pSK_SK	609
cl0937	ct1007	cn1072	pOP-CNIP00815_EST_C_1_pSK_SK	638
cl0937	ct1007	cn1072	pOP-EAP03327_EST_C_1_pBSK_SK	634
cl0938	ct1008	cn1073	pOP-CNH00781_EST_C_1_pSK_SK	323
cl0938	ct1008	cn1073	pOP-CNH04514	769
cl0938	ct1008	cn1073	pOP-CNH04871_EST_C_1_pSK_SK	824
cl0938	ct1008	cn1073	pOP-EAP01521_EST_C_1_pBSK_SK	602
cl0938	ct1008	cn1073	pOP-EAP03343_EST_C_1_pBSK_SK	552
cl0938	ct1008	cn1074	pOP-CNI02102_EST_C_1_pSK_SK	506
cl0939	ct1009	cn1075	pOP-EAP00910_EST_C_1_pBSK_SK	228
cl0939	ct1009	cn1075	pOP-EAP03355_EST_C_1_pBSK_SK	454
cl0940	ct1010	cn1076	pOP-CNIP00974_EST_C_1_pSK_SK	343
cl0940	ct1010	cn1076	pOP-EAP03359_EST_C_1_pBSK_SK	597
cl0941	ct1011	cn1077	pOP-EAP01897_EST_C_1_pBSK_SK	489
cl0941	ct1011	cn1077	pOP-EAP03364_EST_C_1_pBSK_SK	457
cl0941	ct1012	cn1078	pOP-CNH04740_EST_C_1_pSK_SK	554

cl0941	ct1012	cn1078	pOP-EAP00273_EST_C_1_pBSK_SK	598
cl0941	ct1012	cn1078	pOP-EAP00523_EST_C_1_pBSK_SK	662
cl0941	ct1012	cn1078	pOP-EO04092_EST_C_1_pSK_SK	530
cl0942	ct1013	cn1079	pOP-EAP03328_EST_C_1_pBSK_SK	121
cl0942	ct1013	cn1079	pOP-EAP03368_EST_C_1_pBSK_SK	121
cl0943	ct1014	cn1080	pOP-EAP03385_EST_C_1_pBSK_SK	314
cl0943	ct1014	cn1080	pOP-EO05161_EST_C_1_pSK_SK	484
cl0944	ct1015	cn1081	pOP-CNI01132_EST_C_1_pSK_SK	289
cl0944	ct1015	cn1081	pOP-EAP03401_EST_C_1_pBSK_SK	348
cl0945	ct1016	cn1082	pOP-CNH00554_EST_C_1_pSK_SK	595
cl0945	ct1016	cn1082	pOP-CNH03602_EST_C_1_pSK_SK	507
cl0945	ct1016	cn1082	pOP-CNH03610_EST_C_1_pSK_SK	349
cl0945	ct1016	cn1082	pOP-CNH03612_EST_C_1_pSK_SK	548
cl0945	ct1016	cn1082	pOP-CNH05067_EST_C_1_pSK_SK	642
cl0945	ct1016	cn1082	pOP-EAP03425_EST_C_1_pBSK_SK	432
cl0945	ct1017	cn1083	pOP-CNH00687_EST_C_1_pSK_SK	445
cl0945	ct1017	cn1083	pOP-CNH02972_EST_C_1_pSK_SK	590
cl0945	ct1017	cn1083	pOP-CNH03603_EST_C_1_pSK_SK	359
cl0945	ct1017	cn1083	pOP-CNHP00525_EST_C_1_pSK_SK	595
cl0945	ct1017	cn1083	pOP-CNIP00777_EST_C_1_pSK_SK	328
cl0945	ct1017	cn1083	pOP-EAP01850_EST_C_1_pBSK_SK	490
cl0946	ct1018	cn1084	pOP-CNH03427_EST_C_1_pSK_SK	498
cl0946	ct1018	cn1084	pOP-CNH04569	621
cl0946	ct1018	cn1084	pOP-EAP03426_EST_C_1_pBSK_SK	410
cl0947	ct1019	cn1085	pOP-EAP01098_EST_C_1_pBSK_SK	244
cl0947	ct1019	cn1085	pOP-EAP03428_EST_C_1_pBSK_SK	352
cl0948	ct1020	cn1086	pOP-EAP03321_EST_C_1_pBSK_SK	504
cl0948	ct1020	cn1086	pOP-EAP03434_EST_C_1_pBSK_SK	482
cl0949	ct1021	cn1087	pOP-CNIP00411_EST_C_1_pSK_SK	564
cl0949	ct1021	cn1087	pOP-EAP03443_EST_C_1_pBSK_SK	602
cl0950	ct1022	cn1088	pOP-EAP00640_EST_C_1_pBSK_SK	403
cl0950	ct1022	cn1088	pOP-EAP03462_EST_C_1_pBSK_SK	479
cl0951	ct1023	cn1089	pOP-EAP02185_EST_C_1_pBSK_SK	459
cl0951	ct1023	cn1089	pOP-EAP03465_EST_C_1_pBSK_SK	600
cl0952	ct1024	cn1090	pOP-CNHP00401_EST_C_1_pSK_SK	527
cl0952	ct1024	cn1090	pOP-EAP03467_EST_C_1_pBSK_SK	373
cl0953	ct1025	cn1091	pOP-EAP03475_EST_C_1_pBSK_SK	288
cl0953	ct1025	cn1091	pOP-EO03631_EST_C_1_pSK_SK	434
cl0954	ct1026	cn1092	pOP-CNI01635_EST_C_1_pSK_SK	179
cl0954	ct1026	cn1092	pOP-EAP03479_EST_C_1_pBSK_SK	374
cl0955	ct1027	cn1093	pOP-CNIP00055_EST_C_1_pSK_SK	640
cl0955	ct1027	cn1094	pOP-CEO02826_EST_C_1_pSK_SK	383
cl0955	ct1027	cn1095	pOP-EAP03480_EST_C_1_pBSK_SK	296
cl0955	ct1027	cn1095	pOP-EO02567_EST_C_1_pSK_SK	427
cl0956	ct1028	cn1096	pOP-EAP00063_EST_C_1_pBSK_SK	486
cl0956	ct1028	cn1096	pOP-EAP01130_EST_C_1_pBSK_SK	304
cl0956	ct1028	cn1096	pOP-EAP02734_EST_C_1_pBSK_SK	651
cl0956	ct1028	cn1096	pOP-EAP03562_EST_C_1_pBSK_SK	236
cl0957	ct1029	cn1097	pOP-CNI01233_EST_C_1_pSK_SK	425
cl0957	ct1029	cn1097	pOP-EAP03564_EST_C_1_pBSK_SK	192
cl0958	ct1030	cn1098	pOP-CNH01276_EST_C_1_pSK_SK	563
cl0958	ct1030	cn1098	pOP-CNI01186_EST_C_1_pSK_SK	281
cl0958	ct1030	cn1098	pOP-EAP03566_EST_C_1_pBSK_SK	247
cl0958	ct1030	cn1098	pOP-EO05622_EST_C_1_pSK_SK	440
cl0958	ct1030	cn1098	pOP-EO05738_EST_C_1_pSK_SK	514
cl0958	ct1030	cn1099	pOP-CEO02899_EST_C_1_pSK_SK	628

cl0959	ct1031	cn1100	pOP-CNH03192_EST_C_1_pSK_SK	559
cl0959	ct1031	cn1100	pOP-CNH04893_EST_C_1_pSK_SK	595
cl0959	ct1031	cn1100	pOP-CNHP00405_EST_C_1_pSK_SK	319
cl0959	ct1031	cn1100	pOP-CNIP00638_EST_C_1_pSK_SK	363
cl0959	ct1031	cn1100	pOP-EAP02376_EST_C_1_pBSK_SK	763
cl0959	ct1031	cn1100	pOP-EAP03580_EST_C_1_pBSK_SK	326
cl0960	ct1032	cn1101	pOP-CNIP00127_EST_C_1_pSK_SK	644
cl0960	ct1032	cn1101	pOP-EAP03581_EST_C_1_pBSK_SK	250
cl0961	ct1033	cn1102	pOP-EAP03577_EST_C_1_pBSK_SK	490
cl0961	ct1033	cn1102	pOP-EAP03588_EST_C_1_pBSK_SK	525
cl0962	ct1034	cn1103	pOP-CNHP00173_EST_C_1_pSK_SK	473
cl0962	ct1034	cn1103	pOP-CNHP00242_EST_C_1_pSK_SK	531
cl0962	ct1034	cn1103	pOP-EAP00110_EST_C_1_pBSK_SK	627
cl0962	ct1034	cn1103	pOP-EAP03591_EST_C_1_pBSK_SK	529
cl0963	ct1035	cn1104	pOP-CNI01639_EST_C_1_pSK_SK	219
cl0963	ct1035	cn1104	pOP-EAP03595_EST_C_1_pBSK_SK	231
cl0964	ct1036	cn1105	pOP-CNI01194_EST_C_1_pSK_SK	624
cl0964	ct1036	cn1105	pOP-CNIP00642_EST_C_1_pSK_SK	578
cl0964	ct1036	cn1105	pOP-EAP00419_EST_C_1_pBSK_SK	256
cl0964	ct1036	cn1105	pOP-EAP03603_EST_C_1_pBSK_SK	398
cl0965	ct1037	cn1106	pOP-CNI01642_EST_C_1_pSK_SK	363
cl0965	ct1037	cn1106	pOP-EAP03609_EST_C_1_pBSK_SK	531
cl0966	ct1038	cn1107	pOP-CNI01799_EST_C_1_pSK_SK	284
cl0966	ct1038	cn1107	pOP-EAP03619_EST_C_1_pBSK_SK	234
cl0967	ct1039	cn1108	pOP-CNI01696_EST_C_1_pSK_SK	475
cl0967	ct1039	cn1108	pOP-EAP03635_EST_C_1_pBSK_SK	551
cl0968	ct1040	cn1109	pOP-EAP03640_EST_C_1_pBSK_SK	607
cl0968	ct1040	cn1109	pOP-EO06519_EST_C_1_pSK_SK	704
cl0969	ct1041	cn1110	pOP-CAP00237_EST_C_1_pBSK_SK	597
cl0969	ct1041	cn1110	pOP-CEO00527_EST_C_1_pSK_SK	279
cl0969	ct1041	cn1110	pOP-CEO02404_EST_C_1_pSK_SK	346
cl0969	ct1041	cn1110	pOP-CNI01519_EST_C_1_pSK_SK	157
cl0969	ct1041	cn1110	pOP-CNI01786_EST_C_1_pSK_SK	223
cl0969	ct1041	cn1110	pOP-EAP01299_EST_C_1_pBSK_SK	549
cl0969	ct1041	cn1110	pOP-EAP01300_EST_C_1_pBSK_SK	291
cl0969	ct1041	cn1110	pOP-EAP01975_EST_C_1_pBSK_SK	223
cl0969	ct1041	cn1110	pOP-EAP02370_EST_C_1_pBSK_SK	398
cl0969	ct1041	cn1110	pOP-EAP03642_EST_C_1_pBSK_SK	411
cl0970	ct1042	cn1111	pOP-EAP03639_EST_C_1_pBSK_SK	616
cl0970	ct1042	cn1111	pOP-EAP03645_EST_C_1_pBSK_SK	635
cl0970	ct1042	cn1111	pOP-EO05826_EST_C_1_pSK_SK	630
cl0971	ct1043	cn1112	pOP-CNH01710_EST_C_1_pSK_SK	561
cl0971	ct1044	cn1113	pOP-CNH01061_EST_C_1_pSK_SK	590
cl0971	ct1044	cn1113	pOP-CNH03056_EST_C_1_pSK_SK	611
cl0971	ct1044	cn1113	pOP-CNH03073_EST_C_1_pSK_SK	639
cl0971	ct1044	cn1113	pOP-CNH03555_EST_C_1_pSK_SK	511
cl0971	ct1044	cn1113	pOP-CNH03813_EST_C_1_pSK_SK	499
cl0971	ct1044	cn1113	pOP-CNIP00793_EST_C_1_pSK_SK	447
cl0971	ct1044	cn1113	pOP-EAP03646_EST_C_1_pBSK_SK	480
cl0971	ct1044	cn1113	pOP-EN00468_EST_C_1_pSK_SK	516
cl0971	ct1044	cn1114	pOP-CNH01933_EST_C_1_pSK_SK	692
cl0972	ct1045	cn1115	pOP-EAP03649_EST_C_1_pBSK_SK	516
cl0972	ct1045	cn1115	pOP-EO06573_EST_C_1_pSK_SK	705
cl0973	ct1046	cn1116	pOP-EAP01349_EST_C_1_pBSK_SK	398
cl0973	ct1046	cn1116	pOP-EAP03460_EST_C_1_pBSK_SK	239
cl0973	ct1046	cn1116	pOP-EAP03650_EST_C_1_pBSK_SK	191

cl0974	ct1047	cn1117	pOP-CNHP00238_EST_C_1_pSK_SK	294
cl0974	ct1047	cn1117	pOP-CNI01606_EST_C_1_pSK_SK	174
cl0974	ct1047	cn1117	pOP-CNI01644_EST_C_1_pSK_SK	440
cl0974	ct1047	cn1117	pOP-EAP00554_EST_C_1_pBSK_SK	457
cl0974	ct1047	cn1117	pOP-EAP03652_EST_C_1_pBSK_SK	371
cl0974	ct1047	cn1117	pOP-EO02951_EST_C_1_pSK_SK	441
cl0974	ct1047	cn1117	pOP-EO04352_EST_C_1_pSK_SK	527
cl0975	ct1048	cn1118	pOP-CNH02085_EST_C_1_pSK_SK	609
cl0975	ct1048	cn1118	pOP-CNH04585	693
cl0975	ct1048	cn1118	pOP-EAP03653_EST_C_1_pBSK_SK	477
cl0976	ct1049	cn1119	pOP-CNH00612_EST_C_1_pSK_SK	672
cl0976	ct1049	cn1119	pOP-CNH00742_EST_C_1_pSK_SK	388
cl0976	ct1049	cn1119	pOP-CNH02182_EST_C_1_pSK_SK	507
cl0976	ct1049	cn1119	pOP-EAP03679_EST_C_1_pBSK_SK	511
cl0977	ct1050	cn1120	pOP-CEO01947_EST_C_1_pSK_SK	196
cl0977	ct1050	cn1120	pOP-CNH00597_EST_C_1_pSK_SK	540
cl0977	ct1050	cn1120	pOP-EAP00069_EST_C_1_pBSK_SK	530
cl0977	ct1050	cn1120	pOP-EAP00557_EST_C_1_pBSK_SK	345
cl0977	ct1050	cn1120	pOP-EAP01282_EST_C_1_pBSK_SK	432
cl0977	ct1050	cn1120	pOP-EAP01374_EST_C_1_pBSK_SK	349
cl0977	ct1050	cn1120	pOP-EAP02337_EST_C_1_pBSK_SK	609
cl0977	ct1050	cn1120	pOP-EAP02943_EST_C_1_pBSK_SK	664
cl0977	ct1050	cn1120	pOP-EAP03155_EST_C_1_pBSK_SK	444
cl0977	ct1050	cn1120	pOP-EAP03687_EST_C_1_pBSK_SK	434
cl0977	ct1050	cn1120	pOP-EAP03832_EST_C_1_pBSK_SK	604
cl0977	ct1050	cn1120	pOP-EO02057_EST_C_1_pSK_SK	628
cl0977	ct1050	cn1120	pOP-EO04388_EST_C_1_pSK_SK	521
cl0977	ct1050	cn1121	pOP-CEO01627_EST_C_1_pSK_SK	447
cl0978	ct1051	cn1122	pOP-CNI01841_EST_C_1_pSK_SK	516
cl0978	ct1051	cn1122	pOP-EAP03690_EST_C_1_pBSK_SK	296
cl0979	ct1052	cn1123	pOP-CNH01124_EST_C_1_pSK_SK	421
cl0979	ct1052	cn1123	pOP-CNH01218_EST_C_1_pSK_SK	529
cl0979	ct1052	cn1123	pOP-CNH01536_EST_C_1_pSK_SK	558
cl0979	ct1052	cn1123	pOP-CNH02175_EST_C_1_pSK_SK	522
cl0979	ct1052	cn1123	pOP-EAP03706_EST_C_1_pBSK_SK	604
cl0980	ct1053	cn1124	pOP-EAP02707_EST_C_1_pBSK_SK	723
cl0980	ct1053	cn1125	pOP-CNH00670_EST_C_1_pSK_SK	524
cl0980	ct1053	cn1125	pOP-CNHP00279_EST_C_1_pSK_SK	600
cl0980	ct1053	cn1125	pOP-EAP03707_EST_C_1_pBSK_SK	587
cl0981	ct1054	cn1126	pOP-CNHP00224_EST_C_1_pSK_SK	445
cl0981	ct1054	cn1126	pOP-EAP02374_EST_C_1_pBSK_SK	556
cl0981	ct1054	cn1126	pOP-EAP03714_EST_C_1_pBSK_SK	312
cl0982	ct1055	cn1127	pOP-EAP01815_EST_C_1_pBSK_SK	463
cl0982	ct1055	cn1127	pOP-EAP03724_EST_C_1_pBSK_SK	440
cl0983	ct1056	cn1128	pOP-EAP03340_EST_C_1_pBSK_SK	616
cl0983	ct1056	cn1128	pOP-EAP03726_EST_C_1_pBSK_SK	322
cl0984	ct1057	cn1129	pOP-EAP03732_EST_C_1_pBSK_SK	497
cl0984	ct1057	cn1129	pOP-EO04261_EST_C_1_pSK_SK	380
cl0985	ct1058	cn1130	pOP-CNI01901_EST_C_1_pSK_SK	461
cl0985	ct1058	cn1130	pOP-EAP03735_EST_C_1_pBSK_SK	440
cl0986	ct1059	cn1131	pOP-EAP03183_EST_C_1_pBSK_SK	548
cl0986	ct1059	cn1131	pOP-EAP03736_EST_C_1_pBSK_SK	330
cl0987	ct1060	cn1132	pOP-CNH02280_EST_C_1_pSK_SK	538
cl0987	ct1060	cn1132	pOP-CNHP00086_EST_C_1_pSK_SK	615
cl0987	ct1060	cn1132	pOP-EAP02965_EST_C_1_pBSK_SK	409
cl0987	ct1060	cn1132	pOP-EAP03738_EST_C_1_pBSK_SK	522

cl0987	ct1060	cn1132	pOP-EO05931_EST_C_1_pSK_SK	475
cl0988	ct1061	cn1133	pOP-CEO01519_EST_C_1_pSK_SK	126
cl0988	ct1061	cn1133	pOP-EAP02261_EST_C_1_pBSK_SK	577
cl0988	ct1061	cn1133	pOP-EAP02749_EST_C_1_pBSK_SK	567
cl0988	ct1061	cn1133	pOP-EAP03753_EST_C_1_pBSK_SK	459
cl0989	ct1062	cn1134	pOP-EAP01134_EST_C_1_pBSK_SK	116
cl0989	ct1062	cn1134	pOP-EAP03769_EST_C_1_pBSK_SK	421
cl0990	ct1063	cn1135	pOP-CNH04626	695
cl0990	ct1064	cn1136	pOP-CNH00550_EST_C_1_pSK_SK	657
cl0990	ct1064	cn1136	pOP-CNH00691_EST_C_1_pSK_SK	610
cl0990	ct1064	cn1136	pOP-CNH00983_EST_C_1_pSK_SK	578
cl0990	ct1064	cn1136	pOP-CNIP01004_EST_C_1_pSK_SK	766
cl0990	ct1064	cn1136	pOP-EAP03771_EST_C_1_pBSK_SK	223
cl0991	ct1065	cn1137	pOP-CNI01877_EST_C_1_pSK_SK	345
cl0991	ct1065	cn1137	pOP-EAP03776_EST_C_1_pBSK_SK	286
cl0992	ct1066	cn1138	pOP-EAP03239_EST_C_1_pBSK_SK	204
cl0992	ct1066	cn1138	pOP-EAP03781_EST_C_1_pBSK_SK	275
cl0993	ct1067	cn1139	pOP-CNIP00671_EST_C_1_pSK_SK	278
cl0993	ct1067	cn1139	pOP-EAP03782_EST_C_1_pBSK_SK	350
cl0994	ct1068	cn1140	pOP-CNH00763_EST_C_1_pSK_SK	397
cl0994	ct1068	cn1140	pOP-EAP03790_EST_C_1_pBSK_SK	597
cl0995	ct1069	cn1141	pOP-EAP03215_EST_C_1_pBSK_SK	612
cl0995	ct1069	cn1141	pOP-EAP03807_EST_C_1_pBSK_SK	262
cl0996	ct1070	cn1142	pOP-CNH01684_EST_C_1_pSK_SK	686
cl0996	ct1070	cn1142	pOP-EAP03473_EST_C_1_pBSK_SK	447
cl0996	ct1070	cn1142	pOP-EAP03810_EST_C_1_pBSK_SK	624
cl0996	ct1070	cn1142	pOP-EN00328_EST_C_1_pSK_SK	504
cl0997	ct1071	cn1143	pOP-EAP00178_EST_C_1_pBSK_SK	523
cl0997	ct1071	cn1143	pOP-EAP03818_EST_C_1_pBSK_SK	297
cl0997	ct1071	cn1143	pOP-EO06022_EST_C_1_pSK_SK	467
cl0998	ct1072	cn1144	pOP-CNI02016_EST_C_1_pSK_SK	333
cl0998	ct1072	cn1144	pOP-EAP03820_EST_C_1_pBSK_SK	761
cl0999	ct1073	cn1145	pOP-CNI01103_EST_C_1_pSK_SK	424
cl0999	ct1073	cn1145	pOP-EAP03821_EST_C_1_pBSK_SK	398
cl1000	ct1074	cn1146	pOP-EAP00568_EST_C_1_pBSK_SK	398
cl1000	ct1075	cn1147	pOP-CEO01513_EST_C_1_pSK_SK	411
cl1000	ct1075	cn1147	pOP-EAP01434_EST_C_1_pBSK_SK	303
cl1000	ct1076	cn1148	pOP-CNHP00160_EST_C_1_pSK_SK	289
cl1000	ct1076	cn1148	pOP-EN00906_EST_C_1_pSK_SK	512
cl1000	ct1076	cn1148	pOP-EO03353_EST_C_1_pSK_SK	463
cl1000	ct1076	cn1148	pOP-EO04721_EST_C_1_pSK_SK	520
cl1000	ct1076	cn1148	pOP-EO04723_EST_C_1_pSK_SK	512
cl1000	ct1077	cn1149	pOP-CEO00599_EST_C_1_pSK_SK	239
cl1000	ct1077	cn1149	pOP-CEO02051_EST_C_1_pSK_SK	333
cl1000	ct1077	cn1149	pOP-CEO02663_EST_C_1_pSK_SK	458
cl1000	ct1077	cn1149	pOP-CNH00801_EST_C_1_pSK_SK	506
cl1000	ct1077	cn1149	pOP-CNH02334_EST_C_1_pSK_SK	479
cl1000	ct1077	cn1149	pOP-CNH02926_EST_C_1_pSK_SK	584
cl1000	ct1077	cn1149	pOP-CNI01510_EST_C_1_pSK_SK	627
cl1000	ct1077	cn1149	pOP-CNI02246_EST_C_1_pSK_SK	752
cl1000	ct1077	cn1149	pOP-EAP00137_EST_C_1_pBSK_SK	462
cl1000	ct1077	cn1149	pOP-EAP00525_EST_C_1_pBSK_SK	608
cl1000	ct1077	cn1149	pOP-EAP00542_EST_C_1_pBSK_SK	535
cl1000	ct1077	cn1149	pOP-EAP01114_EST_C_1_pBSK_SK	512
cl1000	ct1077	cn1149	pOP-EAP01269_EST_C_1_pBSK_SK	292
cl1000	ct1077	cn1149	pOP-EAP01567_EST_C_1_pBSK_SK	468

cl1000	ct1077	cn1149	pOP-EAP01709_EST_C_1_pBSK_SK	380
cl1000	ct1077	cn1149	pOP-EAP01900_EST_C_1_pBSK_SK	648
cl1000	ct1077	cn1149	pOP-EAP02174_EST_C_1_pBSK_SK	661
cl1000	ct1077	cn1149	pOP-EAP02902_EST_C_1_pBSK_SK	693
cl1000	ct1077	cn1149	pOP-EAP03220_EST_C_1_pBSK_SK	621
cl1000	ct1077	cn1149	pOP-EAP03349_EST_C_1_pBSK_SK	481
cl1000	ct1077	cn1149	pOP-EAP03351_EST_C_1_pBSK_SK	545
cl1000	ct1077	cn1149	pOP-EAP03393_EST_C_1_pBSK_SK	616
cl1000	ct1077	cn1149	pOP-EAP03823_EST_C_1_pBSK_SK	407
cl1000	ct1077	cn1149	pOP-EAP03858_EST_C_1_pBSK_SK	594
cl1000	ct1077	cn1149	pOP-EAP05047_EST_C_1_pBSK_SK	627
cl1000	ct1077	cn1149	pOP-ENP00016_EST_C_1_pSK_SK	348
cl1000	ct1077	cn1149	pOP-EO04411_EST_C_1_pSK_SK	498
cl1001	ct1078	cn1150	pOP-EAP03825_EST_C_1_pBSK_SK	554
cl1001	ct1078	cn1150	pOP-EAP03826_EST_C_1_pBSK_SK	534
cl1002	ct1079	cn1151	pOP-EAP00906_EST_C_1_pBSK_SK	397
cl1002	ct1079	cn1151	pOP-EAP00948_EST_C_1_pBSK_SK	225
cl1002	ct1079	cn1151	pOP-EAP03844_EST_C_1_pBSK_SK	632
cl1003	ct1080	cn1152	pOP-EAP03848_EST_C_1_pBSK_SK	338
cl1003	ct1080	cn1152	pOP-EO06159_EST_C_1_pSK_SK	523
cl1004	ct1081	cn1153	pOP-EAP00349_EST_C_1_pBSK_SK	286
cl1004	ct1081	cn1153	pOP-EAP01305_EST_C_1_pBSK_SK	337
cl1004	ct1081	cn1153	pOP-EAP01572_EST_C_1_pBSK_SK	497
cl1004	ct1081	cn1153	pOP-EAP03852_EST_C_1_pBSK_SK	557
cl1005	ct1082	cn1154	pOP-CNH01088_EST_C_1_pSK_SK	489
cl1005	ct1082	cn1154	pOP-CNH01386_EST_C_1_pSK_SK	721
cl1005	ct1082	cn1154	pOP-CNH02150_EST_C_1_pSK_SK	561
cl1005	ct1082	cn1154	pOP-CNH03517_EST_C_1_pSK_SK	536
cl1005	ct1082	cn1154	pOP-CNH03759_EST_C_1_pSK_SK	430
cl1005	ct1082	cn1154	pOP-EAP03860_EST_C_1_pBSK_SK	575
cl1006	ct1083	cn1155	pOP-CEO01357	159
cl1006	ct1083	cn1155	pOP-EAP01648_EST_C_1_pBSK_SK	632
cl1006	ct1083	cn1155	pOP-EAP03861_EST_C_1_pBSK_SK	473
cl1007	ct1084	cn1156	pOP-CNI01125_EST_C_1_pSK_SK	326
cl1007	ct1084	cn1156	pOP-CNI01126_EST_C_1_pSK_SK	326
cl1007	ct1084	cn1156	pOP-EAP03862_EST_C_1_pBSK_SK	496
cl1008	ct1085	cn1157	pOP-EAP00862_EST_C_1_pBSK_SK	653
cl1008	ct1085	cn1157	pOP-EAP01001_EST_C_1_pBSK_SK	661
cl1008	ct1085	cn1158	pOP-EAP03876_EST_C_1_pBSK_SK	600
cl1009	ct1086	cn1159	pOP-CNH00732_EST_C_1_pSK_SK	658
cl1009	ct1086	cn1159	pOP-CNH00795_EST_C_1_pSK_SK	531
cl1009	ct1086	cn1159	pOP-CNHP00098_EST_C_1_pSK_SK	352
cl1009	ct1086	cn1159	pOP-EAP05000_EST_C_1_pBSK_SK	602
cl1010	ct1087	cn1160	pOP-EAP01888_EST_C_1_pBSK_SK	538
cl1010	ct1087	cn1160	pOP-EAP05011_EST_C_1_pBSK_SK	434
cl1011	ct1088	cn1161	pOP-EAP05013_EST_C_1_pBSK_SK	528
cl1011	ct1088	cn1161	pOP-EAP05014_EST_C_1_pBSK_SK	455
cl1012	ct1089	cn1162	pOP-EAP01828_EST_C_1_pBSK_SK	728
cl1012	ct1089	cn1162	pOP-EAP05020_EST_C_1_pBSK_SK	580
cl1013	ct1090	cn1163	pOP-EAP05025_EST_C_1_pBSK_SK	588
cl1013	ct1090	cn1164	pOP-CNH00857_EST_C_1_pSK_SK	542
cl1014	ct1091	cn1165	pOP-EAP03315_EST_C_1_pBSK_SK	429
cl1014	ct1091	cn1165	pOP-EAP05029_EST_C_1_pBSK_SK	374
cl1015	ct1092	cn1166	pOP-CNI01482_EST_C_1_pSK_SK	386
cl1015	ct1092	cn1166	pOP-CNI01675_EST_C_1_pSK_SK	139
cl1015	ct1092	cn1166	pOP-EAP05031_EST_C_1_pBSK_SK	615

cl1016	ct1093	cn1167	pOP-EAP05016_EST_C_1_pBSK_SK	370
cl1016	ct1093	cn1167	pOP-EAP05050_EST_C_1_pBSK_SK	379
cl1017	ct1094	cn1168	pOP-EAP01872_EST_C_1_pBSK_SK	673
cl1017	ct1094	cn1168	pOP-EAP05055_EST_C_1_pBSK_SK	583
cl1018	ct1095	cn1169	pOP-EAP00681_EST_C_1_pBSK_SK	294
cl1018	ct1095	cn1169	pOP-EBP03103_EST_C_1_pBSK_M13F	130
cl1019	ct1096	cn1170	pOP-CNH03031_EST_C_1_pSK_SK	382
cl1019	ct1096	cn1170	pOP-EBP03108_EST_C_1_pBSK_M13F	246
cl1020	ct1097	cn1171	pOP-EAP02007_EST_C_1_pBSK_SK	200
cl1020	ct1097	cn1171	pOP-EBP03109_EST_C_1_pBSK_M13F	233
cl1021	ct1098	cn1172	pOP-EN00100_EST_C_1_pSK_SK	320
cl1021	ct1098	cn1172	pOP-EO04096_EST_C_1_pSK_SK	359
cl1021	ct1098	cn1172	pOP-EO04202_EST_C_1_pSK_SK	301
cl1021	ct1098	cn1172	pOP-EO08242_EST_C_1_pSK_SK	253
cl1022	ct1099	cn1173	pOP-CNH00607_EST_C_1_pSK_SK	605
cl1022	ct1099	cn1173	pOP-CNH01576_EST_C_1_pSK_SK	608
cl1022	ct1099	cn1173	pOP-EAP00754_EST_C_1_pBSK_SK	562
cl1022	ct1099	cn1173	pOP-EN00108_EST_C_1_pSK_SK	531
cl1023	ct1100	cn1174	pOP-CNIP00563_EST_C_1_pSK_SK	240
cl1023	ct1100	cn1174	pOP-EN00114_EST_C_1_pSK_SK	589
cl1023	ct1100	cn1174	pOP-EO05367_EST_C_1_pSK_SK	521
cl1024	ct1101	cn1175	pOP-CNH00574_EST_C_1_pSK_SK	651
cl1024	ct1101	cn1175	pOP-CNH04685	578
cl1024	ct1101	cn1175	pOP-EN00158_EST_C_1_pSK_SK	513
cl1024	ct1101	cn1176	pOP-CNH05088_EST_C_1_pSK_SK	749
cl1025	ct1102	cn1177	pOP-EN00163_EST_C_1_pSK_SK	491
cl1025	ct1102	cn1177	pOP-EN00197_EST_C_1_pSK_SK	454
cl1026	ct1103	cn1178	pOP-EN00167_EST_C_1_pSK_SK	481
cl1026	ct1103	cn1178	pOP-EN00249_EST_C_1_pSK_SK	568
cl1027	ct1104	cn1179	pOP-CNH02428_EST_C_1_pSK_SK	487
cl1027	ct1104	cn1179	pOP-CNI01084_EST_C_1_pSK_SK	674
cl1027	ct1104	cn1179	pOP-EN00299_EST_C_1_pSK_SK	556
cl1028	ct1105	cn1180	pOP-EN00316_EST_C_1_pSK_SK	542
cl1028	ct1105	cn1180	pOP-EN00317_EST_C_1_pSK_SK	567
cl1029	ct1106	cn1181	pOP-EN00387_EST_C_1_pSK_SK	496
cl1029	ct1106	cn1181	pOP-EN00392_EST_C_1_pSK_SK	496
cl1030	ct1107	cn1182	pOP-EN00425_EST_C_1_pSK_SK	546
cl1030	ct1107	cn1182	pOP-EN00426_EST_C_1_pSK_SK	461
cl1031	ct1108	cn1183	pOP-EN00126_EST_C_1_pSK_SK	600
cl1031	ct1108	cn1183	pOP-EN00505_EST_C_1_pSK_SK	482
cl1032	ct1109	cn1184	pOP-EN00311_EST_C_1_pSK_SK	462
cl1032	ct1109	cn1184	pOP-EN00312_EST_C_1_pSK_SK	565
cl1032	ct1109	cn1184	pOP-EN00521_EST_C_1_pSK_SK	500
cl1033	ct1110	cn1185	pOP-EN00643_EST_C_1_pSK_SK	570
cl1033	ct1110	cn1185	pOP-EN00658_EST_C_1_pSK_SK	570
cl1034	ct1111	cn1186	pOP-EN00645_EST_C_1_pSK_SK	567
cl1034	ct1111	cn1186	pOP-EN00661_EST_C_1_pSK_SK	567
cl1035	ct1112	cn1187	pOP-EN00649_EST_C_1_pSK_SK	564
cl1035	ct1112	cn1187	pOP-EN00667_EST_C_1_pSK_SK	564
cl1036	ct1113	cn1188	pOP-EN00650_EST_C_1_pSK_SK	551
cl1036	ct1113	cn1188	pOP-EN00668_EST_C_1_pSK_SK	551
cl1037	ct1114	cn1189	pOP-EN00651_EST_C_1_pSK_SK	426
cl1037	ct1114	cn1189	pOP-EN00671_EST_C_1_pSK_SK	426
cl1038	ct1115	cn1190	pOP-CNHP00270_EST_C_1_pSK_SK	299
cl1038	ct1115	cn1190	pOP-EN00677_EST_C_1_pSK_SK	518
cl1039	ct1116	cn1191	pOP-CNH05046_EST_C_1_pSK_SK	756

cl1039	ct1116	cn1191	pOP-EN00683_EST_C_1_pSK_SK	508
cl1040	ct1117	cn1192	pOP-EN00681_EST_C_1_pSK_SK	508
cl1040	ct1117	cn1192	pOP-EN00693_EST_C_1_pSK_SK	529
cl1040	ct1117	cn1192	pOP-EN00704_EST_C_1_pSK_SK	458
cl1040	ct1117	cn1192	pOP-EN00708_EST_C_1_pSK_SK	416
cl1041	ct1118	cn1193	pOP-CNH01140_EST_C_1_pSK_SK	378
cl1041	ct1118	cn1193	pOP-EN00711_EST_C_1_pSK_SK	520
cl1042	ct1119	cn1194	pOP-EAP00902_EST_C_1_pBSK_SK	347
cl1042	ct1119	cn1194	pOP-EAP01504_EST_C_1_pBSK_SK	541
cl1042	ct1119	cn1194	pOP-EN00732_EST_C_1_pSK_SK	579
cl1043	ct1120	cn1195	pOP-EN00616_EST_C_1_pSK_SK	572
cl1043	ct1120	cn1195	pOP-EN00738_EST_C_1_pSK_SK	443
cl1044	ct1121	cn1196	pOP-EN00742_EST_C_1_pSK_SK	567
cl1044	ct1121	cn1196	pOP-EN00743_EST_C_1_pSK_SK	573
cl1044	ct1121	cn1196	pOP-EN00744_EST_C_1_pSK_SK	401
cl1045	ct1122	cn1197	pOP-EN00692_EST_C_1_pSK_SK	531
cl1045	ct1122	cn1197	pOP-EN00746_EST_C_1_pSK_SK	576
cl1046	ct1123	cn1198	pOP-EN00609_EST_C_1_pSK_SK	477
cl1046	ct1123	cn1198	pOP-EN00646_EST_C_1_pSK_SK	402
cl1046	ct1123	cn1198	pOP-EN00662_EST_C_1_pSK_SK	402
cl1046	ct1123	cn1198	pOP-EN00778_EST_C_1_pSK_SK	573
cl1047	ct1124	cn1199	pOP-EN00127_EST_C_1_pSK_SK	516
cl1047	ct1124	cn1199	pOP-EN00786_EST_C_1_pSK_SK	455
cl1047	ct1124	cn1199	pOP-EN00787_EST_C_1_pSK_SK	574
cl1047	ct1124	cn1199	pOP-EN00788_EST_C_1_pSK_SK	580
cl1048	ct1125	cn1200	pOP-EN00792_EST_C_1_pSK_SK	567
cl1048	ct1125	cn1200	pOP-EN00793_EST_C_1_pSK_SK	437
cl1049	ct1126	cn1201	pOP-EN00175_EST_C_1_pSK_SK	460
cl1049	ct1126	cn1201	pOP-EN00176_EST_C_1_pSK_SK	362
cl1049	ct1126	cn1201	pOP-EN00812_EST_C_1_pSK_SK	517
cl1050	ct1127	cn1202	pOP-EN00713_EST_C_1_pSK_SK	418
cl1050	ct1127	cn1202	pOP-EN00819_EST_C_1_pSK_SK	520
cl1051	ct1128	cn1203	pOP-EN00830_EST_C_1_pSK_SK	517
cl1051	ct1128	cn1203	pOP-EN00846_EST_C_1_pSK_SK	448
cl1052	ct1129	cn1204	pOP-EN00825_EST_C_1_pSK_SK	333
cl1052	ct1129	cn1204	pOP-EN00868_EST_C_1_pSK_SK	473
cl1053	ct1130	cn1205	pOP-EN00804_EST_C_1_pSK_SK	449
cl1053	ct1130	cn1205	pOP-EN00807_EST_C_1_pSK_SK	449
cl1053	ct1130	cn1205	pOP-EN00879_EST_C_1_pSK_SK	464
cl1054	ct1131	cn1206	pOP-CNH01220_EST_C_1_pSK_SK	453
cl1054	ct1131	cn1206	pOP-CNH04533	738
cl1054	ct1131	cn1206	pOP-EN00894_EST_C_1_pSK_SK	509
cl1055	ct1132	cn1207	pOP-EN00883_EST_C_1_pSK_SK	544
cl1055	ct1132	cn1207	pOP-EN00902_EST_C_1_pSK_SK	539
cl1056	ct1133	cn1208	pOP-EN00179_EST_C_1_pSK_SK	404
cl1056	ct1133	cn1208	pOP-EN00357_EST_C_1_pSK_SK	557
cl1056	ct1133	cn1208	pOP-EN00905_EST_C_1_pSK_SK	535
cl1057	ct1134	cn1209	pOP-EN00710_EST_C_1_pSK_SK	440
cl1057	ct1134	cn1209	pOP-EN00908_EST_C_1_pSK_SK	498
cl1058	ct1135	cn1210	pOP-CNI02213_EST_C_1_pSK_SK	652
cl1058	ct1135	cn1210	pOP-ENP00003_EST_C_1_pSK_SK	652
cl1059	ct1136	cn1211	pOP-CAP00025_EST_C_1_pBSK_SK	480
cl1059	ct1136	cn1211	pOP-EO05896_EST_C_1_pSK_SK	602
cl1059	ct1137	cn1212	pOP-EN00211_EST_C_1_pSK_SK	587
cl1059	ct1137	cn1212	pOP-EN00822_EST_C_1_pSK_SK	517
cl1059	ct1137	cn1212	pOP-EN00859_EST_C_1_pSK_SK	535

cl1060	ct1138	cn1213	pOP-CAP00056_EST_C_1_pBSK_SK	620
cl1060	ct1138	cn1213	pOP-CNH03805_EST_C_1_pSK_SK	580
cl1061	ct1139	cn1214	pOP-CAP00149_EST_C_1_pBSK_SK	476
cl1061	ct1139	cn1215	pOP-CNI01356_EST_C_1_pSK_SK	261
cl1061	ct1139	cn1215	pOP-CNI01645_EST_C_1_pSK_SK	355
cl1061	ct1139	cn1215	pOP-CNI01888_EST_C_1_pSK_SK	366
cl1062	ct1140	cn1216	pOP-CAP00156_EST_C_1_pBSK_SK	675
cl1062	ct1140	cn1216	pOP-CEO02170_EST_C_1_pSK_SK	123
cl1062	ct1140	cn1216	pOP-CNH00906_EST_C_1_pSK_SK	349
cl1062	ct1140	cn1216	pOP-CNH01271_EST_C_1_pSK_SK	690
cl1062	ct1140	cn1216	pOP-CNH02997_EST_C_1_pSK_SK	593
cl1062	ct1140	cn1216	pOP-CNH02999_EST_C_1_pSK_SK	506
cl1062	ct1140	cn1216	pOP-CNI02152_EST_C_1_pSK_SK	283
cl1062	ct1140	cn1216	pOP-CNIP00666_EST_C_1_pSK_SK	581
cl1063	ct1141	cn1217	pOP-CAP00216_EST_C_1_pBSK_SK	630
cl1063	ct1141	cn1217	pOP-CNIP00713_EST_C_1_pSK_SK	366
cl1064	ct1142	cn1218	pOP-CAP00250_EST_C_1_pBSK_SK	569
cl1064	ct1142	cn1218	pOP-CNHP00073_EST_C_1_pSK_SK	660
cl1065	ct1143	cn1219	pOP-CAP00263_EST_C_1_pBSK_SK	655
cl1065	ct1143	cn1219	pOP-EN00399_EST_C_1_pSK_SK	384
cl1066	ct1144	cn1220	pOP-CAP00291_EST_C_1_pBSK_SK	623
cl1066	ct1144	cn1220	pOP-EAP01966_EST_C_1_pBSK_SK	241
cl1067	ct1145	cn1221	pOP-CAP00317_EST_C_1_pBSK_SK	542
cl1067	ct1145	cn1221	pOP-CNH02531_EST_C_1_pSK_SK	487
cl1067	ct1145	cn1221	pOP-CNHP00421_EST_C_1_pSK_SK	725
cl1068	ct1146	cn1222	pOP-CAP00063_EST_C_1_pBSK_SK	409
cl1068	ct1146	cn1222	pOP-CAP00318_EST_C_1_pBSK_SK	600
cl1069	ct1147	cn1223	pOP-CAP00327_EST_C_1_pBSK_SK	610
cl1069	ct1147	cn1223	pOP-EO05707_EST_C_1_pSK_SK	471
cl1070	ct1148	cn1224	pOP-CNH03072_EST_C_1_pSK_SK	657
cl1070	ct1149	cn1225	pOP-CAP00336_EST_C_1_pBSK_SK	420
cl1070	ct1149	cn1225	pOP-EAP01705_EST_C_1_pBSK_SK	435
cl1070	ct1150	cn1226	pOP-CNH01687_EST_C_1_pSK_SK	708
cl1070	ct1150	cn1226	pOP-CNH02405_EST_C_1_pSK_SK	522
cl1070	ct1150	cn1226	pOP-CNHP00455_EST_C_1_pSK_SK	654
cl1070	ct1150	cn1226	pOP-EO07256_EST_C_1_pSK_SK	799
cl1071	ct1151	cn1227	pOP-CAP00050_EST_C_1_pBSK_SK	356
cl1071	ct1151	cn1227	pOP-CAP00340_EST_C_1_pBSK_SK	248
cl1071	ct1151	cn1227	pOP-CAP00356_EST_C_1_pBSK_SK	510
cl1071	ct1151	cn1227	pOP-CBP00055_EST_C_1_pBSK_SK	456
cl1071	ct1151	cn1227	pOP-CBP00108_EST_C_1_pBSK_SK	554
cl1071	ct1151	cn1227	pOP-CBP00116_EST_C_1_pBSK_SK	653
cl1071	ct1151	cn1227	pOP-CBP00186_EST_C_1_pBSK_SK	625
cl1071	ct1151	cn1227	pOP-CBP00268_EST_C_1_pBSK_SK	555
cl1071	ct1151	cn1227	pOP-CNIP00197_EST_C_1_pSK_SK	324
cl1071	ct1151	cn1227	pOP-EAP02860_EST_C_1_pBSK_SK	188
cl1071	ct1151	cn1228	pOP-CAP00052_EST_C_1_pBSK_SK	573
cl1072	ct1152	cn1229	pOP-CAP00097_EST_C_1_pBSK_SK	661
cl1072	ct1152	cn1229	pOP-CAP00363_EST_C_1_pBSK_SK	576
cl1073	ct1153	cn1230	pOP-CAP00313_EST_C_1_pBSK_SK	480
cl1073	ct1153	cn1230	pOP-CAP00409_EST_C_1_pBSK_SK	610
cl1074	ct1154	cn1231	pOP-CAP00288_EST_C_1_pBSK_SK	263
cl1074	ct1154	cn1231	pOP-CAP00417_EST_C_1_pBSK_SK	271
cl1075	ct1155	cn1232	pOP-CAP05003_EST_C_1_pBSK_SK	520
cl1075	ct1155	cn1232	pOP-CEO01229_EST_C_1_pSK_SK	666
cl1075	ct1155	cn1232	pOP-CNH00580_EST_C_1_pSK_SK	523

cl1076	ct1156	cn1233	pOP-CAP05008_EST_C_1_pBSK_SK	603
cl1076	ct1156	cn1233	pOP-CNH04541	320
cl1077	ct1157	cn1234	pOP-CBP00006_EST_C_1_pBSK_SK	524
cl1077	ct1157	cn1234	pOP-CNH00913_EST_C_1_pSK_SK	545
cl1078	ct1158	cn1235	pOP-CBP00047_EST_C_1_pBSK_SK	420
cl1078	ct1158	cn1235	pOP-EAP00403_EST_C_1_pBSK_SK	179
cl1079	ct1159	cn1236	pOP-CBP00058_EST_C_1_pBSK_SK	295
cl1079	ct1159	cn1236	pOP-CNI01308_EST_C_1_pSK_SK	459
cl1079	ct1159	cn1236	pOP-CNIP00397_EST_C_1_pSK_SK	351
cl1080	ct1160	cn1237	pOP-CBP00001_EST_C_1_pBSK_SK	259
cl1080	ct1160	cn1237	pOP-CBP00086_EST_C_1_pBSK_SK	518
cl1081	ct1161	cn1238	pOP-CBP00118_EST_C_1_pBSK_SK	312
cl1081	ct1161	cn1238	pOP-EAP03260_EST_C_1_pBSK_SK	256
cl1081	ct1161	cn1238	pOP-EAP03570_EST_C_1_pBSK_SK	208
cl1081	ct1161	cn1238	pOP-EO03144_EST_C_1_pSK_SK	473
cl1081	ct1161	cn1238	pOP-EO08175_EST_C_1_pSK_SK	572
cl1081	ct1161	cn1238	pOP-EO08390_EST_C_1_pSK_SK	340
cl1082	ct1162	cn1239	pOP-CBP00119_EST_C_1_pBSK_SK	641
cl1082	ct1162	cn1239	pOP-EAP00591_EST_C_1_pBSK_SK	503
cl1082	ct1162	cn1239	pOP-EAP01502_EST_C_1_pBSK_SK	373
cl1082	ct1162	cn1239	pOP-EAP02289_EST_C_1_pBSK_SK	575
cl1082	ct1162	cn1239	pOP-EN00210_EST_C_1_pSK_SK	432
cl1083	ct1163	cn1240	pOP-CBP00122_EST_C_1_pBSK_SK	549
cl1083	ct1163	cn1240	pOP-EAP01865_EST_C_1_pBSK_SK	574
cl1084	ct1164	cn1241	pOP-CBP00124_EST_C_1_pBSK_SK	422
cl1084	ct1164	cn1241	pOP-CEO01305	184
cl1084	ct1164	cn1241	pOP-CNIP00308_EST_C_1_pSK_SK	410
cl1084	ct1164	cn1241	pOP-CNIP00799_EST_C_1_pSK_SK	321
cl1085	ct1165	cn1242	pOP-CBP00128_EST_C_1_pBSK_SK	472
cl1085	ct1165	cn1242	pOP-EO02055_EST_C_1_pSK_SK	529
cl1085	ct1165	cn1242	pOP-EO06295_EST_C_1_pSK_SK	592
cl1086	ct1166	cn1243	pOP-CBP00129_EST_C_1_pBSK_SK	194
cl1086	ct1166	cn1243	pOP-CNHP00533_EST_C_1_pSK_SK	548
cl1086	ct1166	cn1243	pOP-EAP00531_EST_C_1_pBSK_SK	392
cl1086	ct1166	cn1243	pOP-EAP01076_EST_C_1_pBSK_SK	259
cl1086	ct1166	cn1243	pOP-EAP01650_EST_C_1_pBSK_SK	513
cl1086	ct1166	cn1243	pOP-EAP02892_EST_C_1_pBSK_SK	216
cl1086	ct1166	cn1243	pOP-EO06064_EST_C_1_pSK_SK	591
cl1086	ct1166	cn1244	pOP-CEO02509_EST_C_1_pSK_SK	584
cl1087	ct1167	cn1245	pOP-CAP00296_EST_C_1_pBSK_SK	611
cl1087	ct1167	cn1245	pOP-CBP00093_EST_C_1_pBSK_SK	452
cl1087	ct1167	cn1245	pOP-CBP00130_EST_C_1_pBSK_SK	639
cl1087	ct1167	cn1245	pOP-CNH04724_EST_C_1_pSK_SK	556
cl1088	ct1168	cn1246	pOP-CBP00145_EST_C_1_pBSK_SK	636
cl1088	ct1168	cn1246	pOP-CNHP00183_EST_C_1_pSK_SK	582
cl1088	ct1168	cn1246	pOP-CNI01860_EST_C_1_pSK_SK	530
cl1088	ct1168	cn1246	pOP-CNIP00027_EST_C_1_pSK_SK	626
cl1089	ct1169	cn1247	pOP-CBP00163_EST_C_1_pBSK_SK	306
cl1089	ct1169	cn1247	pOP-CBP00164_EST_C_1_pBSK_SK	220
cl1089	ct1169	cn1247	pOP-CNIP00162_EST_C_1_pSK_SK	504
cl1089	ct1169	cn1247	pOP-EO06023_EST_C_1_pSK_SK	456
cl1090	ct1170	cn1248	pOP-CEO03342_EST_C_1_pSK_SK	519
cl1090	ct1171	cn1249	pOP-CBP00200_EST_C_1_pBSK_SK	489
cl1090	ct1171	cn1249	pOP-CEM00172_EST_C_1_pSK_SK	330
cl1090	ct1171	cn1249	pOP-EAP03824_EST_C_1_pBSK_SK	518
cl1090	ct1172	cn1250	pOP-CAP00038_EST_C_1_pBSK_SK	397

cl1090	ct1172	cn1250	pOP-CNH00734_EST_C_1_pSK_SK	535
cl1090	ct1172	cn1250	pOP-CNI01907_EST_C_1_pSK_SK	207
cl1090	ct1172	cn1250	pOP-EO06177_EST_C_1_pSK_SK	289
cl1090	ct1173	cn1251	pOP-CNH02669_EST_C_1_pSK_SK	594
cl1090	ct1173	cn1251	pOP-CNH04196	459
cl1090	ct1173	cn1251	pOP-CNIP00707_EST_C_1_pSK_SK	412
cl1090	ct1173	cn1251	pOP-EO06707_EST_C_1_pSK_SK	752
cl1090	ct1173	cn1251	pOP-EO06716_EST_C_1_pSK_SK	853
cl1090	ct1173	cn1251	pOP-EO08098_EST_C_1_pSK_SK	568
cl1091	ct1174	cn1252	pOP-CBP00234_EST_C_1_pBSK_SK	469
cl1091	ct1174	cn1252	pOP-CNI02067_EST_C_1_pSK_SK	489
cl1092	ct1175	cn1253	pOP-CBP00235_EST_C_1_pBSK_SK	680
cl1092	ct1175	cn1253	pOP-EO03427_EST_C_1_pSK_SK	372
cl1093	ct1176	cn1254	pOP-CBP00249_EST_C_1_pBSK_SK	519
cl1093	ct1176	cn1254	pOP-CNH03162_EST_C_1_pSK_SK	320
cl1093	ct1176	cn1254	pOP-CNI01732_EST_C_1_pSK_SK	476
cl1093	ct1176	cn1254	pOP-CNI02171_EST_C_1_pSK_SK	456
cl1094	ct1177	cn1255	pOP-CAP00304_EST_C_1_pBSK_SK	431
cl1094	ct1177	cn1255	pOP-CBP00261_EST_C_1_pBSK_SK	600
cl1094	ct1177	cn1255	pOP-CNI01613_EST_C_1_pSK_SK	295
cl1095	ct1178	cn1256	pOP-CCP00002_EST_C_1_pBSK_SK	120
cl1095	ct1178	cn1256	pOP-CCP00008_EST_C_1_pBSK_SK	331
cl1096	ct1179	cn1257	pOP-CCP00016_EST_C_1_pBSK_SK	134
cl1096	ct1179	cn1257	pOP-EO03436_EST_C_1_pSK_SK	492
cl1096	ct1179	cn1257	pOP-EO08490_EST_C_1_pSK_SK	124
cl1097	ct1180	cn1258	pOP-CCP00017_EST_C_1_pBSK_SK	208
cl1097	ct1180	cn1258	pOP-EAP02282_EST_C_1_pBSK_SK	249
cl1098	ct1181	cn1259	pOP-CEM00037_EST_C_1_pSK_SK	188
cl1098	ct1181	cn1259	pOP-CNIP00187_EST_C_1_pSK_SK	410
cl1098	ct1182	cn1260	pOP-CNIP01036_EST_C_1_pSK_SK	386
cl1098	ct1182	cn1260	pOP-CNIP01066_EST_C_1_pSK_SK	386
cl1099	ct1183	cn1261	pOP-CEM00038_EST_C_1_pSK_SK	293
cl1099	ct1183	cn1261	pOP-EAP03166_EST_C_1_pBSK_SK	464
cl1100	ct1184	cn1262	pOP-CEM00051_EST_C_1_pSK_SK	330
cl1100	ct1184	cn1262	pOP-CNIP00046_EST_C_1_pSK_SK	219
cl1101	ct1185	cn1263	pOP-CEM00055_EST_C_1_pSK_SK	365
cl1101	ct1185	cn1263	pOP-CNI01815_EST_C_1_pSK_SK	567
cl1101	ct1185	cn1263	pOP-CNI01850_EST_C_1_pSK_SK	557
cl1101	ct1185	cn1263	pOP-EAP00594_EST_C_1_pBSK_SK	527
cl1102	ct1186	cn1264	pOP-CEM00059_EST_C_1_pSK_SK	687
cl1102	ct1186	cn1264	pOP-CEO03497_EST_C_1_pSK_SK	481
cl1103	ct1187	cn1265	pOP-CEM00087_EST_C_1_pSK_SK	226
cl1103	ct1187	cn1265	pOP-CEO02627_EST_C_1_pSK_SK	521
cl1103	ct1188	cn1266	pOP-CEO01014_EST_C_1_pSK_SK	358
cl1103	ct1188	cn1266	pOP-CNI01143_EST_C_1_pSK_SK	675
cl1103	ct1188	cn1266	pOP-CNIP00829_EST_C_1_pSK_SK	217
cl1103	ct1188	cn1266	pOP-EAP01060_EST_C_1_pBSK_SK	338
cl1103	ct1188	cn1266	pOP-EAP02883_EST_C_1_pBSK_SK	319
cl1103	ct1188	cn1266	pOP-EAP03719_EST_C_1_pBSK_SK	455
cl1104	ct1189	cn1267	pOP-CEM00088_EST_C_1_pSK_SK	651
cl1104	ct1189	cn1267	pOP-CNH02381_EST_C_1_pSK_SK	506
cl1104	ct1189	cn1267	pOP-CNI01565_EST_C_1_pSK_SK	398
cl1104	ct1189	cn1267	pOP-EAP03733_EST_C_1_pBSK_SK	454
cl1104	ct1189	cn1267	pOP-EAP03734_EST_C_1_pBSK_SK	484
cl1105	ct1190	cn1268	pOP-CEO00690_EST_C_1_pSK_SK	474
cl1105	ct1190	cn1269	pOP-CEM00096_EST_C_1_pSK_SK	359

cl1106	ct1191	cn1270	pOP-CEM00093_EST_C_1_pSK_SK	699
cl1106	ct1191	cn1270	pOP-CEM00097_EST_C_1_pSK_SK	721
cl1107	ct1192	cn1271	pOP-CEM00101_EST_C_1_pSK_SK	352
cl1107	ct1192	cn1271	pOP-CNIP00986_EST_C_1_pSK_SK	113
cl1108	ct1193	cn1272	pOP-CEM00123_EST_C_1_pSK_SK	468
cl1108	ct1193	cn1272	pOP-CNIP00616_EST_C_1_pSK_SK	314
cl1108	ct1193	cn1272	pOP-EAP01402_EST_C_1_pBSK_SK	229
cl1109	ct1194	cn1273	pOP-CEM00134_EST_C_1_pSK_SK	217
cl1109	ct1194	cn1273	pOP-EAP01899_EST_C_1_pBSK_SK	532
cl1110	ct1195	cn1274	pOP-CEM00135_EST_C_1_pSK_SK	317
cl1110	ct1195	cn1274	pOP-CEM00136_EST_C_1_pSK_SK	317
cl1111	ct1196	cn1275	pOP-CEM00143_EST_C_1_pSK_SK	163
cl1111	ct1196	cn1275	pOP-EAP01015_EST_C_1_pBSK_SK	161
cl1112	ct1197	cn1276	pOP-CNIP00703_EST_C_1_pSK_SK	488
cl1112	ct1197	cn1277	pOP-CEM00167_EST_C_1_pSK_SK	148
cl1112	ct1197	cn1277	pOP-EAP02068_EST_C_1_pBSK_SK	443
cl1113	ct1198	cn1278	pOP-CEM00170_EST_C_1_pSK_SK	104
cl1113	ct1198	cn1278	pOP-CEM00186_EST_C_1_pSK_SK	104
cl1114	ct1199	cn1279	pOP-CNH01617_EST_C_1_pSK_SK	586
cl1114	ct1200	cn1280	pOP-CEM00188_EST_C_1_pSK_SK	368
cl1114	ct1200	cn1280	pOP-CNH03495_EST_C_1_pSK_SK	568
cl1114	ct1200	cn1280	pOP-CNHP00258_EST_C_1_pSK_SK	559
cl1115	ct1201	cn1281	pOP-CEM00191_EST_C_1_pSK_SK	318
cl1115	ct1201	cn1281	pOP-CNH04241	642
cl1116	ct1202	cn1282	pOP-CEM00201_EST_C_1_pSK_SK	338
cl1116	ct1202	cn1282	pOP-CEM00202_EST_C_1_pSK_SK	338
cl1117	ct1203	cn1283	pOP-CEM00122_EST_C_1_pSK_SK	495
cl1117	ct1203	cn1283	pOP-CEM00209_EST_C_1_pSK_SK	580
cl1118	ct1204	cn1284	pOP-CEM00215_EST_C_1_pSK_SK	495
cl1118	ct1204	cn1284	pOP-EAP01053_EST_C_1_pBSK_SK	434
cl1119	ct1205	cn1285	pOP-CEM00151_EST_C_1_pSK_SK	241
cl1119	ct1205	cn1285	pOP-CEM00220_EST_C_1_pSK_SK	241
cl1120	ct1206	cn1286	pOP-CEM00222_EST_C_1_pSK_SK	423
cl1120	ct1206	cn1286	pOP-CNH04481	753
cl1121	ct1207	cn1287	pOP-CEM00239_EST_C_1_pSK_SK	210
cl1121	ct1207	cn1287	pOP-CNH01304_EST_C_1_pSK_SK	548
cl1121	ct1207	cn1287	pOP-CNH04897_EST_C_1_pSK_SK	690
cl1121	ct1207	cn1287	pOP-EAP02991_EST_C_1_pBSK_SK	469
cl1122	ct1208	cn1288	pOP-CEM00248_EST_C_1_pSK_SK	197
cl1122	ct1208	cn1288	pOP-EAP02710_EST_C_1_pBSK_SK	708
cl1123	ct1209	cn1289	pOP-CEM00107_EST_C_1_pSK_SK	314
cl1123	ct1209	cn1289	pOP-CEM00233_EST_C_1_pSK_SK	314
cl1123	ct1209	cn1289	pOP-CEM00249_EST_C_1_pSK_SK	314
cl1124	ct1210	cn1290	pOP-EO05619_EST_C_1_pSK_SK	410
cl1124	ct1211	cn1291	pOP-EO04460_EST_C_1_pSK_SK	506
cl1124	ct1211	cn1291	pOP-EO04461_EST_C_1_pSK_SK	455
cl1124	ct1212	cn1292	pOP-CAP00282_EST_C_1_pBSK_SK	419
cl1124	ct1212	cn1292	pOP-CBP00142_EST_C_1_pBSK_SK	580
cl1124	ct1212	cn1292	pOP-CEMP00007_EST_C_1_pSK_SK	327
cl1124	ct1212	cn1292	pOP-CNIP00865_EST_C_1_pSK_SK	239
cl1124	ct1212	cn1292	pOP-EO05163_EST_C_1_pSK_SK	534
cl1125	ct1213	cn1293	pOP-CAP00033_EST_C_1_pBSK_SK	387
cl1125	ct1213	cn1293	pOP-CEMP00008_EST_C_1_pSK_SK	301
cl1125	ct1213	cn1293	pOP-EO06834_EST_C_1_pSK_SK	605
cl1126	ct1214	cn1294	pOP-CEMP00027_EST_C_1_pSK_SK	452
cl1126	ct1214	cn1294	pOP-EAP02326_EST_C_1_pBSK_SK	255

cl1127	ct1215	cn1295	pOP-CEMP00034_EST_C_1_pSK_SK	338
cl1127	ct1215	cn1295	pOP-CEO02711_EST_C_1_pSK_SK	385
cl1127	ct1215	cn1296	pOP-CEO02986_EST_C_1_pSK_SK	263
cl1127	ct1215	cn1296	pOP-CEO03629_EST_C_1_pSK_SK	497
cl1127	ct1215	cn1296	pOP-CNIP00854_EST_C_1_pSK_SK	292
cl1128	ct1216	cn1297	pOP-CEO00509_EST_C_1_pSK_SK	163
cl1128	ct1216	cn1297	pOP-CNIP00693_EST_C_1_pSK_SK	451
cl1129	ct1217	cn1298	pOP-CEO00510_EST_C_1_pSK_SK	232
cl1129	ct1217	cn1298	pOP-CEO01325	242
cl1130	ct1218	cn1299	pOP-CEO00522_EST_C_1_pSK_SK	240
cl1130	ct1218	cn1299	pOP-CNIP00012_EST_C_1_pSK_SK	160
cl1131	ct1219	cn1300	pOP-CEO00524_EST_C_1_pSK_SK	336
cl1131	ct1219	cn1300	pOP-CNI01689_EST_C_1_pSK_SK	280
cl1132	ct1220	cn1301	pOP-CEO00526_EST_C_1_pSK_SK	672
cl1132	ct1220	cn1301	pOP-CNH01594_EST_C_1_pSK_SK	557
cl1133	ct1221	cn1302	pOP-CEO00531_EST_C_1_pSK_SK	206
cl1133	ct1221	cn1302	pOP-EO05529_EST_C_1_pSK_SK	206
cl1134	ct1222	cn1303	pOP-CEO00536_EST_C_1_pSK_SK	640
cl1134	ct1222	cn1303	pOP-CNH02112_EST_C_1_pSK_SK	609
cl1134	ct1222	cn1303	pOP-CNH04992_EST_C_1_pSK_SK	427
cl1135	ct1223	cn1304	pOP-CNH02268_EST_C_1_pSK_SK	686
cl1135	ct1223	cn1305	pOP-CEO00543_EST_C_1_pSK_SK	348
cl1136	ct1224	cn1306	pOP-CEO00544_EST_C_1_pSK_SK	343
cl1136	ct1224	cn1306	pOP-CEO00546_EST_C_1_pSK_SK	322
cl1136	ct1224	cn1306	pOP-EO04479_EST_C_1_pSK_SK	486
cl1137	ct1225	cn1307	pOP-CEO00540_EST_C_1_pSK_SK	127
cl1137	ct1225	cn1307	pOP-CEO00548_EST_C_1_pSK_SK	126
cl1138	ct1226	cn1308	pOP-CEO00553_EST_C_1_pSK_SK	694
cl1138	ct1226	cn1308	pOP-CNH00966_EST_C_1_pSK_SK	431
cl1138	ct1226	cn1308	pOP-CNH01150_EST_C_1_pSK_SK	305
cl1138	ct1226	cn1308	pOP-CNH04629	766
cl1138	ct1226	cn1308	pOP-CNI01324_EST_C_1_pSK_SK	339
cl1138	ct1226	cn1308	pOP-CNNP00007_EST_C_1_pBSK_SK	632
cl1138	ct1226	cn1308	pOP-EO06414_EST_C_1_pSK_SK	188
cl1139	ct1227	cn1309	pOP-CEO00568_EST_C_1_pSK_SK	390
cl1139	ct1227	cn1309	pOP-CEO00569_EST_C_1_pSK_SK	390
cl1140	ct1228	cn1310	pOP-CEO00570_EST_C_1_pSK_SK	180
cl1140	ct1228	cn1310	pOP-CEO00571_EST_C_1_pSK_SK	180
cl1141	ct1229	cn1311	pOP-CEMP00016_EST_C_1_pSK_SK	120
cl1141	ct1229	cn1311	pOP-CEO00577_EST_C_1_pSK_SK	208
cl1141	ct1229	cn1311	pOP-CNIP00844_EST_C_1_pSK_SK	203
cl1142	ct1230	cn1312	pOP-CEO00582_EST_C_1_pSK_SK	255
cl1142	ct1230	cn1313	pOP-CEO00698_EST_C_1_pSK_SK	223
cl1143	ct1231	cn1314	pOP-CEO00583_EST_C_1_pSK_SK	512
cl1143	ct1231	cn1314	pOP-CNH03324_EST_C_1_pSK_SK	682
cl1144	ct1232	cn1315	pOP-CEO00601_EST_C_1_pSK_SK	112
cl1144	ct1232	cn1315	pOP-EAP02908_EST_C_1_pBSK_SK	475
cl1145	ct1233	cn1316	pOP-CEO00606_EST_C_1_pSK_SK	129
cl1145	ct1233	cn1316	pOP-EAP05048_EST_C_1_pBSK_SK	495
cl1146	ct1234	cn1317	pOP-CEO00607_EST_C_1_pSK_SK	517
cl1146	ct1234	cn1317	pOP-CNH00653_EST_C_1_pSK_SK	558
cl1147	ct1235	cn1318	pOP-CEO00610_EST_C_1_pSK_SK	254
cl1147	ct1235	cn1318	pOP-CEO00611_EST_C_1_pSK_SK	254
cl1148	ct1236	cn1319	pOP-CEO00615_EST_C_1_pSK_SK	329
cl1148	ct1236	cn1319	pOP-CEO00616_EST_C_1_pSK_SK	329
cl1149	ct1237	cn1320	pOP-CEO00633_EST_C_1_pSK_SK	264

cl1149	ct1237	cn1320	pOP-CNIP01038_EST_C_1_pSK_SK	376
cl1150	ct1238	cn1321	pOP-CEO00643_EST_C_1_pSK_SK	199
cl1150	ct1238	cn1321	pOP-CNH04903_EST_C_1_pSK_SK	733
cl1151	ct1239	cn1322	pOP-EAP03405_EST_C_1_pBSK_SK	345
cl1151	ct1239	cn1322	pOP-EO05661_EST_C_1_pSK_SK	454
cl1151	ct1239	cn1322	pOP-EO07193_EST_C_1_pSK_SK	703
cl1151	ct1239	cn1323	pOP-CEO00648_EST_C_1_pSK_SK	403
cl1152	ct1240	cn1324	pOP-CEO00651_EST_C_1_pSK_SK	178
cl1152	ct1240	cn1324	pOP-CEO01333	248
cl1153	ct1241	cn1325	pOP-CEO00653_EST_C_1_pSK_SK	394
cl1153	ct1241	cn1325	pOP-CNI01651_EST_C_1_pSK_SK	671
cl1153	ct1241	cn1326	pOP-EO03609_EST_C_1_pSK_SK	434
cl1154	ct1242	cn1327	pOP-CEO00672_EST_C_1_pSK_SK	772
cl1154	ct1242	cn1327	pOP-CNI01491_EST_C_1_pSK_SK	453
cl1154	ct1242	cn1327	pOP-EAP01792_EST_C_1_pBSK_SK	363
cl1154	ct1242	cn1328	pOP-EAP00738_EST_C_1_pBSK_SK	489
cl1155	ct1243	cn1329	pOP-CEO00674_EST_C_1_pSK_SK	376
cl1155	ct1243	cn1329	pOP-CEO01298	380
cl1155	ct1243	cn1329	pOP-EO06144_EST_C_1_pSK_SK	294
cl1156	ct1244	cn1330	pOP-CEO00683_EST_C_1_pSK_SK	251
cl1156	ct1244	cn1330	pOP-EAP00633_EST_C_1_pBSK_SK	277
cl1156	ct1244	cn1330	pOP-EO07029_EST_C_1_pSK_SK	594
cl1157	ct1245	cn1331	pOP-CEO00692_EST_C_1_pSK_SK	333
cl1157	ct1245	cn1331	pOP-CNH01571_EST_C_1_pSK_SK	656
cl1158	ct1246	cn1332	pOP-CEO00713_EST_C_1_pSK_SK	444
cl1158	ct1246	cn1332	pOP-CNH03474_EST_C_1_pSK_SK	503
cl1158	ct1246	cn1332	pOP-EAP01277_EST_C_1_pBSK_SK	269
cl1159	ct1247	cn1333	pOP-CEO00723_EST_C_1_pSK_SK	288
cl1159	ct1247	cn1333	pOP-CNI01197_EST_C_1_pSK_SK	230
cl1160	ct1248	cn1334	pOP-CEO00759_EST_C_1_pSK_SK	336
cl1160	ct1248	cn1334	pOP-EAP00945_EST_C_1_pBSK_SK	380
cl1161	ct1249	cn1335	pOP-CEO00769_EST_C_1_pSK_SK	334
cl1161	ct1249	cn1335	pOP-CEO00770_EST_C_1_pSK_SK	332
cl1162	ct1250	cn1336	pOP-CEO00792_EST_C_1_pSK_SK	358
cl1162	ct1250	cn1336	pOP-EO04616_EST_C_1_pSK_SK	551
cl1162	ct1250	cn1336	pOP-EO07210_EST_C_1_pSK_SK	631
cl1163	ct1251	cn1337	pOP-CBP00011_EST_C_1_pBSK_SK	215
cl1163	ct1251	cn1337	pOP-CEO00798_EST_C_1_pSK_SK	277
cl1164	ct1252	cn1338	pOP-CEO00838_EST_C_1_pSK_SK	410
cl1164	ct1252	cn1338	pOP-EAP03139_EST_C_1_pBSK_SK	651
cl1165	ct1253	cn1339	pOP-CEO00840_EST_C_1_pSK_SK	211
cl1165	ct1253	cn1339	pOP-CNIP00091_EST_C_1_pSK_SK	160
cl1166	ct1254	cn1340	pOP-CEM00234_EST_C_1_pSK_SK	593
cl1166	ct1254	cn1340	pOP-CEO00857_EST_C_1_pSK_SK	466
cl1166	ct1254	cn1340	pOP-CNHP00210_EST_C_1_pSK_SK	453
cl1166	ct1254	cn1340	pOP-EO05592_EST_C_1_pSK_SK	502
cl1167	ct1255	cn1341	pOP-CEO00869_EST_C_1_pSK_SK	723
cl1167	ct1255	cn1341	pOP-CNI01922_EST_C_1_pSK_SK	177
cl1168	ct1256	cn1342	pOP-CEO00887_EST_C_1_pSK_SK	490
cl1168	ct1256	cn1342	pOP-EAP03834_EST_C_1_pBSK_SK	645
cl1169	ct1257	cn1343	pOP-CEMP00030_EST_C_1_pSK_SK	332
cl1169	ct1257	cn1343	pOP-CEO00891_EST_C_1_pSK_SK	125
cl1170	ct1258	cn1344	pOP-CEO00905_EST_C_1_pSK_SK	135
cl1170	ct1258	cn1344	pOP-CNH04771_EST_C_1_pSK_SK	525
cl1170	ct1258	cn1344	pOP-CNIP00577_EST_C_1_pSK_SK	456
cl1171	ct1259	cn1345	pOP-CEO00920_EST_C_1_pSK_SK	715

cl1171	ct1259	cn1345	pOP-CNIP00236_EST_C_1_pSK_SK	289
cl1171	ct1259	cn1345	pOP-CNIP00409_EST_C_1_pSK_SK	289
cl1172	ct1260	cn1346	pOP-CEO00934_EST_C_1_pSK_SK	297
cl1172	ct1260	cn1346	pOP-CNI01408_EST_C_1_pSK_SK	379
cl1173	ct1261	cn1347	pOP-CEO00941_EST_C_1_pSK_SK	239
cl1173	ct1261	cn1347	pOP-CEO00943_EST_C_1_pSK_SK	239
cl1174	ct1262	cn1348	pOP-CEO00945_EST_C_1_pSK_SK	340
cl1174	ct1262	cn1348	pOP-CEO00949_EST_C_1_pSK_SK	334
cl1175	ct1263	cn1349	pOP-EAP01264_EST_C_1_pBSK_SK	412
cl1175	ct1263	cn1349	pOP-EAP01946_EST_C_1_pBSK_SK	490
cl1175	ct1263	cn1350	pOP-CEO00973_EST_C_1_pSK_SK	489
cl1176	ct1264	cn1351	pOP-CEO01003_EST_C_1_pSK_SK	353
cl1176	ct1264	cn1351	pOP-EAP00804_EST_C_1_pBSK_SK	438
cl1177	ct1265	cn1352	pOP-CEO01017_EST_C_1_pSK_SK	443
cl1177	ct1265	cn1352	pOP-EO03259_EST_C_1_pSK_SK	472
cl1178	ct1266	cn1353	pOP-CEO01027_EST_C_1_pSK_SK	338
cl1178	ct1266	cn1353	pOP-EAP02946_EST_C_1_pBSK_SK	267
cl1179	ct1267	cn1354	pOP-CEO01033_EST_C_1_pSK_SK	533
cl1179	ct1267	cn1354	pOP-CNIP01044_EST_C_1_pSK_SK	464
cl1180	ct1268	cn1355	pOP-CEO01053_EST_C_1_pSK_SK	406
cl1180	ct1268	cn1355	pOP-CNI01830_EST_C_1_pSK_SK	265
cl1180	ct1268	cn1355	pOP-EAP02190_EST_C_1_pBSK_SK	336
cl1181	ct1269	cn1356	pOP-CEO01062_EST_C_1_pSK_SK	667
cl1181	ct1269	cn1356	pOP-CNH00665_EST_C_1_pSK_SK	421
cl1181	ct1269	cn1356	pOP-CNH04991_EST_C_1_pSK_SK	634
cl1182	ct1270	cn1357	pOP-CEO01066_EST_C_1_pSK_SK	534
cl1182	ct1270	cn1357	pOP-CNH02438_EST_C_1_pSK_SK	541
cl1182	ct1270	cn1357	pOP-EAP05039_EST_C_1_pBSK_SK	623
cl1183	ct1271	cn1358	pOP-CEO01069_EST_C_1_pSK_SK	312
cl1183	ct1271	cn1358	pOP-CNH04451	800
cl1184	ct1272	cn1359	pOP-CEO01070_EST_C_1_pSK_SK	448
cl1184	ct1272	cn1359	pOP-CEO01073_EST_C_1_pSK_SK	448
cl1185	ct1273	cn1360	pOP-CEO01108_EST_C_1_pSK_SK	392
cl1185	ct1273	cn1360	pOP-EAP01035_EST_C_1_pBSK_SK	218
cl1185	ct1273	cn1360	pOP-EAP01036_EST_C_1_pBSK_SK	266
cl1185	ct1273	cn1360	pOP-EAP01450_EST_C_1_pBSK_SK	268
cl1186	ct1274	cn1361	pOP-CEO01114_EST_C_1_pSK_SK	452
cl1186	ct1274	cn1361	pOP-CNH01561_EST_C_1_pSK_SK	626
cl1187	ct1275	cn1362	pOP-CEO01117_EST_C_1_pSK_SK	185
cl1187	ct1275	cn1362	pOP-EAP02335_EST_C_1_pBSK_SK	546
cl1188	ct1276	cn1363	pOP-CEMP00009_EST_C_1_pSK_SK	320
cl1188	ct1276	cn1363	pOP-CEO01122_EST_C_1_pSK_SK	384
cl1188	ct1276	cn1363	pOP-CNH02480_EST_C_1_pSK_SK	364
cl1189	ct1277	cn1364	pOP-CEO01130_EST_C_1_pSK_SK	332
cl1189	ct1277	cn1364	pOP-CNI01459_EST_C_1_pSK_SK	189
cl1189	ct1277	cn1364	pOP-EAP03869_EST_C_1_pBSK_SK	561
cl1190	ct1278	cn1365	pOP-CNHP00386_EST_C_1_pSK_SK	633
cl1190	ct1278	cn1365	pOP-CNI01735_EST_C_1_pSK_SK	533
cl1190	ct1278	cn1365	pOP-CNI01774_EST_C_1_pSK_SK	565
cl1190	ct1278	cn1365	pOP-EAP03214_EST_C_1_pBSK_SK	479
cl1190	ct1278	cn1365	pOP-EO02421_EST_C_1_pSK_SK	347
cl1190	ct1279	cn1366	pOP-CEO00501_EST_C_1_pSK_SK	308
cl1190	ct1279	cn1366	pOP-CEO01139_EST_C_1_pSK_SK	201
cl1190	ct1279	cn1366	pOP-CEO02445_EST_C_1_pSK_SK	355
cl1190	ct1279	cn1366	pOP-CNHP00034_EST_C_1_pSK_SK	543
cl1190	ct1279	cn1366	pOP-CNI01549_EST_C_1_pSK_SK	339

cl1190	ct1279	cn1366	pOP-CNIP00911_EST_C_1_pSK_SK	538
cl1190	ct1279	cn1366	pOP-EAP00070_EST_C_1_pBSK_SK	590
cl1190	ct1279	cn1366	pOP-EAP02770_EST_C_1_pBSK_SK	648
cl1190	ct1279	cn1366	pOP-EO02985_EST_C_1_pSK_SK	306
cl1190	ct1279	cn1366	pOP-EO04239_EST_C_1_pSK_SK	385
cl1190	ct1279	cn1367	pOP-CEO01420_EST_C_1_pSK_SK	566
cl1191	ct1280	cn1368	pOP-CEO01143_EST_C_1_pSK_SK	502
cl1191	ct1281	cn1369	pOP-CNH02577_EST_C_1_pSK_SK	521
cl1192	ct1282	cn1370	pOP-CEO01144_EST_C_1_pSK_SK	472
cl1192	ct1282	cn1370	pOP-CNI01470_EST_C_1_pSK_SK	314
cl1193	ct1283	cn1371	pOP-CEM00142_EST_C_1_pSK_SK	366
cl1193	ct1283	cn1371	pOP-CEO01146_EST_C_1_pSK_SK	286
cl1193	ct1283	cn1371	pOP-CEO01767_EST_C_1_pSK_SK	362
cl1193	ct1283	cn1371	pOP-CEO01777_EST_C_1_pSK_SK	362
cl1193	ct1283	cn1371	pOP-CEO01884_EST_C_1_pSK_SK	476
cl1193	ct1283	cn1371	pOP-CEO02683_EST_C_1_pSK_SK	420
cl1193	ct1283	cn1371	pOP-CEO02950_EST_C_1_pSK_SK	444
cl1193	ct1283	cn1371	pOP-CEO03461_EST_C_1_pSK_SK	302
cl1193	ct1283	cn1371	pOP-CNH01033_EST_C_1_pSK_SK	579
cl1193	ct1283	cn1371	pOP-CNH01368_EST_C_1_pSK_SK	551
cl1193	ct1283	cn1371	pOP-CNH01993_EST_C_1_pSK_SK	605
cl1193	ct1283	cn1371	pOP-CNH04318	520
cl1193	ct1283	cn1371	pOP-CNHP00375_EST_C_1_pSK_SK	672
cl1193	ct1283	cn1371	pOP-CNIP00863_EST_C_1_pSK_SK	470
cl1193	ct1283	cn1371	pOP-EAP00605_EST_C_1_pBSK_SK	340
cl1193	ct1283	cn1371	pOP-EAP02790_EST_C_1_pBSK_SK	472
cl1193	ct1283	cn1371	pOP-EN00298_EST_C_1_pSK_SK	527
cl1193	ct1283	cn1371	pOP-EN00674_EST_C_1_pSK_SK	386
cl1193	ct1283	cn1371	pOP-EO03343_EST_C_1_pSK_SK	327
cl1193	ct1283	cn1371	pOP-EO06175_EST_C_1_pSK_SK	541
cl1193	ct1283	cn1371	pOP-EO08083_EST_C_1_pSK_SK	519
cl1193	ct1283	cn1372	pOP-EO05762_EST_C_1_pSK_SK	567
cl1194	ct1284	cn1373	pOP-CEO01150_EST_C_1_pSK_SK	408
cl1194	ct1284	cn1373	pOP-CNH03407_EST_C_1_pSK_SK	537
cl1194	ct1284	cn1373	pOP-EAP03133_EST_C_1_pBSK_SK	598
cl1195	ct1285	cn1374	pOP-CEO01157_EST_C_1_pSK_SK	374
cl1195	ct1285	cn1374	pOP-CNI01849_EST_C_1_pSK_SK	215
cl1196	ct1286	cn1375	pOP-CEO01160_EST_C_1_pSK_SK	292
cl1196	ct1286	cn1375	pOP-EAP03389_EST_C_1_pBSK_SK	182
cl1196	ct1286	cn1375	pOP-EAP03644_EST_C_1_pBSK_SK	305
cl1197	ct1287	cn1376	pOP-CEO01166_EST_C_1_pSK_SK	416
cl1197	ct1287	cn1376	pOP-EO08115_EST_C_1_pSK_SK	575
cl1198	ct1288	cn1377	pOP-CEO01169_EST_C_1_pSK_SK	388
cl1198	ct1288	cn1377	pOP-CNIP00732_EST_C_1_pSK_SK	341
cl1199	ct1289	cn1378	pOP-CEO01175_EST_C_1_pSK_SK	588
cl1199	ct1289	cn1378	pOP-CEO01353	393
cl1200	ct1290	cn1379	pOP-CEO01181_EST_C_1_pSK_SK	317
cl1200	ct1290	cn1379	pOP-CEO02422_EST_C_1_pSK_SK	198
cl1201	ct1291	cn1380	pOP-CEO01185_EST_C_1_pSK_SK	194
cl1201	ct1291	cn1380	pOP-CNI01422_EST_C_1_pSK_SK	240
cl1201	ct1291	cn1380	pOP-CNI01685_EST_C_1_pSK_SK	240
cl1202	ct1292	cn1381	pOP-CEO01193_EST_C_1_pSK_SK	238
cl1202	ct1292	cn1381	pOP-CNI01996_EST_C_1_pSK_SK	299
cl1203	ct1293	cn1382	pOP-CEO01200_EST_C_1_pSK_SK	196
cl1203	ct1293	cn1382	pOP-CEO01208_EST_C_1_pSK_SK	196
cl1204	ct1294	cn1383	pOP-CEO00884_EST_C_1_pSK_SK	244

cl1204	ct1294	cn1383	pOP-CEO01209_EST_C_1_pSK_SK	255
cl1204	ct1294	cn1383	pOP-CNI01598_EST_C_1_pSK_SK	308
cl1204	ct1294	cn1383	pOP-CNIP00377_EST_C_1_pSK_SK	440
cl1204	ct1294	cn1383	pOP-EAP01014_EST_C_1_pBSK_SK	251
cl1204	ct1294	cn1383	pOP-EAP01842_EST_C_1_pBSK_SK	586
cl1204	ct1294	cn1383	pOP-EAP02903_EST_C_1_pBSK_SK	530
cl1204	ct1294	cn1383	pOP-EO02522_EST_C_1_pSK_SK	531
cl1205	ct1295	cn1384	pOP-CAP00213_EST_C_1_pBSK_SK	617
cl1205	ct1295	cn1384	pOP-CEO00859_EST_C_1_pSK_SK	342
cl1205	ct1295	cn1384	pOP-CEO01211_EST_C_1_pSK_SK	201
cl1205	ct1295	cn1384	pOP-CEO03203_EST_C_1_pSK_SK	266
cl1205	ct1295	cn1384	pOP-CNH02193_EST_C_1_pSK_SK	606
cl1205	ct1295	cn1384	pOP-CNI01723_EST_C_1_pSK_SK	436
cl1205	ct1295	cn1384	pOP-CNI01758_EST_C_1_pSK_SK	175
cl1205	ct1295	cn1384	pOP-CNI01792_EST_C_1_pSK_SK	169
cl1205	ct1295	cn1384	pOP-CNIP00665_EST_C_1_pSK_SK	209
cl1205	ct1295	cn1384	pOP-EAP02702_EST_C_1_pBSK_SK	682
cl1206	ct1296	cn1385	pOP-CAP00204_EST_C_1_pBSK_SK	578
cl1206	ct1296	cn1385	pOP-CNH01165_EST_C_1_pSK_SK	540
cl1206	ct1297	cn1386	pOP-CEO01213_EST_C_1_pSK_SK	278
cl1206	ct1297	cn1386	pOP-CEO01363_EST_C_1_pSK_SK	453
cl1206	ct1297	cn1386	pOP-CNH02763_EST_C_1_pSK_SK	650
cl1206	ct1297	cn1386	pOP-CNIP00657_EST_C_1_pSK_SK	597
cl1206	ct1297	cn1386	pOP-EAP00506_EST_C_1_pBSK_SK	674
cl1206	ct1297	cn1386	pOP-EAP00515_EST_C_1_pBSK_SK	678
cl1206	ct1297	cn1386	pOP-EAP02972_EST_C_1_pBSK_SK	326
cl1206	ct1297	cn1386	pOP-EAP05051_EST_C_1_pBSK_SK	565
cl1206	ct1297	cn1386	pOP-EO04251_EST_C_1_pSK_SK	341
cl1206	ct1297	cn1386	pOP-EO07755_EST_C_1_pSK_SK	764
cl1206	ct1297	cn1386	pOP-EO07965_EST_C_1_pSK_SK	405
cl1207	ct1298	cn1387	pOP-CEM00121_EST_C_1_pSK_SK	406
cl1207	ct1298	cn1387	pOP-CEO01025_EST_C_1_pSK_SK	344
cl1207	ct1298	cn1387	pOP-CEO01215_EST_C_1_pSK_SK	342
cl1208	ct1299	cn1388	pOP-CEO01240_EST_C_1_pSK_SK	232
cl1208	ct1299	cn1388	pOP-CEO02850_EST_C_1_pSK_SK	452
cl1208	ct1299	cn1388	pOP-EAP00677_EST_C_1_pBSK_SK	326
cl1208	ct1299	cn1388	pOP-EAP01265_EST_C_1_pBSK_SK	522
cl1209	ct1300	cn1389	pOP-CEM00085_EST_C_1_pSK_SK	350
cl1209	ct1300	cn1389	pOP-CEO01248_EST_C_1_pSK_SK	354
cl1210	ct1301	cn1390	pOP-CEO01236_EST_C_1_pSK_SK	213
cl1210	ct1301	cn1390	pOP-CEO01249_EST_C_1_pSK_SK	212
cl1211	ct1302	cn1391	pOP-CEO01374_EST_C_1_pSK_SK	256
cl1211	ct1302	cn1391	pOP-EAP01913_EST_C_1_pBSK_SK	331
cl1212	ct1303	cn1392	pOP-CEO01395_EST_C_1_pSK_SK	297
cl1212	ct1303	cn1392	pOP-EAP05037_EST_C_1_pBSK_SK	363
cl1213	ct1304	cn1393	pOP-CEO01405_EST_C_1_pSK_SK	552
cl1213	ct1304	cn1393	pOP-CNIP00104_EST_C_1_pSK_SK	503
cl1214	ct1305	cn1394	pOP-CEO01407_EST_C_1_pSK_SK	317
cl1214	ct1305	cn1394	pOP-CNH02287_EST_C_1_pSK_SK	682
cl1215	ct1306	cn1395	pOP-CEO01408_EST_C_1_pSK_SK	521
cl1215	ct1306	cn1395	pOP-CNH00780_EST_C_1_pSK_SK	386
cl1215	ct1306	cn1395	pOP-CNH01139_EST_C_1_pSK_SK	270
cl1215	ct1306	cn1395	pOP-CNH02236_EST_C_1_pSK_SK	709
cl1215	ct1306	cn1395	pOP-CNH04525	733
cl1215	ct1306	cn1395	pOP-CNHP00518_EST_C_1_pSK_SK	589
cl1216	ct1307	cn1396	pOP-CEO01411_EST_C_1_pSK_SK	573

cl1216	ct1307	cn1397	pOP-CEO03181_EST_C_1_pSK_SK	492
cl1217	ct1308	cn1398	pOP-CEO01414_EST_C_1_pSK_SK	267
cl1217	ct1308	cn1398	pOP-CNI01674_EST_C_1_pSK_SK	287
cl1217	ct1308	cn1398	pOP-CNIP01005_EST_C_1_pSK_SK	367
cl1217	ct1308	cn1398	pOP-CNLP00014_EST_C_1_pSK_SK	645
cl1217	ct1308	cn1398	pOP-EAP00347_EST_C_1_pBSK_SK	464
cl1217	ct1308	cn1398	pOP-EAP03803_EST_C_1_pBSK_SK	649
cl1217	ct1308	cn1398	pOP-EO06561_EST_C_1_pSK_SK	494
cl1217	ct1308	cn1399	pOP-CNHP00501_EST_C_1_pSK_SK	554
cl1218	ct1309	cn1400	pOP-CEO01422_EST_C_1_pSK_SK	168
cl1218	ct1309	cn1400	pOP-EO06201_EST_C_1_pSK_SK	332
cl1218	ct1309	cn1400	pOP-EO08315_EST_C_1_pSK_SK	557
cl1219	ct1310	cn1401	pOP-CEO01424_EST_C_1_pSK_SK	357
cl1219	ct1310	cn1401	pOP-EAP00805_EST_C_1_pBSK_SK	410
cl1219	ct1310	cn1401	pOP-EAP03189_EST_C_1_pBSK_SK	518
cl1219	ct1310	cn1401	pOP-EAP03402_EST_C_1_pBSK_SK	615
cl1219	ct1310	cn1402	pOP-EO05156_EST_C_1_pSK_SK	533
cl1219	ct1310	cn1403	pOP-CNHP00176_EST_C_1_pSK_SK	519
cl1220	ct1311	cn1404	pOP-CEO01445_EST_C_1_pSK_SK	408
cl1220	ct1311	cn1405	pOP-CEO02596_EST_C_1_pSK_SK	245
cl1221	ct1312	cn1406	pOP-CEO01446_EST_C_1_pSK_SK	318
cl1221	ct1312	cn1406	pOP-CEO03414_EST_C_1_pSK_SK	457
cl1221	ct1312	cn1406	pOP-CNH01507_EST_C_1_pSK_SK	453
cl1221	ct1312	cn1406	pOP-CNH03209_EST_C_1_pSK_SK	475
cl1221	ct1312	cn1406	pOP-CNH03210_EST_C_1_pSK_SK	574
cl1221	ct1312	cn1406	pOP-CNH03778_EST_C_1_pSK_SK	283
cl1221	ct1312	cn1406	pOP-CNH04762_EST_C_1_pSK_SK	581
cl1221	ct1312	cn1406	pOP-CNH04843_EST_C_1_pSK_SK	780
cl1221	ct1312	cn1406	pOP-CNHP00184_EST_C_1_pSK_SK	674
cl1221	ct1312	cn1406	pOP-CNHP00509_EST_C_1_pSK_SK	604
cl1221	ct1312	cn1406	pOP-CNI01403_EST_C_1_pSK_SK	378
cl1221	ct1312	cn1406	pOP-CNI01751_EST_C_1_pSK_SK	378
cl1222	ct1313	cn1407	pOP-CEO01450_EST_C_1_pSK_SK	289
cl1222	ct1313	cn1407	pOP-EAP01493_EST_C_1_pBSK_SK	624
cl1222	ct1313	cn1407	pOP-EAP05030_EST_C_1_pBSK_SK	632
cl1223	ct1314	cn1408	pOP-CEO01324	398
cl1223	ct1314	cn1408	pOP-CEO01451_EST_C_1_pSK_SK	314
cl1224	ct1315	cn1409	pOP-CBP00175_EST_C_1_pBSK_SK	504
cl1224	ct1315	cn1409	pOP-CEO01476_EST_C_1_pSK_SK	431
cl1224	ct1315	cn1409	pOP-CNH05048_EST_C_1_pSK_SK	719
cl1225	ct1316	cn1410	pOP-CEO01477_EST_C_1_pSK_SK	115
cl1225	ct1316	cn1410	pOP-CEO02750_EST_C_1_pSK_SK	335
cl1225	ct1316	cn1410	pOP-CNH01977_EST_C_1_pSK_SK	207
cl1225	ct1316	cn1410	pOP-CNH02390_EST_C_1_pSK_SK	529
cl1225	ct1316	cn1410	pOP-CNH02993_EST_C_1_pSK_SK	544
cl1226	ct1317	cn1411	pOP-CEO01481_EST_C_1_pSK_SK	284
cl1226	ct1317	cn1411	pOP-CNI01662_EST_C_1_pSK_SK	215
cl1227	ct1318	cn1412	pOP-CEO01482_EST_C_1_pSK_SK	399
cl1227	ct1318	cn1412	pOP-EAP03830_EST_C_1_pBSK_SK	429
cl1227	ct1318	cn1412	pOP-EAP03841_EST_C_1_pBSK_SK	500
cl1228	ct1319	cn1413	pOP-CEO01489_EST_C_1_pSK_SK	339
cl1228	ct1319	cn1413	pOP-CNI01503_EST_C_1_pSK_SK	378
cl1229	ct1320	cn1414	pOP-CEO01491_EST_C_1_pSK_SK	300
cl1229	ct1320	cn1414	pOP-EAP03363_EST_C_1_pBSK_SK	399
cl1230	ct1321	cn1415	pOP-CEM00041_EST_C_1_pSK_SK	355
cl1230	ct1321	cn1415	pOP-CEO01500_EST_C_1_pSK_SK	106

cl1230	ct1321	cn1415	pOP-CNHP00415_EST_C_1_pSK_SK	466
cl1231	ct1322	cn1416	pOP-CEO01502_EST_C_1_pSK_SK	387
cl1231	ct1322	cn1416	pOP-EAP02194_EST_C_1_pBSK_SK	144
cl1232	ct1323	cn1417	pOP-CEO01507_EST_C_1_pSK_SK	570
cl1232	ct1323	cn1417	pOP-CNH01897_EST_C_1_pSK_SK	633
cl1233	ct1324	cn1418	pOP-CEO03452_EST_C_1_pSK_SK	465
cl1233	ct1324	cn1419	pOP-CEO01529_EST_C_1_pSK_SK	389
cl1234	ct1325	cn1420	pOP-CEO01533_EST_C_1_pSK_SK	195
cl1234	ct1325	cn1420	pOP-CEO01534_EST_C_1_pSK_SK	195
cl1235	ct1326	cn1421	pOP-CEO01538_EST_C_1_pSK_SK	332
cl1235	ct1326	cn1421	pOP-CNI02158_EST_C_1_pSK_SK	628
cl1235	ct1326	cn1421	pOP-CNIP00436_EST_C_1_pSK_SK	637
cl1235	ct1326	cn1421	pOP-EAP02202_EST_C_1_pBSK_SK	417
cl1236	ct1327	cn1422	pOP-CEO01540_EST_C_1_pSK_SK	125
cl1236	ct1327	cn1422	pOP-CNIP00771_EST_C_1_pSK_SK	238
cl1236	ct1327	cn1422	pOP-CNIP00791_EST_C_1_pSK_SK	300
cl1237	ct1328	cn1423	pOP-CEO01553_EST_C_1_pSK_SK	135
cl1237	ct1328	cn1423	pOP-EO06225_EST_C_1_pSK_SK	267
cl1238	ct1329	cn1424	pOP-CEO00860_EST_C_1_pSK_SK	443
cl1238	ct1329	cn1424	pOP-CEO01557_EST_C_1_pSK_SK	496
cl1239	ct1330	cn1425	pOP-CEO01567_EST_C_1_pSK_SK	239
cl1239	ct1330	cn1425	pOP-EAP03628_EST_C_1_pBSK_SK	353
cl1240	ct1331	cn1426	pOP-CEM00092_EST_C_1_pSK_SK	572
cl1240	ct1331	cn1426	pOP-CEO01572_EST_C_1_pSK_SK	265
cl1240	ct1331	cn1426	pOP-EAP02865_EST_C_1_pBSK_SK	349
cl1240	ct1331	cn1426	pOP-EAP03750_EST_C_1_pBSK_SK	479
cl1241	ct1332	cn1427	pOP-CEO01585_EST_C_1_pSK_SK	247
cl1241	ct1332	cn1427	pOP-EAP01615_EST_C_1_pBSK_SK	478
cl1242	ct1333	cn1428	pOP-CEO01593_EST_C_1_pSK_SK	295
cl1242	ct1333	cn1428	pOP-EAP00861_EST_C_1_pBSK_SK	565
cl1243	ct1334	cn1429	pOP-CEO01448_EST_C_1_pSK_SK	260
cl1243	ct1334	cn1429	pOP-CEO01594_EST_C_1_pSK_SK	224
cl1243	ct1334	cn1429	pOP-CNH00898_EST_C_1_pSK_SK	406
cl1244	ct1335	cn1430	pOP-CEO00523_EST_C_1_pSK_SK	302
cl1244	ct1335	cn1430	pOP-CEO01599_EST_C_1_pSK_SK	337
cl1245	ct1336	cn1431	pOP-CEO01606_EST_C_1_pSK_SK	512
cl1245	ct1336	cn1431	pOP-EAP00657_EST_C_1_pBSK_SK	224
cl1246	ct1337	cn1432	pOP-CEM00138_EST_C_1_pSK_SK	139
cl1246	ct1337	cn1432	pOP-CEO01607_EST_C_1_pSK_SK	287
cl1247	ct1338	cn1433	pOP-CEO01610_EST_C_1_pSK_SK	363
cl1247	ct1338	cn1433	pOP-CNH00721_EST_C_1_pSK_SK	514
cl1248	ct1339	cn1434	pOP-CEO01635_EST_C_1_pSK_SK	205
cl1248	ct1339	cn1434	pOP-EAP01429_EST_C_1_pBSK_SK	277
cl1248	ct1339	cn1434	pOP-EO03527_EST_C_1_pSK_SK	288
cl1249	ct1340	cn1435	pOP-CEO01640_EST_C_1_pSK_SK	240
cl1249	ct1340	cn1435	pOP-EAP00495_EST_C_1_pBSK_SK	186
cl1250	ct1341	cn1436	pOP-CEO01645_EST_C_1_pSK_SK	331
cl1250	ct1341	cn1436	pOP-EAP03559_EST_C_1_pBSK_SK	221
cl1251	ct1342	cn1437	pOP-CEO01647_EST_C_1_pSK_SK	224
cl1251	ct1342	cn1437	pOP-EAP00829_EST_C_1_pBSK_SK	435
cl1252	ct1343	cn1438	pOP-CEO01653_EST_C_1_pSK_SK	441
cl1252	ct1343	cn1438	pOP-EAP03672_EST_C_1_pBSK_SK	331
cl1253	ct1344	cn1439	pOP-CEO01657_EST_C_1_pSK_SK	251
cl1253	ct1344	cn1439	pOP-CNI02054_EST_C_1_pSK_SK	490
cl1254	ct1345	cn1440	pOP-CEM00148_EST_C_1_pSK_SK	134
cl1254	ct1345	cn1440	pOP-CEM00179_EST_C_1_pSK_SK	134

cl1254	ct1345	cn1440	pOP-CEO01659_EST_C_1_pSK_SK	225
cl1254	ct1345	cn1440	pOP-CNI01230_EST_C_1_pSK_SK	535
cl1254	ct1345	cn1440	pOP-CNI02004_EST_C_1_pSK_SK	141
cl1254	ct1345	cn1440	pOP-EO04569_EST_C_1_pSK_SK	434
cl1254	ct1346	cn1441	pOP-CEO01573_EST_C_1_pSK_SK	391
cl1254	ct1346	cn1441	pOP-CEO03587_EST_C_1_pSK_SK	164
cl1254	ct1346	cn1441	pOP-CNH02823_EST_C_1_pSK_SK	594
cl1254	ct1346	cn1441	pOP-CNH03079_EST_C_1_pSK_SK	513
cl1254	ct1346	cn1441	pOP-CNI01954_EST_C_1_pSK_SK	377
cl1254	ct1346	cn1441	pOP-EAP00646_EST_C_1_pBSK_SK	615
cl1254	ct1346	cn1441	pOP-EAP01982_EST_C_1_pBSK_SK	207
cl1254	ct1346	cn1441	pOP-EAP02825_EST_C_1_pBSK_SK	563
cl1254	ct1346	cn1441	pOP-EAP03729_EST_C_1_pBSK_SK	331
cl1254	ct1346	cn1441	pOP-EO07902_EST_C_1_pSK_SK	670
cl1254	ct1346	cn1441	pOP-EO07921_EST_C_1_pSK_SK	601
cl1255	ct1347	cn1442	pOP-CNH02315_EST_C_1_pSK_SK	693
cl1255	ct1347	cn1442	pOP-CNIP00361_EST_C_1_pSK_SK	501
cl1255	ct1348	cn1443	pOP-CEO01666_EST_C_1_pSK_SK	554
cl1255	ct1348	cn1443	pOP-CNH01862_EST_C_1_pSK_SK	420
cl1255	ct1348	cn1443	pOP-CNH02761_EST_C_1_pSK_SK	624
cl1256	ct1349	cn1444	pOP-CEO01667_EST_C_1_pSK_SK	499
cl1256	ct1349	cn1444	pOP-CNIP00789_EST_C_1_pSK_SK	350
cl1257	ct1350	cn1445	pOP-CEO01673_EST_C_1_pSK_SK	512
cl1257	ct1350	cn1446	pOP-EAP02242_EST_C_1_pBSK_SK	315
cl1258	ct1351	cn1447	pOP-CEO01682_EST_C_1_pSK_SK	407
cl1258	ct1351	cn1447	pOP-EAP03418_EST_C_1_pBSK_SK	386
cl1259	ct1352	cn1448	pOP-CEO01691_EST_C_1_pSK_SK	639
cl1259	ct1352	cn1448	pOP-CNI01405_EST_C_1_pSK_SK	384
cl1259	ct1352	cn1449	pOP-CEO01629_EST_C_1_pSK_SK	394
cl1260	ct1353	cn1450	pOP-CEO01217_EST_C_1_pSK_SK	167
cl1260	ct1353	cn1450	pOP-CEO01695_EST_C_1_pSK_SK	437
cl1261	ct1354	cn1451	pOP-CEO01690_EST_C_1_pSK_SK	437
cl1261	ct1354	cn1451	pOP-CEO01707_EST_C_1_pSK_SK	439
cl1262	ct1355	cn1452	pOP-CEO01373_EST_C_1_pSK_SK	378
cl1262	ct1355	cn1452	pOP-CEO01720_EST_C_1_pSK_SK	453
cl1262	ct1355	cn1452	pOP-EAP00325_EST_C_1_pBSK_SK	342
cl1262	ct1355	cn1452	pOP-EAP00327_EST_C_1_pBSK_SK	342
cl1263	ct1356	cn1453	pOP-CEO01719_EST_C_1_pSK_SK	457
cl1263	ct1356	cn1453	pOP-CEO01721_EST_C_1_pSK_SK	453
cl1264	ct1357	cn1454	pOP-CEO01722_EST_C_1_pSK_SK	278
cl1264	ct1357	cn1454	pOP-CNI01664_EST_C_1_pSK_SK	301
cl1265	ct1358	cn1455	pOP-CEO01731_EST_C_1_pSK_SK	370
cl1265	ct1358	cn1455	pOP-CNIP00709_EST_C_1_pSK_SK	354
cl1265	ct1358	cn1455	pOP-EAP01198_EST_C_1_pBSK_SK	190
cl1265	ct1358	cn1455	pOP-EAP01237_EST_C_1_pBSK_SK	221
cl1266	ct1359	cn1456	pOP-CEO01738_EST_C_1_pSK_SK	244
cl1266	ct1359	cn1456	pOP-CNH03139_EST_C_1_pSK_SK	465
cl1266	ct1359	cn1456	pOP-CNH03248_EST_C_1_pSK_SK	413
cl1266	ct1359	cn1456	pOP-CNI02056_EST_C_1_pSK_SK	210
cl1267	ct1360	cn1457	pOP-CEO01741_EST_C_1_pSK_SK	211
cl1267	ct1360	cn1457	pOP-EAP03201_EST_C_1_pBSK_SK	683
cl1268	ct1361	cn1458	pOP-CEO01747_EST_C_1_pSK_SK	225
cl1268	ct1361	cn1458	pOP-CNI01379_EST_C_1_pSK_SK	289
cl1269	ct1362	cn1459	pOP-CEO01748_EST_C_1_pSK_SK	356
cl1269	ct1362	cn1459	pOP-CNIP00926_EST_C_1_pSK_SK	707
cl1270	ct1363	cn1460	pOP-CEO01753_EST_C_1_pSK_SK	338

cl1270	ct1363	cn1460	pOP-EAP01083_EST_C_1_pBSK_SK	341
cl1270	ct1363	cn1460	pOP-EAP02894_EST_C_1_pBSK_SK	289
cl1271	ct1364	cn1461	pOP-CEO01778_EST_C_1_pSK_SK	368
cl1271	ct1364	cn1461	pOP-CNI01745_EST_C_1_pSK_SK	487
cl1271	ct1364	cn1461	pOP-EAP00245_EST_C_1_pBSK_SK	146
cl1271	ct1364	cn1461	pOP-EAP01713_EST_C_1_pBSK_SK	643
cl1271	ct1364	cn1461	pOP-EAP02909_EST_C_1_pBSK_SK	351
cl1271	ct1364	cn1461	pOP-EAP03288_EST_C_1_pBSK_SK	461
cl1271	ct1364	cn1461	pOP-EAP03671_EST_C_1_pBSK_SK	486
cl1272	ct1365	cn1462	pOP-CEO01781_EST_C_1_pSK_SK	357
cl1272	ct1365	cn1462	pOP-EO08251_EST_C_1_pSK_SK	397
cl1273	ct1366	cn1463	pOP-CEO01798_EST_C_1_pSK_SK	361
cl1273	ct1366	cn1463	pOP-CNH04388	671
cl1273	ct1366	cn1463	pOP-CNI01378_EST_C_1_pSK_SK	529
cl1273	ct1366	cn1463	pOP-CNI02088_EST_C_1_pSK_SK	504
cl1273	ct1366	cn1463	pOP-CNI02209_EST_C_1_pSK_SK	361
cl1273	ct1366	cn1463	pOP-EAP01050_EST_C_1_pBSK_SK	304
cl1273	ct1366	cn1463	pOP-EAP02879_EST_C_1_pBSK_SK	253
cl1274	ct1367	cn1464	pOP-CEO01805_EST_C_1_pSK_SK	352
cl1274	ct1367	cn1464	pOP-CNH02700_EST_C_1_pSK_SK	638
cl1275	ct1368	cn1465	pOP-CEO01811_EST_C_1_pSK_SK	256
cl1275	ct1368	cn1465	pOP-EO02676_EST_C_1_pSK_SK	358
cl1275	ct1368	cn1465	pOP-EO03407_EST_C_1_pSK_SK	448
cl1276	ct1369	cn1466	pOP-CEO01812_EST_C_1_pSK_SK	354
cl1276	ct1369	cn1466	pOP-CNIP01056_EST_C_1_pSK_SK	431
cl1277	ct1370	cn1467	pOP-CNH01442_EST_C_1_pSK_SK	633
cl1277	ct1370	cn1467	pOP-CNH01497_EST_C_1_pSK_SK	572
cl1277	ct1370	cn1467	pOP-CNH04655	810
cl1277	ct1370	cn1467	pOP-CNH04656	778
cl1277	ct1371	cn1468	pOP-CEO01814_EST_C_1_pSK_SK	168
cl1277	ct1371	cn1468	pOP-CEO02723_EST_C_1_pSK_SK	371
cl1277	ct1371	cn1468	pOP-EAP00920_EST_C_1_pBSK_SK	283
cl1277	ct1371	cn1468	pOP-EAP02787_EST_C_1_pBSK_SK	273
cl1278	ct1372	cn1469	pOP-CEO01816_EST_C_1_pSK_SK	114
cl1278	ct1372	cn1469	pOP-EAP00527_EST_C_1_pBSK_SK	383
cl1278	ct1372	cn1469	pOP-EAP03390_EST_C_1_pBSK_SK	334
cl1279	ct1373	cn1470	pOP-CEO01819_EST_C_1_pSK_SK	365
cl1279	ct1373	cn1470	pOP-CNI02239_EST_C_1_pSK_SK	296
cl1280	ct1374	cn1471	pOP-CEO01821_EST_C_1_pSK_SK	238
cl1280	ct1374	cn1471	pOP-CNIP00021_EST_C_1_pSK_SK	259
cl1281	ct1375	cn1472	pOP-CEO01826_EST_C_1_pSK_SK	382
cl1281	ct1375	cn1472	pOP-EAP02210_EST_C_1_pBSK_SK	392
cl1282	ct1376	cn1473	pOP-CEO01828_EST_C_1_pSK_SK	121
cl1282	ct1376	cn1473	pOP-CEO01829_EST_C_1_pSK_SK	121
cl1283	ct1377	cn1474	pOP-CEO01846_EST_C_1_pSK_SK	243
cl1283	ct1377	cn1474	pOP-CNI01436_EST_C_1_pSK_SK	603
cl1284	ct1378	cn1475	pOP-CEO01869_EST_C_1_pSK_SK	228
cl1284	ct1378	cn1475	pOP-CNH03428_EST_C_1_pSK_SK	606
cl1285	ct1379	cn1476	pOP-CEO01872_EST_C_1_pSK_SK	300
cl1285	ct1379	cn1476	pOP-EAP00342_EST_C_1_pBSK_SK	527
cl1285	ct1379	cn1476	pOP-EAP00343_EST_C_1_pBSK_SK	526
cl1285	ct1379	cn1476	pOP-EAP02847_EST_C_1_pBSK_SK	568
cl1286	ct1380	cn1477	pOP-CEO01873_EST_C_1_pSK_SK	263
cl1286	ct1380	cn1477	pOP-CNH01709_EST_C_1_pSK_SK	464
cl1286	ct1380	cn1477	pOP-CNH02421_EST_C_1_pSK_SK	530
cl1287	ct1381	cn1478	pOP-CEO01893_EST_C_1_pSK_SK	175

cl1287	ct1381	cn1478	pOP-CNI01720_EST_C_1_pSK_SK	105
cl1288	ct1382	cn1479	pOP-CEO01416_EST_C_1_pSK_SK	567
cl1288	ct1382	cn1479	pOP-CEO01801_EST_C_1_pSK_SK	360
cl1288	ct1382	cn1479	pOP-CEO01908_EST_C_1_pSK_SK	382
cl1288	ct1382	cn1479	pOP-CNH03165_EST_C_1_pSK_SK	507
cl1288	ct1382	cn1479	pOP-CNIP00082_EST_C_1_pSK_SK	608
cl1289	ct1383	cn1480	pOP-CEO00827_EST_C_1_pSK_SK	317
cl1289	ct1383	cn1480	pOP-CEO01913_EST_C_1_pSK_SK	329
cl1289	ct1383	cn1480	pOP-CEO03074_EST_C_1_pSK_SK	759
cl1290	ct1384	cn1481	pOP-CEO01102_EST_C_1_pSK_SK	378
cl1290	ct1384	cn1481	pOP-CEO01941_EST_C_1_pSK_SK	280
cl1291	ct1385	cn1482	pOP-CEO01112_EST_C_1_pSK_SK	201
cl1291	ct1385	cn1482	pOP-CEO01944_EST_C_1_pSK_SK	201
cl1291	ct1386	cn1483	pOP-CEMP00010_EST_C_1_pSK_SK	412
cl1291	ct1386	cn1483	pOP-CNH00623_EST_C_1_pSK_SK	465
cl1291	ct1386	cn1483	pOP-CNH00724_EST_C_1_pSK_SK	288
cl1292	ct1387	cn1484	pOP-CEO01945_EST_C_1_pSK_SK	553
cl1292	ct1387	cn1484	pOP-EAP03256_EST_C_1_pBSK_SK	638
cl1293	ct1388	cn1485	pOP-CEO01950_EST_C_1_pSK_SK	116
cl1293	ct1388	cn1485	pOP-EAP01859_EST_C_1_pBSK_SK	476
cl1294	ct1389	cn1486	pOP-CEO01954_EST_C_1_pSK_SK	529
cl1294	ct1389	cn1486	pOP-CNH02304_EST_C_1_pSK_SK	654
cl1295	ct1390	cn1487	pOP-CEO01961_EST_C_1_pSK_SK	202
cl1295	ct1390	cn1487	pOP-CNI02029_EST_C_1_pSK_SK	264
cl1296	ct1391	cn1488	pOP-CEO01983_EST_C_1_pSK_SK	467
cl1296	ct1391	cn1488	pOP-CNH00622_EST_C_1_pSK_SK	616
cl1297	ct1392	cn1489	pOP-CNH00803_EST_C_1_pSK_SK	569
cl1297	ct1392	cn1489	pOP-CNH01701_EST_C_1_pSK_SK	553
cl1297	ct1392	cn1490	pOP-CEO01985_EST_C_1_pSK_SK	489
cl1298	ct1393	cn1491	pOP-CEO01938_EST_C_1_pSK_SK	239
cl1298	ct1393	cn1491	pOP-CEO01986_EST_C_1_pSK_SK	372
cl1298	ct1393	cn1492	pOP-CEM00241_EST_C_1_pSK_SK	260
cl1298	ct1393	cn1492	pOP-EO08436_EST_C_1_pSK_SK	391
cl1299	ct1394	cn1493	pOP-CEO01212_EST_C_1_pSK_SK	473
cl1299	ct1394	cn1493	pOP-CEO01994_EST_C_1_pSK_SK	319
cl1299	ct1394	cn1493	pOP-CNH04647	803
cl1299	ct1394	cn1493	pOP-CNHP00042_EST_C_1_pSK_SK	517
cl1299	ct1394	cn1493	pOP-EAP03404_EST_C_1_pBSK_SK	373
cl1300	ct1395	cn1494	pOP-CEO01996_EST_C_1_pSK_SK	272
cl1300	ct1395	cn1494	pOP-EO07758_EST_C_1_pSK_SK	713
cl1301	ct1396	cn1495	pOP-CEO00590_EST_C_1_pSK_SK	563
cl1301	ct1396	cn1495	pOP-CEO02006_EST_C_1_pSK_SK	378
cl1302	ct1397	cn1496	pOP-CEO02018_EST_C_1_pSK_SK	377
cl1302	ct1397	cn1496	pOP-CEO02019_EST_C_1_pSK_SK	377
cl1303	ct1398	cn1497	pOP-CEO01646_EST_C_1_pSK_SK	579
cl1303	ct1398	cn1497	pOP-CEO02020_EST_C_1_pSK_SK	380
cl1305	ct1400	cn1499	pOP-CEO02039_EST_C_1_pSK_SK	338
cl1305	ct1400	cn1499	pOP-CNIP00329_EST_C_1_pSK_SK	367
cl1306	ct1401	cn1500	pOP-CEO02042_EST_C_1_pSK_SK	346
cl1306	ct1401	cn1500	pOP-CNH02401_EST_C_1_pSK_SK	385
cl1307	ct1402	cn1501	pOP-CEO02043_EST_C_1_pSK_SK	282
cl1307	ct1402	cn1501	pOP-CNHP00364_EST_C_1_pSK_SK	646
cl1308	ct1403	cn1502	pOP-CEO02049_EST_C_1_pSK_SK	332
cl1308	ct1403	cn1502	pOP-EO05565_EST_C_1_pSK_SK	455
cl1309	ct1404	cn1503	pOP-CEO02060_EST_C_1_pSK_SK	265
cl1309	ct1404	cn1503	pOP-CNHP00030_EST_C_1_pSK_SK	486

cl1310	ct1405	cn1504	pOP-CEO02072_EST_C_1_pSK_SK	249
cl1310	ct1405	cn1504	pOP-CEO02073_EST_C_1_pSK_SK	249
cl1311	ct1406	cn1505	pOP-CEO02088_EST_C_1_pSK_SK	454
cl1311	ct1406	cn1506	pOP-CEO02089_EST_C_1_pSK_SK	431
cl1312	ct1407	cn1507	pOP-CEO02092_EST_C_1_pSK_SK	303
cl1312	ct1407	cn1507	pOP-CNH03379_EST_C_1_pSK_SK	574
cl1313	ct1408	cn1508	pOP-CEO02093_EST_C_1_pSK_SK	213
cl1313	ct1408	cn1508	pOP-CNIP00745_EST_C_1_pSK_SK	205
cl1314	ct1409	cn1509	pOP-CBP00245_EST_C_1_pBSK_SK	429
cl1314	ct1409	cn1509	pOP-CEO00847_EST_C_1_pSK_SK	256
cl1314	ct1409	cn1509	pOP-CEO02103_EST_C_1_pSK_SK	410
cl1315	ct1410	cn1510	pOP-CNH01943_EST_C_1_pSK_SK	692
cl1315	ct1410	cn1510	pOP-EAP03802_EST_C_1_pBSK_SK	695
cl1315	ct1410	cn1511	pOP-CEO02112_EST_C_1_pSK_SK	426
cl1316	ct1411	cn1512	pOP-CEO02115_EST_C_1_pSK_SK	173
cl1316	ct1411	cn1512	pOP-CNIP00239_EST_C_1_pSK_SK	283
cl1317	ct1412	cn1513	pOP-CEO01148_EST_C_1_pSK_SK	419
cl1317	ct1412	cn1513	pOP-CEO02134_EST_C_1_pSK_SK	265
cl1318	ct1413	cn1514	pOP-CEO02136_EST_C_1_pSK_SK	190
cl1318	ct1413	cn1514	pOP-CNIP00879_EST_C_1_pSK_SK	258
cl1318	ct1413	cn1514	pOP-EO07355_EST_C_1_pSK_SK	721
cl1318	ct1413	cn1515	pOP-EAP00618_EST_C_1_pBSK_SK	436
cl1318	ct1413	cn1515	pOP-EAP00619_EST_C_1_pBSK_SK	407
cl1318	ct1413	cn1515	pOP-EAP03608_EST_C_1_pBSK_SK	297
cl1319	ct1414	cn1516	pOP-CEO02144_EST_C_1_pSK_SK	587
cl1319	ct1414	cn1516	pOP-CNH01346_EST_C_1_pSK_SK	719
cl1319	ct1414	cn1516	pOP-EAP01618_EST_C_1_pBSK_SK	453
cl1320	ct1415	cn1517	pOP-CEO02155_EST_C_1_pSK_SK	384
cl1320	ct1415	cn1517	pOP-CEO02157_EST_C_1_pSK_SK	384
cl1321	ct1416	cn1518	pOP-CEO02162_EST_C_1_pSK_SK	397
cl1321	ct1417	cn1519	pOP-CEO02161_EST_C_1_pSK_SK	167
cl1321	ct1417	cn1519	pOP-EAP01749_EST_C_1_pBSK_SK	708
cl1321	ct1417	cn1519	pOP-EAP02981_EST_C_1_pBSK_SK	502
cl1322	ct1418	cn1520	pOP-CEO02164_EST_C_1_pSK_SK	599
cl1322	ct1418	cn1520	pOP-CNH02828_EST_C_1_pSK_SK	600
cl1323	ct1419	cn1521	pOP-CEM00094_EST_C_1_pSK_SK	362
cl1323	ct1419	cn1521	pOP-CEM00166_EST_C_1_pSK_SK	362
cl1323	ct1419	cn1521	pOP-CEO02171_EST_C_1_pSK_SK	319
cl1323	ct1419	cn1521	pOP-CNH01180_EST_C_1_pSK_SK	449
cl1323	ct1419	cn1521	pOP-EAP02380_EST_C_1_pBSK_SK	422
cl1323	ct1419	cn1521	pOP-EAP03563_EST_C_1_pBSK_SK	214
cl1324	ct1420	cn1522	pOP-CEO01056_EST_C_1_pSK_SK	379
cl1324	ct1420	cn1522	pOP-CEO02190_EST_C_1_pSK_SK	312
cl1325	ct1421	cn1523	pOP-CEO02191_EST_C_1_pSK_SK	640
cl1325	ct1421	cn1523	pOP-CNH01768_EST_C_1_pSK_SK	542
cl1325	ct1421	cn1523	pOP-CNH04790_EST_C_1_pSK_SK	455
cl1326	ct1422	cn1524	pOP-CEO02198_EST_C_1_pSK_SK	234
cl1326	ct1422	cn1524	pOP-EAP01280_EST_C_1_pBSK_SK	373
cl1327	ct1423	cn1525	pOP-CEO02203_EST_C_1_pSK_SK	482
cl1327	ct1423	cn1526	pOP-CBP00185_EST_C_1_pBSK_SK	449
cl1328	ct1424	cn1527	pOP-CEO02214_EST_C_1_pSK_SK	362
cl1328	ct1424	cn1527	pOP-CNI01827_EST_C_1_pSK_SK	402
cl1328	ct1424	cn1527	pOP-CNIP00272_EST_C_1_pSK_SK	387
cl1329	ct1425	cn1528	pOP-CEO02216_EST_C_1_pSK_SK	723
cl1329	ct1425	cn1528	pOP-EAP02717_EST_C_1_pBSK_SK	497
cl1330	ct1426	cn1529	pOP-CAP00211_EST_C_1_pBSK_SK	590

cl1330	ct1426	cn1529	pOP-CAP00212_EST_C_1_pBSK_SK	601
cl1330	ct1426	cn1529	pOP-CAP00287_EST_C_1_pBSK_SK	547
cl1330	ct1426	cn1529	pOP-EO02282_EST_C_1_pSK_SK	531
cl1330	ct1427	cn1530	pOP-CEO01303	167
cl1330	ct1427	cn1530	pOP-CEO01833_EST_C_1_pSK_SK	253
cl1330	ct1427	cn1530	pOP-EAP01625_EST_C_1_pBSK_SK	513
cl1330	ct1427	cn1530	pOP-EO06983_EST_C_1_pSK_SK	686
cl1330	ct1427	cn1531	pOP-CEO02399_EST_C_1_pSK_SK	459
cl1330	ct1427	cn1531	pOP-EAP02074_EST_C_1_pBSK_SK	381
cl1330	ct1427	cn1532	pOP-CEO03472_EST_C_1_pSK_SK	405
cl1330	ct1428	cn1533	pOP-CEO02217_EST_C_1_pSK_SK	614
cl1330	ct1428	cn1533	pOP-CEO03418_EST_C_1_pSK_SK	212
cl1330	ct1428	cn1533	pOP-CNI01770_EST_C_1_pSK_SK	400
cl1330	ct1428	cn1533	pOP-EAP01203_EST_C_1_pBSK_SK	184
cl1330	ct1428	cn1534	pOP-EAP03715_EST_C_1_pBSK_SK	573
cl1330	ct1428	cn1534	pOP-EO04692_EST_C_1_pSK_SK	504
cl1330	ct1428	cn1534	pOP-EO06565_EST_C_1_pSK_SK	648
cl1330	ct1429	cn1535	pOP-CEM00040_EST_C_1_pSK_SK	231
cl1330	ct1429	cn1535	pOP-CEO00841_EST_C_1_pSK_SK	433
cl1330	ct1429	cn1535	pOP-CEO01309	317
cl1330	ct1429	cn1535	pOP-CEO01503_EST_C_1_pSK_SK	258
cl1330	ct1429	cn1535	pOP-CEO02331_EST_C_1_pSK_SK	253
cl1330	ct1429	cn1535	pOP-CEO02559_EST_C_1_pSK_SK	195
cl1330	ct1429	cn1535	pOP-CEO02608_EST_C_1_pSK_SK	147
cl1330	ct1429	cn1535	pOP-CEO02647_EST_C_1_pSK_SK	196
cl1330	ct1429	cn1535	pOP-CEO02699_EST_C_1_pSK_SK	354
cl1330	ct1429	cn1535	pOP-CEO02829_EST_C_1_pSK_SK	361
cl1330	ct1429	cn1535	pOP-CEO03011_EST_C_1_pSK_SK	220
cl1330	ct1429	cn1535	pOP-CEO03027_EST_C_1_pSK_SK	216
cl1330	ct1429	cn1535	pOP-CEO03097_EST_C_1_pSK_SK	222
cl1330	ct1429	cn1535	pOP-CEO03241_EST_C_1_pSK_SK	137
cl1330	ct1429	cn1535	pOP-EAP01109_EST_C_1_pBSK_SK	236
cl1330	ct1429	cn1535	pOP-EAP01856_EST_C_1_pBSK_SK	310
cl1330	ct1429	cn1535	pOP-EAP02095_EST_C_1_pBSK_SK	260
cl1330	ct1429	cn1535	pOP-EAP03711_EST_C_1_pBSK_SK	388
cl1330	ct1429	cn1535	pOP-EO02058_EST_C_1_pSK_SK	552
cl1330	ct1429	cn1535	pOP-EO02181_EST_C_1_pSK_SK	555
cl1330	ct1429	cn1535	pOP-EO02749_EST_C_1_pSK_SK	438
cl1330	ct1429	cn1535	pOP-EO02811_EST_C_1_pSK_SK	447
cl1330	ct1429	cn1535	pOP-EO02887_EST_C_1_pSK_SK	464
cl1330	ct1429	cn1535	pOP-EO02941_EST_C_1_pSK_SK	329
cl1330	ct1429	cn1535	pOP-EO03079_EST_C_1_pSK_SK	476
cl1330	ct1429	cn1535	pOP-EO03299_EST_C_1_pSK_SK	259
cl1330	ct1429	cn1535	pOP-EO03579_EST_C_1_pSK_SK	533
cl1330	ct1429	cn1535	pOP-EO03795_EST_C_1_pSK_SK	527
cl1330	ct1429	cn1535	pOP-EO04272_EST_C_1_pSK_SK	429
cl1330	ct1429	cn1535	pOP-EO04517_EST_C_1_pSK_SK	538
cl1330	ct1429	cn1535	pOP-EO04520_EST_C_1_pSK_SK	525
cl1330	ct1429	cn1535	pOP-EO04919_EST_C_1_pSK_SK	473
cl1330	ct1429	cn1535	pOP-EO04924_EST_C_1_pSK_SK	523
cl1330	ct1429	cn1535	pOP-EO04943_EST_C_1_pSK_SK	515
cl1330	ct1429	cn1535	pOP-EO04953_EST_C_1_pSK_SK	519
cl1330	ct1429	cn1535	pOP-EO05021_EST_C_1_pSK_SK	538
cl1330	ct1429	cn1535	pOP-EO05423_EST_C_1_pSK_SK	531
cl1330	ct1429	cn1535	pOP-EO05559_EST_C_1_pSK_SK	466
cl1330	ct1429	cn1535	pOP-EO05650_EST_C_1_pSK_SK	467

cl1330	ct1429	cn1535	pOP-EO05698_EST_C_1_pSK_SK	517
cl1330	ct1429	cn1535	pOP-EO05777_EST_C_1_pSK_SK	648
cl1330	ct1429	cn1535	pOP-EO06025_EST_C_1_pSK_SK	509
cl1330	ct1429	cn1535	pOP-EO06418_EST_C_1_pSK_SK	732
cl1330	ct1429	cn1536	pOP-CEO03710_EST_C_1_pSK_SK	404
cl1330	ct1429	cn1537	pOP-EAP02094_EST_C_1_pBSK_SK	289
cl1331	ct1430	cn1538	pOP-CEO02220_EST_C_1_pSK_SK	294
cl1331	ct1430	cn1538	pOP-CNIP00202_EST_C_1_pSK_SK	325
cl1331	ct1430	cn1538	pOP-EAP00300_EST_C_1_pBSK_SK	336
cl1332	ct1431	cn1539	pOP-CEO02223_EST_C_1_pSK_SK	389
cl1332	ct1431	cn1539	pOP-EAP01101_EST_C_1_pBSK_SK	279
cl1332	ct1431	cn1539	pOP-EAP02899_EST_C_1_pBSK_SK	278
cl1333	ct1432	cn1540	pOP-CEO02226_EST_C_1_pSK_SK	278
cl1333	ct1432	cn1540	pOP-CNI02042_EST_C_1_pSK_SK	543
cl1333	ct1432	cn1540	pOP-CNIP00338_EST_C_1_pSK_SK	346
cl1334	ct1433	cn1541	pOP-CNI01784_EST_C_1_pSK_SK	526
cl1334	ct1434	cn1542	pOP-CEO02237_EST_C_1_pSK_SK	614
cl1335	ct1435	cn1543	pOP-CEO02001_EST_C_1_pSK_SK	362
cl1335	ct1435	cn1543	pOP-CEO02246_EST_C_1_pSK_SK	571
cl1336	ct1436	cn1544	pOP-CEO01077_EST_C_1_pSK_SK	334
cl1336	ct1436	cn1544	pOP-CEO02248_EST_C_1_pSK_SK	470
cl1336	ct1436	cn1544	pOP-EAP02017_EST_C_1_pBSK_SK	223
cl1337	ct1437	cn1545	pOP-CEO02249_EST_C_1_pSK_SK	735
cl1337	ct1437	cn1545	pOP-CNH01079_EST_C_1_pSK_SK	107
cl1337	ct1437	cn1545	pOP-CNH03178_EST_C_1_pSK_SK	383
cl1337	ct1437	cn1545	pOP-EAP01509_EST_C_1_pBSK_SK	633
cl1338	ct1438	cn1546	pOP-EAP00079_EST_C_1_pBSK_SK	568
cl1338	ct1439	cn1547	pOP-CEO02250_EST_C_1_pSK_SK	611
cl1339	ct1440	cn1548	pOP-CEO02253_EST_C_1_pSK_SK	354
cl1339	ct1440	cn1548	pOP-EO05814_EST_C_1_pSK_SK	549
cl1340	ct1441	cn1549	pOP-CBP00233_EST_C_1_pBSK_SK	659
cl1340	ct1441	cn1549	pOP-CEO02256_EST_C_1_pSK_SK	417
cl1340	ct1442	cn1550	pOP-CNH02128_EST_C_1_pSK_SK	421
cl1340	ct1442	cn1550	pOP-EO06044_EST_C_1_pSK_SK	648
cl1340	ct1442	cn1550	pOP-EO07734_EST_C_1_pSK_SK	769
cl1341	ct1443	cn1551	pOP-CEO01656_EST_C_1_pSK_SK	491
cl1341	ct1443	cn1551	pOP-CEO01744_EST_C_1_pSK_SK	460
cl1341	ct1443	cn1551	pOP-CEO02268_EST_C_1_pSK_SK	539
cl1341	ct1443	cn1551	pOP-EAP00999_EST_C_1_pBSK_SK	156
cl1341	ct1444	cn1552	pOP-CNH00980_EST_C_1_pSK_SK	512
cl1341	ct1444	cn1552	pOP-CNH01658_EST_C_1_pSK_SK	709
cl1341	ct1444	cn1552	pOP-CNH03617_EST_C_1_pSK_SK	602
cl1341	ct1444	cn1552	pOP-CNI01160_EST_C_1_pSK_SK	456
cl1341	ct1444	cn1552	pOP-EO02294_EST_C_1_pSK_SK	426
cl1341	ct1444	cn1552	pOP-EO07030_EST_C_1_pSK_SK	660
cl1341	ct1444	cn1552	pOP-EO08221_EST_C_1_pSK_SK	422
cl1342	ct1445	cn1553	pOP-CEO02293_EST_C_1_pSK_SK	612
cl1342	ct1445	cn1553	pOP-CNIP04084_EST_C_1_pSK_SK	411
cl1342	ct1445	cn1553	pOP-EAP00609_EST_C_1_pBSK_SK	542
cl1342	ct1445	cn1553	pOP-EAP00872_EST_C_1_pBSK_SK	284
cl1343	ct1446	cn1554	pOP-CEO02301_EST_C_1_pSK_SK	261
cl1343	ct1446	cn1554	pOP-CNH00553_EST_C_1_pSK_SK	622
cl1343	ct1446	cn1554	pOP-CNH01337_EST_C_1_pSK_SK	632
cl1343	ct1446	cn1554	pOP-CNH02376_EST_C_1_pSK_SK	520
cl1343	ct1446	cn1554	pOP-EO02182_EST_C_1_pSK_SK	629
cl1343	ct1446	cn1554	pOP-EO04765_EST_C_1_pSK_SK	648

cl1343	ct1447	cn1555	pOP-CNH01259_EST_C_1_pSK_SK	661
cl1343	ct1447	cn1555	pOP-CNH02647_EST_C_1_pSK_SK	662
cl1343	ct1447	cn1555	pOP-CNH02910_EST_C_1_pSK_SK	589
cl1343	ct1447	cn1555	pOP-CNH03480_EST_C_1_pSK_SK	488
cl1343	ct1447	cn1555	pOP-CNH04456	829
cl1343	ct1447	cn1555	pOP-CNHP00369_EST_C_1_pSK_SK	622
cl1344	ct1448	cn1556	pOP-CAP00377_EST_C_1_pBSK_SK	655
cl1344	ct1448	cn1556	pOP-CBP00203_EST_C_1_pBSK_SK	610
cl1344	ct1448	cn1556	pOP-CEM00064_EST_C_1_pSK_SK	263
cl1344	ct1448	cn1556	pOP-CEM00175_EST_C_1_pSK_SK	327
cl1344	ct1448	cn1556	pOP-CEO02303_EST_C_1_pSK_SK	384
cl1344	ct1448	cn1556	pOP-CNH00632_EST_C_1_pSK_SK	683
cl1344	ct1448	cn1556	pOP-CNH01655_EST_C_1_pSK_SK	638
cl1344	ct1448	cn1556	pOP-CNHP00492_EST_C_1_pSK_SK	612
cl1344	ct1448	cn1556	pOP-CNIP00364_EST_C_1_pSK_SK	379
cl1344	ct1448	cn1556	pOP-CNIP00831_EST_C_1_pSK_SK	296
cl1344	ct1448	cn1556	pOP-EAP01917_EST_C_1_pBSK_SK	391
cl1345	ct1449	cn1557	pOP-CEO02316_EST_C_1_pSK_SK	428
cl1345	ct1449	cn1557	pOP-CNI01262_EST_C_1_pSK_SK	350
cl1346	ct1450	cn1558	pOP-CEO01382_EST_C_1_pSK_SK	659
cl1346	ct1450	cn1558	pOP-CEO02327_EST_C_1_pSK_SK	556
cl1346	ct1450	cn1558	pOP-CNI01406_EST_C_1_pSK_SK	168
cl1346	ct1450	cn1558	pOP-CNI02167_EST_C_1_pSK_SK	404
cl1346	ct1450	cn1558	pOP-CNI02199_EST_C_1_pSK_SK	376
cl1346	ct1450	cn1558	pOP-CNIP00617_EST_C_1_pSK_SK	526
cl1346	ct1450	cn1558	pOP-CNIP00819_EST_C_1_pSK_SK	157
cl1346	ct1450	cn1558	pOP-CNIP00913_EST_C_1_pSK_SK	157
cl1346	ct1450	cn1558	pOP-EAP01519_EST_C_1_pBSK_SK	594
cl1347	ct1451	cn1559	pOP-CEO02330_EST_C_1_pSK_SK	305
cl1347	ct1451	cn1559	pOP-CNI01249_EST_C_1_pSK_SK	491
cl1347	ct1451	cn1559	pOP-EAP01689_EST_C_1_pBSK_SK	468
cl1348	ct1452	cn1560	pOP-CEO02335_EST_C_1_pSK_SK	363
cl1348	ct1452	cn1560	pOP-EAP02910_EST_C_1_pBSK_SK	548
cl1349	ct1453	cn1561	pOP-CAP00394_EST_C_1_pBSK_SK	616
cl1349	ct1453	cn1561	pOP-CEO02378_EST_C_1_pSK_SK	440
cl1349	ct1453	cn1561	pOP-CNI02223_EST_C_1_pSK_SK	386
cl1349	ct1453	cn1561	pOP-EO05121_EST_C_1_pSK_SK	491
cl1349	ct1453	cn1561	pOP-EO05922_EST_C_1_pSK_SK	660
cl1349	ct1453	cn1562	pOP-CEO02340_EST_C_1_pSK_SK	567
cl1350	ct1454	cn1563	pOP-CEO02341_EST_C_1_pSK_SK	278
cl1350	ct1454	cn1563	pOP-EO07000_EST_C_1_pSK_SK	271
cl1351	ct1455	cn1564	pOP-CEO02351_EST_C_1_pSK_SK	473
cl1351	ct1455	cn1564	pOP-EAP00421_EST_C_1_pBSK_SK	215
cl1352	ct1456	cn1565	pOP-CEO02352_EST_C_1_pSK_SK	371
cl1352	ct1456	cn1565	pOP-CNIP00343_EST_C_1_pSK_SK	506
cl1353	ct1457	cn1566	pOP-CEO02355_EST_C_1_pSK_SK	329
cl1353	ct1457	cn1566	pOP-CNHP00417_EST_C_1_pSK_SK	735
cl1353	ct1457	cn1566	pOP-CNI02165_EST_C_1_pSK_SK	605
cl1353	ct1457	cn1566	pOP-EAP01210_EST_C_1_pBSK_SK	237
cl1354	ct1458	cn1567	pOP-CEO02356_EST_C_1_pSK_SK	536
cl1354	ct1458	cn1567	pOP-EAP00112_EST_C_1_pBSK_SK	569
cl1354	ct1458	cn1567	pOP-EAP00235_EST_C_1_pBSK_SK	435
cl1354	ct1458	cn1567	pOP-EAP02062_EST_C_1_pBSK_SK	129
cl1354	ct1458	cn1567	pOP-EAP02990_EST_C_1_pBSK_SK	451
cl1355	ct1459	cn1568	pOP-CEO02283_EST_C_1_pSK_SK	451
cl1355	ct1459	cn1568	pOP-CNH00613_EST_C_1_pSK_SK	667

cl1355	ct1459	cn1568	pOP-EO08398_EST_C_1_pSK_SK	431
cl1355	ct1459	cn1569	pOP-CNI01727_EST_C_1_pSK_SK	503
cl1355	ct1460	cn1570	pOP-CEO02363_EST_C_1_pSK_SK	351
cl1355	ct1460	cn1570	pOP-CNH04785_EST_C_1_pSK_SK	559
cl1355	ct1460	cn1570	pOP-CNI01811_EST_C_1_pSK_SK	504
cl1355	ct1460	cn1570	pOP-EO02626_EST_C_1_pSK_SK	372
cl1356	ct1461	cn1571	pOP-CEO00675_EST_C_1_pSK_SK	205
cl1356	ct1461	cn1571	pOP-CEO02372_EST_C_1_pSK_SK	233
cl1356	ct1461	cn1571	pOP-CNIP00412_EST_C_1_pSK_SK	443
cl1356	ct1461	cn1571	pOP-EAP01253_EST_C_1_pBSK_SK	176
cl1356	ct1461	cn1571	pOP-EAP02183_EST_C_1_pBSK_SK	296
cl1357	ct1462	cn1572	pOP-CEO02387_EST_C_1_pSK_SK	336
cl1357	ct1462	cn1572	pOP-CNI01963_EST_C_1_pSK_SK	202
cl1357	ct1462	cn1572	pOP-EAP02126_EST_C_1_pBSK_SK	302
cl1358	ct1463	cn1573	pOP-CEO02409_EST_C_1_pSK_SK	256
cl1358	ct1463	cn1573	pOP-CNIP00070_EST_C_1_pSK_SK	125
cl1358	ct1463	cn1573	pOP-CNIP00857_EST_C_1_pSK_SK	221
cl1358	ct1463	cn1573	pOP-CNIP00967_EST_C_1_pSK_SK	419
cl1359	ct1464	cn1574	pOP-CEO00567_EST_C_1_pSK_SK	320
cl1359	ct1464	cn1574	pOP-CEO02411_EST_C_1_pSK_SK	277
cl1360	ct1465	cn1575	pOP-CAP00040_EST_C_1_pBSK_SK	397
cl1360	ct1465	cn1575	pOP-CEO02414_EST_C_1_pSK_SK	188
cl1361	ct1466	cn1576	pOP-CEO02420_EST_C_1_pSK_SK	587
cl1361	ct1466	cn1576	pOP-CNH00988_EST_C_1_pSK_SK	523
cl1362	ct1467	cn1577	pOP-CEO02423_EST_C_1_pSK_SK	510
cl1362	ct1467	cn1577	pOP-CNI01667_EST_C_1_pSK_SK	542
cl1362	ct1467	cn1577	pOP-CNIP00483_EST_C_1_pSK_SK	492
cl1362	ct1467	cn1577	pOP-CNIP00513_EST_C_1_pSK_SK	348
cl1363	ct1468	cn1578	pOP-CEO02426_EST_C_1_pSK_SK	385
cl1363	ct1468	cn1578	pOP-CNH01526_EST_C_1_pSK_SK	347
cl1363	ct1468	cn1578	pOP-EO06962_EST_C_1_pSK_SK	452
cl1364	ct1469	cn1579	pOP-CEO02436_EST_C_1_pSK_SK	641
cl1364	ct1469	cn1579	pOP-EAP03407_EST_C_1_pBSK_SK	287
cl1364	ct1469	cn1580	pOP-CNI01176_EST_C_1_pSK_SK	599
cl1365	ct1470	cn1581	pOP-CEO00717_EST_C_1_pSK_SK	520
cl1365	ct1470	cn1581	pOP-CEO02454_EST_C_1_pSK_SK	221
cl1366	ct1471	cn1582	pOP-CEO02455_EST_C_1_pSK_SK	163
cl1366	ct1471	cn1582	pOP-CNI01655_EST_C_1_pSK_SK	516
cl1367	ct1472	cn1583	pOP-CEO02467_EST_C_1_pSK_SK	557
cl1367	ct1472	cn1583	pOP-CNIP00225_EST_C_1_pSK_SK	459
cl1368	ct1473	cn1584	pOP-CEO02473_EST_C_1_pSK_SK	365
cl1368	ct1473	cn1584	pOP-CNIP00148_EST_C_1_pSK_SK	391
cl1368	ct1473	cn1584	pOP-CNIP00422_EST_C_1_pSK_SK	368
cl1368	ct1473	cn1584	pOP-EO08112_EST_C_1_pSK_SK	377
cl1369	ct1474	cn1585	pOP-CEO01613_EST_C_1_pSK_SK	348
cl1369	ct1474	cn1585	pOP-CEO02077_EST_C_1_pSK_SK	364
cl1369	ct1474	cn1585	pOP-CEO02478_EST_C_1_pSK_SK	379
cl1369	ct1474	cn1585	pOP-EAP02913_EST_C_1_pBSK_SK	320
cl1369	ct1475	cn1586	pOP-CNH01032_EST_C_1_pSK_SK	226
cl1369	ct1475	cn1586	pOP-CNH03135_EST_C_1_pSK_SK	613
cl1369	ct1475	cn1586	pOP-CNH04356	563
cl1369	ct1475	cn1586	pOP-EAP01498_EST_C_1_pBSK_SK	627
cl1369	ct1475	cn1586	pOP-EAP03691_EST_C_1_pBSK_SK	156
cl1369	ct1475	cn1587	pOP-CEO02078_EST_C_1_pSK_SK	361
cl1370	ct1476	cn1588	pOP-CEO01187_EST_C_1_pSK_SK	105
cl1370	ct1476	cn1588	pOP-CEO02425_EST_C_1_pSK_SK	241

cl1370	ct1476	cn1588	pOP-CEO02490_EST_C_1_pSK_SK	416
cl1371	ct1477	cn1589	pOP-CEO02492_EST_C_1_pSK_SK	614
cl1371	ct1477	cn1589	pOP-EAP01093_EST_C_1_pBSK_SK	186
cl1372	ct1478	cn1590	pOP-CEO02493_EST_C_1_pSK_SK	289
cl1372	ct1478	cn1590	pOP-EAP05012_EST_C_1_pBSK_SK	541
cl1373	ct1479	cn1591	pOP-CEO02497_EST_C_1_pSK_SK	741
cl1373	ct1479	cn1591	pOP-CEO02498_EST_C_1_pSK_SK	687
cl1373	ct1479	cn1591	pOP-EAP00641_EST_C_1_pBSK_SK	592
cl1373	ct1479	cn1592	pOP-CEO01583_EST_C_1_pSK_SK	641
cl1374	ct1480	cn1593	pOP-CBP00190_EST_C_1_pBSK_SK	454
cl1374	ct1480	cn1593	pOP-CEO02511_EST_C_1_pSK_SK	236
cl1374	ct1480	cn1593	pOP-CNHP00163_EST_C_1_pSK_SK	463
cl1374	ct1480	cn1593	pOP-EAP02375_EST_C_1_pBSK_SK	480
cl1374	ct1480	cn1593	pOP-EAP02391_EST_C_1_pBSK_SK	485
cl1375	ct1481	cn1594	pOP-CEO02517_EST_C_1_pSK_SK	141
cl1375	ct1481	cn1594	pOP-CNI01617_EST_C_1_pSK_SK	302
cl1376	ct1482	cn1595	pOP-CEO02259_EST_C_1_pSK_SK	195
cl1376	ct1482	cn1595	pOP-CEO02524_EST_C_1_pSK_SK	289
cl1377	ct1483	cn1596	pOP-CEO02527_EST_C_1_pSK_SK	434
cl1377	ct1483	cn1596	pOP-EAP01217_EST_C_1_pBSK_SK	290
cl1378	ct1484	cn1597	pOP-CEO02538_EST_C_1_pSK_SK	186
cl1378	ct1484	cn1597	pOP-EO08289_EST_C_1_pSK_SK	420
cl1379	ct1485	cn1598	pOP-CEO01019_EST_C_1_pSK_SK	258
cl1379	ct1485	cn1598	pOP-CEO02543_EST_C_1_pSK_SK	341
cl1380	ct1486	cn1599	pOP-CEO02546_EST_C_1_pSK_SK	287
cl1380	ct1486	cn1599	pOP-CNH02125_EST_C_1_pSK_SK	434
cl1380	ct1486	cn1599	pOP-CNH03160_EST_C_1_pSK_SK	605
cl1380	ct1486	cn1599	pOP-CNH03413_EST_C_1_pSK_SK	487
cl1380	ct1486	cn1599	pOP-EN00828_EST_C_1_pSK_SK	519
cl1380	ct1486	cn1599	pOP-EN00832_EST_C_1_pSK_SK	520
cl1380	ct1486	cn1599	pOP-EO02927_EST_C_1_pSK_SK	425
cl1380	ct1486	cn1599	pOP-EO03567_EST_C_1_pSK_SK	544
cl1381	ct1487	cn1600	pOP-CEO02557_EST_C_1_pSK_SK	446
cl1381	ct1487	cn1600	pOP-CNH02091_EST_C_1_pSK_SK	477
cl1381	ct1487	cn1600	pOP-CNIP00872_EST_C_1_pSK_SK	315
cl1381	ct1487	cn1600	pOP-EAP02727_EST_C_1_pBSK_SK	542
cl1382	ct1488	cn1601	pOP-CEO02563_EST_C_1_pSK_SK	708
cl1382	ct1488	cn1601	pOP-CNH04892_EST_C_1_pSK_SK	743
cl1383	ct1489	cn1602	pOP-CEO02572_EST_C_1_pSK_SK	294
cl1383	ct1489	cn1602	pOP-CNI01343_EST_C_1_pSK_SK	319
cl1383	ct1489	cn1602	pOP-EAP03587_EST_C_1_pBSK_SK	184
cl1383	ct1490	cn1603	pOP-EAP01026_EST_C_1_pBSK_SK	167
cl1383	ct1490	cn1603	pOP-EAP01027_EST_C_1_pBSK_SK	225
cl1383	ct1490	cn1603	pOP-EAP01441_EST_C_1_pBSK_SK	220
cl1384	ct1491	cn1604	pOP-CEO00597_EST_C_1_pSK_SK	290
cl1384	ct1491	cn1604	pOP-CEO02579_EST_C_1_pSK_SK	335
cl1385	ct1492	cn1605	pOP-CEO03086_EST_C_1_pSK_SK	593
cl1385	ct1492	cn1605	pOP-EAP03248_EST_C_1_pBSK_SK	645
cl1385	ct1492	cn1605	pOP-EO02178_EST_C_1_pSK_SK	620
cl1385	ct1492	cn1605	pOP-EO02579_EST_C_1_pSK_SK	468
cl1385	ct1492	cn1605	pOP-EO03162_EST_C_1_pSK_SK	461
cl1385	ct1492	cn1606	pOP-CNIP00336_EST_C_1_pSK_SK	489
cl1385	ct1492	cn1606	pOP-EAP01123_EST_C_1_pBSK_SK	661
cl1385	ct1492	cn1607	pOP-CEO02582_EST_C_1_pSK_SK	335
cl1386	ct1493	cn1608	pOP-CEO02586_EST_C_1_pSK_SK	299
cl1386	ct1493	cn1608	pOP-CNH02159_EST_C_1_pSK_SK	528

cl1386	ct1493	cn1608	pOP-CNI01366_EST_C_1_pSK_SK	741
cl1386	ct1493	cn1608	pOP-CNIP00615_EST_C_1_pSK_SK	274
cl1386	ct1493	cn1608	pOP-EO02531_EST_C_1_pSK_SK	525
cl1386	ct1493	cn1608	pOP-EO04889_EST_C_1_pSK_SK	515
cl1387	ct1494	cn1609	pOP-CEO01703_EST_C_1_pSK_SK	293
cl1387	ct1494	cn1609	pOP-CEO02587_EST_C_1_pSK_SK	373
cl1388	ct1495	cn1610	pOP-CEO02386_EST_C_1_pSK_SK	235
cl1388	ct1495	cn1610	pOP-CEO02590_EST_C_1_pSK_SK	391
cl1389	ct1496	cn1611	pOP-CEO01339	204
cl1389	ct1496	cn1611	pOP-CEO02522_EST_C_1_pSK_SK	171
cl1389	ct1496	cn1611	pOP-CEO02594_EST_C_1_pSK_SK	362
cl1389	ct1496	cn1611	pOP-EAP01403_EST_C_1_pBSK_SK	266
cl1389	ct1496	cn1612	pOP-CNLP00018_EST_C_1_pSK_SK	354
cl1390	ct1497	cn1613	pOP-CEO02595_EST_C_1_pSK_SK	309
cl1390	ct1497	cn1613	pOP-EAP00717_EST_C_1_pBSK_SK	408
cl1391	ct1498	cn1614	pOP-CEO02597_EST_C_1_pSK_SK	241
cl1391	ct1498	cn1614	pOP-EAP02193_EST_C_1_pBSK_SK	444
cl1392	ct1499	cn1615	pOP-CEO02605_EST_C_1_pSK_SK	252
cl1392	ct1499	cn1615	pOP-EAP02256_EST_C_1_pBSK_SK	174
cl1393	ct1500	cn1616	pOP-CEO01306	224
cl1393	ct1500	cn1616	pOP-CEO02165_EST_C_1_pSK_SK	246
cl1393	ct1500	cn1616	pOP-CEO02307_EST_C_1_pSK_SK	417
cl1393	ct1500	cn1616	pOP-CEO02413_EST_C_1_pSK_SK	230
cl1393	ct1500	cn1616	pOP-CEO02795_EST_C_1_pSK_SK	132
cl1393	ct1500	cn1616	pOP-CNI01561_EST_C_1_pSK_SK	150
cl1393	ct1500	cn1616	pOP-EAP01117_EST_C_1_pBSK_SK	454
cl1393	ct1500	cn1616	pOP-EO02297_EST_C_1_pSK_SK	276
cl1393	ct1500	cn1616	pOP-EO07356_EST_C_1_pSK_SK	836
cl1393	ct1500	cn1616	pOP-EO07915_EST_C_1_pSK_SK	473
cl1393	ct1500	cn1616	pOP-EO08146_EST_C_1_pSK_SK	455
cl1393	ct1500	cn1617	pOP-CEO02606_EST_C_1_pSK_SK	490
cl1394	ct1501	cn1618	pOP-CEO01757_EST_C_1_pSK_SK	205
cl1394	ct1501	cn1618	pOP-CEO02615_EST_C_1_pSK_SK	183
cl1394	ct1501	cn1618	pOP-CNI01203_EST_C_1_pSK_SK	210
cl1394	ct1501	cn1618	pOP-CNI01331_EST_C_1_pSK_SK	278
cl1395	ct1502	cn1619	pOP-CEO02626_EST_C_1_pSK_SK	196
cl1395	ct1502	cn1619	pOP-CNI01161_EST_C_1_pSK_SK	419
cl1396	ct1503	cn1620	pOP-CEO02639_EST_C_1_pSK_SK	267
cl1396	ct1503	cn1620	pOP-EAP03169_EST_C_1_pBSK_SK	650
cl1397	ct1504	cn1621	pOP-CEM00074_EST_C_1_pSK_SK	173
cl1397	ct1504	cn1621	pOP-CEO02209_EST_C_1_pSK_SK	242
cl1397	ct1504	cn1621	pOP-CEO02656_EST_C_1_pSK_SK	375
cl1397	ct1504	cn1621	pOP-CNI01525_EST_C_1_pSK_SK	482
cl1398	ct1505	cn1622	pOP-CEO02657_EST_C_1_pSK_SK	433
cl1398	ct1505	cn1622	pOP-CEO02658_EST_C_1_pSK_SK	342
cl1398	ct1505	cn1622	pOP-EAP02225_EST_C_1_pBSK_SK	311
cl1398	ct1505	cn1622	pOP-EO07664_EST_C_1_pSK_SK	747
cl1399	ct1506	cn1623	pOP-CEO01109_EST_C_1_pSK_SK	468
cl1399	ct1506	cn1623	pOP-CEO02661_EST_C_1_pSK_SK	526
cl1400	ct1507	cn1624	pOP-CEO00709_EST_C_1_pSK_SK	675
cl1400	ct1507	cn1624	pOP-CEO02662_EST_C_1_pSK_SK	507
cl1401	ct1508	cn1625	pOP-CEO02671_EST_C_1_pSK_SK	216
cl1401	ct1508	cn1625	pOP-CNH00826_EST_C_1_pSK_SK	386
cl1401	ct1508	cn1625	pOP-EAP03766_EST_C_1_pBSK_SK	464
cl1402	ct1509	cn1626	pOP-CEO02707_EST_C_1_pSK_SK	278
cl1402	ct1509	cn1626	pOP-CNH00694_EST_C_1_pSK_SK	629

cl1402	ct1509	cn1626	pOP-CNHP00522_EST_C_1_pSK_SK	543
cl1403	ct1510	cn1627	pOP-CEO02708_EST_C_1_pSK_SK	373
cl1403	ct1510	cn1627	pOP-CNH00704_EST_C_1_pSK_SK	710
cl1404	ct1511	cn1628	pOP-CEO02710_EST_C_1_pSK_SK	448
cl1404	ct1511	cn1628	pOP-CEO02712_EST_C_1_pSK_SK	472
cl1405	ct1512	cn1629	pOP-CEO01510_EST_C_1_pSK_SK	223
cl1405	ct1512	cn1629	pOP-CEO02714_EST_C_1_pSK_SK	476
cl1406	ct1513	cn1630	pOP-CEO02717_EST_C_1_pSK_SK	622
cl1406	ct1513	cn1630	pOP-CNIP00204_EST_C_1_pSK_SK	464
cl1407	ct1514	cn1631	pOP-CEO02720_EST_C_1_pSK_SK	667
cl1407	ct1514	cn1631	pOP-EAP02240_EST_C_1_pBSK_SK	163
cl1407	ct1514	cn1631	pOP-EAP03423_EST_C_1_pBSK_SK	265
cl1408	ct1515	cn1632	pOP-CEO02738_EST_C_1_pSK_SK	485
cl1408	ct1515	cn1632	pOP-EAP03344_EST_C_1_pBSK_SK	620
cl1409	ct1516	cn1633	pOP-CEO02739_EST_C_1_pSK_SK	514
cl1409	ct1516	cn1633	pOP-CNH04143	378
cl1410	ct1517	cn1634	pOP-CEO02743_EST_C_1_pSK_SK	277
cl1410	ct1517	cn1634	pOP-EO04854_EST_C_1_pSK_SK	357
cl1411	ct1518	cn1635	pOP-CEO02745_EST_C_1_pSK_SK	522
cl1411	ct1518	cn1635	pOP-EAP03379_EST_C_1_pBSK_SK	608
cl1412	ct1519	cn1636	pOP-CEO02747_EST_C_1_pSK_SK	502
cl1412	ct1519	cn1636	pOP-CNHP00105_EST_C_1_pSK_SK	482
cl1413	ct1520	cn1637	pOP-CEO02755_EST_C_1_pSK_SK	353
cl1413	ct1520	cn1637	pOP-EAP01218_EST_C_1_pBSK_SK	612
cl1413	ct1520	cn1638	pOP-CEO03523_EST_C_1_pSK_SK	620
cl1414	ct1521	cn1639	pOP-CEO02760_EST_C_1_pSK_SK	559
cl1414	ct1521	cn1639	pOP-CNH03616_EST_C_1_pSK_SK	583
cl1414	ct1521	cn1639	pOP-CNH04602	796
cl1414	ct1521	cn1639	pOP-CNH04755_EST_C_1_pSK_SK	596
cl1414	ct1521	cn1639	pOP-CNI01513_EST_C_1_pSK_SK	558
cl1414	ct1521	cn1639	pOP-CNI02110_EST_C_1_pSK_SK	552
cl1414	ct1521	cn1639	pOP-CNI02240_EST_C_1_pSK_SK	714
cl1415	ct1522	cn1640	pOP-CEO02766_EST_C_1_pSK_SK	518
cl1415	ct1522	cn1640	pOP-EAP00661_EST_C_1_pBSK_SK	510
cl1415	ct1522	cn1640	pOP-EAP00988_EST_C_1_pBSK_SK	177
cl1416	ct1523	cn1641	pOP-CAP00380_EST_C_1_pBSK_SK	639
cl1416	ct1523	cn1641	pOP-CEO02772_EST_C_1_pSK_SK	612
cl1416	ct1523	cn1641	pOP-CNH04998_EST_C_1_pSK_SK	211
cl1416	ct1523	cn1641	pOP-EO02498_EST_C_1_pSK_SK	523
cl1416	ct1523	cn1641	pOP-EO08337_EST_C_1_pSK_SK	297
cl1416	ct1523	cn1642	pOP-CAP00258_EST_C_1_pBSK_SK	577
cl1417	ct1524	cn1643	pOP-CEO02777_EST_C_1_pSK_SK	397
cl1417	ct1524	cn1643	pOP-CEO02778_EST_C_1_pSK_SK	379
cl1418	ct1525	cn1644	pOP-CEO02779_EST_C_1_pSK_SK	220
cl1418	ct1525	cn1644	pOP-CNHP00118_EST_C_1_pSK_SK	574
cl1419	ct1526	cn1645	pOP-CEO02782_EST_C_1_pSK_SK	412
cl1419	ct1526	cn1645	pOP-CNHP00063_EST_C_1_pSK_SK	594
cl1419	ct1526	cn1645	pOP-CNIP00787_EST_C_1_pSK_SK	633
cl1420	ct1527	cn1646	pOP-CEO02791_EST_C_1_pSK_SK	516
cl1420	ct1527	cn1646	pOP-CNH00751_EST_C_1_pSK_SK	509
cl1420	ct1527	cn1646	pOP-EAP00587_EST_C_1_pBSK_SK	538
cl1420	ct1527	cn1646	pOP-EAP01364_EST_C_1_pBSK_SK	315
cl1420	ct1527	cn1646	pOP-EAP01365_EST_C_1_pBSK_SK	336
cl1420	ct1527	cn1646	pOP-EAP01867_EST_C_1_pBSK_SK	690
cl1421	ct1528	cn1647	pOP-CEO01226_EST_C_1_pSK_SK	668
cl1421	ct1528	cn1647	pOP-CEO02796_EST_C_1_pSK_SK	361

cl1421	ct1528	cn1647	pOP-CNH01297_EST_C_1_pSK_SK	606
cl1421	ct1528	cn1647	pOP-CNH01513_EST_C_1_pSK_SK	498
cl1422	ct1529	cn1648	pOP-CEO02797_EST_C_1_pSK_SK	384
cl1422	ct1529	cn1648	pOP-EAP03409_EST_C_1_pBSK_SK	583
cl1423	ct1530	cn1649	pOP-CEO02799_EST_C_1_pSK_SK	399
cl1423	ct1530	cn1649	pOP-EAP03761_EST_C_1_pBSK_SK	412
cl1424	ct1531	cn1650	pOP-CEO01608_EST_C_1_pSK_SK	605
cl1424	ct1531	cn1650	pOP-CEO02803_EST_C_1_pSK_SK	563
cl1425	ct1532	cn1651	pOP-CEO02806_EST_C_1_pSK_SK	345
cl1425	ct1532	cn1651	pOP-CNH03035_EST_C_1_pSK_SK	377
cl1425	ct1532	cn1651	pOP-CNH04475	651
cl1425	ct1532	cn1651	pOP-EAP02288_EST_C_1_pBSK_SK	660
cl1426	ct1533	cn1652	pOP-CEO00828_EST_C_1_pSK_SK	256
cl1426	ct1533	cn1652	pOP-CEO02807_EST_C_1_pSK_SK	256
cl1427	ct1534	cn1653	pOP-CEO01045_EST_C_1_pSK_SK	467
cl1427	ct1534	cn1653	pOP-CEO02810_EST_C_1_pSK_SK	493
cl1427	ct1534	cn1653	pOP-EAP03578_EST_C_1_pBSK_SK	576
cl1428	ct1535	cn1654	pOP-CEO02812_EST_C_1_pSK_SK	396
cl1428	ct1535	cn1654	pOP-EAP01710_EST_C_1_pBSK_SK	640
cl1429	ct1536	cn1655	pOP-CEO02821_EST_C_1_pSK_SK	401
cl1429	ct1536	cn1655	pOP-CNI01991_EST_C_1_pSK_SK	342
cl1430	ct1537	cn1656	pOP-CEO02841_EST_C_1_pSK_SK	275
cl1430	ct1537	cn1656	pOP-CNH01208_EST_C_1_pSK_SK	369
cl1431	ct1538	cn1657	pOP-CEO02847_EST_C_1_pSK_SK	364
cl1431	ct1538	cn1657	pOP-CNH04621	693
cl1431	ct1538	cn1657	pOP-CNHP00162_EST_C_1_pSK_SK	497
cl1431	ct1538	cn1657	pOP-CNHP00532_EST_C_1_pSK_SK	565
cl1432	ct1539	cn1658	pOP-CBP00165_EST_C_1_pBSK_SK	306
cl1432	ct1539	cn1658	pOP-CEO02752_EST_C_1_pSK_SK	446
cl1432	ct1539	cn1658	pOP-CEO02848_EST_C_1_pSK_SK	456
cl1432	ct1539	cn1658	pOP-EAP01030_EST_C_1_pBSK_SK	196
cl1432	ct1539	cn1658	pOP-EAP01444_EST_C_1_pBSK_SK	183
cl1432	ct1539	cn1658	pOP-EO05836_EST_C_1_pSK_SK	149
cl1433	ct1540	cn1659	pOP-CEO02849_EST_C_1_pSK_SK	428
cl1433	ct1540	cn1659	pOP-EO07099_EST_C_1_pSK_SK	511
cl1434	ct1541	cn1660	pOP-CEO01725_EST_C_1_pSK_SK	312
cl1434	ct1541	cn1660	pOP-CEO02857_EST_C_1_pSK_SK	227
cl1435	ct1542	cn1661	pOP-CEO02858_EST_C_1_pSK_SK	258
cl1435	ct1542	cn1661	pOP-CNI01988_EST_C_1_pSK_SK	212
cl1435	ct1542	cn1661	pOP-EAP00427_EST_C_1_pBSK_SK	169
cl1435	ct1542	cn1661	pOP-EAP01238_EST_C_1_pBSK_SK	253
cl1435	ct1542	cn1661	pOP-EAP03466_EST_C_1_pBSK_SK	265
cl1436	ct1543	cn1662	pOP-CEO02863_EST_C_1_pSK_SK	486
cl1436	ct1543	cn1662	pOP-EAP02070_EST_C_1_pBSK_SK	259
cl1437	ct1544	cn1663	pOP-EAP01793_EST_C_1_pBSK_SK	495
cl1437	ct1545	cn1664	pOP-CEO02871_EST_C_1_pSK_SK	405
cl1437	ct1545	cn1664	pOP-CNH04775_EST_C_1_pSK_SK	606
cl1438	ct1546	cn1665	pOP-CEM00204_EST_C_1_pSK_SK	335
cl1438	ct1546	cn1665	pOP-CEO02872_EST_C_1_pSK_SK	263
cl1439	ct1547	cn1666	pOP-CEO02878_EST_C_1_pSK_SK	183
cl1439	ct1547	cn1666	pOP-CEO03750_EST_C_1_pSK_SK	593
cl1440	ct1548	cn1667	pOP-CEO02882_EST_C_1_pSK_SK	416
cl1440	ct1548	cn1667	pOP-CNH01183_EST_C_1_pSK_SK	589
cl1440	ct1549	cn1668	pOP-CNIP00546_EST_C_1_pSK_SK	213
cl1440	ct1549	cn1668	pOP-CNIP04027_EST_C_1_pSK_SK	606
cl1440	ct1549	cn1668	pOP-EAP02709_EST_C_1_pBSK_SK	686

cl1441	ct1550	cn1669	pOP-CEO02887_EST_C_1_pSK_SK	239
cl1441	ct1550	cn1669	pOP-EAP00866_EST_C_1_pBSK_SK	530
cl1442	ct1551	cn1670	pOP-CEO02759_EST_C_1_pSK_SK	354
cl1442	ct1551	cn1670	pOP-CEO02889_EST_C_1_pSK_SK	368
cl1442	ct1551	cn1670	pOP-CNI02043_EST_C_1_pSK_SK	246
cl1442	ct1551	cn1670	pOP-EAP03806_EST_C_1_pBSK_SK	460
cl1443	ct1552	cn1671	pOP-CEO00638_EST_C_1_pSK_SK	110
cl1443	ct1552	cn1671	pOP-CEO00639_EST_C_1_pSK_SK	110
cl1443	ct1552	cn1671	pOP-CEO01388_EST_C_1_pSK_SK	216
cl1443	ct1552	cn1671	pOP-CEO01497_EST_C_1_pSK_SK	211
cl1443	ct1552	cn1671	pOP-CEO02724_EST_C_1_pSK_SK	471
cl1443	ct1552	cn1671	pOP-CEO02925_EST_C_1_pSK_SK	170
cl1443	ct1552	cn1671	pOP-CEO03007_EST_C_1_pSK_SK	337
cl1443	ct1552	cn1671	pOP-CNLP00025_EST_C_1_pSK_SK	474
cl1443	ct1552	cn1671	pOP-EO03087_EST_C_1_pSK_SK	320
cl1443	ct1552	cn1671	pOP-EO03471_EST_C_1_pSK_SK	474
cl1443	ct1552	cn1671	pOP-EO06253_EST_C_1_pSK_SK	514
cl1443	ct1552	cn1671	pOP-EO08000_EST_C_1_pSK_SK	490
cl1443	ct1552	cn1672	pOP-CEM00216_EST_C_1_pSK_SK	375
cl1443	ct1553	cn1673	pOP-CEO02893_EST_C_1_pSK_SK	162
cl1443	ct1553	cn1673	pOP-CEO03563_EST_C_1_pSK_SK	387
cl1443	ct1553	cn1673	pOP-EAP00665_EST_C_1_pBSK_SK	418
cl1443	ct1553	cn1673	pOP-EAP01062_EST_C_1_pBSK_SK	171
cl1443	ct1553	cn1673	pOP-EAP01185_EST_C_1_pBSK_SK	213
cl1443	ct1553	cn1673	pOP-EAP01322_EST_C_1_pBSK_SK	494
cl1443	ct1553	cn1673	pOP-EAP01525_EST_C_1_pBSK_SK	336
cl1443	ct1553	cn1673	pOP-EAP01555_EST_C_1_pBSK_SK	380
cl1443	ct1553	cn1673	pOP-EAP01936_EST_C_1_pBSK_SK	298
cl1443	ct1553	cn1673	pOP-EAP02047_EST_C_1_pBSK_SK	152
cl1443	ct1553	cn1673	pOP-EAP02100_EST_C_1_pBSK_SK	209
cl1443	ct1553	cn1673	pOP-EAP02155_EST_C_1_pBSK_SK	174
cl1443	ct1553	cn1673	pOP-EAP02157_EST_C_1_pBSK_SK	228
cl1443	ct1553	cn1673	pOP-EAP03372_EST_C_1_pBSK_SK	471
cl1443	ct1553	cn1673	pOP-EAP03565_EST_C_1_pBSK_SK	324
cl1443	ct1553	cn1673	pOP-EAP03582_EST_C_1_pBSK_SK	223
cl1443	ct1553	cn1673	pOP-EAP03756_EST_C_1_pBSK_SK	334
cl1443	ct1553	cn1673	pOP-EO02174_EST_C_1_pSK_SK	338
cl1443	ct1553	cn1673	pOP-EO03769_EST_C_1_pSK_SK	435
cl1443	ct1553	cn1673	pOP-EO03926_EST_C_1_pSK_SK	517
cl1443	ct1553	cn1673	pOP-EO07351_EST_C_1_pSK_SK	343
cl1443	ct1553	cn1673	pOP-EO08325_EST_C_1_pSK_SK	472
cl1444	ct1554	cn1674	pOP-CEO00565_EST_C_1_pSK_SK	490
cl1444	ct1554	cn1674	pOP-CEO02895_EST_C_1_pSK_SK	474
cl1445	ct1555	cn1675	pOP-CEO02896_EST_C_1_pSK_SK	641
cl1445	ct1556	cn1676	pOP-CEO01004_EST_C_1_pSK_SK	340
cl1445	ct1556	cn1676	pOP-EAP01849_EST_C_1_pBSK_SK	400
cl1445	ct1556	cn1676	pOP-EAP02922_EST_C_1_pBSK_SK	699
cl1446	ct1557	cn1677	pOP-CEO01794_EST_C_1_pSK_SK	359
cl1446	ct1557	cn1677	pOP-CEO02902_EST_C_1_pSK_SK	458
cl1447	ct1558	cn1678	pOP-CEO02904_EST_C_1_pSK_SK	318
cl1447	ct1558	cn1678	pOP-CNH00563_EST_C_1_pSK_SK	734
cl1447	ct1558	cn1678	pOP-EAP02866_EST_C_1_pBSK_SK	347
cl1448	ct1559	cn1679	pOP-CEO02907_EST_C_1_pSK_SK	309
cl1448	ct1559	cn1679	pOP-EAP01255_EST_C_1_pBSK_SK	370
cl1448	ct1560	cn1680	pOP-EAP00658_EST_C_1_pBSK_SK	640
cl1448	ct1560	cn1680	pOP-EAP01468_EST_C_1_pBSK_SK	541

cl1448	ct1560	cn1680	pOP-EAP01638_EST_C_1_pBSK_SK	482
cl1449	ct1561	cn1681	pOP-CEO01839_EST_C_1_pSK_SK	208
cl1449	ct1561	cn1681	pOP-CEO02908_EST_C_1_pSK_SK	170
cl1450	ct1562	cn1682	pOP-CEO01810_EST_C_1_pSK_SK	356
cl1450	ct1562	cn1682	pOP-CNH02645_EST_C_1_pSK_SK	726
cl1450	ct1562	cn1683	pOP-CEO02913_EST_C_1_pSK_SK	648
cl1451	ct1563	cn1684	pOP-CEM00221_EST_C_1_pSK_SK	296
cl1451	ct1563	cn1684	pOP-CEO02914_EST_C_1_pSK_SK	279
cl1451	ct1563	cn1684	pOP-CNH00866_EST_C_1_pSK_SK	628
cl1451	ct1563	cn1684	pOP-EO02668_EST_C_1_pSK_SK	412
cl1452	ct1564	cn1685	pOP-CEO02688_EST_C_1_pSK_SK	476
cl1452	ct1564	cn1685	pOP-CNHP00450_EST_C_1_pSK_SK	712
cl1452	ct1565	cn1686	pOP-CEO02915_EST_C_1_pSK_SK	336
cl1452	ct1565	cn1686	pOP-CNH02508_EST_C_1_pSK_SK	368
cl1452	ct1565	cn1686	pOP-CNH04783_EST_C_1_pSK_SK	562
cl1452	ct1565	cn1686	pOP-EO07986_EST_C_1_pSK_SK	371
cl1453	ct1566	cn1687	pOP-CEO02918_EST_C_1_pSK_SK	734
cl1453	ct1566	cn1687	pOP-CNH04984_EST_C_1_pSK_SK	756
cl1453	ct1566	cn1687	pOP-CNI02019_EST_C_1_pSK_SK	538
cl1454	ct1567	cn1688	pOP-CEO02920_EST_C_1_pSK_SK	416
cl1454	ct1567	cn1688	pOP-CNH03565_EST_C_1_pSK_SK	313
cl1454	ct1567	cn1688	pOP-EN00314_EST_C_1_pSK_SK	548
cl1454	ct1568	cn1689	pOP-CNH03549_EST_C_1_pSK_SK	550
cl1454	ct1568	cn1689	pOP-CNH05002_EST_C_1_pSK_SK	440
cl1454	ct1568	cn1689	pOP-CNHP00041_EST_C_1_pSK_SK	619
cl1454	ct1568	cn1689	pOP-CNHP00172_EST_C_1_pSK_SK	439
cl1454	ct1568	cn1689	pOP-EAP00629_EST_C_1_pBSK_SK	553
cl1455	ct1569	cn1690	pOP-CAP00415_EST_C_1_pBSK_SK	519
cl1455	ct1569	cn1690	pOP-CEO02926_EST_C_1_pSK_SK	252
cl1456	ct1570	cn1691	pOP-CEO01391_EST_C_1_pSK_SK	105
cl1456	ct1570	cn1691	pOP-CEO02928_EST_C_1_pSK_SK	511
cl1456	ct1570	cn1691	pOP-CNH02063_EST_C_1_pSK_SK	470
cl1456	ct1570	cn1691	pOP-CNH02278_EST_C_1_pSK_SK	574
cl1457	ct1571	cn1692	pOP-CEO02350_EST_C_1_pSK_SK	324
cl1457	ct1571	cn1692	pOP-CEO02930_EST_C_1_pSK_SK	347
cl1458	ct1572	cn1693	pOP-CEO02935_EST_C_1_pSK_SK	264
cl1458	ct1572	cn1693	pOP-EAP03440_EST_C_1_pBSK_SK	229
cl1459	ct1573	cn1694	pOP-CEO00518_EST_C_1_pSK_SK	337
cl1459	ct1573	cn1694	pOP-CEO02937_EST_C_1_pSK_SK	554
cl1460	ct1574	cn1695	pOP-CEM00235_EST_C_1_pSK_SK	411
cl1460	ct1574	cn1695	pOP-CEO02946_EST_C_1_pSK_SK	570
cl1461	ct1575	cn1696	pOP-CEO02769_EST_C_1_pSK_SK	591
cl1461	ct1575	cn1696	pOP-CEO02951_EST_C_1_pSK_SK	591
cl1462	ct1576	cn1697	pOP-CEO02965_EST_C_1_pSK_SK	394
cl1462	ct1576	cn1697	pOP-EAP03357_EST_C_1_pBSK_SK	659
cl1463	ct1577	cn1698	pOP-CEO02968_EST_C_1_pSK_SK	257
cl1463	ct1577	cn1698	pOP-CNIP00221_EST_C_1_pSK_SK	308
cl1464	ct1578	cn1699	pOP-CEO02973_EST_C_1_pSK_SK	274
cl1464	ct1578	cn1699	pOP-CNI01474_EST_C_1_pSK_SK	274
cl1464	ct1578	cn1699	pOP-CNIP00300_EST_C_1_pSK_SK	275
cl1465	ct1579	cn1700	pOP-CEO00918_EST_C_1_pSK_SK	143
cl1465	ct1579	cn1700	pOP-CEO02977_EST_C_1_pSK_SK	510
cl1465	ct1579	cn1700	pOP-EAP00885_EST_C_1_pBSK_SK	278
cl1465	ct1579	cn1700	pOP-EAP02138_EST_C_1_pBSK_SK	157
cl1466	ct1580	cn1701	pOP-CEO00959_EST_C_1_pSK_SK	334
cl1466	ct1580	cn1701	pOP-CEO02980_EST_C_1_pSK_SK	539

cl1467	ct1581	cn1702	pOP-CEO01644_EST_C_1_pSK_SK	333
cl1467	ct1581	cn1702	pOP-CEO02982_EST_C_1_pSK_SK	179
cl1468	ct1582	cn1703	pOP-CEO02992_EST_C_1_pSK_SK	383
cl1468	ct1582	cn1703	pOP-CEO02993_EST_C_1_pSK_SK	385
cl1468	ct1582	cn1703	pOP-CEO02994_EST_C_1_pSK_SK	383
cl1469	ct1583	cn1704	pOP-CBP00127_EST_C_1_pBSK_SK	204
cl1469	ct1583	cn1704	pOP-CEO02997_EST_C_1_pSK_SK	282
cl1469	ct1583	cn1704	pOP-CNI01788_EST_C_1_pSK_SK	462
cl1470	ct1584	cn1705	pOP-CEO03000_EST_C_1_pSK_SK	205
cl1470	ct1584	cn1705	pOP-EAP00892_EST_C_1_pBSK_SK	198
cl1470	ct1584	cn1705	pOP-EAP01017_EST_C_1_pBSK_SK	193
cl1470	ct1584	cn1705	pOP-EAP01533_EST_C_1_pBSK_SK	550
cl1470	ct1584	cn1705	pOP-EAP02097_EST_C_1_pBSK_SK	140
cl1471	ct1585	cn1706	pOP-CEO02353_EST_C_1_pSK_SK	646
cl1471	ct1585	cn1706	pOP-CEO03017_EST_C_1_pSK_SK	281
cl1471	ct1585	cn1706	pOP-CNH04359	758
cl1471	ct1585	cn1706	pOP-CNH04512	757
cl1471	ct1585	cn1706	pOP-EAP03812_EST_C_1_pBSK_SK	604
cl1472	ct1586	cn1707	pOP-CEO02443_EST_C_1_pSK_SK	382
cl1472	ct1586	cn1707	pOP-CEO02474_EST_C_1_pSK_SK	263
cl1472	ct1586	cn1707	pOP-CNHP00285_EST_C_1_pSK_SK	636
cl1472	ct1586	cn1707	pOP-CNI01787_EST_C_1_pSK_SK	365
cl1472	ct1586	cn1707	pOP-EAP00899_EST_C_1_pBSK_SK	476
cl1472	ct1587	cn1708	pOP-CEO00619_EST_C_1_pSK_SK	312
cl1472	ct1587	cn1708	pOP-CEO00966_EST_C_1_pSK_SK	404
cl1472	ct1587	cn1708	pOP-CEO03020_EST_C_1_pSK_SK	117
cl1472	ct1587	cn1708	pOP-CNHP00230_EST_C_1_pSK_SK	410
cl1472	ct1587	cn1708	pOP-CNI01966_EST_C_1_pSK_SK	387
cl1472	ct1587	cn1708	pOP-EO02286_EST_C_1_pSK_SK	509
cl1473	ct1588	cn1709	pOP-CEO03021_EST_C_1_pSK_SK	715
cl1473	ct1588	cn1709	pOP-EAP03829_EST_C_1_pBSK_SK	582
cl1474	ct1589	cn1710	pOP-CEO03022_EST_C_1_pSK_SK	349
cl1474	ct1589	cn1710	pOP-CNIP00585_EST_C_1_pSK_SK	273
cl1475	ct1590	cn1711	pOP-CNI01449_EST_C_1_pSK_SK	231
cl1475	ct1590	cn1711	pOP-EO06066_EST_C_1_pSK_SK	607
cl1475	ct1591	cn1712	pOP-CEO02749_EST_C_1_pSK_SK	450
cl1475	ct1591	cn1712	pOP-CEO03106_EST_C_1_pSK_SK	314
cl1475	ct1591	cn1712	pOP-CNH01330_EST_C_1_pSK_SK	651
cl1475	ct1591	cn1712	pOP-CNH02161_EST_C_1_pSK_SK	581
cl1475	ct1591	cn1712	pOP-CNH02449_EST_C_1_pSK_SK	525
cl1475	ct1591	cn1712	pOP-CNH04265	545
cl1475	ct1591	cn1712	pOP-CNI01905_EST_C_1_pSK_SK	411
cl1475	ct1591	cn1712	pOP-CNI02040_EST_C_1_pSK_SK	487
cl1475	ct1591	cn1712	pOP-EAP01462_EST_C_1_pBSK_SK	660
cl1475	ct1591	cn1712	pOP-EAP02112_EST_C_1_pBSK_SK	352
cl1475	ct1591	cn1712	pOP-EAP03136_EST_C_1_pBSK_SK	622
cl1475	ct1591	cn1712	pOP-EO03172_EST_C_1_pSK_SK	473
cl1475	ct1591	cn1712	pOP-EO03871_EST_C_1_pSK_SK	513
cl1475	ct1591	cn1712	pOP-EO07061_EST_C_1_pSK_SK	553
cl1475	ct1591	cn1713	pOP-CEO03025_EST_C_1_pSK_SK	638
cl1476	ct1592	cn1714	pOP-CEO01962_EST_C_1_pSK_SK	347
cl1476	ct1592	cn1714	pOP-CEO03029_EST_C_1_pSK_SK	495
cl1476	ct1592	cn1714	pOP-CNH02889_EST_C_1_pSK_SK	571
cl1476	ct1592	cn1714	pOP-CNH04236	509
cl1476	ct1592	cn1714	pOP-EAP00625_EST_C_1_pBSK_SK	489
cl1476	ct1592	cn1714	pOP-EAP00626_EST_C_1_pBSK_SK	507

cl1476	ct1592	cn1714	pOP-EO05033_EST_C_1_pSK_SK	538
cl1477	ct1593	cn1715	pOP-CEO03034_EST_C_1_pSK_SK	467
cl1477	ct1593	cn1715	pOP-CNH03761_EST_C_1_pSK_SK	425
cl1478	ct1594	cn1716	pOP-CEO03040_EST_C_1_pSK_SK	552
cl1478	ct1594	cn1716	pOP-CNH01369_EST_C_1_pSK_SK	636
cl1478	ct1594	cn1716	pOP-EAP02054_EST_C_1_pBSK_SK	318
cl1478	ct1594	cn1716	pOP-EAP02055_EST_C_1_pBSK_SK	325
cl1478	ct1594	cn1717	pOP-EAP02111_EST_C_1_pBSK_SK	502
cl1479	ct1595	cn1718	pOP-CBP00008_EST_C_1_pBSK_SK	417
cl1479	ct1595	cn1718	pOP-CEO02313_EST_C_1_pSK_SK	323
cl1479	ct1595	cn1718	pOP-CEO03041_EST_C_1_pSK_SK	312
cl1479	ct1595	cn1718	pOP-CNH04599	154
cl1479	ct1595	cn1718	pOP-CNI01371_EST_C_1_pSK_SK	515
cl1479	ct1595	cn1718	pOP-CNI01554_EST_C_1_pSK_SK	367
cl1479	ct1595	cn1718	pOP-CNI01680_EST_C_1_pSK_SK	353
cl1480	ct1596	cn1719	pOP-CNI01185_EST_C_1_pSK_SK	300
cl1480	ct1596	cn1720	pOP-CEO03048_EST_C_1_pSK_SK	238
cl1480	ct1597	cn1721	pOP-CEM00073_EST_C_1_pSK_SK	314
cl1480	ct1597	cn1721	pOP-CEO02348_EST_C_1_pSK_SK	320
cl1480	ct1597	cn1721	pOP-CNH01177_EST_C_1_pSK_SK	582
cl1480	ct1597	cn1721	pOP-CNH03382_EST_C_1_pSK_SK	524
cl1480	ct1597	cn1721	pOP-EAP03310_EST_C_1_pBSK_SK	416
cl1480	ct1597	cn1721	pOP-EO06614_EST_C_1_pSK_SK	731
cl1480	ct1597	cn1722	pOP-CNH02479_EST_C_1_pSK_SK	530
cl1481	ct1598	cn1723	pOP-CEO03049_EST_C_1_pSK_SK	266
cl1481	ct1598	cn1723	pOP-CNIP00302_EST_C_1_pSK_SK	410
cl1482	ct1599	cn1724	pOP-CEO00708_EST_C_1_pSK_SK	529
cl1482	ct1599	cn1724	pOP-CEO03053_EST_C_1_pSK_SK	402
cl1483	ct1600	cn1725	pOP-CEO03064_EST_C_1_pSK_SK	347
cl1483	ct1600	cn1725	pOP-CNIP00980_EST_C_1_pSK_SK	204
cl1484	ct1601	cn1726	pOP-CEO03073_EST_C_1_pSK_SK	295
cl1484	ct1601	cn1726	pOP-CNI01936_EST_C_1_pSK_SK	354
cl1484	ct1601	cn1726	pOP-CNIP00557_EST_C_1_pSK_SK	351
cl1484	ct1601	cn1726	pOP-CNIP00596_EST_C_1_pSK_SK	507
cl1485	ct1602	cn1727	pOP-CBP00029_EST_C_1_pBSK_SK	410
cl1485	ct1602	cn1727	pOP-CBP00089_EST_C_1_pBSK_SK	437
cl1485	ct1602	cn1727	pOP-CEO03081_EST_C_1_pSK_SK	178
cl1485	ct1602	cn1727	pOP-CNI01150_EST_C_1_pSK_SK	524
cl1485	ct1602	cn1727	pOP-CNI01608_EST_C_1_pSK_SK	284
cl1485	ct1602	cn1727	pOP-CNIP00284_EST_C_1_pSK_SK	184
cl1485	ct1602	cn1727	pOP-CNIP00410_EST_C_1_pSK_SK	290
cl1486	ct1603	cn1728	pOP-CEO03060_EST_C_1_pSK_SK	392
cl1486	ct1603	cn1728	pOP-CEO03085_EST_C_1_pSK_SK	395
cl1487	ct1604	cn1729	pOP-CEO03091_EST_C_1_pSK_SK	385
cl1487	ct1604	cn1729	pOP-CNH01200_EST_C_1_pSK_SK	597
cl1487	ct1604	cn1729	pOP-CNH01367_EST_C_1_pSK_SK	760
cl1487	ct1604	cn1729	pOP-CNH01854_EST_C_1_pSK_SK	431
cl1487	ct1604	cn1729	pOP-CNH03398_EST_C_1_pSK_SK	584
cl1487	ct1604	cn1729	pOP-CNHP00294_EST_C_1_pSK_SK	344
cl1487	ct1604	cn1729	pOP-EO07142_EST_C_1_pSK_SK	459
cl1488	ct1605	cn1730	pOP-CEO02150_EST_C_1_pSK_SK	365
cl1488	ct1605	cn1730	pOP-CEO03032_EST_C_1_pSK_SK	185
cl1488	ct1605	cn1730	pOP-CEO03093_EST_C_1_pSK_SK	181
cl1488	ct1605	cn1730	pOP-EAP00757_EST_C_1_pBSK_SK	449
cl1488	ct1605	cn1730	pOP-EAP00758_EST_C_1_pBSK_SK	105
cl1488	ct1605	cn1730	pOP-EAP00787_EST_C_1_pBSK_SK	476

cl1488	ct1605	cn1730	pOP-EAP01270_EST_C_1_pBSK_SK	452
cl1488	ct1605	cn1730	pOP-EAP01330_EST_C_1_pBSK_SK	274
cl1488	ct1605	cn1730	pOP-EAP03383_EST_C_1_pBSK_SK	162
cl1489	ct1606	cn1731	pOP-CEO03107_EST_C_1_pSK_SK	311
cl1489	ct1606	cn1731	pOP-CEO03633_EST_C_1_pSK_SK	153
cl1490	ct1607	cn1732	pOP-CEO00736_EST_C_1_pSK_SK	356
cl1490	ct1607	cn1732	pOP-CEO03108_EST_C_1_pSK_SK	351
cl1491	ct1608	cn1733	pOP-CEO03119_EST_C_1_pSK_SK	349
cl1491	ct1608	cn1733	pOP-EAP01334_EST_C_1_pBSK_SK	276
cl1492	ct1609	cn1734	pOP-CEO03121_EST_C_1_pSK_SK	313
cl1492	ct1609	cn1734	pOP-EO04908_EST_C_1_pSK_SK	502
cl1493	ct1610	cn1735	pOP-CEO03131_EST_C_1_pSK_SK	277
cl1493	ct1610	cn1735	pOP-EAP01724_EST_C_1_pBSK_SK	554
cl1494	ct1611	cn1736	pOP-CBP00239_EST_C_1_pBSK_SK	538
cl1494	ct1611	cn1736	pOP-CEO03133_EST_C_1_pSK_SK	353
cl1495	ct1612	cn1737	pOP-EO04108_EST_C_1_pSK_SK	508
cl1495	ct1612	cn1737	pOP-EO04228_EST_C_1_pSK_SK	508
cl1495	ct1612	cn1738	pOP-CEO03138_EST_C_1_pSK_SK	380
cl1496	ct1613	cn1739	pOP-CEO03187_EST_C_1_pSK_SK	554
cl1496	ct1613	cn1740	pOP-CEM00103_EST_C_1_pSK_SK	399
cl1496	ct1613	cn1740	pOP-CEO01993_EST_C_1_pSK_SK	419
cl1496	ct1613	cn1740	pOP-CEO03148_EST_C_1_pSK_SK	456
cl1496	ct1613	cn1740	pOP-EAP01004_EST_C_1_pBSK_SK	224
cl1496	ct1613	cn1740	pOP-EN00600_EST_C_1_pSK_SK	427
cl1496	ct1613	cn1740	pOP-EO02931_EST_C_1_pSK_SK	288
cl1497	ct1614	cn1741	pOP-CEO02868_EST_C_1_pSK_SK	411
cl1497	ct1614	cn1741	pOP-CEO03155_EST_C_1_pSK_SK	546
cl1497	ct1614	cn1741	pOP-CNI01497_EST_C_1_pSK_SK	193
cl1497	ct1614	cn1741	pOP-CNIP01049_EST_C_1_pSK_SK	269
cl1497	ct1614	cn1741	pOP-EAP02266_EST_C_1_pBSK_SK	244
cl1498	ct1615	cn1742	pOP-CEO01469_EST_C_1_pSK_SK	347
cl1498	ct1615	cn1742	pOP-CEO03156_EST_C_1_pSK_SK	347
cl1499	ct1616	cn1743	pOP-CEO03161_EST_C_1_pSK_SK	427
cl1499	ct1616	cn1743	pOP-EAP01980_EST_C_1_pBSK_SK	287
cl1500	ct1617	cn1744	pOP-CEO00845_EST_C_1_pSK_SK	311
cl1500	ct1617	cn1744	pOP-CEO01106_EST_C_1_pSK_SK	261
cl1500	ct1617	cn1744	pOP-CEO03164_EST_C_1_pSK_SK	236
cl1500	ct1617	cn1744	pOP-CNI02134_EST_C_1_pSK_SK	472
cl1500	ct1617	cn1744	pOP-EAP00288_EST_C_1_pBSK_SK	476
cl1501	ct1618	cn1745	pOP-CEO00958_EST_C_1_pSK_SK	478
cl1501	ct1618	cn1745	pOP-CEO03165_EST_C_1_pSK_SK	387
cl1501	ct1618	cn1745	pOP-CNH01319_EST_C_1_pSK_SK	356
cl1502	ct1619	cn1746	pOP-CEO03167_EST_C_1_pSK_SK	283
cl1502	ct1619	cn1746	pOP-CNH04440	457
cl1503	ct1620	cn1747	pOP-CEO03175_EST_C_1_pSK_SK	108
cl1503	ct1620	cn1747	pOP-CNH02093_EST_C_1_pSK_SK	357
cl1503	ct1620	cn1747	pOP-CNH03089_EST_C_1_pSK_SK	464
cl1504	ct1621	cn1748	pOP-CEO03201_EST_C_1_pSK_SK	658
cl1504	ct1621	cn1748	pOP-CNH01527_EST_C_1_pSK_SK	470
cl1505	ct1622	cn1749	pOP-CEO03202_EST_C_1_pSK_SK	306
cl1505	ct1622	cn1749	pOP-CNIP00381_EST_C_1_pSK_SK	337
cl1506	ct1623	cn1750	pOP-CEO03204_EST_C_1_pSK_SK	461
cl1506	ct1623	cn1750	pOP-CNH00900_EST_C_1_pSK_SK	324
cl1506	ct1623	cn1750	pOP-CNH02142_EST_C_1_pSK_SK	544
cl1506	ct1623	cn1750	pOP-CNH02378_EST_C_1_pSK_SK	402
cl1506	ct1623	cn1750	pOP-CNI02207_EST_C_1_pSK_SK	681

cl1506	ct1623	cn1750	pOP-CNIP00113_EST_C_1_pSK_SK	624
cl1506	ct1623	cn1750	pOP-CNIP00658_EST_C_1_pSK_SK	614
cl1507	ct1624	cn1751	pOP-CEO01966_EST_C_1_pSK_SK	316
cl1507	ct1624	cn1751	pOP-CEO03205_EST_C_1_pSK_SK	641
cl1508	ct1625	cn1752	pOP-CEO01316	188
cl1508	ct1625	cn1752	pOP-CEO03209_EST_C_1_pSK_SK	363
cl1509	ct1626	cn1753	pOP-CNIP00882_EST_C_1_pSK_SK	256
cl1509	ct1626	cn1753	pOP-EO05392_EST_C_1_pSK_SK	520
cl1509	ct1626	cn1754	pOP-CEO03215_EST_C_1_pSK_SK	364
cl1510	ct1627	cn1755	pOP-CEO02204_EST_C_1_pSK_SK	438
cl1510	ct1627	cn1755	pOP-CEO03220_EST_C_1_pSK_SK	461
cl1510	ct1627	cn1755	pOP-CNIP00895_EST_C_1_pSK_SK	351
cl1511	ct1628	cn1756	pOP-CEM00067_EST_C_1_pSK_SK	338
cl1511	ct1628	cn1756	pOP-CEO03223_EST_C_1_pSK_SK	177
cl1511	ct1628	cn1756	pOP-EAP00907_EST_C_1_pBSK_SK	163
cl1512	ct1629	cn1757	pOP-CEO03225_EST_C_1_pSK_SK	197
cl1512	ct1629	cn1757	pOP-EAP00139_EST_C_1_pBSK_SK	350
cl1513	ct1630	cn1758	pOP-CEO03227_EST_C_1_pSK_SK	310
cl1513	ct1630	cn1758	pOP-EAP02869_EST_C_1_pBSK_SK	506
cl1514	ct1631	cn1759	pOP-CNHP00496_EST_C_1_pSK_SK	574
cl1514	ct1631	cn1759	pOP-CNI01086_EST_C_1_pSK_SK	381
cl1514	ct1631	cn1759	pOP-CNI02225_EST_C_1_pSK_SK	385
cl1514	ct1631	cn1759	pOP-CNIP00994_EST_C_1_pSK_SK	367
cl1514	ct1631	cn1760	pOP-CEO03228_EST_C_1_pSK_SK	385
cl1515	ct1632	cn1761	pOP-CEO00937_EST_C_1_pSK_SK	515
cl1515	ct1632	cn1761	pOP-CEO03231_EST_C_1_pSK_SK	435
cl1515	ct1632	cn1761	pOP-CNH02530_EST_C_1_pSK_SK	472
cl1515	ct1632	cn1761	pOP-CNH03403_EST_C_1_pSK_SK	538
cl1515	ct1632	cn1761	pOP-CNH03560_EST_C_1_pSK_SK	342
cl1515	ct1632	cn1761	pOP-EAP01577_EST_C_1_pBSK_SK	452
cl1515	ct1632	cn1761	pOP-EAP01835_EST_C_1_pBSK_SK	767
cl1515	ct1632	cn1761	pOP-EO06213_EST_C_1_pSK_SK	308
cl1515	ct1632	cn1761	pOP-EO06250_EST_C_1_pSK_SK	713
cl1515	ct1632	cn1761	pOP-EO06590_EST_C_1_pSK_SK	881
cl1515	ct1632	cn1761	pOP-EO07507_EST_C_1_pSK_SK	719
cl1515	ct1632	cn1761	pOP-EO08339_EST_C_1_pSK_SK	541
cl1515	ct1632	cn1761	pOP-EO08346_EST_C_1_pSK_SK	332
cl1516	ct1633	cn1762	pOP-CEO01940_EST_C_1_pSK_SK	510
cl1516	ct1633	cn1762	pOP-CEO03234_EST_C_1_pSK_SK	236
cl1517	ct1634	cn1763	pOP-CEO03239_EST_C_1_pSK_SK	467
cl1517	ct1634	cn1763	pOP-CNHP00026_EST_C_1_pSK_SK	678
cl1518	ct1635	cn1764	pOP-CEO02845_EST_C_1_pSK_SK	340
cl1518	ct1635	cn1764	pOP-CEO03240_EST_C_1_pSK_SK	486
cl1518	ct1635	cn1764	pOP-CNI01629_EST_C_1_pSK_SK	357
cl1519	ct1636	cn1765	pOP-CEO03246_EST_C_1_pSK_SK	614
cl1519	ct1636	cn1765	pOP-EAP01734_EST_C_1_pBSK_SK	482
cl1520	ct1637	cn1766	pOP-CEO03248_EST_C_1_pSK_SK	440
cl1520	ct1637	cn1766	pOP-CNI01536_EST_C_1_pSK_SK	485
cl1520	ct1637	cn1766	pOP-CNI01962_EST_C_1_pSK_SK	209
cl1520	ct1637	cn1766	pOP-CNIP00982_EST_C_1_pSK_SK	537
cl1521	ct1638	cn1767	pOP-CEO03249_EST_C_1_pSK_SK	157
cl1521	ct1638	cn1767	pOP-CNH02301_EST_C_1_pSK_SK	540
cl1521	ct1638	cn1767	pOP-CNH04277	461
cl1521	ct1638	cn1767	pOP-CNI02098_EST_C_1_pSK_SK	615
cl1522	ct1639	cn1768	pOP-CEO03251_EST_C_1_pSK_SK	224
cl1522	ct1639	cn1768	pOP-EO06206_EST_C_1_pSK_SK	587

cl1523	ct1640	cn1769	pOP-CEO03254_EST_C_1_pSK_SK	363
cl1523	ct1640	cn1769	pOP-EAP02926_EST_C_1_pBSK_SK	600
cl1524	ct1641	cn1770	pOP-CEO03257_EST_C_1_pSK_SK	302
cl1524	ct1641	cn1770	pOP-CNI01864_EST_C_1_pSK_SK	299
cl1525	ct1642	cn1771	pOP-CEO01780_EST_C_1_pSK_SK	161
cl1525	ct1642	cn1771	pOP-CEO03258_EST_C_1_pSK_SK	191
cl1526	ct1643	cn1772	pOP-CEO03274_EST_C_1_pSK_SK	578
cl1526	ct1643	cn1772	pOP-CNI01532_EST_C_1_pSK_SK	333
cl1527	ct1644	cn1773	pOP-CEO03275_EST_C_1_pSK_SK	336
cl1527	ct1644	cn1773	pOP-CNH01924_EST_C_1_pSK_SK	710
cl1528	ct1645	cn1774	pOP-CEO03118_EST_C_1_pSK_SK	356
cl1528	ct1645	cn1774	pOP-CEO03269_EST_C_1_pSK_SK	215
cl1528	ct1645	cn1774	pOP-CEO03277_EST_C_1_pSK_SK	211
cl1528	ct1645	cn1774	pOP-CNH02694_EST_C_1_pSK_SK	586
cl1528	ct1645	cn1774	pOP-CNIP00838_EST_C_1_pSK_SK	266
cl1528	ct1645	cn1774	pOP-EAP00864_EST_C_1_pBSK_SK	588
cl1528	ct1645	cn1774	pOP-EO07725_EST_C_1_pSK_SK	587
cl1529	ct1646	cn1775	pOP-CNH04616	694
cl1529	ct1647	cn1776	pOP-CEO01486_EST_C_1_pSK_SK	439
cl1529	ct1647	cn1776	pOP-CEO01857_EST_C_1_pSK_SK	209
cl1529	ct1647	cn1776	pOP-CEO02971_EST_C_1_pSK_SK	386
cl1529	ct1647	cn1776	pOP-CEO03282_EST_C_1_pSK_SK	214
cl1529	ct1647	cn1776	pOP-CNI01121_EST_C_1_pSK_SK	331
cl1529	ct1647	cn1776	pOP-CNI01130_EST_C_1_pSK_SK	370
cl1529	ct1647	cn1776	pOP-CNI01156_EST_C_1_pSK_SK	443
cl1529	ct1647	cn1776	pOP-CNI01220_EST_C_1_pSK_SK	412
cl1529	ct1647	cn1776	pOP-CNI01455_EST_C_1_pSK_SK	440
cl1529	ct1647	cn1776	pOP-CNI01884_EST_C_1_pSK_SK	358
cl1529	ct1647	cn1776	pOP-CNI02142_EST_C_1_pSK_SK	467
cl1529	ct1647	cn1776	pOP-CNIP00087_EST_C_1_pSK_SK	247
cl1529	ct1647	cn1776	pOP-CNIP04086_EST_C_1_pSK_SK	443
cl1529	ct1647	cn1776	pOP-EAP02234_EST_C_1_pBSK_SK	389
cl1529	ct1647	cn1776	pOP-EO05787_EST_C_1_pSK_SK	462
cl1530	ct1648	cn1777	pOP-CEO03286_EST_C_1_pSK_SK	146
cl1530	ct1648	cn1777	pOP-CNIP00906_EST_C_1_pSK_SK	474
cl1531	ct1649	cn1778	pOP-CEO03288_EST_C_1_pSK_SK	258
cl1531	ct1649	cn1778	pOP-CNH01224_EST_C_1_pSK_SK	621
cl1531	ct1649	cn1778	pOP-CNH02358_EST_C_1_pSK_SK	577
cl1531	ct1649	cn1778	pOP-EAP03225_EST_C_1_pBSK_SK	626
cl1531	ct1649	cn1778	pOP-EAP03764_EST_C_1_pBSK_SK	554
cl1532	ct1650	cn1779	pOP-CEO03314_EST_C_1_pSK_SK	198
cl1532	ct1650	cn1779	pOP-EAP02217_EST_C_1_pBSK_SK	243
cl1533	ct1651	cn1780	pOP-CAP00260_EST_C_1_pBSK_SK	659
cl1533	ct1651	cn1780	pOP-CEO03317_EST_C_1_pSK_SK	276
cl1534	ct1652	cn1781	pOP-CEO03292_EST_C_1_pSK_SK	590
cl1534	ct1652	cn1781	pOP-CEO03319_EST_C_1_pSK_SK	553
cl1535	ct1653	cn1782	pOP-CEO03324_EST_C_1_pSK_SK	135
cl1535	ct1653	cn1782	pOP-CNH04570	720
cl1535	ct1653	cn1782	pOP-CNH04595	743
cl1535	ct1653	cn1782	pOP-CNI01568_EST_C_1_pSK_SK	269
cl1536	ct1654	cn1783	pOP-CEO03325_EST_C_1_pSK_SK	314
cl1536	ct1654	cn1783	pOP-EAP01029_EST_C_1_pBSK_SK	358
cl1536	ct1654	cn1783	pOP-EAP01442_EST_C_1_pBSK_SK	336
cl1537	ct1655	cn1784	pOP-CEO01465_EST_C_1_pSK_SK	454
cl1537	ct1655	cn1784	pOP-CEO01795_EST_C_1_pSK_SK	296
cl1537	ct1655	cn1784	pOP-CEO03334_EST_C_1_pSK_SK	448

cl1538	ct1656	cn1785	pOP-CEO03338_EST_C_1_pSK_SK	349
cl1538	ct1656	cn1785	pOP-CNI02193_EST_C_1_pSK_SK	473
cl1539	ct1657	cn1786	pOP-CEO03347_EST_C_1_pSK_SK	545
cl1539	ct1657	cn1786	pOP-CNI02210_EST_C_1_pSK_SK	498
cl1539	ct1657	cn1786	pOP-CNIP00154_EST_C_1_pSK_SK	610
cl1540	ct1658	cn1787	pOP-CEO03360_EST_C_1_pSK_SK	259
cl1540	ct1658	cn1787	pOP-CNI01465_EST_C_1_pSK_SK	127
cl1541	ct1659	cn1788	pOP-CEM00035_EST_C_1_pSK_SK	359
cl1541	ct1659	cn1788	pOP-CEO03363_EST_C_1_pSK_SK	441
cl1541	ct1659	cn1788	pOP-CEO03366_EST_C_1_pSK_SK	381
cl1541	ct1659	cn1788	pOP-EAP01295_EST_C_1_pBSK_SK	375
cl1542	ct1660	cn1789	pOP-CEO03256_EST_C_1_pSK_SK	233
cl1542	ct1660	cn1789	pOP-CEO03373_EST_C_1_pSK_SK	193
cl1543	ct1661	cn1790	pOP-CEO03378_EST_C_1_pSK_SK	348
cl1543	ct1661	cn1790	pOP-CNIP00312_EST_C_1_pSK_SK	228
cl1544	ct1662	cn1791	pOP-CEO03385_EST_C_1_pSK_SK	333
cl1544	ct1662	cn1791	pOP-EAP03811_EST_C_1_pBSK_SK	623
cl1545	ct1663	cn1792	pOP-CEO03391_EST_C_1_pSK_SK	492
cl1545	ct1663	cn1792	pOP-CNH04803_EST_C_1_pSK_SK	642
cl1545	ct1663	cn1792	pOP-CNH04947_EST_C_1_pSK_SK	684
cl1546	ct1664	cn1793	pOP-CEO03394_EST_C_1_pSK_SK	114
cl1546	ct1664	cn1793	pOP-CNI01105_EST_C_1_pSK_SK	364
cl1546	ct1664	cn1793	pOP-CNI01736_EST_C_1_pSK_SK	496
cl1546	ct1664	cn1793	pOP-EO05183_EST_C_1_pSK_SK	364
cl1546	ct1664	cn1793	pOP-EO06376_EST_C_1_pSK_SK	597
cl1547	ct1665	cn1794	pOP-CEO01709_EST_C_1_pSK_SK	469
cl1547	ct1665	cn1794	pOP-CEO03397_EST_C_1_pSK_SK	345
cl1547	ct1665	cn1794	pOP-CNIP00756_EST_C_1_pSK_SK	566
cl1548	ct1666	cn1795	pOP-CEO03333_EST_C_1_pSK_SK	382
cl1548	ct1666	cn1795	pOP-CEO03401_EST_C_1_pSK_SK	518
cl1549	ct1667	cn1796	pOP-CEO01617_EST_C_1_pSK_SK	312
cl1549	ct1667	cn1796	pOP-CEO03402_EST_C_1_pSK_SK	312
cl1550	ct1668	cn1797	pOP-CEO03410_EST_C_1_pSK_SK	304
cl1550	ct1668	cn1797	pOP-CNH01893_EST_C_1_pSK_SK	594
cl1550	ct1668	cn1797	pOP-CNIP00735_EST_C_1_pSK_SK	281
cl1551	ct1669	cn1798	pOP-CEO03411_EST_C_1_pSK_SK	574
cl1551	ct1669	cn1798	pOP-CNH01688_EST_C_1_pSK_SK	579
cl1551	ct1669	cn1798	pOP-CNH02419_EST_C_1_pSK_SK	559
cl1551	ct1669	cn1798	pOP-CNH02732_EST_C_1_pSK_SK	700
cl1551	ct1669	cn1798	pOP-CNH03479_EST_C_1_pSK_SK	668
cl1552	ct1670	cn1799	pOP-CEO03413_EST_C_1_pSK_SK	490
cl1552	ct1670	cn1799	pOP-CNI01212_EST_C_1_pSK_SK	223
cl1553	ct1671	cn1800	pOP-CEO03415_EST_C_1_pSK_SK	446
cl1553	ct1671	cn1800	pOP-CNI01224_EST_C_1_pSK_SK	609
cl1553	ct1671	cn1800	pOP-CNI01290_EST_C_1_pSK_SK	398
cl1553	ct1671	cn1800	pOP-CNI01921_EST_C_1_pSK_SK	388
cl1553	ct1671	cn1800	pOP-CNI02190_EST_C_1_pSK_SK	494
cl1553	ct1671	cn1800	pOP-CNIP00346_EST_C_1_pSK_SK	401
cl1553	ct1671	cn1800	pOP-CNIP00801_EST_C_1_pSK_SK	398
cl1554	ct1672	cn1801	pOP-CEO03210_EST_C_1_pSK_SK	163
cl1554	ct1672	cn1801	pOP-CEO03416_EST_C_1_pSK_SK	697
cl1554	ct1672	cn1801	pOP-CNI01111_EST_C_1_pSK_SK	415
cl1555	ct1673	cn1802	pOP-CEO03417_EST_C_1_pSK_SK	320
cl1555	ct1673	cn1802	pOP-CNI01781_EST_C_1_pSK_SK	159
cl1556	ct1674	cn1803	pOP-EAP03177_EST_C_1_pBSK_SK	589
cl1556	ct1674	cn1803	pOP-EO05890_EST_C_1_pSK_SK	491

cl1556	ct1674	cn1804	pOP-CEO03419_EST_C_1_pSK_SK	292
cl1557	ct1675	cn1805	pOP-CEO02116_EST_C_1_pSK_SK	274
cl1557	ct1675	cn1805	pOP-CEO02117_EST_C_1_pSK_SK	274
cl1557	ct1675	cn1805	pOP-CEO03428_EST_C_1_pSK_SK	404
cl1558	ct1676	cn1806	pOP-CEO03442_EST_C_1_pSK_SK	567
cl1558	ct1676	cn1806	pOP-CNH01903_EST_C_1_pSK_SK	735
cl1558	ct1676	cn1806	pOP-CNH03250_EST_C_1_pSK_SK	538
cl1559	ct1677	cn1807	pOP-CEO00566_EST_C_1_pSK_SK	112
cl1559	ct1677	cn1807	pOP-CEO00689_EST_C_1_pSK_SK	409
cl1559	ct1677	cn1807	pOP-CEO00693_EST_C_1_pSK_SK	224
cl1559	ct1677	cn1807	pOP-CEO03449_EST_C_1_pSK_SK	255
cl1559	ct1677	cn1807	pOP-CNI01587_EST_C_1_pSK_SK	366
cl1559	ct1677	cn1807	pOP-EAP00549_EST_C_1_pBSK_SK	265
cl1559	ct1677	cn1807	pOP-EAP02258_EST_C_1_pBSK_SK	184
cl1560	ct1678	cn1808	pOP-CEO01649_EST_C_1_pSK_SK	214
cl1560	ct1678	cn1808	pOP-CEO03038_EST_C_1_pSK_SK	290
cl1560	ct1678	cn1808	pOP-CEO03454_EST_C_1_pSK_SK	240
cl1561	ct1679	cn1809	pOP-CEO02163_EST_C_1_pSK_SK	215
cl1561	ct1679	cn1809	pOP-CEO03460_EST_C_1_pSK_SK	324
cl1562	ct1680	cn1810	pOP-CEO03468_EST_C_1_pSK_SK	118
cl1562	ct1680	cn1810	pOP-CNI01795_EST_C_1_pSK_SK	454
cl1563	ct1681	cn1811	pOP-CEO03059_EST_C_1_pSK_SK	293
cl1563	ct1681	cn1811	pOP-CEO03471_EST_C_1_pSK_SK	302
cl1564	ct1682	cn1812	pOP-CEO01464_EST_C_1_pSK_SK	551
cl1564	ct1682	cn1812	pOP-CEO03474_EST_C_1_pSK_SK	172
cl1564	ct1682	cn1812	pOP-CNIP00366_EST_C_1_pSK_SK	345
cl1564	ct1682	cn1812	pOP-CNIP04070_EST_C_1_pSK_SK	448
cl1565	ct1683	cn1813	pOP-CEO03479_EST_C_1_pSK_SK	545
cl1565	ct1683	cn1813	pOP-CNH02679_EST_C_1_pSK_SK	606
cl1565	ct1683	cn1813	pOP-CNH04562	732
cl1566	ct1684	cn1814	pOP-CEO01220_EST_C_1_pSK_SK	250
cl1566	ct1684	cn1814	pOP-CEO03484_EST_C_1_pSK_SK	258
cl1567	ct1685	cn1815	pOP-CEO03487_EST_C_1_pSK_SK	480
cl1567	ct1685	cn1815	pOP-CEO03488_EST_C_1_pSK_SK	383
cl1568	ct1686	cn1816	pOP-CEO01714_EST_C_1_pSK_SK	112
cl1568	ct1686	cn1816	pOP-CEO03496_EST_C_1_pSK_SK	160
cl1569	ct1687	cn1817	pOP-CEO03344_EST_C_1_pSK_SK	431
cl1569	ct1687	cn1817	pOP-CEO03501_EST_C_1_pSK_SK	562
cl1569	ct1687	cn1817	pOP-CNH01468_EST_C_1_pSK_SK	472
cl1569	ct1687	cn1817	pOP-CNH04515	811
cl1569	ct1687	cn1817	pOP-CNHP00104_EST_C_1_pSK_SK	614
cl1570	ct1688	cn1818	pOP-CEO03506_EST_C_1_pSK_SK	134
cl1570	ct1688	cn1818	pOP-EAP03660_EST_C_1_pBSK_SK	393
cl1571	ct1689	cn1819	pOP-CEO00895_EST_C_1_pSK_SK	538
cl1571	ct1689	cn1819	pOP-CEO03264_EST_C_1_pSK_SK	303
cl1571	ct1689	cn1819	pOP-CEO03507_EST_C_1_pSK_SK	589
cl1571	ct1689	cn1819	pOP-CNI01346_EST_C_1_pSK_SK	518
cl1571	ct1689	cn1819	pOP-EAP03747_EST_C_1_pBSK_SK	245
cl1571	ct1689	cn1819	pOP-EAP03760_EST_C_1_pBSK_SK	252
cl1572	ct1690	cn1820	pOP-CEO03518_EST_C_1_pSK_SK	351
cl1572	ct1690	cn1820	pOP-CNH00546_EST_C_1_pSK_SK	278
cl1572	ct1690	cn1820	pOP-CNI01605_EST_C_1_pSK_SK	278
cl1573	ct1691	cn1821	pOP-CEO03526_EST_C_1_pSK_SK	665
cl1573	ct1691	cn1821	pOP-CNH00668_EST_C_1_pSK_SK	518
cl1573	ct1691	cn1821	pOP-CNH04458	661
cl1573	ct1691	cn1821	pOP-CNHP00194_EST_C_1_pSK_SK	657

cl1573	ct1691	cn1821	pOP-CNHP00385_EST_C_1_pSK_SK	701
cl1574	ct1692	cn1822	pOP-CEO03534_EST_C_1_pSK_SK	116
cl1574	ct1692	cn1822	pOP-CNI01630_EST_C_1_pSK_SK	172
cl1574	ct1692	cn1822	pOP-CNIP00843_EST_C_1_pSK_SK	217
cl1575	ct1693	cn1823	pOP-CEM00194_EST_C_1_pSK_SK	128
cl1575	ct1693	cn1823	pOP-CEO01554_EST_C_1_pSK_SK	230
cl1575	ct1693	cn1823	pOP-CEO02071_EST_C_1_pSK_SK	303
cl1575	ct1693	cn1823	pOP-CEO03537_EST_C_1_pSK_SK	185
cl1575	ct1693	cn1823	pOP-CNI01573_EST_C_1_pSK_SK	163
cl1576	ct1694	cn1824	pOP-CEO03531_EST_C_1_pSK_SK	577
cl1576	ct1694	cn1824	pOP-EO07306_EST_C_1_pSK_SK	661
cl1576	ct1694	cn1825	pOP-CEO03540_EST_C_1_pSK_SK	348
cl1576	ct1694	cn1825	pOP-EO07411_EST_C_1_pSK_SK	661
cl1577	ct1695	cn1826	pOP-CEO03349_EST_C_1_pSK_SK	484
cl1577	ct1695	cn1826	pOP-CEO03549_EST_C_1_pSK_SK	655
cl1577	ct1695	cn1826	pOP-CNH02561_EST_C_1_pSK_SK	542
cl1577	ct1695	cn1826	pOP-CNH03007_EST_C_1_pSK_SK	656
cl1577	ct1695	cn1826	pOP-CNH03068_EST_C_1_pSK_SK	664
cl1577	ct1695	cn1826	pOP-CNI01438_EST_C_1_pSK_SK	609
cl1578	ct1696	cn1827	pOP-CEO03553_EST_C_1_pSK_SK	268
cl1578	ct1696	cn1827	pOP-CNH02882_EST_C_1_pSK_SK	554
cl1578	ct1696	cn1827	pOP-CNH03281_EST_C_1_pSK_SK	512
cl1578	ct1696	cn1827	pOP-CNH05031_EST_C_1_pSK_SK	371
cl1579	ct1697	cn1828	pOP-CEO01939_EST_C_1_pSK_SK	551
cl1579	ct1697	cn1828	pOP-CEO03552_EST_C_1_pSK_SK	515
cl1579	ct1697	cn1828	pOP-CEO03555_EST_C_1_pSK_SK	511
cl1580	ct1698	cn1829	pOP-CEO03217_EST_C_1_pSK_SK	605
cl1580	ct1698	cn1829	pOP-CEO03243_EST_C_1_pSK_SK	676
cl1580	ct1698	cn1829	pOP-CEO03559_EST_C_1_pSK_SK	119
cl1580	ct1698	cn1829	pOP-CEO03561_EST_C_1_pSK_SK	150
cl1580	ct1698	cn1829	pOP-CNHP00130_EST_C_1_pSK_SK	563
cl1580	ct1698	cn1829	pOP-CNIP00922_EST_C_1_pSK_SK	719
cl1581	ct1699	cn1830	pOP-CEO02015_EST_C_1_pSK_SK	330
cl1581	ct1699	cn1830	pOP-CEO03566_EST_C_1_pSK_SK	370
cl1581	ct1699	cn1830	pOP-EAP01311_EST_C_1_pBSK_SK	593
cl1581	ct1699	cn1830	pOP-EAP01400_EST_C_1_pBSK_SK	352
cl1581	ct1699	cn1830	pOP-EAP01691_EST_C_1_pBSK_SK	325
cl1581	ct1699	cn1830	pOP-EAP01731_EST_C_1_pBSK_SK	508
cl1582	ct1700	cn1831	pOP-CEO03570_EST_C_1_pSK_SK	193
cl1582	ct1700	cn1831	pOP-EAP01335_EST_C_1_pBSK_SK	530
cl1582	ct1700	cn1831	pOP-EAP01642_EST_C_1_pBSK_SK	374
cl1582	ct1700	cn1831	pOP-EAP02388_EST_C_1_pBSK_SK	446
cl1582	ct1700	cn1831	pOP-EAP03160_EST_C_1_pBSK_SK	555
cl1582	ct1700	cn1831	pOP-EAP03303_EST_C_1_pBSK_SK	315
cl1582	ct1700	cn1831	pOP-EAP03376_EST_C_1_pBSK_SK	383
cl1583	ct1701	cn1832	pOP-CEO02306_EST_C_1_pSK_SK	191
cl1583	ct1701	cn1832	pOP-CEO03576_EST_C_1_pSK_SK	107
cl1584	ct1702	cn1833	pOP-CEO03578_EST_C_1_pSK_SK	364
cl1584	ct1702	cn1833	pOP-CNIP00133_EST_C_1_pSK_SK	473
cl1584	ct1702	cn1833	pOP-EAP00498_EST_C_1_pBSK_SK	538
cl1584	ct1702	cn1833	pOP-EAP03743_EST_C_1_pBSK_SK	353
cl1585	ct1703	cn1834	pOP-CEO03579_EST_C_1_pSK_SK	295
cl1585	ct1703	cn1834	pOP-CNIP00717_EST_C_1_pSK_SK	319
cl1585	ct1703	cn1834	pOP-EBP03124_EST_C_1_pBSK_M13F	233
cl1586	ct1704	cn1835	pOP-CBP00020_EST_C_1_pBSK_SK	241
cl1586	ct1704	cn1835	pOP-CEO03581_EST_C_1_pSK_SK	219

cl1587	ct1705	cn1836	pOP-CEO03591_EST_C_1_pSK_SK	248
cl1587	ct1705	cn1836	pOP-CNI01273_EST_C_1_pSK_SK	366
cl1587	ct1705	cn1836	pOP-CNIP00628_EST_C_1_pSK_SK	208
cl1588	ct1706	cn1837	pOP-CEO03595_EST_C_1_pSK_SK	192
cl1588	ct1706	cn1837	pOP-CNIP00195_EST_C_1_pSK_SK	434
cl1589	ct1707	cn1838	pOP-CEO03594_EST_C_1_pSK_SK	245
cl1589	ct1707	cn1838	pOP-CEO03602_EST_C_1_pSK_SK	427
cl1590	ct1708	cn1839	pOP-CEO03606_EST_C_1_pSK_SK	387
cl1590	ct1708	cn1839	pOP-CNH01217_EST_C_1_pSK_SK	494
cl1590	ct1708	cn1839	pOP-CNH02673_EST_C_1_pSK_SK	622
cl1590	ct1708	cn1839	pOP-CNH04154	558
cl1590	ct1708	cn1839	pOP-CNI01540_EST_C_1_pSK_SK	196
cl1591	ct1709	cn1840	pOP-CEO01932_EST_C_1_pSK_SK	328
cl1591	ct1709	cn1840	pOP-CEO03614_EST_C_1_pSK_SK	629
cl1591	ct1709	cn1840	pOP-EAP01475_EST_C_1_pBSK_SK	616
cl1592	ct1710	cn1841	pOP-CAP00419_EST_C_1_pBSK_SK	656
cl1592	ct1710	cn1841	pOP-CEO01441_EST_C_1_pSK_SK	262
cl1592	ct1710	cn1841	pOP-CEO03617_EST_C_1_pSK_SK	446
cl1592	ct1710	cn1841	pOP-EAP03778_EST_C_1_pBSK_SK	441
cl1593	ct1711	cn1842	pOP-CEO03502_EST_C_1_pSK_SK	294
cl1593	ct1711	cn1842	pOP-EAP03381_EST_C_1_pBSK_SK	366
cl1593	ct1712	cn1843	pOP-CEO01203_EST_C_1_pSK_SK	227
cl1593	ct1712	cn1843	pOP-CEO01977_EST_C_1_pSK_SK	305
cl1593	ct1712	cn1843	pOP-CEO03618_EST_C_1_pSK_SK	392
cl1594	ct1713	cn1844	pOP-CEO03599_EST_C_1_pSK_SK	413
cl1594	ct1713	cn1844	pOP-CEO03619_EST_C_1_pSK_SK	413
cl1595	ct1714	cn1845	pOP-CEO03621_EST_C_1_pSK_SK	469
cl1595	ct1714	cn1845	pOP-CNIP01006_EST_C_1_pSK_SK	324
cl1595	ct1714	cn1845	pOP-EAP01549_EST_C_1_pBSK_SK	381
cl1596	ct1715	cn1846	pOP-CEO03626_EST_C_1_pSK_SK	201
cl1596	ct1715	cn1846	pOP-CNH01256_EST_C_1_pSK_SK	561
cl1596	ct1715	cn1846	pOP-CNH01770_EST_C_1_pSK_SK	550
cl1596	ct1715	cn1846	pOP-CNH03033_EST_C_1_pSK_SK	411
cl1596	ct1716	cn1847	pOP-CAP00224_EST_C_1_pBSK_SK	645
cl1596	ct1716	cn1847	pOP-CNH02357_EST_C_1_pSK_SK	662
cl1596	ct1716	cn1847	pOP-EAP01544_EST_C_1_pBSK_SK	620
cl1596	ct1716	cn1847	pOP-EAP01801_EST_C_1_pBSK_SK	540
cl1597	ct1717	cn1848	pOP-CEO03624_EST_C_1_pSK_SK	490
cl1597	ct1717	cn1848	pOP-CEO03627_EST_C_1_pSK_SK	537
cl1598	ct1718	cn1849	pOP-CEO01822_EST_C_1_pSK_SK	153
cl1598	ct1718	cn1849	pOP-CEO03630_EST_C_1_pSK_SK	300
cl1598	ct1718	cn1849	pOP-CNI01429_EST_C_1_pSK_SK	369
cl1599	ct1719	cn1850	pOP-CEO03635_EST_C_1_pSK_SK	208
cl1599	ct1719	cn1850	pOP-EAP02360_EST_C_1_pBSK_SK	449
cl1600	ct1720	cn1851	pOP-CEO03328_EST_C_1_pSK_SK	530
cl1600	ct1720	cn1851	pOP-CEO03638_EST_C_1_pSK_SK	525
cl1601	ct1721	cn1852	pOP-CEO03639_EST_C_1_pSK_SK	287
cl1601	ct1721	cn1852	pOP-EAP01510_EST_C_1_pBSK_SK	236
cl1602	ct1722	cn1853	pOP-CEO03652_EST_C_1_pSK_SK	357
cl1602	ct1722	cn1853	pOP-EAP03140_EST_C_1_pBSK_SK	422
cl1603	ct1723	cn1854	pOP-CNI01979_EST_C_1_pSK_SK	370
cl1603	ct1723	cn1854	pOP-CNI01990_EST_C_1_pSK_SK	412
cl1603	ct1723	cn1854	pOP-CNI02009_EST_C_1_pSK_SK	532
cl1603	ct1724	cn1855	pOP-CEO03653_EST_C_1_pSK_SK	471
cl1603	ct1724	cn1855	pOP-CNH00903_EST_C_1_pSK_SK	393
cl1603	ct1724	cn1855	pOP-CNH00964_EST_C_1_pSK_SK	599

cl1603	ct1724	cn1855	pOP-CNH03356_EST_C_1_pSK_SK	729
cl1603	ct1724	cn1855	pOP-CNH03358_EST_C_1_pSK_SK	679
cl1603	ct1724	cn1855	pOP-CNH04636	727
cl1604	ct1725	cn1856	pOP-CAP00351_EST_C_1_pBSK_SK	547
cl1604	ct1725	cn1856	pOP-CNIP04069_EST_C_1_pSK_SK	413
cl1604	ct1725	cn1856	pOP-EO05091_EST_C_1_pSK_SK	483
cl1604	ct1725	cn1857	pOP-CEO03654_EST_C_1_pSK_SK	302
cl1605	ct1726	cn1858	pOP-CEO02451_EST_C_1_pSK_SK	322
cl1605	ct1726	cn1858	pOP-CEO03655_EST_C_1_pSK_SK	291
cl1606	ct1727	cn1859	pOP-CEO00855_EST_C_1_pSK_SK	603
cl1606	ct1727	cn1859	pOP-CEO03669_EST_C_1_pSK_SK	281
cl1606	ct1727	cn1859	pOP-CNH00834_EST_C_1_pSK_SK	343
cl1606	ct1727	cn1859	pOP-CNH00957_EST_C_1_pSK_SK	574
cl1607	ct1728	cn1860	pOP-CEO03656_EST_C_1_pSK_SK	501
cl1607	ct1728	cn1860	pOP-CEO03670_EST_C_1_pSK_SK	369
cl1608	ct1729	cn1861	pOP-CEO00899_EST_C_1_pSK_SK	116
cl1608	ct1729	cn1861	pOP-CEO03671_EST_C_1_pSK_SK	118
cl1609	ct1730	cn1862	pOP-CEO03678_EST_C_1_pSK_SK	259
cl1609	ct1730	cn1862	pOP-EAP01126_EST_C_1_pBSK_SK	215
cl1609	ct1730	cn1862	pOP-EAP01699_EST_C_1_pBSK_SK	416
cl1609	ct1730	cn1862	pOP-EAP03346_EST_C_1_pBSK_SK	164
cl1609	ct1730	cn1862	pOP-EAP03380_EST_C_1_pBSK_SK	147
cl1609	ct1730	cn1862	pOP-EAP05007_EST_C_1_pBSK_SK	604
cl1609	ct1730	cn1863	pOP-EAP01569_EST_C_1_pBSK_SK	505
cl1610	ct1731	cn1864	pOP-CBP00229_EST_C_1_pBSK_SK	516
cl1610	ct1731	cn1864	pOP-CEO03696_EST_C_1_pSK_SK	329
cl1610	ct1731	cn1864	pOP-CNI01332_EST_C_1_pSK_SK	367
cl1610	ct1731	cn1864	pOP-CNIP00095_EST_C_1_pSK_SK	524
cl1610	ct1731	cn1864	pOP-CNIP00519_EST_C_1_pSK_SK	351
cl1611	ct1732	cn1865	pOP-CEO03698_EST_C_1_pSK_SK	520
cl1611	ct1732	cn1865	pOP-CNIP00802_EST_C_1_pSK_SK	470
cl1611	ct1732	cn1865	pOP-EAP00814_EST_C_1_pBSK_SK	189
cl1611	ct1732	cn1865	pOP-EAP01663_EST_C_1_pBSK_SK	640
cl1612	ct1733	cn1866	pOP-CEO02458_EST_C_1_pSK_SK	141
cl1612	ct1733	cn1866	pOP-CEO02556_EST_C_1_pSK_SK	519
cl1612	ct1733	cn1866	pOP-CEO03699_EST_C_1_pSK_SK	271
cl1612	ct1733	cn1866	pOP-EAP00114_EST_C_1_pBSK_SK	626
cl1612	ct1733	cn1866	pOP-EAP02982_EST_C_1_pBSK_SK	335
cl1612	ct1733	cn1866	pOP-EO02968_EST_C_1_pSK_SK	447
cl1612	ct1733	cn1866	pOP-EO03387_EST_C_1_pSK_SK	464
cl1612	ct1733	cn1866	pOP-EO04935_EST_C_1_pSK_SK	523
cl1612	ct1734	cn1867	pOP-CEO00775_EST_C_1_pSK_SK	150
cl1612	ct1734	cn1867	pOP-CEO00776_EST_C_1_pSK_SK	332
cl1612	ct1734	cn1867	pOP-CEO01518_EST_C_1_pSK_SK	489
cl1612	ct1734	cn1867	pOP-CNH00907_EST_C_1_pSK_SK	394
cl1612	ct1734	cn1867	pOP-CNH03812_EST_C_1_pSK_SK	549
cl1612	ct1734	cn1867	pOP-CNI01367_EST_C_1_pSK_SK	368
cl1612	ct1734	cn1867	pOP-CNI01765_EST_C_1_pSK_SK	368
cl1612	ct1734	cn1867	pOP-EAP00863_EST_C_1_pBSK_SK	632
cl1612	ct1734	cn1867	pOP-EAP02775_EST_C_1_pBSK_SK	459
cl1612	ct1734	cn1867	pOP-EAP02962_EST_C_1_pBSK_SK	326
cl1612	ct1734	cn1867	pOP-EO05002_EST_C_1_pSK_SK	499
cl1612	ct1734	cn1867	pOP-EO08142_EST_C_1_pSK_SK	487
cl1613	ct1735	cn1868	pOP-CEO02713_EST_C_1_pSK_SK	394
cl1613	ct1735	cn1868	pOP-CEO03700_EST_C_1_pSK_SK	446
cl1613	ct1735	cn1868	pOP-CNH02535_EST_C_1_pSK_SK	445

cl1614	ct1736	cn1869	pOP-CEO01756_EST_C_1_pSK_SK	372
cl1614	ct1736	cn1869	pOP-CEO03702_EST_C_1_pSK_SK	398
cl1615	ct1737	cn1870	pOP-CEO00968_EST_C_1_pSK_SK	195
cl1615	ct1737	cn1870	pOP-CNIP00319_EST_C_1_pSK_SK	180
cl1615	ct1738	cn1871	pOP-CEO03076_EST_C_1_pSK_SK	254
cl1615	ct1738	cn1871	pOP-CEO03703_EST_C_1_pSK_SK	250
cl1616	ct1739	cn1872	pOP-CEO03704_EST_C_1_pSK_SK	497
cl1616	ct1739	cn1872	pOP-CNH00716_EST_C_1_pSK_SK	506
cl1616	ct1739	cn1872	pOP-CNH02960_EST_C_1_pSK_SK	490
cl1617	ct1740	cn1873	pOP-CEO03708_EST_C_1_pSK_SK	439
cl1617	ct1740	cn1874	pOP-CEO03357_EST_C_1_pSK_SK	296
cl1617	ct1740	cn1874	pOP-CEO03359_EST_C_1_pSK_SK	302
cl1618	ct1741	cn1875	pOP-CEO03713_EST_C_1_pSK_SK	423
cl1618	ct1741	cn1875	pOP-EAP02904_EST_C_1_pBSK_SK	707
cl1619	ct1742	cn1876	pOP-CEO03726_EST_C_1_pSK_SK	433
cl1619	ct1742	cn1876	pOP-EAP02805_EST_C_1_pBSK_SK	598
cl1620	ct1743	cn1877	pOP-CEO03727_EST_C_1_pSK_SK	277
cl1620	ct1743	cn1877	pOP-CNIP00142_EST_C_1_pSK_SK	233
cl1621	ct1744	cn1878	pOP-CEO03733_EST_C_1_pSK_SK	277
cl1621	ct1744	cn1878	pOP-EAP02237_EST_C_1_pBSK_SK	238
cl1622	ct1745	cn1879	pOP-CEO03740_EST_C_1_pSK_SK	214
cl1622	ct1745	cn1879	pOP-CNI02145_EST_C_1_pSK_SK	474
cl1623	ct1746	cn1880	pOP-CEO00534_EST_C_1_pSK_SK	171
cl1623	ct1746	cn1880	pOP-CEO02611_EST_C_1_pSK_SK	169
cl1623	ct1746	cn1880	pOP-CEO03741_EST_C_1_pSK_SK	171
cl1624	ct1747	cn1881	pOP-CEO01623_EST_C_1_pSK_SK	485
cl1624	ct1747	cn1881	pOP-CEO03744_EST_C_1_pSK_SK	391
cl1624	ct1747	cn1881	pOP-CNIP00552_EST_C_1_pSK_SK	648
cl1624	ct1747	cn1881	pOP-CNIP04017_EST_C_1_pSK_SK	410
cl1625	ct1748	cn1882	pOP-CAP00102_EST_C_1_pBSK_SK	452
cl1625	ct1748	cn1882	pOP-CBP00102_EST_C_1_pBSK_SK	467
cl1625	ct1748	cn1882	pOP-CEO03746_EST_C_1_pSK_SK	312
cl1625	ct1748	cn1882	pOP-CNI01223_EST_C_1_pSK_SK	511
cl1625	ct1748	cn1882	pOP-CNI01809_EST_C_1_pSK_SK	391
cl1625	ct1748	cn1882	pOP-CNI01994_EST_C_1_pSK_SK	203
cl1626	ct1749	cn1883	pOP-CEO01119_EST_C_1_pSK_SK	378
cl1626	ct1749	cn1883	pOP-CEO03753_EST_C_1_pSK_SK	147
cl1627	ct1750	cn1884	pOP-CEO03519_EST_C_1_pSK_SK	509
cl1627	ct1750	cn1884	pOP-CEO03754_EST_C_1_pSK_SK	395
cl1627	ct1750	cn1884	pOP-CNH02218_EST_C_1_pSK_SK	411
cl1627	ct1750	cn1884	pOP-CNH02347_EST_C_1_pSK_SK	472
cl1627	ct1750	cn1884	pOP-CNI01652_EST_C_1_pSK_SK	474
cl1628	ct1751	cn1885	pOP-CEO03764_EST_C_1_pSK_SK	716
cl1628	ct1751	cn1886	pOP-EO04862_EST_C_1_pSK_SK	453
cl1629	ct1752	cn1887	pOP-CEO03771_EST_C_1_pSK_SK	483
cl1629	ct1752	cn1887	pOP-CNH04489	575
cl1629	ct1752	cn1887	pOP-CNI01489_EST_C_1_pSK_SK	538
cl1630	ct1753	cn1888	pOP-CEO03778_EST_C_1_pSK_SK	490
cl1630	ct1753	cn1888	pOP-EO05770_EST_C_1_pSK_SK	477
cl1631	ct1754	cn1889	pOP-CEM00229_EST_C_1_pSK_SK	223
cl1631	ct1754	cn1889	pOP-CEO01746_EST_C_1_pSK_SK	285
cl1631	ct1754	cn1889	pOP-CEO03780_EST_C_1_pSK_SK	456
cl1631	ct1754	cn1889	pOP-EAP01562_EST_C_1_pBSK_SK	435
cl1631	ct1754	cn1889	pOP-EAP02317_EST_C_1_pBSK_SK	628
cl1631	ct1754	cn1889	pOP-EO02561_EST_C_1_pSK_SK	476
cl1631	ct1754	cn1889	pOP-EO08170_EST_C_1_pSK_SK	538

cl1631	ct1754	cn1890	pOP-EAP02994_EST_C_1_pBSK_SK	543
cl1632	ct1755	cn1891	pOP-CEO01571_EST_C_1_pSK_SK	250
cl1632	ct1755	cn1891	pOP-CEO03737_EST_C_1_pSK_SK	320
cl1632	ct1755	cn1891	pOP-CEO03785_EST_C_1_pSK_SK	206
cl1632	ct1755	cn1891	pOP-CNIP00569_EST_C_1_pSK_SK	511
cl1632	ct1755	cn1891	pOP-EO08345_EST_C_1_pSK_SK	382
cl1633	ct1756	cn1892	pOP-CEO03790_EST_C_1_pSK_SK	302
cl1633	ct1756	cn1892	pOP-CNH02076_EST_C_1_pSK_SK	519
cl1633	ct1756	cn1892	pOP-CNH03504_EST_C_1_pSK_SK	601
cl1633	ct1756	cn1892	pOP-CNH04257	518
cl1633	ct1756	cn1892	pOP-CNI01948_EST_C_1_pSK_SK	556
cl1633	ct1756	cn1892	pOP-CNIP01070_EST_C_1_pSK_SK	474
cl1633	ct1756	cn1892	pOP-EAP03122_EST_C_1_pBSK_SK	488
cl1633	ct1756	cn1892	pOP-EAP03637_EST_C_1_pBSK_SK	635
cl1633	ct1756	cn1892	pOP-EO02056_EST_C_1_pSK_SK	648
cl1633	ct1756	cn1892	pOP-EO07058_EST_C_1_pSK_SK	533
cl1633	ct1756	cn1893	pOP-CNH02114_EST_C_1_pSK_SK	464
cl1634	ct1757	cn1894	pOP-CEOP00015_EST_C_1_pSK_SK	150
cl1634	ct1757	cn1894	pOP-CNH02476_EST_C_1_pSK_SK	459
cl1634	ct1757	cn1894	pOP-CNIP00566_EST_C_1_pSK_SK	352
cl1635	ct1758	cn1895	pOP-CEOP00022_EST_C_1_pSK_SK	231
cl1635	ct1758	cn1895	pOP-EAP00794_EST_C_1_pBSK_SK	279
cl1635	ct1758	cn1895	pOP-EAP01983_EST_C_1_pBSK_SK	183
cl1635	ct1758	cn1895	pOP-EO02359_EST_C_1_pSK_SK	459
cl1635	ct1758	cn1895	pOP-EO02361_EST_C_1_pSK_SK	670
cl1635	ct1758	cn1895	pOP-EO05748_EST_C_1_pSK_SK	516
cl1636	ct1759	cn1896	pOP-CAP05006_EST_C_1_pBSK_SK	654
cl1636	ct1759	cn1896	pOP-CEO01110_EST_C_1_pSK_SK	268
cl1636	ct1759	cn1896	pOP-CEOP00023_EST_C_1_pSK_SK	168
cl1636	ct1759	cn1896	pOP-CNIP00545_EST_C_1_pSK_SK	285
cl1636	ct1759	cn1896	pOP-CNIP00711_EST_C_1_pSK_SK	470
cl1636	ct1759	cn1896	pOP-EAP02180_EST_C_1_pBSK_SK	224
cl1636	ct1759	cn1896	pOP-EAP03773_EST_C_1_pBSK_SK	259
cl1637	ct1760	cn1897	pOP-CNH04495	562
cl1637	ct1760	cn1898	pOP-CEOP00028_EST_C_1_pSK_SK	275
cl1638	ct1761	cn1899	pOP-CEO02156_EST_C_1_pSK_SK	452
cl1638	ct1761	cn1899	pOP-CEO02158_EST_C_1_pSK_SK	452
cl1638	ct1761	cn1899	pOP-CEOP00032_EST_C_1_pSK_SK	564
cl1638	ct1761	cn1899	pOP-CNI02035_EST_C_1_pSK_SK	473
cl1638	ct1761	cn1899	pOP-CNIP00344_EST_C_1_pSK_SK	454
cl1638	ct1761	cn1899	pOP-EAP01634_EST_C_1_pBSK_SK	578
cl1639	ct1762	cn1900	pOP-CEO01988_EST_C_1_pSK_SK	185
cl1639	ct1762	cn1900	pOP-CEO03214_EST_C_1_pSK_SK	185
cl1639	ct1762	cn1900	pOP-CEOP00035_EST_C_1_pSK_SK	185
cl1640	ct1763	cn1901	pOP-CEO03150_EST_C_1_pSK_SK	472
cl1640	ct1763	cn1901	pOP-CEOP00038_EST_C_1_pSK_SK	397
cl1641	ct1764	cn1902	pOP-CEOP00046_EST_C_1_pSK_SK	211
cl1641	ct1764	cn1902	pOP-CNH00636_EST_C_1_pSK_SK	485
cl1641	ct1764	cn1902	pOP-CNH01344_EST_C_1_pSK_SK	491
cl1641	ct1764	cn1902	pOP-CNH01447_EST_C_1_pSK_SK	484
cl1641	ct1764	cn1902	pOP-CNHP00300_EST_C_1_pSK_SK	609
cl1642	ct1765	cn1903	pOP-CEO02452_EST_C_1_pSK_SK	227
cl1642	ct1765	cn1903	pOP-CEOP00051_EST_C_1_pSK_SK	414
cl1643	ct1766	cn1904	pOP-CNI01271_EST_C_1_pSK_SK	310
cl1643	ct1766	cn1904	pOP-CNI02220_EST_C_1_pSK_SK	205
cl1643	ct1766	cn1904	pOP-EO06373_EST_C_1_pSK_SK	716

cl1643	ct1766	cn1904	pOP-EO07026_EST_C_1_pSK_SK	686
cl1643	ct1766	cn1904	pOP-EO07274_EST_C_1_pSK_SK	779
cl1643	ct1767	cn1905	pOP-EAP00274_EST_C_1_pBSK_SK	359
cl1643	ct1767	cn1905	pOP-EAP02066_EST_C_1_pBSK_SK	257
cl1643	ct1767	cn1905	pOP-EAP03323_EST_C_1_pBSK_SK	444
cl1643	ct1767	cn1905	pOP-EO02003_EST_C_1_pSK_SK	604
cl1643	ct1767	cn1905	pOP-EO02004_EST_C_1_pSK_SK	365
cl1643	ct1767	cn1905	pOP-EO02900_EST_C_1_pSK_SK	436
cl1643	ct1767	cn1905	pOP-EO05885_EST_C_1_pSK_SK	549
cl1644	ct1768	cn1906	pOP-EO02005_EST_C_1_pSK_SK	610
cl1644	ct1768	cn1906	pOP-EO07075_EST_C_1_pSK_SK	537
cl1645	ct1769	cn1907	pOP-CEO03271_EST_C_1_pSK_SK	367
cl1645	ct1769	cn1907	pOP-CNH00844_EST_C_1_pSK_SK	586
cl1645	ct1769	cn1907	pOP-CNIP00920_EST_C_1_pSK_SK	477
cl1645	ct1769	cn1907	pOP-EAP03448_EST_C_1_pBSK_SK	285
cl1645	ct1769	cn1907	pOP-EAP03842_EST_C_1_pBSK_SK	399
cl1645	ct1769	cn1907	pOP-EO02006_EST_C_1_pSK_SK	617
cl1646	ct1770	cn1908	pOP-EAP01031_EST_C_1_pBSK_SK	510
cl1646	ct1770	cn1908	pOP-EAP01259_EST_C_1_pBSK_SK	201
cl1646	ct1770	cn1908	pOP-EAP01296_EST_C_1_pBSK_SK	404
cl1646	ct1770	cn1908	pOP-EAP01310_EST_C_1_pBSK_SK	371
cl1646	ct1770	cn1908	pOP-EAP01445_EST_C_1_pBSK_SK	105
cl1646	ct1770	cn1908	pOP-EAP01861_EST_C_1_pBSK_SK	279
cl1646	ct1770	cn1908	pOP-EAP03716_EST_C_1_pBSK_SK	387
cl1646	ct1770	cn1908	pOP-EO02011_EST_C_1_pSK_SK	427
cl1647	ct1771	cn1909	pOP-EO02013_EST_C_1_pSK_SK	165
cl1647	ct1771	cn1909	pOP-EO06314_EST_C_1_pSK_SK	606
cl1648	ct1772	cn1910	pOP-CNH02121_EST_C_1_pSK_SK	472
cl1648	ct1772	cn1910	pOP-EO02015_EST_C_1_pSK_SK	599
cl1648	ct1772	cn1910	pOP-EO07936_EST_C_1_pSK_SK	371
cl1649	ct1773	cn1911	pOP-CNI01094_EST_C_1_pSK_SK	449
cl1649	ct1773	cn1911	pOP-CNIP00780_EST_C_1_pSK_SK	425
cl1649	ct1773	cn1911	pOP-EO02024_EST_C_1_pSK_SK	516
cl1650	ct1774	cn1912	pOP-EO02044_EST_C_1_pSK_SK	739
cl1650	ct1774	cn1913	pOP-CNH01580_EST_C_1_pSK_SK	613
cl1650	ct1774	cn1913	pOP-CNI01968_EST_C_1_pSK_SK	436
cl1651	ct1775	cn1914	pOP-CAP00198_EST_C_1_pBSK_SK	274
cl1651	ct1775	cn1914	pOP-CNH04972_EST_C_1_pSK_SK	296
cl1651	ct1775	cn1914	pOP-CNH05004_EST_C_1_pSK_SK	383
cl1651	ct1775	cn1914	pOP-EAP03309_EST_C_1_pBSK_SK	662
cl1651	ct1775	cn1914	pOP-EO02045_EST_C_1_pSK_SK	653
cl1652	ct1776	cn1915	pOP-CEM00219_EST_C_1_pSK_SK	273
cl1652	ct1776	cn1915	pOP-EO02046_EST_C_1_pSK_SK	717
cl1653	ct1777	cn1916	pOP-CEO02495_EST_C_1_pSK_SK	442
cl1653	ct1777	cn1916	pOP-EO02047_EST_C_1_pSK_SK	390
cl1654	ct1778	cn1917	pOP-CNHP00293_EST_C_1_pSK_SK	486
cl1654	ct1778	cn1917	pOP-CNIP00916_EST_C_1_pSK_SK	670
cl1654	ct1778	cn1917	pOP-EAP02957_EST_C_1_pBSK_SK	521
cl1654	ct1778	cn1917	pOP-EO02083_EST_C_1_pSK_SK	670
cl1655	ct1779	cn1918	pOP-CEOP00026_EST_C_1_pSK_SK	440
cl1655	ct1779	cn1918	pOP-EAP01837_EST_C_1_pBSK_SK	673
cl1655	ct1779	cn1918	pOP-EAP01976_EST_C_1_pBSK_SK	238
cl1655	ct1779	cn1918	pOP-EO02094_EST_C_1_pSK_SK	413
cl1655	ct1779	cn1918	pOP-EO04236_EST_C_1_pSK_SK	411
cl1656	ct1780	cn1919	pOP-CEO02553_EST_C_1_pSK_SK	603
cl1656	ct1780	cn1919	pOP-EO02119_EST_C_1_pSK_SK	631

cl1657	ct1781	cn1920	pOP-CEO03033_EST_C_1_pSK_SK	463
cl1657	ct1781	cn1920	pOP-CNH01575_EST_C_1_pSK_SK	637
cl1657	ct1781	cn1920	pOP-CNH02617_EST_C_1_pSK_SK	338
cl1657	ct1781	cn1920	pOP-EO02120_EST_C_1_pSK_SK	530
cl1658	ct1782	cn1921	pOP-CNI01529_EST_C_1_pSK_SK	334
cl1658	ct1782	cn1921	pOP-EO02128_EST_C_1_pSK_SK	327
cl1659	ct1783	cn1922	pOP-CEM00133_EST_C_1_pSK_SK	285
cl1659	ct1783	cn1922	pOP-CEMP00022_EST_C_1_pSK_SK	285
cl1659	ct1783	cn1922	pOP-EAP01007_EST_C_1_pBSK_SK	569
cl1659	ct1783	cn1922	pOP-EO02135_EST_C_1_pSK_SK	588
cl1660	ct1784	cn1923	pOP-EO02085_EST_C_1_pSK_SK	530
cl1660	ct1785	cn1924	pOP-CNI01167_EST_C_1_pSK_SK	417
cl1660	ct1785	cn1924	pOP-EAP00418_EST_C_1_pBSK_SK	188
cl1660	ct1785	cn1924	pOP-EO06642_EST_C_1_pSK_SK	639
cl1660	ct1785	cn1924	pOP-EO06781_EST_C_1_pSK_SK	835
cl1660	ct1785	cn1924	pOP-EO08241_EST_C_1_pSK_SK	503
cl1660	ct1786	cn1925	pOP-CEO01774_EST_C_1_pSK_SK	334
cl1660	ct1786	cn1925	pOP-CEO02698_EST_C_1_pSK_SK	417
cl1660	ct1786	cn1925	pOP-CNIP00946_EST_C_1_pSK_SK	509
cl1660	ct1786	cn1925	pOP-EAP00882_EST_C_1_pBSK_SK	192
cl1660	ct1786	cn1925	pOP-EO02141_EST_C_1_pSK_SK	490
cl1660	ct1786	cn1925	pOP-EO05666_EST_C_1_pSK_SK	469
cl1660	ct1786	cn1926	pOP-EO06098_EST_C_1_pSK_SK	591
cl1661	ct1787	cn1927	pOP-CEO01342	222
cl1661	ct1787	cn1927	pOP-CEO03467_EST_C_1_pSK_SK	347
cl1661	ct1787	cn1927	pOP-CNH00657_EST_C_1_pSK_SK	499
cl1661	ct1787	cn1927	pOP-CNHP00059_EST_C_1_pSK_SK	602
cl1661	ct1787	cn1927	pOP-EO02142_EST_C_1_pSK_SK	468
cl1661	ct1787	cn1927	pOP-EO06404_EST_C_1_pSK_SK	565
cl1662	ct1788	cn1928	pOP-CEO00767_EST_C_1_pSK_SK	372
cl1662	ct1788	cn1928	pOP-EO02145_EST_C_1_pSK_SK	507
cl1663	ct1789	cn1929	pOP-CEO01505_EST_C_1_pSK_SK	345
cl1663	ct1789	cn1929	pOP-EAP01550_EST_C_1_pBSK_SK	579
cl1663	ct1789	cn1929	pOP-EO02166_EST_C_1_pSK_SK	565
cl1664	ct1790	cn1930	pOP-CNI01688_EST_C_1_pSK_SK	220
cl1664	ct1790	cn1930	pOP-EO02169_EST_C_1_pSK_SK	348
cl1665	ct1791	cn1931	pOP-CNH03005_EST_C_1_pSK_SK	372
cl1665	ct1791	cn1931	pOP-EO02171_EST_C_1_pSK_SK	510
cl1666	ct1792	cn1932	pOP-EO02184_EST_C_1_pSK_SK	653
cl1666	ct1792	cn1932	pOP-EO02185_EST_C_1_pSK_SK	653
cl1667	ct1793	cn1933	pOP-CNH00833_EST_C_1_pSK_SK	311
cl1667	ct1793	cn1933	pOP-EO02191_EST_C_1_pSK_SK	622
cl1668	ct1794	cn1934	pOP-EO02197_EST_C_1_pSK_SK	603
cl1668	ct1794	cn1934	pOP-EO07494_EST_C_1_pSK_SK	702
cl1668	ct1795	cn1935	pOP-CEO01147_EST_C_1_pSK_SK	201
cl1668	ct1795	cn1935	pOP-CEO01155_EST_C_1_pSK_SK	201
cl1668	ct1795	cn1935	pOP-CEO02773_EST_C_1_pSK_SK	257
cl1668	ct1795	cn1935	pOP-CNI02000_EST_C_1_pSK_SK	173
cl1668	ct1795	cn1935	pOP-CNIP00063_EST_C_1_pSK_SK	353
cl1668	ct1795	cn1935	pOP-CNIP00372_EST_C_1_pSK_SK	274
cl1668	ct1795	cn1935	pOP-CNIP00581_EST_C_1_pSK_SK	417
cl1668	ct1795	cn1935	pOP-CNIP00842_EST_C_1_pSK_SK	262
cl1668	ct1795	cn1935	pOP-EAP01274_EST_C_1_pBSK_SK	217
cl1668	ct1795	cn1935	pOP-EAP01476_EST_C_1_pBSK_SK	655
cl1668	ct1795	cn1935	pOP-EAP02729_EST_C_1_pBSK_SK	411
cl1668	ct1795	cn1935	pOP-EAP03731_EST_C_1_pBSK_SK	417

cl1668	ct1795	cn1935	pOP-EO02124_EST_C_1_pSK_SK	421
cl1668	ct1795	cn1935	pOP-EO03257_EST_C_1_pSK_SK	409
cl1668	ct1795	cn1935	pOP-EO05835_EST_C_1_pSK_SK	436
cl1668	ct1795	cn1936	pOP-EO05958_EST_C_1_pSK_SK	569
cl1669	ct1796	cn1937	pOP-EN00880_EST_C_1_pSK_SK	522
cl1669	ct1796	cn1937	pOP-EO02208_EST_C_1_pSK_SK	566
cl1670	ct1797	cn1938	pOP-CNH00842_EST_C_1_pSK_SK	539
cl1670	ct1797	cn1938	pOP-EO02210_EST_C_1_pSK_SK	622
cl1671	ct1798	cn1939	pOP-CBP00078_EST_C_1_pBSK_SK	310
cl1671	ct1798	cn1939	pOP-CEO03575_EST_C_1_pSK_SK	597
cl1671	ct1798	cn1939	pOP-CNHP00181_EST_C_1_pSK_SK	635
cl1671	ct1798	cn1939	pOP-EO02211_EST_C_1_pSK_SK	584
cl1672	ct1799	cn1940	pOP-CEO03353_EST_C_1_pSK_SK	562
cl1672	ct1799	cn1940	pOP-CNH01873_EST_C_1_pSK_SK	455
cl1672	ct1799	cn1940	pOP-CNH04639	665
cl1672	ct1799	cn1940	pOP-EO02216_EST_C_1_pSK_SK	553
cl1673	ct1800	cn1941	pOP-EAP01797_EST_C_1_pBSK_SK	642
cl1673	ct1800	cn1941	pOP-EO02221_EST_C_1_pSK_SK	371
cl1674	ct1801	cn1942	pOP-CAP00042_EST_C_1_pBSK_SK	411
cl1674	ct1801	cn1942	pOP-EO02228_EST_C_1_pSK_SK	494
cl1675	ct1802	cn1943	pOP-EAP01209_EST_C_1_pBSK_SK	525
cl1675	ct1802	cn1943	pOP-EO02237_EST_C_1_pSK_SK	625
cl1676	ct1803	cn1944	pOP-EAP02356_EST_C_1_pBSK_SK	331
cl1676	ct1803	cn1944	pOP-EAP02357_EST_C_1_pBSK_SK	566
cl1676	ct1803	cn1944	pOP-EAP03269_EST_C_1_pBSK_SK	419
cl1676	ct1803	cn1944	pOP-EO02244_EST_C_1_pSK_SK	301
cl1677	ct1804	cn1945	pOP-EAP02214_EST_C_1_pBSK_SK	337
cl1677	ct1804	cn1945	pOP-EO02250_EST_C_1_pSK_SK	300
cl1678	ct1805	cn1946	pOP-EN00824_EST_C_1_pSK_SK	461
cl1678	ct1805	cn1946	pOP-EO02254_EST_C_1_pSK_SK	630
cl1679	ct1806	cn1947	pOP-CAP00398_EST_C_1_pBSK_SK	615
cl1679	ct1806	cn1947	pOP-CBP00007_EST_C_1_pBSK_SK	698
cl1679	ct1806	cn1947	pOP-EN00907_EST_C_1_pSK_SK	500
cl1679	ct1806	cn1947	pOP-EO02265_EST_C_1_pSK_SK	670
cl1680	ct1807	cn1948	pOP-EAP01702_EST_C_1_pBSK_SK	391
cl1680	ct1807	cn1948	pOP-EO02277_EST_C_1_pSK_SK	685
cl1681	ct1808	cn1949	pOP-CNH01199_EST_C_1_pSK_SK	543
cl1681	ct1808	cn1949	pOP-CNH04717_EST_C_1_pSK_SK	524
cl1681	ct1808	cn1949	pOP-CNH04932_EST_C_1_pSK_SK	610
cl1681	ct1808	cn1949	pOP-EO02280_EST_C_1_pSK_SK	621
cl1682	ct1809	cn1950	pOP-CEO02828_EST_C_1_pSK_SK	384
cl1682	ct1809	cn1950	pOP-EAP00552_EST_C_1_pBSK_SK	453
cl1682	ct1809	cn1950	pOP-EAP01992_EST_C_1_pBSK_SK	274
cl1682	ct1809	cn1950	pOP-EO02289_EST_C_1_pSK_SK	575
cl1683	ct1810	cn1951	pOP-CAP00020_EST_C_1_pBSK_SK	662
cl1683	ct1810	cn1951	pOP-EO02305_EST_C_1_pSK_SK	622
cl1684	ct1811	cn1952	pOP-CNIP00078_EST_C_1_pSK_SK	590
cl1684	ct1811	cn1952	pOP-EAP01885_EST_C_1_pBSK_SK	238
cl1684	ct1811	cn1952	pOP-EAP02920_EST_C_1_pBSK_SK	732
cl1684	ct1811	cn1952	pOP-EAP03304_EST_C_1_pBSK_SK	523
cl1684	ct1811	cn1952	pOP-EO02329_EST_C_1_pSK_SK	696
cl1684	ct1811	cn1952	pOP-EO06372_EST_C_1_pSK_SK	675
cl1684	ct1811	cn1953	pOP-EO06423_EST_C_1_pSK_SK	734
cl1685	ct1812	cn1954	pOP-CEO00787_EST_C_1_pSK_SK	352
cl1685	ct1812	cn1954	pOP-CEO02861_EST_C_1_pSK_SK	552
cl1685	ct1812	cn1954	pOP-EAP01181_EST_C_1_pBSK_SK	495

cl1685	ct1812	cn1954	pOP-EAP02755_EST_C_1_pBSK_SK	608
cl1685	ct1812	cn1954	pOP-EO02330_EST_C_1_pSK_SK	685
cl1686	ct1813	cn1955	pOP-CAP00385_EST_C_1_pBSK_SK	623
cl1686	ct1813	cn1955	pOP-CAP00386_EST_C_1_pBSK_SK	396
cl1686	ct1813	cn1955	pOP-EO02340_EST_C_1_pSK_SK	499
cl1686	ct1813	cn1955	pOP-EO03094_EST_C_1_pSK_SK	478
cl1687	ct1814	cn1956	pOP-CEO03341_EST_C_1_pSK_SK	537
cl1687	ct1814	cn1956	pOP-EO02341_EST_C_1_pSK_SK	628
cl1688	ct1815	cn1957	pOP-CBP00099_EST_C_1_pBSK_SK	545
cl1688	ct1815	cn1957	pOP-EO02360_EST_C_1_pSK_SK	694
cl1689	ct1816	cn1958	pOP-EAP01174_EST_C_1_pBSK_SK	261
cl1689	ct1816	cn1958	pOP-EO02375_EST_C_1_pSK_SK	377
cl1689	ct1816	cn1958	pOP-EO06102_EST_C_1_pSK_SK	448
cl1690	ct1817	cn1959	pOP-EN00286_EST_C_1_pSK_SK	552
cl1690	ct1817	cn1959	pOP-EO02326_EST_C_1_pSK_SK	689
cl1690	ct1817	cn1959	pOP-EO02376_EST_C_1_pSK_SK	691
cl1690	ct1817	cn1960	pOP-EO06485_EST_C_1_pSK_SK	713
cl1691	ct1818	cn1961	pOP-CEO01559_EST_C_1_pSK_SK	253
cl1691	ct1818	cn1961	pOP-EO02379_EST_C_1_pSK_SK	611
cl1692	ct1819	cn1962	pOP-CEO02771_EST_C_1_pSK_SK	440
cl1692	ct1819	cn1962	pOP-EO02384_EST_C_1_pSK_SK	473
cl1693	ct1820	cn1963	pOP-CNIP04074_EST_C_1_pSK_SK	209
cl1693	ct1820	cn1963	pOP-EO02385_EST_C_1_pSK_SK	404
cl1694	ct1821	cn1964	pOP-CEO00768_EST_C_1_pSK_SK	372
cl1694	ct1821	cn1964	pOP-CNIP00749_EST_C_1_pSK_SK	359
cl1694	ct1821	cn1964	pOP-EO02401_EST_C_1_pSK_SK	489
cl1695	ct1822	cn1965	pOP-CEO00646_EST_C_1_pSK_SK	106
cl1695	ct1822	cn1965	pOP-CEO02939_EST_C_1_pSK_SK	102
cl1695	ct1822	cn1965	pOP-CNIP00509_EST_C_1_pSK_SK	236
cl1695	ct1822	cn1965	pOP-EO02274_EST_C_1_pSK_SK	562
cl1695	ct1822	cn1965	pOP-EO02403_EST_C_1_pSK_SK	571
cl1696	ct1823	cn1966	pOP-CEO03295_EST_C_1_pSK_SK	625
cl1696	ct1823	cn1966	pOP-CEO03303_EST_C_1_pSK_SK	383
cl1696	ct1823	cn1966	pOP-EO02404_EST_C_1_pSK_SK	568
cl1697	ct1824	cn1967	pOP-CNIP01025_EST_C_1_pSK_SK	662
cl1697	ct1824	cn1967	pOP-EO02407_EST_C_1_pSK_SK	480
cl1698	ct1825	cn1968	pOP-CNH02313_EST_C_1_pSK_SK	675
cl1698	ct1825	cn1968	pOP-EO02415_EST_C_1_pSK_SK	468
cl1698	ct1825	cn1968	pOP-EO06184_EST_C_1_pSK_SK	614
cl1699	ct1826	cn1969	pOP-CEO02884_EST_C_1_pSK_SK	547
cl1699	ct1826	cn1969	pOP-EO02422_EST_C_1_pSK_SK	461
cl1700	ct1827	cn1970	pOP-CEO03766_EST_C_1_pSK_SK	275
cl1700	ct1827	cn1970	pOP-CNI01309_EST_C_1_pSK_SK	499
cl1700	ct1827	cn1971	pOP-CEM00104_EST_C_1_pSK_SK	228
cl1700	ct1827	cn1971	pOP-CNIP00900_EST_C_1_pSK_SK	248
cl1700	ct1827	cn1971	pOP-EAP01483_EST_C_1_pBSK_SK	507
cl1700	ct1827	cn1971	pOP-EAP02912_EST_C_1_pBSK_SK	340
cl1700	ct1827	cn1971	pOP-EO02423_EST_C_1_pSK_SK	314
cl1701	ct1828	cn1972	pOP-EAP03627_EST_C_1_pBSK_SK	224
cl1701	ct1828	cn1972	pOP-EAP03677_EST_C_1_pBSK_SK	277
cl1701	ct1828	cn1972	pOP-EO02440_EST_C_1_pSK_SK	461
cl1702	ct1829	cn1973	pOP-EAP03632_EST_C_1_pBSK_SK	517
cl1702	ct1829	cn1973	pOP-EO05512_EST_C_1_pSK_SK	394
cl1702	ct1830	cn1974	pOP-EN00384_EST_C_1_pSK_SK	555
cl1702	ct1830	cn1975	pOP-EO02444_EST_C_1_pSK_SK	463
cl1703	ct1831	cn1976	pOP-CNI02093_EST_C_1_pSK_SK	508

cl1703	ct1831	cn1976	pOP-EO02478_EST_C_1_pSK_SK	470
cl1704	ct1832	cn1977	pOP-CEO02562_EST_C_1_pSK_SK	541
cl1704	ct1832	cn1977	pOP-EO02481_EST_C_1_pSK_SK	520
cl1705	ct1833	cn1978	pOP-CBP00120_EST_C_1_pBSK_SK	650
cl1705	ct1833	cn1978	pOP-CEO01697_EST_C_1_pSK_SK	388
cl1705	ct1833	cn1978	pOP-CNI01594_EST_C_1_pSK_SK	480
cl1705	ct1833	cn1978	pOP-EAP00511_EST_C_1_pBSK_SK	671
cl1705	ct1833	cn1978	pOP-EAP00602_EST_C_1_pBSK_SK	512
cl1705	ct1833	cn1978	pOP-EO02483_EST_C_1_pSK_SK	523
cl1705	ct1833	cn1978	pOP-EO05035_EST_C_1_pSK_SK	473
cl1706	ct1834	cn1979	pOP-EO02412_EST_C_1_pSK_SK	255
cl1706	ct1834	cn1979	pOP-EO02490_EST_C_1_pSK_SK	255
cl1707	ct1835	cn1980	pOP-CEO03356_EST_C_1_pSK_SK	415
cl1707	ct1835	cn1980	pOP-EO02495_EST_C_1_pSK_SK	528
cl1708	ct1836	cn1981	pOP-CEO02933_EST_C_1_pSK_SK	281
cl1708	ct1836	cn1981	pOP-EO02507_EST_C_1_pSK_SK	529
cl1709	ct1837	cn1982	pOP-CEO02561_EST_C_1_pSK_SK	478
cl1709	ct1837	cn1982	pOP-EAP00411_EST_C_1_pBSK_SK	207
cl1709	ct1837	cn1982	pOP-EAP01276_EST_C_1_pBSK_SK	233
cl1709	ct1837	cn1982	pOP-EO02511_EST_C_1_pSK_SK	527
cl1709	ct1838	cn1983	pOP-CEM00149_EST_C_1_pSK_SK	307
cl1709	ct1838	cn1983	pOP-CEO02247_EST_C_1_pSK_SK	317
cl1709	ct1838	cn1983	pOP-EAP01245_EST_C_1_pBSK_SK	370
cl1709	ct1838	cn1983	pOP-EAP01677_EST_C_1_pBSK_SK	472
cl1709	ct1838	cn1983	pOP-EAP01884_EST_C_1_pBSK_SK	373
cl1709	ct1838	cn1983	pOP-EAP02230_EST_C_1_pBSK_SK	174
cl1709	ct1838	cn1983	pOP-EAP02323_EST_C_1_pBSK_SK	552
cl1709	ct1838	cn1983	pOP-EO06587_EST_C_1_pSK_SK	469
cl1710	ct1839	cn1984	pOP-CNIP00071_EST_C_1_pSK_SK	250
cl1710	ct1839	cn1984	pOP-EO02520_EST_C_1_pSK_SK	531
cl1711	ct1840	cn1985	pOP-CEO01487_EST_C_1_pSK_SK	413
cl1711	ct1840	cn1985	pOP-CEO03197_EST_C_1_pSK_SK	484
cl1711	ct1840	cn1985	pOP-EO02521_EST_C_1_pSK_SK	525
cl1712	ct1841	cn1986	pOP-CNIP00601_EST_C_1_pSK_SK	417
cl1712	ct1841	cn1986	pOP-EAP01552_EST_C_1_pBSK_SK	599
cl1712	ct1841	cn1986	pOP-EO02532_EST_C_1_pSK_SK	529
cl1713	ct1842	cn1987	pOP-EO02026_EST_C_1_pSK_SK	477
cl1713	ct1842	cn1987	pOP-EO02299_EST_C_1_pSK_SK	536
cl1713	ct1842	cn1987	pOP-EO02536_EST_C_1_pSK_SK	530
cl1714	ct1843	cn1988	pOP-EO02267_EST_C_1_pSK_SK	669
cl1714	ct1843	cn1988	pOP-EO02542_EST_C_1_pSK_SK	528
cl1715	ct1844	cn1989	pOP-CNIP00111_EST_C_1_pSK_SK	674
cl1715	ct1844	cn1989	pOP-EO02570_EST_C_1_pSK_SK	479
cl1716	ct1845	cn1990	pOP-CNH01431_EST_C_1_pSK_SK	413
cl1716	ct1845	cn1990	pOP-EAP00064_EST_C_1_pBSK_SK	527
cl1716	ct1845	cn1990	pOP-EAP00065_EST_C_1_pBSK_SK	512
cl1716	ct1845	cn1990	pOP-EO02573_EST_C_1_pSK_SK	480
cl1717	ct1846	cn1991	pOP-CEO01153_EST_C_1_pSK_SK	462
cl1717	ct1846	cn1991	pOP-CEO01847_EST_C_1_pSK_SK	498
cl1717	ct1846	cn1991	pOP-EO02574_EST_C_1_pSK_SK	479
cl1718	ct1847	cn1992	pOP-CBP00176_EST_C_1_pBSK_SK	502
cl1718	ct1847	cn1992	pOP-CEO02934_EST_C_1_pSK_SK	499
cl1718	ct1847	cn1992	pOP-CNH00598_EST_C_1_pSK_SK	667
cl1718	ct1847	cn1992	pOP-EAP00842_EST_C_1_pBSK_SK	392
cl1718	ct1847	cn1992	pOP-EAP00925_EST_C_1_pBSK_SK	583
cl1718	ct1847	cn1992	pOP-EAP02116_EST_C_1_pBSK_SK	369

cl1718	ct1847	cn1992	pOP-EAP02907_EST_C_1_pBSK_SK	297
cl1718	ct1847	cn1992	pOP-EO02245_EST_C_1_pSK_SK	606
cl1718	ct1847	cn1992	pOP-EO02576_EST_C_1_pSK_SK	476
cl1719	ct1848	cn1993	pOP-CAP00247_EST_C_1_pBSK_SK	483
cl1719	ct1848	cn1993	pOP-EO02578_EST_C_1_pSK_SK	479
cl1720	ct1849	cn1994	pOP-CEO02148_EST_C_1_pSK_SK	524
cl1720	ct1849	cn1994	pOP-EO02580_EST_C_1_pSK_SK	473
cl1721	ct1850	cn1995	pOP-CEO02238_EST_C_1_pSK_SK	340
cl1721	ct1850	cn1995	pOP-EO02590_EST_C_1_pSK_SK	473
cl1722	ct1851	cn1996	pOP-EO02203_EST_C_1_pSK_SK	457
cl1722	ct1851	cn1996	pOP-EO02594_EST_C_1_pSK_SK	372
cl1723	ct1852	cn1997	pOP-CEM00082_EST_C_1_pSK_SK	317
cl1723	ct1852	cn1997	pOP-EAP03614_EST_C_1_pBSK_SK	268
cl1723	ct1852	cn1997	pOP-EO02308_EST_C_1_pSK_SK	541
cl1723	ct1852	cn1997	pOP-EO02599_EST_C_1_pSK_SK	376
cl1723	ct1852	cn1997	pOP-EO03524_EST_C_1_pSK_SK	542
cl1723	ct1852	cn1997	pOP-EO04593_EST_C_1_pSK_SK	449
cl1723	ct1853	cn1998	pOP-CEO02300_EST_C_1_pSK_SK	560
cl1723	ct1853	cn1998	pOP-CNI01166_EST_C_1_pSK_SK	388
cl1723	ct1853	cn1998	pOP-EAP01043_EST_C_1_pBSK_SK	264
cl1723	ct1853	cn1998	pOP-EAP01455_EST_C_1_pBSK_SK	266
cl1723	ct1853	cn1998	pOP-EO02110_EST_C_1_pSK_SK	455
cl1723	ct1853	cn1998	pOP-EO03949_EST_C_1_pSK_SK	495
cl1723	ct1853	cn1998	pOP-EOP00018_EST_C_1_pSK_SK	643
cl1724	ct1854	cn1999	pOP-EN00575_EST_C_1_pSK_SK	370
cl1724	ct1854	cn1999	pOP-EO02605_EST_C_1_pSK_SK	332
cl1725	ct1855	cn2000	pOP-CNI02224_EST_C_1_pSK_SK	669
cl1725	ct1855	cn2000	pOP-EAP01438_EST_C_1_pBSK_SK	384
cl1725	ct1855	cn2001	pOP-EAP00675_EST_C_1_pBSK_SK	621
cl1725	ct1856	cn2002	pOP-CBP00211_EST_C_1_pBSK_SK	182
cl1725	ct1856	cn2002	pOP-CEO00885_EST_C_1_pSK_SK	422
cl1725	ct1856	cn2002	pOP-CNH00863_EST_C_1_pSK_SK	697
cl1725	ct1856	cn2002	pOP-CNHP00303_EST_C_1_pSK_SK	631
cl1725	ct1856	cn2002	pOP-CNHP00480_EST_C_1_pSK_SK	613
cl1725	ct1856	cn2002	pOP-CNIP00185_EST_C_1_pSK_SK	406
cl1725	ct1856	cn2002	pOP-CNLP00022_EST_C_1_pSK_SK	328
cl1725	ct1856	cn2002	pOP-EO02606_EST_C_1_pSK_SK	371
cl1725	ct1856	cn2002	pOP-EO06448_EST_C_1_pSK_SK	648
cl1726	ct1857	cn2003	pOP-CAP00034_EST_C_1_pBSK_SK	389
cl1726	ct1857	cn2003	pOP-CEO02541_EST_C_1_pSK_SK	199
cl1726	ct1857	cn2003	pOP-CNH02267_EST_C_1_pSK_SK	548
cl1726	ct1857	cn2003	pOP-CNH03534_EST_C_1_pSK_SK	334
cl1726	ct1857	cn2003	pOP-EO02610_EST_C_1_pSK_SK	374
cl1727	ct1858	cn2004	pOP-EN00124_EST_C_1_pSK_SK	611
cl1727	ct1858	cn2004	pOP-EO02630_EST_C_1_pSK_SK	460
cl1728	ct1859	cn2005	pOP-CNH00753_EST_C_1_pSK_SK	502
cl1728	ct1859	cn2005	pOP-CNH00825_EST_C_1_pSK_SK	477
cl1728	ct1859	cn2005	pOP-CNHP00513_EST_C_1_pSK_SK	524
cl1728	ct1859	cn2005	pOP-EO02654_EST_C_1_pSK_SK	455
cl1729	ct1860	cn2006	pOP-CNH01560_EST_C_1_pSK_SK	597
cl1729	ct1860	cn2006	pOP-CNH01998_EST_C_1_pSK_SK	479
cl1729	ct1860	cn2006	pOP-EO02678_EST_C_1_pSK_SK	447
cl1730	ct1861	cn2007	pOP-CNHP00268_EST_C_1_pSK_SK	315
cl1730	ct1861	cn2007	pOP-CNI02069_EST_C_1_pSK_SK	672
cl1730	ct1861	cn2008	pOP-CEO03244_EST_C_1_pSK_SK	510
cl1730	ct1862	cn2009	pOP-CAP00320_EST_C_1_pBSK_SK	645

cl1730	ct1862	cn2009	pOP-EO02721_EST_C_1_pSK_SK	451
cl1730	ct1862	cn2009	pOP-EO07304_EST_C_1_pSK_SK	797
cl1731	ct1863	cn2010	pOP-CEO03500_EST_C_1_pSK_SK	368
cl1731	ct1863	cn2010	pOP-EO02722_EST_C_1_pSK_SK	249
cl1732	ct1864	cn2011	pOP-CEO01186_EST_C_1_pSK_SK	340
cl1732	ct1864	cn2011	pOP-CNI01839_EST_C_1_pSK_SK	287
cl1732	ct1864	cn2011	pOP-CNI01989_EST_C_1_pSK_SK	186
cl1732	ct1864	cn2011	pOP-EO02728_EST_C_1_pSK_SK	426
cl1733	ct1865	cn2012	pOP-CNH02760_EST_C_1_pSK_SK	527
cl1733	ct1865	cn2012	pOP-CNH04267	526
cl1733	ct1865	cn2012	pOP-CNIP00775_EST_C_1_pSK_SK	585
cl1733	ct1866	cn2013	pOP-EAP01501_EST_C_1_pBSK_SK	331
cl1733	ct1866	cn2013	pOP-EAP02052_EST_C_1_pBSK_SK	392
cl1733	ct1866	cn2013	pOP-EO02744_EST_C_1_pSK_SK	448
cl1733	ct1866	cn2013	pOP-EO03827_EST_C_1_pSK_SK	527
cl1733	ct1866	cn2013	pOP-EO05200_EST_C_1_pSK_SK	531
cl1733	ct1866	cn2013	pOP-EO05747_EST_C_1_pSK_SK	558
cl1734	ct1867	cn2014	pOP-CEM00110_EST_C_1_pSK_SK	286
cl1734	ct1867	cn2014	pOP-CEM00111_EST_C_1_pSK_SK	322
cl1734	ct1867	cn2014	pOP-EO02746_EST_C_1_pSK_SK	448
cl1735	ct1868	cn2015	pOP-CAP00341_EST_C_1_pBSK_SK	297
cl1735	ct1868	cn2015	pOP-CAP00414_EST_C_1_pBSK_SK	578
cl1735	ct1868	cn2015	pOP-EO02753_EST_C_1_pSK_SK	440
cl1736	ct1869	cn2016	pOP-CNI01195_EST_C_1_pSK_SK	431
cl1736	ct1869	cn2016	pOP-CNI01887_EST_C_1_pSK_SK	432
cl1736	ct1870	cn2017	pOP-CEO01871_EST_C_1_pSK_SK	449
cl1736	ct1870	cn2017	pOP-EO05218_EST_C_1_pSK_SK	490
cl1736	ct1871	cn2018	pOP-CNI01162_EST_C_1_pSK_SK	569
cl1736	ct1871	cn2019	pOP-CNH01624_EST_C_1_pSK_SK	424
cl1736	ct1871	cn2019	pOP-CNH01906_EST_C_1_pSK_SK	430
cl1736	ct1871	cn2019	pOP-CNH02246_EST_C_1_pSK_SK	433
cl1736	ct1871	cn2019	pOP-CNH03501_EST_C_1_pSK_SK	341
cl1736	ct1871	cn2019	pOP-EO02757_EST_C_1_pSK_SK	449
cl1736	ct1872	cn2020	pOP-CBP00134_EST_C_1_pBSK_SK	366
cl1736	ct1872	cn2020	pOP-CEO01176_EST_C_1_pSK_SK	356
cl1736	ct1872	cn2020	pOP-CEO01317	362
cl1736	ct1872	cn2020	pOP-CEO01454_EST_C_1_pSK_SK	227
cl1736	ct1872	cn2020	pOP-CEO03285_EST_C_1_pSK_SK	307
cl1736	ct1872	cn2020	pOP-EAP01357_EST_C_1_pBSK_SK	296
cl1736	ct1872	cn2020	pOP-EO06417_EST_C_1_pSK_SK	371
cl1736	ct1872	cn2020	pOP-EO07098_EST_C_1_pSK_SK	375
cl1736	ct1872	cn2020	pOP-EO07462_EST_C_1_pSK_SK	349
cl1737	ct1873	cn2021	pOP-CEO03765_EST_C_1_pSK_SK	326
cl1737	ct1873	cn2021	pOP-CNI01192_EST_C_1_pSK_SK	304
cl1737	ct1873	cn2021	pOP-EO02760_EST_C_1_pSK_SK	444
cl1737	ct1873	cn2021	pOP-EO08016_EST_C_1_pSK_SK	382
cl1738	ct1874	cn2022	pOP-CNI01658_EST_C_1_pSK_SK	433
cl1738	ct1874	cn2022	pOP-EO02770_EST_C_1_pSK_SK	378
cl1738	ct1874	cn2023	pOP-CNIP00161_EST_C_1_pSK_SK	369
cl1739	ct1875	cn2024	pOP-CEO02054_EST_C_1_pSK_SK	207
cl1739	ct1875	cn2024	pOP-CEO03365_EST_C_1_pSK_SK	141
cl1739	ct1875	cn2024	pOP-CEO03791_EST_C_1_pSK_SK	116
cl1739	ct1875	cn2024	pOP-EO02774_EST_C_1_pSK_SK	323
cl1740	ct1876	cn2025	pOP-EO06992_EST_C_1_pSK_SK	720
cl1740	ct1876	cn2026	pOP-EAP00743_EST_C_1_pBSK_SK	414
cl1740	ct1876	cn2026	pOP-EAP03120_EST_C_1_pBSK_SK	500

cl1740	ct1876	cn2026	pOP-EO02794_EST_C_1_pSK_SK	438
cl1740	ct1876	cn2027	pOP-EAP02018_EST_C_1_pBSK_SK	443
cl1741	ct1877	cn2028	pOP-EO02780_EST_C_1_pSK_SK	284
cl1741	ct1877	cn2028	pOP-EO02796_EST_C_1_pSK_SK	332
cl1742	ct1878	cn2029	pOP-CNIP00345_EST_C_1_pSK_SK	518
cl1742	ct1878	cn2029	pOP-EAP00704_EST_C_1_pBSK_SK	574
cl1742	ct1878	cn2029	pOP-EAP02364_EST_C_1_pBSK_SK	485
cl1742	ct1878	cn2029	pOP-EO02810_EST_C_1_pSK_SK	424
cl1743	ct1879	cn2030	pOP-EAP00834_EST_C_1_pBSK_SK	434
cl1743	ct1879	cn2030	pOP-EAP00835_EST_C_1_pBSK_SK	249
cl1743	ct1879	cn2030	pOP-EAP03394_EST_C_1_pBSK_SK	420
cl1743	ct1879	cn2030	pOP-EO02813_EST_C_1_pSK_SK	402
cl1744	ct1880	cn2031	pOP-EAP03482_EST_C_1_pBSK_SK	205
cl1744	ct1880	cn2031	pOP-EO02829_EST_C_1_pSK_SK	410
cl1745	ct1881	cn2032	pOP-CEO02017_EST_C_1_pSK_SK	348
cl1745	ct1881	cn2032	pOP-CNH02708_EST_C_1_pSK_SK	544
cl1745	ct1882	cn2033	pOP-CEO01320	160
cl1745	ct1882	cn2033	pOP-CEO01493_EST_C_1_pSK_SK	353
cl1745	ct1882	cn2033	pOP-CEO02482_EST_C_1_pSK_SK	364
cl1745	ct1882	cn2033	pOP-CEO03339_EST_C_1_pSK_SK	301
cl1745	ct1882	cn2033	pOP-CEO03728_EST_C_1_pSK_SK	382
cl1745	ct1882	cn2033	pOP-CNH03254_EST_C_1_pSK_SK	444
cl1745	ct1882	cn2033	pOP-CNIP00632_EST_C_1_pSK_SK	594
cl1745	ct1882	cn2033	pOP-CNIP04077_EST_C_1_pSK_SK	621
cl1745	ct1882	cn2033	pOP-EO02831_EST_C_1_pSK_SK	372
cl1745	ct1882	cn2033	pOP-EO05925_EST_C_1_pSK_SK	524
cl1745	ct1882	cn2034	pOP-CEO02003_EST_C_1_pSK_SK	338
cl1746	ct1883	cn2035	pOP-CNIP04058_EST_C_1_pSK_SK	649
cl1746	ct1883	cn2035	pOP-EO02852_EST_C_1_pSK_SK	461
cl1747	ct1884	cn2036	pOP-EAP01372_EST_C_1_pBSK_SK	331
cl1747	ct1884	cn2036	pOP-EO02856_EST_C_1_pSK_SK	468
cl1747	ct1884	cn2036	pOP-EO04665_EST_C_1_pSK_SK	489
cl1748	ct1885	cn2037	pOP-CNH00765_EST_C_1_pSK_SK	488
cl1748	ct1885	cn2037	pOP-EO02857_EST_C_1_pSK_SK	358
cl1749	ct1886	cn2038	pOP-CEO02630_EST_C_1_pSK_SK	500
cl1749	ct1886	cn2038	pOP-EAP03330_EST_C_1_pBSK_SK	468
cl1749	ct1886	cn2038	pOP-EO02862_EST_C_1_pSK_SK	335
cl1749	ct1886	cn2038	pOP-EO06367_EST_C_1_pSK_SK	643
cl1749	ct1887	cn2039	pOP-CNIP00605_EST_C_1_pSK_SK	567
cl1749	ct1887	cn2039	pOP-EAP01355_EST_C_1_pBSK_SK	174
cl1749	ct1887	cn2039	pOP-EO02615_EST_C_1_pSK_SK	371
cl1749	ct1887	cn2039	pOP-EO02906_EST_C_1_pSK_SK	330
cl1749	ct1887	cn2039	pOP-EO05209_EST_C_1_pSK_SK	482
cl1749	ct1887	cn2040	pOP-CEO00520_EST_C_1_pSK_SK	391
cl1750	ct1888	cn2041	pOP-EO02710_EST_C_1_pSK_SK	447
cl1750	ct1888	cn2041	pOP-EO02866_EST_C_1_pSK_SK	293
cl1751	ct1889	cn2042	pOP-CEO02730_EST_C_1_pSK_SK	449
cl1751	ct1889	cn2042	pOP-CNH03014_EST_C_1_pSK_SK	562
cl1751	ct1889	cn2042	pOP-EO02870_EST_C_1_pSK_SK	371
cl1751	ct1889	cn2042	pOP-EO06557_EST_C_1_pSK_SK	777
cl1751	ct1889	cn2042	pOP-EO06985_EST_C_1_pSK_SK	770
cl1752	ct1890	cn2043	pOP-CEO00650_EST_C_1_pSK_SK	213
cl1752	ct1890	cn2043	pOP-CNHP00211_EST_C_1_pSK_SK	492
cl1752	ct1890	cn2043	pOP-CNI01340_EST_C_1_pSK_SK	382
cl1752	ct1890	cn2043	pOP-CNI01616_EST_C_1_pSK_SK	517
cl1752	ct1890	cn2043	pOP-CNI01803_EST_C_1_pSK_SK	432

cl1752	ct1890	cn2043	pOP-CNIP00737_EST_C_1_pSK_SK	607
cl1752	ct1890	cn2043	pOP-EAP00655_EST_C_1_pBSK_SK	610
cl1752	ct1890	cn2043	pOP-EAP02329_EST_C_1_pBSK_SK	566
cl1752	ct1890	cn2043	pOP-EAP02918_EST_C_1_pBSK_SK	602
cl1752	ct1890	cn2043	pOP-EO02871_EST_C_1_pSK_SK	456
cl1753	ct1891	cn2044	pOP-CEM00114_EST_C_1_pSK_SK	322
cl1753	ct1891	cn2044	pOP-EO02874_EST_C_1_pSK_SK	285
cl1754	ct1892	cn2045	pOP-EAP03227_EST_C_1_pBSK_SK	416
cl1754	ct1892	cn2045	pOP-EO02878_EST_C_1_pSK_SK	421
cl1755	ct1893	cn2046	pOP-CEO02126_EST_C_1_pSK_SK	431
cl1755	ct1893	cn2046	pOP-EAP01409_EST_C_1_pBSK_SK	284
cl1755	ct1894	cn2047	pOP-CAP00308_EST_C_1_pBSK_SK	646
cl1755	ct1894	cn2047	pOP-CNI01334_EST_C_1_pSK_SK	370
cl1755	ct1894	cn2047	pOP-EAP02296_EST_C_1_pBSK_SK	413
cl1755	ct1894	cn2047	pOP-EAP02788_EST_C_1_pBSK_SK	377
cl1755	ct1894	cn2047	pOP-EO02882_EST_C_1_pSK_SK	449
cl1756	ct1895	cn2048	pOP-CNIP00119_EST_C_1_pSK_SK	379
cl1756	ct1895	cn2048	pOP-EAP03192_EST_C_1_pBSK_SK	514
cl1756	ct1895	cn2048	pOP-EAP03398_EST_C_1_pBSK_SK	408
cl1756	ct1895	cn2048	pOP-EAP03624_EST_C_1_pBSK_SK	330
cl1756	ct1895	cn2048	pOP-EO02672_EST_C_1_pSK_SK	459
cl1756	ct1895	cn2048	pOP-EO02884_EST_C_1_pSK_SK	442
cl1757	ct1896	cn2049	pOP-EN00416_EST_C_1_pSK_SK	518
cl1757	ct1896	cn2049	pOP-EO02885_EST_C_1_pSK_SK	437
cl1758	ct1897	cn2050	pOP-EO02104_EST_C_1_pSK_SK	649
cl1758	ct1897	cn2050	pOP-EO02168_EST_C_1_pSK_SK	540
cl1758	ct1897	cn2050	pOP-EO02886_EST_C_1_pSK_SK	442
cl1759	ct1898	cn2051	pOP-CEM00075_EST_C_1_pSK_SK	109
cl1759	ct1898	cn2051	pOP-CEM00128_EST_C_1_pSK_SK	478
cl1759	ct1898	cn2051	pOP-CEO02955_EST_C_1_pSK_SK	274
cl1759	ct1898	cn2051	pOP-CNH03615_EST_C_1_pSK_SK	626
cl1759	ct1898	cn2051	pOP-EAP01593_EST_C_1_pBSK_SK	421
cl1759	ct1898	cn2051	pOP-EO02533_EST_C_1_pSK_SK	515
cl1759	ct1898	cn2051	pOP-EO02889_EST_C_1_pSK_SK	409
cl1760	ct1899	cn2052	pOP-CEM00046_EST_C_1_pSK_SK	146
cl1760	ct1899	cn2052	pOP-CEO03092_EST_C_1_pSK_SK	361
cl1760	ct1899	cn2052	pOP-CNIP00472_EST_C_1_pSK_SK	385
cl1760	ct1899	cn2052	pOP-EAP00077_EST_C_1_pBSK_SK	513
cl1760	ct1899	cn2052	pOP-EO02724_EST_C_1_pSK_SK	363
cl1760	ct1899	cn2052	pOP-EO02902_EST_C_1_pSK_SK	366
cl1760	ct1899	cn2052	pOP-EO04373_EST_C_1_pSK_SK	446
cl1760	ct1899	cn2052	pOP-EO04755_EST_C_1_pSK_SK	525
cl1760	ct1899	cn2052	pOP-EO05633_EST_C_1_pSK_SK	485
cl1760	ct1899	cn2052	pOP-EO07113_EST_C_1_pSK_SK	677
cl1760	ct1899	cn2052	pOP-EO08080_EST_C_1_pSK_SK	442
cl1760	ct1899	cn2053	pOP-EO05927_EST_C_1_pSK_SK	548
cl1761	ct1900	cn2054	pOP-CAP00326_EST_C_1_pBSK_SK	343
cl1761	ct1900	cn2054	pOP-CNI02150_EST_C_1_pSK_SK	323
cl1761	ct1900	cn2054	pOP-EO02913_EST_C_1_pSK_SK	274
cl1762	ct1901	cn2055	pOP-CEO03083_EST_C_1_pSK_SK	291
cl1762	ct1901	cn2055	pOP-EO02067_EST_C_1_pSK_SK	601
cl1762	ct1901	cn2055	pOP-EO02917_EST_C_1_pSK_SK	453
cl1762	ct1901	cn2055	pOP-EO04072_EST_C_1_pSK_SK	534
cl1763	ct1902	cn2056	pOP-CAP00400_EST_C_1_pBSK_SK	637
cl1763	ct1902	cn2056	pOP-CEO01057_EST_C_1_pSK_SK	237
cl1763	ct1902	cn2056	pOP-EAP02312_EST_C_1_pBSK_SK	762

cl1763	ct1902	cn2056	pOP-EO02918_EST_C_1_pSK_SK	447
cl1763	ct1902	cn2056	pOP-EO04362_EST_C_1_pSK_SK	419
cl1764	ct1903	cn2057	pOP-EO02243_EST_C_1_pSK_SK	629
cl1764	ct1903	cn2057	pOP-EO02937_EST_C_1_pSK_SK	445
cl1764	ct1903	cn2058	pOP-EO02669_EST_C_1_pSK_SK	457
cl1764	ct1903	cn2059	pOP-EO02117_EST_C_1_pSK_SK	244
cl1765	ct1904	cn2060	pOP-EAP03421_EST_C_1_pBSK_SK	533
cl1765	ct1904	cn2060	pOP-EO02224_EST_C_1_pSK_SK	626
cl1765	ct1904	cn2060	pOP-EO02957_EST_C_1_pSK_SK	327
cl1766	ct1905	cn2061	pOP-EO02010_EST_C_1_pSK_SK	609
cl1766	ct1905	cn2061	pOP-EO02958_EST_C_1_pSK_SK	354
cl1766	ct1905	cn2062	pOP-EN00794_EST_C_1_pSK_SK	518
cl1766	ct1905	cn2063	pOP-EO02643_EST_C_1_pSK_SK	383
cl1767	ct1906	cn2064	pOP-CNI01909_EST_C_1_pSK_SK	131
cl1767	ct1906	cn2064	pOP-EO02965_EST_C_1_pSK_SK	348
cl1768	ct1907	cn2065	pOP-CAP00302_EST_C_1_pBSK_SK	405
cl1768	ct1907	cn2065	pOP-EO02973_EST_C_1_pSK_SK	378
cl1769	ct1908	cn2066	pOP-CEOP00009_EST_C_1_pSK_SK	442
cl1769	ct1908	cn2067	pOP-EO02994_EST_C_1_pSK_SK	236
cl1770	ct1909	cn2068	pOP-CAP00092_EST_C_1_pBSK_SK	640
cl1770	ct1909	cn2068	pOP-EO03001_EST_C_1_pSK_SK	367
cl1771	ct1910	cn2069	pOP-EO03007_EST_C_1_pSK_SK	314
cl1771	ct1910	cn2069	pOP-EO03090_EST_C_1_pSK_SK	441
cl1771	ct1910	cn2069	pOP-EO05176_EST_C_1_pSK_SK	414
cl1771	ct1910	cn2069	pOP-EO05438_EST_C_1_pSK_SK	427
cl1772	ct1911	cn2070	pOP-EN00614_EST_C_1_pSK_SK	318
cl1772	ct1911	cn2070	pOP-ENP00014_EST_C_1_pSK_SK	234
cl1772	ct1911	cn2070	pOP-EO02468_EST_C_1_pSK_SK	333
cl1772	ct1911	cn2070	pOP-EO03010_EST_C_1_pSK_SK	118
cl1772	ct1911	cn2070	pOP-EO03837_EST_C_1_pSK_SK	523
cl1772	ct1912	cn2071	pOP-CAP00219_EST_C_1_pBSK_SK	592
cl1772	ct1912	cn2071	pOP-CEO01433_EST_C_1_pSK_SK	299
cl1772	ct1912	cn2071	pOP-CNI01400_EST_C_1_pSK_SK	143
cl1772	ct1912	cn2071	pOP-CNIP00237_EST_C_1_pSK_SK	347
cl1772	ct1912	cn2071	pOP-EAP02936_EST_C_1_pBSK_SK	670
cl1772	ct1912	cn2071	pOP-EO02037_EST_C_1_pSK_SK	661
cl1772	ct1912	cn2071	pOP-EO03171_EST_C_1_pSK_SK	456
cl1772	ct1912	cn2071	pOP-EO03236_EST_C_1_pSK_SK	446
cl1772	ct1912	cn2071	pOP-EO04502_EST_C_1_pSK_SK	523
cl1772	ct1912	cn2071	pOP-EO04979_EST_C_1_pSK_SK	328
cl1772	ct1912	cn2071	pOP-EO04980_EST_C_1_pSK_SK	511
cl1772	ct1912	cn2071	pOP-EO06117_EST_C_1_pSK_SK	583
cl1773	ct1913	cn2072	pOP-EN00306_EST_C_1_pSK_SK	549
cl1773	ct1913	cn2072	pOP-EO02980_EST_C_1_pSK_SK	454
cl1773	ct1914	cn2073	pOP-EN00900_EST_C_1_pSK_SK	538
cl1773	ct1914	cn2073	pOP-EO03289_EST_C_1_pSK_SK	377
cl1773	ct1915	cn2074	pOP-CAP00206_EST_C_1_pBSK_SK	513
cl1773	ct1915	cn2074	pOP-CAP00217_EST_C_1_pBSK_SK	631
cl1773	ct1915	cn2074	pOP-EN00368_EST_C_1_pSK_SK	460
cl1773	ct1915	cn2074	pOP-EN00910_EST_C_1_pSK_SK	538
cl1773	ct1915	cn2074	pOP-EO02735_EST_C_1_pSK_SK	447
cl1773	ct1915	cn2074	pOP-EO03021_EST_C_1_pSK_SK	484
cl1774	ct1916	cn2075	pOP-EO02227_EST_C_1_pSK_SK	668
cl1774	ct1916	cn2075	pOP-EO03045_EST_C_1_pSK_SK	248
cl1774	ct1916	cn2076	pOP-EO03053_EST_C_1_pSK_SK	474
cl1775	ct1917	cn2077	pOP-EO03056_EST_C_1_pSK_SK	318

cl1775	ct1917	cn2077	pOP-EO03062_EST_C_1_pSK_SK	341
cl1776	ct1918	cn2078	pOP-CEO01315	363
cl1776	ct1918	cn2078	pOP-EO03082_EST_C_1_pSK_SK	315
cl1777	ct1919	cn2079	pOP-EO03047_EST_C_1_pSK_SK	301
cl1777	ct1919	cn2079	pOP-EO03054_EST_C_1_pSK_SK	264
cl1777	ct1919	cn2079	pOP-EO03086_EST_C_1_pSK_SK	471
cl1778	ct1920	cn2080	pOP-CAP00039_EST_C_1_pBSK_SK	407
cl1778	ct1920	cn2080	pOP-EO03091_EST_C_1_pSK_SK	480
cl1779	ct1921	cn2081	pOP-CNH04258	578
cl1779	ct1921	cn2081	pOP-EO03098_EST_C_1_pSK_SK	423
cl1780	ct1922	cn2082	pOP-EN00377_EST_C_1_pSK_SK	547
cl1780	ct1922	cn2082	pOP-EO03115_EST_C_1_pSK_SK	390
cl1781	ct1923	cn2083	pOP-EAP03453_EST_C_1_pBSK_SK	255
cl1781	ct1923	cn2083	pOP-EO03128_EST_C_1_pSK_SK	302
cl1782	ct1924	cn2084	pOP-CNH02775_EST_C_1_pSK_SK	625
cl1782	ct1924	cn2084	pOP-CNH03315_EST_C_1_pSK_SK	714
cl1782	ct1924	cn2084	pOP-EO03170_EST_C_1_pSK_SK	466
cl1783	ct1925	cn2085	pOP-CNH00705_EST_C_1_pSK_SK	654
cl1783	ct1925	cn2085	pOP-EO03193_EST_C_1_pSK_SK	417
cl1784	ct1926	cn2086	pOP-CEO02130_EST_C_1_pSK_SK	618
cl1784	ct1926	cn2086	pOP-CEO03667_EST_C_1_pSK_SK	463
cl1784	ct1926	cn2086	pOP-CNHP00460_EST_C_1_pSK_SK	457
cl1784	ct1926	cn2086	pOP-EO03194_EST_C_1_pSK_SK	469
cl1785	ct1927	cn2087	pOP-CNHP00272_EST_C_1_pSK_SK	685
cl1785	ct1927	cn2087	pOP-CNI01083_EST_C_1_pSK_SK	499
cl1785	ct1927	cn2087	pOP-CNI01182_EST_C_1_pSK_SK	568
cl1785	ct1927	cn2087	pOP-CNI02031_EST_C_1_pSK_SK	147
cl1785	ct1927	cn2087	pOP-EO02164_EST_C_1_pSK_SK	539
cl1785	ct1927	cn2087	pOP-EO03195_EST_C_1_pSK_SK	471
cl1786	ct1928	cn2088	pOP-EO02825_EST_C_1_pSK_SK	348
cl1786	ct1928	cn2088	pOP-EO02826_EST_C_1_pSK_SK	154
cl1786	ct1928	cn2088	pOP-EO03214_EST_C_1_pSK_SK	609
cl1787	ct1929	cn2089	pOP-CNH02211_EST_C_1_pSK_SK	636
cl1787	ct1929	cn2089	pOP-EO03226_EST_C_1_pSK_SK	472
cl1787	ct1929	cn2090	pOP-CNH04582	526
cl1788	ct1930	cn2091	pOP-CEO01427_EST_C_1_pSK_SK	200
cl1788	ct1930	cn2091	pOP-CEO02680_EST_C_1_pSK_SK	255
cl1788	ct1930	cn2091	pOP-CNH01721_EST_C_1_pSK_SK	443
cl1788	ct1930	cn2091	pOP-CNH02041_EST_C_1_pSK_SK	453
cl1788	ct1930	cn2091	pOP-CNH02126_EST_C_1_pSK_SK	230
cl1788	ct1930	cn2091	pOP-CNH02229_EST_C_1_pSK_SK	218
cl1788	ct1930	cn2091	pOP-CNH02451_EST_C_1_pSK_SK	393
cl1788	ct1930	cn2091	pOP-CNH03392_EST_C_1_pSK_SK	443
cl1788	ct1930	cn2091	pOP-CNHP00452_EST_C_1_pSK_SK	221
cl1788	ct1930	cn2091	pOP-CNIP00880_EST_C_1_pSK_SK	321
cl1788	ct1930	cn2091	pOP-CNNP00020_EST_C_1_pBSK_SK	161
cl1788	ct1930	cn2091	pOP-EO03228_EST_C_1_pSK_SK	393
cl1789	ct1931	cn2092	pOP-CEO03111_EST_C_1_pSK_SK	355
cl1789	ct1931	cn2092	pOP-CNH01339_EST_C_1_pSK_SK	605
cl1789	ct1931	cn2092	pOP-EO02992_EST_C_1_pSK_SK	440
cl1789	ct1931	cn2092	pOP-EO03233_EST_C_1_pSK_SK	475
cl1790	ct1932	cn2093	pOP-EO02596_EST_C_1_pSK_SK	373
cl1790	ct1932	cn2093	pOP-EO03237_EST_C_1_pSK_SK	473
cl1791	ct1933	cn2094	pOP-EN00290_EST_C_1_pSK_SK	534
cl1791	ct1933	cn2094	pOP-EN00808_EST_C_1_pSK_SK	523
cl1791	ct1933	cn2094	pOP-EO03241_EST_C_1_pSK_SK	477

cl1792	ct1934	cn2095	pOP-CAP00242_EST_C_1_pBSK_SK	631
cl1792	ct1934	cn2095	pOP-EO03248_EST_C_1_pSK_SK	474
cl1793	ct1935	cn2096	pOP-EAP02752_EST_C_1_pBSK_SK	390
cl1793	ct1935	cn2096	pOP-EN00343_EST_C_1_pSK_SK	556
cl1793	ct1935	cn2096	pOP-ENP00012_EST_C_1_pSK_SK	390
cl1793	ct1935	cn2096	pOP-EO03253_EST_C_1_pSK_SK	476
cl1794	ct1936	cn2097	pOP-CNH04191	490
cl1794	ct1936	cn2097	pOP-EO03255_EST_C_1_pSK_SK	456
cl1795	ct1937	cn2098	pOP-CEO01661_EST_C_1_pSK_SK	473
cl1795	ct1937	cn2098	pOP-EAP02944_EST_C_1_pBSK_SK	455
cl1795	ct1937	cn2098	pOP-EAP03833_EST_C_1_pBSK_SK	550
cl1795	ct1937	cn2098	pOP-EAP03868_EST_C_1_pBSK_SK	474
cl1795	ct1937	cn2098	pOP-EO03260_EST_C_1_pSK_SK	474
cl1796	ct1938	cn2099	pOP-CEO02684_EST_C_1_pSK_SK	370
cl1796	ct1938	cn2099	pOP-CNH01068_EST_C_1_pSK_SK	426
cl1796	ct1938	cn2099	pOP-CNIP00678_EST_C_1_pSK_SK	418
cl1796	ct1938	cn2099	pOP-EO03268_EST_C_1_pSK_SK	404
cl1796	ct1938	cn2099	pOP-EO03272_EST_C_1_pSK_SK	476
cl1797	ct1939	cn2100	pOP-CEO00658_EST_C_1_pSK_SK	248
cl1797	ct1939	cn2100	pOP-CEO00908_EST_C_1_pSK_SK	349
cl1797	ct1939	cn2100	pOP-CEO01660_EST_C_1_pSK_SK	320
cl1797	ct1939	cn2100	pOP-CEO01936_EST_C_1_pSK_SK	367
cl1797	ct1939	cn2100	pOP-CEO02785_EST_C_1_pSK_SK	331
cl1797	ct1939	cn2100	pOP-CEO02924_EST_C_1_pSK_SK	257
cl1797	ct1939	cn2100	pOP-EAP00666_EST_C_1_pBSK_SK	419
cl1797	ct1939	cn2100	pOP-EAP01090_EST_C_1_pBSK_SK	243
cl1797	ct1939	cn2100	pOP-EAP01234_EST_C_1_pBSK_SK	218
cl1797	ct1939	cn2100	pOP-EAP01235_EST_C_1_pBSK_SK	306
cl1797	ct1939	cn2100	pOP-EAP01289_EST_C_1_pBSK_SK	315
cl1797	ct1939	cn2100	pOP-EAP01319_EST_C_1_pBSK_SK	321
cl1797	ct1939	cn2100	pOP-EAP01371_EST_C_1_pBSK_SK	373
cl1797	ct1939	cn2100	pOP-EAP02839_EST_C_1_pBSK_SK	440
cl1797	ct1939	cn2100	pOP-EAP02898_EST_C_1_pBSK_SK	232
cl1797	ct1939	cn2100	pOP-EAP02921_EST_C_1_pBSK_SK	325
cl1797	ct1939	cn2100	pOP-EAP03187_EST_C_1_pBSK_SK	383
cl1797	ct1939	cn2100	pOP-EAP03375_EST_C_1_pBSK_SK	414
cl1797	ct1939	cn2100	pOP-EAP03560_EST_C_1_pBSK_SK	277
cl1797	ct1939	cn2100	pOP-EO03276_EST_C_1_pSK_SK	341
cl1797	ct1939	cn2100	pOP-EO03772_EST_C_1_pSK_SK	510
cl1798	ct1940	cn2101	pOP-EO03250_EST_C_1_pSK_SK	469
cl1798	ct1940	cn2101	pOP-EO03283_EST_C_1_pSK_SK	361
cl1799	ct1941	cn2102	pOP-CAP00240_EST_C_1_pBSK_SK	641
cl1799	ct1942	cn2103	pOP-EO02618_EST_C_1_pSK_SK	371
cl1799	ct1943	cn2104	pOP-EO03287_EST_C_1_pSK_SK	315
cl1800	ct1944	cn2105	pOP-CNH00829_EST_C_1_pSK_SK	483
cl1800	ct1944	cn2105	pOP-EAP02381_EST_C_1_pBSK_SK	638
cl1800	ct1944	cn2105	pOP-EO03305_EST_C_1_pSK_SK	323
cl1800	ct1944	cn2105	pOP-EO06285_EST_C_1_pSK_SK	668
cl1801	ct1945	cn2106	pOP-CNH00630_EST_C_1_pSK_SK	771
cl1801	ct1945	cn2106	pOP-CNH00836_EST_C_1_pSK_SK	550
cl1801	ct1946	cn2107	pOP-CNH00559_EST_C_1_pSK_SK	652
cl1801	ct1946	cn2107	pOP-CNH00733_EST_C_1_pSK_SK	442
cl1801	ct1946	cn2107	pOP-CNIP00979_EST_C_1_pSK_SK	266
cl1801	ct1946	cn2107	pOP-EAP03447_EST_C_1_pBSK_SK	319
cl1801	ct1946	cn2107	pOP-EO03213_EST_C_1_pSK_SK	698
cl1801	ct1946	cn2107	pOP-EO03312_EST_C_1_pSK_SK	462

cl1802	ct1947	cn2108	pOP-EO02647_EST_C_1_pSK_SK	460
cl1802	ct1947	cn2108	pOP-EO03329_EST_C_1_pSK_SK	464
cl1802	ct1948	cn2109	pOP-CBP00090_EST_C_1_pBSK_SK	500
cl1802	ct1948	cn2109	pOP-CEO02716_EST_C_1_pSK_SK	419
cl1802	ct1948	cn2109	pOP-CEO03477_EST_C_1_pSK_SK	412
cl1802	ct1948	cn2109	pOP-CNI02086_EST_C_1_pSK_SK	402
cl1802	ct1948	cn2109	pOP-CNIP00069_EST_C_1_pSK_SK	453
cl1802	ct1948	cn2109	pOP-CNIP00885_EST_C_1_pSK_SK	373
cl1802	ct1948	cn2109	pOP-CNIP04033_EST_C_1_pSK_SK	290
cl1802	ct1948	cn2109	pOP-EO02519_EST_C_1_pSK_SK	371
cl1802	ct1948	cn2109	pOP-EO02991_EST_C_1_pSK_SK	379
cl1802	ct1948	cn2109	pOP-EO07008_EST_C_1_pSK_SK	541
cl1803	ct1949	cn2110	pOP-CNH02470_EST_C_1_pSK_SK	413
cl1803	ct1949	cn2110	pOP-EN00601_EST_C_1_pSK_SK	538
cl1803	ct1949	cn2110	pOP-EO03338_EST_C_1_pSK_SK	467
cl1804	ct1950	cn2111	pOP-EO03322_EST_C_1_pSK_SK	461
cl1804	ct1950	cn2111	pOP-EO03347_EST_C_1_pSK_SK	375
cl1805	ct1951	cn2112	pOP-CEO03096_EST_C_1_pSK_SK	219
cl1805	ct1951	cn2112	pOP-EAP00998_EST_C_1_pBSK_SK	153
cl1805	ct1951	cn2112	pOP-EAP01127_EST_C_1_pBSK_SK	465
cl1805	ct1951	cn2112	pOP-EO06507_EST_C_1_pSK_SK	728
cl1805	ct1951	cn2113	pOP-EO02091_EST_C_1_pSK_SK	649
cl1805	ct1951	cn2113	pOP-EO03351_EST_C_1_pSK_SK	376
cl1805	ct1951	cn2114	pOP-EO05634_EST_C_1_pSK_SK	523
cl1806	ct1952	cn2115	pOP-CAP00311_EST_C_1_pBSK_SK	449
cl1806	ct1952	cn2115	pOP-EO03095_EST_C_1_pSK_SK	481
cl1806	ct1952	cn2115	pOP-EO03365_EST_C_1_pSK_SK	463
cl1807	ct1953	cn2116	pOP-EAP01882_EST_C_1_pBSK_SK	539
cl1807	ct1954	cn2117	pOP-CEO02244_EST_C_1_pSK_SK	375
cl1807	ct1954	cn2117	pOP-EO07082_EST_C_1_pSK_SK	519
cl1807	ct1955	cn2118	pOP-CNI02133_EST_C_1_pSK_SK	626
cl1807	ct1955	cn2118	pOP-EAP00108_EST_C_1_pBSK_SK	645
cl1807	ct1955	cn2118	pOP-EAP03873_EST_C_1_pBSK_SK	618
cl1807	ct1955	cn2118	pOP-EO03227_EST_C_1_pSK_SK	473
cl1807	ct1955	cn2118	pOP-EO03376_EST_C_1_pSK_SK	405
cl1808	ct1956	cn2119	pOP-CNH00689_EST_C_1_pSK_SK	452
cl1808	ct1956	cn2119	pOP-CNI02157_EST_C_1_pSK_SK	509
cl1808	ct1956	cn2119	pOP-EAP02956_EST_C_1_pBSK_SK	574
cl1808	ct1956	cn2119	pOP-EO02506_EST_C_1_pSK_SK	528
cl1808	ct1956	cn2119	pOP-EO03380_EST_C_1_pSK_SK	464
cl1808	ct1956	cn2119	pOP-EO04110_EST_C_1_pSK_SK	421
cl1809	ct1957	cn2120	pOP-CEO01754_EST_C_1_pSK_SK	369
cl1809	ct1957	cn2120	pOP-CEO02061_EST_C_1_pSK_SK	261
cl1809	ct1957	cn2120	pOP-CEO03098_EST_C_1_pSK_SK	264
cl1809	ct1957	cn2120	pOP-EAP01870_EST_C_1_pBSK_SK	502
cl1809	ct1957	cn2120	pOP-EAP03411_EST_C_1_pBSK_SK	589
cl1809	ct1957	cn2120	pOP-EO03388_EST_C_1_pSK_SK	462
cl1810	ct1958	cn2121	pOP-CAP00339_EST_C_1_pBSK_SK	325
cl1810	ct1958	cn2121	pOP-CAP00346_EST_C_1_pBSK_SK	527
cl1810	ct1958	cn2121	pOP-EO03394_EST_C_1_pSK_SK	459
cl1811	ct1959	cn2122	pOP-CNH04305	420
cl1811	ct1959	cn2122	pOP-CNI02226_EST_C_1_pSK_SK	512
cl1811	ct1959	cn2122	pOP-CNI02253_EST_C_1_pSK_SK	512
cl1811	ct1959	cn2122	pOP-CNIP00674_EST_C_1_pSK_SK	369
cl1811	ct1959	cn2122	pOP-EO03043_EST_C_1_pSK_SK	268
cl1811	ct1959	cn2122	pOP-EO03398_EST_C_1_pSK_SK	460

cl1812	ct1960	cn2123	pOP-CNH04788_EST_C_1_pSK_SK	565
cl1812	ct1960	cn2123	pOP-EO03403_EST_C_1_pSK_SK	406
cl1813	ct1961	cn2124	pOP-EAP01883_EST_C_1_pBSK_SK	589
cl1813	ct1961	cn2124	pOP-EO03433_EST_C_1_pSK_SK	523
cl1814	ct1962	cn2125	pOP-CNHP00038_EST_C_1_pSK_SK	682
cl1814	ct1962	cn2125	pOP-EO02334_EST_C_1_pSK_SK	792
cl1814	ct1962	cn2125	pOP-EO03434_EST_C_1_pSK_SK	537
cl1815	ct1963	cn2126	pOP-EO03460_EST_C_1_pSK_SK	475
cl1815	ct1963	cn2126	pOP-EO06119_EST_C_1_pSK_SK	566
cl1816	ct1964	cn2127	pOP-CAP00010_EST_C_1_pBSK_SK	627
cl1816	ct1964	cn2127	pOP-EO02436_EST_C_1_pSK_SK	465
cl1816	ct1964	cn2127	pOP-EO03463_EST_C_1_pSK_SK	538
cl1817	ct1965	cn2128	pOP-CNH01277_EST_C_1_pSK_SK	689
cl1817	ct1965	cn2128	pOP-EO03467_EST_C_1_pSK_SK	499
cl1817	ct1965	cn2128	pOP-EO06502_EST_C_1_pSK_SK	732
cl1817	ct1965	cn2129	pOP-EO03426_EST_C_1_pSK_SK	473
cl1818	ct1966	cn2130	pOP-CBP00152_EST_C_1_pBSK_SK	631
cl1818	ct1966	cn2130	pOP-EAP01396_EST_C_1_pBSK_SK	344
cl1818	ct1966	cn2130	pOP-EO03503_EST_C_1_pSK_SK	546
cl1819	ct1967	cn2131	pOP-CAP00226_EST_C_1_pBSK_SK	635
cl1819	ct1967	cn2131	pOP-CAP00397_EST_C_1_pBSK_SK	657
cl1819	ct1967	cn2131	pOP-CBP00084_EST_C_1_pBSK_SK	219
cl1819	ct1967	cn2131	pOP-CNH00924_EST_C_1_pSK_SK	399
cl1819	ct1967	cn2131	pOP-CNH03144_EST_C_1_pSK_SK	536
cl1819	ct1967	cn2131	pOP-CNI01813_EST_C_1_pSK_SK	515
cl1819	ct1967	cn2131	pOP-CNIP00492_EST_C_1_pSK_SK	597
cl1819	ct1967	cn2131	pOP-CNIP00836_EST_C_1_pSK_SK	537
cl1819	ct1967	cn2131	pOP-EAP00993_EST_C_1_pBSK_SK	122
cl1819	ct1967	cn2131	pOP-EO02419_EST_C_1_pSK_SK	462
cl1819	ct1967	cn2131	pOP-EO02601_EST_C_1_pSK_SK	371
cl1819	ct1967	cn2131	pOP-EO03485_EST_C_1_pSK_SK	537
cl1819	ct1967	cn2131	pOP-EO03526_EST_C_1_pSK_SK	535
cl1819	ct1967	cn2131	pOP-EO03566_EST_C_1_pSK_SK	109
cl1819	ct1967	cn2131	pOP-EO04539_EST_C_1_pSK_SK	483
cl1820	ct1968	cn2132	pOP-CAP00277_EST_C_1_pBSK_SK	498
cl1820	ct1968	cn2132	pOP-CBP00263_EST_C_1_pBSK_SK	575
cl1820	ct1968	cn2132	pOP-CNI01244_EST_C_1_pSK_SK	380
cl1820	ct1968	cn2132	pOP-CNI01670_EST_C_1_pSK_SK	370
cl1820	ct1968	cn2132	pOP-CNIP00402_EST_C_1_pSK_SK	167
cl1820	ct1968	cn2132	pOP-EO02276_EST_C_1_pSK_SK	670
cl1820	ct1968	cn2132	pOP-EO02345_EST_C_1_pSK_SK	631
cl1820	ct1968	cn2132	pOP-EO04273_EST_C_1_pSK_SK	505
cl1820	ct1968	cn2132	pOP-EO04630_EST_C_1_pSK_SK	535
cl1820	ct1969	cn2133	pOP-CAP00187_EST_C_1_pBSK_SK	585
cl1820	ct1969	cn2133	pOP-CAP00239_EST_C_1_pBSK_SK	563
cl1820	ct1969	cn2133	pOP-CBP00051_EST_C_1_pBSK_SK	571
cl1820	ct1969	cn2133	pOP-CBP00063_EST_C_1_pBSK_SK	585
cl1820	ct1969	cn2133	pOP-CBP00076_EST_C_1_pBSK_SK	465
cl1820	ct1969	cn2133	pOP-CBP00237_EST_C_1_pBSK_SK	546
cl1820	ct1969	cn2133	pOP-CEO02956_EST_C_1_pSK_SK	105
cl1820	ct1969	cn2133	pOP-CNI01360_EST_C_1_pSK_SK	309
cl1820	ct1969	cn2133	pOP-CNI01369_EST_C_1_pSK_SK	511
cl1820	ct1969	cn2133	pOP-CNI01401_EST_C_1_pSK_SK	588
cl1820	ct1969	cn2133	pOP-CNI01566_EST_C_1_pSK_SK	203
cl1820	ct1969	cn2133	pOP-CNI01595_EST_C_1_pSK_SK	197
cl1820	ct1969	cn2133	pOP-CNI01609_EST_C_1_pSK_SK	124

cl1820	ct1969	cn2133	pOP-CNI01686_EST_C_1_pSK_SK	167
cl1820	ct1969	cn2133	pOP-CNI01693_EST_C_1_pSK_SK	359
cl1820	ct1969	cn2133	pOP-CNI01812_EST_C_1_pSK_SK	185
cl1820	ct1969	cn2133	pOP-CNIP00124_EST_C_1_pSK_SK	276
cl1820	ct1969	cn2133	pOP-CNIP00245_EST_C_1_pSK_SK	235
cl1820	ct1969	cn2133	pOP-CNIP00547_EST_C_1_pSK_SK	423
cl1820	ct1969	cn2133	pOP-CNIP04001_EST_C_1_pSK_SK	274
cl1820	ct1969	cn2133	pOP-CNIP04046_EST_C_1_pSK_SK	237
cl1820	ct1969	cn2133	pOP-EAP00314_EST_C_1_pBSK_SK	289
cl1820	ct1969	cn2133	pOP-EAP02000_EST_C_1_pBSK_SK	396
cl1820	ct1969	cn2133	pOP-EAP02149_EST_C_1_pBSK_SK	301
cl1820	ct1970	cn2134	pOP-CBP00041_EST_C_1_pBSK_SK	595
cl1820	ct1970	cn2134	pOP-CBP00095_EST_C_1_pBSK_SK	471
cl1820	ct1970	cn2134	pOP-CBP00101_EST_C_1_pBSK_SK	426
cl1820	ct1970	cn2134	pOP-CBP00206_EST_C_1_pBSK_SK	219
cl1820	ct1970	cn2134	pOP-CBP00221_EST_C_1_pBSK_SK	221
cl1820	ct1970	cn2134	pOP-CBP00243_EST_C_1_pBSK_SK	479
cl1820	ct1970	cn2134	pOP-CEO00549_EST_C_1_pSK_SK	377
cl1820	ct1970	cn2134	pOP-CEO01463_EST_C_1_pSK_SK	580
cl1820	ct1970	cn2134	pOP-CEO02696_EST_C_1_pSK_SK	415
cl1820	ct1970	cn2134	pOP-CEOP00024_EST_C_1_pSK_SK	159
cl1820	ct1970	cn2134	pOP-CNI01627_EST_C_1_pSK_SK	388
cl1820	ct1970	cn2134	pOP-CNIP00485_EST_C_1_pSK_SK	371
cl1820	ct1970	cn2134	pOP-CNIP04028_EST_C_1_pSK_SK	459
cl1820	ct1970	cn2134	pOP-EAP02927_EST_C_1_pBSK_SK	344
cl1820	ct1970	cn2134	pOP-EAP03689_EST_C_1_pBSK_SK	228
cl1820	ct1970	cn2134	pOP-EO02470_EST_C_1_pSK_SK	470
cl1820	ct1970	cn2134	pOP-EO02891_EST_C_1_pSK_SK	419
cl1820	ct1970	cn2134	pOP-EO02908_EST_C_1_pSK_SK	413
cl1820	ct1970	cn2134	pOP-EO03478_EST_C_1_pSK_SK	530
cl1820	ct1970	cn2134	pOP-EO03484_EST_C_1_pSK_SK	536
cl1820	ct1970	cn2134	pOP-EO03547_EST_C_1_pSK_SK	471
cl1820	ct1970	cn2134	pOP-EO03585_EST_C_1_pSK_SK	518
cl1820	ct1970	cn2134	pOP-EO04467_EST_C_1_pSK_SK	319
cl1820	ct1970	cn2134	pOP-EO05025_EST_C_1_pSK_SK	505
cl1820	ct1970	cn2134	pOP-EOP00022_EST_C_1_pSK_SK	292
cl1820	ct1971	cn2135	pOP-CAP00072_EST_C_1_pBSK_SK	611
cl1820	ct1971	cn2135	pOP-CAP00111_EST_C_1_pBSK_SK	514
cl1820	ct1971	cn2135	pOP-CAP00261_EST_C_1_pBSK_SK	546
cl1820	ct1971	cn2135	pOP-CBP00083_EST_C_1_pBSK_SK	486
cl1820	ct1971	cn2135	pOP-CEO00862_EST_C_1_pSK_SK	247
cl1820	ct1971	cn2135	pOP-CEO01523_EST_C_1_pSK_SK	246
cl1820	ct1971	cn2135	pOP-CEO02827_EST_C_1_pSK_SK	540
cl1820	ct1971	cn2135	pOP-CEO03094_EST_C_1_pSK_SK	226
cl1820	ct1971	cn2135	pOP-CEO03681_EST_C_1_pSK_SK	292
cl1820	ct1971	cn2135	pOP-CNH01616_EST_C_1_pSK_SK	247
cl1820	ct1971	cn2135	pOP-CNI01725_EST_C_1_pSK_SK	227
cl1820	ct1971	cn2135	pOP-CNI01733_EST_C_1_pSK_SK	337
cl1820	ct1971	cn2135	pOP-CNI01904_EST_C_1_pSK_SK	238
cl1820	ct1971	cn2135	pOP-CNIP00341_EST_C_1_pSK_SK	490
cl1820	ct1971	cn2135	pOP-CNIP00378_EST_C_1_pSK_SK	246
cl1820	ct1971	cn2135	pOP-CNIP00689_EST_C_1_pSK_SK	266
cl1820	ct1971	cn2135	pOP-EAP00901_EST_C_1_pBSK_SK	169
cl1820	ct1971	cn2135	pOP-EAP01121_EST_C_1_pBSK_SK	345
cl1820	ct1971	cn2135	pOP-EAP02106_EST_C_1_pBSK_SK	167
cl1820	ct1971	cn2135	pOP-EO02018_EST_C_1_pSK_SK	395

cl1820	ct1971	cn2135	pOP-EO02362_EST_C_1_pSK_SK	625
cl1820	ct1971	cn2135	pOP-EO02381_EST_C_1_pSK_SK	633
cl1820	ct1971	cn2135	pOP-EO02628_EST_C_1_pSK_SK	357
cl1820	ct1971	cn2135	pOP-EO02879_EST_C_1_pSK_SK	455
cl1820	ct1971	cn2135	pOP-EO02907_EST_C_1_pSK_SK	394
cl1820	ct1971	cn2135	pOP-EO03089_EST_C_1_pSK_SK	476
cl1820	ct1971	cn2135	pOP-EO03464_EST_C_1_pSK_SK	540
cl1820	ct1971	cn2135	pOP-EO03533_EST_C_1_pSK_SK	188
cl1820	ct1971	cn2135	pOP-EO05412_EST_C_1_pSK_SK	421
cl1820	ct1971	cn2135	pOP-EO06613_EST_C_1_pSK_SK	641
cl1820	ct1971	cn2135	pOP-EO08247_EST_C_1_pSK_SK	463
cl1820	ct1971	cn2135	pOP-EOP00002_EST_C_1_pSK_SK	623
cl1821	ct1972	cn2136	pOP-EAP01039_EST_C_1_pBSK_SK	242
cl1821	ct1972	cn2136	pOP-EAP01451_EST_C_1_pBSK_SK	245
cl1821	ct1972	cn2136	pOP-EO03548_EST_C_1_pSK_SK	191
cl1822	ct1973	cn2137	pOP-CNI02052_EST_C_1_pSK_SK	362
cl1822	ct1973	cn2137	pOP-EO03553_EST_C_1_pSK_SK	509
cl1822	ct1973	cn2137	pOP-EO04037_EST_C_1_pSK_SK	543
cl1822	ct1973	cn2137	pOP-EO04148_EST_C_1_pSK_SK	504
cl1823	ct1974	cn2138	pOP-CBP00132_EST_C_1_pBSK_SK	526
cl1823	ct1974	cn2138	pOP-CBP00256_EST_C_1_pBSK_SK	570
cl1823	ct1974	cn2138	pOP-CEO02021_EST_C_1_pSK_SK	211
cl1823	ct1974	cn2138	pOP-CEO02407_EST_C_1_pSK_SK	281
cl1823	ct1974	cn2138	pOP-CNH00676_EST_C_1_pSK_SK	571
cl1823	ct1974	cn2138	pOP-CNH03001_EST_C_1_pSK_SK	455
cl1823	ct1974	cn2138	pOP-CNH03374_EST_C_1_pSK_SK	437
cl1823	ct1974	cn2138	pOP-CNH05018_EST_C_1_pSK_SK	458
cl1823	ct1974	cn2138	pOP-CNI01480_EST_C_1_pSK_SK	495
cl1823	ct1974	cn2138	pOP-CNI01593_EST_C_1_pSK_SK	226
cl1823	ct1974	cn2138	pOP-CNI01756_EST_C_1_pSK_SK	268
cl1823	ct1974	cn2138	pOP-CNI01848_EST_C_1_pSK_SK	284
cl1823	ct1974	cn2138	pOP-CNI01977_EST_C_1_pSK_SK	416
cl1823	ct1974	cn2138	pOP-CNIP00186_EST_C_1_pSK_SK	463
cl1823	ct1974	cn2138	pOP-CNIP00427_EST_C_1_pSK_SK	241
cl1823	ct1974	cn2138	pOP-CNIP00522_EST_C_1_pSK_SK	559
cl1823	ct1974	cn2138	pOP-CNIP00846_EST_C_1_pSK_SK	602
cl1823	ct1974	cn2138	pOP-CNIP00932_EST_C_1_pSK_SK	192
cl1823	ct1974	cn2138	pOP-CNIP01047_EST_C_1_pSK_SK	519
cl1823	ct1974	cn2138	pOP-CNIP04022_EST_C_1_pSK_SK	385
cl1823	ct1974	cn2138	pOP-EO02126_EST_C_1_pSK_SK	512
cl1823	ct1974	cn2138	pOP-EO03555_EST_C_1_pSK_SK	519
cl1823	ct1974	cn2139	pOP-CEO00696_EST_C_1_pSK_SK	470
cl1824	ct1975	cn2140	pOP-CNI01322_EST_C_1_pSK_SK	555
cl1824	ct1975	cn2140	pOP-EO03558_EST_C_1_pSK_SK	542
cl1825	ct1976	cn2141	pOP-CBP00042_EST_C_1_pBSK_SK	575
cl1825	ct1976	cn2141	pOP-EO03580_EST_C_1_pSK_SK	545
cl1826	ct1977	cn2142	pOP-EO02666_EST_C_1_pSK_SK	457
cl1826	ct1977	cn2142	pOP-EO03587_EST_C_1_pSK_SK	499
cl1827	ct1978	cn2143	pOP-EO03570_EST_C_1_pSK_SK	241
cl1827	ct1978	cn2143	pOP-EO03605_EST_C_1_pSK_SK	426
cl1828	ct1979	cn2144	pOP-CNH01007_EST_C_1_pSK_SK	304
cl1828	ct1979	cn2144	pOP-CNH01573_EST_C_1_pSK_SK	706
cl1828	ct1979	cn2144	pOP-CNH02031_EST_C_1_pSK_SK	548
cl1828	ct1979	cn2144	pOP-CNH04227	483
cl1828	ct1979	cn2144	pOP-CNH04256	540
cl1828	ct1979	cn2144	pOP-EO03610_EST_C_1_pSK_SK	469

cl1829	ct1980	cn2145	pOP-EO03628_EST_C_1_pSK_SK	475
cl1829	ct1980	cn2145	pOP-EO05536_EST_C_1_pSK_SK	436
cl1830	ct1981	cn2146	pOP-CEO03037_EST_C_1_pSK_SK	497
cl1830	ct1981	cn2146	pOP-CEO03695_EST_C_1_pSK_SK	297
cl1830	ct1981	cn2146	pOP-EO03635_EST_C_1_pSK_SK	457
cl1831	ct1982	cn2147	pOP-CNH03080_EST_C_1_pSK_SK	706
cl1831	ct1982	cn2147	pOP-EO03639_EST_C_1_pSK_SK	447
cl1832	ct1983	cn2148	pOP-EO05902_EST_C_1_pSK_SK	526
cl1832	ct1983	cn2149	pOP-EO02445_EST_C_1_pSK_SK	465
cl1832	ct1983	cn2150	pOP-EO03658_EST_C_1_pSK_SK	328
cl1833	ct1984	cn2151	pOP-CNH01114_EST_C_1_pSK_SK	362
cl1833	ct1984	cn2151	pOP-EO03662_EST_C_1_pSK_SK	477
cl1834	ct1985	cn2152	pOP-CEO01113_EST_C_1_pSK_SK	369
cl1834	ct1985	cn2152	pOP-CEO01558_EST_C_1_pSK_SK	185
cl1834	ct1985	cn2152	pOP-EO03251_EST_C_1_pSK_SK	433
cl1834	ct1985	cn2152	pOP-EO03663_EST_C_1_pSK_SK	357
cl1834	ct1985	cn2152	pOP-EO06166_EST_C_1_pSK_SK	468
cl1835	ct1986	cn2153	pOP-CEO03112_EST_C_1_pSK_SK	356
cl1835	ct1986	cn2153	pOP-CNH02735_EST_C_1_pSK_SK	698
cl1835	ct1986	cn2153	pOP-CNH03709_EST_C_1_pSK_SK	629
cl1835	ct1986	cn2153	pOP-CNH03712_EST_C_1_pSK_SK	294
cl1835	ct1986	cn2153	pOP-CNH03713_EST_C_1_pSK_SK	301
cl1835	ct1986	cn2153	pOP-CNH03717_EST_C_1_pSK_SK	405
cl1835	ct1986	cn2153	pOP-CNH04767_EST_C_1_pSK_SK	576
cl1835	ct1986	cn2153	pOP-CNIP00720_EST_C_1_pSK_SK	442
cl1835	ct1986	cn2153	pOP-EO02809_EST_C_1_pSK_SK	365
cl1835	ct1986	cn2153	pOP-EO03667_EST_C_1_pSK_SK	400
cl1836	ct1987	cn2154	pOP-CAP00358_EST_C_1_pBSK_SK	504
cl1836	ct1987	cn2154	pOP-CAP00376_EST_C_1_pBSK_SK	598
cl1836	ct1987	cn2154	pOP-CAP00384_EST_C_1_pBSK_SK	621
cl1836	ct1987	cn2154	pOP-CEO00637_EST_C_1_pSK_SK	211
cl1836	ct1987	cn2154	pOP-CEOP00018_EST_C_1_pSK_SK	133
cl1836	ct1987	cn2154	pOP-EAP00514_EST_C_1_pBSK_SK	286
cl1836	ct1987	cn2154	pOP-EAP02725_EST_C_1_pBSK_SK	449
cl1836	ct1987	cn2154	pOP-EO02201_EST_C_1_pSK_SK	605
cl1836	ct1987	cn2154	pOP-EO02214_EST_C_1_pSK_SK	385
cl1836	ct1987	cn2154	pOP-EO02736_EST_C_1_pSK_SK	448
cl1836	ct1987	cn2154	pOP-EO02841_EST_C_1_pSK_SK	458
cl1836	ct1987	cn2154	pOP-EO02849_EST_C_1_pSK_SK	445
cl1836	ct1987	cn2154	pOP-EO02896_EST_C_1_pSK_SK	219
cl1836	ct1987	cn2154	pOP-EO03682_EST_C_1_pSK_SK	434
cl1836	ct1987	cn2154	pOP-EO04463_EST_C_1_pSK_SK	471
cl1836	ct1987	cn2154	pOP-EO06861_EST_C_1_pSK_SK	546
cl1836	ct1987	cn2154	pOP-EO07019_EST_C_1_pSK_SK	672
cl1836	ct1987	cn2154	pOP-EO08188_EST_C_1_pSK_SK	441
cl1836	ct1987	cn2154	pOP-EO08258_EST_C_1_pSK_SK	340
cl1837	ct1988	cn2155	pOP-CNHP00027_EST_C_1_pSK_SK	575
cl1837	ct1988	cn2155	pOP-EO03683_EST_C_1_pSK_SK	337
cl1838	ct1989	cn2156	pOP-CNH00698_EST_C_1_pSK_SK	586
cl1838	ct1989	cn2156	pOP-CNH00916_EST_C_1_pSK_SK	479
cl1838	ct1989	cn2156	pOP-EO03702_EST_C_1_pSK_SK	357
cl1839	ct1990	cn2157	pOP-EO03718_EST_C_1_pSK_SK	346
cl1839	ct1991	cn2158	pOP-EO02170_EST_C_1_pSK_SK	628
cl1840	ct1992	cn2159	pOP-CNH04887_EST_C_1_pSK_SK	740
cl1840	ct1992	cn2160	pOP-EAP02753_EST_C_1_pBSK_SK	508
cl1840	ct1992	cn2160	pOP-EO03734_EST_C_1_pSK_SK	465

cl1841	ct1993	cn2161	pOP-EAP01551_EST_C_1_pBSK_SK	386
cl1841	ct1993	cn2161	pOP-EO02328_EST_C_1_pSK_SK	457
cl1841	ct1993	cn2161	pOP-EO03736_EST_C_1_pSK_SK	483
cl1842	ct1994	cn2162	pOP-EO03738_EST_C_1_pSK_SK	528
cl1842	ct1995	cn2163	pOP-CNHP00070_EST_C_1_pSK_SK	652
cl1843	ct1996	cn2164	pOP-CNNP00028_EST_C_1_pBSK_SK	327
cl1843	ct1996	cn2164	pOP-EO03751_EST_C_1_pSK_SK	522
cl1844	ct1997	cn2165	pOP-EN00519_EST_C_1_pSK_SK	497
cl1844	ct1997	cn2165	pOP-EO03755_EST_C_1_pSK_SK	530
cl1845	ct1998	cn2166	pOP-CNHP00338_EST_C_1_pSK_SK	671
cl1845	ct1998	cn2166	pOP-EO03508_EST_C_1_pSK_SK	489
cl1845	ct1998	cn2167	pOP-EO03757_EST_C_1_pSK_SK	531
cl1846	ct1999	cn2168	pOP-EN00517_EST_C_1_pSK_SK	513
cl1846	ct1999	cn2168	pOP-EO03760_EST_C_1_pSK_SK	489
cl1847	ct2000	cn2169	pOP-EO03764_EST_C_1_pSK_SK	529
cl1847	ct2000	cn2169	pOP-EO06877_EST_C_1_pSK_SK	632
cl1847	ct2000	cn2169	pOP-EO06980_EST_C_1_pSK_SK	679
cl1848	ct2001	cn2170	pOP-CEO03103_EST_C_1_pSK_SK	294
cl1848	ct2001	cn2170	pOP-CNIP00687_EST_C_1_pSK_SK	405
cl1848	ct2001	cn2170	pOP-EO02078_EST_C_1_pSK_SK	167
cl1848	ct2001	cn2170	pOP-EO03780_EST_C_1_pSK_SK	536
cl1849	ct2002	cn2171	pOP-EN00410_EST_C_1_pSK_SK	493
cl1849	ct2002	cn2171	pOP-EO03781_EST_C_1_pSK_SK	534
cl1850	ct2003	cn2172	pOP-CNIP00321_EST_C_1_pSK_SK	311
cl1850	ct2003	cn2172	pOP-EO02220_EST_C_1_pSK_SK	636
cl1850	ct2003	cn2172	pOP-EO03811_EST_C_1_pSK_SK	263
cl1850	ct2003	cn2172	pOP-EO07055_EST_C_1_pSK_SK	560
cl1851	ct2004	cn2173	pOP-EO02035_EST_C_1_pSK_SK	654
cl1851	ct2004	cn2173	pOP-EO03004_EST_C_1_pSK_SK	450
cl1851	ct2004	cn2173	pOP-EO03821_EST_C_1_pSK_SK	528
cl1852	ct2005	cn2174	pOP-CNIP01061_EST_C_1_pSK_SK	404
cl1852	ct2005	cn2174	pOP-EAP03779_EST_C_1_pBSK_SK	320
cl1852	ct2005	cn2174	pOP-EO03830_EST_C_1_pSK_SK	398
cl1853	ct2006	cn2175	pOP-EAP00505_EST_C_1_pBSK_SK	677
cl1853	ct2006	cn2175	pOP-EO03839_EST_C_1_pSK_SK	525
cl1854	ct2007	cn2176	pOP-CBP00043_EST_C_1_pBSK_SK	625
cl1854	ct2007	cn2176	pOP-CEO00942_EST_C_1_pSK_SK	222
cl1854	ct2007	cn2176	pOP-CEO01565_EST_C_1_pSK_SK	430
cl1854	ct2008	cn2177	pOP-CEO03732_EST_C_1_pSK_SK	398
cl1854	ct2008	cn2177	pOP-EO03846_EST_C_1_pSK_SK	532
cl1854	ct2008	cn2177	pOP-EO05286_EST_C_1_pSK_SK	597
cl1854	ct2008	cn2177	pOP-EO07367_EST_C_1_pSK_SK	834
cl1854	ct2009	cn2178	pOP-CEM00225_EST_C_1_pSK_SK	227
cl1854	ct2009	cn2178	pOP-CEM00226_EST_C_1_pSK_SK	226
cl1854	ct2009	cn2178	pOP-CEO00807_EST_C_1_pSK_SK	323
cl1854	ct2009	cn2178	pOP-CNH00892_EST_C_1_pSK_SK	463
cl1854	ct2009	cn2178	pOP-CNH01681_EST_C_1_pSK_SK	598
cl1854	ct2009	cn2178	pOP-CNI01117_EST_C_1_pSK_SK	362
cl1854	ct2009	cn2178	pOP-CNI02146_EST_C_1_pSK_SK	553
cl1854	ct2009	cn2178	pOP-CNIP00692_EST_C_1_pSK_SK	695
cl1854	ct2009	cn2178	pOP-EO04871_EST_C_1_pSK_SK	510
cl1854	ct2010	cn2179	pOP-CEO02488_EST_C_1_pSK_SK	568
cl1854	ct2010	cn2179	pOP-CEO03183_EST_C_1_pSK_SK	352
cl1854	ct2010	cn2179	pOP-CEO03493_EST_C_1_pSK_SK	661
cl1854	ct2010	cn2179	pOP-CEO03494_EST_C_1_pSK_SK	383
cl1854	ct2010	cn2179	pOP-CNH01239_EST_C_1_pSK_SK	535

cl1854	ct2010	cn2179	pOP-CNH01646_EST_C_1_pSK_SK	664
cl1854	ct2010	cn2179	pOP-CNH04772_EST_C_1_pSK_SK	445
cl1854	ct2010	cn2179	pOP-CNHP00425_EST_C_1_pSK_SK	720
cl1854	ct2010	cn2179	pOP-EAP01494_EST_C_1_pBSK_SK	657
cl1854	ct2010	cn2179	pOP-EAP01690_EST_C_1_pBSK_SK	542
cl1854	ct2010	cn2179	pOP-EO02727_EST_C_1_pSK_SK	451
cl1854	ct2010	cn2179	pOP-EO03310_EST_C_1_pSK_SK	424
cl1854	ct2010	cn2179	pOP-EO04550_EST_C_1_pSK_SK	371
cl1854	ct2010	cn2179	pOP-EO04713_EST_C_1_pSK_SK	518
cl1854	ct2010	cn2179	pOP-EO05174_EST_C_1_pSK_SK	493
cl1854	ct2010	cn2179	pOP-EO08402_EST_C_1_pSK_SK	336
cl1855	ct2011	cn2180	pOP-CNH01159_EST_C_1_pSK_SK	521
cl1855	ct2011	cn2180	pOP-CNHP00247_EST_C_1_pSK_SK	469
cl1855	ct2011	cn2180	pOP-CNHP00371_EST_C_1_pSK_SK	596
cl1855	ct2011	cn2180	pOP-EO03847_EST_C_1_pSK_SK	523
cl1856	ct2012	cn2181	pOP-EO03843_EST_C_1_pSK_SK	520
cl1856	ct2012	cn2181	pOP-EO03849_EST_C_1_pSK_SK	521
cl1857	ct2013	cn2182	pOP-CEO01527_EST_C_1_pSK_SK	541
cl1857	ct2013	cn2182	pOP-CEO01698_EST_C_1_pSK_SK	591
cl1857	ct2013	cn2182	pOP-CNH02632_EST_C_1_pSK_SK	629
cl1857	ct2013	cn2182	pOP-CNH02726_EST_C_1_pSK_SK	694
cl1857	ct2013	cn2182	pOP-CNH04461	667
cl1857	ct2013	cn2182	pOP-CNHP00267_EST_C_1_pSK_SK	313
cl1857	ct2013	cn2182	pOP-CNI01845_EST_C_1_pSK_SK	409
cl1857	ct2013	cn2182	pOP-CNIP00599_EST_C_1_pSK_SK	615
cl1857	ct2013	cn2182	pOP-EO02587_EST_C_1_pSK_SK	477
cl1857	ct2013	cn2182	pOP-EO03335_EST_C_1_pSK_SK	405
cl1857	ct2013	cn2182	pOP-EO03707_EST_C_1_pSK_SK	361
cl1857	ct2013	cn2182	pOP-EO03856_EST_C_1_pSK_SK	522
cl1858	ct2014	cn2183	pOP-EO03813_EST_C_1_pSK_SK	518
cl1858	ct2014	cn2183	pOP-EO03864_EST_C_1_pSK_SK	518
cl1859	ct2015	cn2184	pOP-CEO01198_EST_C_1_pSK_SK	648
cl1859	ct2015	cn2184	pOP-CNH03009_EST_C_1_pSK_SK	568
cl1859	ct2015	cn2184	pOP-CNH04335	788
cl1859	ct2015	cn2184	pOP-CNH04357	771
cl1859	ct2015	cn2184	pOP-CNH04716_EST_C_1_pSK_SK	389
cl1859	ct2015	cn2184	pOP-CNIP00963_EST_C_1_pSK_SK	651
cl1859	ct2015	cn2184	pOP-EO08410_EST_C_1_pSK_SK	373
cl1859	ct2016	cn2185	pOP-CEO00849_EST_C_1_pSK_SK	392
cl1859	ct2016	cn2185	pOP-CEO01952_EST_C_1_pSK_SK	261
cl1859	ct2016	cn2185	pOP-CNH00678_EST_C_1_pSK_SK	543
cl1859	ct2016	cn2185	pOP-CNH02572_EST_C_1_pSK_SK	549
cl1859	ct2016	cn2185	pOP-EO03392_EST_C_1_pSK_SK	430
cl1859	ct2016	cn2185	pOP-EO03865_EST_C_1_pSK_SK	528
cl1859	ct2016	cn2185	pOP-EO08113_EST_C_1_pSK_SK	374
cl1860	ct2017	cn2186	pOP-EO03901_EST_C_1_pSK_SK	595
cl1860	ct2017	cn2186	pOP-EO03903_EST_C_1_pSK_SK	595
cl1861	ct2018	cn2187	pOP-EO03277_EST_C_1_pSK_SK	414
cl1861	ct2018	cn2187	pOP-EO03647_EST_C_1_pSK_SK	477
cl1861	ct2018	cn2187	pOP-EO03905_EST_C_1_pSK_SK	560
cl1862	ct2019	cn2188	pOP-EN00443_EST_C_1_pSK_SK	498
cl1862	ct2019	cn2188	pOP-EO03933_EST_C_1_pSK_SK	535
cl1863	ct2020	cn2189	pOP-EO02071_EST_C_1_pSK_SK	466
cl1863	ct2020	cn2189	pOP-EO03939_EST_C_1_pSK_SK	487
cl1864	ct2021	cn2190	pOP-EO03937_EST_C_1_pSK_SK	484
cl1864	ct2021	cn2190	pOP-EO03941_EST_C_1_pSK_SK	488

cl1865	ct2022	cn2191	pOP-EO03912_EST_C_1_pSK_SK	335
cl1865	ct2022	cn2191	pOP-EO03944_EST_C_1_pSK_SK	273
cl1866	ct2023	cn2192	pOP-EO02251_EST_C_1_pSK_SK	620
cl1866	ct2023	cn2192	pOP-EO03877_EST_C_1_pSK_SK	390
cl1866	ct2023	cn2192	pOP-EO03878_EST_C_1_pSK_SK	454
cl1866	ct2023	cn2192	pOP-EO03881_EST_C_1_pSK_SK	505
cl1866	ct2023	cn2192	pOP-EO03882_EST_C_1_pSK_SK	454
cl1866	ct2023	cn2192	pOP-EO03883_EST_C_1_pSK_SK	544
cl1866	ct2023	cn2192	pOP-EO03884_EST_C_1_pSK_SK	294
cl1866	ct2023	cn2192	pOP-EO03948_EST_C_1_pSK_SK	475
cl1867	ct2024	cn2193	pOP-EAP03664_EST_C_1_pBSK_SK	589
cl1867	ct2024	cn2193	pOP-EO03950_EST_C_1_pSK_SK	486
cl1868	ct2025	cn2194	pOP-EO03913_EST_C_1_pSK_SK	526
cl1868	ct2025	cn2194	pOP-EO03928_EST_C_1_pSK_SK	524
cl1868	ct2025	cn2194	pOP-EO03953_EST_C_1_pSK_SK	488
cl1869	ct2026	cn2195	pOP-CNIP00203_EST_C_1_pSK_SK	504
cl1869	ct2026	cn2195	pOP-EO03964_EST_C_1_pSK_SK	431
cl1870	ct2027	cn2196	pOP-EO03792_EST_C_1_pSK_SK	490
cl1870	ct2028	cn2197	pOP-EO03972_EST_C_1_pSK_SK	447
cl1871	ct2029	cn2198	pOP-EO03986_EST_C_1_pSK_SK	506
cl1871	ct2029	cn2198	pOP-EO04003_EST_C_1_pSK_SK	528
cl1872	ct2030	cn2199	pOP-EAP01686_EST_C_1_pBSK_SK	483
cl1872	ct2030	cn2199	pOP-EO04024_EST_C_1_pSK_SK	559
cl1873	ct2031	cn2200	pOP-CEO03327_EST_C_1_pSK_SK	550
cl1873	ct2031	cn2200	pOP-CEO03354_EST_C_1_pSK_SK	446
cl1873	ct2031	cn2200	pOP-CNH01782_EST_C_1_pSK_SK	512
cl1873	ct2031	cn2200	pOP-CNH01831_EST_C_1_pSK_SK	508
cl1873	ct2031	cn2200	pOP-EAP01383_EST_C_1_pBSK_SK	220
cl1873	ct2031	cn2200	pOP-EO04046_EST_C_1_pSK_SK	279
cl1874	ct2032	cn2201	pOP-CNIP00134_EST_C_1_pSK_SK	298
cl1874	ct2032	cn2201	pOP-EAP01985_EST_C_1_pBSK_SK	425
cl1874	ct2033	cn2202	pOP-CNH01414_EST_C_1_pSK_SK	619
cl1874	ct2033	cn2203	pOP-CEO02205_EST_C_1_pSK_SK	575
cl1874	ct2033	cn2203	pOP-CNH01066_EST_C_1_pSK_SK	237
cl1874	ct2034	cn2204	pOP-CAP00369_EST_C_1_pBSK_SK	418
cl1874	ct2034	cn2204	pOP-CBP00172_EST_C_1_pBSK_SK	299
cl1874	ct2034	cn2204	pOP-CEO01042_EST_C_1_pSK_SK	270
cl1874	ct2034	cn2204	pOP-CEO01061_EST_C_1_pSK_SK	450
cl1874	ct2034	cn2204	pOP-EAP00662_EST_C_1_pBSK_SK	462
cl1874	ct2034	cn2204	pOP-EAP01389_EST_C_1_pBSK_SK	210
cl1874	ct2034	cn2204	pOP-EAP02307_EST_C_1_pBSK_SK	226
cl1874	ct2034	cn2204	pOP-EAP03280_EST_C_1_pBSK_SK	460
cl1874	ct2034	cn2204	pOP-EO02366_EST_C_1_pSK_SK	444
cl1874	ct2034	cn2204	pOP-EO03670_EST_C_1_pSK_SK	480
cl1874	ct2034	cn2204	pOP-EO04047_EST_C_1_pSK_SK	511
cl1874	ct2034	cn2204	pOP-EO04048_EST_C_1_pSK_SK	203
cl1874	ct2034	cn2204	pOP-EO04135_EST_C_1_pSK_SK	544
cl1874	ct2034	cn2204	pOP-EO05148_EST_C_1_pSK_SK	512
cl1875	ct2035	cn2205	pOP-CEO01917_EST_C_1_pSK_SK	190
cl1875	ct2035	cn2205	pOP-EAP00904_EST_C_1_pBSK_SK	272
cl1875	ct2035	cn2205	pOP-EAP03182_EST_C_1_pBSK_SK	288
cl1875	ct2035	cn2205	pOP-EO04058_EST_C_1_pSK_SK	487
cl1876	ct2036	cn2206	pOP-EAP00287_EST_C_1_pBSK_SK	516
cl1876	ct2036	cn2206	pOP-EO04100_EST_C_1_pSK_SK	441
cl1877	ct2037	cn2207	pOP-EO03977_EST_C_1_pSK_SK	461
cl1877	ct2037	cn2207	pOP-EO04115_EST_C_1_pSK_SK	270

cl1878	ct2038	cn2208	pOP-EO03980_EST_C_1_pSK_SK	470
cl1878	ct2038	cn2208	pOP-EO04117_EST_C_1_pSK_SK	370
cl1879	ct2039	cn2209	pOP-EO03971_EST_C_1_pSK_SK	409
cl1879	ct2039	cn2209	pOP-EO04120_EST_C_1_pSK_SK	413
cl1880	ct2040	cn2210	pOP-EO04013_EST_C_1_pSK_SK	436
cl1880	ct2040	cn2210	pOP-EO04121_EST_C_1_pSK_SK	406
cl1881	ct2041	cn2211	pOP-EO03978_EST_C_1_pSK_SK	516
cl1881	ct2041	cn2211	pOP-EO04123_EST_C_1_pSK_SK	354
cl1882	ct2042	cn2212	pOP-EO04012_EST_C_1_pSK_SK	458
cl1882	ct2042	cn2212	pOP-EO04124_EST_C_1_pSK_SK	398
cl1883	ct2043	cn2213	pOP-EO04049_EST_C_1_pSK_SK	616
cl1883	ct2043	cn2213	pOP-EO04126_EST_C_1_pSK_SK	526
cl1884	ct2044	cn2214	pOP-EO04023_EST_C_1_pSK_SK	439
cl1884	ct2044	cn2214	pOP-EO04035_EST_C_1_pSK_SK	337
cl1884	ct2044	cn2214	pOP-EO04127_EST_C_1_pSK_SK	482
cl1884	ct2044	cn2214	pOP-EO04128_EST_C_1_pSK_SK	330
cl1885	ct2045	cn2215	pOP-CEO01882_EST_C_1_pSK_SK	251
cl1885	ct2045	cn2215	pOP-CNH01296_EST_C_1_pSK_SK	512
cl1885	ct2045	cn2215	pOP-CNH01702_EST_C_1_pSK_SK	483
cl1885	ct2045	cn2215	pOP-EO03449_EST_C_1_pSK_SK	457
cl1885	ct2045	cn2215	pOP-EO03456_EST_C_1_pSK_SK	538
cl1885	ct2045	cn2215	pOP-EO03645_EST_C_1_pSK_SK	449
cl1885	ct2045	cn2215	pOP-EO04006_EST_C_1_pSK_SK	498
cl1885	ct2045	cn2215	pOP-EO04137_EST_C_1_pSK_SK	441
cl1886	ct2046	cn2216	pOP-EO04111_EST_C_1_pSK_SK	535
cl1886	ct2046	cn2216	pOP-EO04112_EST_C_1_pSK_SK	539
cl1886	ct2046	cn2216	pOP-EO04113_EST_C_1_pSK_SK	544
cl1886	ct2046	cn2216	pOP-EO04138_EST_C_1_pSK_SK	481
cl1887	ct2047	cn2217	pOP-EO04028_EST_C_1_pSK_SK	538
cl1887	ct2047	cn2217	pOP-EO04142_EST_C_1_pSK_SK	443
cl1888	ct2048	cn2218	pOP-EO03974_EST_C_1_pSK_SK	484
cl1888	ct2048	cn2218	pOP-EO04144_EST_C_1_pSK_SK	410
cl1889	ct2049	cn2219	pOP-EO02720_EST_C_1_pSK_SK	403
cl1889	ct2049	cn2219	pOP-EO04015_EST_C_1_pSK_SK	457
cl1889	ct2049	cn2219	pOP-EO04145_EST_C_1_pSK_SK	358
cl1890	ct2050	cn2220	pOP-EO03975_EST_C_1_pSK_SK	520
cl1890	ct2050	cn2220	pOP-EO04146_EST_C_1_pSK_SK	390
cl1891	ct2051	cn2221	pOP-EO03981_EST_C_1_pSK_SK	482
cl1891	ct2051	cn2221	pOP-EO04150_EST_C_1_pSK_SK	490
cl1892	ct2052	cn2222	pOP-EO04016_EST_C_1_pSK_SK	369
cl1892	ct2052	cn2222	pOP-EO04151_EST_C_1_pSK_SK	471
cl1893	ct2053	cn2223	pOP-EO03982_EST_C_1_pSK_SK	477
cl1893	ct2053	cn2223	pOP-EO03997_EST_C_1_pSK_SK	452
cl1893	ct2053	cn2223	pOP-EO04004_EST_C_1_pSK_SK	491
cl1893	ct2053	cn2223	pOP-EO04005_EST_C_1_pSK_SK	408
cl1893	ct2053	cn2223	pOP-EO04153_EST_C_1_pSK_SK	370
cl1894	ct2054	cn2224	pOP-EO04020_EST_C_1_pSK_SK	398
cl1894	ct2054	cn2224	pOP-EO04154_EST_C_1_pSK_SK	474
cl1895	ct2055	cn2225	pOP-CEO01399_EST_C_1_pSK_SK	499
cl1895	ct2055	cn2225	pOP-EO02383_EST_C_1_pSK_SK	631
cl1895	ct2055	cn2225	pOP-EO04029_EST_C_1_pSK_SK	519
cl1895	ct2055	cn2225	pOP-EO04157_EST_C_1_pSK_SK	333
cl1896	ct2056	cn2226	pOP-EO03998_EST_C_1_pSK_SK	379
cl1896	ct2056	cn2226	pOP-EO04161_EST_C_1_pSK_SK	387
cl1897	ct2057	cn2227	pOP-EO03973_EST_C_1_pSK_SK	517
cl1897	ct2057	cn2227	pOP-EO04162_EST_C_1_pSK_SK	515

cl1898	ct2058	cn2228	pOP-EO04021_EST_C_1_pSK_SK	467
cl1898	ct2058	cn2228	pOP-EO04165_EST_C_1_pSK_SK	336
cl1899	ct2059	cn2229	pOP-EO04019_EST_C_1_pSK_SK	587
cl1899	ct2059	cn2229	pOP-EO04172_EST_C_1_pSK_SK	394
cl1900	ct2060	cn2230	pOP-EO04055_EST_C_1_pSK_SK	568
cl1900	ct2060	cn2230	pOP-EO04177_EST_C_1_pSK_SK	533
cl1901	ct2061	cn2231	pOP-CEO03605_EST_C_1_pSK_SK	195
cl1901	ct2061	cn2231	pOP-CEOP00027_EST_C_1_pSK_SK	159
cl1901	ct2061	cn2231	pOP-EO02608_EST_C_1_pSK_SK	373
cl1901	ct2061	cn2231	pOP-EO04002_EST_C_1_pSK_SK	468
cl1901	ct2061	cn2231	pOP-EO04178_EST_C_1_pSK_SK	387
cl1901	ct2061	cn2231	pOP-EO07021_EST_C_1_pSK_SK	654
cl1902	ct2062	cn2232	pOP-EO04086_EST_C_1_pSK_SK	382
cl1902	ct2062	cn2232	pOP-EO04179_EST_C_1_pSK_SK	541
cl1903	ct2063	cn2233	pOP-EO04085_EST_C_1_pSK_SK	380
cl1903	ct2063	cn2233	pOP-EO04180_EST_C_1_pSK_SK	508
cl1904	ct2064	cn2234	pOP-EO04027_EST_C_1_pSK_SK	481
cl1904	ct2064	cn2234	pOP-EO04181_EST_C_1_pSK_SK	493
cl1905	ct2065	cn2235	pOP-EO04078_EST_C_1_pSK_SK	440
cl1905	ct2065	cn2235	pOP-EO04183_EST_C_1_pSK_SK	496
cl1906	ct2066	cn2236	pOP-CNH01778_EST_C_1_pSK_SK	476
cl1906	ct2066	cn2236	pOP-EO04191_EST_C_1_pSK_SK	545
cl1907	ct2067	cn2237	pOP-CEO00914_EST_C_1_pSK_SK	211
cl1907	ct2067	cn2237	pOP-CEO00931_EST_C_1_pSK_SK	211
cl1907	ct2067	cn2237	pOP-EO04043_EST_C_1_pSK_SK	524
cl1907	ct2067	cn2237	pOP-EO04196_EST_C_1_pSK_SK	544
cl1908	ct2068	cn2238	pOP-CEO00681_EST_C_1_pSK_SK	229
cl1908	ct2068	cn2238	pOP-CEO00993_EST_C_1_pSK_SK	390
cl1908	ct2068	cn2238	pOP-EO04197_EST_C_1_pSK_SK	387
cl1909	ct2069	cn2239	pOP-CEO03105_EST_C_1_pSK_SK	490
cl1909	ct2069	cn2239	pOP-CNI01872_EST_C_1_pSK_SK	321
cl1909	ct2069	cn2239	pOP-EO04099_EST_C_1_pSK_SK	537
cl1909	ct2069	cn2239	pOP-EO04206_EST_C_1_pSK_SK	527
cl1910	ct2070	cn2240	pOP-EO04068_EST_C_1_pSK_SK	319
cl1910	ct2070	cn2240	pOP-EO04208_EST_C_1_pSK_SK	317
cl1911	ct2071	cn2241	pOP-EO04081_EST_C_1_pSK_SK	543
cl1911	ct2071	cn2241	pOP-EO04223_EST_C_1_pSK_SK	543
cl1912	ct2072	cn2242	pOP-EO04052_EST_C_1_pSK_SK	596
cl1912	ct2072	cn2242	pOP-EO04225_EST_C_1_pSK_SK	543
cl1913	ct2073	cn2243	pOP-EO02302_EST_C_1_pSK_SK	709
cl1913	ct2073	cn2243	pOP-EO04098_EST_C_1_pSK_SK	457
cl1913	ct2073	cn2243	pOP-EO04226_EST_C_1_pSK_SK	449
cl1914	ct2074	cn2244	pOP-EO04104_EST_C_1_pSK_SK	555
cl1914	ct2074	cn2244	pOP-EO04230_EST_C_1_pSK_SK	542
cl1915	ct2075	cn2245	pOP-CAP00202_EST_C_1_pBSK_SK	591
cl1915	ct2075	cn2245	pOP-EO04084_EST_C_1_pSK_SK	384
cl1915	ct2075	cn2245	pOP-EO04231_EST_C_1_pSK_SK	546
cl1916	ct2076	cn2246	pOP-EO04075_EST_C_1_pSK_SK	496
cl1916	ct2076	cn2246	pOP-EO04232_EST_C_1_pSK_SK	544
cl1917	ct2077	cn2247	pOP-EO04041_EST_C_1_pSK_SK	572
cl1917	ct2077	cn2247	pOP-EO04234_EST_C_1_pSK_SK	540
cl1918	ct2078	cn2248	pOP-EAP00761_EST_C_1_pBSK_SK	403
cl1918	ct2078	cn2248	pOP-EAP03348_EST_C_1_pBSK_SK	470
cl1918	ct2078	cn2248	pOP-EO03404_EST_C_1_pSK_SK	458
cl1918	ct2078	cn2248	pOP-EO04296_EST_C_1_pSK_SK	511
cl1918	ct2078	cn2248	pOP-EO07041_EST_C_1_pSK_SK	503

cl1918	ct2078	cn2249	pOP-EO04246_EST_C_1_pSK_SK	346
cl1919	ct2079	cn2250	pOP-EO04253_EST_C_1_pSK_SK	260
cl1919	ct2079	cn2250	pOP-EO04254_EST_C_1_pSK_SK	113
cl1920	ct2080	cn2251	pOP-CNI02051_EST_C_1_pSK_SK	222
cl1920	ct2080	cn2251	pOP-EO04255_EST_C_1_pSK_SK	310
cl1921	ct2081	cn2252	pOP-CEO01687_EST_C_1_pSK_SK	597
cl1921	ct2081	cn2252	pOP-CNI01174_EST_C_1_pSK_SK	319
cl1921	ct2081	cn2252	pOP-CNI01329_EST_C_1_pSK_SK	498
cl1921	ct2081	cn2252	pOP-CNI01690_EST_C_1_pSK_SK	316
cl1921	ct2081	cn2252	pOP-EO04259_EST_C_1_pSK_SK	375
cl1921	ct2082	cn2253	pOP-CEO01677_EST_C_1_pSK_SK	216
cl1921	ct2082	cn2253	pOP-CEO03380_EST_C_1_pSK_SK	606
cl1921	ct2082	cn2253	pOP-EAP00242_EST_C_1_pBSK_SK	322
cl1921	ct2082	cn2253	pOP-EAP01213_EST_C_1_pBSK_SK	275
cl1921	ct2082	cn2253	pOP-EAP01343_EST_C_1_pBSK_SK	293
cl1921	ct2082	cn2253	pOP-EAP01554_EST_C_1_pBSK_SK	535
cl1921	ct2082	cn2253	pOP-EAP01740_EST_C_1_pBSK_SK	447
cl1921	ct2082	cn2253	pOP-EAP01800_EST_C_1_pBSK_SK	503
cl1921	ct2082	cn2253	pOP-EAP01894_EST_C_1_pBSK_SK	412
cl1921	ct2082	cn2253	pOP-EAP02836_EST_C_1_pBSK_SK	602
cl1921	ct2082	cn2253	pOP-EAP02929_EST_C_1_pBSK_SK	716
cl1921	ct2082	cn2253	pOP-EAP03173_EST_C_1_pBSK_SK	601
cl1921	ct2082	cn2253	pOP-EAP03276_EST_C_1_pBSK_SK	435
cl1921	ct2082	cn2253	pOP-EAP03872_EST_C_1_pBSK_SK	647
cl1922	ct2083	cn2254	pOP-CNH01340_EST_C_1_pSK_SK	656
cl1922	ct2083	cn2254	pOP-CNH02709_EST_C_1_pSK_SK	592
cl1922	ct2083	cn2254	pOP-CNHP00488_EST_C_1_pSK_SK	555
cl1922	ct2083	cn2254	pOP-EAP03855_EST_C_1_pBSK_SK	685
cl1922	ct2083	cn2254	pOP-EO04267_EST_C_1_pSK_SK	415
cl1922	ct2083	cn2255	pOP-EO07040_EST_C_1_pSK_SK	569
cl1923	ct2084	cn2256	pOP-CNH00674_EST_C_1_pSK_SK	513
cl1923	ct2084	cn2256	pOP-EO04275_EST_C_1_pSK_SK	352
cl1924	ct2085	cn2257	pOP-EO04280_EST_C_1_pSK_SK	519
cl1924	ct2085	cn2258	pOP-EN00875_EST_C_1_pSK_SK	431
cl1925	ct2086	cn2259	pOP-EN00190_EST_C_1_pSK_SK	446
cl1925	ct2086	cn2259	pOP-EO04291_EST_C_1_pSK_SK	514
cl1926	ct2087	cn2260	pOP-EO03187_EST_C_1_pSK_SK	458
cl1926	ct2087	cn2260	pOP-EO04295_EST_C_1_pSK_SK	514
cl1927	ct2088	cn2261	pOP-CAP00064_EST_C_1_pBSK_SK	515
cl1927	ct2088	cn2261	pOP-CAP00362_EST_C_1_pBSK_SK	548
cl1927	ct2088	cn2261	pOP-CAP00383_EST_C_1_pBSK_SK	412
cl1927	ct2088	cn2261	pOP-CNIP00683_EST_C_1_pSK_SK	357
cl1927	ct2088	cn2261	pOP-EAP02841_EST_C_1_pBSK_SK	561
cl1927	ct2088	cn2261	pOP-EO02971_EST_C_1_pSK_SK	434
cl1927	ct2088	cn2261	pOP-EO04308_EST_C_1_pSK_SK	343
cl1928	ct2089	cn2262	pOP-EO02983_EST_C_1_pSK_SK	392
cl1928	ct2089	cn2262	pOP-EO04309_EST_C_1_pSK_SK	348
cl1929	ct2090	cn2263	pOP-EO04317_EST_C_1_pSK_SK	408
cl1929	ct2090	cn2263	pOP-EO05589_EST_C_1_pSK_SK	451
cl1930	ct2091	cn2264	pOP-EO04320_EST_C_1_pSK_SK	201
cl1930	ct2091	cn2264	pOP-EO04323_EST_C_1_pSK_SK	210
cl1931	ct2092	cn2265	pOP-CNI01873_EST_C_1_pSK_SK	374
cl1931	ct2092	cn2265	pOP-EO04326_EST_C_1_pSK_SK	499
cl1932	ct2093	cn2266	pOP-CNIP00233_EST_C_1_pSK_SK	295
cl1932	ct2093	cn2266	pOP-CNIP00947_EST_C_1_pSK_SK	502
cl1932	ct2093	cn2266	pOP-EAP00836_EST_C_1_pBSK_SK	324

cl1932	ct2093	cn2266	pOP-EO04336_EST_C_1_pSK_SK	525
cl1933	ct2094	cn2267	pOP-EO02733_EST_C_1_pSK_SK	440
cl1933	ct2094	cn2267	pOP-EO04337_EST_C_1_pSK_SK	527
cl1934	ct2095	cn2268	pOP-CNIP01023_EST_C_1_pSK_SK	206
cl1934	ct2095	cn2268	pOP-EAP02143_EST_C_1_pBSK_SK	124
cl1934	ct2095	cn2268	pOP-EO03448_EST_C_1_pSK_SK	282
cl1934	ct2095	cn2268	pOP-EO04947_EST_C_1_pSK_SK	475
cl1934	ct2095	cn2269	pOP-CEO00642_EST_C_1_pSK_SK	153
cl1934	ct2095	cn2269	pOP-EO04338_EST_C_1_pSK_SK	327
cl1935	ct2096	cn2270	pOP-CNIP00773_EST_C_1_pSK_SK	545
cl1935	ct2096	cn2270	pOP-EAP00607_EST_C_1_pBSK_SK	530
cl1935	ct2096	cn2270	pOP-EO04342_EST_C_1_pSK_SK	486
cl1936	ct2097	cn2271	pOP-CEO02442_EST_C_1_pSK_SK	669
cl1936	ct2097	cn2271	pOP-CNH00608_EST_C_1_pSK_SK	586
cl1936	ct2097	cn2271	pOP-EAP01232_EST_C_1_pBSK_SK	315
cl1936	ct2097	cn2271	pOP-EO04343_EST_C_1_pSK_SK	466
cl1937	ct2098	cn2272	pOP-EO04348_EST_C_1_pSK_SK	349
cl1937	ct2098	cn2272	pOP-EO04349_EST_C_1_pSK_SK	349
cl1938	ct2099	cn2273	pOP-EO04299_EST_C_1_pSK_SK	430
cl1938	ct2099	cn2273	pOP-EO04353_EST_C_1_pSK_SK	521
cl1938	ct2099	cn2273	pOP-EO04354_EST_C_1_pSK_SK	521
cl1939	ct2100	cn2274	pOP-CAP00228_EST_C_1_pBSK_SK	597
cl1939	ct2100	cn2274	pOP-CNH02494_EST_C_1_pSK_SK	459
cl1939	ct2100	cn2274	pOP-CNH02948_EST_C_1_pSK_SK	589
cl1939	ct2100	cn2274	pOP-CNH04355	494
cl1939	ct2100	cn2274	pOP-CNHP00447_EST_C_1_pSK_SK	489
cl1939	ct2100	cn2274	pOP-EAP02970_EST_C_1_pBSK_SK	452
cl1939	ct2100	cn2274	pOP-EO04363_EST_C_1_pSK_SK	301
cl1939	ct2100	cn2274	pOP-EO04615_EST_C_1_pSK_SK	402
cl1940	ct2101	cn2275	pOP-EO02464_EST_C_1_pSK_SK	459
cl1940	ct2101	cn2275	pOP-EO04367_EST_C_1_pSK_SK	506
cl1941	ct2102	cn2276	pOP-CNH04168	578
cl1941	ct2102	cn2276	pOP-EO04368_EST_C_1_pSK_SK	509
cl1942	ct2103	cn2277	pOP-CEM00054_EST_C_1_pSK_SK	302
cl1942	ct2103	cn2277	pOP-CEO02569_EST_C_1_pSK_SK	460
cl1942	ct2103	cn2277	pOP-CEO02570_EST_C_1_pSK_SK	469
cl1942	ct2103	cn2277	pOP-CEOP00016_EST_C_1_pSK_SK	234
cl1942	ct2103	cn2277	pOP-EAP03244_EST_C_1_pBSK_SK	669
cl1942	ct2103	cn2277	pOP-EO04369_EST_C_1_pSK_SK	500
cl1942	ct2103	cn2278	pOP-EAP00874_EST_C_1_pBSK_SK	170
cl1942	ct2103	cn2278	pOP-EO02030_EST_C_1_pSK_SK	577
cl1943	ct2104	cn2279	pOP-CEO00513_EST_C_1_pSK_SK	171
cl1943	ct2104	cn2279	pOP-CEO01415_EST_C_1_pSK_SK	251
cl1943	ct2104	cn2279	pOP-CEO02056_EST_C_1_pSK_SK	233
cl1943	ct2104	cn2279	pOP-CNI02076_EST_C_1_pSK_SK	527
cl1943	ct2104	cn2279	pOP-CNI02114_EST_C_1_pSK_SK	248
cl1943	ct2104	cn2279	pOP-EAP01115_EST_C_1_pBSK_SK	130
cl1943	ct2104	cn2279	pOP-EO04376_EST_C_1_pSK_SK	442
cl1944	ct2105	cn2280	pOP-EO04380_EST_C_1_pSK_SK	380
cl1944	ct2105	cn2280	pOP-EO04381_EST_C_1_pSK_SK	417
cl1945	ct2106	cn2281	pOP-EO03715_EST_C_1_pSK_SK	301
cl1945	ct2106	cn2281	pOP-EO04382_EST_C_1_pSK_SK	339
cl1946	ct2107	cn2282	pOP-EAP00831_EST_C_1_pBSK_SK	326
cl1946	ct2107	cn2282	pOP-EO04401_EST_C_1_pSK_SK	214
cl1947	ct2108	cn2283	pOP-CNH00619_EST_C_1_pSK_SK	580
cl1947	ct2108	cn2283	pOP-EO04403_EST_C_1_pSK_SK	413

cl1948	ct2109	cn2284	pOP-EO03458_EST_C_1_pSK_SK	538
cl1948	ct2109	cn2284	pOP-EO04409_EST_C_1_pSK_SK	418
cl1949	ct2110	cn2285	pOP-CEM00131_EST_C_1_pSK_SK	172
cl1949	ct2110	cn2285	pOP-CEO00933_EST_C_1_pSK_SK	730
cl1949	ct2110	cn2285	pOP-CEO03786_EST_C_1_pSK_SK	484
cl1949	ct2110	cn2285	pOP-CNH00987_EST_C_1_pSK_SK	585
cl1949	ct2110	cn2285	pOP-CNH01233_EST_C_1_pSK_SK	558
cl1949	ct2110	cn2285	pOP-CNH02046_EST_C_1_pSK_SK	464
cl1949	ct2110	cn2285	pOP-CNH02373_EST_C_1_pSK_SK	452
cl1949	ct2110	cn2285	pOP-CNH02453_EST_C_1_pSK_SK	391
cl1949	ct2110	cn2285	pOP-CNH02628_EST_C_1_pSK_SK	538
cl1949	ct2110	cn2285	pOP-CNH02722_EST_C_1_pSK_SK	608
cl1949	ct2110	cn2285	pOP-CNH02885_EST_C_1_pSK_SK	594
cl1949	ct2110	cn2285	pOP-CNH03461_EST_C_1_pSK_SK	473
cl1949	ct2110	cn2285	pOP-CNHP00150_EST_C_1_pSK_SK	389
cl1949	ct2110	cn2285	pOP-CNI01701_EST_C_1_pSK_SK	484
cl1949	ct2110	cn2285	pOP-CNI01930_EST_C_1_pSK_SK	484
cl1949	ct2110	cn2285	pOP-EAP01824_EST_C_1_pBSK_SK	736
cl1949	ct2110	cn2285	pOP-EAP02914_EST_C_1_pBSK_SK	700
cl1949	ct2110	cn2285	pOP-EAP03484_EST_C_1_pBSK_SK	314
cl1949	ct2110	cn2285	pOP-EO06207_EST_C_1_pSK_SK	455
cl1949	ct2110	cn2285	pOP-EO08391_EST_C_1_pSK_SK	432
cl1949	ct2110	cn2286	pOP-EO04425_EST_C_1_pSK_SK	524
cl1950	ct2111	cn2287	pOP-EO02418_EST_C_1_pSK_SK	466
cl1950	ct2111	cn2287	pOP-EO04434_EST_C_1_pSK_SK	491
cl1951	ct2112	cn2288	pOP-CAP00210_EST_C_1_pBSK_SK	540
cl1951	ct2113	cn2289	pOP-CAP00208_EST_C_1_pBSK_SK	583
cl1951	ct2113	cn2290	pOP-CAP00209_EST_C_1_pBSK_SK	577
cl1951	ct2114	cn2291	pOP-CNHP00477_EST_C_1_pSK_SK	609
cl1951	ct2114	cn2291	pOP-EO02099_EST_C_1_pSK_SK	693
cl1951	ct2114	cn2291	pOP-EO04439_EST_C_1_pSK_SK	295
cl1952	ct2115	cn2292	pOP-EO04449_EST_C_1_pSK_SK	341
cl1952	ct2115	cn2292	pOP-EO05840_EST_C_1_pSK_SK	540
cl1953	ct2116	cn2293	pOP-EO03996_EST_C_1_pSK_SK	449
cl1953	ct2116	cn2293	pOP-EO04155_EST_C_1_pSK_SK	282
cl1953	ct2116	cn2293	pOP-EO04450_EST_C_1_pSK_SK	448
cl1954	ct2117	cn2294	pOP-CEO02701_EST_C_1_pSK_SK	301
cl1954	ct2117	cn2294	pOP-CEO03350_EST_C_1_pSK_SK	468
cl1954	ct2117	cn2294	pOP-CNIP00463_EST_C_1_pSK_SK	676
cl1954	ct2117	cn2294	pOP-EO03417_EST_C_1_pSK_SK	385
cl1954	ct2117	cn2294	pOP-EO04453_EST_C_1_pSK_SK	517
cl1955	ct2118	cn2295	pOP-CEO00917_EST_C_1_pSK_SK	358
cl1955	ct2118	cn2295	pOP-CEO03396_EST_C_1_pSK_SK	308
cl1955	ct2118	cn2295	pOP-EO04456_EST_C_1_pSK_SK	530
cl1956	ct2119	cn2296	pOP-EO02306_EST_C_1_pSK_SK	462
cl1956	ct2119	cn2296	pOP-EO02577_EST_C_1_pSK_SK	474
cl1956	ct2119	cn2296	pOP-EO04457_EST_C_1_pSK_SK	454
cl1957	ct2120	cn2297	pOP-CNI01270_EST_C_1_pSK_SK	435
cl1957	ct2120	cn2297	pOP-EAP00853_EST_C_1_pBSK_SK	511
cl1957	ct2120	cn2297	pOP-EAP00854_EST_C_1_pBSK_SK	511
cl1957	ct2121	cn2298	pOP-CAP00284_EST_C_1_pBSK_SK	575
cl1957	ct2121	cn2298	pOP-CBP00227_EST_C_1_pBSK_SK	588
cl1957	ct2121	cn2298	pOP-CNHP00330_EST_C_1_pSK_SK	409
cl1957	ct2121	cn2298	pOP-EO04472_EST_C_1_pSK_SK	485
cl1958	ct2122	cn2299	pOP-EAP01727_EST_C_1_pBSK_SK	286
cl1958	ct2122	cn2299	pOP-EO04480_EST_C_1_pSK_SK	430

cl1959	ct2123	cn2300	pOP-CNH00995_EST_C_1_pSK_SK	314
cl1959	ct2123	cn2300	pOP-CNH01338_EST_C_1_pSK_SK	579
cl1959	ct2123	cn2300	pOP-CNH01650_EST_C_1_pSK_SK	542
cl1959	ct2123	cn2300	pOP-CNH01798_EST_C_1_pSK_SK	464
cl1959	ct2123	cn2300	pOP-EAP03838_EST_C_1_pBSK_SK	538
cl1959	ct2123	cn2300	pOP-EO04485_EST_C_1_pSK_SK	462
cl1960	ct2124	cn2301	pOP-EN00848_EST_C_1_pSK_SK	409
cl1960	ct2124	cn2301	pOP-EN00888_EST_C_1_pSK_SK	532
cl1960	ct2124	cn2301	pOP-EO03860_EST_C_1_pSK_SK	518
cl1960	ct2124	cn2301	pOP-EO03891_EST_C_1_pSK_SK	471
cl1960	ct2124	cn2301	pOP-EO04492_EST_C_1_pSK_SK	361
cl1960	ct2124	cn2301	pOP-EO08487_EST_C_1_pSK_SK	232
cl1961	ct2125	cn2302	pOP-CAP00388_EST_C_1_pBSK_SK	627
cl1961	ct2125	cn2302	pOP-CNH00779_EST_C_1_pSK_SK	499
cl1961	ct2126	cn2303	pOP-CEO00766_EST_C_1_pSK_SK	374
cl1961	ct2126	cn2303	pOP-CEO01532_EST_C_1_pSK_SK	348
cl1961	ct2126	cn2303	pOP-CEO02824_EST_C_1_pSK_SK	164
cl1961	ct2126	cn2303	pOP-CEO03293_EST_C_1_pSK_SK	366
cl1961	ct2126	cn2303	pOP-CEO03430_EST_C_1_pSK_SK	619
cl1961	ct2126	cn2303	pOP-CNH01247_EST_C_1_pSK_SK	665
cl1961	ct2126	cn2303	pOP-CNH01252_EST_C_1_pSK_SK	679
cl1961	ct2126	cn2303	pOP-CNH01284_EST_C_1_pSK_SK	561
cl1961	ct2126	cn2303	pOP-CNH01341_EST_C_1_pSK_SK	420
cl1961	ct2126	cn2303	pOP-CNH01556_EST_C_1_pSK_SK	605
cl1961	ct2126	cn2303	pOP-CNH01674_EST_C_1_pSK_SK	665
cl1961	ct2126	cn2303	pOP-CNH01717_EST_C_1_pSK_SK	540
cl1961	ct2126	cn2303	pOP-CNH02227_EST_C_1_pSK_SK	727
cl1961	ct2126	cn2303	pOP-CNH02343_EST_C_1_pSK_SK	650
cl1961	ct2126	cn2303	pOP-CNH02488_EST_C_1_pSK_SK	332
cl1961	ct2126	cn2303	pOP-CNH02710_EST_C_1_pSK_SK	468
cl1961	ct2126	cn2303	pOP-CNH02786_EST_C_1_pSK_SK	678
cl1961	ct2126	cn2303	pOP-CNH02947_EST_C_1_pSK_SK	603
cl1961	ct2126	cn2303	pOP-CNH02950_EST_C_1_pSK_SK	621
cl1961	ct2126	cn2303	pOP-CNH03106_EST_C_1_pSK_SK	563
cl1961	ct2126	cn2303	pOP-CNH03532_EST_C_1_pSK_SK	593
cl1961	ct2126	cn2303	pOP-CNH03729_EST_C_1_pSK_SK	365
cl1961	ct2126	cn2303	pOP-CNH03814_EST_C_1_pSK_SK	668
cl1961	ct2126	cn2303	pOP-CNH03815_EST_C_1_pSK_SK	425
cl1961	ct2126	cn2303	pOP-CNH04296	560
cl1961	ct2126	cn2303	pOP-CNI01397_EST_C_1_pSK_SK	697
cl1961	ct2126	cn2303	pOP-CNI01944_EST_C_1_pSK_SK	517
cl1961	ct2126	cn2303	pOP-CNI02071_EST_C_1_pSK_SK	517
cl1961	ct2126	cn2303	pOP-CNI02099_EST_C_1_pSK_SK	458
cl1961	ct2126	cn2303	pOP-CNI02243_EST_C_1_pSK_SK	344
cl1961	ct2126	cn2303	pOP-CNI02254_EST_C_1_pSK_SK	344
cl1961	ct2126	cn2303	pOP-CNIP00057_EST_C_1_pSK_SK	480
cl1961	ct2126	cn2303	pOP-CNIP00680_EST_C_1_pSK_SK	571
cl1961	ct2126	cn2303	pOP-CNIP00884_EST_C_1_pSK_SK	723
cl1961	ct2126	cn2303	pOP-EAP01152_EST_C_1_pBSK_SK	374
cl1961	ct2126	cn2303	pOP-EAP02715_EST_C_1_pBSK_SK	507
cl1961	ct2126	cn2303	pOP-EAP02789_EST_C_1_pBSK_SK	699
cl1961	ct2126	cn2303	pOP-EAP03620_EST_C_1_pBSK_SK	582
cl1961	ct2126	cn2303	pOP-EO04501_EST_C_1_pSK_SK	506
cl1961	ct2126	cn2303	pOP-EO08204_EST_C_1_pSK_SK	592
cl1962	ct2127	cn2304	pOP-CEO03429_EST_C_1_pSK_SK	381
cl1962	ct2127	cn2304	pOP-EO04503_EST_C_1_pSK_SK	539

cl1963	ct2128	cn2305	pOP-CEO03005_EST_C_1_pSK_SK	551
cl1963	ct2128	cn2305	pOP-EO04512_EST_C_1_pSK_SK	539
cl1964	ct2129	cn2306	pOP-EO03507_EST_C_1_pSK_SK	486
cl1964	ct2129	cn2306	pOP-EO04513_EST_C_1_pSK_SK	431
cl1964	ct2129	cn2306	pOP-EO05599_EST_C_1_pSK_SK	466
cl1965	ct2130	cn2307	pOP-CNH01410_EST_C_1_pSK_SK	395
cl1965	ct2130	cn2307	pOP-CNHP00516_EST_C_1_pSK_SK	565
cl1965	ct2130	cn2307	pOP-EO03960_EST_C_1_pSK_SK	490
cl1965	ct2130	cn2307	pOP-EO04515_EST_C_1_pSK_SK	515
cl1966	ct2131	cn2308	pOP-EO03189_EST_C_1_pSK_SK	470
cl1966	ct2131	cn2308	pOP-EO04524_EST_C_1_pSK_SK	536
cl1967	ct2132	cn2309	pOP-CAP00175_EST_C_1_pBSK_SK	634
cl1967	ct2132	cn2309	pOP-CAP00413_EST_C_1_pBSK_SK	442
cl1967	ct2132	cn2309	pOP-CBP00228_EST_C_1_pBSK_SK	622
cl1967	ct2132	cn2309	pOP-EAP00169_EST_C_1_pBSK_SK	308
cl1967	ct2132	cn2309	pOP-EAP01747_EST_C_1_pBSK_SK	279
cl1967	ct2132	cn2309	pOP-EO04528_EST_C_1_pSK_SK	531
cl1967	ct2132	cn2309	pOP-EO07763_EST_C_1_pSK_SK	688
cl1968	ct2133	cn2310	pOP-CNIP00415_EST_C_1_pSK_SK	571
cl1968	ct2133	cn2310	pOP-EO04529_EST_C_1_pSK_SK	434
cl1969	ct2134	cn2311	pOP-EAP03643_EST_C_1_pBSK_SK	557
cl1969	ct2134	cn2311	pOP-EO04531_EST_C_1_pSK_SK	526
cl1970	ct2135	cn2312	pOP-CEO00685_EST_C_1_pSK_SK	267
cl1970	ct2135	cn2312	pOP-EAP01688_EST_C_1_pBSK_SK	570
cl1970	ct2135	cn2312	pOP-EAP02848_EST_C_1_pBSK_SK	641
cl1970	ct2135	cn2312	pOP-EO04536_EST_C_1_pSK_SK	542
cl1971	ct2136	cn2313	pOP-CEO00625_EST_C_1_pSK_SK	373
cl1971	ct2136	cn2313	pOP-EAP02010_EST_C_1_pBSK_SK	175
cl1971	ct2136	cn2313	pOP-EO04537_EST_C_1_pSK_SK	414
cl1972	ct2137	cn2314	pOP-CAP00044_EST_C_1_pBSK_SK	382
cl1972	ct2137	cn2314	pOP-CBP00068_EST_C_1_pBSK_SK	414
cl1972	ct2137	cn2314	pOP-EO04543_EST_C_1_pSK_SK	256
cl1973	ct2138	cn2315	pOP-EAP01819_EST_C_1_pBSK_SK	221
cl1973	ct2138	cn2315	pOP-EO04555_EST_C_1_pSK_SK	540
cl1974	ct2139	cn2316	pOP-EO04560_EST_C_1_pSK_SK	494
cl1974	ct2140	cn2317	pOP-CEO00999_EST_C_1_pSK_SK	286
cl1974	ct2140	cn2317	pOP-CEO01039_EST_C_1_pSK_SK	286
cl1974	ct2140	cn2317	pOP-CEO01159_EST_C_1_pSK_SK	208
cl1974	ct2140	cn2317	pOP-CNH00659_EST_C_1_pSK_SK	597
cl1974	ct2140	cn2317	pOP-EAP00616_EST_C_1_pBSK_SK	279
cl1974	ct2140	cn2317	pOP-EAP00855_EST_C_1_pBSK_SK	327
cl1974	ct2140	cn2317	pOP-EAP01620_EST_C_1_pBSK_SK	581
cl1974	ct2140	cn2317	pOP-EO03542_EST_C_1_pSK_SK	541
cl1974	ct2140	cn2317	pOP-EO05825_EST_C_1_pSK_SK	589
cl1975	ct2141	cn2318	pOP-EO02233_EST_C_1_pSK_SK	637
cl1975	ct2141	cn2318	pOP-EO04571_EST_C_1_pSK_SK	545
cl1976	ct2142	cn2319	pOP-ENP00021_EST_C_1_pSK_SK	184
cl1976	ct2142	cn2319	pOP-EO04580_EST_C_1_pSK_SK	482
cl1976	ct2143	cn2320	pOP-EAP02881_EST_C_1_pBSK_SK	480
cl1976	ct2143	cn2320	pOP-EO03520_EST_C_1_pSK_SK	354
cl1976	ct2143	cn2320	pOP-EO03800_EST_C_1_pSK_SK	460
cl1976	ct2144	cn2321	pOP-CEM00120_EST_C_1_pSK_SK	135
cl1976	ct2144	cn2321	pOP-CEO01376_EST_C_1_pSK_SK	286
cl1976	ct2144	cn2321	pOP-CEO01377_EST_C_1_pSK_SK	286
cl1976	ct2144	cn2321	pOP-CEO03513_EST_C_1_pSK_SK	494
cl1976	ct2144	cn2321	pOP-CNH00877_EST_C_1_pSK_SK	557

cl1976	ct2144	cn2321	pOP-CNH01638_EST_C_1_pSK_SK	538
cl1976	ct2144	cn2321	pOP-CNI01673_EST_C_1_pSK_SK	530
cl1976	ct2144	cn2321	pOP-CNIP00357_EST_C_1_pSK_SK	347
cl1976	ct2144	cn2321	pOP-EAP03145_EST_C_1_pBSK_SK	637
cl1976	ct2144	cn2321	pOP-EAP03828_EST_C_1_pBSK_SK	591
cl1976	ct2144	cn2321	pOP-EAP03850_EST_C_1_pBSK_SK	551
cl1976	ct2144	cn2321	pOP-EO08025_EST_C_1_pSK_SK	374
cl1976	ct2144	cn2322	pOP-CEM00259_EST_C_1_pSK_SK	473
cl1977	ct2145	cn2323	pOP-EO03046_EST_C_1_pSK_SK	327
cl1977	ct2146	cn2324	pOP-EO05867_EST_C_1_pSK_SK	522
cl1977	ct2147	cn2325	pOP-EAP01737_EST_C_1_pBSK_SK	688
cl1977	ct2147	cn2325	pOP-EO03618_EST_C_1_pSK_SK	454
cl1977	ct2147	cn2326	pOP-CEO02032_EST_C_1_pSK_SK	331
cl1977	ct2148	cn2327	pOP-EO03262_EST_C_1_pSK_SK	364
cl1977	ct2148	cn2327	pOP-EO03314_EST_C_1_pSK_SK	264
cl1977	ct2148	cn2327	pOP-EO06648_EST_C_1_pSK_SK	770
cl1977	ct2148	cn2327	pOP-EO06775_EST_C_1_pSK_SK	818
cl1977	ct2148	cn2328	pOP-EO02741_EST_C_1_pSK_SK	451
cl1977	ct2149	cn2329	pOP-CEO01999_EST_C_1_pSK_SK	325
cl1977	ct2149	cn2329	pOP-CEO02446_EST_C_1_pSK_SK	235
cl1977	ct2149	cn2329	pOP-CEO03637_EST_C_1_pSK_SK	207
cl1977	ct2149	cn2329	pOP-EO02558_EST_C_1_pSK_SK	479
cl1977	ct2149	cn2329	pOP-EO03581_EST_C_1_pSK_SK	423
cl1977	ct2149	cn2329	pOP-EO03790_EST_C_1_pSK_SK	515
cl1977	ct2149	cn2329	pOP-EO04065_EST_C_1_pSK_SK	514
cl1977	ct2149	cn2329	pOP-EO04229_EST_C_1_pSK_SK	545
cl1977	ct2149	cn2329	pOP-EO06013_EST_C_1_pSK_SK	590
cl1977	ct2149	cn2329	pOP-EO06272_EST_C_1_pSK_SK	546
cl1977	ct2149	cn2329	pOP-EO06535_EST_C_1_pSK_SK	731
cl1977	ct2149	cn2329	pOP-EO07742_EST_C_1_pSK_SK	678
cl1977	ct2149	cn2329	pOP-EO08101_EST_C_1_pSK_SK	598
cl1977	ct2150	cn2330	pOP-CEO02941_EST_C_1_pSK_SK	344
cl1977	ct2150	cn2330	pOP-CNI01295_EST_C_1_pSK_SK	350
cl1977	ct2150	cn2330	pOP-EAP02830_EST_C_1_pBSK_SK	356
cl1977	ct2150	cn2330	pOP-EAP03458_EST_C_1_pBSK_SK	294
cl1977	ct2150	cn2330	pOP-EAP05019_EST_C_1_pBSK_SK	221
cl1977	ct2150	cn2330	pOP-EO02828_EST_C_1_pSK_SK	396
cl1977	ct2150	cn2330	pOP-EO03131_EST_C_1_pSK_SK	392
cl1977	ct2150	cn2330	pOP-EO03356_EST_C_1_pSK_SK	482
cl1977	ct2150	cn2330	pOP-EO03406_EST_C_1_pSK_SK	344
cl1977	ct2150	cn2330	pOP-EO03446_EST_C_1_pSK_SK	535
cl1977	ct2150	cn2330	pOP-EO03578_EST_C_1_pSK_SK	347
cl1977	ct2150	cn2330	pOP-EO03691_EST_C_1_pSK_SK	364
cl1977	ct2150	cn2330	pOP-EO03694_EST_C_1_pSK_SK	363
cl1977	ct2150	cn2330	pOP-EO03727_EST_C_1_pSK_SK	388
cl1977	ct2150	cn2330	pOP-EO03866_EST_C_1_pSK_SK	519
cl1977	ct2150	cn2330	pOP-EO03867_EST_C_1_pSK_SK	243
cl1977	ct2150	cn2330	pOP-EO04269_EST_C_1_pSK_SK	385
cl1977	ct2150	cn2330	pOP-EO04344_EST_C_1_pSK_SK	510
cl1977	ct2150	cn2330	pOP-EO04346_EST_C_1_pSK_SK	510
cl1977	ct2150	cn2330	pOP-EO04581_EST_C_1_pSK_SK	516
cl1977	ct2150	cn2330	pOP-EO04891_EST_C_1_pSK_SK	513
cl1977	ct2150	cn2331	pOP-CEO02906_EST_C_1_pSK_SK	578
cl1977	ct2150	cn2332	pOP-EO03015_EST_C_1_pSK_SK	396
cl1978	ct2151	cn2333	pOP-EO04051_EST_C_1_pSK_SK	492
cl1978	ct2151	cn2333	pOP-EO04586_EST_C_1_pSK_SK	390

cl1979	ct2152	cn2334	pOP-CEM00125_EST_C_1_pSK_SK	392
cl1979	ct2152	cn2334	pOP-EAP01131_EST_C_1_pBSK_SK	222
cl1979	ct2152	cn2334	pOP-EAP02796_EST_C_1_pBSK_SK	566
cl1979	ct2152	cn2334	pOP-EO04590_EST_C_1_pSK_SK	543
cl1980	ct2153	cn2335	pOP-EO04116_EST_C_1_pSK_SK	514
cl1980	ct2153	cn2335	pOP-EO04591_EST_C_1_pSK_SK	511
cl1981	ct2154	cn2336	pOP-CAP00416_EST_C_1_pBSK_SK	570
cl1981	ct2154	cn2336	pOP-CNH01317_EST_C_1_pSK_SK	577
cl1981	ct2154	cn2336	pOP-CNH01350_EST_C_1_pSK_SK	591
cl1981	ct2154	cn2336	pOP-CNHP00024_EST_C_1_pSK_SK	661
cl1981	ct2154	cn2336	pOP-EO02713_EST_C_1_pSK_SK	451
cl1981	ct2154	cn2336	pOP-EO04592_EST_C_1_pSK_SK	527
cl1981	ct2154	cn2336	pOP-EO06268_EST_C_1_pSK_SK	553
cl1982	ct2155	cn2337	pOP-EO03224_EST_C_1_pSK_SK	474
cl1982	ct2155	cn2337	pOP-EO04595_EST_C_1_pSK_SK	511
cl1983	ct2156	cn2338	pOP-CNH03736_EST_C_1_pSK_SK	423
cl1983	ct2156	cn2338	pOP-CNH04734_EST_C_1_pSK_SK	345
cl1983	ct2156	cn2338	pOP-EAP03686_EST_C_1_pBSK_SK	573
cl1983	ct2156	cn2338	pOP-EO04504_EST_C_1_pSK_SK	491
cl1983	ct2156	cn2338	pOP-EO04598_EST_C_1_pSK_SK	549
cl1984	ct2157	cn2339	pOP-EAP03786_EST_C_1_pBSK_SK	424
cl1984	ct2157	cn2340	pOP-EO04603_EST_C_1_pSK_SK	298
cl1985	ct2158	cn2341	pOP-CNH02312_EST_C_1_pSK_SK	414
cl1985	ct2158	cn2341	pOP-EO04605_EST_C_1_pSK_SK	552
cl1986	ct2159	cn2342	pOP-EO03606_EST_C_1_pSK_SK	340
cl1986	ct2159	cn2342	pOP-EO04607_EST_C_1_pSK_SK	552
cl1987	ct2160	cn2343	pOP-EO04608_EST_C_1_pSK_SK	539
cl1987	ct2161	cn2344	pOP-EAP00824_EST_C_1_pBSK_SK	407
cl1987	ct2161	cn2344	pOP-EO05229_EST_C_1_pSK_SK	487
cl1988	ct2162	cn2345	pOP-CNHP00523_EST_C_1_pSK_SK	418
cl1988	ct2162	cn2345	pOP-CNIP00246_EST_C_1_pSK_SK	393
cl1988	ct2162	cn2345	pOP-EO04610_EST_C_1_pSK_SK	551
cl1989	ct2163	cn2346	pOP-CNH02448_EST_C_1_pSK_SK	520
cl1989	ct2163	cn2346	pOP-EO04618_EST_C_1_pSK_SK	547
cl1990	ct2164	cn2347	pOP-CNH02502_EST_C_1_pSK_SK	495
cl1990	ct2164	cn2347	pOP-EO04623_EST_C_1_pSK_SK	507
cl1991	ct2165	cn2348	pOP-EO04271_EST_C_1_pSK_SK	446
cl1991	ct2165	cn2348	pOP-EO04628_EST_C_1_pSK_SK	530
cl1992	ct2166	cn2349	pOP-EAP00951_EST_C_1_pBSK_SK	345
cl1992	ct2166	cn2349	pOP-EO04633_EST_C_1_pSK_SK	529
cl1993	ct2167	cn2350	pOP-CNH02196_EST_C_1_pSK_SK	718
cl1993	ct2167	cn2350	pOP-EO04638_EST_C_1_pSK_SK	536
cl1994	ct2168	cn2351	pOP-CEO02483_EST_C_1_pSK_SK	534
cl1994	ct2168	cn2351	pOP-CNIP04024_EST_C_1_pSK_SK	339
cl1994	ct2168	cn2351	pOP-EAP02954_EST_C_1_pBSK_SK	596
cl1994	ct2168	cn2351	pOP-EO02157_EST_C_1_pSK_SK	634
cl1994	ct2168	cn2351	pOP-EO04033_EST_C_1_pSK_SK	544
cl1994	ct2168	cn2351	pOP-EO04130_EST_C_1_pSK_SK	321
cl1994	ct2168	cn2351	pOP-EO04639_EST_C_1_pSK_SK	527
cl1995	ct2169	cn2352	pOP-EO04640_EST_C_1_pSK_SK	533
cl1995	ct2169	cn2352	pOP-EO07232_EST_C_1_pSK_SK	702
cl1996	ct2170	cn2353	pOP-EO07290_EST_C_1_pSK_SK	595
cl1996	ct2170	cn2354	pOP-EO04644_EST_C_1_pSK_SK	525
cl1997	ct2171	cn2355	pOP-CNI02139_EST_C_1_pSK_SK	304
cl1997	ct2171	cn2355	pOP-EO04645_EST_C_1_pSK_SK	501
cl1998	ct2172	cn2356	pOP-EAP00553_EST_C_1_pBSK_SK	634

cl1998	ct2173	cn2357	pOP-CEM00078_EST_C_1_pSK_SK	399
cl1998	ct2173	cn2357	pOP-CNIP00588_EST_C_1_pSK_SK	446
cl1998	ct2173	cn2357	pOP-EO04652_EST_C_1_pSK_SK	537
cl1999	ct2174	cn2358	pOP-CAP00205_EST_C_1_pBSK_SK	608
cl1999	ct2174	cn2358	pOP-CEO03625_EST_C_1_pSK_SK	345
cl1999	ct2174	cn2358	pOP-CNH03381_EST_C_1_pSK_SK	659
cl1999	ct2174	cn2358	pOP-EO04656_EST_C_1_pSK_SK	524
cl2000	ct2175	cn2359	pOP-CNI01265_EST_C_1_pSK_SK	435
cl2000	ct2175	cn2359	pOP-EAP02319_EST_C_1_pBSK_SK	674
cl2000	ct2175	cn2359	pOP-EO04659_EST_C_1_pSK_SK	534
cl2001	ct2176	cn2360	pOP-EAP02919_EST_C_1_pBSK_SK	610
cl2001	ct2176	cn2360	pOP-EO04661_EST_C_1_pSK_SK	525
cl2002	ct2177	cn2361	pOP-EN00455_EST_C_1_pSK_SK	513
cl2002	ct2177	cn2361	pOP-EO04663_EST_C_1_pSK_SK	531
cl2003	ct2178	cn2362	pOP-CEO02905_EST_C_1_pSK_SK	268
cl2003	ct2178	cn2362	pOP-CEO03072_EST_C_1_pSK_SK	570
cl2003	ct2178	cn2362	pOP-CNHP00282_EST_C_1_pSK_SK	366
cl2003	ct2178	cn2362	pOP-CNI02021_EST_C_1_pSK_SK	477
cl2003	ct2178	cn2362	pOP-EO02948_EST_C_1_pSK_SK	407
cl2003	ct2178	cn2362	pOP-EO03447_EST_C_1_pSK_SK	484
cl2003	ct2179	cn2363	pOP-CEO01570_EST_C_1_pSK_SK	417
cl2003	ct2179	cn2363	pOP-CEO02024_EST_C_1_pSK_SK	382
cl2003	ct2179	cn2363	pOP-EAP00796_EST_C_1_pBSK_SK	379
cl2003	ct2179	cn2363	pOP-EO04424_EST_C_1_pSK_SK	529
cl2003	ct2179	cn2363	pOP-EO04444_EST_C_1_pSK_SK	482
cl2003	ct2179	cn2363	pOP-EO04666_EST_C_1_pSK_SK	532
cl2004	ct2180	cn2364	pOP-EO04670_EST_C_1_pSK_SK	355
cl2004	ct2180	cn2364	pOP-EO06076_EST_C_1_pSK_SK	664
cl2005	ct2181	cn2365	pOP-CNH00947_EST_C_1_pSK_SK	591
cl2005	ct2181	cn2365	pOP-CNH03064_EST_C_1_pSK_SK	676
cl2005	ct2181	cn2366	pOP-EO04674_EST_C_1_pSK_SK	532
cl2006	ct2182	cn2367	pOP-CNH02497_EST_C_1_pSK_SK	521
cl2006	ct2182	cn2367	pOP-CNH04797_EST_C_1_pSK_SK	338
cl2006	ct2182	cn2367	pOP-EAP02742_EST_C_1_pBSK_SK	703
cl2006	ct2182	cn2367	pOP-EO04680_EST_C_1_pSK_SK	538
cl2007	ct2183	cn2368	pOP-CEO02189_EST_C_1_pSK_SK	667
cl2007	ct2183	cn2368	pOP-EAP00672_EST_C_1_pBSK_SK	665
cl2007	ct2183	cn2368	pOP-EAP01893_EST_C_1_pBSK_SK	478
cl2007	ct2183	cn2368	pOP-EO04685_EST_C_1_pSK_SK	532
cl2008	ct2184	cn2369	pOP-CEO02709_EST_C_1_pSK_SK	347
cl2008	ct2184	cn2369	pOP-EAP01508_EST_C_1_pBSK_SK	618
cl2008	ct2184	cn2369	pOP-EO04686_EST_C_1_pSK_SK	528
cl2009	ct2185	cn2370	pOP-CNIP00207_EST_C_1_pSK_SK	236
cl2009	ct2185	cn2370	pOP-EAP02389_EST_C_1_pBSK_SK	209
cl2009	ct2185	cn2370	pOP-EO04696_EST_C_1_pSK_SK	535
cl2010	ct2186	cn2371	pOP-CNH02025_EST_C_1_pSK_SK	529
cl2010	ct2187	cn2372	pOP-CEO02764_EST_C_1_pSK_SK	339
cl2010	ct2187	cn2372	pOP-CNH03054_EST_C_1_pSK_SK	548
cl2010	ct2187	cn2372	pOP-CNH04469	785
cl2010	ct2187	cn2372	pOP-EO02766_EST_C_1_pSK_SK	443
cl2010	ct2187	cn2372	pOP-EO04703_EST_C_1_pSK_SK	528
cl2011	ct2188	cn2373	pOP-CNIP00999_EST_C_1_pSK_SK	299
cl2011	ct2188	cn2373	pOP-EO04716_EST_C_1_pSK_SK	537
cl2012	ct2189	cn2374	pOP-EO04097_EST_C_1_pSK_SK	535
cl2012	ct2189	cn2374	pOP-EO04207_EST_C_1_pSK_SK	488
cl2012	ct2189	cn2374	pOP-EO04719_EST_C_1_pSK_SK	482

cl2013	ct2190	cn2375	pOP-CBP00270_EST_C_1_pBSK_SK	512
cl2013	ct2190	cn2375	pOP-CNH00594_EST_C_1_pSK_SK	591
cl2013	ct2190	cn2375	pOP-CNIP00258_EST_C_1_pSK_SK	276
cl2013	ct2190	cn2375	pOP-EAP02973_EST_C_1_pBSK_SK	370
cl2013	ct2190	cn2375	pOP-EAP03461_EST_C_1_pBSK_SK	509
cl2013	ct2190	cn2375	pOP-EAP03611_EST_C_1_pBSK_SK	301
cl2013	ct2190	cn2375	pOP-EO02497_EST_C_1_pSK_SK	516
cl2013	ct2190	cn2375	pOP-EO04733_EST_C_1_pSK_SK	495
cl2014	ct2191	cn2376	pOP-CNH04931_EST_C_1_pSK_SK	751
cl2014	ct2191	cn2376	pOP-EO04739_EST_C_1_pSK_SK	439
cl2015	ct2192	cn2377	pOP-CEO01790_EST_C_1_pSK_SK	108
cl2015	ct2192	cn2377	pOP-CEO01792_EST_C_1_pSK_SK	108
cl2015	ct2192	cn2377	pOP-EO06581_EST_C_1_pSK_SK	407
cl2015	ct2193	cn2378	pOP-CEO02181_EST_C_1_pSK_SK	406
cl2015	ct2193	cn2378	pOP-CNI02089_EST_C_1_pSK_SK	558
cl2015	ct2193	cn2378	pOP-CNIP00534_EST_C_1_pSK_SK	434
cl2015	ct2193	cn2378	pOP-EAP01953_EST_C_1_pBSK_SK	547
cl2015	ct2194	cn2379	pOP-CEO01107_EST_C_1_pSK_SK	337
cl2015	ct2194	cn2379	pOP-CEO02418_EST_C_1_pSK_SK	373
cl2015	ct2194	cn2379	pOP-CEO03329_EST_C_1_pSK_SK	160
cl2015	ct2194	cn2379	pOP-CEO03425_EST_C_1_pSK_SK	495
cl2015	ct2194	cn2379	pOP-CEO03688_EST_C_1_pSK_SK	353
cl2015	ct2194	cn2379	pOP-CNI01558_EST_C_1_pSK_SK	216
cl2015	ct2194	cn2379	pOP-EAP00118_EST_C_1_pBSK_SK	640
cl2015	ct2194	cn2379	pOP-EAP02129_EST_C_1_pBSK_SK	371
cl2015	ct2194	cn2379	pOP-EAP03877_EST_C_1_pBSK_SK	626
cl2015	ct2194	cn2379	pOP-EO04741_EST_C_1_pSK_SK	524
cl2016	ct2195	cn2380	pOP-EO03968_EST_C_1_pSK_SK	488
cl2016	ct2195	cn2380	pOP-EO04744_EST_C_1_pSK_SK	466
cl2017	ct2196	cn2381	pOP-CBP00111_EST_C_1_pBSK_SK	496
cl2017	ct2196	cn2381	pOP-EO05304_EST_C_1_pSK_SK	527
cl2017	ct2196	cn2381	pOP-EO07314_EST_C_1_pSK_SK	693
cl2017	ct2197	cn2382	pOP-EN00434_EST_C_1_pSK_SK	516
cl2017	ct2197	cn2382	pOP-EO02702_EST_C_1_pSK_SK	317
cl2017	ct2197	cn2382	pOP-EO03429_EST_C_1_pSK_SK	512
cl2017	ct2197	cn2382	pOP-EO04745_EST_C_1_pSK_SK	452
cl2017	ct2197	cn2382	pOP-EO04940_EST_C_1_pSK_SK	511
cl2017	ct2197	cn2382	pOP-EO08276_EST_C_1_pSK_SK	545
cl2017	ct2197	cn2382	pOP-EO08396_EST_C_1_pSK_SK	335
cl2017	ct2197	cn2382	pOP-EO08430_EST_C_1_pSK_SK	381
cl2018	ct2198	cn2383	pOP-CNH02307_EST_C_1_pSK_SK	587
cl2018	ct2198	cn2383	pOP-EO02222_EST_C_1_pSK_SK	647
cl2018	ct2198	cn2383	pOP-EO04747_EST_C_1_pSK_SK	530
cl2019	ct2199	cn2384	pOP-EAP02760_EST_C_1_pBSK_SK	640
cl2019	ct2199	cn2384	pOP-EO02877_EST_C_1_pSK_SK	298
cl2019	ct2199	cn2384	pOP-EO03499_EST_C_1_pSK_SK	534
cl2019	ct2199	cn2384	pOP-EO04751_EST_C_1_pSK_SK	351
cl2020	ct2200	cn2385	pOP-CBP00106_EST_C_1_pBSK_SK	597
cl2020	ct2200	cn2385	pOP-CEO02132_EST_C_1_pSK_SK	322
cl2020	ct2200	cn2385	pOP-EO03770_EST_C_1_pSK_SK	532
cl2020	ct2200	cn2385	pOP-EO04082_EST_C_1_pSK_SK	360
cl2020	ct2200	cn2385	pOP-EO04194_EST_C_1_pSK_SK	423
cl2020	ct2200	cn2385	pOP-EO04757_EST_C_1_pSK_SK	523
cl2021	ct2201	cn2386	pOP-EAP03456_EST_C_1_pBSK_SK	268
cl2021	ct2201	cn2386	pOP-EO04288_EST_C_1_pSK_SK	398
cl2021	ct2201	cn2386	pOP-EO04763_EST_C_1_pSK_SK	522

cl2022	ct2202	cn2387	pOP-CAP00299_EST_C_1_pBSK_SK	576
cl2022	ct2202	cn2387	pOP-CNIP00539_EST_C_1_pSK_SK	323
cl2022	ct2202	cn2387	pOP-EO04772_EST_C_1_pSK_SK	511
cl2023	ct2203	cn2388	pOP-CEO02175_EST_C_1_pSK_SK	608
cl2023	ct2203	cn2388	pOP-EO04797_EST_C_1_pSK_SK	561
cl2024	ct2204	cn2389	pOP-CNI01507_EST_C_1_pSK_SK	438
cl2024	ct2204	cn2389	pOP-CNIP00118_EST_C_1_pSK_SK	358
cl2024	ct2204	cn2389	pOP-EO04542_EST_C_1_pSK_SK	497
cl2024	ct2204	cn2389	pOP-EO04804_EST_C_1_pSK_SK	569
cl2025	ct2205	cn2390	pOP-CNH00793_EST_C_1_pSK_SK	513
cl2025	ct2205	cn2390	pOP-CNIP00662_EST_C_1_pSK_SK	403
cl2025	ct2205	cn2390	pOP-EO04823_EST_C_1_pSK_SK	566
cl2026	ct2206	cn2391	pOP-EO03125_EST_C_1_pSK_SK	346
cl2026	ct2206	cn2391	pOP-EO04841_EST_C_1_pSK_SK	587
cl2027	ct2207	cn2392	pOP-EAP00270_EST_C_1_pBSK_SK	534
cl2027	ct2207	cn2392	pOP-EAP03616_EST_C_1_pBSK_SK	277
cl2027	ct2207	cn2392	pOP-EO04842_EST_C_1_pSK_SK	266
cl2028	ct2208	cn2393	pOP-CEO02786_EST_C_1_pSK_SK	455
cl2028	ct2208	cn2393	pOP-EAP02976_EST_C_1_pBSK_SK	467
cl2028	ct2208	cn2393	pOP-EO04845_EST_C_1_pSK_SK	461
cl2028	ct2208	cn2394	pOP-CEO03088_EST_C_1_pSK_SK	550
cl2029	ct2209	cn2395	pOP-EO03892_EST_C_1_pSK_SK	485
cl2029	ct2209	cn2395	pOP-EO04853_EST_C_1_pSK_SK	473
cl2030	ct2210	cn2396	pOP-CEO02318_EST_C_1_pSK_SK	324
cl2030	ct2211	cn2397	pOP-EO04867_EST_C_1_pSK_SK	511
cl2031	ct2212	cn2398	pOP-EAP03149_EST_C_1_pBSK_SK	605
cl2031	ct2212	cn2399	pOP-EO04873_EST_C_1_pSK_SK	506
cl2032	ct2213	cn2400	pOP-CEO03512_EST_C_1_pSK_SK	486
cl2032	ct2213	cn2400	pOP-EO04877_EST_C_1_pSK_SK	496
cl2033	ct2214	cn2401	pOP-CNHP00206_EST_C_1_pSK_SK	455
cl2033	ct2214	cn2401	pOP-EBP03098_EST_C_1_pBSK_M13F	165
cl2033	ct2214	cn2401	pOP-EN00390_EST_C_1_pSK_SK	512
cl2033	ct2214	cn2401	pOP-EO04880_EST_C_1_pSK_SK	511
cl2034	ct2215	cn2402	pOP-EO04700_EST_C_1_pSK_SK	530
cl2034	ct2215	cn2402	pOP-EO04883_EST_C_1_pSK_SK	507
cl2035	ct2216	cn2403	pOP-EO03281_EST_C_1_pSK_SK	398
cl2035	ct2216	cn2403	pOP-EO04902_EST_C_1_pSK_SK	498
cl2036	ct2217	cn2404	pOP-EO02875_EST_C_1_pSK_SK	378
cl2036	ct2217	cn2404	pOP-EO04817_EST_C_1_pSK_SK	557
cl2036	ct2217	cn2404	pOP-EO04926_EST_C_1_pSK_SK	526
cl2037	ct2218	cn2405	pOP-CNI01681_EST_C_1_pSK_SK	445
cl2037	ct2218	cn2405	pOP-EO04941_EST_C_1_pSK_SK	393
cl2038	ct2219	cn2406	pOP-EO07032_EST_C_1_pSK_SK	559
cl2038	ct2220	cn2407	pOP-EO02864_EST_C_1_pSK_SK	437
cl2038	ct2220	cn2407	pOP-EO04948_EST_C_1_pSK_SK	519
cl2039	ct2221	cn2408	pOP-EO04725_EST_C_1_pSK_SK	489
cl2039	ct2221	cn2408	pOP-EO04839_EST_C_1_pSK_SK	380
cl2039	ct2221	cn2408	pOP-EO04956_EST_C_1_pSK_SK	520
cl2040	ct2222	cn2409	pOP-EN00294_EST_C_1_pSK_SK	559
cl2040	ct2222	cn2409	pOP-EO02595_EST_C_1_pSK_SK	374
cl2040	ct2222	cn2409	pOP-EO04631_EST_C_1_pSK_SK	532
cl2040	ct2223	cn2410	pOP-CEO02243_EST_C_1_pSK_SK	442
cl2040	ct2223	cn2410	pOP-CNHP00058_EST_C_1_pSK_SK	570
cl2040	ct2223	cn2410	pOP-EO02374_EST_C_1_pSK_SK	751
cl2040	ct2223	cn2410	pOP-EO04957_EST_C_1_pSK_SK	521
cl2041	ct2224	cn2411	pOP-EO02207_EST_C_1_pSK_SK	573

cl2041	ct2224	cn2411	pOP-EO04967_EST_C_1_pSK_SK	524
cl2042	ct2225	cn2412	pOP-CEO01154_EST_C_1_pSK_SK	297
cl2042	ct2225	cn2412	pOP-CNIP00542_EST_C_1_pSK_SK	212
cl2042	ct2225	cn2412	pOP-EAP00846_EST_C_1_pBSK_SK	500
cl2042	ct2225	cn2412	pOP-EAP03669_EST_C_1_pBSK_SK	190
cl2042	ct2225	cn2412	pOP-EO03633_EST_C_1_pSK_SK	477
cl2042	ct2225	cn2412	pOP-EO04969_EST_C_1_pSK_SK	522
cl2043	ct2226	cn2413	pOP-CEO03099_EST_C_1_pSK_SK	640
cl2043	ct2226	cn2413	pOP-EO03225_EST_C_1_pSK_SK	476
cl2043	ct2226	cn2413	pOP-EO04971_EST_C_1_pSK_SK	520
cl2044	ct2227	cn2414	pOP-CEO01224_EST_C_1_pSK_SK	429
cl2044	ct2227	cn2414	pOP-CNH02013_EST_C_1_pSK_SK	520
cl2044	ct2227	cn2414	pOP-EO04032_EST_C_1_pSK_SK	547
cl2044	ct2227	cn2414	pOP-EO04220_EST_C_1_pSK_SK	541
cl2044	ct2227	cn2414	pOP-EO04221_EST_C_1_pSK_SK	108
cl2044	ct2227	cn2414	pOP-EO04465_EST_C_1_pSK_SK	453
cl2044	ct2227	cn2414	pOP-EO04981_EST_C_1_pSK_SK	520
cl2045	ct2228	cn2415	pOP-EAP01175_EST_C_1_pBSK_SK	480
cl2045	ct2228	cn2415	pOP-EAP02371_EST_C_1_pBSK_SK	353
cl2045	ct2228	cn2415	pOP-EO02945_EST_C_1_pSK_SK	381
cl2045	ct2228	cn2415	pOP-EO03571_EST_C_1_pSK_SK	554
cl2045	ct2228	cn2415	pOP-EO04983_EST_C_1_pSK_SK	522
cl2045	ct2228	cn2415	pOP-EO05673_EST_C_1_pSK_SK	354
cl2045	ct2228	cn2415	pOP-EO07393_EST_C_1_pSK_SK	688
cl2045	ct2228	cn2416	pOP-EO04087_EST_C_1_pSK_SK	436
cl2046	ct2229	cn2417	pOP-EAP01578_EST_C_1_pBSK_SK	469
cl2046	ct2229	cn2417	pOP-EO04996_EST_C_1_pSK_SK	509
cl2047	ct2230	cn2418	pOP-CAP00127_EST_C_1_pBSK_SK	615
cl2047	ct2230	cn2418	pOP-EO04999_EST_C_1_pSK_SK	508
cl2048	ct2231	cn2419	pOP-CNH00888_EST_C_1_pSK_SK	567
cl2048	ct2231	cn2419	pOP-CNH01322_EST_C_1_pSK_SK	726
cl2048	ct2231	cn2419	pOP-CNH01579_EST_C_1_pSK_SK	672
cl2048	ct2231	cn2419	pOP-EO05004_EST_C_1_pSK_SK	508
cl2049	ct2232	cn2420	pOP-EO04089_EST_C_1_pSK_SK	449
cl2049	ct2232	cn2420	pOP-EO04186_EST_C_1_pSK_SK	521
cl2049	ct2233	cn2421	pOP-CEO03024_EST_C_1_pSK_SK	199
cl2049	ct2233	cn2421	pOP-EO02367_EST_C_1_pSK_SK	713
cl2049	ct2233	cn2421	pOP-EO04833_EST_C_1_pSK_SK	493
cl2049	ct2233	cn2421	pOP-EO05005_EST_C_1_pSK_SK	522
cl2050	ct2234	cn2422	pOP-CEO02736_EST_C_1_pSK_SK	535
cl2050	ct2234	cn2422	pOP-CNH00614_EST_C_1_pSK_SK	715
cl2050	ct2234	cn2422	pOP-CNH03297_EST_C_1_pSK_SK	503
cl2050	ct2234	cn2422	pOP-CNI01741_EST_C_1_pSK_SK	404
cl2050	ct2234	cn2422	pOP-EO05019_EST_C_1_pSK_SK	520
cl2050	ct2234	cn2423	pOP-EO07334_EST_C_1_pSK_SK	697
cl2051	ct2235	cn2424	pOP-CNH00874_EST_C_1_pSK_SK	722
cl2051	ct2235	cn2424	pOP-CNIP04061_EST_C_1_pSK_SK	699
cl2051	ct2235	cn2424	pOP-EO04566_EST_C_1_pSK_SK	532
cl2051	ct2235	cn2424	pOP-EO05028_EST_C_1_pSK_SK	533
cl2052	ct2236	cn2425	pOP-EO04410_EST_C_1_pSK_SK	529
cl2052	ct2236	cn2425	pOP-EO05038_EST_C_1_pSK_SK	452
cl2053	ct2237	cn2426	pOP-EAP02993_EST_C_1_pBSK_SK	552
cl2053	ct2237	cn2426	pOP-EO05042_EST_C_1_pSK_SK	490
cl2054	ct2238	cn2427	pOP-EO05043_EST_C_1_pSK_SK	512
cl2054	ct2239	cn2428	pOP-CBP00061_EST_C_1_pBSK_SK	583
cl2054	ct2239	cn2428	pOP-CEO01241_EST_C_1_pSK_SK	302

cl2054	ct2239	cn2428	pOP-CEO01901_EST_C_1_pSK_SK	455
cl2054	ct2239	cn2428	pOP-CNH03550_EST_C_1_pSK_SK	494
cl2054	ct2239	cn2428	pOP-CNHP00474_EST_C_1_pSK_SK	408
cl2054	ct2239	cn2428	pOP-CNI02187_EST_C_1_pSK_SK	454
cl2055	ct2240	cn2429	pOP-EAP00710_EST_C_1_pBSK_SK	505
cl2055	ct2240	cn2429	pOP-EO05049_EST_C_1_pSK_SK	519
cl2056	ct2241	cn2430	pOP-EAP03365_EST_C_1_pBSK_SK	513
cl2056	ct2241	cn2430	pOP-EO03243_EST_C_1_pSK_SK	429
cl2056	ct2241	cn2430	pOP-EO05056_EST_C_1_pSK_SK	509
cl2057	ct2242	cn2431	pOP-CEO01809_EST_C_1_pSK_SK	360
cl2057	ct2242	cn2431	pOP-CNH01336_EST_C_1_pSK_SK	661
cl2057	ct2243	cn2432	pOP-CBP00065_EST_C_1_pBSK_SK	579
cl2057	ct2243	cn2432	pOP-CNIP04051_EST_C_1_pSK_SK	256
cl2057	ct2243	cn2432	pOP-EAP01040_EST_C_1_pBSK_SK	412
cl2057	ct2243	cn2432	pOP-EAP01452_EST_C_1_pBSK_SK	401
cl2057	ct2243	cn2432	pOP-EAP01832_EST_C_1_pBSK_SK	374
cl2057	ct2243	cn2432	pOP-EO04521_EST_C_1_pSK_SK	532
cl2057	ct2243	cn2432	pOP-EO05061_EST_C_1_pSK_SK	528
cl2058	ct2244	cn2433	pOP-CEM00036_EST_C_1_pSK_SK	149
cl2058	ct2244	cn2433	pOP-CEO01334	121
cl2058	ct2244	cn2433	pOP-EO04106_EST_C_1_pSK_SK	349
cl2058	ct2244	cn2433	pOP-EO05062_EST_C_1_pSK_SK	346
cl2059	ct2245	cn2434	pOP-CEO02239_EST_C_1_pSK_SK	218
cl2059	ct2245	cn2434	pOP-EO02956_EST_C_1_pSK_SK	398
cl2059	ct2245	cn2434	pOP-EO05063_EST_C_1_pSK_SK	505
cl2059	ct2245	cn2434	pOP-EO06114_EST_C_1_pSK_SK	590
cl2060	ct2246	cn2435	pOP-CEO01386_EST_C_1_pSK_SK	212
cl2060	ct2246	cn2435	pOP-EO05071_EST_C_1_pSK_SK	383
cl2061	ct2247	cn2436	pOP-CEO01475_EST_C_1_pSK_SK	149
cl2061	ct2247	cn2436	pOP-EO05074_EST_C_1_pSK_SK	533
cl2062	ct2248	cn2437	pOP-CEO01634_EST_C_1_pSK_SK	455
cl2062	ct2248	cn2437	pOP-CEO02201_EST_C_1_pSK_SK	697
cl2062	ct2248	cn2437	pOP-CEO02390_EST_C_1_pSK_SK	412
cl2062	ct2248	cn2437	pOP-CNIP04037_EST_C_1_pSK_SK	569
cl2062	ct2248	cn2437	pOP-EO05084_EST_C_1_pSK_SK	445
cl2062	ct2248	cn2438	pOP-CNI01714_EST_C_1_pSK_SK	508
cl2063	ct2249	cn2439	pOP-CNH00896_EST_C_1_pSK_SK	497
cl2063	ct2249	cn2439	pOP-EAP03332_EST_C_1_pBSK_SK	348
cl2063	ct2249	cn2439	pOP-EO05027_EST_C_1_pSK_SK	535
cl2063	ct2249	cn2439	pOP-EO05085_EST_C_1_pSK_SK	531
cl2064	ct2250	cn2440	pOP-EO03134_EST_C_1_pSK_SK	359
cl2064	ct2250	cn2440	pOP-EO05086_EST_C_1_pSK_SK	492
cl2065	ct2251	cn2441	pOP-EO03746_EST_C_1_pSK_SK	529
cl2065	ct2251	cn2441	pOP-EO05088_EST_C_1_pSK_SK	503
cl2066	ct2252	cn2442	pOP-EO03430_EST_C_1_pSK_SK	533
cl2066	ct2252	cn2442	pOP-EO05096_EST_C_1_pSK_SK	441
cl2067	ct2253	cn2443	pOP-EO03731_EST_C_1_pSK_SK	363
cl2067	ct2254	cn2444	pOP-CNIP00937_EST_C_1_pSK_SK	622
cl2067	ct2254	cn2444	pOP-EO04481_EST_C_1_pSK_SK	362
cl2067	ct2255	cn2445	pOP-CEO03043_EST_C_1_pSK_SK	372
cl2067	ct2255	cn2445	pOP-EAP02338_EST_C_1_pBSK_SK	657
cl2067	ct2256	cn2446	pOP-CEO01460_EST_C_1_pSK_SK	263
cl2067	ct2256	cn2446	pOP-EO02734_EST_C_1_pSK_SK	449
cl2067	ct2256	cn2446	pOP-EO02756_EST_C_1_pSK_SK	440
cl2067	ct2256	cn2446	pOP-EO03309_EST_C_1_pSK_SK	379
cl2067	ct2256	cn2446	pOP-EO04022_EST_C_1_pSK_SK	464

cl2067	ct2257	cn2447	pOP-EAP01547_EST_C_1_pBSK_SK	581
cl2067	ct2257	cn2447	pOP-EAP03463_EST_C_1_pBSK_SK	195
cl2067	ct2257	cn2447	pOP-EAP03648_EST_C_1_pBSK_SK	346
cl2067	ct2257	cn2447	pOP-EO02113_EST_C_1_pSK_SK	582
cl2067	ct2257	cn2447	pOP-EO02674_EST_C_1_pSK_SK	232
cl2067	ct2257	cn2447	pOP-EO02785_EST_C_1_pSK_SK	360
cl2067	ct2257	cn2447	pOP-EO03211_EST_C_1_pSK_SK	579
cl2067	ct2257	cn2447	pOP-EO03284_EST_C_1_pSK_SK	468
cl2067	ct2257	cn2447	pOP-EO03290_EST_C_1_pSK_SK	419
cl2067	ct2257	cn2447	pOP-EO03796_EST_C_1_pSK_SK	530
cl2067	ct2257	cn2447	pOP-EO03908_EST_C_1_pSK_SK	522
cl2067	ct2257	cn2447	pOP-EO04668_EST_C_1_pSK_SK	536
cl2067	ct2257	cn2447	pOP-EO04702_EST_C_1_pSK_SK	531
cl2067	ct2257	cn2447	pOP-EO04929_EST_C_1_pSK_SK	522
cl2067	ct2257	cn2447	pOP-EO05097_EST_C_1_pSK_SK	526
cl2067	ct2257	cn2447	pOP-EO05198_EST_C_1_pSK_SK	508
cl2067	ct2258	cn2448	pOP-CAP00342_EST_C_1_pBSK_SK	404
cl2067	ct2258	cn2448	pOP-CBP00202_EST_C_1_pBSK_SK	449
cl2067	ct2258	cn2448	pOP-CEO00904_EST_C_1_pSK_SK	372
cl2067	ct2258	cn2448	pOP-CEO02846_EST_C_1_pSK_SK	546
cl2067	ct2258	cn2448	pOP-CNH00767_EST_C_1_pSK_SK	574
cl2067	ct2258	cn2448	pOP-CNH01115_EST_C_1_pSK_SK	284
cl2067	ct2258	cn2448	pOP-CNH01181_EST_C_1_pSK_SK	582
cl2067	ct2258	cn2448	pOP-CNH01441_EST_C_1_pSK_SK	654
cl2067	ct2258	cn2448	pOP-CNHP00468_EST_C_1_pSK_SK	577
cl2067	ct2258	cn2448	pOP-CNI01647_EST_C_1_pSK_SK	267
cl2067	ct2258	cn2448	pOP-CNI01790_EST_C_1_pSK_SK	254
cl2067	ct2258	cn2448	pOP-CNI01858_EST_C_1_pSK_SK	389
cl2067	ct2258	cn2448	pOP-CNIP00304_EST_C_1_pSK_SK	302
cl2067	ct2258	cn2448	pOP-CNIP00486_EST_C_1_pSK_SK	422
cl2067	ct2258	cn2448	pOP-CNIP00927_EST_C_1_pSK_SK	716
cl2067	ct2258	cn2448	pOP-EAP02977_EST_C_1_pBSK_SK	353
cl2067	ct2258	cn2448	pOP-EAP03727_EST_C_1_pBSK_SK	502
cl2067	ct2258	cn2448	pOP-EO06287_EST_C_1_pSK_SK	623
cl2067	ct2258	cn2449	pOP-EAP00153_EST_C_1_pBSK_SK	671
cl2067	ct2258	cn2450	pOP-CNHP00186_EST_C_1_pSK_SK	559
cl2067	ct2259	cn2451	pOP-CAP00418_EST_C_1_pBSK_SK	667
cl2067	ct2259	cn2451	pOP-CEO01494_EST_C_1_pSK_SK	273
cl2067	ct2259	cn2451	pOP-CEO01752_EST_C_1_pSK_SK	186
cl2067	ct2259	cn2451	pOP-CEO01969_EST_C_1_pSK_SK	348
cl2067	ct2259	cn2451	pOP-CNH02026_EST_C_1_pSK_SK	507
cl2067	ct2259	cn2451	pOP-CNH02283_EST_C_1_pSK_SK	585
cl2067	ct2259	cn2451	pOP-CNH02356_EST_C_1_pSK_SK	659
cl2067	ct2259	cn2451	pOP-CNH02460_EST_C_1_pSK_SK	485
cl2067	ct2259	cn2451	pOP-CNH03676_EST_C_1_pSK_SK	431
cl2067	ct2259	cn2451	pOP-CNHP00208_EST_C_1_pSK_SK	351
cl2067	ct2259	cn2451	pOP-CNHP00283_EST_C_1_pSK_SK	330
cl2067	ct2259	cn2451	pOP-CNHP00328_EST_C_1_pSK_SK	621
cl2067	ct2259	cn2451	pOP-CNI01737_EST_C_1_pSK_SK	413
cl2067	ct2259	cn2451	pOP-CNI01742_EST_C_1_pSK_SK	570
cl2067	ct2259	cn2451	pOP-CNI01895_EST_C_1_pSK_SK	370
cl2067	ct2259	cn2451	pOP-CNI02022_EST_C_1_pSK_SK	425
cl2067	ct2259	cn2451	pOP-CNIP00110_EST_C_1_pSK_SK	528
cl2067	ct2259	cn2451	pOP-CNIP00114_EST_C_1_pSK_SK	588
cl2067	ct2259	cn2451	pOP-CNIP00138_EST_C_1_pSK_SK	474
cl2067	ct2259	cn2451	pOP-CNIP00391_EST_C_1_pSK_SK	435

cl2067	ct2259	cn2451	pOP-CNIP04073_EST_C_1_pSK_SK	273
cl2067	ct2259	cn2451	pOP-EO02064_EST_C_1_pSK_SK	496
cl2067	ct2259	cn2451	pOP-EO02324_EST_C_1_pSK_SK	501
cl2067	ct2259	cn2451	pOP-EO04890_EST_C_1_pSK_SK	513
cl2067	ct2259	cn2451	pOP-EO05204_EST_C_1_pSK_SK	529
cl2067	ct2259	cn2451	pOP-EO05379_EST_C_1_pSK_SK	464
cl2067	ct2259	cn2451	pOP-EO05561_EST_C_1_pSK_SK	382
cl2067	ct2259	cn2451	pOP-EO08472_EST_C_1_pSK_SK	257
cl2067	ct2259	cn2452	pOP-CNIP00811_EST_C_1_pSK_SK	721
cl2067	ct2259	cn2452	pOP-EO05457_EST_C_1_pSK_SK	507
cl2067	ct2259	cn2453	pOP-CNI01085_EST_C_1_pSK_SK	509
cl2067	ct2259	cn2454	pOP-EO02467_EST_C_1_pSK_SK	463
cl2067	ct2259	cn2455	pOP-CEO01165_EST_C_1_pSK_SK	395
cl2067	ct2259	cn2456	pOP-CEO02732_EST_C_1_pSK_SK	189
cl2067	ct2259	cn2456	pOP-CNIP00223_EST_C_1_pSK_SK	247
cl2067	ct2259	cn2456	pOP-EAP01171_EST_C_1_pBSK_SK	195
cl2067	ct2259	cn2456	pOP-EAP03397_EST_C_1_pBSK_SK	277
cl2067	ct2259	cn2457	pOP-CNH01155_EST_C_1_pSK_SK	260
cl2068	ct2260	cn2458	pOP-CEO02285_EST_C_1_pSK_SK	460
cl2068	ct2260	cn2458	pOP-EO05114_EST_C_1_pSK_SK	483
cl2069	ct2261	cn2459	pOP-EN00664_EST_C_1_pSK_SK	536
cl2069	ct2261	cn2459	pOP-EN00791_EST_C_1_pSK_SK	497
cl2069	ct2261	cn2459	pOP-EO05139_EST_C_1_pSK_SK	517
cl2070	ct2262	cn2460	pOP-EO03934_EST_C_1_pSK_SK	537
cl2070	ct2262	cn2460	pOP-EO05104_EST_C_1_pSK_SK	532
cl2070	ct2262	cn2460	pOP-EO05143_EST_C_1_pSK_SK	489
cl2071	ct2263	cn2461	pOP-CNH02454_EST_C_1_pSK_SK	539
cl2071	ct2263	cn2461	pOP-CNH03151_EST_C_1_pSK_SK	591
cl2071	ct2263	cn2461	pOP-EO05155_EST_C_1_pSK_SK	516
cl2072	ct2264	cn2462	pOP-EO02925_EST_C_1_pSK_SK	399
cl2072	ct2264	cn2462	pOP-EO04294_EST_C_1_pSK_SK	385
cl2072	ct2264	cn2462	pOP-EO05172_EST_C_1_pSK_SK	534
cl2073	ct2265	cn2463	pOP-CAP00266_EST_C_1_pBSK_SK	625
cl2073	ct2265	cn2463	pOP-CBP00104_EST_C_1_pBSK_SK	455
cl2073	ct2265	cn2463	pOP-EO03909_EST_C_1_pSK_SK	373
cl2073	ct2265	cn2463	pOP-EO05184_EST_C_1_pSK_SK	448
cl2074	ct2266	cn2464	pOP-CEO01800_EST_C_1_pSK_SK	350
cl2074	ct2266	cn2464	pOP-EO04562_EST_C_1_pSK_SK	544
cl2074	ct2266	cn2464	pOP-EO04563_EST_C_1_pSK_SK	545
cl2074	ct2266	cn2464	pOP-EO05185_EST_C_1_pSK_SK	535
cl2075	ct2267	cn2465	pOP-CEO02435_EST_C_1_pSK_SK	604
cl2075	ct2267	cn2465	pOP-CNIP00009_EST_C_1_pSK_SK	584
cl2075	ct2267	cn2465	pOP-CNIP00213_EST_C_1_pSK_SK	517
cl2075	ct2267	cn2465	pOP-EO05191_EST_C_1_pSK_SK	528
cl2076	ct2268	cn2466	pOP-CAP00048_EST_C_1_pBSK_SK	413
cl2076	ct2268	cn2466	pOP-CEO02397_EST_C_1_pSK_SK	630
cl2076	ct2268	cn2466	pOP-EO04526_EST_C_1_pSK_SK	476
cl2076	ct2268	cn2466	pOP-EO05203_EST_C_1_pSK_SK	475
cl2077	ct2269	cn2467	pOP-CNH00974_EST_C_1_pSK_SK	679
cl2077	ct2269	cn2467	pOP-CNH01984_EST_C_1_pSK_SK	727
cl2077	ct2269	cn2467	pOP-CNH03545_EST_C_1_pSK_SK	568
cl2077	ct2269	cn2467	pOP-CNH03669_EST_C_1_pSK_SK	428
cl2077	ct2269	cn2467	pOP-CNH04603	702
cl2077	ct2269	cn2467	pOP-EO05207_EST_C_1_pSK_SK	531
cl2078	ct2270	cn2468	pOP-CNHP00229_EST_C_1_pSK_SK	401
cl2078	ct2270	cn2468	pOP-EO05210_EST_C_1_pSK_SK	509

cl2078	ct2270	cn2468	pOP-EO07138_EST_C_1_pSK_SK	439
cl2078	ct2271	cn2469	pOP-CEO01275	311
cl2078	ct2271	cn2469	pOP-CEO02382_EST_C_1_pSK_SK	316
cl2078	ct2271	cn2469	pOP-CEO02704_EST_C_1_pSK_SK	378
cl2078	ct2271	cn2469	pOP-EO04442_EST_C_1_pSK_SK	527
cl2079	ct2272	cn2470	pOP-CEO03491_EST_C_1_pSK_SK	577
cl2079	ct2272	cn2470	pOP-EAP02229_EST_C_1_pBSK_SK	218
cl2079	ct2272	cn2471	pOP-CNH02000_EST_C_1_pSK_SK	552
cl2079	ct2273	cn2472	pOP-CEO02470_EST_C_1_pSK_SK	660
cl2079	ct2273	cn2472	pOP-CEO02922_EST_C_1_pSK_SK	402
cl2079	ct2273	cn2472	pOP-CEO03664_EST_C_1_pSK_SK	203
cl2079	ct2274	cn2473	pOP-CEM00245_EST_C_1_pSK_SK	241
cl2079	ct2274	cn2473	pOP-CEO00782_EST_C_1_pSK_SK	230
cl2079	ct2274	cn2473	pOP-CEO00784_EST_C_1_pSK_SK	226
cl2079	ct2274	cn2473	pOP-CEO01023_EST_C_1_pSK_SK	304
cl2079	ct2274	cn2473	pOP-CEO01495_EST_C_1_pSK_SK	237
cl2079	ct2274	cn2473	pOP-CEO02673_EST_C_1_pSK_SK	318
cl2079	ct2274	cn2473	pOP-EAP01243_EST_C_1_pBSK_SK	239
cl2079	ct2274	cn2473	pOP-EAP01316_EST_C_1_pBSK_SK	341
cl2079	ct2274	cn2473	pOP-EAP02140_EST_C_1_pBSK_SK	383
cl2079	ct2274	cn2473	pOP-EAP03147_EST_C_1_pBSK_SK	312
cl2079	ct2274	cn2473	pOP-EAP03283_EST_C_1_pBSK_SK	121
cl2079	ct2274	cn2473	pOP-EAP03472_EST_C_1_pBSK_SK	449
cl2079	ct2274	cn2473	pOP-EO02155_EST_C_1_pSK_SK	230
cl2079	ct2274	cn2473	pOP-EO02259_EST_C_1_pSK_SK	591
cl2079	ct2274	cn2473	pOP-EO02609_EST_C_1_pSK_SK	375
cl2079	ct2274	cn2473	pOP-EO02832_EST_C_1_pSK_SK	396
cl2079	ct2274	cn2473	pOP-EO02838_EST_C_1_pSK_SK	400
cl2079	ct2274	cn2473	pOP-EO02851_EST_C_1_pSK_SK	379
cl2079	ct2274	cn2473	pOP-EO02975_EST_C_1_pSK_SK	202
cl2079	ct2274	cn2473	pOP-EO03367_EST_C_1_pSK_SK	294
cl2079	ct2274	cn2473	pOP-EO03504_EST_C_1_pSK_SK	443
cl2079	ct2274	cn2473	pOP-EO03506_EST_C_1_pSK_SK	525
cl2079	ct2274	cn2473	pOP-EO03636_EST_C_1_pSK_SK	456
cl2079	ct2274	cn2473	pOP-EO03823_EST_C_1_pSK_SK	517
cl2079	ct2274	cn2473	pOP-EO04188_EST_C_1_pSK_SK	475
cl2079	ct2274	cn2473	pOP-EO04312_EST_C_1_pSK_SK	404
cl2079	ct2274	cn2473	pOP-EO04642_EST_C_1_pSK_SK	347
cl2079	ct2274	cn2473	pOP-EO04792_EST_C_1_pSK_SK	578
cl2079	ct2274	cn2473	pOP-EO04857_EST_C_1_pSK_SK	511
cl2079	ct2274	cn2473	pOP-EO04893_EST_C_1_pSK_SK	459
cl2079	ct2274	cn2473	pOP-EO04938_EST_C_1_pSK_SK	512
cl2079	ct2274	cn2473	pOP-EO05103_EST_C_1_pSK_SK	457
cl2079	ct2274	cn2473	pOP-EO05124_EST_C_1_pSK_SK	537
cl2079	ct2274	cn2473	pOP-EO05169_EST_C_1_pSK_SK	509
cl2079	ct2274	cn2473	pOP-EO05211_EST_C_1_pSK_SK	105
cl2079	ct2274	cn2473	pOP-EO05235_EST_C_1_pSK_SK	512
cl2079	ct2274	cn2473	pOP-EO05847_EST_C_1_pSK_SK	601
cl2079	ct2274	cn2473	pOP-EO05850_EST_C_1_pSK_SK	578
cl2079	ct2274	cn2473	pOP-EO06187_EST_C_1_pSK_SK	551
cl2079	ct2274	cn2473	pOP-EO06464_EST_C_1_pSK_SK	654
cl2079	ct2274	cn2473	pOP-EO06491_EST_C_1_pSK_SK	682
cl2079	ct2274	cn2473	pOP-EO06513_EST_C_1_pSK_SK	660
cl2079	ct2274	cn2473	pOP-EO06529_EST_C_1_pSK_SK	656
cl2079	ct2274	cn2473	pOP-EO06739_EST_C_1_pSK_SK	548
cl2079	ct2274	cn2473	pOP-EO07287_EST_C_1_pSK_SK	647

cl2079	ct2274	cn2473	pOP-EO07331_EST_C_1_pSK_SK	605
cl2079	ct2274	cn2473	pOP-EO08018_EST_C_1_pSK_SK	195
cl2079	ct2274	cn2473	pOP-EO08054_EST_C_1_pSK_SK	391
cl2079	ct2274	cn2473	pOP-EO08192_EST_C_1_pSK_SK	506
cl2079	ct2274	cn2473	pOP-EOP00007_EST_C_1_pSK_SK	600
cl2079	ct2274	cn2474	pOP-CEM00063_EST_C_1_pSK_SK	167
cl2079	ct2274	cn2474	pOP-CEM00068_EST_C_1_pSK_SK	159
cl2079	ct2274	cn2474	pOP-CEO03676_EST_C_1_pSK_SK	227
cl2079	ct2274	cn2474	pOP-EO05948_EST_C_1_pSK_SK	502
cl2080	ct2275	cn2475	pOP-CEO03172_EST_C_1_pSK_SK	172
cl2080	ct2275	cn2475	pOP-CNH04362	653
cl2080	ct2275	cn2475	pOP-CNIP00176_EST_C_1_pSK_SK	419
cl2080	ct2275	cn2475	pOP-EO05217_EST_C_1_pSK_SK	495
cl2081	ct2276	cn2476	pOP-EAP01594_EST_C_1_pBSK_SK	539
cl2081	ct2276	cn2476	pOP-EO05222_EST_C_1_pSK_SK	515
cl2082	ct2277	cn2477	pOP-CEO01174_EST_C_1_pSK_SK	438
cl2082	ct2277	cn2477	pOP-EO05225_EST_C_1_pSK_SK	519
cl2083	ct2278	cn2478	pOP-CBP00222_EST_C_1_pBSK_SK	479
cl2083	ct2278	cn2478	pOP-EO02132_EST_C_1_pSK_SK	252
cl2083	ct2278	cn2478	pOP-EO05237_EST_C_1_pSK_SK	533
cl2084	ct2279	cn2479	pOP-EO02230_EST_C_1_pSK_SK	404
cl2084	ct2279	cn2479	pOP-EO05224_EST_C_1_pSK_SK	533
cl2084	ct2279	cn2479	pOP-EO05289_EST_C_1_pSK_SK	523
cl2085	ct2280	cn2480	pOP-EO04257_EST_C_1_pSK_SK	331
cl2085	ct2280	cn2480	pOP-EO05296_EST_C_1_pSK_SK	510
cl2086	ct2281	cn2481	pOP-CNH01581_EST_C_1_pSK_SK	624
cl2086	ct2281	cn2481	pOP-CNH01757_EST_C_1_pSK_SK	481
cl2086	ct2281	cn2481	pOP-CNH01841_EST_C_1_pSK_SK	451
cl2086	ct2281	cn2481	pOP-CNH02605_EST_C_1_pSK_SK	663
cl2086	ct2281	cn2481	pOP-CNH02638_EST_C_1_pSK_SK	637
cl2086	ct2281	cn2481	pOP-CNH03263_EST_C_1_pSK_SK	598
cl2086	ct2281	cn2481	pOP-CNH04186	538
cl2086	ct2281	cn2481	pOP-CNH04213	582
cl2086	ct2281	cn2481	pOP-CNH04477	802
cl2086	ct2281	cn2481	pOP-CNH04677	643
cl2086	ct2281	cn2481	pOP-CNH04712_EST_C_1_pSK_SK	581
cl2086	ct2281	cn2481	pOP-CNHP00185_EST_C_1_pSK_SK	646
cl2086	ct2281	cn2481	pOP-CNIP00112_EST_C_1_pSK_SK	405
cl2086	ct2281	cn2481	pOP-EAP01352_EST_C_1_pBSK_SK	352
cl2086	ct2281	cn2481	pOP-EO05297_EST_C_1_pSK_SK	554
cl2087	ct2282	cn2482	pOP-EAP02986_EST_C_1_pBSK_SK	562
cl2087	ct2282	cn2482	pOP-EO05166_EST_C_1_pSK_SK	539
cl2087	ct2282	cn2482	pOP-EO05307_EST_C_1_pSK_SK	589
cl2087	ct2282	cn2483	pOP-CNI02198_EST_C_1_pSK_SK	441
cl2088	ct2283	cn2484	pOP-CNH00828_EST_C_1_pSK_SK	525
cl2088	ct2283	cn2484	pOP-CNI01804_EST_C_1_pSK_SK	521
cl2088	ct2283	cn2484	pOP-EO02659_EST_C_1_pSK_SK	409
cl2088	ct2283	cn2484	pOP-EO05309_EST_C_1_pSK_SK	570
cl2089	ct2284	cn2485	pOP-EN00795_EST_C_1_pSK_SK	477
cl2089	ct2284	cn2485	pOP-EO05303_EST_C_1_pSK_SK	369
cl2089	ct2284	cn2485	pOP-EO05314_EST_C_1_pSK_SK	556
cl2090	ct2285	cn2486	pOP-EO04008_EST_C_1_pSK_SK	460
cl2090	ct2285	cn2486	pOP-EO04163_EST_C_1_pSK_SK	377
cl2090	ct2285	cn2486	pOP-EO05322_EST_C_1_pSK_SK	487
cl2091	ct2286	cn2487	pOP-CEO01282	450
cl2091	ct2286	cn2487	pOP-CNH03466_EST_C_1_pSK_SK	526

cl2091	ct2286	cn2487	pOP-EO05325_EST_C_1_pSK_SK	564
cl2092	ct2287	cn2488	pOP-CEO01976_EST_C_1_pSK_SK	121
cl2092	ct2287	cn2488	pOP-EO05342_EST_C_1_pSK_SK	435
cl2093	ct2288	cn2489	pOP-EN00628_EST_C_1_pSK_SK	492
cl2093	ct2288	cn2489	pOP-EO05346_EST_C_1_pSK_SK	558
cl2094	ct2289	cn2490	pOP-CEO00939_EST_C_1_pSK_SK	571
cl2094	ct2289	cn2491	pOP-EO05348_EST_C_1_pSK_SK	508
cl2095	ct2290	cn2492	pOP-CNH01460_EST_C_1_pSK_SK	647
cl2095	ct2290	cn2492	pOP-CNH02987_EST_C_1_pSK_SK	574
cl2095	ct2290	cn2492	pOP-EN00452_EST_C_1_pSK_SK	493
cl2095	ct2290	cn2492	pOP-EO05349_EST_C_1_pSK_SK	555
cl2096	ct2291	cn2493	pOP-CEO01396_EST_C_1_pSK_SK	748
cl2096	ct2291	cn2493	pOP-CEOP00059_EST_C_1_pSK_SK	160
cl2096	ct2291	cn2493	pOP-EO05362_EST_C_1_pSK_SK	471
cl2097	ct2292	cn2494	pOP-CEO01856_EST_C_1_pSK_SK	526
cl2097	ct2292	cn2494	pOP-EO05363_EST_C_1_pSK_SK	417
cl2097	ct2292	cn2494	pOP-EO06160_EST_C_1_pSK_SK	507
cl2098	ct2293	cn2495	pOP-CAP00066_EST_C_1_pBSK_SK	519
cl2098	ct2293	cn2495	pOP-EO05369_EST_C_1_pSK_SK	550
cl2099	ct2294	cn2496	pOP-EAP00072_EST_C_1_pBSK_SK	593
cl2099	ct2294	cn2496	pOP-EAP03822_EST_C_1_pBSK_SK	513
cl2099	ct2294	cn2496	pOP-EO05376_EST_C_1_pSK_SK	559
cl2100	ct2295	cn2497	pOP-CNH01861_EST_C_1_pSK_SK	463
cl2100	ct2295	cn2497	pOP-CNH01909_EST_C_1_pSK_SK	625
cl2100	ct2295	cn2497	pOP-CNH02637_EST_C_1_pSK_SK	425
cl2100	ct2295	cn2497	pOP-CNH03390_EST_C_1_pSK_SK	521
cl2100	ct2295	cn2497	pOP-CNHP00351_EST_C_1_pSK_SK	596
cl2100	ct2295	cn2497	pOP-EAP02935_EST_C_1_pBSK_SK	688
cl2100	ct2295	cn2497	pOP-EO05385_EST_C_1_pSK_SK	426
cl2101	ct2296	cn2498	pOP-EO03112_EST_C_1_pSK_SK	166
cl2101	ct2296	cn2498	pOP-EO05391_EST_C_1_pSK_SK	553
cl2102	ct2297	cn2499	pOP-EO04556_EST_C_1_pSK_SK	473
cl2102	ct2297	cn2499	pOP-EO05399_EST_C_1_pSK_SK	477
cl2103	ct2298	cn2500	pOP-CEO03462_EST_C_1_pSK_SK	362
cl2103	ct2298	cn2500	pOP-EO05413_EST_C_1_pSK_SK	547
cl2104	ct2299	cn2501	pOP-EO03354_EST_C_1_pSK_SK	340
cl2104	ct2299	cn2501	pOP-EO05427_EST_C_1_pSK_SK	314
cl2105	ct2300	cn2502	pOP-CNH04672	166
cl2105	ct2300	cn2502	pOP-EO05437_EST_C_1_pSK_SK	259
cl2106	ct2301	cn2503	pOP-CEO03255_EST_C_1_pSK_SK	517
cl2106	ct2301	cn2503	pOP-CEO03547_EST_C_1_pSK_SK	539
cl2106	ct2301	cn2503	pOP-EAP01990_EST_C_1_pBSK_SK	415
cl2106	ct2301	cn2503	pOP-EO03341_EST_C_1_pSK_SK	464
cl2106	ct2301	cn2503	pOP-EO05442_EST_C_1_pSK_SK	296
cl2106	ct2301	cn2503	pOP-EO05461_EST_C_1_pSK_SK	490
cl2106	ct2301	cn2503	pOP-EO05490_EST_C_1_pSK_SK	424
cl2106	ct2301	cn2503	pOP-EO05732_EST_C_1_pSK_SK	556
cl2106	ct2301	cn2503	pOP-EO05783_EST_C_1_pSK_SK	530
cl2106	ct2301	cn2503	pOP-EO05946_EST_C_1_pSK_SK	350
cl2106	ct2301	cn2503	pOP-EO06837_EST_C_1_pSK_SK	533
cl2106	ct2301	cn2503	pOP-EO07648_EST_C_1_pSK_SK	676
cl2106	ct2301	cn2503	pOP-EO07929_EST_C_1_pSK_SK	760
cl2106	ct2301	cn2503	pOP-EO08096_EST_C_1_pSK_SK	495
cl2106	ct2301	cn2503	pOP-EO08120_EST_C_1_pSK_SK	487
cl2106	ct2301	cn2503	pOP-EO08179_EST_C_1_pSK_SK	567
cl2106	ct2301	cn2503	pOP-EO08198_EST_C_1_pSK_SK	652

cl2106	ct2301	cn2503	pOP-EO08281_EST_C_1_pSK_SK	611
cl2107	ct2302	cn2504	pOP-EO03516_EST_C_1_pSK_SK	433
cl2107	ct2302	cn2504	pOP-EO05463_EST_C_1_pSK_SK	511
cl2108	ct2303	cn2505	pOP-EAP02169_EST_C_1_pBSK_SK	244
cl2108	ct2303	cn2505	pOP-EO05467_EST_C_1_pSK_SK	313
cl2109	ct2304	cn2506	pOP-CAP00248_EST_C_1_pBSK_SK	584
cl2109	ct2304	cn2506	pOP-CEO00861_EST_C_1_pSK_SK	306
cl2109	ct2304	cn2506	pOP-CEO01920_EST_C_1_pSK_SK	429
cl2109	ct2304	cn2506	pOP-CEO03061_EST_C_1_pSK_SK	205
cl2109	ct2304	cn2506	pOP-EAP00484_EST_C_1_pBSK_SK	359
cl2109	ct2304	cn2506	pOP-EAP00485_EST_C_1_pBSK_SK	376
cl2109	ct2304	cn2506	pOP-EAP00501_EST_C_1_pBSK_SK	624
cl2109	ct2304	cn2506	pOP-EAP00547_EST_C_1_pBSK_SK	578
cl2109	ct2304	cn2506	pOP-EAP00648_EST_C_1_pBSK_SK	591
cl2109	ct2304	cn2506	pOP-EAP00703_EST_C_1_pBSK_SK	629
cl2109	ct2304	cn2506	pOP-EAP01187_EST_C_1_pBSK_SK	162
cl2109	ct2304	cn2506	pOP-EAP01467_EST_C_1_pBSK_SK	485
cl2109	ct2304	cn2506	pOP-EAP01728_EST_C_1_pBSK_SK	606
cl2109	ct2304	cn2506	pOP-EAP01799_EST_C_1_pBSK_SK	506
cl2109	ct2304	cn2506	pOP-EAP01958_EST_C_1_pBSK_SK	552
cl2109	ct2304	cn2506	pOP-EAP01991_EST_C_1_pBSK_SK	533
cl2109	ct2304	cn2506	pOP-EAP02348_EST_C_1_pBSK_SK	111
cl2109	ct2304	cn2506	pOP-EAP02349_EST_C_1_pBSK_SK	735
cl2109	ct2304	cn2506	pOP-EAP02925_EST_C_1_pBSK_SK	341
cl2109	ct2304	cn2506	pOP-EAP03406_EST_C_1_pBSK_SK	295
cl2109	ct2304	cn2506	pOP-EAP05018_EST_C_1_pBSK_SK	577
cl2109	ct2304	cn2506	pOP-EO03776_EST_C_1_pSK_SK	535
cl2109	ct2304	cn2506	pOP-EO03820_EST_C_1_pSK_SK	520
cl2109	ct2304	cn2506	pOP-EO03895_EST_C_1_pSK_SK	520
cl2109	ct2304	cn2506	pOP-EO03925_EST_C_1_pSK_SK	538
cl2109	ct2304	cn2506	pOP-EO03943_EST_C_1_pSK_SK	488
cl2109	ct2304	cn2506	pOP-EO03965_EST_C_1_pSK_SK	488
cl2109	ct2304	cn2506	pOP-EO04820_EST_C_1_pSK_SK	567
cl2109	ct2304	cn2506	pOP-EO05431_EST_C_1_pSK_SK	547
cl2109	ct2304	cn2506	pOP-EO05479_EST_C_1_pSK_SK	425
cl2109	ct2304	cn2506	pOP-EO05621_EST_C_1_pSK_SK	497
cl2109	ct2304	cn2506	pOP-EO06357_EST_C_1_pSK_SK	710
cl2109	ct2304	cn2506	pOP-EO06427_EST_C_1_pSK_SK	760
cl2109	ct2304	cn2506	pOP-EO06559_EST_C_1_pSK_SK	644
cl2109	ct2304	cn2506	pOP-EO06672_EST_C_1_pSK_SK	691
cl2109	ct2304	cn2506	pOP-EO06751_EST_C_1_pSK_SK	831
cl2109	ct2304	cn2506	pOP-EO06929_EST_C_1_pSK_SK	645
cl2109	ct2304	cn2506	pOP-EO07433_EST_C_1_pSK_SK	820
cl2109	ct2304	cn2506	pOP-EO07621_EST_C_1_pSK_SK	737
cl2109	ct2304	cn2506	pOP-EO07629_EST_C_1_pSK_SK	797
cl2109	ct2304	cn2506	pOP-EO08249_EST_C_1_pSK_SK	461
cl2109	ct2304	cn2507	pOP-CNIP00572_EST_C_1_pSK_SK	543
cl2109	ct2304	cn2508	pOP-CEO03736_EST_C_1_pSK_SK	103
cl2109	ct2304	cn2508	pOP-EO05246_EST_C_1_pSK_SK	475
cl2109	ct2304	cn2509	pOP-CEO02964_EST_C_1_pSK_SK	378
cl2109	ct2304	cn2510	pOP-EO02944_EST_C_1_pSK_SK	373
cl2109	ct2304	cn2511	pOP-CNI02234_EST_C_1_pSK_SK	153
cl2110	ct2305	cn2512	pOP-EN00699_EST_C_1_pSK_SK	511
cl2110	ct2305	cn2512	pOP-EO03794_EST_C_1_pSK_SK	490
cl2110	ct2305	cn2512	pOP-EO05484_EST_C_1_pSK_SK	441
cl2111	ct2306	cn2513	pOP-EO03097_EST_C_1_pSK_SK	482

cl2111	ct2306	cn2513	pOP-EO05499_EST_C_1_pSK_SK	331
cl2111	ct2306	cn2514	pOP-CEO00836_EST_C_1_pSK_SK	351
cl2112	ct2307	cn2515	pOP-EO02455_EST_C_1_pSK_SK	461
cl2112	ct2307	cn2515	pOP-EO05505_EST_C_1_pSK_SK	476
cl2114	ct2309	cn2517	pOP-EO02648_EST_C_1_pSK_SK	293
cl2114	ct2309	cn2517	pOP-EO05012_EST_C_1_pSK_SK	535
cl2114	ct2309	cn2517	pOP-EO05539_EST_C_1_pSK_SK	494
cl2115	ct2310	cn2518	pOP-CNIP00275_EST_C_1_pSK_SK	309
cl2115	ct2310	cn2518	pOP-EO02460_EST_C_1_pSK_SK	463
cl2115	ct2310	cn2518	pOP-EO04694_EST_C_1_pSK_SK	465
cl2115	ct2310	cn2518	pOP-EO05542_EST_C_1_pSK_SK	549
cl2115	ct2310	cn2518	pOP-EO07326_EST_C_1_pSK_SK	736
cl2115	ct2310	cn2518	pOP-EO08270_EST_C_1_pSK_SK	481
cl2116	ct2311	cn2519	pOP-CNI02007_EST_C_1_pSK_SK	513
cl2116	ct2311	cn2519	pOP-EAP01606_EST_C_1_pBSK_SK	477
cl2116	ct2311	cn2519	pOP-EO05549_EST_C_1_pSK_SK	501
cl2117	ct2312	cn2520	pOP-EAP00642_EST_C_1_pBSK_SK	574
cl2117	ct2312	cn2520	pOP-EAP03658_EST_C_1_pBSK_SK	435
cl2117	ct2312	cn2520	pOP-EO05552_EST_C_1_pSK_SK	326
cl2118	ct2313	cn2521	pOP-CEM00192_EST_C_1_pSK_SK	192
cl2118	ct2313	cn2521	pOP-CEO02082_EST_C_1_pSK_SK	349
cl2118	ct2313	cn2521	pOP-EO05574_EST_C_1_pSK_SK	503
cl2118	ct2313	cn2522	pOP-EO04386_EST_C_1_pSK_SK	511
cl2118	ct2313	cn2523	pOP-CNI02143_EST_C_1_pSK_SK	508
cl2118	ct2313	cn2524	pOP-CEO03113_EST_C_1_pSK_SK	357
cl2119	ct2314	cn2525	pOP-EO05737_EST_C_1_pSK_SK	563
cl2119	ct2314	cn2526	pOP-EO05584_EST_C_1_pSK_SK	489
cl2120	ct2315	cn2527	pOP-CEO02897_EST_C_1_pSK_SK	669
cl2120	ct2315	cn2527	pOP-CNI01148_EST_C_1_pSK_SK	278
cl2120	ct2316	cn2528	pOP-EAP03704_EST_C_1_pBSK_SK	308
cl2120	ct2316	cn2528	pOP-EO05594_EST_C_1_pSK_SK	434
cl2121	ct2317	cn2529	pOP-EO03545_EST_C_1_pSK_SK	328
cl2121	ct2317	cn2529	pOP-EO05613_EST_C_1_pSK_SK	331
cl2122	ct2318	cn2530	pOP-CEO02774_EST_C_1_pSK_SK	360
cl2122	ct2318	cn2530	pOP-CEO03261_EST_C_1_pSK_SK	507
cl2122	ct2318	cn2530	pOP-EAP01292_EST_C_1_pBSK_SK	270
cl2122	ct2318	cn2530	pOP-EO04697_EST_C_1_pSK_SK	488
cl2122	ct2318	cn2530	pOP-EO05052_EST_C_1_pSK_SK	525
cl2122	ct2318	cn2530	pOP-EO05614_EST_C_1_pSK_SK	366
cl2123	ct2319	cn2531	pOP-EO02662_EST_C_1_pSK_SK	318
cl2123	ct2319	cn2531	pOP-EO05623_EST_C_1_pSK_SK	472
cl2124	ct2320	cn2532	pOP-CEO01552_EST_C_1_pSK_SK	458
cl2124	ct2320	cn2532	pOP-EO02589_EST_C_1_pSK_SK	479
cl2124	ct2320	cn2532	pOP-EO05628_EST_C_1_pSK_SK	479
cl2125	ct2321	cn2533	pOP-CEO02487_EST_C_1_pSK_SK	521
cl2125	ct2321	cn2533	pOP-CEO03536_EST_C_1_pSK_SK	681
cl2125	ct2321	cn2533	pOP-EO05635_EST_C_1_pSK_SK	556
cl2126	ct2322	cn2534	pOP-ENP00009_EST_C_1_pSK_SK	248
cl2126	ct2322	cn2534	pOP-EO05637_EST_C_1_pSK_SK	535
cl2127	ct2323	cn2535	pOP-CEO02751_EST_C_1_pSK_SK	330
cl2127	ct2323	cn2535	pOP-EO08091_EST_C_1_pSK_SK	577
cl2127	ct2323	cn2536	pOP-CEO01702_EST_C_1_pSK_SK	522
cl2127	ct2324	cn2537	pOP-CEM00228_EST_C_1_pSK_SK	127
cl2127	ct2324	cn2537	pOP-CEO03189_EST_C_1_pSK_SK	490
cl2127	ct2324	cn2537	pOP-CNH03649_EST_C_1_pSK_SK	379
cl2127	ct2324	cn2537	pOP-CNI01949_EST_C_1_pSK_SK	488

cl2127	ct2324	cn2537	pOP-CNI02216_EST_C_1_pSK_SK	341
cl2127	ct2324	cn2537	pOP-CNIP00943_EST_C_1_pSK_SK	183
cl2127	ct2324	cn2537	pOP-EO02029_EST_C_1_pSK_SK	413
cl2127	ct2324	cn2537	pOP-EO04105_EST_C_1_pSK_SK	706
cl2127	ct2324	cn2537	pOP-EO04864_EST_C_1_pSK_SK	509
cl2127	ct2324	cn2537	pOP-EO05044_EST_C_1_pSK_SK	539
cl2127	ct2324	cn2537	pOP-EO05646_EST_C_1_pSK_SK	468
cl2127	ct2324	cn2537	pOP-EO06896_EST_C_1_pSK_SK	723
cl2128	ct2325	cn2538	pOP-CAP00353_EST_C_1_pBSK_SK	324
cl2128	ct2325	cn2538	pOP-CEO03691_EST_C_1_pSK_SK	407
cl2128	ct2325	cn2538	pOP-EO02205_EST_C_1_pSK_SK	630
cl2128	ct2325	cn2538	pOP-EO05668_EST_C_1_pSK_SK	413
cl2128	ct2325	cn2539	pOP-EO05383_EST_C_1_pSK_SK	548
cl2129	ct2326	cn2540	pOP-CEO03711_EST_C_1_pSK_SK	273
cl2129	ct2326	cn2540	pOP-CNI01210_EST_C_1_pSK_SK	273
cl2129	ct2326	cn2540	pOP-CNLP00001_EST_C_1_pSK_SK	258
cl2129	ct2326	cn2540	pOP-EO03234_EST_C_1_pSK_SK	372
cl2129	ct2326	cn2540	pOP-EO05671_EST_C_1_pSK_SK	428
cl2129	ct2326	cn2540	pOP-EO07343_EST_C_1_pSK_SK	641
cl2130	ct2327	cn2541	pOP-EN00441_EST_C_1_pSK_SK	513
cl2130	ct2327	cn2541	pOP-EO05684_EST_C_1_pSK_SK	443
cl2131	ct2328	cn2542	pOP-CEO01438_EST_C_1_pSK_SK	468
cl2131	ct2328	cn2542	pOP-CEO02110_EST_C_1_pSK_SK	231
cl2131	ct2328	cn2542	pOP-CEO03760_EST_C_1_pSK_SK	205
cl2131	ct2328	cn2542	pOP-EAP05026_EST_C_1_pBSK_SK	589
cl2131	ct2328	cn2542	pOP-EO02042_EST_C_1_pSK_SK	678
cl2131	ct2328	cn2542	pOP-EO05686_EST_C_1_pSK_SK	453
cl2132	ct2329	cn2543	pOP-EO05495_EST_C_1_pSK_SK	490
cl2132	ct2329	cn2543	pOP-EO05704_EST_C_1_pSK_SK	507
cl2133	ct2330	cn2544	pOP-EO02508_EST_C_1_pSK_SK	530
cl2133	ct2330	cn2544	pOP-EO02516_EST_C_1_pSK_SK	530
cl2133	ct2330	cn2544	pOP-EO05716_EST_C_1_pSK_SK	486
cl2133	ct2330	cn2545	pOP-EAP01346_EST_C_1_pBSK_SK	303
cl2134	ct2331	cn2546	pOP-CNH02598_EST_C_1_pSK_SK	542
cl2134	ct2331	cn2546	pOP-EAP01284_EST_C_1_pBSK_SK	273
cl2134	ct2331	cn2546	pOP-EO05726_EST_C_1_pSK_SK	433
cl2135	ct2332	cn2547	pOP-EAP02901_EST_C_1_pBSK_SK	589
cl2135	ct2332	cn2547	pOP-EO05731_EST_C_1_pSK_SK	561
cl2136	ct2333	cn2548	pOP-EO05736_EST_C_1_pSK_SK	555
cl2136	ct2333	cn2549	pOP-EAP01479_EST_C_1_pBSK_SK	425
cl2137	ct2334	cn2550	pOP-EO02310_EST_C_1_pSK_SK	702
cl2137	ct2334	cn2551	pOP-EO05750_EST_C_1_pSK_SK	495
cl2138	ct2335	cn2552	pOP-EAP02347_EST_C_1_pBSK_SK	671
cl2138	ct2335	cn2552	pOP-EO05753_EST_C_1_pSK_SK	665
cl2139	ct2336	cn2553	pOP-CEO01307	182
cl2139	ct2336	cn2553	pOP-EO05755_EST_C_1_pSK_SK	475
cl2140	ct2337	cn2554	pOP-EO05356_EST_C_1_pSK_SK	526
cl2140	ct2337	cn2554	pOP-EO05756_EST_C_1_pSK_SK	457
cl2141	ct2338	cn2555	pOP-EO03452_EST_C_1_pSK_SK	534
cl2141	ct2338	cn2555	pOP-EO05758_EST_C_1_pSK_SK	551
cl2142	ct2339	cn2556	pOP-EO03956_EST_C_1_pSK_SK	489
cl2142	ct2339	cn2556	pOP-EO05760_EST_C_1_pSK_SK	646
cl2143	ct2340	cn2557	pOP-CEO03646_EST_C_1_pSK_SK	245
cl2143	ct2340	cn2557	pOP-EO05761_EST_C_1_pSK_SK	588
cl2144	ct2341	cn2558	pOP-EO05102_EST_C_1_pSK_SK	429
cl2144	ct2341	cn2558	pOP-EO05765_EST_C_1_pSK_SK	580

cl2145	ct2342	cn2559	pOP-CEO02416_EST_C_1_pSK_SK	196
cl2145	ct2342	cn2559	pOP-EAP01858_EST_C_1_pBSK_SK	724
cl2145	ct2342	cn2559	pOP-EO05767_EST_C_1_pSK_SK	694
cl2146	ct2343	cn2560	pOP-CNH04249	567
cl2146	ct2343	cn2560	pOP-EO05772_EST_C_1_pSK_SK	670
cl2147	ct2344	cn2561	pOP-EO05773_EST_C_1_pSK_SK	644
cl2147	ct2345	cn2562	pOP-CNH01746_EST_C_1_pSK_SK	556
cl2147	ct2345	cn2562	pOP-CNH03666_EST_C_1_pSK_SK	574
cl2148	ct2346	cn2563	pOP-EO05700_EST_C_1_pSK_SK	502
cl2148	ct2346	cn2563	pOP-EO05774_EST_C_1_pSK_SK	644
cl2149	ct2347	cn2564	pOP-CBP00264_EST_C_1_pBSK_SK	515
cl2149	ct2347	cn2564	pOP-CEM00052_EST_C_1_pSK_SK	225
cl2149	ct2347	cn2564	pOP-CEO02477_EST_C_1_pSK_SK	317
cl2149	ct2347	cn2564	pOP-CEO02883_EST_C_1_pSK_SK	214
cl2149	ct2347	cn2564	pOP-CNIP00830_EST_C_1_pSK_SK	269
cl2149	ct2347	cn2564	pOP-EAP00407_EST_C_1_pBSK_SK	288
cl2149	ct2347	cn2564	pOP-EAP00964_EST_C_1_pBSK_SK	251
cl2149	ct2347	cn2564	pOP-EAP02099_EST_C_1_pBSK_SK	368
cl2149	ct2347	cn2564	pOP-EAP03567_EST_C_1_pBSK_SK	187
cl2149	ct2347	cn2564	pOP-EO02392_EST_C_1_pSK_SK	646
cl2149	ct2347	cn2564	pOP-EO03576_EST_C_1_pSK_SK	535
cl2149	ct2347	cn2564	pOP-EO05779_EST_C_1_pSK_SK	598
cl2150	ct2348	cn2565	pOP-CNH00848_EST_C_1_pSK_SK	622
cl2150	ct2348	cn2565	pOP-CNH03621_EST_C_1_pSK_SK	608
cl2150	ct2348	cn2565	pOP-EAP02358_EST_C_1_pBSK_SK	602
cl2150	ct2348	cn2565	pOP-EO02738_EST_C_1_pSK_SK	450
cl2150	ct2348	cn2565	pOP-EO03424_EST_C_1_pSK_SK	437
cl2150	ct2348	cn2565	pOP-EO05780_EST_C_1_pSK_SK	718
cl2151	ct2349	cn2566	pOP-EO04851_EST_C_1_pSK_SK	551
cl2151	ct2349	cn2566	pOP-EO05781_EST_C_1_pSK_SK	556
cl2152	ct2350	cn2567	pOP-EN00816_EST_C_1_pSK_SK	493
cl2152	ct2350	cn2567	pOP-EO04277_EST_C_1_pSK_SK	267
cl2152	ct2350	cn2567	pOP-EO05785_EST_C_1_pSK_SK	644
cl2153	ct2351	cn2568	pOP-EO05789_EST_C_1_pSK_SK	462
cl2153	ct2351	cn2568	pOP-EO06645_EST_C_1_pSK_SK	463
cl2153	ct2351	cn2568	pOP-EO06778_EST_C_1_pSK_SK	456
cl2154	ct2352	cn2569	pOP-CNH02217_EST_C_1_pSK_SK	698
cl2154	ct2352	cn2569	pOP-CNH02485_EST_C_1_pSK_SK	395
cl2154	ct2352	cn2569	pOP-CNHP00482_EST_C_1_pSK_SK	615
cl2154	ct2352	cn2569	pOP-EO05793_EST_C_1_pSK_SK	642
cl2155	ct2353	cn2570	pOP-EO02798_EST_C_1_pSK_SK	423
cl2155	ct2353	cn2570	pOP-EO05109_EST_C_1_pSK_SK	530
cl2155	ct2353	cn2570	pOP-EO05794_EST_C_1_pSK_SK	599
cl2156	ct2354	cn2571	pOP-CEO02969_EST_C_1_pSK_SK	413
cl2156	ct2354	cn2571	pOP-EAP01291_EST_C_1_pBSK_SK	367
cl2156	ct2354	cn2571	pOP-EAP03419_EST_C_1_pBSK_SK	608
cl2156	ct2354	cn2571	pOP-EO05797_EST_C_1_pSK_SK	688
cl2156	ct2354	cn2572	pOP-CEO03160_EST_C_1_pSK_SK	530
cl2157	ct2355	cn2573	pOP-EO05799_EST_C_1_pSK_SK	512
cl2157	ct2355	cn2574	pOP-CEO01879_EST_C_1_pSK_SK	356
cl2158	ct2356	cn2575	pOP-EO04333_EST_C_1_pSK_SK	486
cl2158	ct2356	cn2575	pOP-EO05805_EST_C_1_pSK_SK	637
cl2158	ct2356	cn2575	pOP-EO05808_EST_C_1_pSK_SK	631
cl2159	ct2357	cn2576	pOP-CNH03359_EST_C_1_pSK_SK	567
cl2159	ct2357	cn2576	pOP-EO05813_EST_C_1_pSK_SK	501
cl2160	ct2358	cn2577	pOP-CNI01080_EST_C_1_pSK_SK	699

cl2160	ct2358	cn2578	pOP-CNH01843_EST_C_1_pSK_SK	455
cl2160	ct2358	cn2578	pOP-CNIP04056_EST_C_1_pSK_SK	434
cl2160	ct2358	cn2578	pOP-EAP02810_EST_C_1_pBSK_SK	652
cl2160	ct2358	cn2578	pOP-EO03771_EST_C_1_pSK_SK	493
cl2160	ct2359	cn2579	pOP-CAP00235_EST_C_1_pBSK_SK	566
cl2160	ct2359	cn2579	pOP-EO02177_EST_C_1_pSK_SK	301
cl2160	ct2359	cn2579	pOP-EO03782_EST_C_1_pSK_SK	347
cl2160	ct2359	cn2579	pOP-EO03966_EST_C_1_pSK_SK	496
cl2160	ct2359	cn2579	pOP-EO05818_EST_C_1_pSK_SK	651
cl2161	ct2360	cn2580	pOP-EO03549_EST_C_1_pSK_SK	517
cl2161	ct2360	cn2580	pOP-EO05819_EST_C_1_pSK_SK	691
cl2162	ct2361	cn2581	pOP-CEO00552_EST_C_1_pSK_SK	275
cl2162	ct2361	cn2581	pOP-CEO02290_EST_C_1_pSK_SK	296
cl2162	ct2361	cn2581	pOP-EO02740_EST_C_1_pSK_SK	416
cl2162	ct2361	cn2581	pOP-EO03656_EST_C_1_pSK_SK	358
cl2162	ct2361	cn2581	pOP-EO05212_EST_C_1_pSK_SK	536
cl2162	ct2361	cn2581	pOP-EO05841_EST_C_1_pSK_SK	526
cl2162	ct2361	cn2581	pOP-EO06653_EST_C_1_pSK_SK	658
cl2162	ct2361	cn2581	pOP-EO06770_EST_C_1_pSK_SK	738
cl2162	ct2361	cn2581	pOP-EO07140_EST_C_1_pSK_SK	656
cl2163	ct2362	cn2582	pOP-EO05858_EST_C_1_pSK_SK	606
cl2163	ct2362	cn2583	pOP-CNIP00439_EST_C_1_pSK_SK	331
cl2164	ct2363	cn2584	pOP-EO02790_EST_C_1_pSK_SK	414
cl2164	ct2363	cn2584	pOP-EO05860_EST_C_1_pSK_SK	476
cl2165	ct2364	cn2585	pOP-CNHP00239_EST_C_1_pSK_SK	458
cl2165	ct2364	cn2585	pOP-EO05861_EST_C_1_pSK_SK	355
cl2166	ct2365	cn2586	pOP-EO04495_EST_C_1_pSK_SK	528
cl2166	ct2365	cn2586	pOP-EO05863_EST_C_1_pSK_SK	562
cl2167	ct2366	cn2587	pOP-EO02517_EST_C_1_pSK_SK	528
cl2167	ct2367	cn2588	pOP-CEM00190_EST_C_1_pSK_SK	157
cl2167	ct2367	cn2588	pOP-EAP01066_EST_C_1_pBSK_SK	107
cl2167	ct2367	cn2588	pOP-EAP01851_EST_C_1_pBSK_SK	595
cl2167	ct2367	cn2588	pOP-EAP03573_EST_C_1_pBSK_SK	612
cl2167	ct2367	cn2588	pOP-EO02380_EST_C_1_pSK_SK	619
cl2167	ct2367	cn2588	pOP-EO05294_EST_C_1_pSK_SK	588
cl2167	ct2367	cn2588	pOP-EO05874_EST_C_1_pSK_SK	457
cl2167	ct2367	cn2588	pOP-EO07161_EST_C_1_pSK_SK	624
cl2167	ct2367	cn2588	pOP-EO07564_EST_C_1_pSK_SK	779
cl2168	ct2368	cn2589	pOP-CEO00875_EST_C_1_pSK_SK	538
cl2168	ct2368	cn2589	pOP-CNIP00856_EST_C_1_pSK_SK	269
cl2168	ct2368	cn2589	pOP-EAP00134_EST_C_1_pBSK_SK	278
cl2168	ct2368	cn2589	pOP-EBP03084_EST_C_1_pBSK_M13F	230
cl2168	ct2368	cn2589	pOP-EO05877_EST_C_1_pSK_SK	364
cl2169	ct2369	cn2590	pOP-EO02447_EST_C_1_pSK_SK	464
cl2169	ct2369	cn2590	pOP-EO04094_EST_C_1_pSK_SK	516
cl2169	ct2369	cn2590	pOP-EO04192_EST_C_1_pSK_SK	509
cl2169	ct2369	cn2590	pOP-EO05878_EST_C_1_pSK_SK	526
cl2170	ct2370	cn2591	pOP-CAP00096_EST_C_1_pBSK_SK	543
cl2170	ct2370	cn2591	pOP-CBP00262_EST_C_1_pBSK_SK	572
cl2170	ct2370	cn2591	pOP-EO02684_EST_C_1_pSK_SK	447
cl2170	ct2370	cn2591	pOP-EO05882_EST_C_1_pSK_SK	492
cl2171	ct2371	cn2592	pOP-CEO03615_EST_C_1_pSK_SK	111
cl2171	ct2371	cn2592	pOP-CNH04560	737
cl2171	ct2371	cn2592	pOP-EO05891_EST_C_1_pSK_SK	636
cl2172	ct2372	cn2593	pOP-CEO02123_EST_C_1_pSK_SK	223
cl2172	ct2372	cn2593	pOP-CNI01924_EST_C_1_pSK_SK	567

cl2172	ct2372	cn2593	pOP-EO05900_EST_C_1_pSK_SK	271
cl2173	ct2373	cn2594	pOP-CNH01505_EST_C_1_pSK_SK	354
cl2173	ct2373	cn2594	pOP-EO05901_EST_C_1_pSK_SK	525
cl2173	ct2373	cn2595	pOP-EO04391_EST_C_1_pSK_SK	407
cl2174	ct2374	cn2596	pOP-CEO01192_EST_C_1_pSK_SK	376
cl2174	ct2374	cn2596	pOP-EO02446_EST_C_1_pSK_SK	460
cl2174	ct2374	cn2596	pOP-EO03669_EST_C_1_pSK_SK	338
cl2174	ct2374	cn2596	pOP-EO04304_EST_C_1_pSK_SK	473
cl2174	ct2375	cn2597	pOP-CCP00018_EST_C_1_pBSK_SK	273
cl2174	ct2375	cn2597	pOP-CEO01098_EST_C_1_pSK_SK	158
cl2174	ct2375	cn2597	pOP-CEO01662_EST_C_1_pSK_SK	250
cl2174	ct2375	cn2597	pOP-CEO03403_EST_C_1_pSK_SK	456
cl2174	ct2375	cn2597	pOP-EO05918_EST_C_1_pSK_SK	577
cl2175	ct2376	cn2598	pOP-CNLP00007_EST_C_1_pSK_SK	623
cl2175	ct2376	cn2598	pOP-EO05919_EST_C_1_pSK_SK	687
cl2176	ct2377	cn2599	pOP-EO04617_EST_C_1_pSK_SK	527
cl2176	ct2377	cn2599	pOP-EO05920_EST_C_1_pSK_SK	626
cl2177	ct2378	cn2600	pOP-EN00657_EST_C_1_pSK_SK	573
cl2177	ct2378	cn2600	pOP-EO05923_EST_C_1_pSK_SK	517
cl2177	ct2378	cn2601	pOP-EN00882_EST_C_1_pSK_SK	729
cl2178	ct2379	cn2602	pOP-EAP03154_EST_C_1_pBSK_SK	453
cl2178	ct2379	cn2602	pOP-EO05953_EST_C_1_pSK_SK	443
cl2179	ct2380	cn2603	pOP-CNI01854_EST_C_1_pSK_SK	457
cl2179	ct2380	cn2603	pOP-EAP02708_EST_C_1_pBSK_SK	546
cl2179	ct2380	cn2603	pOP-EO05089_EST_C_1_pSK_SK	519
cl2179	ct2380	cn2603	pOP-EO05963_EST_C_1_pSK_SK	509
cl2180	ct2381	cn2604	pOP-CEO02910_EST_C_1_pSK_SK	168
cl2180	ct2381	cn2604	pOP-CNI02037_EST_C_1_pSK_SK	388
cl2180	ct2381	cn2604	pOP-EAP02843_EST_C_1_pBSK_SK	570
cl2180	ct2381	cn2604	pOP-EO05978_EST_C_1_pSK_SK	571
cl2180	ct2382	cn2605	pOP-CEO03755_EST_C_1_pSK_SK	483
cl2180	ct2382	cn2606	pOP-CEO01430_EST_C_1_pSK_SK	428
cl2180	ct2382	cn2606	pOP-CEO02947_EST_C_1_pSK_SK	180
cl2180	ct2382	cn2606	pOP-CNI01344_EST_C_1_pSK_SK	267
cl2180	ct2382	cn2606	pOP-EO04678_EST_C_1_pSK_SK	242
cl2181	ct2383	cn2607	pOP-EAP02952_EST_C_1_pBSK_SK	529
cl2181	ct2383	cn2607	pOP-EO03263_EST_C_1_pSK_SK	433
cl2181	ct2383	cn2607	pOP-EO05980_EST_C_1_pSK_SK	586
cl2182	ct2384	cn2608	pOP-CEO02419_EST_C_1_pSK_SK	648
cl2182	ct2384	cn2608	pOP-CEO02862_EST_C_1_pSK_SK	499
cl2182	ct2384	cn2608	pOP-EAP01362_EST_C_1_pBSK_SK	284
cl2182	ct2384	cn2608	pOP-EAP03455_EST_C_1_pBSK_SK	625
cl2182	ct2384	cn2608	pOP-EO05981_EST_C_1_pSK_SK	522
cl2183	ct2385	cn2609	pOP-EO05995_EST_C_1_pSK_SK	570
cl2183	ct2385	cn2610	pOP-CEO01948_EST_C_1_pSK_SK	441
cl2183	ct2386	cn2611	pOP-CEO00644_EST_C_1_pSK_SK	162
cl2183	ct2386	cn2611	pOP-CEO02775_EST_C_1_pSK_SK	301
cl2183	ct2386	cn2611	pOP-CEO03250_EST_C_1_pSK_SK	271
cl2183	ct2386	cn2611	pOP-CNI02034_EST_C_1_pSK_SK	525
cl2183	ct2386	cn2611	pOP-EAP01524_EST_C_1_pBSK_SK	533
cl2183	ct2386	cn2611	pOP-EAP03320_EST_C_1_pBSK_SK	535
cl2184	ct2387	cn2612	pOP-CNIP00122_EST_C_1_pSK_SK	333
cl2184	ct2387	cn2612	pOP-EAP01742_EST_C_1_pBSK_SK	282
cl2184	ct2387	cn2612	pOP-EO05996_EST_C_1_pSK_SK	363
cl2185	ct2388	cn2613	pOP-CEO01219_EST_C_1_pSK_SK	678
cl2185	ct2388	cn2613	pOP-EO06008_EST_C_1_pSK_SK	608

cl2186	ct2389	cn2614	pOP-CNH01225_EST_C_1_pSK_SK	582
cl2186	ct2389	cn2614	pOP-CNHP00120_EST_C_1_pSK_SK	583
cl2186	ct2389	cn2614	pOP-EO06016_EST_C_1_pSK_SK	578
cl2187	ct2390	cn2615	pOP-CEO01131_EST_C_1_pSK_SK	184
cl2187	ct2390	cn2615	pOP-CNIP00525_EST_C_1_pSK_SK	423
cl2187	ct2390	cn2615	pOP-CNIP01019_EST_C_1_pSK_SK	379
cl2187	ct2390	cn2615	pOP-EAP00313_EST_C_1_pBSK_SK	251
cl2187	ct2390	cn2615	pOP-EAP01987_EST_C_1_pBSK_SK	386
cl2187	ct2390	cn2615	pOP-EAP03636_EST_C_1_pBSK_SK	510
cl2187	ct2390	cn2615	pOP-EO02642_EST_C_1_pSK_SK	441
cl2187	ct2390	cn2615	pOP-EO03657_EST_C_1_pSK_SK	467
cl2187	ct2390	cn2615	pOP-EO03979_EST_C_1_pSK_SK	522
cl2187	ct2390	cn2615	pOP-EO04114_EST_C_1_pSK_SK	543
cl2187	ct2390	cn2615	pOP-EO06039_EST_C_1_pSK_SK	133
cl2187	ct2390	cn2615	pOP-EO06839_EST_C_1_pSK_SK	422
cl2187	ct2390	cn2615	pOP-EO07893_EST_C_1_pSK_SK	553
cl2188	ct2391	cn2616	pOP-CAP00399_EST_C_1_pBSK_SK	645
cl2188	ct2391	cn2616	pOP-EO06041_EST_C_1_pSK_SK	610
cl2189	ct2392	cn2617	pOP-EN00411_EST_C_1_pSK_SK	530
cl2189	ct2392	cn2617	pOP-EO02027_EST_C_1_pSK_SK	503
cl2189	ct2392	cn2617	pOP-EO03887_EST_C_1_pSK_SK	503
cl2189	ct2392	cn2617	pOP-EO03995_EST_C_1_pSK_SK	522
cl2189	ct2392	cn2617	pOP-EO04149_EST_C_1_pSK_SK	396
cl2189	ct2392	cn2617	pOP-EO04152_EST_C_1_pSK_SK	458
cl2189	ct2392	cn2617	pOP-EO04214_EST_C_1_pSK_SK	542
cl2189	ct2392	cn2617	pOP-EO05122_EST_C_1_pSK_SK	534
cl2189	ct2392	cn2617	pOP-EO06048_EST_C_1_pSK_SK	513
cl2189	ct2392	cn2617	pOP-EO07426_EST_C_1_pSK_SK	725
cl2190	ct2393	cn2618	pOP-EO05566_EST_C_1_pSK_SK	496
cl2190	ct2393	cn2618	pOP-EO06049_EST_C_1_pSK_SK	627
cl2191	ct2394	cn2619	pOP-CNH00776_EST_C_1_pSK_SK	422
cl2191	ct2394	cn2619	pOP-EO05387_EST_C_1_pSK_SK	552
cl2191	ct2394	cn2619	pOP-EO06053_EST_C_1_pSK_SK	335
cl2191	ct2394	cn2620	pOP-EN00489_EST_C_1_pSK_SK	485
cl2192	ct2395	cn2621	pOP-EAP03221_EST_C_1_pBSK_SK	243
cl2192	ct2395	cn2621	pOP-EO06060_EST_C_1_pSK_SK	655
cl2192	ct2395	cn2622	pOP-CBP00139_EST_C_1_pBSK_SK	601
cl2193	ct2396	cn2623	pOP-EAP03745_EST_C_1_pBSK_SK	526
cl2193	ct2396	cn2623	pOP-EO04997_EST_C_1_pSK_SK	489
cl2193	ct2396	cn2623	pOP-EO06067_EST_C_1_pSK_SK	630
cl2194	ct2397	cn2624	pOP-EO02396_EST_C_1_pSK_SK	428
cl2194	ct2397	cn2624	pOP-EO06074_EST_C_1_pSK_SK	481
cl2195	ct2398	cn2625	pOP-CAP00234_EST_C_1_pBSK_SK	580
cl2195	ct2398	cn2625	pOP-CNH01156_EST_C_1_pSK_SK	379
cl2195	ct2398	cn2625	pOP-CNI01184_EST_C_1_pSK_SK	432
cl2195	ct2398	cn2625	pOP-CNIP04090_EST_C_1_pSK_SK	477
cl2195	ct2398	cn2625	pOP-EO02112_EST_C_1_pSK_SK	581
cl2195	ct2398	cn2625	pOP-EO06078_EST_C_1_pSK_SK	644
cl2196	ct2399	cn2626	pOP-EO03762_EST_C_1_pSK_SK	400
cl2196	ct2399	cn2626	pOP-EO06080_EST_C_1_pSK_SK	636
cl2197	ct2400	cn2627	pOP-CAP00349_EST_C_1_pBSK_SK	568
cl2197	ct2400	cn2627	pOP-CNH02774_EST_C_1_pSK_SK	700
cl2197	ct2400	cn2627	pOP-EO06090_EST_C_1_pSK_SK	650
cl2198	ct2401	cn2628	pOP-EO03124_EST_C_1_pSK_SK	456
cl2198	ct2401	cn2628	pOP-EO04500_EST_C_1_pSK_SK	447
cl2198	ct2401	cn2628	pOP-EO04798_EST_C_1_pSK_SK	571

cl2198	ct2401	cn2628	pOP-EO05786_EST_C_1_pSK_SK	421
cl2198	ct2401	cn2628	pOP-EO06093_EST_C_1_pSK_SK	646
cl2198	ct2401	cn2629	pOP-EN00608_EST_C_1_pSK_SK	570
cl2199	ct2402	cn2630	pOP-EO04576_EST_C_1_pSK_SK	548
cl2199	ct2402	cn2630	pOP-EO04649_EST_C_1_pSK_SK	534
cl2199	ct2402	cn2630	pOP-EO04881_EST_C_1_pSK_SK	504
cl2199	ct2402	cn2630	pOP-EO05080_EST_C_1_pSK_SK	525
cl2199	ct2402	cn2630	pOP-EO06094_EST_C_1_pSK_SK	516
cl2200	ct2403	cn2631	pOP-EAP00319_EST_C_1_pBSK_SK	623
cl2200	ct2403	cn2631	pOP-EAP00320_EST_C_1_pBSK_SK	625
cl2200	ct2403	cn2631	pOP-EAP05005_EST_C_1_pBSK_SK	566
cl2200	ct2403	cn2631	pOP-EO02788_EST_C_1_pSK_SK	328
cl2200	ct2403	cn2631	pOP-EO04389_EST_C_1_pSK_SK	511
cl2200	ct2403	cn2631	pOP-EO04561_EST_C_1_pSK_SK	428
cl2200	ct2403	cn2631	pOP-EO06084_EST_C_1_pSK_SK	642
cl2200	ct2403	cn2631	pOP-EO06096_EST_C_1_pSK_SK	262
cl2200	ct2403	cn2631	pOP-EO06532_EST_C_1_pSK_SK	780
cl2200	ct2403	cn2631	pOP-EO07897_EST_C_1_pSK_SK	674
cl2201	ct2404	cn2632	pOP-CNH02969_EST_C_1_pSK_SK	440
cl2201	ct2404	cn2632	pOP-EO03209_EST_C_1_pSK_SK	468
cl2201	ct2404	cn2632	pOP-EO05870_EST_C_1_pSK_SK	486
cl2201	ct2405	cn2633	pOP-CEO03647_EST_C_1_pSK_SK	358
cl2201	ct2405	cn2633	pOP-CNI01626_EST_C_1_pSK_SK	542
cl2201	ct2405	cn2633	pOP-EAP00769_EST_C_1_pBSK_SK	285
cl2201	ct2405	cn2633	pOP-EO04307_EST_C_1_pSK_SK	460
cl2201	ct2405	cn2633	pOP-EO06115_EST_C_1_pSK_SK	721
cl2201	ct2405	cn2633	pOP-EO06390_EST_C_1_pSK_SK	754
cl2201	ct2405	cn2633	pOP-EO07251_EST_C_1_pSK_SK	770
cl2202	ct2406	cn2634	pOP-EO06116_EST_C_1_pSK_SK	697
cl2202	ct2406	cn2635	pOP-EO04886_EST_C_1_pSK_SK	510
cl2203	ct2407	cn2636	pOP-CEO01590_EST_C_1_pSK_SK	366
cl2203	ct2407	cn2636	pOP-CEO03013_EST_C_1_pSK_SK	162
cl2203	ct2407	cn2636	pOP-CEO03014_EST_C_1_pSK_SK	397
cl2203	ct2407	cn2636	pOP-CEO03589_EST_C_1_pSK_SK	325
cl2203	ct2407	cn2636	pOP-EAP00694_EST_C_1_pBSK_SK	306
cl2203	ct2407	cn2636	pOP-EAP01855_EST_C_1_pBSK_SK	677
cl2203	ct2407	cn2636	pOP-EO06134_EST_C_1_pSK_SK	333
cl2204	ct2408	cn2637	pOP-CNH01335_EST_C_1_pSK_SK	685
cl2204	ct2408	cn2637	pOP-CNH01589_EST_C_1_pSK_SK	425
cl2204	ct2408	cn2637	pOP-CNH01905_EST_C_1_pSK_SK	634
cl2204	ct2408	cn2637	pOP-CNI02161_EST_C_1_pSK_SK	373
cl2204	ct2408	cn2637	pOP-CNIP00020_EST_C_1_pSK_SK	656
cl2204	ct2408	cn2637	pOP-EO06141_EST_C_1_pSK_SK	340
cl2204	ct2408	cn2637	pOP-EO08077_EST_C_1_pSK_SK	476
cl2205	ct2409	cn2638	pOP-CNI01965_EST_C_1_pSK_SK	513
cl2205	ct2409	cn2638	pOP-CNI02169_EST_C_1_pSK_SK	441
cl2205	ct2409	cn2638	pOP-EN00236_EST_C_1_pSK_SK	437
cl2205	ct2410	cn2639	pOP-CEM00126_EST_C_1_pSK_SK	450
cl2205	ct2410	cn2639	pOP-CEO00657_EST_C_1_pSK_SK	360
cl2205	ct2410	cn2639	pOP-CEO01264	179
cl2205	ct2410	cn2639	pOP-CEO02550_EST_C_1_pSK_SK	547
cl2205	ct2410	cn2639	pOP-EO05234_EST_C_1_pSK_SK	535
cl2205	ct2410	cn2639	pOP-EO05433_EST_C_1_pSK_SK	486
cl2205	ct2410	cn2639	pOP-EO06145_EST_C_1_pSK_SK	493
cl2206	ct2411	cn2640	pOP-CNH00959_EST_C_1_pSK_SK	617
cl2206	ct2411	cn2640	pOP-EO05715_EST_C_1_pSK_SK	383

cl2206	ct2411	cn2640	pOP-EO06147_EST_C_1_pSK_SK	538
cl2207	ct2412	cn2641	pOP-CEO01771_EST_C_1_pSK_SK	364
cl2207	ct2412	cn2641	pOP-CNIP00715_EST_C_1_pSK_SK	583
cl2207	ct2412	cn2641	pOP-EAP03247_EST_C_1_pBSK_SK	682
cl2207	ct2412	cn2641	pOP-EO06168_EST_C_1_pSK_SK	588
cl2208	ct2413	cn2642	pOP-CNH01310_EST_C_1_pSK_SK	695
cl2208	ct2413	cn2642	pOP-CNH04934_EST_C_1_pSK_SK	709
cl2208	ct2413	cn2642	pOP-EO06169_EST_C_1_pSK_SK	442
cl2209	ct2414	cn2643	pOP-CNI02027_EST_C_1_pSK_SK	231
cl2209	ct2414	cn2643	pOP-EO02758_EST_C_1_pSK_SK	448
cl2209	ct2414	cn2643	pOP-EO03235_EST_C_1_pSK_SK	461
cl2209	ct2414	cn2643	pOP-EO04532_EST_C_1_pSK_SK	507
cl2209	ct2414	cn2643	pOP-EO06170_EST_C_1_pSK_SK	560
cl2209	ct2414	cn2643	pOP-EO06181_EST_C_1_pSK_SK	640
cl2210	ct2415	cn2644	pOP-CEO02151_EST_C_1_pSK_SK	461
cl2210	ct2415	cn2644	pOP-EO04648_EST_C_1_pSK_SK	474
cl2210	ct2415	cn2644	pOP-EO06486_EST_C_1_pSK_SK	533
cl2210	ct2415	cn2644	pOP-EO08201_EST_C_1_pSK_SK	573
cl2210	ct2415	cn2644	pOP-EO08452_EST_C_1_pSK_SK	212
cl2210	ct2415	cn2644	pOP-EOP00008_EST_C_1_pSK_SK	619
cl2210	ct2416	cn2645	pOP-CEO03632_EST_C_1_pSK_SK	458
cl2210	ct2416	cn2645	pOP-CNI01082_EST_C_1_pSK_SK	613
cl2210	ct2416	cn2645	pOP-EAP00878_EST_C_1_pBSK_SK	353
cl2210	ct2416	cn2645	pOP-EAP02871_EST_C_1_pBSK_SK	459
cl2210	ct2416	cn2645	pOP-EAP03415_EST_C_1_pBSK_SK	379
cl2210	ct2416	cn2645	pOP-EAP03668_EST_C_1_pBSK_SK	285
cl2210	ct2416	cn2646	pOP-CNI02084_EST_C_1_pSK_SK	605
cl2210	ct2416	cn2647	pOP-CEM00210_EST_C_1_pSK_SK	456
cl2210	ct2416	cn2647	pOP-CEM00232_EST_C_1_pSK_SK	138
cl2210	ct2416	cn2647	pOP-CEO02146_EST_C_1_pSK_SK	421
cl2210	ct2416	cn2647	pOP-CEO02715_EST_C_1_pSK_SK	436
cl2210	ct2416	cn2647	pOP-CNH02368_EST_C_1_pSK_SK	380
cl2210	ct2416	cn2647	pOP-CNH04403	432
cl2210	ct2416	cn2647	pOP-CNI01479_EST_C_1_pSK_SK	329
cl2210	ct2416	cn2647	pOP-CNI02081_EST_C_1_pSK_SK	317
cl2210	ct2416	cn2647	pOP-CNIP00679_EST_C_1_pSK_SK	246
cl2210	ct2416	cn2647	pOP-EAP00317_EST_C_1_pBSK_SK	111
cl2210	ct2416	cn2647	pOP-EAP00748_EST_C_1_pBSK_SK	200
cl2210	ct2416	cn2647	pOP-EAP00749_EST_C_1_pBSK_SK	159
cl2210	ct2416	cn2647	pOP-EAP01617_EST_C_1_pBSK_SK	663
cl2210	ct2416	cn2647	pOP-EAP01866_EST_C_1_pBSK_SK	472
cl2210	ct2416	cn2647	pOP-EO02503_EST_C_1_pSK_SK	522
cl2210	ct2416	cn2647	pOP-EO06188_EST_C_1_pSK_SK	343
cl2210	ct2416	cn2647	pOP-EO07247_EST_C_1_pSK_SK	771
cl2210	ct2416	cn2647	pOP-EO07771_EST_C_1_pSK_SK	736
cl2210	ct2416	cn2648	pOP-CBP00148_EST_C_1_pBSK_SK	500
cl2210	ct2416	cn2648	pOP-CNH01628_EST_C_1_pSK_SK	327
cl2210	ct2416	cn2648	pOP-CNH01996_EST_C_1_pSK_SK	460
cl2210	ct2416	cn2648	pOP-CNHP00323_EST_C_1_pSK_SK	511
cl2210	ct2416	cn2648	pOP-CNHP00324_EST_C_1_pSK_SK	513
cl2210	ct2416	cn2648	pOP-CNHP00435_EST_C_1_pSK_SK	460
cl2210	ct2416	cn2648	pOP-CNI01352_EST_C_1_pSK_SK	471
cl2210	ct2416	cn2648	pOP-CNI01421_EST_C_1_pSK_SK	175
cl2210	ct2416	cn2648	pOP-CNI01430_EST_C_1_pSK_SK	416
cl2210	ct2416	cn2648	pOP-CNI01545_EST_C_1_pSK_SK	149
cl2210	ct2416	cn2648	pOP-CNI01852_EST_C_1_pSK_SK	310

cl2210	ct2416	cn2648	pOP-CNI02166_EST_C_1_pSK_SK	354
cl2210	ct2416	cn2648	pOP-CNI02174_EST_C_1_pSK_SK	201
cl2210	ct2416	cn2648	pOP-CNIP00061_EST_C_1_pSK_SK	167
cl2210	ct2416	cn2648	pOP-CNIP00072_EST_C_1_pSK_SK	406
cl2210	ct2416	cn2648	pOP-CNIP00360_EST_C_1_pSK_SK	454
cl2210	ct2416	cn2648	pOP-CNIP00480_EST_C_1_pSK_SK	413
cl2210	ct2416	cn2648	pOP-CNIP00564_EST_C_1_pSK_SK	355
cl2210	ct2416	cn2648	pOP-CNIP00710_EST_C_1_pSK_SK	246
cl2210	ct2416	cn2648	pOP-CNIP04057_EST_C_1_pSK_SK	134
cl2210	ct2416	cn2648	pOP-EAP00480_EST_C_1_pBSK_SK	144
cl2210	ct2416	cn2648	pOP-EAP01047_EST_C_1_pBSK_SK	520
cl2210	ct2416	cn2648	pOP-EAP01059_EST_C_1_pBSK_SK	111
cl2210	ct2416	cn2648	pOP-EAP01080_EST_C_1_pBSK_SK	320
cl2210	ct2416	cn2648	pOP-EAP01328_EST_C_1_pBSK_SK	257
cl2210	ct2416	cn2648	pOP-EAP01487_EST_C_1_pBSK_SK	519
cl2210	ct2416	cn2648	pOP-EAP01652_EST_C_1_pBSK_SK	516
cl2210	ct2416	cn2648	pOP-EAP01714_EST_C_1_pBSK_SK	628
cl2210	ct2416	cn2648	pOP-EAP02009_EST_C_1_pBSK_SK	362
cl2210	ct2416	cn2648	pOP-EAP02345_EST_C_1_pBSK_SK	349
cl2210	ct2416	cn2648	pOP-EAP02872_EST_C_1_pBSK_SK	267
cl2210	ct2416	cn2648	pOP-EAP02876_EST_C_1_pBSK_SK	510
cl2210	ct2416	cn2648	pOP-EAP03435_EST_C_1_pBSK_SK	333
cl2210	ct2416	cn2648	pOP-EAP03749_EST_C_1_pBSK_SK	315
cl2210	ct2416	cn2648	pOP-EN00145_EST_C_1_pSK_SK	478
cl2210	ct2416	cn2648	pOP-EO05889_EST_C_1_pSK_SK	590
cl2210	ct2416	cn2649	pOP-EO02021_EST_C_1_pSK_SK	323
cl2210	ct2416	cn2650	pOP-CNIP00697_EST_C_1_pSK_SK	193
cl2211	ct2417	cn2651	pOP-EN00581_EST_C_1_pSK_SK	541
cl2211	ct2417	cn2651	pOP-EO06191_EST_C_1_pSK_SK	676
cl2212	ct2418	cn2652	pOP-EO04010_EST_C_1_pSK_SK	498
cl2212	ct2418	cn2652	pOP-EO04658_EST_C_1_pSK_SK	507
cl2212	ct2418	cn2652	pOP-EO06192_EST_C_1_pSK_SK	671
cl2213	ct2419	cn2653	pOP-CBP00271_EST_C_1_pBSK_SK	581
cl2213	ct2419	cn2653	pOP-CEO01685_EST_C_1_pSK_SK	312
cl2213	ct2419	cn2653	pOP-CNIP00451_EST_C_1_pSK_SK	106
cl2213	ct2419	cn2653	pOP-EO06197_EST_C_1_pSK_SK	544
cl2214	ct2420	cn2654	pOP-EO02317_EST_C_1_pSK_SK	529
cl2214	ct2420	cn2654	pOP-EO06208_EST_C_1_pSK_SK	504
cl2215	ct2421	cn2655	pOP-CEM00218_EST_C_1_pSK_SK	496
cl2215	ct2421	cn2655	pOP-CNH02116_EST_C_1_pSK_SK	460
cl2215	ct2421	cn2655	pOP-EAP03132_EST_C_1_pBSK_SK	523
cl2215	ct2421	cn2655	pOP-EO07094_EST_C_1_pSK_SK	504
cl2215	ct2422	cn2656	pOP-CNH00917_EST_C_1_pSK_SK	570
cl2215	ct2422	cn2656	pOP-CNH02843_EST_C_1_pSK_SK	593
cl2215	ct2422	cn2656	pOP-CNH04162	565
cl2215	ct2422	cn2656	pOP-EN00834_EST_C_1_pSK_SK	522
cl2215	ct2422	cn2656	pOP-EN00904_EST_C_1_pSK_SK	459
cl2215	ct2422	cn2656	pOP-EO05032_EST_C_1_pSK_SK	424
cl2215	ct2422	cn2656	pOP-EO06209_EST_C_1_pSK_SK	426
cl2216	ct2423	cn2657	pOP-CNI01368_EST_C_1_pSK_SK	294
cl2216	ct2423	cn2657	pOP-EAP01378_EST_C_1_pBSK_SK	340
cl2216	ct2423	cn2657	pOP-EO06212_EST_C_1_pSK_SK	307
cl2216	ct2423	cn2657	pOP-EO07187_EST_C_1_pSK_SK	550
cl2216	ct2423	cn2657	pOP-EO08211_EST_C_1_pSK_SK	519
cl2216	ct2423	cn2658	pOP-CEO00927_EST_C_1_pSK_SK	333
cl2217	ct2424	cn2659	pOP-CEO02410_EST_C_1_pSK_SK	462

cl2217	ct2424	cn2659	pOP-CNH00745_EST_C_1_pSK_SK	572
cl2217	ct2424	cn2659	pOP-CNHP00271_EST_C_1_pSK_SK	551
cl2217	ct2424	cn2659	pOP-CNI01958_EST_C_1_pSK_SK	299
cl2217	ct2424	cn2659	pOP-CNI02233_EST_C_1_pSK_SK	343
cl2217	ct2424	cn2659	pOP-CNIP00131_EST_C_1_pSK_SK	344
cl2217	ct2424	cn2659	pOP-CNIP00461_EST_C_1_pSK_SK	357
cl2217	ct2424	cn2659	pOP-CNIP00668_EST_C_1_pSK_SK	511
cl2217	ct2424	cn2659	pOP-CNIP00783_EST_C_1_pSK_SK	390
cl2217	ct2424	cn2659	pOP-EO06217_EST_C_1_pSK_SK	616
cl2218	ct2425	cn2660	pOP-EO04493_EST_C_1_pSK_SK	403
cl2218	ct2425	cn2660	pOP-EO05100_EST_C_1_pSK_SK	492
cl2218	ct2425	cn2660	pOP-EO06221_EST_C_1_pSK_SK	504
cl2219	ct2426	cn2661	pOP-CNI01131_EST_C_1_pSK_SK	342
cl2219	ct2426	cn2661	pOP-CNIP00073_EST_C_1_pSK_SK	508
cl2219	ct2426	cn2661	pOP-EO06227_EST_C_1_pSK_SK	570
cl2220	ct2427	cn2662	pOP-EO06234_EST_C_1_pSK_SK	651
cl2220	ct2427	cn2662	pOP-EO07218_EST_C_1_pSK_SK	487
cl2221	ct2428	cn2663	pOP-EO03940_EST_C_1_pSK_SK	401
cl2221	ct2428	cn2663	pOP-EO06240_EST_C_1_pSK_SK	401
cl2221	ct2428	cn2664	pOP-CEO01105_EST_C_1_pSK_SK	350
cl2221	ct2428	cn2665	pOP-CEO01858_EST_C_1_pSK_SK	318
cl2222	ct2429	cn2666	pOP-CNH00688_EST_C_1_pSK_SK	456
cl2222	ct2429	cn2666	pOP-CNH01501_EST_C_1_pSK_SK	646
cl2222	ct2429	cn2666	pOP-CNH01823_EST_C_1_pSK_SK	454
cl2222	ct2429	cn2666	pOP-EAP02304_EST_C_1_pBSK_SK	676
cl2222	ct2429	cn2666	pOP-EO06246_EST_C_1_pSK_SK	580
cl2223	ct2430	cn2667	pOP-CBP00141_EST_C_1_pBSK_SK	646
cl2223	ct2430	cn2667	pOP-EO03190_EST_C_1_pSK_SK	417
cl2223	ct2430	cn2667	pOP-EO04026_EST_C_1_pSK_SK	524
cl2223	ct2430	cn2667	pOP-EO04568_EST_C_1_pSK_SK	543
cl2223	ct2431	cn2668	pOP-CEO01281	426
cl2223	ct2431	cn2668	pOP-CNIP00475_EST_C_1_pSK_SK	463
cl2223	ct2431	cn2668	pOP-CNIP00684_EST_C_1_pSK_SK	291
cl2223	ct2431	cn2668	pOP-EO03027_EST_C_1_pSK_SK	465
cl2223	ct2431	cn2668	pOP-EO04266_EST_C_1_pSK_SK	503
cl2223	ct2432	cn2669	pOP-CEO01369_EST_C_1_pSK_SK	531
cl2223	ct2432	cn2669	pOP-CNI01373_EST_C_1_pSK_SK	215
cl2223	ct2432	cn2669	pOP-EAP03121_EST_C_1_pBSK_SK	546
cl2223	ct2432	cn2669	pOP-EAP05054_EST_C_1_pBSK_SK	577
cl2223	ct2432	cn2669	pOP-EO02249_EST_C_1_pSK_SK	627
cl2223	ct2432	cn2669	pOP-EO04486_EST_C_1_pSK_SK	330
cl2223	ct2432	cn2669	pOP-EO04904_EST_C_1_pSK_SK	499
cl2223	ct2432	cn2669	pOP-EO05054_EST_C_1_pSK_SK	515
cl2223	ct2432	cn2669	pOP-EO06251_EST_C_1_pSK_SK	689
cl2224	ct2433	cn2670	pOP-CNH02505_EST_C_1_pSK_SK	525
cl2224	ct2433	cn2670	pOP-EAP01909_EST_C_1_pBSK_SK	650
cl2224	ct2433	cn2670	pOP-EO03280_EST_C_1_pSK_SK	450
cl2224	ct2433	cn2670	pOP-EO06261_EST_C_1_pSK_SK	656
cl2225	ct2434	cn2671	pOP-EO03313_EST_C_1_pSK_SK	499
cl2225	ct2434	cn2671	pOP-EO06277_EST_C_1_pSK_SK	642
cl2226	ct2435	cn2672	pOP-EAP00073_EST_C_1_pBSK_SK	599
cl2226	ct2435	cn2672	pOP-EO06132_EST_C_1_pSK_SK	669
cl2226	ct2435	cn2672	pOP-EO06283_EST_C_1_pSK_SK	648
cl2226	ct2435	cn2673	pOP-EAP02719_EST_C_1_pBSK_SK	569
cl2227	ct2436	cn2674	pOP-CAP00061_EST_C_1_pBSK_SK	585
cl2227	ct2436	cn2674	pOP-CAP00285_EST_C_1_pBSK_SK	594

cl2227	ct2436	cn2674	pOP-CAP00395_EST_C_1_pBSK_SK	580
cl2227	ct2436	cn2674	pOP-EO06284_EST_C_1_pSK_SK	323
cl2228	ct2437	cn2675	pOP-EO02158_EST_C_1_pSK_SK	579
cl2228	ct2437	cn2675	pOP-EO06296_EST_C_1_pSK_SK	683
cl2229	ct2438	cn2676	pOP-CNH02386_EST_C_1_pSK_SK	522
cl2229	ct2438	cn2676	pOP-EO06297_EST_C_1_pSK_SK	698
cl2230	ct2439	cn2677	pOP-CNI02041_EST_C_1_pSK_SK	546
cl2230	ct2440	cn2678	pOP-CNI02091_EST_C_1_pSK_SK	375
cl2230	ct2440	cn2678	pOP-EO06301_EST_C_1_pSK_SK	345
cl2230	ct2441	cn2679	pOP-CNIP00476_EST_C_1_pSK_SK	221
cl2230	ct2441	cn2679	pOP-EAP00535_EST_C_1_pBSK_SK	276
cl2230	ct2441	cn2679	pOP-EO05829_EST_C_1_pSK_SK	598
cl2231	ct2442	cn2680	pOP-EO04345_EST_C_1_pSK_SK	514
cl2231	ct2442	cn2680	pOP-EO04347_EST_C_1_pSK_SK	514
cl2231	ct2442	cn2680	pOP-EO06308_EST_C_1_pSK_SK	615
cl2232	ct2443	cn2681	pOP-EAP03255_EST_C_1_pBSK_SK	552
cl2232	ct2443	cn2681	pOP-EO05227_EST_C_1_pSK_SK	534
cl2232	ct2443	cn2681	pOP-EO06311_EST_C_1_pSK_SK	577
cl2233	ct2444	cn2682	pOP-CNH01313_EST_C_1_pSK_SK	568
cl2233	ct2444	cn2682	pOP-CNH02207_EST_C_1_pSK_SK	560
cl2233	ct2444	cn2682	pOP-EO06329_EST_C_1_pSK_SK	737
cl2234	ct2445	cn2683	pOP-EO04263_EST_C_1_pSK_SK	330
cl2234	ct2445	cn2683	pOP-EO06337_EST_C_1_pSK_SK	764
cl2235	ct2446	cn2684	pOP-CEOP00011_EST_C_1_pSK_SK	359
cl2235	ct2446	cn2684	pOP-EO02115_EST_C_1_pSK_SK	721
cl2235	ct2446	cn2684	pOP-EO02808_EST_C_1_pSK_SK	399
cl2235	ct2446	cn2684	pOP-EO02836_EST_C_1_pSK_SK	297
cl2235	ct2446	cn2684	pOP-EO03060_EST_C_1_pSK_SK	204
cl2235	ct2446	cn2684	pOP-EO03066_EST_C_1_pSK_SK	285
cl2235	ct2446	cn2684	pOP-EO03759_EST_C_1_pSK_SK	533
cl2235	ct2446	cn2684	pOP-EO03942_EST_C_1_pSK_SK	447
cl2235	ct2446	cn2684	pOP-EO04398_EST_C_1_pSK_SK	525
cl2235	ct2446	cn2684	pOP-EO04706_EST_C_1_pSK_SK	537
cl2235	ct2446	cn2684	pOP-EO05880_EST_C_1_pSK_SK	634
cl2235	ct2446	cn2684	pOP-EO06346_EST_C_1_pSK_SK	687
cl2235	ct2446	cn2684	pOP-EO06968_EST_C_1_pSK_SK	755
cl2235	ct2446	cn2684	pOP-EO07118_EST_C_1_pSK_SK	645
cl2235	ct2446	cn2684	pOP-EO07941_EST_C_1_pSK_SK	677
cl2236	ct2447	cn2685	pOP-EO03483_EST_C_1_pSK_SK	537
cl2236	ct2447	cn2685	pOP-EO06356_EST_C_1_pSK_SK	698
cl2237	ct2448	cn2686	pOP-CEO00514_EST_C_1_pSK_SK	335
cl2237	ct2448	cn2686	pOP-EAP01097_EST_C_1_pBSK_SK	350
cl2237	ct2448	cn2686	pOP-EAP01994_EST_C_1_pBSK_SK	397
cl2237	ct2448	cn2686	pOP-EO04025_EST_C_1_pSK_SK	489
cl2237	ct2448	cn2686	pOP-EO04224_EST_C_1_pSK_SK	469
cl2237	ct2448	cn2686	pOP-EO04494_EST_C_1_pSK_SK	522
cl2237	ct2448	cn2686	pOP-EO06358_EST_C_1_pSK_SK	645
cl2237	ct2448	cn2686	pOP-EO08274_EST_C_1_pSK_SK	236
cl2237	ct2448	cn2687	pOP-CEO01724_EST_C_1_pSK_SK	338
cl2237	ct2448	cn2687	pOP-CEO01905_EST_C_1_pSK_SK	292
cl2238	ct2449	cn2688	pOP-CBP00024_EST_C_1_pBSK_SK	372
cl2238	ct2449	cn2688	pOP-CBP00103_EST_C_1_pBSK_SK	509
cl2238	ct2449	cn2688	pOP-CBP00178_EST_C_1_pBSK_SK	374
cl2238	ct2449	cn2688	pOP-CNIP00280_EST_C_1_pSK_SK	162
cl2238	ct2449	cn2688	pOP-EAP02332_EST_C_1_pBSK_SK	338
cl2238	ct2449	cn2688	pOP-EO02473_EST_C_1_pSK_SK	472

cl2238	ct2449	cn2688	pOP-EO02935_EST_C_1_pSK_SK	453
cl2238	ct2449	cn2688	pOP-EO03080_EST_C_1_pSK_SK	459
cl2238	ct2449	cn2688	pOP-EO06361_EST_C_1_pSK_SK	436
cl2239	ct2450	cn2689	pOP-EO03360_EST_C_1_pSK_SK	284
cl2239	ct2450	cn2689	pOP-EO06366_EST_C_1_pSK_SK	709
cl2240	ct2451	cn2690	pOP-EO04311_EST_C_1_pSK_SK	356
cl2240	ct2451	cn2690	pOP-EO06368_EST_C_1_pSK_SK	798
cl2241	ct2452	cn2691	pOP-CEO01136_EST_C_1_pSK_SK	414
cl2241	ct2452	cn2691	pOP-EAP03216_EST_C_1_pBSK_SK	615
cl2241	ct2452	cn2691	pOP-EO06378_EST_C_1_pSK_SK	760
cl2242	ct2453	cn2692	pOP-EO05345_EST_C_1_pSK_SK	313
cl2242	ct2453	cn2692	pOP-EO06383_EST_C_1_pSK_SK	703
cl2243	ct2454	cn2693	pOP-CNH00570_EST_C_1_pSK_SK	636
cl2243	ct2454	cn2693	pOP-CNH02582_EST_C_1_pSK_SK	535
cl2243	ct2454	cn2693	pOP-CNH04463	738
cl2243	ct2454	cn2693	pOP-CNHP00309_EST_C_1_pSK_SK	648
cl2243	ct2454	cn2693	pOP-CNHP00311_EST_C_1_pSK_SK	683
cl2243	ct2454	cn2693	pOP-CNIP01053_EST_C_1_pSK_SK	281
cl2243	ct2454	cn2693	pOP-EO04655_EST_C_1_pSK_SK	494
cl2243	ct2454	cn2693	pOP-EO06307_EST_C_1_pSK_SK	643
cl2243	ct2454	cn2693	pOP-EO06394_EST_C_1_pSK_SK	484
cl2243	ct2454	cn2693	pOP-EO07464_EST_C_1_pSK_SK	808
cl2244	ct2455	cn2694	pOP-CNH02978_EST_C_1_pSK_SK	541
cl2244	ct2455	cn2694	pOP-CNH03112_EST_C_1_pSK_SK	609
cl2244	ct2455	cn2694	pOP-EO06396_EST_C_1_pSK_SK	746
cl2245	ct2456	cn2695	pOP-EO03679_EST_C_1_pSK_SK	393
cl2245	ct2456	cn2695	pOP-EO06403_EST_C_1_pSK_SK	568
cl2246	ct2457	cn2696	pOP-CBP00131_EST_C_1_pBSK_SK	679
cl2246	ct2457	cn2696	pOP-EO06405_EST_C_1_pSK_SK	586
cl2247	ct2458	cn2697	pOP-EO02800_EST_C_1_pSK_SK	374
cl2247	ct2458	cn2697	pOP-EO04359_EST_C_1_pSK_SK	520
cl2247	ct2458	cn2697	pOP-EO06406_EST_C_1_pSK_SK	704
cl2248	ct2459	cn2698	pOP-CEO01768_EST_C_1_pSK_SK	245
cl2248	ct2459	cn2698	pOP-EO06407_EST_C_1_pSK_SK	629
cl2249	ct2460	cn2699	pOP-CEO01453_EST_C_1_pSK_SK	327
cl2249	ct2460	cn2699	pOP-EO06409_EST_C_1_pSK_SK	514
cl2250	ct2461	cn2700	pOP-CEO01280	439
cl2250	ct2461	cn2700	pOP-EO06412_EST_C_1_pSK_SK	650
cl2251	ct2462	cn2701	pOP-CEO00602_EST_C_1_pSK_SK	571
cl2251	ct2462	cn2701	pOP-CEO00603_EST_C_1_pSK_SK	570
cl2251	ct2462	cn2701	pOP-CEO03159_EST_C_1_pSK_SK	200
cl2251	ct2462	cn2701	pOP-CEO03166_EST_C_1_pSK_SK	399
cl2251	ct2462	cn2701	pOP-CNI01099_EST_C_1_pSK_SK	426
cl2251	ct2462	cn2701	pOP-EO04944_EST_C_1_pSK_SK	524
cl2251	ct2462	cn2701	pOP-EO05449_EST_C_1_pSK_SK	499
cl2251	ct2462	cn2701	pOP-EO06421_EST_C_1_pSK_SK	573
cl2252	ct2463	cn2702	pOP-EN00131_EST_C_1_pSK_SK	647
cl2252	ct2463	cn2702	pOP-EN00132_EST_C_1_pSK_SK	641
cl2252	ct2463	cn2702	pOP-EN00899_EST_C_1_pSK_SK	531
cl2252	ct2463	cn2702	pOP-EO06425_EST_C_1_pSK_SK	737
cl2253	ct2464	cn2703	pOP-CNH02501_EST_C_1_pSK_SK	334
cl2253	ct2464	cn2703	pOP-EAP03369_EST_C_1_pBSK_SK	260
cl2253	ct2464	cn2703	pOP-EO06426_EST_C_1_pSK_SK	757
cl2254	ct2465	cn2704	pOP-CAP00286_EST_C_1_pBSK_SK	668
cl2254	ct2465	cn2704	pOP-EO06309_EST_C_1_pSK_SK	649
cl2254	ct2465	cn2704	pOP-EO06432_EST_C_1_pSK_SK	729

cl2254	ct2465	cn2705	pOP-EAP00570_EST_C_1_pBSK_SK	458
cl2255	ct2466	cn2706	pOP-CNI01170_EST_C_1_pSK_SK	431
cl2255	ct2466	cn2706	pOP-EO06437_EST_C_1_pSK_SK	636
cl2256	ct2467	cn2707	pOP-CNH01952_EST_C_1_pSK_SK	481
cl2256	ct2468	cn2708	pOP-EO06438_EST_C_1_pSK_SK	767
cl2257	ct2469	cn2709	pOP-EAP02813_EST_C_1_pBSK_SK	606
cl2257	ct2469	cn2709	pOP-EO06445_EST_C_1_pSK_SK	696
cl2258	ct2470	cn2710	pOP-EO04372_EST_C_1_pSK_SK	400
cl2258	ct2470	cn2710	pOP-EO06447_EST_C_1_pSK_SK	735
cl2259	ct2471	cn2711	pOP-EAP00773_EST_C_1_pBSK_SK	346
cl2259	ct2471	cn2711	pOP-EO04067_EST_C_1_pSK_SK	522
cl2259	ct2471	cn2711	pOP-EO06450_EST_C_1_pSK_SK	598
cl2260	ct2472	cn2712	pOP-CEO02069_EST_C_1_pSK_SK	286
cl2260	ct2472	cn2712	pOP-EO06457_EST_C_1_pSK_SK	691
cl2261	ct2473	cn2713	pOP-EO04437_EST_C_1_pSK_SK	528
cl2261	ct2473	cn2713	pOP-EO06462_EST_C_1_pSK_SK	786
cl2262	ct2474	cn2714	pOP-EO05970_EST_C_1_pSK_SK	623
cl2262	ct2474	cn2714	pOP-EO06468_EST_C_1_pSK_SK	769
cl2263	ct2475	cn2715	pOP-CEO03392_EST_C_1_pSK_SK	567
cl2263	ct2475	cn2715	pOP-EAP01122_EST_C_1_pBSK_SK	525
cl2263	ct2475	cn2715	pOP-EO02108_EST_C_1_pSK_SK	458
cl2263	ct2476	cn2716	pOP-EAP02859_EST_C_1_pBSK_SK	288
cl2263	ct2476	cn2716	pOP-EO02165_EST_C_1_pSK_SK	473
cl2263	ct2476	cn2716	pOP-EO02807_EST_C_1_pSK_SK	432
cl2263	ct2476	cn2717	pOP-EO06474_EST_C_1_pSK_SK	500
cl2264	ct2477	cn2718	pOP-EAP02014_EST_C_1_pBSK_SK	696
cl2264	ct2477	cn2718	pOP-EO06476_EST_C_1_pSK_SK	821
cl2265	ct2478	cn2719	pOP-CAP00371_EST_C_1_pBSK_SK	233
cl2265	ct2478	cn2719	pOP-CEO01665_EST_C_1_pSK_SK	554
cl2265	ct2478	cn2719	pOP-CEO02295_EST_C_1_pSK_SK	463
cl2265	ct2478	cn2719	pOP-CEO02674_EST_C_1_pSK_SK	379
cl2265	ct2478	cn2719	pOP-CNH00736_EST_C_1_pSK_SK	491
cl2265	ct2478	cn2719	pOP-CNH01814_EST_C_1_pSK_SK	478
cl2265	ct2478	cn2719	pOP-CNH04308	526
cl2265	ct2478	cn2719	pOP-CNIP00247_EST_C_1_pSK_SK	200
cl2265	ct2478	cn2719	pOP-CNIP04013_EST_C_1_pSK_SK	305
cl2265	ct2478	cn2719	pOP-CNIP04014_EST_C_1_pSK_SK	305
cl2265	ct2478	cn2719	pOP-EAP00240_EST_C_1_pBSK_SK	292
cl2265	ct2478	cn2719	pOP-EAP00294_EST_C_1_pBSK_SK	369
cl2265	ct2478	cn2719	pOP-EAP00408_EST_C_1_pBSK_SK	147
cl2265	ct2478	cn2719	pOP-EAP00896_EST_C_1_pBSK_SK	185
cl2265	ct2478	cn2719	pOP-EAP00963_EST_C_1_pBSK_SK	129
cl2265	ct2478	cn2719	pOP-EAP01129_EST_C_1_pBSK_SK	576
cl2265	ct2478	cn2719	pOP-EAP01340_EST_C_1_pBSK_SK	320
cl2265	ct2478	cn2719	pOP-EAP01505_EST_C_1_pBSK_SK	435
cl2265	ct2478	cn2719	pOP-EAP01902_EST_C_1_pBSK_SK	586
cl2265	ct2478	cn2719	pOP-EAP02147_EST_C_1_pBSK_SK	412
cl2265	ct2478	cn2719	pOP-EAP02945_EST_C_1_pBSK_SK	516
cl2265	ct2478	cn2719	pOP-EAP03572_EST_C_1_pBSK_SK	233
cl2265	ct2478	cn2719	pOP-EO02283_EST_C_1_pSK_SK	485
cl2265	ct2478	cn2719	pOP-EO03334_EST_C_1_pSK_SK	462
cl2265	ct2478	cn2719	pOP-EO03454_EST_C_1_pSK_SK	460
cl2265	ct2478	cn2719	pOP-EO03779_EST_C_1_pSK_SK	485
cl2265	ct2478	cn2719	pOP-EO04394_EST_C_1_pSK_SK	468
cl2265	ct2478	cn2719	pOP-EO04865_EST_C_1_pSK_SK	514
cl2265	ct2478	cn2719	pOP-EO06058_EST_C_1_pSK_SK	473

cl2265	ct2478	cn2719	pOP-EO06236_EST_C_1_pSK_SK	563
cl2265	ct2478	cn2719	pOP-EO06481_EST_C_1_pSK_SK	819
cl2265	ct2478	cn2719	pOP-EO06872_EST_C_1_pSK_SK	590
cl2266	ct2479	cn2720	pOP-EAP00923_EST_C_1_pBSK_SK	164
cl2266	ct2479	cn2720	pOP-EO06274_EST_C_1_pSK_SK	594
cl2266	ct2479	cn2720	pOP-EO06490_EST_C_1_pSK_SK	474
cl2267	ct2480	cn2721	pOP-EAP02297_EST_C_1_pBSK_SK	494
cl2267	ct2480	cn2721	pOP-EO06499_EST_C_1_pSK_SK	775
cl2268	ct2481	cn2722	pOP-CEO01808_EST_C_1_pSK_SK	248
cl2268	ct2481	cn2722	pOP-CNI02080_EST_C_1_pSK_SK	467
cl2268	ct2481	cn2722	pOP-EO02263_EST_C_1_pSK_SK	514
cl2268	ct2481	cn2722	pOP-EO03130_EST_C_1_pSK_SK	396
cl2268	ct2481	cn2722	pOP-EO05011_EST_C_1_pSK_SK	536
cl2268	ct2481	cn2722	pOP-EO06500_EST_C_1_pSK_SK	721
cl2269	ct2482	cn2723	pOP-CNH04750_EST_C_1_pSK_SK	467
cl2269	ct2482	cn2723	pOP-EAP02816_EST_C_1_pBSK_SK	556
cl2269	ct2482	cn2723	pOP-EO06218_EST_C_1_pSK_SK	591
cl2269	ct2482	cn2723	pOP-EO06504_EST_C_1_pSK_SK	859
cl2270	ct2483	cn2724	pOP-CNH02884_EST_C_1_pSK_SK	605
cl2270	ct2483	cn2724	pOP-EO03238_EST_C_1_pSK_SK	476
cl2270	ct2483	cn2724	pOP-EO06517_EST_C_1_pSK_SK	751
cl2271	ct2484	cn2725	pOP-CEM00156_EST_C_1_pSK_SK	280
cl2271	ct2484	cn2725	pOP-CNI01467_EST_C_1_pSK_SK	226
cl2271	ct2484	cn2725	pOP-EO02193_EST_C_1_pSK_SK	618
cl2271	ct2484	cn2725	pOP-EO02614_EST_C_1_pSK_SK	371
cl2271	ct2484	cn2725	pOP-EO02915_EST_C_1_pSK_SK	444
cl2271	ct2484	cn2725	pOP-EO03807_EST_C_1_pSK_SK	416
cl2271	ct2484	cn2725	pOP-EO04803_EST_C_1_pSK_SK	569
cl2271	ct2484	cn2725	pOP-EO05620_EST_C_1_pSK_SK	429
cl2271	ct2484	cn2725	pOP-EO05641_EST_C_1_pSK_SK	401
cl2271	ct2484	cn2725	pOP-EO06065_EST_C_1_pSK_SK	659
cl2271	ct2484	cn2725	pOP-EO06237_EST_C_1_pSK_SK	262
cl2271	ct2484	cn2725	pOP-EO06512_EST_C_1_pSK_SK	841
cl2271	ct2484	cn2725	pOP-EO06521_EST_C_1_pSK_SK	648
cl2271	ct2484	cn2725	pOP-EO06525_EST_C_1_pSK_SK	843
cl2271	ct2484	cn2725	pOP-EO07239_EST_C_1_pSK_SK	682
cl2271	ct2484	cn2726	pOP-CNI02033_EST_C_1_pSK_SK	432
cl2272	ct2485	cn2727	pOP-CEO01189_EST_C_1_pSK_SK	274
cl2272	ct2485	cn2727	pOP-CNH04769_EST_C_1_pSK_SK	503
cl2272	ct2485	cn2727	pOP-EO06254_EST_C_1_pSK_SK	654
cl2272	ct2485	cn2727	pOP-EO06275_EST_C_1_pSK_SK	690
cl2272	ct2485	cn2727	pOP-EO06408_EST_C_1_pSK_SK	684
cl2272	ct2485	cn2727	pOP-EO06531_EST_C_1_pSK_SK	657
cl2273	ct2486	cn2728	pOP-EAP01046_EST_C_1_pBSK_SK	247
cl2273	ct2486	cn2728	pOP-EAP02875_EST_C_1_pBSK_SK	221
cl2273	ct2486	cn2728	pOP-EO06533_EST_C_1_pSK_SK	834
cl2274	ct2487	cn2729	pOP-EO06534_EST_C_1_pSK_SK	787
cl2274	ct2487	cn2729	pOP-EO07856_EST_C_1_pSK_SK	779
cl2275	ct2488	cn2730	pOP-EO05253_EST_C_1_pSK_SK	534
cl2275	ct2488	cn2730	pOP-EO06539_EST_C_1_pSK_SK	901
cl2276	ct2489	cn2731	pOP-EO04406_EST_C_1_pSK_SK	530
cl2276	ct2489	cn2731	pOP-EO06540_EST_C_1_pSK_SK	666
cl2277	ct2490	cn2732	pOP-EO06509_EST_C_1_pSK_SK	748
cl2277	ct2490	cn2732	pOP-EO06543_EST_C_1_pSK_SK	811
cl2278	ct2491	cn2733	pOP-CEO03340_EST_C_1_pSK_SK	300
cl2278	ct2491	cn2733	pOP-EO06545_EST_C_1_pSK_SK	552

cl2279	ct2492	cn2734	pOP-EO06549_EST_C_1_pSK_SK	681
cl2279	ct2492	cn2734	pOP-EO06602_EST_C_1_pSK_SK	650
cl2280	ct2493	cn2735	pOP-EO05708_EST_C_1_pSK_SK	495
cl2280	ct2493	cn2735	pOP-EO06553_EST_C_1_pSK_SK	693
cl2281	ct2494	cn2736	pOP-CNI01547_EST_C_1_pSK_SK	272
cl2281	ct2494	cn2736	pOP-CNI02212_EST_C_1_pSK_SK	302
cl2281	ct2494	cn2736	pOP-EO06554_EST_C_1_pSK_SK	489
cl2282	ct2495	cn2737	pOP-CNI01154_EST_C_1_pSK_SK	530
cl2282	ct2495	cn2737	pOP-EO06560_EST_C_1_pSK_SK	515
cl2283	ct2496	cn2738	pOP-EO03076_EST_C_1_pSK_SK	337
cl2283	ct2496	cn2738	pOP-EO06570_EST_C_1_pSK_SK	736
cl2284	ct2497	cn2739	pOP-EO02356_EST_C_1_pSK_SK	716
cl2284	ct2497	cn2739	pOP-EO04548_EST_C_1_pSK_SK	455
cl2284	ct2497	cn2739	pOP-EO05939_EST_C_1_pSK_SK	425
cl2284	ct2497	cn2739	pOP-EO06576_EST_C_1_pSK_SK	849
cl2284	ct2497	cn2740	pOP-EAP00558_EST_C_1_pBSK_SK	449
cl2285	ct2498	cn2741	pOP-EO06578_EST_C_1_pSK_SK	838
cl2285	ct2498	cn2741	pOP-EO06585_EST_C_1_pSK_SK	867
cl2286	ct2499	cn2742	pOP-CNI01319_EST_C_1_pSK_SK	449
cl2286	ct2499	cn2742	pOP-EO06589_EST_C_1_pSK_SK	715
cl2287	ct2500	cn2743	pOP-EO02264_EST_C_1_pSK_SK	478
cl2287	ct2500	cn2743	pOP-EO02439_EST_C_1_pSK_SK	464
cl2287	ct2500	cn2743	pOP-EO05905_EST_C_1_pSK_SK	558
cl2287	ct2500	cn2743	pOP-EO06599_EST_C_1_pSK_SK	872
cl2288	ct2501	cn2744	pOP-CNIP00365_EST_C_1_pSK_SK	427
cl2288	ct2501	cn2744	pOP-EO06607_EST_C_1_pSK_SK	798
cl2289	ct2502	cn2745	pOP-CEO01104_EST_C_1_pSK_SK	443
cl2289	ct2502	cn2745	pOP-EO06608_EST_C_1_pSK_SK	742
cl2290	ct2503	cn2746	pOP-EN00525_EST_C_1_pSK_SK	390
cl2290	ct2503	cn2746	pOP-EO06609_EST_C_1_pSK_SK	784
cl2291	ct2504	cn2747	pOP-EO02059_EST_C_1_pSK_SK	542
cl2291	ct2504	cn2747	pOP-EO06610_EST_C_1_pSK_SK	779
cl2292	ct2505	cn2748	pOP-CEO02784_EST_C_1_pSK_SK	449
cl2292	ct2505	cn2748	pOP-CEO02864_EST_C_1_pSK_SK	462
cl2292	ct2505	cn2748	pOP-EO05208_EST_C_1_pSK_SK	497
cl2292	ct2505	cn2748	pOP-EO05997_EST_C_1_pSK_SK	495
cl2292	ct2505	cn2748	pOP-EO06611_EST_C_1_pSK_SK	495
cl2293	ct2506	cn2749	pOP-EO04884_EST_C_1_pSK_SK	454
cl2293	ct2506	cn2749	pOP-EO06612_EST_C_1_pSK_SK	802
cl2294	ct2507	cn2750	pOP-CNIP00972_EST_C_1_pSK_SK	353
cl2294	ct2507	cn2750	pOP-EAP03197_EST_C_1_pBSK_SK	690
cl2294	ct2507	cn2751	pOP-CEO03348_EST_C_1_pSK_SK	507
cl2294	ct2508	cn2752	pOP-EO02377_EST_C_1_pSK_SK	568
cl2294	ct2508	cn2752	pOP-EO04325_EST_C_1_pSK_SK	439
cl2294	ct2508	cn2752	pOP-EO06600_EST_C_1_pSK_SK	876
cl2294	ct2508	cn2752	pOP-EO06699_EST_C_1_pSK_SK	769
cl2294	ct2508	cn2752	pOP-EO06724_EST_C_1_pSK_SK	818
cl2295	ct2509	cn2753	pOP-EO02449_EST_C_1_pSK_SK	460
cl2295	ct2509	cn2753	pOP-EO06332_EST_C_1_pSK_SK	841
cl2295	ct2509	cn2754	pOP-EO06701_EST_C_1_pSK_SK	475
cl2296	ct2510	cn2755	pOP-EO03930_EST_C_1_pSK_SK	523
cl2296	ct2510	cn2755	pOP-EO06711_EST_C_1_pSK_SK	753
cl2296	ct2510	cn2755	pOP-EO06712_EST_C_1_pSK_SK	858
cl2296	ct2510	cn2755	pOP-EO07273_EST_C_1_pSK_SK	296
cl2297	ct2511	cn2756	pOP-EO06710_EST_C_1_pSK_SK	685
cl2297	ct2511	cn2756	pOP-EO06713_EST_C_1_pSK_SK	823

cl2298	ct2512	cn2757	pOP-CEMP00006_EST_C_1_pSK_SK	316
cl2298	ct2512	cn2757	pOP-CEO01762_EST_C_1_pSK_SK	151
cl2298	ct2512	cn2757	pOP-EO02143_EST_C_1_pSK_SK	440
cl2298	ct2512	cn2757	pOP-EO02762_EST_C_1_pSK_SK	437
cl2298	ct2512	cn2757	pOP-EO06480_EST_C_1_pSK_SK	734
cl2298	ct2512	cn2757	pOP-EO06558_EST_C_1_pSK_SK	757
cl2298	ct2512	cn2757	pOP-EO06714_EST_C_1_pSK_SK	413
cl2298	ct2512	cn2757	pOP-EO07667_EST_C_1_pSK_SK	779
cl2299	ct2513	cn2758	pOP-EO06706_EST_C_1_pSK_SK	792
cl2299	ct2513	cn2758	pOP-EO06717_EST_C_1_pSK_SK	790
cl2300	ct2514	cn2759	pOP-EO06704_EST_C_1_pSK_SK	721
cl2300	ct2514	cn2759	pOP-EO06719_EST_C_1_pSK_SK	755
cl2301	ct2515	cn2760	pOP-EO06702_EST_C_1_pSK_SK	720
cl2301	ct2515	cn2760	pOP-EO06703_EST_C_1_pSK_SK	326
cl2301	ct2515	cn2760	pOP-EO06721_EST_C_1_pSK_SK	743
cl2302	ct2516	cn2761	pOP-EO06698_EST_C_1_pSK_SK	561
cl2302	ct2516	cn2761	pOP-EO06725_EST_C_1_pSK_SK	668
cl2303	ct2517	cn2762	pOP-EO06697_EST_C_1_pSK_SK	659
cl2303	ct2517	cn2762	pOP-EO06726_EST_C_1_pSK_SK	659
cl2304	ct2518	cn2763	pOP-EO06696_EST_C_1_pSK_SK	714
cl2304	ct2518	cn2763	pOP-EO06727_EST_C_1_pSK_SK	733
cl2305	ct2519	cn2764	pOP-EO06695_EST_C_1_pSK_SK	752
cl2305	ct2519	cn2764	pOP-EO06728_EST_C_1_pSK_SK	825
cl2306	ct2520	cn2765	pOP-EO06694_EST_C_1_pSK_SK	705
cl2306	ct2520	cn2765	pOP-EO06729_EST_C_1_pSK_SK	750
cl2307	ct2521	cn2766	pOP-CEO03476_EST_C_1_pSK_SK	314
cl2307	ct2521	cn2766	pOP-CEO03712_EST_C_1_pSK_SK	337
cl2307	ct2521	cn2766	pOP-CNI01719_EST_C_1_pSK_SK	492
cl2307	ct2521	cn2766	pOP-EO04469_EST_C_1_pSK_SK	491
cl2307	ct2521	cn2766	pOP-EO06693_EST_C_1_pSK_SK	780
cl2307	ct2521	cn2766	pOP-EO06730_EST_C_1_pSK_SK	810
cl2308	ct2522	cn2767	pOP-EO04673_EST_C_1_pSK_SK	536
cl2308	ct2522	cn2767	pOP-EO06692_EST_C_1_pSK_SK	739
cl2308	ct2522	cn2767	pOP-EO06731_EST_C_1_pSK_SK	819
cl2309	ct2523	cn2768	pOP-EO05522_EST_C_1_pSK_SK	464
cl2309	ct2523	cn2768	pOP-EO06689_EST_C_1_pSK_SK	702
cl2309	ct2523	cn2768	pOP-EO06734_EST_C_1_pSK_SK	771
cl2310	ct2524	cn2769	pOP-EO06688_EST_C_1_pSK_SK	798
cl2310	ct2524	cn2769	pOP-EO06735_EST_C_1_pSK_SK	796
cl2311	ct2525	cn2770	pOP-EO06687_EST_C_1_pSK_SK	660
cl2311	ct2525	cn2770	pOP-EO06736_EST_C_1_pSK_SK	638
cl2312	ct2526	cn2771	pOP-EO05935_EST_C_1_pSK_SK	518
cl2312	ct2526	cn2771	pOP-EO06685_EST_C_1_pSK_SK	750
cl2312	ct2526	cn2771	pOP-EO06738_EST_C_1_pSK_SK	750
cl2312	ct2526	cn2772	pOP-CNH02204_EST_C_1_pSK_SK	554
cl2313	ct2527	cn2773	pOP-EO06682_EST_C_1_pSK_SK	701
cl2313	ct2527	cn2773	pOP-EO06741_EST_C_1_pSK_SK	720
cl2314	ct2528	cn2774	pOP-CAP00100_EST_C_1_pBSK_SK	660
cl2314	ct2528	cn2774	pOP-EAP01329_EST_C_1_pBSK_SK	534
cl2314	ct2528	cn2774	pOP-EAP03234_EST_C_1_pBSK_SK	444
cl2314	ct2528	cn2774	pOP-EO06681_EST_C_1_pSK_SK	583
cl2314	ct2528	cn2774	pOP-EO06742_EST_C_1_pSK_SK	592
cl2315	ct2529	cn2775	pOP-EO06680_EST_C_1_pSK_SK	544
cl2315	ct2529	cn2775	pOP-EO06743_EST_C_1_pSK_SK	544
cl2316	ct2530	cn2776	pOP-EO06677_EST_C_1_pSK_SK	655
cl2316	ct2530	cn2776	pOP-EO06746_EST_C_1_pSK_SK	658

cl2317	ct2531	cn2777	pOP-EO06675_EST_C_1_pSK_SK	737
cl2317	ct2531	cn2777	pOP-EO06748_EST_C_1_pSK_SK	818
cl2318	ct2532	cn2778	pOP-EO06674_EST_C_1_pSK_SK	719
cl2318	ct2532	cn2778	pOP-EO06749_EST_C_1_pSK_SK	834
cl2319	ct2533	cn2779	pOP-EO06673_EST_C_1_pSK_SK	599
cl2319	ct2533	cn2779	pOP-EO06750_EST_C_1_pSK_SK	642
cl2320	ct2534	cn2780	pOP-EO06668_EST_C_1_pSK_SK	776
cl2320	ct2534	cn2780	pOP-EO06755_EST_C_1_pSK_SK	736
cl2321	ct2535	cn2781	pOP-EO06666_EST_C_1_pSK_SK	677
cl2321	ct2535	cn2781	pOP-EO06757_EST_C_1_pSK_SK	848
cl2322	ct2536	cn2782	pOP-CNH02206_EST_C_1_pSK_SK	553
cl2322	ct2536	cn2782	pOP-CNH03463_EST_C_1_pSK_SK	555
cl2322	ct2536	cn2782	pOP-CNH04170	531
cl2322	ct2536	cn2782	pOP-EO06431_EST_C_1_pSK_SK	714
cl2322	ct2536	cn2782	pOP-EO06665_EST_C_1_pSK_SK	610
cl2322	ct2536	cn2782	pOP-EO06758_EST_C_1_pSK_SK	814
cl2323	ct2537	cn2783	pOP-EO06664_EST_C_1_pSK_SK	642
cl2323	ct2537	cn2783	pOP-EO06759_EST_C_1_pSK_SK	677
cl2324	ct2538	cn2784	pOP-EO06662_EST_C_1_pSK_SK	797
cl2324	ct2538	cn2784	pOP-EO06761_EST_C_1_pSK_SK	838
cl2325	ct2539	cn2785	pOP-EO02687_EST_C_1_pSK_SK	448
cl2325	ct2540	cn2786	pOP-CNH05089_EST_C_1_pSK_SK	408
cl2325	ct2540	cn2786	pOP-EO06661_EST_C_1_pSK_SK	703
cl2325	ct2540	cn2786	pOP-EO06762_EST_C_1_pSK_SK	547
cl2325	ct2541	cn2787	pOP-EO02036_EST_C_1_pSK_SK	537
cl2325	ct2541	cn2787	pOP-EO03563_EST_C_1_pSK_SK	449
cl2325	ct2541	cn2787	pOP-EO04356_EST_C_1_pSK_SK	440
cl2325	ct2541	cn2787	pOP-EO04455_EST_C_1_pSK_SK	522
cl2326	ct2542	cn2788	pOP-CNH02082_EST_C_1_pSK_SK	459
cl2326	ct2542	cn2788	pOP-EO06659_EST_C_1_pSK_SK	768
cl2326	ct2542	cn2788	pOP-EO06764_EST_C_1_pSK_SK	703
cl2327	ct2543	cn2789	pOP-CEO03321_EST_C_1_pSK_SK	315
cl2327	ct2543	cn2789	pOP-EO06658_EST_C_1_pSK_SK	798
cl2327	ct2543	cn2789	pOP-EO06765_EST_C_1_pSK_SK	695
cl2328	ct2544	cn2790	pOP-EO06655_EST_C_1_pSK_SK	539
cl2328	ct2544	cn2790	pOP-EO06768_EST_C_1_pSK_SK	611
cl2329	ct2545	cn2791	pOP-EO06654_EST_C_1_pSK_SK	571
cl2329	ct2545	cn2791	pOP-EO06769_EST_C_1_pSK_SK	571
cl2330	ct2546	cn2792	pOP-EO06652_EST_C_1_pSK_SK	664
cl2330	ct2546	cn2792	pOP-EO06771_EST_C_1_pSK_SK	668
cl2331	ct2547	cn2793	pOP-EO06651_EST_C_1_pSK_SK	227
cl2331	ct2547	cn2793	pOP-EO06772_EST_C_1_pSK_SK	305
cl2332	ct2548	cn2794	pOP-EO02223_EST_C_1_pSK_SK	552
cl2332	ct2549	cn2795	pOP-EO06647_EST_C_1_pSK_SK	704
cl2332	ct2549	cn2795	pOP-EO06776_EST_C_1_pSK_SK	853
cl2333	ct2550	cn2796	pOP-EAP00635_EST_C_1_pBSK_SK	605
cl2333	ct2550	cn2796	pOP-EO06646_EST_C_1_pSK_SK	468
cl2333	ct2550	cn2796	pOP-EO06777_EST_C_1_pSK_SK	460
cl2334	ct2551	cn2797	pOP-EO06643_EST_C_1_pSK_SK	498
cl2334	ct2551	cn2797	pOP-EO06780_EST_C_1_pSK_SK	494
cl2335	ct2552	cn2798	pOP-EO06639_EST_C_1_pSK_SK	680
cl2335	ct2552	cn2798	pOP-EO06784_EST_C_1_pSK_SK	790
cl2336	ct2553	cn2799	pOP-EO06638_EST_C_1_pSK_SK	764
cl2336	ct2553	cn2799	pOP-EO06785_EST_C_1_pSK_SK	883
cl2337	ct2554	cn2800	pOP-EO06636_EST_C_1_pSK_SK	706
cl2337	ct2554	cn2800	pOP-EO06787_EST_C_1_pSK_SK	781

cl2338	ct2555	cn2801	pOP-EO06635_EST_C_1_pSK_SK	509
cl2338	ct2555	cn2801	pOP-EO06788_EST_C_1_pSK_SK	488
cl2339	ct2556	cn2802	pOP-EAP03685_EST_C_1_pBSK_SK	503
cl2339	ct2556	cn2802	pOP-EO06634_EST_C_1_pSK_SK	729
cl2339	ct2556	cn2802	pOP-EO06789_EST_C_1_pSK_SK	685
cl2340	ct2557	cn2803	pOP-EO06633_EST_C_1_pSK_SK	675
cl2340	ct2557	cn2803	pOP-EO06790_EST_C_1_pSK_SK	753
cl2341	ct2558	cn2804	pOP-EO06632_EST_C_1_pSK_SK	672
cl2341	ct2558	cn2804	pOP-EO06791_EST_C_1_pSK_SK	799
cl2342	ct2559	cn2805	pOP-CNH01020_EST_C_1_pSK_SK	269
cl2342	ct2559	cn2805	pOP-CNHP00068_EST_C_1_pSK_SK	633
cl2342	ct2559	cn2805	pOP-CNHP00413_EST_C_1_pSK_SK	790
cl2342	ct2559	cn2805	pOP-EAP01629_EST_C_1_pBSK_SK	564
cl2342	ct2559	cn2805	pOP-EO03009_EST_C_1_pSK_SK	442
cl2342	ct2559	cn2805	pOP-EO06511_EST_C_1_pSK_SK	741
cl2342	ct2559	cn2805	pOP-EO06631_EST_C_1_pSK_SK	396
cl2342	ct2559	cn2805	pOP-EO06792_EST_C_1_pSK_SK	383
cl2343	ct2560	cn2806	pOP-EO06630_EST_C_1_pSK_SK	659
cl2343	ct2560	cn2806	pOP-EO06793_EST_C_1_pSK_SK	916
cl2344	ct2561	cn2807	pOP-CEO03511_EST_C_1_pSK_SK	369
cl2344	ct2561	cn2807	pOP-CNH02332_EST_C_1_pSK_SK	559
cl2344	ct2561	cn2807	pOP-EAP00786_EST_C_1_pBSK_SK	358
cl2344	ct2561	cn2807	pOP-EO06628_EST_C_1_pSK_SK	764
cl2344	ct2561	cn2807	pOP-EO06795_EST_C_1_pSK_SK	770
cl2345	ct2562	cn2808	pOP-EO06625_EST_C_1_pSK_SK	735
cl2345	ct2562	cn2808	pOP-EO06798_EST_C_1_pSK_SK	842
cl2346	ct2563	cn2809	pOP-EO06624_EST_C_1_pSK_SK	646
cl2346	ct2563	cn2809	pOP-EO06799_EST_C_1_pSK_SK	767
cl2347	ct2564	cn2810	pOP-EO06623_EST_C_1_pSK_SK	747
cl2347	ct2564	cn2810	pOP-EO06800_EST_C_1_pSK_SK	811
cl2348	ct2565	cn2811	pOP-EO06621_EST_C_1_pSK_SK	526
cl2348	ct2565	cn2811	pOP-EO06802_EST_C_1_pSK_SK	525
cl2349	ct2566	cn2812	pOP-EO06620_EST_C_1_pSK_SK	763
cl2349	ct2566	cn2812	pOP-EO06803_EST_C_1_pSK_SK	843
cl2350	ct2567	cn2813	pOP-EO06618_EST_C_1_pSK_SK	574
cl2350	ct2567	cn2813	pOP-EO06805_EST_C_1_pSK_SK	574
cl2351	ct2568	cn2814	pOP-EO06617_EST_C_1_pSK_SK	799
cl2351	ct2568	cn2814	pOP-EO06806_EST_C_1_pSK_SK	793
cl2352	ct2569	cn2815	pOP-EO05530_EST_C_1_pSK_SK	523
cl2352	ct2569	cn2815	pOP-EO06808_EST_C_1_pSK_SK	554
cl2353	ct2570	cn2816	pOP-EO02936_EST_C_1_pSK_SK	350
cl2353	ct2570	cn2816	pOP-EO04365_EST_C_1_pSK_SK	492
cl2353	ct2571	cn2817	pOP-EO04600_EST_C_1_pSK_SK	541
cl2353	ct2571	cn2817	pOP-EO06811_EST_C_1_pSK_SK	715
cl2353	ct2571	cn2817	pOP-EO08071_EST_C_1_pSK_SK	546
cl2354	ct2572	cn2818	pOP-CEO01615_EST_C_1_pSK_SK	259
cl2354	ct2572	cn2818	pOP-EO06817_EST_C_1_pSK_SK	549
cl2355	ct2573	cn2819	pOP-EAP01812_EST_C_1_pBSK_SK	504
cl2355	ct2573	cn2819	pOP-EO06835_EST_C_1_pSK_SK	546
cl2356	ct2574	cn2820	pOP-CBP00087_EST_C_1_pBSK_SK	279
cl2356	ct2574	cn2820	pOP-CBP00219_EST_C_1_pBSK_SK	336
cl2356	ct2574	cn2820	pOP-EO06838_EST_C_1_pSK_SK	109
cl2357	ct2575	cn2821	pOP-CEMP00023_EST_C_1_pSK_SK	326
cl2357	ct2575	cn2821	pOP-CNI01821_EST_C_1_pSK_SK	486
cl2357	ct2575	cn2821	pOP-EAP01945_EST_C_1_pBSK_SK	342
cl2357	ct2575	cn2821	pOP-EAP02318_EST_C_1_pBSK_SK	565

cl2357	ct2575	cn2821	pOP-EAP03774_EST_C_1_pBSK_SK	460
cl2357	ct2575	cn2821	pOP-EO02196_EST_C_1_pSK_SK	425
cl2357	ct2575	cn2821	pOP-EO06849_EST_C_1_pSK_SK	793
cl2357	ct2575	cn2822	pOP-CNH04464	671
cl2357	ct2575	cn2823	pOP-EAP02091_EST_C_1_pBSK_SK	183
cl2358	ct2576	cn2824	pOP-CAP00251_EST_C_1_pBSK_SK	570
cl2358	ct2576	cn2824	pOP-EO06852_EST_C_1_pSK_SK	120
cl2359	ct2577	cn2825	pOP-CEO00812_EST_C_1_pSK_SK	245
cl2359	ct2577	cn2825	pOP-EO06857_EST_C_1_pSK_SK	493
cl2360	ct2578	cn2826	pOP-CEO02957_EST_C_1_pSK_SK	332
cl2360	ct2578	cn2826	pOP-CEO03490_EST_C_1_pSK_SK	240
cl2360	ct2578	cn2826	pOP-EO06869_EST_C_1_pSK_SK	635
cl2361	ct2579	cn2827	pOP-EO06359_EST_C_1_pSK_SK	749
cl2361	ct2579	cn2827	pOP-EO06873_EST_C_1_pSK_SK	786
cl2362	ct2580	cn2828	pOP-EO02129_EST_C_1_pSK_SK	492
cl2362	ct2580	cn2828	pOP-EO04278_EST_C_1_pSK_SK	344
cl2362	ct2580	cn2828	pOP-EO06888_EST_C_1_pSK_SK	362
cl2363	ct2581	cn2829	pOP-EAP02270_EST_C_1_pBSK_SK	353
cl2363	ct2581	cn2829	pOP-EO06892_EST_C_1_pSK_SK	569
cl2364	ct2582	cn2830	pOP-EO06897_EST_C_1_pSK_SK	721
cl2364	ct2582	cn2831	pOP-CNH00545_EST_C_1_pSK_SK	675
cl2365	ct2583	cn2832	pOP-EO05662_EST_C_1_pSK_SK	399
cl2365	ct2583	cn2832	pOP-EO06815_EST_C_1_pSK_SK	696
cl2365	ct2583	cn2832	pOP-EO06900_EST_C_1_pSK_SK	747
cl2366	ct2584	cn2833	pOP-CNHP00360_EST_C_1_pSK_SK	652
cl2366	ct2584	cn2833	pOP-CNI02078_EST_C_1_pSK_SK	605
cl2366	ct2584	cn2833	pOP-EO06905_EST_C_1_pSK_SK	632
cl2367	ct2585	cn2834	pOP-CNI01097_EST_C_1_pSK_SK	360
cl2367	ct2585	cn2834	pOP-EAP01188_EST_C_1_pBSK_SK	123
cl2367	ct2585	cn2834	pOP-EAP03137_EST_C_1_pBSK_SK	470
cl2367	ct2585	cn2834	pOP-EO06395_EST_C_1_pSK_SK	648
cl2367	ct2585	cn2834	pOP-EO06912_EST_C_1_pSK_SK	672
cl2368	ct2586	cn2835	pOP-CEO03408_EST_C_1_pSK_SK	321
cl2368	ct2586	cn2835	pOP-EO06952_EST_C_1_pSK_SK	569
cl2369	ct2587	cn2836	pOP-EO04054_EST_C_1_pSK_SK	506
cl2369	ct2587	cn2836	pOP-EO04596_EST_C_1_pSK_SK	549
cl2369	ct2587	cn2836	pOP-EO06953_EST_C_1_pSK_SK	681
cl2370	ct2588	cn2837	pOP-CNH03443_EST_C_1_pSK_SK	439
cl2370	ct2588	cn2837	pOP-CNI01119_EST_C_1_pSK_SK	298
cl2370	ct2588	cn2837	pOP-EO06126_EST_C_1_pSK_SK	664
cl2370	ct2588	cn2837	pOP-EO06956_EST_C_1_pSK_SK	633
cl2371	ct2589	cn2838	pOP-EO06960_EST_C_1_pSK_SK	741
cl2371	ct2590	cn2839	pOP-CNH05029_EST_C_1_pSK_SK	363
cl2371	ct2591	cn2840	pOP-EAP01338_EST_C_1_pBSK_SK	433
cl2371	ct2592	cn2841	pOP-EO03629_EST_C_1_pSK_SK	427
cl2372	ct2593	cn2842	pOP-EO06079_EST_C_1_pSK_SK	584
cl2372	ct2594	cn2843	pOP-CEO00990_EST_C_1_pSK_SK	399
cl2372	ct2594	cn2843	pOP-CEO02919_EST_C_1_pSK_SK	326
cl2372	ct2594	cn2843	pOP-EN00758_EST_C_1_pSK_SK	432
cl2372	ct2594	cn2843	pOP-EO02187_EST_C_1_pSK_SK	602
cl2372	ct2594	cn2843	pOP-EO02292_EST_C_1_pSK_SK	534
cl2372	ct2594	cn2843	pOP-EO02817_EST_C_1_pSK_SK	277
cl2372	ct2594	cn2843	pOP-EO03025_EST_C_1_pSK_SK	484
cl2372	ct2594	cn2843	pOP-EO04001_EST_C_1_pSK_SK	599
cl2372	ct2594	cn2843	pOP-EO04158_EST_C_1_pSK_SK	482
cl2372	ct2594	cn2843	pOP-EO04795_EST_C_1_pSK_SK	551

cl2372	ct2594	cn2843	pOP-EO05280_EST_C_1_pSK_SK	581
cl2372	ct2594	cn2843	pOP-EO05933_EST_C_1_pSK_SK	568
cl2372	ct2594	cn2843	pOP-EO06011_EST_C_1_pSK_SK	553
cl2372	ct2594	cn2843	pOP-EO06649_EST_C_1_pSK_SK	653
cl2372	ct2594	cn2843	pOP-EO06774_EST_C_1_pSK_SK	725
cl2372	ct2594	cn2843	pOP-EO06932_EST_C_1_pSK_SK	711
cl2372	ct2594	cn2843	pOP-EO06967_EST_C_1_pSK_SK	785
cl2373	ct2595	cn2844	pOP-EN00168_EST_C_1_pSK_SK	576
cl2373	ct2595	cn2844	pOP-EN00508_EST_C_1_pSK_SK	513
cl2373	ct2595	cn2844	pOP-EO06977_EST_C_1_pSK_SK	799
cl2374	ct2596	cn2845	pOP-CEO03734_EST_C_1_pSK_SK	376
cl2374	ct2596	cn2845	pOP-EO04511_EST_C_1_pSK_SK	520
cl2374	ct2596	cn2845	pOP-EO04584_EST_C_1_pSK_SK	449
cl2374	ct2596	cn2845	pOP-EO05079_EST_C_1_pSK_SK	460
cl2374	ct2596	cn2845	pOP-EO06988_EST_C_1_pSK_SK	636
cl2375	ct2597	cn2846	pOP-CAP05004_EST_C_1_pBSK_SK	580
cl2375	ct2597	cn2846	pOP-EO02068_EST_C_1_pSK_SK	463
cl2375	ct2597	cn2846	pOP-EO02413_EST_C_1_pSK_SK	319
cl2375	ct2597	cn2846	pOP-EO03151_EST_C_1_pSK_SK	465
cl2375	ct2597	cn2846	pOP-EO03655_EST_C_1_pSK_SK	476
cl2375	ct2597	cn2846	pOP-EO04030_EST_C_1_pSK_SK	532
cl2375	ct2597	cn2846	pOP-EO05739_EST_C_1_pSK_SK	515
cl2375	ct2597	cn2846	pOP-EO06459_EST_C_1_pSK_SK	665
cl2375	ct2597	cn2846	pOP-EO06993_EST_C_1_pSK_SK	746
cl2376	ct2598	cn2847	pOP-EN00717_EST_C_1_pSK_SK	428
cl2376	ct2598	cn2847	pOP-EN00844_EST_C_1_pSK_SK	534
cl2376	ct2598	cn2847	pOP-EO06627_EST_C_1_pSK_SK	758
cl2376	ct2598	cn2847	pOP-EO06796_EST_C_1_pSK_SK	679
cl2376	ct2598	cn2847	pOP-EO06996_EST_C_1_pSK_SK	760
cl2377	ct2599	cn2848	pOP-EO02217_EST_C_1_pSK_SK	415
cl2377	ct2599	cn2848	pOP-EO02640_EST_C_1_pSK_SK	339
cl2377	ct2599	cn2848	pOP-EO03637_EST_C_1_pSK_SK	311
cl2377	ct2599	cn2848	pOP-EO06882_EST_C_1_pSK_SK	733
cl2377	ct2599	cn2848	pOP-EO07002_EST_C_1_pSK_SK	689
cl2378	ct2600	cn2849	pOP-EO03612_EST_C_1_pSK_SK	329
cl2378	ct2600	cn2849	pOP-EO07003_EST_C_1_pSK_SK	566
cl2379	ct2601	cn2850	pOP-CNH00985_EST_C_1_pSK_SK	554
cl2379	ct2601	cn2850	pOP-CNH03175_EST_C_1_pSK_SK	574
cl2379	ct2601	cn2850	pOP-EAP03808_EST_C_1_pBSK_SK	618
cl2379	ct2601	cn2850	pOP-EN00227_EST_C_1_pSK_SK	439
cl2379	ct2601	cn2850	pOP-EN00321_EST_C_1_pSK_SK	557
cl2379	ct2601	cn2850	pOP-EN00476_EST_C_1_pSK_SK	510
cl2379	ct2601	cn2850	pOP-EN00506_EST_C_1_pSK_SK	510
cl2379	ct2601	cn2850	pOP-EN00654_EST_C_1_pSK_SK	547
cl2379	ct2601	cn2850	pOP-EN00814_EST_C_1_pSK_SK	668
cl2379	ct2601	cn2850	pOP-EN00870_EST_C_1_pSK_SK	544
cl2379	ct2601	cn2850	pOP-EO02986_EST_C_1_pSK_SK	372
cl2379	ct2601	cn2850	pOP-EO05099_EST_C_1_pSK_SK	511
cl2379	ct2601	cn2850	pOP-EO05567_EST_C_1_pSK_SK	497
cl2379	ct2601	cn2850	pOP-EO05917_EST_C_1_pSK_SK	610
cl2379	ct2601	cn2850	pOP-EO06005_EST_C_1_pSK_SK	522
cl2379	ct2601	cn2850	pOP-EO06019_EST_C_1_pSK_SK	483
cl2379	ct2601	cn2850	pOP-EO06640_EST_C_1_pSK_SK	675
cl2379	ct2601	cn2850	pOP-EO06783_EST_C_1_pSK_SK	797
cl2379	ct2601	cn2850	pOP-EO06826_EST_C_1_pSK_SK	743
cl2379	ct2601	cn2850	pOP-EO06883_EST_C_1_pSK_SK	739

cl2379	ct2601	cn2850	pOP-EO06885_EST_C_1_pSK_SK	758
cl2379	ct2601	cn2850	pOP-EO06974_EST_C_1_pSK_SK	725
cl2379	ct2601	cn2850	pOP-EO07006_EST_C_1_pSK_SK	636
cl2379	ct2601	cn2850	pOP-EO08102_EST_C_1_pSK_SK	557
cl2380	ct2602	cn2851	pOP-EO05908_EST_C_1_pSK_SK	621
cl2380	ct2602	cn2851	pOP-EO07007_EST_C_1_pSK_SK	699
cl2381	ct2603	cn2852	pOP-CEO03374_EST_C_1_pSK_SK	312
cl2381	ct2603	cn2852	pOP-CNI01095_EST_C_1_pSK_SK	500
cl2381	ct2603	cn2852	pOP-EO03723_EST_C_1_pSK_SK	471
cl2381	ct2603	cn2852	pOP-EO07009_EST_C_1_pSK_SK	518
cl2382	ct2604	cn2853	pOP-CEO02197_EST_C_1_pSK_SK	223
cl2382	ct2604	cn2853	pOP-CEO02312_EST_C_1_pSK_SK	542
cl2382	ct2604	cn2853	pOP-CNIP00633_EST_C_1_pSK_SK	279
cl2382	ct2604	cn2853	pOP-EO05334_EST_C_1_pSK_SK	567
cl2382	ct2604	cn2853	pOP-EO07014_EST_C_1_pSK_SK	561
cl2383	ct2605	cn2854	pOP-EO03476_EST_C_1_pSK_SK	540
cl2383	ct2605	cn2854	pOP-EO07025_EST_C_1_pSK_SK	647
cl2384	ct2606	cn2855	pOP-EO04459_EST_C_1_pSK_SK	403
cl2384	ct2606	cn2855	pOP-EO07027_EST_C_1_pSK_SK	433
cl2385	ct2607	cn2856	pOP-CEO00654_EST_C_1_pSK_SK	111
cl2385	ct2607	cn2856	pOP-CEO00965_EST_C_1_pSK_SK	196
cl2385	ct2607	cn2856	pOP-CEO01531_EST_C_1_pSK_SK	282
cl2385	ct2607	cn2856	pOP-CEO01556_EST_C_1_pSK_SK	252
cl2385	ct2607	cn2856	pOP-CEO01575_EST_C_1_pSK_SK	264
cl2385	ct2607	cn2856	pOP-CEO01639_EST_C_1_pSK_SK	242
cl2385	ct2607	cn2856	pOP-CEO03216_EST_C_1_pSK_SK	296
cl2385	ct2607	cn2856	pOP-CEO03224_EST_C_1_pSK_SK	101
cl2385	ct2607	cn2856	pOP-CEO03657_EST_C_1_pSK_SK	260
cl2385	ct2607	cn2856	pOP-EO07033_EST_C_1_pSK_SK	406
cl2386	ct2608	cn2857	pOP-CNH01025_EST_C_1_pSK_SK	478
cl2386	ct2608	cn2857	pOP-CNH02697_EST_C_1_pSK_SK	684
cl2386	ct2608	cn2857	pOP-EO07037_EST_C_1_pSK_SK	636
cl2387	ct2609	cn2858	pOP-EO06552_EST_C_1_pSK_SK	821
cl2387	ct2609	cn2858	pOP-EO07038_EST_C_1_pSK_SK	602
cl2388	ct2610	cn2859	pOP-CEM00161_EST_C_1_pSK_SK	420
cl2388	ct2610	cn2859	pOP-CEO01285	491
cl2388	ct2610	cn2859	pOP-CEO01751_EST_C_1_pSK_SK	372
cl2388	ct2610	cn2859	pOP-EO07043_EST_C_1_pSK_SK	659
cl2389	ct2611	cn2860	pOP-EO04422_EST_C_1_pSK_SK	531
cl2389	ct2611	cn2860	pOP-EO06004_EST_C_1_pSK_SK	621
cl2389	ct2611	cn2860	pOP-EO07045_EST_C_1_pSK_SK	276
cl2390	ct2612	cn2861	pOP-EO02241_EST_C_1_pSK_SK	368
cl2390	ct2612	cn2861	pOP-EO03366_EST_C_1_pSK_SK	339
cl2390	ct2612	cn2861	pOP-EO06524_EST_C_1_pSK_SK	635
cl2390	ct2612	cn2861	pOP-EO07046_EST_C_1_pSK_SK	631
cl2390	ct2612	cn2862	pOP-EO03530_EST_C_1_pSK_SK	464
cl2391	ct2613	cn2863	pOP-CEO00634_EST_C_1_pSK_SK	240
cl2391	ct2613	cn2863	pOP-EO07053_EST_C_1_pSK_SK	379
cl2392	ct2614	cn2864	pOP-CEO02705_EST_C_1_pSK_SK	199
cl2392	ct2614	cn2864	pOP-EO04445_EST_C_1_pSK_SK	524
cl2392	ct2614	cn2864	pOP-EO07056_EST_C_1_pSK_SK	512
cl2393	ct2615	cn2865	pOP-EO04960_EST_C_1_pSK_SK	520
cl2393	ct2615	cn2865	pOP-EO07057_EST_C_1_pSK_SK	613
cl2394	ct2616	cn2866	pOP-CBP00191_EST_C_1_pBSK_SK	520
cl2394	ct2616	cn2866	pOP-CBP00192_EST_C_1_pBSK_SK	558
cl2394	ct2616	cn2866	pOP-CNH01991_EST_C_1_pSK_SK	586

cl2394	ct2616	cn2866	pOP-CNH04760_EST_C_1_pSK_SK	525
cl2394	ct2616	cn2866	pOP-EO03987_EST_C_1_pSK_SK	492
cl2394	ct2616	cn2866	pOP-EO04141_EST_C_1_pSK_SK	322
cl2394	ct2616	cn2866	pOP-EO07065_EST_C_1_pSK_SK	627
cl2395	ct2617	cn2867	pOP-EN00226_EST_C_1_pSK_SK	452
cl2395	ct2617	cn2867	pOP-EO07071_EST_C_1_pSK_SK	514
cl2396	ct2618	cn2868	pOP-EO06433_EST_C_1_pSK_SK	362
cl2396	ct2618	cn2868	pOP-EO07077_EST_C_1_pSK_SK	362
cl2397	ct2619	cn2869	pOP-CEO03592_EST_C_1_pSK_SK	223
cl2397	ct2619	cn2869	pOP-CNI01757_EST_C_1_pSK_SK	488
cl2397	ct2619	cn2869	pOP-EO07078_EST_C_1_pSK_SK	652
cl2398	ct2620	cn2870	pOP-CNHP00121_EST_C_1_pSK_SK	508
cl2398	ct2620	cn2870	pOP-CNHP00251_EST_C_1_pSK_SK	464
cl2398	ct2621	cn2871	pOP-CNI01917_EST_C_1_pSK_SK	554
cl2398	ct2621	cn2871	pOP-EO07081_EST_C_1_pSK_SK	625
cl2399	ct2622	cn2872	pOP-CNHP00281_EST_C_1_pSK_SK	553
cl2399	ct2622	cn2872	pOP-EO02842_EST_C_1_pSK_SK	446
cl2399	ct2622	cn2872	pOP-EO07084_EST_C_1_pSK_SK	624
cl2400	ct2623	cn2873	pOP-EN00548_EST_C_1_pSK_SK	225
cl2400	ct2623	cn2873	pOP-EO07090_EST_C_1_pSK_SK	585
cl2401	ct2624	cn2874	pOP-EO06419_EST_C_1_pSK_SK	744
cl2401	ct2624	cn2874	pOP-EO07092_EST_C_1_pSK_SK	541
cl2402	ct2625	cn2875	pOP-CNH00720_EST_C_1_pSK_SK	685
cl2402	ct2625	cn2875	pOP-EO07100_EST_C_1_pSK_SK	521
cl2403	ct2626	cn2876	pOP-EN00139_EST_C_1_pSK_SK	581
cl2403	ct2626	cn2876	pOP-EO06411_EST_C_1_pSK_SK	824
cl2403	ct2626	cn2876	pOP-EO07115_EST_C_1_pSK_SK	619
cl2404	ct2627	cn2877	pOP-CNH00761_EST_C_1_pSK_SK	449
cl2404	ct2627	cn2877	pOP-CNIP00016_EST_C_1_pSK_SK	626
cl2404	ct2627	cn2877	pOP-EO07122_EST_C_1_pSK_SK	243
cl2405	ct2628	cn2878	pOP-EO05037_EST_C_1_pSK_SK	523
cl2405	ct2628	cn2878	pOP-EO07135_EST_C_1_pSK_SK	540
cl2406	ct2629	cn2879	pOP-CNI01844_EST_C_1_pSK_SK	285
cl2406	ct2629	cn2879	pOP-EO07147_EST_C_1_pSK_SK	598
cl2407	ct2630	cn2880	pOP-EO04734_EST_C_1_pSK_SK	507
cl2407	ct2630	cn2880	pOP-EO07151_EST_C_1_pSK_SK	617
cl2408	ct2631	cn2881	pOP-EO07152_EST_C_1_pSK_SK	667
cl2408	ct2631	cn2882	pOP-CEO02607_EST_C_1_pSK_SK	241
cl2409	ct2632	cn2883	pOP-CNH01113_EST_C_1_pSK_SK	353
cl2409	ct2632	cn2883	pOP-EO05516_EST_C_1_pSK_SK	508
cl2409	ct2632	cn2883	pOP-EO07157_EST_C_1_pSK_SK	565
cl2410	ct2633	cn2884	pOP-EN00762_EST_C_1_pSK_SK	434
cl2410	ct2633	cn2884	pOP-EO05654_EST_C_1_pSK_SK	501
cl2410	ct2633	cn2884	pOP-EO07162_EST_C_1_pSK_SK	543
cl2410	ct2634	cn2885	pOP-CAP05001_EST_C_1_pBSK_SK	650
cl2410	ct2634	cn2885	pOP-EN00297_EST_C_1_pSK_SK	551
cl2410	ct2634	cn2885	pOP-EO02699_EST_C_1_pSK_SK	449
cl2410	ct2634	cn2885	pOP-EO03653_EST_C_1_pSK_SK	417
cl2410	ct2634	cn2885	pOP-EO06216_EST_C_1_pSK_SK	694
cl2411	ct2635	cn2886	pOP-EO05957_EST_C_1_pSK_SK	567
cl2411	ct2635	cn2886	pOP-EO07178_EST_C_1_pSK_SK	581
cl2412	ct2636	cn2887	pOP-EO03584_EST_C_1_pSK_SK	466
cl2412	ct2636	cn2887	pOP-EO04837_EST_C_1_pSK_SK	503
cl2412	ct2636	cn2887	pOP-EO07183_EST_C_1_pSK_SK	622
cl2413	ct2637	cn2888	pOP-CEO02519_EST_C_1_pSK_SK	140
cl2413	ct2637	cn2888	pOP-CNH01789_EST_C_1_pSK_SK	524

cl2413	ct2637	cn2888	pOP-CNH01979_EST_C_1_pSK_SK	487
cl2413	ct2637	cn2888	pOP-CNH03414_EST_C_1_pSK_SK	487
cl2413	ct2637	cn2888	pOP-EN00881_EST_C_1_pSK_SK	531
cl2413	ct2637	cn2888	pOP-EO06024_EST_C_1_pSK_SK	617
cl2413	ct2637	cn2888	pOP-EO06095_EST_C_1_pSK_SK	489
cl2413	ct2637	cn2888	pOP-EO07012_EST_C_1_pSK_SK	603
cl2413	ct2637	cn2888	pOP-EO07129_EST_C_1_pSK_SK	612
cl2413	ct2637	cn2888	pOP-EO07184_EST_C_1_pSK_SK	636
cl2413	ct2637	cn2888	pOP-EO07311_EST_C_1_pSK_SK	779
cl2414	ct2638	cn2889	pOP-CAP00354_EST_C_1_pBSK_SK	497
cl2414	ct2638	cn2889	pOP-CNIP00150_EST_C_1_pSK_SK	527
cl2414	ct2638	cn2889	pOP-CNIP00457_EST_C_1_pSK_SK	221
cl2414	ct2638	cn2889	pOP-CNNP00033_EST_C_1_pBSK_SK	171
cl2414	ct2638	cn2889	pOP-EO02487_EST_C_1_pSK_SK	513
cl2414	ct2638	cn2889	pOP-EO07195_EST_C_1_pSK_SK	717
cl2415	ct2639	cn2890	pOP-CNH00654_EST_C_1_pSK_SK	535
cl2415	ct2639	cn2890	pOP-EO07198_EST_C_1_pSK_SK	779
cl2416	ct2640	cn2891	pOP-EN00733_EST_C_1_pSK_SK	590
cl2416	ct2640	cn2891	pOP-EO07201_EST_C_1_pSK_SK	781
cl2417	ct2641	cn2892	pOP-EAP01077_EST_C_1_pBSK_SK	425
cl2417	ct2641	cn2892	pOP-EAP02221_EST_C_1_pBSK_SK	269
cl2417	ct2641	cn2892	pOP-EAP02893_EST_C_1_pBSK_SK	402
cl2417	ct2641	cn2892	pOP-EO04802_EST_C_1_pSK_SK	574
cl2417	ct2641	cn2892	pOP-EO04847_EST_C_1_pSK_SK	424
cl2417	ct2641	cn2892	pOP-EO07203_EST_C_1_pSK_SK	752
cl2418	ct2642	cn2893	pOP-CBP00226_EST_C_1_pBSK_SK	531
cl2418	ct2642	cn2893	pOP-CEO02836_EST_C_1_pSK_SK	318
cl2418	ct2642	cn2893	pOP-CEO03705_EST_C_1_pSK_SK	263
cl2418	ct2642	cn2893	pOP-CNH01059_EST_C_1_pSK_SK	380
cl2418	ct2642	cn2893	pOP-EO02777_EST_C_1_pSK_SK	332
cl2418	ct2642	cn2893	pOP-EO06667_EST_C_1_pSK_SK	619
cl2418	ct2642	cn2893	pOP-EO06756_EST_C_1_pSK_SK	614
cl2418	ct2642	cn2893	pOP-EO07209_EST_C_1_pSK_SK	720
cl2419	ct2643	cn2894	pOP-EO02854_EST_C_1_pSK_SK	445
cl2419	ct2643	cn2894	pOP-EO07215_EST_C_1_pSK_SK	744
cl2420	ct2644	cn2895	pOP-EO02664_EST_C_1_pSK_SK	454
cl2420	ct2644	cn2895	pOP-EO04701_EST_C_1_pSK_SK	545
cl2420	ct2644	cn2895	pOP-EO06446_EST_C_1_pSK_SK	586
cl2420	ct2644	cn2895	pOP-EO07217_EST_C_1_pSK_SK	561
cl2421	ct2645	cn2896	pOP-CNI01325_EST_C_1_pSK_SK	146
cl2421	ct2645	cn2896	pOP-EAP01071_EST_C_1_pBSK_SK	437
cl2421	ct2645	cn2896	pOP-EAP02889_EST_C_1_pBSK_SK	408
cl2421	ct2645	cn2896	pOP-EO07228_EST_C_1_pSK_SK	790
cl2422	ct2646	cn2897	pOP-EN00765_EST_C_1_pSK_SK	567
cl2422	ct2646	cn2897	pOP-EO07229_EST_C_1_pSK_SK	719
cl2423	ct2647	cn2898	pOP-EO06264_EST_C_1_pSK_SK	659
cl2423	ct2647	cn2898	pOP-EO07246_EST_C_1_pSK_SK	713
cl2423	ct2647	cn2899	pOP-CNH05055_EST_C_1_pSK_SK	628
cl2424	ct2648	cn2900	pOP-CEO03435_EST_C_1_pSK_SK	472
cl2424	ct2648	cn2900	pOP-EO07250_EST_C_1_pSK_SK	663
cl2425	ct2649	cn2901	pOP-CEO03306_EST_C_1_pSK_SK	578
cl2425	ct2649	cn2901	pOP-EAP00078_EST_C_1_pBSK_SK	362
cl2425	ct2649	cn2901	pOP-EAP00693_EST_C_1_pBSK_SK	651
cl2425	ct2649	cn2901	pOP-EAP00812_EST_C_1_pBSK_SK	414
cl2425	ct2649	cn2901	pOP-EAP01033_EST_C_1_pBSK_SK	180
cl2425	ct2649	cn2901	pOP-EAP01447_EST_C_1_pBSK_SK	179

cl2425	ct2649	cn2901	pOP-EO02186_EST_C_1_pSK_SK	434
cl2425	ct2649	cn2901	pOP-EO03814_EST_C_1_pSK_SK	482
cl2425	ct2649	cn2901	pOP-EO04609_EST_C_1_pSK_SK	551
cl2425	ct2649	cn2901	pOP-EO04836_EST_C_1_pSK_SK	574
cl2425	ct2649	cn2901	pOP-EO07263_EST_C_1_pSK_SK	675
cl2425	ct2649	cn2901	pOP-EO07593_EST_C_1_pSK_SK	747
cl2425	ct2649	cn2901	pOP-EO07594_EST_C_1_pSK_SK	486
cl2426	ct2650	cn2902	pOP-CNH01298_EST_C_1_pSK_SK	582
cl2426	ct2650	cn2902	pOP-CNH02263_EST_C_1_pSK_SK	698
cl2426	ct2650	cn2902	pOP-CNH04372	809
cl2426	ct2650	cn2902	pOP-EO06230_EST_C_1_pSK_SK	604
cl2426	ct2650	cn2902	pOP-EO07268_EST_C_1_pSK_SK	748
cl2427	ct2651	cn2903	pOP-EO04920_EST_C_1_pSK_SK	490
cl2427	ct2651	cn2903	pOP-EO07279_EST_C_1_pSK_SK	629
cl2428	ct2652	cn2904	pOP-EO07197_EST_C_1_pSK_SK	723
cl2428	ct2652	cn2904	pOP-EO07283_EST_C_1_pSK_SK	719
cl2429	ct2653	cn2905	pOP-EO04671_EST_C_1_pSK_SK	534
cl2429	ct2653	cn2905	pOP-EO07291_EST_C_1_pSK_SK	751
cl2430	ct2654	cn2906	pOP-CNI01451_EST_C_1_pSK_SK	161
cl2430	ct2654	cn2906	pOP-EO07292_EST_C_1_pSK_SK	479
cl2431	ct2655	cn2907	pOP-EO06505_EST_C_1_pSK_SK	765
cl2431	ct2656	cn2908	pOP-EO07293_EST_C_1_pSK_SK	787
cl2432	ct2657	cn2909	pOP-CNH01626_EST_C_1_pSK_SK	609
cl2432	ct2657	cn2909	pOP-CNH03559_EST_C_1_pSK_SK	350
cl2432	ct2657	cn2909	pOP-EO02820_EST_C_1_pSK_SK	378
cl2432	ct2657	cn2909	pOP-EO05265_EST_C_1_pSK_SK	584
cl2432	ct2657	cn2909	pOP-EO07297_EST_C_1_pSK_SK	781
cl2433	ct2658	cn2910	pOP-CEO00627_EST_C_1_pSK_SK	413
cl2433	ct2658	cn2910	pOP-CNH00703_EST_C_1_pSK_SK	626
cl2433	ct2658	cn2910	pOP-CNIP00550_EST_C_1_pSK_SK	117
cl2433	ct2658	cn2910	pOP-EO02260_EST_C_1_pSK_SK	304
cl2433	ct2658	cn2910	pOP-EO03480_EST_C_1_pSK_SK	540
cl2433	ct2658	cn2910	pOP-EO07299_EST_C_1_pSK_SK	733
cl2434	ct2659	cn2911	pOP-EAP01417_EST_C_1_pBSK_SK	277
cl2434	ct2659	cn2911	pOP-EAP02192_EST_C_1_pBSK_SK	190
cl2434	ct2659	cn2911	pOP-EN00690_EST_C_1_pSK_SK	537
cl2434	ct2659	cn2911	pOP-EO02114_EST_C_1_pSK_SK	631
cl2434	ct2659	cn2911	pOP-EO02509_EST_C_1_pSK_SK	458
cl2434	ct2659	cn2911	pOP-EO05093_EST_C_1_pSK_SK	505
cl2434	ct2659	cn2911	pOP-EO05365_EST_C_1_pSK_SK	546
cl2434	ct2659	cn2911	pOP-EO07016_EST_C_1_pSK_SK	666
cl2434	ct2659	cn2911	pOP-EO07307_EST_C_1_pSK_SK	839
cl2434	ct2659	cn2912	pOP-CEO01765_EST_C_1_pSK_SK	331
cl2435	ct2660	cn2913	pOP-CNH01969_EST_C_1_pSK_SK	586
cl2435	ct2660	cn2913	pOP-CNH02848_EST_C_1_pSK_SK	614
cl2435	ct2660	cn2913	pOP-CNH03008_EST_C_1_pSK_SK	531
cl2435	ct2660	cn2913	pOP-EO06428_EST_C_1_pSK_SK	753
cl2435	ct2660	cn2913	pOP-EO07321_EST_C_1_pSK_SK	771
cl2436	ct2661	cn2914	pOP-EO05137_EST_C_1_pSK_SK	536
cl2436	ct2661	cn2914	pOP-EO07328_EST_C_1_pSK_SK	790
cl2437	ct2662	cn2915	pOP-CAP00298_EST_C_1_pBSK_SK	566
cl2437	ct2662	cn2915	pOP-EO07330_EST_C_1_pSK_SK	816
cl2438	ct2663	cn2916	pOP-CNH03317_EST_C_1_pSK_SK	579
cl2438	ct2663	cn2916	pOP-EN00451_EST_C_1_pSK_SK	513
cl2438	ct2663	cn2916	pOP-EO02890_EST_C_1_pSK_SK	443
cl2438	ct2663	cn2916	pOP-EO07333_EST_C_1_pSK_SK	681

cl2439	ct2664	cn2917	pOP-EN00497_EST_C_1_pSK_SK	430
cl2439	ct2664	cn2917	pOP-EO07335_EST_C_1_pSK_SK	557
cl2440	ct2665	cn2918	pOP-EO03142_EST_C_1_pSK_SK	450
cl2440	ct2665	cn2918	pOP-EO04416_EST_C_1_pSK_SK	528
cl2440	ct2665	cn2918	pOP-EO06890_EST_C_1_pSK_SK	729
cl2440	ct2665	cn2918	pOP-EO07337_EST_C_1_pSK_SK	689
cl2441	ct2666	cn2919	pOP-CNH00589_EST_C_1_pSK_SK	558
cl2441	ct2666	cn2919	pOP-CNH02317_EST_C_1_pSK_SK	557
cl2441	ct2667	cn2920	pOP-EO06562_EST_C_1_pSK_SK	836
cl2441	ct2667	cn2920	pOP-EO06564_EST_C_1_pSK_SK	736
cl2441	ct2667	cn2920	pOP-EO07339_EST_C_1_pSK_SK	814
cl2442	ct2668	cn2921	pOP-CNH00564_EST_C_1_pSK_SK	665
cl2442	ct2668	cn2922	pOP-CEO02507_EST_C_1_pSK_SK	422
cl2442	ct2669	cn2923	pOP-EO07144_EST_C_1_pSK_SK	571
cl2442	ct2669	cn2924	pOP-EO04477_EST_C_1_pSK_SK	311
cl2442	ct2670	cn2925	pOP-EAP03203_EST_C_1_pBSK_SK	508
cl2442	ct2670	cn2925	pOP-EO06377_EST_C_1_pSK_SK	705
cl2442	ct2670	cn2926	pOP-CNH02946_EST_C_1_pSK_SK	614
cl2442	ct2670	cn2927	pOP-CEO03307_EST_C_1_pSK_SK	290
cl2442	ct2671	cn2928	pOP-CNI01762_EST_C_1_pSK_SK	312
cl2442	ct2671	cn2928	pOP-CNI01816_EST_C_1_pSK_SK	177
cl2442	ct2671	cn2928	pOP-EAP01063_EST_C_1_pBSK_SK	192
cl2442	ct2671	cn2928	pOP-EO05563_EST_C_1_pSK_SK	348
cl2442	ct2672	cn2929	pOP-CBP00054_EST_C_1_pBSK_SK	330
cl2442	ct2672	cn2929	pOP-CBP00069_EST_C_1_pBSK_SK	376
cl2442	ct2672	cn2929	pOP-CBP00072_EST_C_1_pBSK_SK	271
cl2442	ct2672	cn2929	pOP-CBP00170_EST_C_1_pBSK_SK	334
cl2442	ct2672	cn2929	pOP-CBP00217_EST_C_1_pBSK_SK	330
cl2442	ct2672	cn2929	pOP-CNI01563_EST_C_1_pSK_SK	184
cl2442	ct2672	cn2929	pOP-CNIP01051_EST_C_1_pSK_SK	195
cl2442	ct2672	cn2929	pOP-CNIP04079_EST_C_1_pSK_SK	142
cl2442	ct2673	cn2930	pOP-CNH02834_EST_C_1_pSK_SK	532
cl2442	ct2673	cn2930	pOP-CNH04462	784
cl2442	ct2673	cn2930	pOP-CNH04791_EST_C_1_pSK_SK	503
cl2442	ct2673	cn2930	pOP-CNH04918_EST_C_1_pSK_SK	658
cl2442	ct2673	cn2930	pOP-CNHP00200_EST_C_1_pSK_SK	467
cl2442	ct2673	cn2930	pOP-CNIP00210_EST_C_1_pSK_SK	496
cl2442	ct2673	cn2930	pOP-EAP01633_EST_C_1_pBSK_SK	152
cl2442	ct2673	cn2930	pOP-EAP01684_EST_C_1_pBSK_SK	612
cl2442	ct2673	cn2930	pOP-EAP01959_EST_C_1_pBSK_SK	458
cl2442	ct2673	cn2930	pOP-EAP03816_EST_C_1_pBSK_SK	442
cl2442	ct2674	cn2931	pOP-CNH02415_EST_C_1_pSK_SK	520
cl2442	ct2674	cn2931	pOP-CNH02583_EST_C_1_pSK_SK	529
cl2442	ct2674	cn2931	pOP-CNH03531_EST_C_1_pSK_SK	351
cl2442	ct2674	cn2931	pOP-CNH04710_EST_C_1_pSK_SK	591
cl2442	ct2674	cn2931	pOP-CNHP00429_EST_C_1_pSK_SK	726
cl2442	ct2674	cn2931	pOP-CNI01229_EST_C_1_pSK_SK	726
cl2442	ct2674	cn2931	pOP-CNI01604_EST_C_1_pSK_SK	482
cl2442	ct2674	cn2931	pOP-EO06392_EST_C_1_pSK_SK	766
cl2442	ct2674	cn2931	pOP-EO07174_EST_C_1_pSK_SK	574
cl2442	ct2674	cn2932	pOP-CEO00911_EST_C_1_pSK_SK	470
cl2442	ct2675	cn2933	pOP-CEO03104_EST_C_1_pSK_SK	172
cl2442	ct2675	cn2933	pOP-CEO03423_EST_C_1_pSK_SK	239
cl2442	ct2675	cn2933	pOP-CEO03466_EST_C_1_pSK_SK	210
cl2442	ct2675	cn2933	pOP-CNH02195_EST_C_1_pSK_SK	582
cl2442	ct2675	cn2933	pOP-CNIP00140_EST_C_1_pSK_SK	140

cl2442	ct2675	cn2933	pOP-CNIP00278_EST_C_1_pSK_SK	111
cl2442	ct2675	cn2933	pOP-EO02457_EST_C_1_pSK_SK	291
cl2442	ct2675	cn2933	pOP-EO07308_EST_C_1_pSK_SK	363
cl2442	ct2675	cn2933	pOP-EO07346_EST_C_1_pSK_SK	192
cl2442	ct2675	cn2933	pOP-EO08313_EST_C_1_pSK_SK	522
cl2442	ct2676	cn2934	pOP-CNH00615_EST_C_1_pSK_SK	716
cl2442	ct2676	cn2934	pOP-CNH00706_EST_C_1_pSK_SK	677
cl2442	ct2676	cn2934	pOP-CNH04208	494
cl2442	ct2676	cn2934	pOP-CNH04646	850
cl2442	ct2676	cn2934	pOP-CNHP00151_EST_C_1_pSK_SK	315
cl2442	ct2676	cn2934	pOP-CNHP00254_EST_C_1_pSK_SK	551
cl2442	ct2676	cn2934	pOP-CNI01819_EST_C_1_pSK_SK	501
cl2442	ct2676	cn2934	pOP-EAP05046_EST_C_1_pBSK_SK	319
cl2442	ct2676	cn2934	pOP-EO02527_EST_C_1_pSK_SK	525
cl2442	ct2676	cn2934	pOP-EO03325_EST_C_1_pSK_SK	392
cl2442	ct2676	cn2934	pOP-EO03414_EST_C_1_pSK_SK	452
cl2442	ct2676	cn2934	pOP-EO04217_EST_C_1_pSK_SK	544
cl2442	ct2676	cn2934	pOP-EO04622_EST_C_1_pSK_SK	551
cl2442	ct2676	cn2934	pOP-EO04813_EST_C_1_pSK_SK	567
cl2442	ct2676	cn2934	pOP-EO05220_EST_C_1_pSK_SK	532
cl2442	ct2677	cn2935	pOP-CAP00183_EST_C_1_pBSK_SK	670
cl2442	ct2677	cn2935	pOP-CEO02141_EST_C_1_pSK_SK	586
cl2442	ct2677	cn2935	pOP-CNH00577_EST_C_1_pSK_SK	632
cl2442	ct2677	cn2935	pOP-CNH00651_EST_C_1_pSK_SK	557
cl2442	ct2677	cn2935	pOP-CNH00762_EST_C_1_pSK_SK	445
cl2442	ct2677	cn2935	pOP-CNH01098_EST_C_1_pSK_SK	295
cl2442	ct2677	cn2935	pOP-CNH01401_EST_C_1_pSK_SK	597
cl2442	ct2677	cn2935	pOP-CNH01500_EST_C_1_pSK_SK	557
cl2442	ct2677	cn2935	pOP-CNH02096_EST_C_1_pSK_SK	548
cl2442	ct2677	cn2935	pOP-CNH02188_EST_C_1_pSK_SK	513
cl2442	ct2677	cn2935	pOP-CNH02711_EST_C_1_pSK_SK	368
cl2442	ct2677	cn2935	pOP-CNH02794_EST_C_1_pSK_SK	522
cl2442	ct2677	cn2935	pOP-CNH04687	806
cl2442	ct2677	cn2935	pOP-CNH04732_EST_C_1_pSK_SK	455
cl2442	ct2677	cn2935	pOP-CNHP00314_EST_C_1_pSK_SK	630
cl2442	ct2677	cn2935	pOP-CNHP00483_EST_C_1_pSK_SK	621
cl2442	ct2677	cn2935	pOP-CNIP01068_EST_C_1_pSK_SK	564
cl2442	ct2677	cn2935	pOP-EO05871_EST_C_1_pSK_SK	511
cl2442	ct2677	cn2936	pOP-CNH01103_EST_C_1_pSK_SK	325
cl2442	ct2677	cn2936	pOP-CNHP00220_EST_C_1_pSK_SK	396
cl2442	ct2677	cn2936	pOP-CNHP00326_EST_C_1_pSK_SK	620
cl2442	ct2677	cn2936	pOP-EO03993_EST_C_1_pSK_SK	478
cl2442	ct2677	cn2936	pOP-EO04164_EST_C_1_pSK_SK	498
cl2442	ct2677	cn2937	pOP-CNI01258_EST_C_1_pSK_SK	576
cl2442	ct2677	cn2938	pOP-EO04430_EST_C_1_pSK_SK	488
cl2442	ct2677	cn2939	pOP-CEO00813_EST_C_1_pSK_SK	319
cl2442	ct2677	cn2939	pOP-CNI01441_EST_C_1_pSK_SK	362
cl2442	ct2677	cn2939	pOP-CNI02178_EST_C_1_pSK_SK	331
cl2442	ct2677	cn2940	pOP-EO04279_EST_C_1_pSK_SK	375
cl2442	ct2677	cn2941	pOP-EO02151_EST_C_1_pSK_SK	207
cl2442	ct2677	cn2942	pOP-EO08498_EST_C_1_pSK_SK	122
cl2442	ct2678	cn2943	pOP-CEO01312	157
cl2442	ct2678	cn2943	pOP-CEO01955_EST_C_1_pSK_SK	404
cl2442	ct2678	cn2943	pOP-CEO02437_EST_C_1_pSK_SK	733
cl2442	ct2678	cn2943	pOP-CEO02783_EST_C_1_pSK_SK	138
cl2442	ct2678	cn2943	pOP-CEO02880_EST_C_1_pSK_SK	408

cl2442	ct2678	cn2943	pOP-CEO03336_EST_C_1_pSK_SK	503
cl2442	ct2678	cn2943	pOP-CNI01978_EST_C_1_pSK_SK	409
cl2442	ct2678	cn2943	pOP-CNIP00281_EST_C_1_pSK_SK	179
cl2442	ct2678	cn2943	pOP-EAP01407_EST_C_1_pBSK_SK	180
cl2442	ct2678	cn2943	pOP-EAP02995_EST_C_1_pBSK_SK	497
cl2442	ct2678	cn2943	pOP-EN00277_EST_C_1_pSK_SK	524
cl2442	ct2678	cn2943	pOP-EN00599_EST_C_1_pSK_SK	490
cl2442	ct2678	cn2943	pOP-EO02130_EST_C_1_pSK_SK	499
cl2442	ct2678	cn2943	pOP-EO02262_EST_C_1_pSK_SK	488
cl2442	ct2678	cn2943	pOP-EO03143_EST_C_1_pSK_SK	474
cl2442	ct2678	cn2943	pOP-EO03311_EST_C_1_pSK_SK	389
cl2442	ct2678	cn2943	pOP-EO03361_EST_C_1_pSK_SK	433
cl2442	ct2678	cn2943	pOP-EO03574_EST_C_1_pSK_SK	545
cl2442	ct2678	cn2943	pOP-EO03726_EST_C_1_pSK_SK	528
cl2442	ct2678	cn2943	pOP-EO04717_EST_C_1_pSK_SK	504
cl2442	ct2678	cn2943	pOP-EO04844_EST_C_1_pSK_SK	512
cl2442	ct2678	cn2943	pOP-EO04906_EST_C_1_pSK_SK	512
cl2442	ct2678	cn2943	pOP-EO04915_EST_C_1_pSK_SK	465
cl2442	ct2678	cn2943	pOP-EO05094_EST_C_1_pSK_SK	491
cl2442	ct2678	cn2943	pOP-EO05095_EST_C_1_pSK_SK	481
cl2442	ct2678	cn2943	pOP-EO05282_EST_C_1_pSK_SK	501
cl2442	ct2678	cn2943	pOP-EO05508_EST_C_1_pSK_SK	499
cl2442	ct2678	cn2943	pOP-EO05824_EST_C_1_pSK_SK	428
cl2442	ct2678	cn2943	pOP-EO07392_EST_C_1_pSK_SK	748
cl2442	ct2678	cn2943	pOP-EO07562_EST_C_1_pSK_SK	667
cl2442	ct2678	cn2943	pOP-EO08245_EST_C_1_pSK_SK	401
cl2442	ct2678	cn2944	pOP-CNI01221_EST_C_1_pSK_SK	516
cl2442	ct2678	cn2945	pOP-CBP00050_EST_C_1_pBSK_SK	337
cl2442	ct2678	cn2945	pOP-CBP00056_EST_C_1_pBSK_SK	358
cl2442	ct2678	cn2945	pOP-CBP00117_EST_C_1_pBSK_SK	497
cl2442	ct2678	cn2945	pOP-CBP00137_EST_C_1_pBSK_SK	337
cl2442	ct2678	cn2945	pOP-CBP00146_EST_C_1_pBSK_SK	358
cl2442	ct2678	cn2945	pOP-CBP00266_EST_C_1_pBSK_SK	408
cl2442	ct2678	cn2945	pOP-EO04958_EST_C_1_pSK_SK	464
cl2442	ct2678	cn2946	pOP-CBP00107_EST_C_1_pBSK_SK	362
cl2442	ct2678	cn2947	pOP-CBP00204_EST_C_1_pBSK_SK	339
cl2442	ct2678	cn2948	pOP-CBP00059_EST_C_1_pBSK_SK	290
cl2442	ct2678	cn2948	pOP-CBP00184_EST_C_1_pBSK_SK	324
cl2442	ct2678	cn2949	pOP-CBP00224_EST_C_1_pBSK_SK	239
cl2442	ct2678	cn2950	pOP-CBP00002_EST_C_1_pBSK_SK	180
cl2442	ct2678	cn2950	pOP-CBP00030_EST_C_1_pBSK_SK	210
cl2442	ct2678	cn2950	pOP-CBP00044_EST_C_1_pBSK_SK	194
cl2442	ct2678	cn2950	pOP-CBP00064_EST_C_1_pBSK_SK	210
cl2442	ct2678	cn2950	pOP-CBP00267_EST_C_1_pBSK_SK	211
cl2442	ct2678	cn2950	pOP-CEO03751_EST_C_1_pSK_SK	129
cl2442	ct2678	cn2950	pOP-CNI01198_EST_C_1_pSK_SK	157
cl2442	ct2678	cn2950	pOP-CNI01302_EST_C_1_pSK_SK	238
cl2442	ct2678	cn2950	pOP-CNI01385_EST_C_1_pSK_SK	157
cl2442	ct2678	cn2950	pOP-CNI01552_EST_C_1_pSK_SK	207
cl2442	ct2678	cn2950	pOP-CNI01611_EST_C_1_pSK_SK	129
cl2442	ct2678	cn2950	pOP-CNI01669_EST_C_1_pSK_SK	128
cl2442	ct2678	cn2950	pOP-CNIP04066_EST_C_1_pSK_SK	157
cl2443	ct2679	cn2951	pOP-CEM00140_EST_C_1_pSK_SK	190
cl2443	ct2679	cn2951	pOP-EO07347_EST_C_1_pSK_SK	635
cl2444	ct2680	cn2952	pOP-EAP01397_EST_C_1_pBSK_SK	152
cl2444	ct2680	cn2952	pOP-EAP01421_EST_C_1_pBSK_SK	348

cl2444	ct2680	cn2952	pOP-EAP01661_EST_C_1_pBSK_SK	262
cl2444	ct2680	cn2952	pOP-EN00896_EST_C_1_pSK_SK	543
cl2444	ct2680	cn2952	pOP-EO06951_EST_C_1_pSK_SK	791
cl2444	ct2680	cn2952	pOP-EO07116_EST_C_1_pSK_SK	632
cl2444	ct2680	cn2952	pOP-EO07349_EST_C_1_pSK_SK	525
cl2444	ct2680	cn2952	pOP-EO07904_EST_C_1_pSK_SK	624
cl2445	ct2681	cn2953	pOP-CNI01501_EST_C_1_pSK_SK	425
cl2445	ct2681	cn2953	pOP-EO06415_EST_C_1_pSK_SK	779
cl2445	ct2681	cn2953	pOP-EO07353_EST_C_1_pSK_SK	845
cl2445	ct2682	cn2954	pOP-CNI02177_EST_C_1_pSK_SK	485
cl2445	ct2682	cn2954	pOP-EO02959_EST_C_1_pSK_SK	194
cl2445	ct2682	cn2954	pOP-EO03602_EST_C_1_pSK_SK	190
cl2445	ct2682	cn2954	pOP-EO04415_EST_C_1_pSK_SK	394
cl2445	ct2682	cn2954	pOP-EO04849_EST_C_1_pSK_SK	477
cl2445	ct2682	cn2954	pOP-EO05938_EST_C_1_pSK_SK	337
cl2445	ct2682	cn2954	pOP-EO06536_EST_C_1_pSK_SK	709
cl2446	ct2683	cn2955	pOP-EO02386_EST_C_1_pSK_SK	666
cl2446	ct2683	cn2955	pOP-EO05982_EST_C_1_pSK_SK	531
cl2446	ct2683	cn2955	pOP-EO07354_EST_C_1_pSK_SK	326
cl2447	ct2684	cn2956	pOP-CNIP00282_EST_C_1_pSK_SK	211
cl2447	ct2684	cn2956	pOP-EN00551_EST_C_1_pSK_SK	622
cl2447	ct2684	cn2956	pOP-EO02202_EST_C_1_pSK_SK	485
cl2447	ct2684	cn2956	pOP-EO02919_EST_C_1_pSK_SK	453
cl2447	ct2684	cn2956	pOP-EO03767_EST_C_1_pSK_SK	493
cl2447	ct2684	cn2956	pOP-EO07360_EST_C_1_pSK_SK	834
cl2447	ct2684	cn2957	pOP-EO06571_EST_C_1_pSK_SK	572
cl2448	ct2685	cn2958	pOP-CAP00057_EST_C_1_pBSK_SK	585
cl2448	ct2685	cn2958	pOP-EO04689_EST_C_1_pSK_SK	534
cl2448	ct2686	cn2959	pOP-CEO02008_EST_C_1_pSK_SK	321
cl2448	ct2686	cn2959	pOP-EO06976_EST_C_1_pSK_SK	789
cl2448	ct2687	cn2960	pOP-CNH04611	824
cl2448	ct2687	cn2960	pOP-CNH04673	330
cl2448	ct2687	cn2960	pOP-EO06514_EST_C_1_pSK_SK	712
cl2448	ct2688	cn2961	pOP-EO03597_EST_C_1_pSK_SK	427
cl2448	ct2688	cn2961	pOP-EO06660_EST_C_1_pSK_SK	729
cl2448	ct2688	cn2961	pOP-EO06763_EST_C_1_pSK_SK	756
cl2448	ct2688	cn2961	pOP-EO07361_EST_C_1_pSK_SK	752
cl2448	ct2689	cn2962	pOP-EO02031_EST_C_1_pSK_SK	584
cl2448	ct2689	cn2962	pOP-EO02194_EST_C_1_pSK_SK	627
cl2448	ct2689	cn2962	pOP-EO02427_EST_C_1_pSK_SK	466
cl2448	ct2689	cn2962	pOP-EO03739_EST_C_1_pSK_SK	461
cl2448	ct2689	cn2962	pOP-EO04018_EST_C_1_pSK_SK	355
cl2448	ct2689	cn2962	pOP-EO04066_EST_C_1_pSK_SK	485
cl2448	ct2689	cn2962	pOP-EO04160_EST_C_1_pSK_SK	396
cl2448	ct2689	cn2962	pOP-EO08066_EST_C_1_pSK_SK	578
cl2449	ct2690	cn2963	pOP-CNHP00219_EST_C_1_pSK_SK	553
cl2449	ct2690	cn2963	pOP-EO07363_EST_C_1_pSK_SK	820
cl2450	ct2691	cn2964	pOP-CNH02544_EST_C_1_pSK_SK	411
cl2450	ct2691	cn2964	pOP-CNH02789_EST_C_1_pSK_SK	424
cl2450	ct2691	cn2964	pOP-CNH03447_EST_C_1_pSK_SK	424
cl2450	ct2692	cn2965	pOP-EO07372_EST_C_1_pSK_SK	663
cl2450	ct2692	cn2966	pOP-CNI01908_EST_C_1_pSK_SK	472
cl2450	ct2692	cn2966	pOP-CNIP00997_EST_C_1_pSK_SK	523
cl2451	ct2693	cn2967	pOP-CNH02185_EST_C_1_pSK_SK	597
cl2451	ct2693	cn2967	pOP-EN00255_EST_C_1_pSK_SK	402
cl2451	ct2693	cn2967	pOP-EO05956_EST_C_1_pSK_SK	573

cl2451	ct2693	cn2967	pOP-EO07149_EST_C_1_pSK_SK	570
cl2451	ct2693	cn2967	pOP-EO07373_EST_C_1_pSK_SK	852
cl2452	ct2694	cn2968	pOP-CNI01296_EST_C_1_pSK_SK	317
cl2452	ct2695	cn2969	pOP-EO07377_EST_C_1_pSK_SK	881
cl2453	ct2696	cn2970	pOP-CEO03355_EST_C_1_pSK_SK	158
cl2453	ct2696	cn2970	pOP-CNH02986_EST_C_1_pSK_SK	514
cl2453	ct2696	cn2970	pOP-CNHP00136_EST_C_1_pSK_SK	507
cl2453	ct2696	cn2970	pOP-EAP01326_EST_C_1_pBSK_SK	530
cl2453	ct2696	cn2970	pOP-EN00716_EST_C_1_pSK_SK	461
cl2453	ct2696	cn2970	pOP-EO06279_EST_C_1_pSK_SK	679
cl2453	ct2696	cn2970	pOP-EO07379_EST_C_1_pSK_SK	693
cl2454	ct2697	cn2971	pOP-CEO03090_EST_C_1_pSK_SK	282
cl2454	ct2697	cn2971	pOP-EAP01257_EST_C_1_pBSK_SK	405
cl2454	ct2697	cn2971	pOP-EO05275_EST_C_1_pSK_SK	397
cl2454	ct2697	cn2971	pOP-EO07384_EST_C_1_pSK_SK	380
cl2455	ct2698	cn2972	pOP-EO05581_EST_C_1_pSK_SK	480
cl2455	ct2698	cn2972	pOP-EO06222_EST_C_1_pSK_SK	210
cl2455	ct2698	cn2972	pOP-EO07385_EST_C_1_pSK_SK	556
cl2456	ct2699	cn2973	pOP-CEM00065_EST_C_1_pSK_SK	412
cl2456	ct2699	cn2973	pOP-CEO01579_EST_C_1_pSK_SK	389
cl2456	ct2699	cn2973	pOP-CEO02879_EST_C_1_pSK_SK	393
cl2456	ct2699	cn2973	pOP-EO03489_EST_C_1_pSK_SK	473
cl2456	ct2699	cn2973	pOP-EO07395_EST_C_1_pSK_SK	430
cl2457	ct2700	cn2974	pOP-CEO01689_EST_C_1_pSK_SK	414
cl2457	ct2700	cn2974	pOP-CNIP00254_EST_C_1_pSK_SK	391
cl2457	ct2700	cn2974	pOP-EAP01939_EST_C_1_pBSK_SK	378
cl2457	ct2700	cn2974	pOP-EO07397_EST_C_1_pSK_SK	621
cl2458	ct2701	cn2975	pOP-EO05582_EST_C_1_pSK_SK	481
cl2458	ct2701	cn2975	pOP-EO07400_EST_C_1_pSK_SK	771
cl2459	ct2702	cn2976	pOP-CAP00387_EST_C_1_pBSK_SK	620
cl2459	ct2702	cn2976	pOP-CNH03498_EST_C_1_pSK_SK	587
cl2459	ct2702	cn2976	pOP-EO03386_EST_C_1_pSK_SK	454
cl2459	ct2702	cn2976	pOP-EO07404_EST_C_1_pSK_SK	666
cl2460	ct2703	cn2977	pOP-EO07418_EST_C_1_pSK_SK	727
cl2460	ct2703	cn2978	pOP-EN00201_EST_C_1_pSK_SK	517
cl2461	ct2704	cn2979	pOP-CNH02652_EST_C_1_pSK_SK	585
cl2461	ct2704	cn2979	pOP-CNH03223_EST_C_1_pSK_SK	542
cl2461	ct2704	cn2979	pOP-CNH03435_EST_C_1_pSK_SK	510
cl2461	ct2704	cn2979	pOP-EAP01881_EST_C_1_pBSK_SK	611
cl2461	ct2704	cn2979	pOP-EO07419_EST_C_1_pSK_SK	694
cl2462	ct2705	cn2980	pOP-EN00541_EST_C_1_pSK_SK	477
cl2462	ct2705	cn2980	pOP-EN00590_EST_C_1_pSK_SK	487
cl2462	ct2705	cn2980	pOP-EN00837_EST_C_1_pSK_SK	522
cl2462	ct2705	cn2980	pOP-EO03411_EST_C_1_pSK_SK	321
cl2462	ct2705	cn2980	pOP-EO04476_EST_C_1_pSK_SK	493
cl2462	ct2705	cn2980	pOP-EO05651_EST_C_1_pSK_SK	373
cl2462	ct2705	cn2980	pOP-EO05796_EST_C_1_pSK_SK	656
cl2462	ct2705	cn2980	pOP-EO06110_EST_C_1_pSK_SK	612
cl2462	ct2705	cn2980	pOP-EO06626_EST_C_1_pSK_SK	810
cl2462	ct2705	cn2980	pOP-EO06641_EST_C_1_pSK_SK	780
cl2462	ct2705	cn2980	pOP-EO06782_EST_C_1_pSK_SK	859
cl2462	ct2705	cn2980	pOP-EO06797_EST_C_1_pSK_SK	886
cl2462	ct2705	cn2980	pOP-EO07420_EST_C_1_pSK_SK	807
cl2463	ct2706	cn2981	pOP-EAP02739_EST_C_1_pBSK_SK	729
cl2463	ct2706	cn2981	pOP-EO07421_EST_C_1_pSK_SK	534
cl2464	ct2707	cn2982	pOP-EO07277_EST_C_1_pSK_SK	790

cl2464	ct2707	cn2982	pOP-EO07422_EST_C_1_pSK_SK	791
cl2465	ct2708	cn2983	pOP-CNH02333_EST_C_1_pSK_SK	560
cl2465	ct2708	cn2983	pOP-EO07424_EST_C_1_pSK_SK	671
cl2466	ct2709	cn2984	pOP-EO04281_EST_C_1_pSK_SK	473
cl2466	ct2709	cn2984	pOP-EO06320_EST_C_1_pSK_SK	612
cl2466	ct2709	cn2984	pOP-EO07441_EST_C_1_pSK_SK	738
cl2467	ct2710	cn2985	pOP-CAP00225_EST_C_1_pBSK_SK	640
cl2467	ct2710	cn2985	pOP-EN00464_EST_C_1_pSK_SK	510
cl2467	ct2710	cn2985	pOP-EO05711_EST_C_1_pSK_SK	435
cl2467	ct2710	cn2985	pOP-EO07443_EST_C_1_pSK_SK	778
cl2468	ct2711	cn2986	pOP-EO04059_EST_C_1_pSK_SK	531
cl2468	ct2711	cn2986	pOP-EO04139_EST_C_1_pSK_SK	451
cl2468	ct2711	cn2986	pOP-EO07451_EST_C_1_pSK_SK	817
cl2469	ct2712	cn2987	pOP-CBP00015_EST_C_1_pBSK_SK	596
cl2469	ct2712	cn2987	pOP-EO07457_EST_C_1_pSK_SK	846
cl2470	ct2713	cn2988	pOP-CEO02192_EST_C_1_pSK_SK	404
cl2470	ct2713	cn2988	pOP-CNIP00332_EST_C_1_pSK_SK	268
cl2470	ct2713	cn2988	pOP-EAP00121_EST_C_1_pBSK_SK	367
cl2470	ct2713	cn2988	pOP-EAP03341_EST_C_1_pBSK_SK	434
cl2470	ct2713	cn2988	pOP-EO02411_EST_C_1_pSK_SK	466
cl2470	ct2713	cn2988	pOP-EO03413_EST_C_1_pSK_SK	500
cl2470	ct2713	cn2988	pOP-EO07468_EST_C_1_pSK_SK	510
cl2471	ct2714	cn2989	pOP-CBP00183_EST_C_1_pBSK_SK	542
cl2471	ct2714	cn2989	pOP-CEO01060_EST_C_1_pSK_SK	256
cl2471	ct2714	cn2989	pOP-EO06479_EST_C_1_pSK_SK	816
cl2471	ct2714	cn2989	pOP-EO06683_EST_C_1_pSK_SK	764
cl2471	ct2714	cn2989	pOP-EO06740_EST_C_1_pSK_SK	775
cl2471	ct2714	cn2989	pOP-EO07469_EST_C_1_pSK_SK	504
cl2471	ct2714	cn2990	pOP-EO05844_EST_C_1_pSK_SK	603
cl2472	ct2715	cn2991	pOP-EO03704_EST_C_1_pSK_SK	354
cl2472	ct2715	cn2991	pOP-EO07475_EST_C_1_pSK_SK	774
cl2472	ct2715	cn2991	pOP-EO08011_EST_C_1_pSK_SK	342
cl2473	ct2716	cn2992	pOP-EO07066_EST_C_1_pSK_SK	479
cl2473	ct2717	cn2993	pOP-CAP00256_EST_C_1_pBSK_SK	624
cl2473	ct2717	cn2993	pOP-EO07476_EST_C_1_pSK_SK	848
cl2474	ct2718	cn2994	pOP-CBP00039_EST_C_1_pBSK_SK	524
cl2474	ct2718	cn2994	pOP-CBP00153_EST_C_1_pBSK_SK	564
cl2474	ct2718	cn2994	pOP-CEO01601_EST_C_1_pSK_SK	129
cl2474	ct2718	cn2994	pOP-CEO02514_EST_C_1_pSK_SK	153
cl2474	ct2718	cn2994	pOP-CNI01486_EST_C_1_pSK_SK	379
cl2474	ct2718	cn2994	pOP-CNI01814_EST_C_1_pSK_SK	277
cl2474	ct2718	cn2994	pOP-CNI01892_EST_C_1_pSK_SK	392
cl2474	ct2718	cn2994	pOP-CNI01956_EST_C_1_pSK_SK	455
cl2474	ct2718	cn2994	pOP-CNI01992_EST_C_1_pSK_SK	437
cl2474	ct2718	cn2994	pOP-CNIP00067_EST_C_1_pSK_SK	319
cl2474	ct2718	cn2994	pOP-CNIP00107_EST_C_1_pSK_SK	555
cl2474	ct2718	cn2994	pOP-CNIP00156_EST_C_1_pSK_SK	199
cl2474	ct2718	cn2994	pOP-CNIP00502_EST_C_1_pSK_SK	178
cl2474	ct2718	cn2994	pOP-CNIP04045_EST_C_1_pSK_SK	321
cl2474	ct2718	cn2994	pOP-EAP02104_EST_C_1_pBSK_SK	131
cl2474	ct2718	cn2994	pOP-EAP03165_EST_C_1_pBSK_SK	496
cl2474	ct2718	cn2994	pOP-EO02089_EST_C_1_pSK_SK	355
cl2474	ct2718	cn2994	pOP-EO03897_EST_C_1_pSK_SK	502
cl2474	ct2718	cn2994	pOP-EO04830_EST_C_1_pSK_SK	548
cl2474	ct2718	cn2994	pOP-EO05030_EST_C_1_pSK_SK	541
cl2474	ct2718	cn2995	pOP-EO07479_EST_C_1_pSK_SK	751

cl2475	ct2719	cn2996	pOP-EO07480_EST_C_1_pSK_SK	743
cl2475	ct2719	cn2996	pOP-EO07481_EST_C_1_pSK_SK	662
cl2476	ct2720	cn2997	pOP-CNH01901_EST_C_1_pSK_SK	575
cl2476	ct2720	cn2997	pOP-CNH02461_EST_C_1_pSK_SK	419
cl2476	ct2720	cn2997	pOP-CNH02712_EST_C_1_pSK_SK	589
cl2476	ct2720	cn2997	pOP-CNH04210	685
cl2476	ct2720	cn2997	pOP-CNH04574	707
cl2476	ct2720	cn2997	pOP-CNLP00017_EST_C_1_pSK_SK	541
cl2476	ct2720	cn2997	pOP-EO07485_EST_C_1_pSK_SK	735
cl2477	ct2721	cn2998	pOP-CNI01138_EST_C_1_pSK_SK	453
cl2477	ct2721	cn2998	pOP-CNI01139_EST_C_1_pSK_SK	470
cl2477	ct2721	cn2998	pOP-EO07488_EST_C_1_pSK_SK	687
cl2478	ct2722	cn2999	pOP-EO07496_EST_C_1_pSK_SK	746
cl2478	ct2722	cn2999	pOP-EO07497_EST_C_1_pSK_SK	679
cl2479	ct2723	cn3000	pOP-CEO02057_EST_C_1_pSK_SK	192
cl2479	ct2723	cn3000	pOP-EO07499_EST_C_1_pSK_SK	146
cl2480	ct2724	cn3001	pOP-CNH03346_EST_C_1_pSK_SK	654
cl2480	ct2724	cn3001	pOP-CNHP00252_EST_C_1_pSK_SK	549
cl2480	ct2724	cn3001	pOP-EAP01752_EST_C_1_pBSK_SK	309
cl2480	ct2724	cn3001	pOP-EO02136_EST_C_1_pSK_SK	457
cl2480	ct2724	cn3001	pOP-EO07504_EST_C_1_pSK_SK	748
cl2481	ct2725	cn3002	pOP-CNH01042_EST_C_1_pSK_SK	629
cl2481	ct2725	cn3002	pOP-CNH03376_EST_C_1_pSK_SK	587
cl2481	ct2725	cn3002	pOP-CNI01897_EST_C_1_pSK_SK	529
cl2481	ct2725	cn3002	pOP-EN00276_EST_C_1_pSK_SK	547
cl2481	ct2725	cn3002	pOP-EO07506_EST_C_1_pSK_SK	629
cl2482	ct2726	cn3003	pOP-CEO03136_EST_C_1_pSK_SK	351
cl2482	ct2726	cn3003	pOP-EO05532_EST_C_1_pSK_SK	517
cl2482	ct2726	cn3003	pOP-EO07514_EST_C_1_pSK_SK	785
cl2483	ct2727	cn3004	pOP-EO07516_EST_C_1_pSK_SK	486
cl2483	ct2727	cn3004	pOP-EO07517_EST_C_1_pSK_SK	497
cl2484	ct2728	cn3005	pOP-EO05278_EST_C_1_pSK_SK	564
cl2484	ct2728	cn3005	pOP-EO07520_EST_C_1_pSK_SK	759
cl2485	ct2729	cn3006	pOP-EO04397_EST_C_1_pSK_SK	355
cl2485	ct2729	cn3006	pOP-EO07524_EST_C_1_pSK_SK	720
cl2486	ct2730	cn3007	pOP-EO03307_EST_C_1_pSK_SK	292
cl2486	ct2730	cn3007	pOP-EO03438_EST_C_1_pSK_SK	432
cl2486	ct2730	cn3007	pOP-EO04541_EST_C_1_pSK_SK	464
cl2486	ct2730	cn3007	pOP-EO04859_EST_C_1_pSK_SK	511
cl2486	ct2730	cn3007	pOP-EO06910_EST_C_1_pSK_SK	703
cl2486	ct2730	cn3007	pOP-EO07236_EST_C_1_pSK_SK	751
cl2486	ct2730	cn3007	pOP-EO07450_EST_C_1_pSK_SK	743
cl2486	ct2730	cn3007	pOP-EO07527_EST_C_1_pSK_SK	493
cl2487	ct2731	cn3008	pOP-EAP03696_EST_C_1_pBSK_SK	589
cl2487	ct2731	cn3008	pOP-EO07529_EST_C_1_pSK_SK	698
cl2488	ct2732	cn3009	pOP-CEO03345_EST_C_1_pSK_SK	539
cl2488	ct2732	cn3009	pOP-CNH02059_EST_C_1_pSK_SK	597
cl2488	ct2732	cn3009	pOP-EAP02238_EST_C_1_pBSK_SK	229
cl2488	ct2732	cn3009	pOP-EO03306_EST_C_1_pSK_SK	253
cl2488	ct2732	cn3009	pOP-EO06603_EST_C_1_pSK_SK	886
cl2488	ct2732	cn3009	pOP-EO07543_EST_C_1_pSK_SK	772
cl2489	ct2733	cn3010	pOP-EO07535_EST_C_1_pSK_SK	530
cl2489	ct2733	cn3010	pOP-EO07550_EST_C_1_pSK_SK	812
cl2490	ct2734	cn3011	pOP-EO04011_EST_C_1_pSK_SK	354
cl2490	ct2734	cn3011	pOP-EO07213_EST_C_1_pSK_SK	352
cl2490	ct2734	cn3011	pOP-EO07554_EST_C_1_pSK_SK	771

cl2491	ct2735	cn3012	pOP-EO06183_EST_C_1_pSK_SK	444
cl2491	ct2735	cn3012	pOP-EO07557_EST_C_1_pSK_SK	459
cl2492	ct2736	cn3013	pOP-EO04315_EST_C_1_pSK_SK	363
cl2492	ct2736	cn3013	pOP-EO07558_EST_C_1_pSK_SK	787
cl2493	ct2737	cn3014	pOP-EAP02274_EST_C_1_pBSK_SK	615
cl2493	ct2737	cn3014	pOP-EO07560_EST_C_1_pSK_SK	812
cl2494	ct2738	cn3015	pOP-CEM00189_EST_C_1_pSK_SK	247
cl2494	ct2738	cn3015	pOP-EAP01492_EST_C_1_pBSK_SK	477
cl2494	ct2738	cn3015	pOP-EO02541_EST_C_1_pSK_SK	529
cl2494	ct2738	cn3015	pOP-EO07271_EST_C_1_pSK_SK	786
cl2494	ct2738	cn3015	pOP-EO07565_EST_C_1_pSK_SK	783
cl2494	ct2738	cn3015	pOP-EO07566_EST_C_1_pSK_SK	740
cl2495	ct2739	cn3016	pOP-EO07222_EST_C_1_pSK_SK	734
cl2495	ct2739	cn3016	pOP-EO07569_EST_C_1_pSK_SK	777
cl2496	ct2740	cn3017	pOP-EO03399_EST_C_1_pSK_SK	455
cl2496	ct2740	cn3017	pOP-EO07575_EST_C_1_pSK_SK	743
cl2497	ct2741	cn3018	pOP-EO02881_EST_C_1_pSK_SK	279
cl2497	ct2741	cn3018	pOP-EO05677_EST_C_1_pSK_SK	504
cl2497	ct2741	cn3018	pOP-EO07580_EST_C_1_pSK_SK	737
cl2498	ct2742	cn3019	pOP-EO06863_EST_C_1_pSK_SK	110
cl2498	ct2742	cn3019	pOP-EO07598_EST_C_1_pSK_SK	725
cl2499	ct2743	cn3020	pOP-EO02453_EST_C_1_pSK_SK	416
cl2499	ct2743	cn3020	pOP-EO07606_EST_C_1_pSK_SK	673
cl2500	ct2744	cn3021	pOP-CEO01048_EST_C_1_pSK_SK	199
cl2500	ct2744	cn3021	pOP-EO03619_EST_C_1_pSK_SK	478
cl2500	ct2744	cn3021	pOP-EO07622_EST_C_1_pSK_SK	587
cl2501	ct2745	cn3022	pOP-EO03286_EST_C_1_pSK_SK	254
cl2501	ct2745	cn3022	pOP-EO04083_EST_C_1_pSK_SK	304
cl2501	ct2745	cn3022	pOP-EO04213_EST_C_1_pSK_SK	544
cl2501	ct2745	cn3022	pOP-EO07625_EST_C_1_pSK_SK	781
cl2502	ct2746	cn3023	pOP-EAP00627_EST_C_1_pBSK_SK	425
cl2502	ct2746	cn3023	pOP-EAP02176_EST_C_1_pBSK_SK	308
cl2502	ct2746	cn3023	pOP-EAP02851_EST_C_1_pBSK_SK	360
cl2502	ct2746	cn3023	pOP-EO02285_EST_C_1_pSK_SK	253
cl2502	ct2746	cn3023	pOP-EO05950_EST_C_1_pSK_SK	473
cl2502	ct2746	cn3023	pOP-EO07653_EST_C_1_pSK_SK	511
cl2502	ct2746	cn3023	pOP-EO08365_EST_C_1_pSK_SK	318
cl2503	ct2747	cn3024	pOP-EO04852_EST_C_1_pSK_SK	507
cl2503	ct2747	cn3024	pOP-EO07659_EST_C_1_pSK_SK	642
cl2503	ct2748	cn3025	pOP-CEO03731_EST_C_1_pSK_SK	467
cl2503	ct2748	cn3025	pOP-EAP02275_EST_C_1_pBSK_SK	407
cl2503	ct2748	cn3025	pOP-EO02450_EST_C_1_pSK_SK	418
cl2503	ct2748	cn3025	pOP-EO05897_EST_C_1_pSK_SK	660
cl2503	ct2748	cn3025	pOP-EO07988_EST_C_1_pSK_SK	321
cl2504	ct2749	cn3026	pOP-CNH01342_EST_C_1_pSK_SK	466
cl2504	ct2749	cn3026	pOP-EO03673_EST_C_1_pSK_SK	124
cl2504	ct2749	cn3026	pOP-EO03899_EST_C_1_pSK_SK	500
cl2504	ct2749	cn3026	pOP-EO04366_EST_C_1_pSK_SK	402
cl2504	ct2749	cn3026	pOP-EO06676_EST_C_1_pSK_SK	382
cl2504	ct2749	cn3026	pOP-EO06747_EST_C_1_pSK_SK	379
cl2504	ct2749	cn3026	pOP-EO06927_EST_C_1_pSK_SK	709
cl2504	ct2749	cn3026	pOP-EO07662_EST_C_1_pSK_SK	792
cl2504	ct2749	cn3026	pOP-EO07663_EST_C_1_pSK_SK	331
cl2504	ct2749	cn3026	pOP-EO07852_EST_C_1_pSK_SK	819
cl2505	ct2750	cn3027	pOP-CEO02976_EST_C_1_pSK_SK	128
cl2505	ct2750	cn3027	pOP-EO07672_EST_C_1_pSK_SK	320

cl2506	ct2751	cn3028	pOP-CAP00041_EST_C_1_pBSK_SK	557
cl2506	ct2751	cn3028	pOP-EAP03610_EST_C_1_pBSK_SK	350
cl2506	ct2751	cn3028	pOP-EO06875_EST_C_1_pSK_SK	808
cl2506	ct2751	cn3028	pOP-EO07258_EST_C_1_pSK_SK	808
cl2506	ct2751	cn3028	pOP-EO07281_EST_C_1_pSK_SK	790
cl2506	ct2751	cn3028	pOP-EO07675_EST_C_1_pSK_SK	793
cl2507	ct2752	cn3029	pOP-EO04810_EST_C_1_pSK_SK	570
cl2507	ct2752	cn3029	pOP-EO07076_EST_C_1_pSK_SK	616
cl2507	ct2752	cn3029	pOP-EO07691_EST_C_1_pSK_SK	673
cl2508	ct2753	cn3030	pOP-CEO02660_EST_C_1_pSK_SK	493
cl2508	ct2753	cn3030	pOP-EO07693_EST_C_1_pSK_SK	718
cl2509	ct2754	cn3031	pOP-EO02338_EST_C_1_pSK_SK	589
cl2509	ct2754	cn3031	pOP-EO06494_EST_C_1_pSK_SK	738
cl2509	ct2754	cn3031	pOP-EO07696_EST_C_1_pSK_SK	657
cl2510	ct2755	cn3032	pOP-EO07683_EST_C_1_pSK_SK	698
cl2510	ct2756	cn3033	pOP-EO07721_EST_C_1_pSK_SK	562
cl2511	ct2757	cn3034	pOP-CAP00012_EST_C_1_pBSK_SK	596
cl2511	ct2757	cn3034	pOP-CBP00110_EST_C_1_pBSK_SK	419
cl2511	ct2757	cn3034	pOP-EN00394_EST_C_1_pSK_SK	455
cl2511	ct2757	cn3034	pOP-EO04036_EST_C_1_pSK_SK	404
cl2511	ct2757	cn3034	pOP-EO04235_EST_C_1_pSK_SK	500
cl2511	ct2757	cn3034	pOP-EO07508_EST_C_1_pSK_SK	685
cl2511	ct2757	cn3034	pOP-EO07726_EST_C_1_pSK_SK	685
cl2512	ct2758	cn3035	pOP-CNI01268_EST_C_1_pSK_SK	525
cl2512	ct2758	cn3035	pOP-EO07741_EST_C_1_pSK_SK	646
cl2513	ct2759	cn3036	pOP-EO05892_EST_C_1_pSK_SK	490
cl2513	ct2759	cn3036	pOP-EO07615_EST_C_1_pSK_SK	671
cl2513	ct2759	cn3036	pOP-EO07743_EST_C_1_pSK_SK	773
cl2513	ct2759	cn3037	pOP-EO06290_EST_C_1_pSK_SK	680
cl2514	ct2760	cn3038	pOP-CNH01953_EST_C_1_pSK_SK	512
cl2514	ct2760	cn3038	pOP-CNH02022_EST_C_1_pSK_SK	547
cl2514	ct2760	cn3038	pOP-CNH02384_EST_C_1_pSK_SK	535
cl2514	ct2760	cn3038	pOP-CNH03433_EST_C_1_pSK_SK	511
cl2514	ct2760	cn3038	pOP-CNH04546	662
cl2514	ct2760	cn3038	pOP-CNH04691	666
cl2514	ct2760	cn3038	pOP-CNH04965_EST_C_1_pSK_SK	669
cl2514	ct2760	cn3038	pOP-EO07717_EST_C_1_pSK_SK	342
cl2514	ct2760	cn3038	pOP-EO07747_EST_C_1_pSK_SK	682
cl2515	ct2761	cn3039	pOP-EN00228_EST_C_1_pSK_SK	446
cl2515	ct2761	cn3039	pOP-EN00843_EST_C_1_pSK_SK	514
cl2515	ct2761	cn3039	pOP-EO03855_EST_C_1_pSK_SK	518
cl2515	ct2761	cn3039	pOP-EO07751_EST_C_1_pSK_SK	758
cl2516	ct2762	cn3040	pOP-CNH00671_EST_C_1_pSK_SK	580
cl2516	ct2762	cn3040	pOP-CNH01406_EST_C_1_pSK_SK	678
cl2516	ct2762	cn3040	pOP-CNHP00407_EST_C_1_pSK_SK	636
cl2516	ct2762	cn3040	pOP-EO05125_EST_C_1_pSK_SK	495
cl2516	ct2762	cn3040	pOP-EO07759_EST_C_1_pSK_SK	748
cl2517	ct2763	cn3041	pOP-EO03141_EST_C_1_pSK_SK	455
cl2517	ct2763	cn3041	pOP-EO07766_EST_C_1_pSK_SK	284
cl2518	ct2764	cn3042	pOP-CNH00975_EST_C_1_pSK_SK	613
cl2518	ct2764	cn3042	pOP-EO07769_EST_C_1_pSK_SK	817
cl2519	ct2765	cn3043	pOP-EO02255_EST_C_1_pSK_SK	727
cl2519	ct2765	cn3043	pOP-EO04088_EST_C_1_pSK_SK	478
cl2519	ct2765	cn3043	pOP-EO07772_EST_C_1_pSK_SK	822
cl2520	ct2766	cn3044	pOP-EO04420_EST_C_1_pSK_SK	526
cl2520	ct2766	cn3044	pOP-EO04421_EST_C_1_pSK_SK	485

cl2520	ct2766	cn3044	pOP-EO07777_EST_C_1_pSK_SK	750
cl2521	ct2767	cn3045	pOP-CNH01973_EST_C_1_pSK_SK	580
cl2521	ct2767	cn3045	pOP-EO07801_EST_C_1_pSK_SK	559
cl2522	ct2768	cn3046	pOP-CNH03137_EST_C_1_pSK_SK	616
cl2522	ct2768	cn3046	pOP-EO02600_EST_C_1_pSK_SK	374
cl2522	ct2768	cn3046	pOP-EO07805_EST_C_1_pSK_SK	826
cl2523	ct2769	cn3047	pOP-EO03500_EST_C_1_pSK_SK	538
cl2523	ct2769	cn3047	pOP-EO07807_EST_C_1_pSK_SK	680
cl2524	ct2770	cn3048	pOP-EO06180_EST_C_1_pSK_SK	632
cl2524	ct2770	cn3048	pOP-EO07808_EST_C_1_pSK_SK	796
cl2525	ct2771	cn3049	pOP-CNH01620_EST_C_1_pSK_SK	540
cl2525	ct2771	cn3049	pOP-EO07815_EST_C_1_pSK_SK	676
cl2526	ct2772	cn3050	pOP-EN00861_EST_C_1_pSK_SK	541
cl2526	ct2772	cn3050	pOP-EN00891_EST_C_1_pSK_SK	532
cl2526	ct2772	cn3050	pOP-EO02812_EST_C_1_pSK_SK	342
cl2526	ct2772	cn3050	pOP-EO07817_EST_C_1_pSK_SK	210
cl2527	ct2773	cn3051	pOP-EO07317_EST_C_1_pSK_SK	445
cl2527	ct2773	cn3051	pOP-EO07822_EST_C_1_pSK_SK	445
cl2528	ct2774	cn3052	pOP-EO06577_EST_C_1_pSK_SK	849
cl2528	ct2774	cn3052	pOP-EO08252_EST_C_1_pSK_SK	424
cl2528	ct2774	cn3053	pOP-EO07837_EST_C_1_pSK_SK	830
cl2529	ct2775	cn3054	pOP-CBP00150_EST_C_1_pBSK_SK	327
cl2529	ct2775	cn3054	pOP-CBP00154_EST_C_1_pBSK_SK	363
cl2529	ct2775	cn3054	pOP-CBP00168_EST_C_1_pBSK_SK	323
cl2529	ct2775	cn3054	pOP-EO07839_EST_C_1_pSK_SK	337
cl2530	ct2776	cn3055	pOP-EO06165_EST_C_1_pSK_SK	288
cl2530	ct2776	cn3055	pOP-EO07844_EST_C_1_pSK_SK	739
cl2531	ct2777	cn3056	pOP-CEO00600_EST_C_1_pSK_SK	317
cl2531	ct2777	cn3056	pOP-EAP00611_EST_C_1_pBSK_SK	178
cl2531	ct2777	cn3056	pOP-EAP01546_EST_C_1_pBSK_SK	490
cl2531	ct2777	cn3056	pOP-EAP02122_EST_C_1_pBSK_SK	238
cl2531	ct2777	cn3056	pOP-EO06650_EST_C_1_pSK_SK	751
cl2531	ct2777	cn3056	pOP-EO06773_EST_C_1_pSK_SK	849
cl2531	ct2777	cn3056	pOP-EO07848_EST_C_1_pSK_SK	487
cl2532	ct2778	cn3057	pOP-CEO03730_EST_C_1_pSK_SK	400
cl2532	ct2779	cn3058	pOP-CNIP00975_EST_C_1_pSK_SK	545
cl2532	ct2779	cn3058	pOP-EO02077_EST_C_1_pSK_SK	233
cl2532	ct2780	cn3059	pOP-EO07850_EST_C_1_pSK_SK	721
cl2532	ct2780	cn3060	pOP-CEM00045_EST_C_1_pSK_SK	291
cl2532	ct2780	cn3060	pOP-CEO03763_EST_C_1_pSK_SK	283
cl2532	ct2780	cn3060	pOP-EAP00793_EST_C_1_pBSK_SK	448
cl2532	ct2780	cn3060	pOP-EO02103_EST_C_1_pSK_SK	474
cl2532	ct2780	cn3060	pOP-EO05718_EST_C_1_pSK_SK	514
cl2533	ct2781	cn3061	pOP-EN00237_EST_C_1_pSK_SK	434
cl2533	ct2781	cn3061	pOP-EO07264_EST_C_1_pSK_SK	847
cl2533	ct2781	cn3062	pOP-EO07859_EST_C_1_pSK_SK	765
cl2534	ct2782	cn3063	pOP-CNH02344_EST_C_1_pSK_SK	648
cl2534	ct2782	cn3063	pOP-EO07867_EST_C_1_pSK_SK	560
cl2535	ct2783	cn3064	pOP-EO04258_EST_C_1_pSK_SK	376
cl2535	ct2783	cn3064	pOP-EO06998_EST_C_1_pSK_SK	773
cl2535	ct2783	cn3064	pOP-EO07136_EST_C_1_pSK_SK	656
cl2535	ct2783	cn3064	pOP-EO07868_EST_C_1_pSK_SK	579
cl2536	ct2784	cn3065	pOP-EO06137_EST_C_1_pSK_SK	400
cl2536	ct2784	cn3065	pOP-EO07871_EST_C_1_pSK_SK	726
cl2537	ct2785	cn3066	pOP-EO07762_EST_C_1_pSK_SK	793
cl2537	ct2785	cn3066	pOP-EO07872_EST_C_1_pSK_SK	602

cl2538	ct2786	cn3067	pOP-CEO03206_EST_C_1_pSK_SK	702
cl2538	ct2787	cn3068	pOP-CNH01143_EST_C_1_pSK_SK	178
cl2538	ct2787	cn3068	pOP-EO04433_EST_C_1_pSK_SK	526
cl2538	ct2788	cn3069	pOP-CAP00366_EST_C_1_pBSK_SK	440
cl2538	ct2788	cn3069	pOP-CBP00182_EST_C_1_pBSK_SK	417
cl2538	ct2788	cn3069	pOP-CBP00216_EST_C_1_pBSK_SK	359
cl2538	ct2788	cn3069	pOP-CBP00232_EST_C_1_pBSK_SK	484
cl2538	ct2788	cn3069	pOP-CBP00260_EST_C_1_pBSK_SK	233
cl2538	ct2788	cn3069	pOP-CEO00704_EST_C_1_pSK_SK	518
cl2538	ct2788	cn3069	pOP-CEO00964_EST_C_1_pSK_SK	185
cl2538	ct2788	cn3069	pOP-CEO01329	490
cl2538	ct2788	cn3069	pOP-CEO01705_EST_C_1_pSK_SK	531
cl2538	ct2788	cn3069	pOP-CEO02424_EST_C_1_pSK_SK	179
cl2538	ct2788	cn3069	pOP-CEO02464_EST_C_1_pSK_SK	405
cl2538	ct2788	cn3069	pOP-CEO02718_EST_C_1_pSK_SK	338
cl2538	ct2788	cn3069	pOP-CEO02989_EST_C_1_pSK_SK	475
cl2538	ct2788	cn3069	pOP-CEO03424_EST_C_1_pSK_SK	570
cl2538	ct2788	cn3069	pOP-CEO03565_EST_C_1_pSK_SK	481
cl2538	ct2788	cn3069	pOP-CEO03573_EST_C_1_pSK_SK	384
cl2538	ct2788	cn3069	pOP-CEO03613_EST_C_1_pSK_SK	376
cl2538	ct2788	cn3069	pOP-CEO03690_EST_C_1_pSK_SK	334
cl2538	ct2788	cn3069	pOP-CEOP00019_EST_C_1_pSK_SK	446
cl2538	ct2788	cn3069	pOP-CNH00565_EST_C_1_pSK_SK	489
cl2538	ct2788	cn3069	pOP-CNH00595_EST_C_1_pSK_SK	528
cl2538	ct2788	cn3069	pOP-CNH00604_EST_C_1_pSK_SK	438
cl2538	ct2788	cn3069	pOP-CNH00746_EST_C_1_pSK_SK	398
cl2538	ct2788	cn3069	pOP-CNH00822_EST_C_1_pSK_SK	488
cl2538	ct2788	cn3069	pOP-CNH00832_EST_C_1_pSK_SK	398
cl2538	ct2788	cn3069	pOP-CNH01119_EST_C_1_pSK_SK	241
cl2538	ct2788	cn3069	pOP-CNH01451_EST_C_1_pSK_SK	485
cl2538	ct2788	cn3069	pOP-CNH01493_EST_C_1_pSK_SK	470
cl2538	ct2788	cn3069	pOP-CNH01980_EST_C_1_pSK_SK	488
cl2538	ct2788	cn3069	pOP-CNH02291_EST_C_1_pSK_SK	491
cl2538	ct2788	cn3069	pOP-CNH03094_EST_C_1_pSK_SK	474
cl2538	ct2788	cn3069	pOP-CNH03691_EST_C_1_pSK_SK	369
cl2538	ct2788	cn3069	pOP-CNH03694_EST_C_1_pSK_SK	471
cl2538	ct2788	cn3069	pOP-CNH04281	471
cl2538	ct2788	cn3069	pOP-CNH04330	430
cl2538	ct2788	cn3069	pOP-CNH05062_EST_C_1_pSK_SK	487
cl2538	ct2788	cn3069	pOP-CNI01116_EST_C_1_pSK_SK	494
cl2538	ct2788	cn3069	pOP-CNI01179_EST_C_1_pSK_SK	489
cl2538	ct2788	cn3069	pOP-CNI01240_EST_C_1_pSK_SK	382
cl2538	ct2788	cn3069	pOP-CNI01279_EST_C_1_pSK_SK	427
cl2538	ct2788	cn3069	pOP-CNI01280_EST_C_1_pSK_SK	473
cl2538	ct2788	cn3069	pOP-CNI01362_EST_C_1_pSK_SK	441
cl2538	ct2788	cn3069	pOP-CNI01365_EST_C_1_pSK_SK	511
cl2538	ct2788	cn3069	pOP-CNI01386_EST_C_1_pSK_SK	490
cl2538	ct2788	cn3069	pOP-CNI01391_EST_C_1_pSK_SK	479
cl2538	ct2788	cn3069	pOP-CNI01415_EST_C_1_pSK_SK	510
cl2538	ct2788	cn3069	pOP-CNI01431_EST_C_1_pSK_SK	488
cl2538	ct2788	cn3069	pOP-CNI01435_EST_C_1_pSK_SK	213
cl2538	ct2788	cn3069	pOP-CNI01456_EST_C_1_pSK_SK	464
cl2538	ct2788	cn3069	pOP-CNI01461_EST_C_1_pSK_SK	481
cl2538	ct2788	cn3069	pOP-CNI01469_EST_C_1_pSK_SK	487
cl2538	ct2788	cn3069	pOP-CNI01477_EST_C_1_pSK_SK	467
cl2538	ct2788	cn3069	pOP-CNI01481_EST_C_1_pSK_SK	488

cl2538	ct2788	cn3069	pOP-CNI01496_EST_C_1_pSK_SK	512
cl2538	ct2788	cn3069	pOP-CNI01531_EST_C_1_pSK_SK	356
cl2538	ct2788	cn3069	pOP-CNI01533_EST_C_1_pSK_SK	260
cl2538	ct2788	cn3069	pOP-CNI01600_EST_C_1_pSK_SK	385
cl2538	ct2788	cn3069	pOP-CNI01640_EST_C_1_pSK_SK	484
cl2538	ct2788	cn3069	pOP-CNI01676_EST_C_1_pSK_SK	367
cl2538	ct2788	cn3069	pOP-CNI01678_EST_C_1_pSK_SK	501
cl2538	ct2788	cn3069	pOP-CNI01697_EST_C_1_pSK_SK	359
cl2538	ct2788	cn3069	pOP-CNI01707_EST_C_1_pSK_SK	467
cl2538	ct2788	cn3069	pOP-CNI01728_EST_C_1_pSK_SK	492
cl2538	ct2788	cn3069	pOP-CNI01738_EST_C_1_pSK_SK	119
cl2538	ct2788	cn3069	pOP-CNI01768_EST_C_1_pSK_SK	462
cl2538	ct2788	cn3069	pOP-CNI01871_EST_C_1_pSK_SK	496
cl2538	ct2788	cn3069	pOP-CNI01898_EST_C_1_pSK_SK	485
cl2538	ct2788	cn3069	pOP-CNI01902_EST_C_1_pSK_SK	256
cl2538	ct2788	cn3069	pOP-CNI01914_EST_C_1_pSK_SK	501
cl2538	ct2788	cn3069	pOP-CNI01929_EST_C_1_pSK_SK	475
cl2538	ct2788	cn3069	pOP-CNI01941_EST_C_1_pSK_SK	472
cl2538	ct2788	cn3069	pOP-CNI01945_EST_C_1_pSK_SK	453
cl2538	ct2788	cn3069	pOP-CNI02030_EST_C_1_pSK_SK	502
cl2538	ct2788	cn3069	pOP-CNI02113_EST_C_1_pSK_SK	484
cl2538	ct2788	cn3069	pOP-CNI02125_EST_C_1_pSK_SK	491
cl2538	ct2788	cn3069	pOP-CNI02227_EST_C_1_pSK_SK	468
cl2538	ct2788	cn3069	pOP-CNI02228_EST_C_1_pSK_SK	470
cl2538	ct2788	cn3069	pOP-CNIP00034_EST_C_1_pSK_SK	354
cl2538	ct2788	cn3069	pOP-CNIP00189_EST_C_1_pSK_SK	460
cl2538	ct2788	cn3069	pOP-CNIP00292_EST_C_1_pSK_SK	269
cl2538	ct2788	cn3069	pOP-CNIP00337_EST_C_1_pSK_SK	440
cl2538	ct2788	cn3069	pOP-CNIP00431_EST_C_1_pSK_SK	367
cl2538	ct2788	cn3069	pOP-CNIP00608_EST_C_1_pSK_SK	397
cl2538	ct2788	cn3069	pOP-CNIP00624_EST_C_1_pSK_SK	415
cl2538	ct2788	cn3069	pOP-CNIP00672_EST_C_1_pSK_SK	504
cl2538	ct2788	cn3069	pOP-CNIP00682_EST_C_1_pSK_SK	442
cl2538	ct2788	cn3069	pOP-CNIP00691_EST_C_1_pSK_SK	488
cl2538	ct2788	cn3069	pOP-CNIP00790_EST_C_1_pSK_SK	385
cl2538	ct2788	cn3069	pOP-CNIP00848_EST_C_1_pSK_SK	480
cl2538	ct2788	cn3069	pOP-CNIP00861_EST_C_1_pSK_SK	481
cl2538	ct2788	cn3069	pOP-CNIP00949_EST_C_1_pSK_SK	516
cl2538	ct2788	cn3069	pOP-CNIP01000_EST_C_1_pSK_SK	454
cl2538	ct2788	cn3069	pOP-CNIP01003_EST_C_1_pSK_SK	498
cl2538	ct2788	cn3069	pOP-CNIP01046_EST_C_1_pSK_SK	483
cl2538	ct2788	cn3069	pOP-CNIP01060_EST_C_1_pSK_SK	482
cl2538	ct2788	cn3069	pOP-CNIP04036_EST_C_1_pSK_SK	486
cl2538	ct2788	cn3069	pOP-CNIP04044_EST_C_1_pSK_SK	494
cl2538	ct2788	cn3069	pOP-EAP00222_EST_C_1_pBSK_SK	347
cl2538	ct2788	cn3069	pOP-EAP00223_EST_C_1_pBSK_SK	232
cl2538	ct2788	cn3069	pOP-EAP00291_EST_C_1_pBSK_SK	389
cl2538	ct2788	cn3069	pOP-EAP00828_EST_C_1_pBSK_SK	520
cl2538	ct2788	cn3069	pOP-EAP01112_EST_C_1_pBSK_SK	278
cl2538	ct2788	cn3069	pOP-EAP01189_EST_C_1_pBSK_SK	367
cl2538	ct2788	cn3069	pOP-EAP01717_EST_C_1_pBSK_SK	449
cl2538	ct2788	cn3069	pOP-EAP01718_EST_C_1_pBSK_SK	462
cl2538	ct2788	cn3069	pOP-EAP01736_EST_C_1_pBSK_SK	420
cl2538	ct2788	cn3069	pOP-EAP02059_EST_C_1_pBSK_SK	311
cl2538	ct2788	cn3069	pOP-EAP03748_EST_C_1_pBSK_SK	422
cl2538	ct2788	cn3069	pOP-EAP05015_EST_C_1_pBSK_SK	469

cl2538	ct2788	cn3069	pOP-EO02304_EST_C_1_pSK_SK	467
cl2538	ct2788	cn3069	pOP-EO02633_EST_C_1_pSK_SK	333
cl2538	ct2788	cn3069	pOP-EO03621_EST_C_1_pSK_SK	450
cl2538	ct2788	cn3069	pOP-EO05107_EST_C_1_pSK_SK	477
cl2538	ct2788	cn3069	pOP-EO06705_EST_C_1_pSK_SK	483
cl2538	ct2788	cn3069	pOP-EO06718_EST_C_1_pSK_SK	477
cl2538	ct2788	cn3069	pOP-EO07022_EST_C_1_pSK_SK	468
cl2538	ct2788	cn3069	pOP-EO07888_EST_C_1_pSK_SK	435
cl2538	ct2788	cn3070	pOP-CEO03337_EST_C_1_pSK_SK	250
cl2538	ct2788	cn3070	pOP-CNI02118_EST_C_1_pSK_SK	515
cl2538	ct2788	cn3070	pOP-EO03686_EST_C_1_pSK_SK	305
cl2538	ct2788	cn3071	pOP-CNH02874_EST_C_1_pSK_SK	365
cl2539	ct2789	cn3072	pOP-EN00300_EST_C_1_pSK_SK	537
cl2539	ct2790	cn3073	pOP-CNH03502_EST_C_1_pSK_SK	602
cl2539	ct2790	cn3073	pOP-EN00153_EST_C_1_pSK_SK	441
cl2539	ct2791	cn3074	pOP-CEO01918_EST_C_1_pSK_SK	369
cl2539	ct2791	cn3074	pOP-CEO02468_EST_C_1_pSK_SK	450
cl2539	ct2791	cn3074	pOP-CEOP00031_EST_C_1_pSK_SK	436
cl2539	ct2791	cn3074	pOP-CNH00768_EST_C_1_pSK_SK	462
cl2539	ct2791	cn3074	pOP-CNH03126_EST_C_1_pSK_SK	471
cl2539	ct2791	cn3074	pOP-CNH04340	809
cl2539	ct2791	cn3074	pOP-CNI02064_EST_C_1_pSK_SK	421
cl2539	ct2791	cn3074	pOP-CNI02153_EST_C_1_pSK_SK	178
cl2539	ct2791	cn3074	pOP-EAP01949_EST_C_1_pBSK_SK	421
cl2539	ct2791	cn3074	pOP-EN00437_EST_C_1_pSK_SK	449
cl2539	ct2791	cn3074	pOP-EO03217_EST_C_1_pSK_SK	688
cl2539	ct2791	cn3074	pOP-EO05347_EST_C_1_pSK_SK	550
cl2539	ct2791	cn3074	pOP-EO06075_EST_C_1_pSK_SK	526
cl2539	ct2791	cn3074	pOP-EO07891_EST_C_1_pSK_SK	632
cl2540	ct2792	cn3075	pOP-EO03174_EST_C_1_pSK_SK	418
cl2540	ct2792	cn3075	pOP-EO03742_EST_C_1_pSK_SK	497
cl2540	ct2792	cn3075	pOP-EO05962_EST_C_1_pSK_SK	566
cl2540	ct2792	cn3075	pOP-EO07896_EST_C_1_pSK_SK	588
cl2541	ct2793	cn3076	pOP-EO02928_EST_C_1_pSK_SK	397
cl2541	ct2793	cn3076	pOP-EO05537_EST_C_1_pSK_SK	428
cl2541	ct2793	cn3076	pOP-EO05546_EST_C_1_pSK_SK	421
cl2541	ct2793	cn3076	pOP-EO06913_EST_C_1_pSK_SK	650
cl2541	ct2793	cn3076	pOP-EO07898_EST_C_1_pSK_SK	355
cl2542	ct2794	cn3077	pOP-CNH01519_EST_C_1_pSK_SK	569
cl2542	ct2794	cn3077	pOP-CNH02929_EST_C_1_pSK_SK	629
cl2542	ct2794	cn3077	pOP-CNH04445	761
cl2542	ct2794	cn3077	pOP-CNI01440_EST_C_1_pSK_SK	674
cl2542	ct2795	cn3078	pOP-CNH02098_EST_C_1_pSK_SK	446
cl2542	ct2795	cn3078	pOP-CNH02465_EST_C_1_pSK_SK	420
cl2542	ct2795	cn3078	pOP-EO06847_EST_C_1_pSK_SK	705
cl2542	ct2795	cn3078	pOP-EO07907_EST_C_1_pSK_SK	750
cl2543	ct2796	cn3079	pOP-CNH01238_EST_C_1_pSK_SK	665
cl2543	ct2796	cn3079	pOP-EO07913_EST_C_1_pSK_SK	368
cl2544	ct2797	cn3080	pOP-EO05749_EST_C_1_pSK_SK	567
cl2544	ct2797	cn3080	pOP-EO07916_EST_C_1_pSK_SK	682
cl2545	ct2798	cn3081	pOP-CNH02027_EST_C_1_pSK_SK	571
cl2545	ct2798	cn3081	pOP-EO05498_EST_C_1_pSK_SK	515
cl2545	ct2798	cn3081	pOP-EO07918_EST_C_1_pSK_SK	702
cl2546	ct2799	cn3082	pOP-EO04759_EST_C_1_pSK_SK	518
cl2546	ct2799	cn3082	pOP-EO07923_EST_C_1_pSK_SK	713
cl2547	ct2800	cn3083	pOP-EO07906_EST_C_1_pSK_SK	506

cl2547	ct2800	cn3083	pOP-EO07925_EST_C_1_pSK_SK	508
cl2548	ct2801	cn3084	pOP-EO02572_EST_C_1_pSK_SK	475
cl2548	ct2801	cn3084	pOP-EO07926_EST_C_1_pSK_SK	642
cl2549	ct2802	cn3085	pOP-CNH04908_EST_C_1_pSK_SK	494
cl2549	ct2802	cn3085	pOP-EO02088_EST_C_1_pSK_SK	532
cl2549	ct2802	cn3085	pOP-EO07467_EST_C_1_pSK_SK	581
cl2549	ct2802	cn3085	pOP-EO07932_EST_C_1_pSK_SK	143
cl2549	ct2802	cn3086	pOP-EAP02756_EST_C_1_pBSK_SK	636
cl2550	ct2803	cn3087	pOP-EO06239_EST_C_1_pSK_SK	681
cl2550	ct2803	cn3087	pOP-EO07933_EST_C_1_pSK_SK	264
cl2551	ct2804	cn3088	pOP-EO04327_EST_C_1_pSK_SK	515
cl2551	ct2804	cn3088	pOP-EO07944_EST_C_1_pSK_SK	694
cl2552	ct2805	cn3089	pOP-CBP00097_EST_C_1_pBSK_SK	458
cl2552	ct2805	cn3089	pOP-CEMP00001_EST_C_1_pSK_SK	265
cl2552	ct2805	cn3089	pOP-CEO00773_EST_C_1_pSK_SK	204
cl2552	ct2805	cn3089	pOP-CEO01622_EST_C_1_pSK_SK	413
cl2552	ct2805	cn3089	pOP-CEO02405_EST_C_1_pSK_SK	381
cl2552	ct2805	cn3089	pOP-CNI01261_EST_C_1_pSK_SK	469
cl2552	ct2805	cn3089	pOP-CNI02006_EST_C_1_pSK_SK	243
cl2552	ct2805	cn3089	pOP-CNI02105_EST_C_1_pSK_SK	255
cl2552	ct2805	cn3089	pOP-CNIP00310_EST_C_1_pSK_SK	292
cl2552	ct2805	cn3089	pOP-EO07953_EST_C_1_pSK_SK	688
cl2553	ct2806	cn3090	pOP-EO07072_EST_C_1_pSK_SK	594
cl2553	ct2806	cn3090	pOP-EO07954_EST_C_1_pSK_SK	736
cl2554	ct2807	cn3091	pOP-EO03753_EST_C_1_pSK_SK	473
cl2554	ct2807	cn3091	pOP-EO07956_EST_C_1_pSK_SK	719
cl2555	ct2808	cn3092	pOP-CEO02496_EST_C_1_pSK_SK	505
cl2555	ct2808	cn3092	pOP-EO02390_EST_C_1_pSK_SK	564
cl2555	ct2808	cn3092	pOP-EO04063_EST_C_1_pSK_SK	555
cl2555	ct2808	cn3092	pOP-EO07870_EST_C_1_pSK_SK	686
cl2555	ct2808	cn3092	pOP-EO07973_EST_C_1_pSK_SK	232
cl2556	ct2809	cn3093	pOP-CAP00262_EST_C_1_pBSK_SK	694
cl2556	ct2809	cn3093	pOP-EO07979_EST_C_1_pSK_SK	427
cl2557	ct2810	cn3094	pOP-EO02073_EST_C_1_pSK_SK	338
cl2557	ct2810	cn3094	pOP-EO07993_EST_C_1_pSK_SK	425
cl2558	ct2811	cn3095	pOP-EO08004_EST_C_1_pSK_SK	435
cl2558	ct2812	cn3096	pOP-CNH00909_EST_C_1_pSK_SK	490
cl2559	ct2813	cn3097	pOP-EO02617_EST_C_1_pSK_SK	373
cl2559	ct2813	cn3097	pOP-EO07262_EST_C_1_pSK_SK	741
cl2559	ct2813	cn3097	pOP-EO08013_EST_C_1_pSK_SK	324
cl2560	ct2814	cn3098	pOP-EN00247_EST_C_1_pSK_SK	522
cl2560	ct2814	cn3098	pOP-EO06081_EST_C_1_pSK_SK	693
cl2560	ct2815	cn3099	pOP-EN00691_EST_C_1_pSK_SK	529
cl2560	ct2815	cn3099	pOP-EO05930_EST_C_1_pSK_SK	579
cl2560	ct2815	cn3099	pOP-EO08015_EST_C_1_pSK_SK	288
cl2561	ct2816	cn3100	pOP-CNH01003_EST_C_1_pSK_SK	247
cl2561	ct2816	cn3100	pOP-CNH01030_EST_C_1_pSK_SK	432
cl2561	ct2817	cn3101	pOP-EAP02726_EST_C_1_pBSK_SK	516
cl2561	ct2817	cn3101	pOP-EO08022_EST_C_1_pSK_SK	334
cl2562	ct2818	cn3102	pOP-CNIP00755_EST_C_1_pSK_SK	631
cl2562	ct2819	cn3103	pOP-EO06130_EST_C_1_pSK_SK	629
cl2562	ct2819	cn3103	pOP-EO08026_EST_C_1_pSK_SK	376
cl2562	ct2819	cn3104	pOP-CNH03207_EST_C_1_pSK_SK	527
cl2562	ct2819	cn3104	pOP-CNI01424_EST_C_1_pSK_SK	486
cl2563	ct2820	cn3105	pOP-CNHP00190_EST_C_1_pSK_SK	651
cl2563	ct2820	cn3105	pOP-EO08028_EST_C_1_pSK_SK	391

cl2564	ct2821	cn3106	pOP-EN00481_EST_C_1_pSK_SK	527
cl2564	ct2821	cn3106	pOP-EO05810_EST_C_1_pSK_SK	605
cl2564	ct2821	cn3106	pOP-EO08032_EST_C_1_pSK_SK	475
cl2565	ct2822	cn3107	pOP-EAP01298_EST_C_1_pBSK_SK	319
cl2565	ct2822	cn3107	pOP-EO04570_EST_C_1_pSK_SK	523
cl2565	ct2822	cn3107	pOP-EO05223_EST_C_1_pSK_SK	523
cl2565	ct2822	cn3107	pOP-EO05261_EST_C_1_pSK_SK	519
cl2565	ct2822	cn3107	pOP-EO06107_EST_C_1_pSK_SK	597
cl2565	ct2822	cn3107	pOP-EO07207_EST_C_1_pSK_SK	721
cl2565	ct2822	cn3107	pOP-EO08041_EST_C_1_pSK_SK	418
cl2566	ct2823	cn3108	pOP-EO02974_EST_C_1_pSK_SK	347
cl2566	ct2823	cn3108	pOP-EO08042_EST_C_1_pSK_SK	436
cl2567	ct2824	cn3109	pOP-CNH01237_EST_C_1_pSK_SK	658
cl2567	ct2824	cn3109	pOP-CNH01910_EST_C_1_pSK_SK	665
cl2567	ct2824	cn3109	pOP-EN00348_EST_C_1_pSK_SK	535
cl2567	ct2824	cn3109	pOP-EO04118_EST_C_1_pSK_SK	336
cl2567	ct2824	cn3109	pOP-EO08045_EST_C_1_pSK_SK	370
cl2568	ct2825	cn3110	pOP-CNH00890_EST_C_1_pSK_SK	596
cl2568	ct2825	cn3110	pOP-CNH03024_EST_C_1_pSK_SK	514
cl2568	ct2825	cn3110	pOP-CNH03395_EST_C_1_pSK_SK	530
cl2568	ct2825	cn3110	pOP-CNH03470_EST_C_1_pSK_SK	507
cl2568	ct2825	cn3110	pOP-CNH04940_EST_C_1_pSK_SK	699
cl2568	ct2825	cn3110	pOP-CNHP00231_EST_C_1_pSK_SK	389
cl2568	ct2825	cn3110	pOP-CNHP00274_EST_C_1_pSK_SK	356
cl2568	ct2825	cn3110	pOP-CNI01211_EST_C_1_pSK_SK	284
cl2568	ct2825	cn3110	pOP-CNIP00729_EST_C_1_pSK_SK	317
cl2568	ct2825	cn3110	pOP-EAP01676_EST_C_1_pBSK_SK	603
cl2568	ct2825	cn3110	pOP-EO05709_EST_C_1_pSK_SK	428
cl2568	ct2825	cn3110	pOP-EO08047_EST_C_1_pSK_SK	363
cl2568	ct2825	cn3111	pOP-CNI02244_EST_C_1_pSK_SK	640
cl2569	ct2826	cn3112	pOP-EN00838_EST_C_1_pSK_SK	502
cl2569	ct2826	cn3112	pOP-EO05959_EST_C_1_pSK_SK	627
cl2569	ct2826	cn3112	pOP-EO06575_EST_C_1_pSK_SK	848
cl2569	ct2826	cn3112	pOP-EO08051_EST_C_1_pSK_SK	353
cl2570	ct2827	cn3113	pOP-EO04069_EST_C_1_pSK_SK	554
cl2570	ct2827	cn3113	pOP-EO04205_EST_C_1_pSK_SK	552
cl2570	ct2827	cn3113	pOP-EO06288_EST_C_1_pSK_SK	686
cl2570	ct2827	cn3113	pOP-EO08059_EST_C_1_pSK_SK	588
cl2571	ct2828	cn3114	pOP-CAP00347_EST_C_1_pBSK_SK	488
cl2571	ct2828	cn3114	pOP-CNH03536_EST_C_1_pSK_SK	224
cl2571	ct2828	cn3114	pOP-CNH04594	677
cl2571	ct2828	cn3114	pOP-EO02231_EST_C_1_pSK_SK	525
cl2571	ct2828	cn3114	pOP-EO02940_EST_C_1_pSK_SK	272
cl2571	ct2828	cn3114	pOP-EO03826_EST_C_1_pSK_SK	443
cl2571	ct2828	cn3114	pOP-EO05486_EST_C_1_pSK_SK	408
cl2571	ct2828	cn3114	pOP-EO07788_EST_C_1_pSK_SK	428
cl2571	ct2828	cn3114	pOP-EO08061_EST_C_1_pSK_SK	551
cl2572	ct2829	cn3115	pOP-CNI01543_EST_C_1_pSK_SK	362
cl2572	ct2829	cn3115	pOP-EO07316_EST_C_1_pSK_SK	637
cl2572	ct2829	cn3115	pOP-EO08063_EST_C_1_pSK_SK	419
cl2572	ct2830	cn3116	pOP-CNHP00476_EST_C_1_pSK_SK	606
cl2572	ct2830	cn3116	pOP-CNIP00987_EST_C_1_pSK_SK	350
cl2572	ct2830	cn3116	pOP-EO05945_EST_C_1_pSK_SK	575
cl2572	ct2830	cn3116	pOP-EO06615_EST_C_1_pSK_SK	840
cl2572	ct2830	cn3116	pOP-EO07642_EST_C_1_pSK_SK	446
cl2572	ct2830	cn3116	pOP-EO07651_EST_C_1_pSK_SK	756

cl2573	ct2831	cn3117	pOP-CNH00719_EST_C_1_pSK_SK	565
cl2573	ct2831	cn3117	pOP-CNH02916_EST_C_1_pSK_SK	634
cl2573	ct2831	cn3117	pOP-CNH04499	743
cl2573	ct2831	cn3117	pOP-EN00345_EST_C_1_pSK_SK	553
cl2573	ct2831	cn3117	pOP-EO07984_EST_C_1_pSK_SK	494
cl2573	ct2831	cn3117	pOP-EO08064_EST_C_1_pSK_SK	533
cl2574	ct2832	cn3118	pOP-CAP00045_EST_C_1_pBSK_SK	399
cl2574	ct2832	cn3118	pOP-CAP00104_EST_C_1_pBSK_SK	621
cl2574	ct2832	cn3118	pOP-CAP00390_EST_C_1_pBSK_SK	626
cl2574	ct2832	cn3118	pOP-CEO02291_EST_C_1_pSK_SK	285
cl2574	ct2832	cn3118	pOP-EAP03602_EST_C_1_pBSK_SK	398
cl2574	ct2832	cn3118	pOP-EN00105_EST_C_1_pSK_SK	516
cl2574	ct2832	cn3118	pOP-EN00289_EST_C_1_pSK_SK	553
cl2574	ct2832	cn3118	pOP-EO02001_EST_C_1_pSK_SK	415
cl2574	ct2832	cn3118	pOP-EO02358_EST_C_1_pSK_SK	606
cl2574	ct2832	cn3118	pOP-EO02417_EST_C_1_pSK_SK	418
cl2574	ct2832	cn3118	pOP-EO03363_EST_C_1_pSK_SK	465
cl2574	ct2832	cn3118	pOP-EO03402_EST_C_1_pSK_SK	461
cl2574	ct2832	cn3118	pOP-EO03523_EST_C_1_pSK_SK	512
cl2574	ct2832	cn3118	pOP-EO04992_EST_C_1_pSK_SK	465
cl2574	ct2832	cn3118	pOP-EO05678_EST_C_1_pSK_SK	459
cl2574	ct2832	cn3118	pOP-EO08067_EST_C_1_pSK_SK	473
cl2574	ct2832	cn3118	pOP-EO08434_EST_C_1_pSK_SK	389
cl2575	ct2833	cn3119	pOP-CEO00771_EST_C_1_pSK_SK	369
cl2575	ct2833	cn3119	pOP-CEO03482_EST_C_1_pSK_SK	320
cl2575	ct2833	cn3119	pOP-CNHP00359_EST_C_1_pSK_SK	728
cl2575	ct2833	cn3119	pOP-CNI01650_EST_C_1_pSK_SK	598
cl2575	ct2833	cn3119	pOP-CNI02252_EST_C_1_pSK_SK	585
cl2575	ct2833	cn3119	pOP-EO03026_EST_C_1_pSK_SK	495
cl2575	ct2833	cn3119	pOP-EO06077_EST_C_1_pSK_SK	424
cl2575	ct2833	cn3119	pOP-EO08068_EST_C_1_pSK_SK	468
cl2576	ct2834	cn3120	pOP-CNH01345_EST_C_1_pSK_SK	511
cl2576	ct2834	cn3120	pOP-EO08069_EST_C_1_pSK_SK	667
cl2577	ct2835	cn3121	pOP-EO06252_EST_C_1_pSK_SK	694
cl2577	ct2835	cn3121	pOP-EO06444_EST_C_1_pSK_SK	656
cl2577	ct2835	cn3121	pOP-EO08092_EST_C_1_pSK_SK	599
cl2578	ct2836	cn3122	pOP-EO05471_EST_C_1_pSK_SK	433
cl2578	ct2836	cn3122	pOP-EO08093_EST_C_1_pSK_SK	587
cl2579	ct2837	cn3123	pOP-EO08108_EST_C_1_pSK_SK	590
cl2579	ct2837	cn3124	pOP-CNI01423_EST_C_1_pSK_SK	520
cl2580	ct2838	cn3125	pOP-CEO00671_EST_C_1_pSK_SK	397
cl2580	ct2838	cn3125	pOP-CNH01585_EST_C_1_pSK_SK	584
cl2580	ct2838	cn3125	pOP-CNH02892_EST_C_1_pSK_SK	638
cl2580	ct2838	cn3125	pOP-CNI02014_EST_C_1_pSK_SK	137
cl2580	ct2838	cn3125	pOP-EO03428_EST_C_1_pSK_SK	536
cl2580	ct2838	cn3125	pOP-EO04962_EST_C_1_pSK_SK	503
cl2580	ct2838	cn3125	pOP-EO07310_EST_C_1_pSK_SK	659
cl2580	ct2839	cn3126	pOP-CEO00604_EST_C_1_pSK_SK	276
cl2580	ct2839	cn3126	pOP-CEO00686_EST_C_1_pSK_SK	301
cl2580	ct2839	cn3126	pOP-CEO01990_EST_C_1_pSK_SK	255
cl2580	ct2839	cn3126	pOP-CEO03102_EST_C_1_pSK_SK	187
cl2580	ct2839	cn3126	pOP-CNIP00160_EST_C_1_pSK_SK	155
cl2580	ct2839	cn3126	pOP-CNIP00444_EST_C_1_pSK_SK	484
cl2580	ct2839	cn3126	pOP-EAP00321_EST_C_1_pBSK_SK	154
cl2580	ct2839	cn3126	pOP-EAP01102_EST_C_1_pBSK_SK	625
cl2580	ct2839	cn3126	pOP-EAP01387_EST_C_1_pBSK_SK	256

cl2580	ct2839	cn3126	pOP-EAP01942_EST_C_1_pBSK_SK	436
cl2580	ct2839	cn3126	pOP-EAP03481_EST_C_1_pBSK_SK	255
cl2580	ct2839	cn3126	pOP-EAP03831_EST_C_1_pBSK_SK	571
cl2580	ct2839	cn3126	pOP-EN00554_EST_C_1_pSK_SK	421
cl2580	ct2839	cn3126	pOP-EO02123_EST_C_1_pSK_SK	500
cl2580	ct2839	cn3126	pOP-EO04636_EST_C_1_pSK_SK	533
cl2580	ct2839	cn3126	pOP-EO05488_EST_C_1_pSK_SK	520
cl2580	ct2839	cn3126	pOP-EO05517_EST_C_1_pSK_SK	425
cl2580	ct2839	cn3126	pOP-EO06293_EST_C_1_pSK_SK	675
cl2580	ct2839	cn3126	pOP-EO08002_EST_C_1_pSK_SK	345
cl2580	ct2839	cn3126	pOP-EO08110_EST_C_1_pSK_SK	511
cl2581	ct2840	cn3127	pOP-CEO01058_EST_C_1_pSK_SK	440
cl2581	ct2840	cn3127	pOP-CEO02153_EST_C_1_pSK_SK	577
cl2581	ct2840	cn3127	pOP-CEO02154_EST_C_1_pSK_SK	461
cl2581	ct2840	cn3127	pOP-CEO02719_EST_C_1_pSK_SK	447
cl2581	ct2840	cn3127	pOP-CEO02721_EST_C_1_pSK_SK	412
cl2581	ct2840	cn3127	pOP-CNH00820_EST_C_1_pSK_SK	600
cl2581	ct2840	cn3127	pOP-CNH01758_EST_C_1_pSK_SK	481
cl2581	ct2840	cn3127	pOP-CNI01488_EST_C_1_pSK_SK	532
cl2581	ct2840	cn3127	pOP-CNIP00850_EST_C_1_pSK_SK	525
cl2581	ct2840	cn3127	pOP-EAP01041_EST_C_1_pBSK_SK	291
cl2581	ct2840	cn3127	pOP-EAP01453_EST_C_1_pBSK_SK	291
cl2581	ct2840	cn3127	pOP-EO02616_EST_C_1_pSK_SK	373
cl2581	ct2841	cn3128	pOP-CEM00181_EST_C_1_pSK_SK	364
cl2581	ct2841	cn3128	pOP-CEO00609_EST_C_1_pSK_SK	421
cl2581	ct2841	cn3128	pOP-CEO00916_EST_C_1_pSK_SK	487
cl2581	ct2841	cn3128	pOP-CEO01120_EST_C_1_pSK_SK	315
cl2581	ct2841	cn3128	pOP-CEO01652_EST_C_1_pSK_SK	621
cl2581	ct2841	cn3128	pOP-CEO01953_EST_C_1_pSK_SK	476
cl2581	ct2841	cn3128	pOP-CEO02852_EST_C_1_pSK_SK	535
cl2581	ct2841	cn3128	pOP-CEO02903_EST_C_1_pSK_SK	714
cl2581	ct2841	cn3128	pOP-CEO03252_EST_C_1_pSK_SK	636
cl2581	ct2841	cn3128	pOP-CEO03315_EST_C_1_pSK_SK	638
cl2581	ct2841	cn3128	pOP-CEO03478_EST_C_1_pSK_SK	660
cl2581	ct2841	cn3128	pOP-CEO03577_EST_C_1_pSK_SK	485
cl2581	ct2841	cn3128	pOP-CEO03721_EST_C_1_pSK_SK	358
cl2581	ct2841	cn3128	pOP-CEOP00037_EST_C_1_pSK_SK	578
cl2581	ct2841	cn3128	pOP-CNH00547_EST_C_1_pSK_SK	533
cl2581	ct2841	cn3128	pOP-CNH00792_EST_C_1_pSK_SK	521
cl2581	ct2841	cn3128	pOP-CNH02130_EST_C_1_pSK_SK	521
cl2581	ct2841	cn3128	pOP-CNH02571_EST_C_1_pSK_SK	662
cl2581	ct2841	cn3128	pOP-CNH02888_EST_C_1_pSK_SK	574
cl2581	ct2841	cn3128	pOP-CNH03573_EST_C_1_pSK_SK	351
cl2581	ct2841	cn3128	pOP-CNH03609_EST_C_1_pSK_SK	342
cl2581	ct2841	cn3128	pOP-CNH04393	849
cl2581	ct2841	cn3128	pOP-CNH04531	779
cl2581	ct2841	cn3128	pOP-CNH04950_EST_C_1_pSK_SK	407
cl2581	ct2841	cn3128	pOP-CNH05085_EST_C_1_pSK_SK	462
cl2581	ct2841	cn3128	pOP-CNHP00095_EST_C_1_pSK_SK	290
cl2581	ct2841	cn3128	pOP-CNHP00485_EST_C_1_pSK_SK	626
cl2581	ct2841	cn3128	pOP-CNI01173_EST_C_1_pSK_SK	566
cl2581	ct2841	cn3128	pOP-EAP00080_EST_C_1_pBSK_SK	325
cl2581	ct2841	cn3128	pOP-EAP00081_EST_C_1_pBSK_SK	325
cl2581	ct2841	cn3128	pOP-EAP00516_EST_C_1_pBSK_SK	673
cl2581	ct2841	cn3128	pOP-EAP01337_EST_C_1_pBSK_SK	577
cl2581	ct2841	cn3128	pOP-EAP02367_EST_C_1_pBSK_SK	780

cl2581	ct2841	cn3128	pOP-EAP02870_EST_C_1_pBSK_SK	663
cl2581	ct2841	cn3128	pOP-EAP02950_EST_C_1_pBSK_SK	671
cl2581	ct2841	cn3128	pOP-EN00578_EST_C_1_pSK_SK	540
cl2581	ct2841	cn3128	pOP-EN00588_EST_C_1_pSK_SK	587
cl2581	ct2841	cn3128	pOP-EN00680_EST_C_1_pSK_SK	452
cl2581	ct2841	cn3128	pOP-EN00724_EST_C_1_pSK_SK	339
cl2581	ct2841	cn3128	pOP-EN00768_EST_C_1_pSK_SK	580
cl2581	ct2841	cn3128	pOP-EN00769_EST_C_1_pSK_SK	577
cl2581	ct2841	cn3128	pOP-EO03613_EST_C_1_pSK_SK	406
cl2581	ct2841	cn3128	pOP-EO03648_EST_C_1_pSK_SK	417
cl2581	ct2841	cn3128	pOP-EO03841_EST_C_1_pSK_SK	346
cl2581	ct2841	cn3128	pOP-EO03845_EST_C_1_pSK_SK	346
cl2581	ct2841	cn3128	pOP-EO03851_EST_C_1_pSK_SK	344
cl2581	ct2841	cn3128	pOP-EO03852_EST_C_1_pSK_SK	344
cl2581	ct2841	cn3128	pOP-EO03853_EST_C_1_pSK_SK	344
cl2581	ct2841	cn3128	pOP-EO03857_EST_C_1_pSK_SK	344
cl2581	ct2841	cn3128	pOP-EO03858_EST_C_1_pSK_SK	344
cl2581	ct2841	cn3128	pOP-EO03875_EST_C_1_pSK_SK	324
cl2581	ct2841	cn3128	pOP-EO03924_EST_C_1_pSK_SK	353
cl2581	ct2841	cn3128	pOP-EO03936_EST_C_1_pSK_SK	309
cl2581	ct2841	cn3128	pOP-EO04553_EST_C_1_pSK_SK	532
cl2581	ct2841	cn3128	pOP-EO04601_EST_C_1_pSK_SK	378
cl2581	ct2841	cn3128	pOP-EO04729_EST_C_1_pSK_SK	341
cl2581	ct2841	cn3128	pOP-EO05696_EST_C_1_pSK_SK	358
cl2581	ct2841	cn3128	pOP-EO05710_EST_C_1_pSK_SK	483
cl2581	ct2841	cn3128	pOP-EO05834_EST_C_1_pSK_SK	471
cl2581	ct2841	cn3128	pOP-EO05846_EST_C_1_pSK_SK	517
cl2581	ct2841	cn3128	pOP-EO06528_EST_C_1_pSK_SK	795
cl2581	ct2841	cn3128	pOP-EO06606_EST_C_1_pSK_SK	694
cl2581	ct2841	cn3128	pOP-EO06990_EST_C_1_pSK_SK	626
cl2581	ct2841	cn3128	pOP-EO07001_EST_C_1_pSK_SK	660
cl2581	ct2841	cn3128	pOP-EO07146_EST_C_1_pSK_SK	482
cl2581	ct2841	cn3128	pOP-EO07901_EST_C_1_pSK_SK	530
cl2581	ct2841	cn3128	pOP-EO08021_EST_C_1_pSK_SK	300
cl2581	ct2841	cn3128	pOP-EO08104_EST_C_1_pSK_SK	377
cl2581	ct2841	cn3128	pOP-EO08117_EST_C_1_pSK_SK	512
cl2581	ct2841	cn3129	pOP-CEO00576_EST_C_1_pSK_SK	681
cl2581	ct2841	cn3129	pOP-CEO02763_EST_C_1_pSK_SK	373
cl2582	ct2842	cn3130	pOP-CNIP00425_EST_C_1_pSK_SK	265
cl2582	ct2842	cn3130	pOP-EAP01107_EST_C_1_pBSK_SK	134
cl2582	ct2842	cn3130	pOP-EAP01756_EST_C_1_pBSK_SK	382
cl2582	ct2842	cn3130	pOP-EO02346_EST_C_1_pSK_SK	458
cl2582	ct2842	cn3130	pOP-EO08082_EST_C_1_pSK_SK	444
cl2582	ct2842	cn3130	pOP-EO08121_EST_C_1_pSK_SK	521
cl2583	ct2843	cn3131	pOP-CEO01133_EST_C_1_pSK_SK	374
cl2583	ct2843	cn3131	pOP-CNI01266_EST_C_1_pSK_SK	447
cl2583	ct2843	cn3131	pOP-CNI01544_EST_C_1_pSK_SK	313
cl2583	ct2843	cn3131	pOP-EO06384_EST_C_1_pSK_SK	375
cl2583	ct2843	cn3131	pOP-EO08122_EST_C_1_pSK_SK	351
cl2584	ct2844	cn3132	pOP-EO06150_EST_C_1_pSK_SK	470
cl2584	ct2844	cn3132	pOP-EO08125_EST_C_1_pSK_SK	537
cl2585	ct2845	cn3133	pOP-EN00613_EST_C_1_pSK_SK	485
cl2585	ct2845	cn3133	pOP-EO08131_EST_C_1_pSK_SK	499
cl2586	ct2846	cn3134	pOP-EAP01103_EST_C_1_pBSK_SK	218
cl2586	ct2846	cn3134	pOP-EO07233_EST_C_1_pSK_SK	721
cl2586	ct2846	cn3134	pOP-EO08133_EST_C_1_pSK_SK	579

cl2586	ct2847	cn3135	pOP-EAP00716_EST_C_1_pBSK_SK	524
cl2586	ct2847	cn3135	pOP-EAP00736_EST_C_1_pBSK_SK	555
cl2586	ct2847	cn3135	pOP-EO04815_EST_C_1_pSK_SK	567
cl2586	ct2847	cn3135	pOP-EO05644_EST_C_1_pSK_SK	371
cl2586	ct2847	cn3135	pOP-EO06821_EST_C_1_pSK_SK	633
cl2586	ct2847	cn3136	pOP-CEO00952_EST_C_1_pSK_SK	433
cl2587	ct2848	cn3137	pOP-EO04730_EST_C_1_pSK_SK	516
cl2587	ct2848	cn3137	pOP-EO08145_EST_C_1_pSK_SK	496
cl2588	ct2849	cn3138	pOP-CNI01158_EST_C_1_pSK_SK	408
cl2588	ct2849	cn3138	pOP-CNI01159_EST_C_1_pSK_SK	698
cl2588	ct2849	cn3138	pOP-CNIP00905_EST_C_1_pSK_SK	544
cl2588	ct2849	cn3138	pOP-EO07160_EST_C_1_pSK_SK	531
cl2588	ct2849	cn3138	pOP-EO08153_EST_C_1_pSK_SK	436
cl2589	ct2850	cn3139	pOP-EO06151_EST_C_1_pSK_SK	549
cl2589	ct2851	cn3140	pOP-EO08159_EST_C_1_pSK_SK	638
cl2590	ct2852	cn3141	pOP-EO05059_EST_C_1_pSK_SK	520
cl2590	ct2852	cn3141	pOP-EO08165_EST_C_1_pSK_SK	556
cl2590	ct2852	cn3142	pOP-EO08012_EST_C_1_pSK_SK	355
cl2591	ct2853	cn3143	pOP-CEO00711_EST_C_1_pSK_SK	521
cl2591	ct2854	cn3144	pOP-CEO00821_EST_C_1_pSK_SK	367
cl2591	ct2854	cn3144	pOP-CEO01764_EST_C_1_pSK_SK	224
cl2591	ct2854	cn3144	pOP-CEO03115_EST_C_1_pSK_SK	180
cl2591	ct2854	cn3144	pOP-EAP01138_EST_C_1_pBSK_SK	188
cl2591	ct2854	cn3144	pOP-EAP01513_EST_C_1_pBSK_SK	613
cl2591	ct2854	cn3144	pOP-EAP02233_EST_C_1_pBSK_SK	338
cl2591	ct2854	cn3144	pOP-EO08167_EST_C_1_pSK_SK	630
cl2591	ct2855	cn3145	pOP-CEO01712_EST_C_1_pSK_SK	183
cl2591	ct2855	cn3145	pOP-CEO03163_EST_C_1_pSK_SK	511
cl2591	ct2855	cn3146	pOP-CEM00206_EST_C_1_pSK_SK	365
cl2591	ct2855	cn3147	pOP-CEO02621_EST_C_1_pSK_SK	221
cl2591	ct2855	cn3147	pOP-CEO03658_EST_C_1_pSK_SK	308
cl2591	ct2855	cn3147	pOP-EO04995_EST_C_1_pSK_SK	516
cl2591	ct2855	cn3147	pOP-EO06101_EST_C_1_pSK_SK	446
cl2592	ct2856	cn3148	pOP-CNH00675_EST_C_1_pSK_SK	558
cl2592	ct2856	cn3148	pOP-CNH02707_EST_C_1_pSK_SK	568
cl2592	ct2856	cn3148	pOP-EO08169_EST_C_1_pSK_SK	599
cl2593	ct2857	cn3149	pOP-EN00137_EST_C_1_pSK_SK	653
cl2593	ct2857	cn3149	pOP-EO08171_EST_C_1_pSK_SK	481
cl2594	ct2858	cn3150	pOP-EO03907_EST_C_1_pSK_SK	562
cl2594	ct2858	cn3150	pOP-EO08172_EST_C_1_pSK_SK	539
cl2595	ct2859	cn3151	pOP-EAP01595_EST_C_1_pBSK_SK	450
cl2595	ct2859	cn3151	pOP-EN00549_EST_C_1_pSK_SK	432
cl2595	ct2859	cn3151	pOP-EO02405_EST_C_1_pSK_SK	567
cl2595	ct2859	cn3151	pOP-EO03799_EST_C_1_pSK_SK	388
cl2595	ct2859	cn3151	pOP-EO04863_EST_C_1_pSK_SK	509
cl2595	ct2859	cn3151	pOP-EO08178_EST_C_1_pSK_SK	552
cl2596	ct2860	cn3152	pOP-EO04574_EST_C_1_pSK_SK	396
cl2596	ct2860	cn3152	pOP-EO08181_EST_C_1_pSK_SK	586
cl2597	ct2861	cn3153	pOP-EO03874_EST_C_1_pSK_SK	327
cl2597	ct2861	cn3153	pOP-EO08183_EST_C_1_pSK_SK	484
cl2598	ct2862	cn3154	pOP-EO05260_EST_C_1_pSK_SK	460
cl2598	ct2862	cn3154	pOP-EO08177_EST_C_1_pSK_SK	571
cl2598	ct2862	cn3154	pOP-EO08185_EST_C_1_pSK_SK	591
cl2599	ct2863	cn3155	pOP-CEO02125_EST_C_1_pSK_SK	526
cl2599	ct2863	cn3155	pOP-EO08187_EST_C_1_pSK_SK	680
cl2599	ct2863	cn3156	pOP-CNH02677_EST_C_1_pSK_SK	563

cl2600	ct2864	cn3157	pOP-CEO02817_EST_C_1_pSK_SK	409
cl2600	ct2864	cn3157	pOP-EAP00263_EST_C_1_pBSK_SK	108
cl2600	ct2864	cn3157	pOP-EAP00266_EST_C_1_pBSK_SK	242
cl2600	ct2864	cn3157	pOP-EAP00536_EST_C_1_pBSK_SK	536
cl2600	ct2864	cn3157	pOP-EAP00638_EST_C_1_pBSK_SK	573
cl2600	ct2864	cn3157	pOP-EAP00639_EST_C_1_pBSK_SK	567
cl2600	ct2864	cn3157	pOP-EAP01020_EST_C_1_pBSK_SK	148
cl2600	ct2864	cn3157	pOP-EAP02386_EST_C_1_pBSK_SK	469
cl2600	ct2864	cn3157	pOP-EAP02864_EST_C_1_pBSK_SK	587
cl2600	ct2864	cn3157	pOP-EAP03667_EST_C_1_pBSK_SK	447
cl2600	ct2864	cn3157	pOP-EAP05044_EST_C_1_pBSK_SK	544
cl2600	ct2864	cn3157	pOP-EN00301_EST_C_1_pSK_SK	524
cl2600	ct2864	cn3157	pOP-EN00333_EST_C_1_pSK_SK	482
cl2600	ct2864	cn3157	pOP-EN00640_EST_C_1_pSK_SK	518
cl2600	ct2864	cn3157	pOP-EN00669_EST_C_1_pSK_SK	412
cl2600	ct2864	cn3157	pOP-EN00817_EST_C_1_pSK_SK	444
cl2600	ct2864	cn3157	pOP-EN00863_EST_C_1_pSK_SK	541
cl2600	ct2864	cn3157	pOP-EO02972_EST_C_1_pSK_SK	365
cl2600	ct2864	cn3157	pOP-EO03077_EST_C_1_pSK_SK	478
cl2600	ct2864	cn3157	pOP-EO03519_EST_C_1_pSK_SK	347
cl2600	ct2864	cn3157	pOP-EO03959_EST_C_1_pSK_SK	488
cl2600	ct2864	cn3157	pOP-EO04007_EST_C_1_pSK_SK	519
cl2600	ct2864	cn3157	pOP-EO04506_EST_C_1_pSK_SK	485
cl2600	ct2864	cn3157	pOP-EO04578_EST_C_1_pSK_SK	282
cl2600	ct2864	cn3157	pOP-EO04651_EST_C_1_pSK_SK	518
cl2600	ct2864	cn3157	pOP-EO04805_EST_C_1_pSK_SK	551
cl2600	ct2864	cn3157	pOP-EO04827_EST_C_1_pSK_SK	564
cl2600	ct2864	cn3157	pOP-EO04828_EST_C_1_pSK_SK	587
cl2600	ct2864	cn3157	pOP-EO04903_EST_C_1_pSK_SK	525
cl2600	ct2864	cn3157	pOP-EO05386_EST_C_1_pSK_SK	487
cl2600	ct2864	cn3157	pOP-EO05593_EST_C_1_pSK_SK	490
cl2600	ct2864	cn3157	pOP-EO05784_EST_C_1_pSK_SK	619
cl2600	ct2864	cn3157	pOP-EO06001_EST_C_1_pSK_SK	622
cl2600	ct2864	cn3157	pOP-EO06299_EST_C_1_pSK_SK	557
cl2600	ct2864	cn3157	pOP-EO06441_EST_C_1_pSK_SK	708
cl2600	ct2864	cn3157	pOP-EO07114_EST_C_1_pSK_SK	668
cl2600	ct2864	cn3157	pOP-EO07300_EST_C_1_pSK_SK	719
cl2600	ct2864	cn3157	pOP-EO07329_EST_C_1_pSK_SK	677
cl2600	ct2864	cn3157	pOP-EO07985_EST_C_1_pSK_SK	322
cl2600	ct2864	cn3157	pOP-EO08105_EST_C_1_pSK_SK	593
cl2600	ct2864	cn3157	pOP-EO08157_EST_C_1_pSK_SK	590
cl2600	ct2864	cn3157	pOP-EO08191_EST_C_1_pSK_SK	638
cl2600	ct2864	cn3157	pOP-EO08294_EST_C_1_pSK_SK	451
cl2600	ct2864	cn3157	pOP-EO08518_EST_C_1_pSK_SK	177
cl2601	ct2865	cn3158	pOP-EO05680_EST_C_1_pSK_SK	472
cl2601	ct2865	cn3158	pOP-EO08193_EST_C_1_pSK_SK	596
cl2602	ct2866	cn3159	pOP-CEO02943_EST_C_1_pSK_SK	534
cl2602	ct2866	cn3159	pOP-EAP00426_EST_C_1_pBSK_SK	423
cl2602	ct2866	cn3159	pOP-EO07320_EST_C_1_pSK_SK	819
cl2602	ct2866	cn3159	pOP-EO08202_EST_C_1_pSK_SK	670
cl2603	ct2867	cn3160	pOP-CAP00013_EST_C_1_pBSK_SK	657
cl2603	ct2867	cn3160	pOP-EO08203_EST_C_1_pSK_SK	457
cl2604	ct2868	cn3161	pOP-EAP02791_EST_C_1_pBSK_SK	588
cl2604	ct2868	cn3162	pOP-EO08210_EST_C_1_pSK_SK	498
cl2605	ct2869	cn3163	pOP-EO05575_EST_C_1_pSK_SK	457
cl2605	ct2869	cn3163	pOP-EO08212_EST_C_1_pSK_SK	330

cl2606	ct2870	cn3164	pOP-EO05617_EST_C_1_pSK_SK	463
cl2606	ct2870	cn3164	pOP-EO08218_EST_C_1_pSK_SK	462
cl2607	ct2871	cn3165	pOP-EO04793_EST_C_1_pSK_SK	534
cl2607	ct2871	cn3165	pOP-EO08226_EST_C_1_pSK_SK	567
cl2608	ct2872	cn3166	pOP-EO02009_EST_C_1_pSK_SK	587
cl2608	ct2872	cn3166	pOP-EO05676_EST_C_1_pSK_SK	409
cl2608	ct2872	cn3166	pOP-EO08232_EST_C_1_pSK_SK	652
cl2609	ct2873	cn3167	pOP-CEO02855_EST_C_1_pSK_SK	423
cl2609	ct2873	cn3167	pOP-CNH00551_EST_C_1_pSK_SK	618
cl2609	ct2873	cn3167	pOP-CNH02851_EST_C_1_pSK_SK	678
cl2609	ct2873	cn3167	pOP-CNH02957_EST_C_1_pSK_SK	600
cl2609	ct2873	cn3167	pOP-CNHP00427_EST_C_1_pSK_SK	721
cl2609	ct2873	cn3167	pOP-CNI01272_EST_C_1_pSK_SK	376
cl2609	ct2873	cn3167	pOP-CNIP00098_EST_C_1_pSK_SK	593
cl2609	ct2873	cn3167	pOP-EAP05040_EST_C_1_pBSK_SK	661
cl2609	ct2873	cn3167	pOP-EO03106_EST_C_1_pSK_SK	431
cl2609	ct2873	cn3168	pOP-EO07389_EST_C_1_pSK_SK	728
cl2609	ct2873	cn3169	pOP-EO02725_EST_C_1_pSK_SK	448
cl2609	ct2873	cn3170	pOP-EO08235_EST_C_1_pSK_SK	326
cl2610	ct2874	cn3171	pOP-CNH04373	670
cl2610	ct2875	cn3172	pOP-EO03022_EST_C_1_pSK_SK	504
cl2610	ct2875	cn3172	pOP-EO08236_EST_C_1_pSK_SK	503
cl2610	ct2876	cn3173	pOP-CEO02377_EST_C_1_pSK_SK	580
cl2610	ct2876	cn3173	pOP-EAP02736_EST_C_1_pBSK_SK	552
cl2610	ct2876	cn3173	pOP-EO06656_EST_C_1_pSK_SK	701
cl2610	ct2876	cn3173	pOP-EO06767_EST_C_1_pSK_SK	899
cl2611	ct2877	cn3174	pOP-EO06909_EST_C_1_pSK_SK	381
cl2611	ct2877	cn3174	pOP-EO08239_EST_C_1_pSK_SK	644
cl2612	ct2878	cn3175	pOP-EO05665_EST_C_1_pSK_SK	332
cl2612	ct2878	cn3175	pOP-EO08240_EST_C_1_pSK_SK	201
cl2613	ct2879	cn3176	pOP-CNH01915_EST_C_1_pSK_SK	630
cl2613	ct2879	cn3176	pOP-CNH02656_EST_C_1_pSK_SK	619
cl2613	ct2879	cn3176	pOP-CNH03494_EST_C_1_pSK_SK	583
cl2613	ct2879	cn3176	pOP-EO08250_EST_C_1_pSK_SK	594
cl2614	ct2880	cn3177	pOP-EO03720_EST_C_1_pSK_SK	360
cl2614	ct2880	cn3177	pOP-EO06303_EST_C_1_pSK_SK	530
cl2614	ct2880	cn3177	pOP-EO08255_EST_C_1_pSK_SK	463
cl2615	ct2881	cn3178	pOP-EAP00644_EST_C_1_pBSK_SK	545
cl2615	ct2881	cn3178	pOP-EN00183_EST_C_1_pSK_SK	373
cl2615	ct2881	cn3178	pOP-EN00224_EST_C_1_pSK_SK	500
cl2615	ct2881	cn3178	pOP-EN00446_EST_C_1_pSK_SK	512
cl2615	ct2881	cn3178	pOP-EO02020_EST_C_1_pSK_SK	486
cl2615	ct2881	cn3178	pOP-EO06924_EST_C_1_pSK_SK	718
cl2615	ct2881	cn3178	pOP-EO08259_EST_C_1_pSK_SK	516
cl2615	ct2882	cn3179	pOP-CNH01643_EST_C_1_pSK_SK	641
cl2615	ct2882	cn3179	pOP-CNH01812_EST_C_1_pSK_SK	462
cl2615	ct2882	cn3179	pOP-CNH02030_EST_C_1_pSK_SK	490
cl2615	ct2882	cn3179	pOP-CNH03465_EST_C_1_pSK_SK	292
cl2615	ct2882	cn3179	pOP-CNH04246	533
cl2615	ct2882	cn3179	pOP-CNH04272	531
cl2615	ct2882	cn3179	pOP-CNI02024_EST_C_1_pSK_SK	484
cl2615	ct2882	cn3179	pOP-EO04339_EST_C_1_pSK_SK	526
cl2615	ct2882	cn3180	pOP-CNI02250_EST_C_1_pSK_SK	700
cl2615	ct2882	cn3181	pOP-CNH03541_EST_C_1_pSK_SK	368
cl2616	ct2883	cn3182	pOP-CAP00028_EST_C_1_pBSK_SK	393
cl2616	ct2883	cn3182	pOP-CEO02213_EST_C_1_pSK_SK	512

cl2616	ct2883	cn3182	pOP-CNI01347_EST_C_1_pSK_SK	501
cl2616	ct2883	cn3182	pOP-EAP01072_EST_C_1_pBSK_SK	318
cl2616	ct2883	cn3182	pOP-EO02039_EST_C_1_pSK_SK	511
cl2616	ct2883	cn3182	pOP-EO02979_EST_C_1_pSK_SK	365
cl2616	ct2883	cn3182	pOP-EO05443_EST_C_1_pSK_SK	401
cl2616	ct2883	cn3182	pOP-EO05482_EST_C_1_pSK_SK	479
cl2616	ct2883	cn3182	pOP-EO06265_EST_C_1_pSK_SK	670
cl2616	ct2883	cn3182	pOP-EO07288_EST_C_1_pSK_SK	695
cl2616	ct2883	cn3182	pOP-EO08264_EST_C_1_pSK_SK	503
cl2617	ct2884	cn3183	pOP-EO06982_EST_C_1_pSK_SK	822
cl2617	ct2884	cn3183	pOP-EO08265_EST_C_1_pSK_SK	569
cl2618	ct2885	cn3184	pOP-CAP00301_EST_C_1_pBSK_SK	435
cl2618	ct2885	cn3184	pOP-EN00362_EST_C_1_pSK_SK	555
cl2618	ct2885	cn3184	pOP-EN00465_EST_C_1_pSK_SK	515
cl2618	ct2885	cn3184	pOP-EO02261_EST_C_1_pSK_SK	618
cl2618	ct2885	cn3184	pOP-EO03147_EST_C_1_pSK_SK	404
cl2618	ct2885	cn3184	pOP-EO03750_EST_C_1_pSK_SK	477
cl2618	ct2885	cn3184	pOP-EO03886_EST_C_1_pSK_SK	404
cl2618	ct2885	cn3184	pOP-EO05856_EST_C_1_pSK_SK	632
cl2618	ct2885	cn3184	pOP-EO07255_EST_C_1_pSK_SK	733
cl2618	ct2885	cn3184	pOP-EO08266_EST_C_1_pSK_SK	416
cl2619	ct2886	cn3185	pOP-CNI01666_EST_C_1_pSK_SK	466
cl2619	ct2886	cn3185	pOP-EO07492_EST_C_1_pSK_SK	439
cl2619	ct2887	cn3186	pOP-CAP00255_EST_C_1_pBSK_SK	534
cl2619	ct2887	cn3186	pOP-CEM00086_EST_C_1_pSK_SK	619
cl2619	ct2887	cn3186	pOP-CEO00511_EST_C_1_pSK_SK	368
cl2619	ct2887	cn3186	pOP-EO02537_EST_C_1_pSK_SK	530
cl2619	ct2887	cn3186	pOP-EO05724_EST_C_1_pSK_SK	386
cl2619	ct2887	cn3186	pOP-EO05928_EST_C_1_pSK_SK	601
cl2619	ct2887	cn3186	pOP-EO06195_EST_C_1_pSK_SK	564
cl2619	ct2887	cn3186	pOP-EO06258_EST_C_1_pSK_SK	473
cl2619	ct2887	cn3186	pOP-EO06452_EST_C_1_pSK_SK	755
cl2619	ct2887	cn3186	pOP-EO06465_EST_C_1_pSK_SK	782
cl2619	ct2887	cn3186	pOP-EO08269_EST_C_1_pSK_SK	513
cl2620	ct2888	cn3187	pOP-CEO03642_EST_C_1_pSK_SK	222
cl2620	ct2888	cn3187	pOP-EO08271_EST_C_1_pSK_SK	665
cl2621	ct2889	cn3188	pOP-CAP00393_EST_C_1_pBSK_SK	651
cl2621	ct2889	cn3188	pOP-EO08275_EST_C_1_pSK_SK	515
cl2622	ct2890	cn3189	pOP-EO06057_EST_C_1_pSK_SK	634
cl2622	ct2890	cn3189	pOP-EO08282_EST_C_1_pSK_SK	620
cl2623	ct2891	cn3190	pOP-CNIP00228_EST_C_1_pSK_SK	387
cl2623	ct2891	cn3190	pOP-EAP00973_EST_C_1_pBSK_SK	172
cl2623	ct2892	cn3191	pOP-EAP01970_EST_C_1_pBSK_SK	510
cl2623	ct2892	cn3191	pOP-EO08284_EST_C_1_pSK_SK	600
cl2624	ct2893	cn3192	pOP-EO05098_EST_C_1_pSK_SK	467
cl2624	ct2893	cn3192	pOP-EO05972_EST_C_1_pSK_SK	376
cl2624	ct2893	cn3192	pOP-EO08286_EST_C_1_pSK_SK	563
cl2625	ct2894	cn3193	pOP-EO07212_EST_C_1_pSK_SK	649
cl2625	ct2894	cn3193	pOP-EO07724_EST_C_1_pSK_SK	374
cl2625	ct2894	cn3193	pOP-EO08296_EST_C_1_pSK_SK	428
cl2626	ct2895	cn3194	pOP-EO07943_EST_C_1_pSK_SK	691
cl2626	ct2895	cn3194	pOP-EO08297_EST_C_1_pSK_SK	466
cl2627	ct2896	cn3195	pOP-EO06006_EST_C_1_pSK_SK	575
cl2627	ct2896	cn3195	pOP-EO08299_EST_C_1_pSK_SK	543
cl2628	ct2897	cn3196	pOP-EO05606_EST_C_1_pSK_SK	495
cl2628	ct2897	cn3196	pOP-EO08301_EST_C_1_pSK_SK	476

cl2629	ct2898	cn3197	pOP-CEM00062_EST_C_1_pSK_SK	426
cl2629	ct2898	cn3197	pOP-CEM00258_EST_C_1_pSK_SK	430
cl2629	ct2898	cn3197	pOP-CNHP00047_EST_C_1_pSK_SK	642
cl2629	ct2898	cn3197	pOP-CNI01433_EST_C_1_pSK_SK	557
cl2629	ct2898	cn3197	pOP-EAP01539_EST_C_1_pBSK_SK	424
cl2629	ct2898	cn3197	pOP-EAP03839_EST_C_1_pBSK_SK	666
cl2629	ct2898	cn3197	pOP-EAP03863_EST_C_1_pBSK_SK	294
cl2629	ct2898	cn3197	pOP-EO06483_EST_C_1_pSK_SK	677
cl2629	ct2898	cn3197	pOP-EO08302_EST_C_1_pSK_SK	561
cl2630	ct2899	cn3198	pOP-EO06926_EST_C_1_pSK_SK	745
cl2630	ct2899	cn3198	pOP-EO08316_EST_C_1_pSK_SK	503
cl2631	ct2900	cn3199	pOP-EO07768_EST_C_1_pSK_SK	806
cl2631	ct2900	cn3199	pOP-EO08318_EST_C_1_pSK_SK	574
cl2632	ct2901	cn3200	pOP-EO06099_EST_C_1_pSK_SK	676
cl2632	ct2902	cn3201	pOP-EO06328_EST_C_1_pSK_SK	680
cl2632	ct2903	cn3202	pOP-CEO00542_EST_C_1_pSK_SK	359
cl2632	ct2903	cn3202	pOP-CEO00866_EST_C_1_pSK_SK	372
cl2632	ct2903	cn3202	pOP-CNH01464_EST_C_1_pSK_SK	598
cl2632	ct2903	cn3202	pOP-EAP00636_EST_C_1_pBSK_SK	589
cl2632	ct2903	cn3202	pOP-EO02288_EST_C_1_pSK_SK	523
cl2632	ct2903	cn3202	pOP-EO02474_EST_C_1_pSK_SK	476
cl2632	ct2903	cn3202	pOP-EO05150_EST_C_1_pSK_SK	436
cl2632	ct2903	cn3202	pOP-EO05468_EST_C_1_pSK_SK	469
cl2632	ct2903	cn3202	pOP-EO06182_EST_C_1_pSK_SK	553
cl2632	ct2903	cn3202	pOP-EO08322_EST_C_1_pSK_SK	530
cl2633	ct2904	cn3203	pOP-EO08209_EST_C_1_pSK_SK	620
cl2633	ct2904	cn3203	pOP-EO08324_EST_C_1_pSK_SK	401
cl2634	ct2905	cn3204	pOP-CNH03383_EST_C_1_pSK_SK	542
cl2634	ct2905	cn3204	pOP-EO07995_EST_C_1_pSK_SK	222
cl2634	ct2905	cn3204	pOP-EO08328_EST_C_1_pSK_SK	514
cl2635	ct2906	cn3205	pOP-CEO03427_EST_C_1_pSK_SK	314
cl2635	ct2906	cn3205	pOP-CNI01328_EST_C_1_pSK_SK	342
cl2635	ct2906	cn3206	pOP-CEO03123_EST_C_1_pSK_SK	307
cl2635	ct2907	cn3207	pOP-CNI01589_EST_C_1_pSK_SK	191
cl2635	ct2907	cn3207	pOP-CNI01793_EST_C_1_pSK_SK	363
cl2635	ct2907	cn3207	pOP-CNI01995_EST_C_1_pSK_SK	477
cl2635	ct2907	cn3207	pOP-CNI02205_EST_C_1_pSK_SK	355
cl2635	ct2907	cn3207	pOP-CNI02237_EST_C_1_pSK_SK	601
cl2635	ct2907	cn3207	pOP-CNIP00136_EST_C_1_pSK_SK	472
cl2635	ct2907	cn3207	pOP-CNIP00175_EST_C_1_pSK_SK	502
cl2635	ct2907	cn3207	pOP-CNIP00211_EST_C_1_pSK_SK	267
cl2635	ct2907	cn3207	pOP-CNIP00594_EST_C_1_pSK_SK	289
cl2635	ct2907	cn3207	pOP-CNIP00968_EST_C_1_pSK_SK	472
cl2635	ct2907	cn3207	pOP-CNIP00971_EST_C_1_pSK_SK	274
cl2635	ct2907	cn3207	pOP-EO02984_EST_C_1_pSK_SK	283
cl2635	ct2907	cn3207	pOP-EO03000_EST_C_1_pSK_SK	241
cl2635	ct2907	cn3207	pOP-EO03031_EST_C_1_pSK_SK	444
cl2635	ct2907	cn3207	pOP-EO03375_EST_C_1_pSK_SK	462
cl2635	ct2907	cn3207	pOP-EO04248_EST_C_1_pSK_SK	361
cl2635	ct2907	cn3207	pOP-EO04879_EST_C_1_pSK_SK	491
cl2635	ct2907	cn3207	pOP-EO06054_EST_C_1_pSK_SK	636
cl2635	ct2907	cn3207	pOP-EO07298_EST_C_1_pSK_SK	619
cl2635	ct2907	cn3207	pOP-EO07961_EST_C_1_pSK_SK	397
cl2635	ct2907	cn3207	pOP-EO08214_EST_C_1_pSK_SK	637
cl2635	ct2907	cn3207	pOP-EO08329_EST_C_1_pSK_SK	588
cl2636	ct2908	cn3208	pOP-EO02321_EST_C_1_pSK_SK	578

cl2636	ct2908	cn3208	pOP-EO03119_EST_C_1_pSK_SK	402
cl2636	ct2908	cn3208	pOP-EO04414_EST_C_1_pSK_SK	528
cl2636	ct2908	cn3208	pOP-EO07266_EST_C_1_pSK_SK	670
cl2636	ct2908	cn3208	pOP-EO08334_EST_C_1_pSK_SK	376
cl2637	ct2909	cn3209	pOP-EO04093_EST_C_1_pSK_SK	553
cl2637	ct2909	cn3209	pOP-EO04198_EST_C_1_pSK_SK	358
cl2637	ct2909	cn3209	pOP-EO04314_EST_C_1_pSK_SK	488
cl2637	ct2909	cn3209	pOP-EO08336_EST_C_1_pSK_SK	564
cl2638	ct2910	cn3210	pOP-EO05816_EST_C_1_pSK_SK	546
cl2638	ct2910	cn3210	pOP-EO07899_EST_C_1_pSK_SK	666
cl2638	ct2910	cn3210	pOP-EO08141_EST_C_1_pSK_SK	546
cl2638	ct2910	cn3210	pOP-EO08338_EST_C_1_pSK_SK	531
cl2639	ct2911	cn3211	pOP-EO06845_EST_C_1_pSK_SK	621
cl2639	ct2911	cn3211	pOP-EO08348_EST_C_1_pSK_SK	361
cl2640	ct2912	cn3212	pOP-EO05616_EST_C_1_pSK_SK	497
cl2640	ct2912	cn3212	pOP-EO08353_EST_C_1_pSK_SK	438
cl2641	ct2913	cn3213	pOP-EN00467_EST_C_1_pSK_SK	516
cl2641	ct2913	cn3213	pOP-EO08357_EST_C_1_pSK_SK	415
cl2642	ct2914	cn3214	pOP-CAP00030_EST_C_1_pBSK_SK	421
cl2642	ct2914	cn3214	pOP-CEO01979_EST_C_1_pSK_SK	340
cl2642	ct2914	cn3214	pOP-CNI01509_EST_C_1_pSK_SK	268
cl2642	ct2914	cn3214	pOP-CNIP00553_EST_C_1_pSK_SK	206
cl2642	ct2914	cn3214	pOP-EAP01163_EST_C_1_pBSK_SK	223
cl2642	ct2914	cn3214	pOP-EO03396_EST_C_1_pSK_SK	362
cl2642	ct2914	cn3214	pOP-EO05510_EST_C_1_pSK_SK	503
cl2642	ct2914	cn3214	pOP-EO05690_EST_C_1_pSK_SK	414
cl2642	ct2914	cn3214	pOP-EO05851_EST_C_1_pSK_SK	552
cl2642	ct2914	cn3214	pOP-EO05973_EST_C_1_pSK_SK	531
cl2642	ct2914	cn3214	pOP-EO06142_EST_C_1_pSK_SK	575
cl2642	ct2914	cn3214	pOP-EO06157_EST_C_1_pSK_SK	580
cl2642	ct2914	cn3214	pOP-EO07957_EST_C_1_pSK_SK	755
cl2642	ct2914	cn3214	pOP-EO08126_EST_C_1_pSK_SK	389
cl2642	ct2914	cn3214	pOP-EO08364_EST_C_1_pSK_SK	310
cl2643	ct2915	cn3215	pOP-CNH00726_EST_C_1_pSK_SK	675
cl2643	ct2915	cn3215	pOP-CNH01666_EST_C_1_pSK_SK	600
cl2643	ct2915	cn3215	pOP-CNH01784_EST_C_1_pSK_SK	566
cl2643	ct2915	cn3215	pOP-CNH01958_EST_C_1_pSK_SK	668
cl2643	ct2915	cn3215	pOP-CNH02153_EST_C_1_pSK_SK	572
cl2643	ct2915	cn3215	pOP-CNH02675_EST_C_1_pSK_SK	727
cl2643	ct2915	cn3215	pOP-CNH02687_EST_C_1_pSK_SK	607
cl2643	ct2915	cn3215	pOP-CNH02811_EST_C_1_pSK_SK	572
cl2643	ct2915	cn3215	pOP-CNH02877_EST_C_1_pSK_SK	501
cl2643	ct2915	cn3215	pOP-CNH02964_EST_C_1_pSK_SK	577
cl2643	ct2915	cn3215	pOP-CNH03047_EST_C_1_pSK_SK	528
cl2643	ct2915	cn3215	pOP-CNH03251_EST_C_1_pSK_SK	548
cl2643	ct2915	cn3215	pOP-CNH03771_EST_C_1_pSK_SK	445
cl2643	ct2915	cn3215	pOP-CNH04240	565
cl2643	ct2915	cn3215	pOP-CNH04321	594
cl2643	ct2915	cn3215	pOP-CNH04342	597
cl2643	ct2915	cn3215	pOP-CNH04361	720
cl2643	ct2915	cn3215	pOP-CNH04428	721
cl2643	ct2915	cn3215	pOP-CNH04684	587
cl2643	ct2915	cn3215	pOP-CNH04802_EST_C_1_pSK_SK	524
cl2643	ct2915	cn3215	pOP-CNHP00491_EST_C_1_pSK_SK	611
cl2643	ct2915	cn3215	pOP-EAP02351_EST_C_1_pBSK_SK	747
cl2643	ct2915	cn3215	pOP-EO08137_EST_C_1_pSK_SK	584

cl2643	ct2915	cn3215	pOP-EO08138_EST_C_1_pSK_SK	602
cl2643	ct2915	cn3215	pOP-EO08369_EST_C_1_pSK_SK	406
cl2643	ct2915	cn3216	pOP-CNH02035_EST_C_1_pSK_SK	414
cl2644	ct2916	cn3217	pOP-EO02048_EST_C_1_pSK_SK	444
cl2644	ct2916	cn3217	pOP-EO08372_EST_C_1_pSK_SK	181
cl2645	ct2917	cn3218	pOP-EO03183_EST_C_1_pSK_SK	472
cl2645	ct2917	cn3218	pOP-EO04050_EST_C_1_pSK_SK	601
cl2645	ct2917	cn3218	pOP-EO04218_EST_C_1_pSK_SK	544
cl2645	ct2917	cn3218	pOP-EO05240_EST_C_1_pSK_SK	483
cl2645	ct2917	cn3218	pOP-EO06555_EST_C_1_pSK_SK	826
cl2645	ct2917	cn3218	pOP-EO07962_EST_C_1_pSK_SK	528
cl2645	ct2917	cn3218	pOP-EO08380_EST_C_1_pSK_SK	243
cl2645	ct2917	cn3219	pOP-CEO01434_EST_C_1_pSK_SK	357
cl2645	ct2917	cn3219	pOP-CNH02075_EST_C_1_pSK_SK	560
cl2645	ct2917	cn3219	pOP-EO03120_EST_C_1_pSK_SK	408
cl2646	ct2918	cn3220	pOP-EO03868_EST_C_1_pSK_SK	440
cl2646	ct2918	cn3220	pOP-EO08386_EST_C_1_pSK_SK	338
cl2647	ct2919	cn3221	pOP-EO03443_EST_C_1_pSK_SK	534
cl2647	ct2919	cn3221	pOP-EO05893_EST_C_1_pSK_SK	585
cl2647	ct2919	cn3221	pOP-EO06669_EST_C_1_pSK_SK	663
cl2647	ct2919	cn3221	pOP-EO06754_EST_C_1_pSK_SK	663
cl2647	ct2919	cn3221	pOP-EO08403_EST_C_1_pSK_SK	302
cl2648	ct2920	cn3222	pOP-CNH03198_EST_C_1_pSK_SK	574
cl2648	ct2920	cn3222	pOP-CNH03633_EST_C_1_pSK_SK	629
cl2648	ct2920	cn3222	pOP-CNH04217	577
cl2648	ct2920	cn3222	pOP-CNH04779_EST_C_1_pSK_SK	561
cl2648	ct2920	cn3222	pOP-CNHP00288_EST_C_1_pSK_SK	390
cl2648	ct2920	cn3222	pOP-EO03421_EST_C_1_pSK_SK	482
cl2648	ct2920	cn3222	pOP-EO08413_EST_C_1_pSK_SK	365
cl2649	ct2921	cn3223	pOP-EO02441_EST_C_1_pSK_SK	467
cl2649	ct2921	cn3223	pOP-EO08417_EST_C_1_pSK_SK	339
cl2650	ct2922	cn3224	pOP-EO08029_EST_C_1_pSK_SK	305
cl2650	ct2922	cn3224	pOP-EO08420_EST_C_1_pSK_SK	513
cl2651	ct2923	cn3225	pOP-EO05560_EST_C_1_pSK_SK	499
cl2651	ct2923	cn3225	pOP-EO08425_EST_C_1_pSK_SK	352
cl2652	ct2924	cn3226	pOP-EN00284_EST_C_1_pSK_SK	570
cl2652	ct2924	cn3226	pOP-EO08432_EST_C_1_pSK_SK	399
cl2653	ct2925	cn3227	pOP-CEO01485_EST_C_1_pSK_SK	237
cl2653	ct2925	cn3227	pOP-CEO01890_EST_C_1_pSK_SK	460
cl2653	ct2925	cn3227	pOP-CEO02695_EST_C_1_pSK_SK	399
cl2653	ct2925	cn3227	pOP-CNH01306_EST_C_1_pSK_SK	596
cl2653	ct2925	cn3227	pOP-CNH01512_EST_C_1_pSK_SK	502
cl2653	ct2925	cn3227	pOP-CNH02352_EST_C_1_pSK_SK	658
cl2653	ct2925	cn3227	pOP-CNH03471_EST_C_1_pSK_SK	350
cl2653	ct2925	cn3227	pOP-EO03681_EST_C_1_pSK_SK	313
cl2653	ct2925	cn3227	pOP-EO07773_EST_C_1_pSK_SK	662
cl2653	ct2925	cn3227	pOP-EO08433_EST_C_1_pSK_SK	173
cl2654	ct2926	cn3228	pOP-CNI01292_EST_C_1_pSK_SK	666
cl2654	ct2926	cn3228	pOP-EO08443_EST_C_1_pSK_SK	159
cl2655	ct2927	cn3229	pOP-CEO01417_EST_C_1_pSK_SK	654
cl2655	ct2927	cn3229	pOP-CNI01234_EST_C_1_pSK_SK	706
cl2655	ct2927	cn3229	pOP-CNIP00772_EST_C_1_pSK_SK	272
cl2655	ct2927	cn3229	pOP-EO06678_EST_C_1_pSK_SK	786
cl2655	ct2927	cn3229	pOP-EO06745_EST_C_1_pSK_SK	913
cl2655	ct2927	cn3229	pOP-EO08295_EST_C_1_pSK_SK	609
cl2655	ct2927	cn3229	pOP-EO08448_EST_C_1_pSK_SK	468

cl2656	ct2928	cn3230	pOP-CNIP00853_EST_C_1_pSK_SK	579
cl2656	ct2928	cn3230	pOP-EAP01517_EST_C_1_pBSK_SK	379
cl2656	ct2928	cn3230	pOP-EAP01518_EST_C_1_pBSK_SK	589
cl2656	ct2928	cn3230	pOP-EAP02747_EST_C_1_pBSK_SK	647
cl2656	ct2928	cn3230	pOP-EO02389_EST_C_1_pSK_SK	674
cl2656	ct2928	cn3230	pOP-EO03304_EST_C_1_pSK_SK	267
cl2656	ct2928	cn3230	pOP-EO03487_EST_C_1_pSK_SK	537
cl2656	ct2928	cn3230	pOP-EO03532_EST_C_1_pSK_SK	452
cl2656	ct2928	cn3230	pOP-EO08453_EST_C_1_pSK_SK	189
cl2657	ct2929	cn3231	pOP-CEO02923_EST_C_1_pSK_SK	194
cl2657	ct2929	cn3231	pOP-CEO03597_EST_C_1_pSK_SK	193
cl2657	ct2929	cn3231	pOP-EO06105_EST_C_1_pSK_SK	489
cl2657	ct2929	cn3231	pOP-EO08455_EST_C_1_pSK_SK	358
cl2658	ct2930	cn3232	pOP-EO04284_EST_C_1_pSK_SK	361
cl2658	ct2930	cn3232	pOP-EO08457_EST_C_1_pSK_SK	424
cl2659	ct2931	cn3233	pOP-EO06379_EST_C_1_pSK_SK	769
cl2659	ct2931	cn3233	pOP-EO08458_EST_C_1_pSK_SK	189
cl2660	ct2932	cn3234	pOP-CBP00046_EST_C_1_pBSK_SK	415
cl2660	ct2932	cn3234	pOP-EO02087_EST_C_1_pSK_SK	480
cl2660	ct2932	cn3234	pOP-EO08460_EST_C_1_pSK_SK	218
cl2661	ct2933	cn3235	pOP-EN00480_EST_C_1_pSK_SK	507
cl2661	ct2933	cn3235	pOP-EO08464_EST_C_1_pSK_SK	334
cl2662	ct2934	cn3236	pOP-CEO03441_EST_C_1_pSK_SK	181
cl2662	ct2934	cn3236	pOP-EO03888_EST_C_1_pSK_SK	484
cl2662	ct2934	cn3236	pOP-EO04968_EST_C_1_pSK_SK	513
cl2662	ct2934	cn3236	pOP-EO08467_EST_C_1_pSK_SK	282
cl2663	ct2935	cn3237	pOP-EO07428_EST_C_1_pSK_SK	672
cl2663	ct2935	cn3237	pOP-EO08469_EST_C_1_pSK_SK	256
cl2664	ct2936	cn3238	pOP-EN00244_EST_C_1_pSK_SK	485
cl2664	ct2936	cn3238	pOP-EN00341_EST_C_1_pSK_SK	564
cl2664	ct2936	cn3238	pOP-EN00569_EST_C_1_pSK_SK	535
cl2664	ct2936	cn3238	pOP-EN00594_EST_C_1_pSK_SK	600
cl2664	ct2936	cn3238	pOP-EN00631_EST_C_1_pSK_SK	433
cl2664	ct2936	cn3238	pOP-EO03345_EST_C_1_pSK_SK	447
cl2664	ct2936	cn3238	pOP-EO05511_EST_C_1_pSK_SK	518
cl2664	ct2936	cn3238	pOP-EO05693_EST_C_1_pSK_SK	488
cl2664	ct2936	cn3238	pOP-EO05733_EST_C_1_pSK_SK	525
cl2664	ct2936	cn3238	pOP-EO06104_EST_C_1_pSK_SK	670
cl2664	ct2936	cn3238	pOP-EO06179_EST_C_1_pSK_SK	654
cl2664	ct2936	cn3238	pOP-EO06365_EST_C_1_pSK_SK	610
cl2664	ct2936	cn3238	pOP-EO06708_EST_C_1_pSK_SK	389
cl2664	ct2936	cn3238	pOP-EO06715_EST_C_1_pSK_SK	755
cl2664	ct2936	cn3238	pOP-EO07049_EST_C_1_pSK_SK	609
cl2664	ct2936	cn3238	pOP-EO07376_EST_C_1_pSK_SK	824
cl2664	ct2936	cn3238	pOP-EO07410_EST_C_1_pSK_SK	778
cl2664	ct2936	cn3238	pOP-EO07541_EST_C_1_pSK_SK	769
cl2664	ct2936	cn3238	pOP-EO07948_EST_C_1_pSK_SK	299
cl2664	ct2936	cn3238	pOP-EO08038_EST_C_1_pSK_SK	392
cl2664	ct2936	cn3238	pOP-EO08310_EST_C_1_pSK_SK	523
cl2664	ct2936	cn3238	pOP-EO08355_EST_C_1_pSK_SK	202
cl2664	ct2936	cn3238	pOP-EO08471_EST_C_1_pSK_SK	307
cl2665	ct2937	cn3239	pOP-EO02008_EST_C_1_pSK_SK	402
cl2665	ct2937	cn3239	pOP-EO02876_EST_C_1_pSK_SK	441
cl2665	ct2937	cn3239	pOP-EO03491_EST_C_1_pSK_SK	538
cl2665	ct2937	cn3239	pOP-EO04390_EST_C_1_pSK_SK	436
cl2665	ct2937	cn3239	pOP-EO04468_EST_C_1_pSK_SK	529

cl2665	ct2937	cn3239	pOP-EO05476_EST_C_1_pSK_SK	521
cl2665	ct2937	cn3239	pOP-EO05775_EST_C_1_pSK_SK	480
cl2665	ct2937	cn3239	pOP-EO06908_EST_C_1_pSK_SK	636
cl2665	ct2937	cn3239	pOP-EO07380_EST_C_1_pSK_SK	732
cl2665	ct2937	cn3239	pOP-EO08343_EST_C_1_pSK_SK	480
cl2665	ct2938	cn3240	pOP-EO02038_EST_C_1_pSK_SK	708
cl2665	ct2938	cn3240	pOP-EO02060_EST_C_1_pSK_SK	601
cl2665	ct2938	cn3240	pOP-EO02660_EST_C_1_pSK_SK	459
cl2665	ct2938	cn3240	pOP-EO04103_EST_C_1_pSK_SK	556
cl2665	ct2938	cn3240	pOP-EO04252_EST_C_1_pSK_SK	346
cl2665	ct2938	cn3240	pOP-EO04705_EST_C_1_pSK_SK	422
cl2665	ct2938	cn3240	pOP-EO05568_EST_C_1_pSK_SK	446
cl2665	ct2938	cn3240	pOP-EO05949_EST_C_1_pSK_SK	508
cl2665	ct2938	cn3240	pOP-EO07225_EST_C_1_pSK_SK	695
cl2665	ct2938	cn3240	pOP-EO07502_EST_C_1_pSK_SK	638
cl2665	ct2938	cn3240	pOP-EO07975_EST_C_1_pSK_SK	342
cl2665	ct2938	cn3240	pOP-EO08244_EST_C_1_pSK_SK	546
cl2665	ct2938	cn3240	pOP-EO08476_EST_C_1_pSK_SK	207
cl2666	ct2939	cn3241	pOP-CAP00207_EST_C_1_pBSK_SK	583
cl2666	ct2939	cn3241	pOP-EO07044_EST_C_1_pSK_SK	670
cl2666	ct2939	cn3241	pOP-EO08478_EST_C_1_pSK_SK	157
cl2667	ct2940	cn3242	pOP-CEO03666_EST_C_1_pSK_SK	192
cl2667	ct2940	cn3242	pOP-CNH00708_EST_C_1_pSK_SK	600
cl2667	ct2940	cn3242	pOP-CNH00800_EST_C_1_pSK_SK	538
cl2667	ct2940	cn3242	pOP-CNH01390_EST_C_1_pSK_SK	754
cl2667	ct2940	cn3242	pOP-CNH02900_EST_C_1_pSK_SK	530
cl2667	ct2940	cn3242	pOP-CNH03117_EST_C_1_pSK_SK	562
cl2667	ct2940	cn3242	pOP-CNH04312	401
cl2667	ct2940	cn3242	pOP-CNH04876_EST_C_1_pSK_SK	552
cl2667	ct2940	cn3242	pOP-CNHP00076_EST_C_1_pSK_SK	558
cl2667	ct2940	cn3242	pOP-CNI01301_EST_C_1_pSK_SK	313
cl2667	ct2940	cn3242	pOP-CNI02122_EST_C_1_pSK_SK	172
cl2667	ct2940	cn3242	pOP-EAP01876_EST_C_1_pBSK_SK	624
cl2667	ct2940	cn3242	pOP-EAP01877_EST_C_1_pBSK_SK	658
cl2667	ct2940	cn3242	pOP-EAP02368_EST_C_1_pBSK_SK	564
cl2667	ct2940	cn3242	pOP-EN00172_EST_C_1_pSK_SK	365
cl2667	ct2940	cn3242	pOP-EN00493_EST_C_1_pSK_SK	514
cl2667	ct2940	cn3242	pOP-EO05971_EST_C_1_pSK_SK	557
cl2667	ct2940	cn3242	pOP-EO06298_EST_C_1_pSK_SK	601
cl2667	ct2940	cn3242	pOP-EO08483_EST_C_1_pSK_SK	153
cl2668	ct2941	cn3243	pOP-EO05445_EST_C_1_pSK_SK	402
cl2668	ct2941	cn3243	pOP-EO07132_EST_C_1_pSK_SK	614
cl2668	ct2941	cn3243	pOP-EO08495_EST_C_1_pSK_SK	301
cl2669	ct2942	cn3244	pOP-CEO02503_EST_C_1_pSK_SK	240
cl2669	ct2942	cn3244	pOP-CEO02504_EST_C_1_pSK_SK	251
cl2669	ct2942	cn3244	pOP-EO08503_EST_C_1_pSK_SK	172
cl2670	ct2943	cn3245	pOP-EO07465_EST_C_1_pSK_SK	785
cl2670	ct2943	cn3245	pOP-EO07619_EST_C_1_pSK_SK	660
cl2670	ct2943	cn3245	pOP-EO08511_EST_C_1_pSK_SK	329
cl2671	ct2944	cn3246	pOP-EN00291_EST_C_1_pSK_SK	488
cl2671	ct2944	cn3246	pOP-EO02611_EST_C_1_pSK_SK	374
cl2671	ct2944	cn3246	pOP-EO03393_EST_C_1_pSK_SK	461
cl2671	ct2944	cn3246	pOP-EO04897_EST_C_1_pSK_SK	516
cl2671	ct2944	cn3246	pOP-EO08156_EST_C_1_pSK_SK	422
cl2671	ct2944	cn3246	pOP-EO08512_EST_C_1_pSK_SK	262
cl2671	ct2944	cn3247	pOP-EN00845_EST_C_1_pSK_SK	373

cl2672	ct2945	cn3248	pOP-EO03486_EST_C_1_pSK_SK	533
cl2672	ct2945	cn3248	pOP-EO08514_EST_C_1_pSK_SK	307
cl2673	ct2946	cn3249	pOP-EO02092_EST_C_1_pSK_SK	357
cl2673	ct2946	cn3249	pOP-EO06300_EST_C_1_pSK_SK	346
cl2673	ct2946	cn3249	pOP-EO08520_EST_C_1_pSK_SK	278
cl2674	ct2947	cn3250	pOP-CEO01888_EST_C_1_pSK_SK	317
cl2674	ct2947	cn3250	pOP-EO03902_EST_C_1_pSK_SK	445
cl2674	ct2947	cn3250	pOP-EO08521_EST_C_1_pSK_SK	338
cl2675	ct2948	cn3251	pOP-CEO00878_EST_C_1_pSK_SK	175
cl2675	ct2948	cn3251	pOP-CNI01475_EST_C_1_pSK_SK	400
cl2675	ct2948	cn3251	pOP-CNI02156_EST_C_1_pSK_SK	262
cl2675	ct2948	cn3251	pOP-EO06854_EST_C_1_pSK_SK	767
cl2675	ct2948	cn3251	pOP-EO08522_EST_C_1_pSK_SK	196
cl2676	ct2949	cn3252	pOP-CNI01434_EST_C_1_pSK_SK	182
cl2676	ct2949	cn3252	pOP-EAP01025_EST_C_1_pBSK_SK	274
cl2676	ct2949	cn3252	pOP-EAP01359_EST_C_1_pBSK_SK	322
cl2676	ct2949	cn3252	pOP-EAP02064_EST_C_1_pBSK_SK	271
cl2676	ct2949	cn3252	pOP-EO06401_EST_C_1_pSK_SK	792
cl2676	ct2949	cn3252	pOP-EO06918_EST_C_1_pSK_SK	358
cl2676	ct2949	cn3252	pOP-EO07579_EST_C_1_pSK_SK	632
cl2676	ct2949	cn3252	pOP-EO07905_EST_C_1_pSK_SK	694
cl2676	ct2949	cn3252	pOP-EO08529_EST_C_1_pSK_SK	235
cl2676	ct2949	cn3253	pOP-EO07194_EST_C_1_pSK_SK	652
cl2677	ct2950	cn3254	pOP-EN00421_EST_C_1_pSK_SK	503
cl2677	ct2950	cn3254	pOP-EO06911_EST_C_1_pSK_SK	674
cl2677	ct2950	cn3254	pOP-EO08533_EST_C_1_pSK_SK	250
cl2678	ct2951	cn3255	pOP-CNH04588	661
cl2678	ct2951	cn3255	pOP-EN00625_EST_C_1_pSK_SK	408
cl2678	ct2951	cn3255	pOP-EO03688_EST_C_1_pSK_SK	359
cl2678	ct2951	cn3255	pOP-EOP00001_EST_C_1_pSK_SK	584
cl2679	ct2952	cn3256	pOP-CBP00181_EST_C_1_pBSK_SK	633
cl2679	ct2952	cn3256	pOP-CNI01098_EST_C_1_pSK_SK	412
cl2679	ct2952	cn3256	pOP-EO04824_EST_C_1_pSK_SK	530
cl2679	ct2952	cn3256	pOP-EO04928_EST_C_1_pSK_SK	470
cl2679	ct2952	cn3256	pOP-EO04951_EST_C_1_pSK_SK	473
cl2679	ct2953	cn3257	pOP-CEO01127_EST_C_1_pSK_SK	124
cl2679	ct2953	cn3257	pOP-CEO01740_EST_C_1_pSK_SK	143
cl2679	ct2953	cn3257	pOP-CEO03458_EST_C_1_pSK_SK	116
cl2679	ct2953	cn3257	pOP-EO06087_EST_C_1_pSK_SK	390
cl2679	ct2953	cn3257	pOP-EO06205_EST_C_1_pSK_SK	470
cl2679	ct2953	cn3257	pOP-EO06380_EST_C_1_pSK_SK	609
cl2679	ct2953	cn3257	pOP-EO06569_EST_C_1_pSK_SK	604
cl2679	ct2953	cn3257	pOP-EO07735_EST_C_1_pSK_SK	451
cl2679	ct2953	cn3257	pOP-EO08140_EST_C_1_pSK_SK	586
cl2679	ct2953	cn3257	pOP-EO08233_EST_C_1_pSK_SK	524
cl2679	ct2953	cn3257	pOP-EOP00003_EST_C_1_pSK_SK	600
cl2680	ct2954	cn3258	pOP-CEO02545_EST_C_1_pSK_SK	357
cl2680	ct2954	cn3258	pOP-CNI01767_EST_C_1_pSK_SK	537
cl2680	ct2954	cn3258	pOP-EAP00596_EST_C_1_pBSK_SK	634
cl2680	ct2954	cn3258	pOP-EAP00845_EST_C_1_pBSK_SK	593
cl2680	ct2954	cn3258	pOP-EAP01831_EST_C_1_pBSK_SK	533
cl2680	ct2954	cn3258	pOP-EAP03805_EST_C_1_pBSK_SK	745
cl2680	ct2954	cn3259	pOP-EOP00004_EST_C_1_pSK_SK	670
cl2680	ct2954	cn3260	pOP-EO06226_EST_C_1_pSK_SK	662
cl2680	ct2954	cn3261	pOP-EAP01563_EST_C_1_pBSK_SK	571
cl2681	ct2955	cn3262	pOP-EAP02768_EST_C_1_pBSK_SK	539

cl2681	ct2955	cn3262	pOP-EOP00010_EST_C_1_pSK_SK	506
cl2682	ct2956	cn3263	pOP-CNH02923_EST_C_1_pSK_SK	656
cl2682	ct2956	cn3263	pOP-EO03541_EST_C_1_pSK_SK	501
cl2682	ct2956	cn3263	pOP-EOP00011_EST_C_1_pSK_SK	658
cl2683	ct2957	cn3264	pOP-CEO03529_EST_C_1_pSK_SK	150
cl2683	ct2957	cn3264	pOP-EO06482_EST_C_1_pSK_SK	732
cl2683	ct2957	cn3264	pOP-EOP00013_EST_C_1_pSK_SK	645
cl2684	ct2958	cn3265	pOP-CEO01520_EST_C_1_pSK_SK	379
cl2684	ct2958	cn3265	pOP-EO03922_EST_C_1_pSK_SK	539
cl2684	ct2958	cn3265	pOP-EO05647_EST_C_1_pSK_SK	500
cl2684	ct2958	cn3265	pOP-EOP00014_EST_C_1_pSK_SK	609
cl2685	ct2959	cn3266	pOP-CAP00099_EST_C_1_pBSK_SK	669
cl2685	ct2959	cn3266	pOP-CAP00411_EST_C_1_pBSK_SK	649
cl2685	ct2959	cn3266	pOP-EAP03150_EST_C_1_pBSK_SK	520
cl2685	ct2959	cn3266	pOP-EO04377_EST_C_1_pSK_SK	457
cl2685	ct2959	cn3266	pOP-EO05525_EST_C_1_pSK_SK	468
cl2685	ct2960	cn3267	pOP-CEO01038_EST_C_1_pSK_SK	517
cl2685	ct2960	cn3267	pOP-CEO01637_EST_C_1_pSK_SK	187
cl2685	ct2960	cn3267	pOP-CEO01827_EST_C_1_pSK_SK	204
cl2685	ct2960	cn3267	pOP-EAP00125_EST_C_1_pBSK_SK	529
cl2685	ct2960	cn3267	pOP-EO02742_EST_C_1_pSK_SK	411
cl2685	ct2960	cn3267	pOP-EOP00015_EST_C_1_pSK_SK	297
cl2686	ct2961	cn3268	pOP-CEO00545_EST_C_1_pSK_SK	348
cl2686	ct2961	cn3268	pOP-CEO00563_EST_C_1_pSK_SK	323
cl2686	ct2961	cn3268	pOP-CEO00922_EST_C_1_pSK_SK	237
cl2686	ct2961	cn3268	pOP-CEO01598_EST_C_1_pSK_SK	350
cl2686	ct2961	cn3268	pOP-CEO01604_EST_C_1_pSK_SK	350
cl2686	ct2961	cn3268	pOP-CEO01636_EST_C_1_pSK_SK	310
cl2686	ct2961	cn3268	pOP-CEO01651_EST_C_1_pSK_SK	331
cl2686	ct2961	cn3268	pOP-CEO01680_EST_C_1_pSK_SK	350
cl2686	ct2961	cn3268	pOP-CEO01875_EST_C_1_pSK_SK	344
cl2686	ct2961	cn3268	pOP-CEO02840_EST_C_1_pSK_SK	352
cl2686	ct2961	cn3268	pOP-CEO03026_EST_C_1_pSK_SK	320
cl2686	ct2961	cn3268	pOP-CEO03030_EST_C_1_pSK_SK	322
cl2686	ct2961	cn3268	pOP-CEO03137_EST_C_1_pSK_SK	352
cl2686	ct2961	cn3268	pOP-CEO03152_EST_C_1_pSK_SK	331
cl2686	ct2961	cn3268	pOP-CEO03305_EST_C_1_pSK_SK	335
cl2686	ct2961	cn3268	pOP-CEO03331_EST_C_1_pSK_SK	323
cl2686	ct2961	cn3268	pOP-CEO03361_EST_C_1_pSK_SK	258
cl2686	ct2961	cn3268	pOP-CEO03422_EST_C_1_pSK_SK	285
cl2686	ct2961	cn3268	pOP-CEO03492_EST_C_1_pSK_SK	348
cl2686	ct2961	cn3268	pOP-CEO03571_EST_C_1_pSK_SK	352
cl2686	ct2961	cn3268	pOP-CEO03640_EST_C_1_pSK_SK	352
cl2686	ct2961	cn3268	pOP-CEO03687_EST_C_1_pSK_SK	356
cl2686	ct2961	cn3268	pOP-CEO03762_EST_C_1_pSK_SK	348
cl2686	ct2961	cn3268	pOP-CNI01252_EST_C_1_pSK_SK	342
cl2686	ct2961	cn3268	pOP-CNI01500_EST_C_1_pSK_SK	350
cl2686	ct2961	cn3268	pOP-CNI01987_EST_C_1_pSK_SK	297
cl2686	ct2961	cn3268	pOP-CNIP00128_EST_C_1_pSK_SK	336
cl2686	ct2961	cn3268	pOP-CNIP00459_EST_C_1_pSK_SK	234
cl2686	ct2961	cn3268	pOP-EAP00579_EST_C_1_pBSK_SK	331
cl2686	ct2961	cn3268	pOP-EAP01184_EST_C_1_pBSK_SK	296
cl2686	ct2961	cn3268	pOP-EAP01273_EST_C_1_pBSK_SK	323
cl2686	ct2961	cn3268	pOP-EAP03613_EST_C_1_pBSK_SK	350
cl2686	ct2961	cn3268	pOP-EO03266_EST_C_1_pSK_SK	352
cl2686	ct2961	cn3268	pOP-EO03740_EST_C_1_pSK_SK	308

cl2686	ct2961	cn3268	pOP-EO03900_EST_C_1_pSK_SK	325
cl2686	ct2961	cn3268	pOP-EO03970_EST_C_1_pSK_SK	352
cl2686	ct2961	cn3268	pOP-EO03976_EST_C_1_pSK_SK	352
cl2686	ct2961	cn3268	pOP-EO04119_EST_C_1_pSK_SK	352
cl2686	ct2961	cn3268	pOP-EO04131_EST_C_1_pSK_SK	352
cl2686	ct2961	cn3268	pOP-EO04203_EST_C_1_pSK_SK	318
cl2686	ct2961	cn3268	pOP-EO06616_EST_C_1_pSK_SK	352
cl2686	ct2961	cn3268	pOP-EO06690_EST_C_1_pSK_SK	352
cl2686	ct2961	cn3268	pOP-EO06733_EST_C_1_pSK_SK	352
cl2686	ct2961	cn3268	pOP-EO06807_EST_C_1_pSK_SK	352
cl2686	ct2961	cn3268	pOP-EO06902_EST_C_1_pSK_SK	697
cl2686	ct2961	cn3268	pOP-EO06969_EST_C_1_pSK_SK	352
cl2686	ct2961	cn3268	pOP-EO07087_EST_C_1_pSK_SK	352
cl2686	ct2961	cn3268	pOP-EO07104_EST_C_1_pSK_SK	352
cl2686	ct2961	cn3268	pOP-EO07110_EST_C_1_pSK_SK	352
cl2686	ct2961	cn3268	pOP-EO07165_EST_C_1_pSK_SK	342
cl2686	ct2961	cn3268	pOP-EO07983_EST_C_1_pSK_SK	322
cl2686	ct2961	cn3268	pOP-EO08394_EST_C_1_pSK_SK	203
cl2686	ct2961	cn3268	pOP-EOP00016_EST_C_1_pSK_SK	334
cl2687	ct2962	cn3269	pOP-EAP03158_EST_C_1_pBSK_SK	468
cl2687	ct2962	cn3269	pOP-EO02355_EST_C_1_pSK_SK	559
cl2687	ct2962	cn3269	pOP-EO03457_EST_C_1_pSK_SK	532
cl2687	ct2962	cn3269	pOP-EO06172_EST_C_1_pSK_SK	441
cl2687	ct2962	cn3269	pOP-EO07463_EST_C_1_pSK_SK	670
cl2687	ct2962	cn3269	pOP-EO08213_EST_C_1_pSK_SK	564
cl2687	ct2962	cn3269	pOP-EOP00020_EST_C_1_pSK_SK	577
cl2688	ct2963	cn3270	pOP-CBP00010_EST_C_1_pBSK_SK	640
cl2688	ct2963	cn3270	pOP-CNHP00112_EST_C_1_pSK_SK	490
cl2688	ct2963	cn3270	pOP-CNI01460_EST_C_1_pSK_SK	341
cl2688	ct2963	cn3270	pOP-EO04478_EST_C_1_pSK_SK	474
cl2688	ct2963	cn3270	pOP-EOP00021_EST_C_1_pSK_SK	346
			pOP-CAP00005_EST_C_1_pBSK_SK	660
			pOP-CAP00008_EST_C_1_pBSK_SK	607
			pOP-CAP00014_EST_C_1_pBSK_SK	651
			pOP-CAP00016_EST_C_1_pBSK_SK	563
			pOP-CAP00017_EST_C_1_pBSK_SK	646
			pOP-CAP00023_EST_C_1_pBSK_SK	391
			pOP-CAP00026_EST_C_1_pBSK_SK	398
			pOP-CAP00027_EST_C_1_pBSK_SK	516
			pOP-CAP00031_EST_C_1_pBSK_SK	524
			pOP-CAP00032_EST_C_1_pBSK_SK	603
			pOP-CAP00035_EST_C_1_pBSK_SK	541
			pOP-CAP00036_EST_C_1_pBSK_SK	452
			pOP-CAP00037_EST_C_1_pBSK_SK	360
			pOP-CAP00043_EST_C_1_pBSK_SK	481
			pOP-CAP00049_EST_C_1_pBSK_SK	373
			pOP-CAP00053_EST_C_1_pBSK_SK	617
			pOP-CAP00054_EST_C_1_pBSK_SK	543
			pOP-CAP00055_EST_C_1_pBSK_SK	622
			pOP-CAP00058_EST_C_1_pBSK_SK	650
			pOP-CAP00059_EST_C_1_pBSK_SK	592
			pOP-CAP00060_EST_C_1_pBSK_SK	464
			pOP-CAP00062_EST_C_1_pBSK_SK	552
			pOP-CAP00071_EST_C_1_pBSK_SK	643
			pOP-CAP00073_EST_C_1_pBSK_SK	671
			pOP-CAP00090_EST_C_1_pBSK_SK	651

			pOP-CAP00107_EST_C_1_pBSK_SK	646
			pOP-CAP00109_EST_C_1_pBSK_SK	620
			pOP-CAP00112_EST_C_1_pBSK_SK	654
			pOP-CAP00113_EST_C_1_pBSK_SK	655
			pOP-CAP00136_EST_C_1_pBSK_SK	663
			pOP-CAP00140_EST_C_1_pBSK_SK	587
			pOP-CAP00148_EST_C_1_pBSK_SK	211
			pOP-CAP00161_EST_C_1_pBSK_SK	623
			pOP-CAP00168_EST_C_1_pBSK_SK	670
			pOP-CAP00179_EST_C_1_pBSK_SK	644
			pOP-CAP00184_EST_C_1_pBSK_SK	647
			pOP-CAP00188_EST_C_1_pBSK_SK	649
			pOP-CAP00194_EST_C_1_pBSK_SK	654
			pOP-CAP00197_EST_C_1_pBSK_SK	654
			pOP-CAP00199_EST_C_1_pBSK_SK	259
			pOP-CAP00200_EST_C_1_pBSK_SK	526
			pOP-CAP00201_EST_C_1_pBSK_SK	542
			pOP-CAP00203_EST_C_1_pBSK_SK	474
			pOP-CAP00215_EST_C_1_pBSK_SK	600
			pOP-CAP00218_EST_C_1_pBSK_SK	614
			pOP-CAP00220_EST_C_1_pBSK_SK	385
			pOP-CAP00222_EST_C_1_pBSK_SK	634
			pOP-CAP00223_EST_C_1_pBSK_SK	641
			pOP-CAP00227_EST_C_1_pBSK_SK	573
			pOP-CAP00233_EST_C_1_pBSK_SK	557
			pOP-CAP00236_EST_C_1_pBSK_SK	464
			pOP-CAP00238_EST_C_1_pBSK_SK	551
			pOP-CAP00241_EST_C_1_pBSK_SK	638
			pOP-CAP00243_EST_C_1_pBSK_SK	589
			pOP-CAP00244_EST_C_1_pBSK_SK	368
			pOP-CAP00245_EST_C_1_pBSK_SK	580
			pOP-CAP00246_EST_C_1_pBSK_SK	534
			pOP-CAP00249_EST_C_1_pBSK_SK	610
			pOP-CAP00252_EST_C_1_pBSK_SK	580
			pOP-CAP00253_EST_C_1_pBSK_SK	553
			pOP-CAP00254_EST_C_1_pBSK_SK	517
			pOP-CAP00259_EST_C_1_pBSK_SK	349
			pOP-CAP00264_EST_C_1_pBSK_SK	650
			pOP-CAP00265_EST_C_1_pBSK_SK	616
			pOP-CAP00267_EST_C_1_pBSK_SK	315
			pOP-CAP00268_EST_C_1_pBSK_SK	584
			pOP-CAP00270_EST_C_1_pBSK_SK	516
			pOP-CAP00272_EST_C_1_pBSK_SK	521
			pOP-CAP00274_EST_C_1_pBSK_SK	659
			pOP-CAP00276_EST_C_1_pBSK_SK	686
			pOP-CAP00278_EST_C_1_pBSK_SK	418
			pOP-CAP00279_EST_C_1_pBSK_SK	539
			pOP-CAP00281_EST_C_1_pBSK_SK	691
			pOP-CAP00283_EST_C_1_pBSK_SK	700
			pOP-CAP00289_EST_C_1_pBSK_SK	652
			pOP-CAP00290_EST_C_1_pBSK_SK	631
			pOP-CAP00293_EST_C_1_pBSK_SK	647
			pOP-CAP00295_EST_C_1_pBSK_SK	609
			pOP-CAP00297_EST_C_1_pBSK_SK	363
			pOP-CAP00300_EST_C_1_pBSK_SK	520
			pOP-CAP00303_EST_C_1_pBSK_SK	442

			pOP-CAP00310_EST_C_1_pBSK_SK	397
			pOP-CAP00312_EST_C_1_pBSK_SK	571
			pOP-CAP00315_EST_C_1_pBSK_SK	439
			pOP-CAP00319_EST_C_1_pBSK_SK	610
			pOP-CAP00323_EST_C_1_pBSK_SK	577
			pOP-CAP00337_EST_C_1_pBSK_SK	644
			pOP-CAP00338_EST_C_1_pBSK_SK	639
			pOP-CAP00343_EST_C_1_pBSK_SK	270
			pOP-CAP00348_EST_C_1_pBSK_SK	620
			pOP-CAP00350_EST_C_1_pBSK_SK	438
			pOP-CAP00352_EST_C_1_pBSK_SK	571
			pOP-CAP00357_EST_C_1_pBSK_SK	616
			pOP-CAP00361_EST_C_1_pBSK_SK	414
			pOP-CAP00365_EST_C_1_pBSK_SK	114
			pOP-CAP00367_EST_C_1_pBSK_SK	600
			pOP-CAP00368_EST_C_1_pBSK_SK	481
			pOP-CAP00375_EST_C_1_pBSK_SK	668
			pOP-CAP00391_EST_C_1_pBSK_SK	643
			pOP-CAP00401_EST_C_1_pBSK_SK	613
			pOP-CAP00403_EST_C_1_pBSK_SK	630
			pOP-CAP00407_EST_C_1_pBSK_SK	636
			pOP-CAP00412_EST_C_1_pBSK_SK	618
			pOP-CAP00420_EST_C_1_pBSK_SK	605
			pOP-CAP05007_EST_C_1_pBSK_SK	590
			pOP-CAP05009_EST_C_1_pBSK_SK	556
			pOP-CAP05010_EST_C_1_pBSK_SK	618
			pOP-CBP00003_EST_C_1_pBSK_SK	702
			pOP-CBP00004_EST_C_1_pBSK_SK	501
			pOP-CBP00009_EST_C_1_pBSK_SK	621
			pOP-CBP00014_EST_C_1_pBSK_SK	553
			pOP-CBP00016_EST_C_1_pBSK_SK	487
			pOP-CBP00018_EST_C_1_pBSK_SK	680
			pOP-CBP00019_EST_C_1_pBSK_SK	328
			pOP-CBP00023_EST_C_1_pBSK_SK	521
			pOP-CBP00028_EST_C_1_pBSK_SK	616
			pOP-CBP00034_EST_C_1_pBSK_SK	366
			pOP-CBP00038_EST_C_1_pBSK_SK	339
			pOP-CBP00048_EST_C_1_pBSK_SK	615
			pOP-CBP00053_EST_C_1_pBSK_SK	443
			pOP-CBP00057_EST_C_1_pBSK_SK	272
			pOP-CBP00060_EST_C_1_pBSK_SK	242
			pOP-CBP00062_EST_C_1_pBSK_SK	613
			pOP-CBP00066_EST_C_1_pBSK_SK	437
			pOP-CBP00067_EST_C_1_pBSK_SK	279
			pOP-CBP00071_EST_C_1_pBSK_SK	239
			pOP-CBP00075_EST_C_1_pBSK_SK	440
			pOP-CBP00077_EST_C_1_pBSK_SK	130
			pOP-CBP00079_EST_C_1_pBSK_SK	466
			pOP-CBP00081_EST_C_1_pBSK_SK	248
			pOP-CBP00085_EST_C_1_pBSK_SK	490
			pOP-CBP00088_EST_C_1_pBSK_SK	458
			pOP-CBP00091_EST_C_1_pBSK_SK	351
			pOP-CBP00094_EST_C_1_pBSK_SK	156
			pOP-CBP00100_EST_C_1_pBSK_SK	449
			pOP-CBP00105_EST_C_1_pBSK_SK	398
			pOP-CBP00109_EST_C_1_pBSK_SK	653

			pOP-CBP00112_EST_C_1_pBSK_SK	657
			pOP-CBP00113_EST_C_1_pBSK_SK	637
			pOP-CBP00114_EST_C_1_pBSK_SK	650
			pOP-CBP00135_EST_C_1_pBSK_SK	614
			pOP-CBP00138_EST_C_1_pBSK_SK	612
			pOP-CBP00140_EST_C_1_pBSK_SK	564
			pOP-CBP00143_EST_C_1_pBSK_SK	538
			pOP-CBP00144_EST_C_1_pBSK_SK	650
			pOP-CBP00147_EST_C_1_pBSK_SK	655
			pOP-CBP00151_EST_C_1_pBSK_SK	681
			pOP-CBP00155_EST_C_1_pBSK_SK	497
			pOP-CBP00156_EST_C_1_pBSK_SK	589
			pOP-CBP00157_EST_C_1_pBSK_SK	414
			pOP-CBP00160_EST_C_1_pBSK_SK	483
			pOP-CBP00161_EST_C_1_pBSK_SK	403
			pOP-CBP00162_EST_C_1_pBSK_SK	549
			pOP-CBP00169_EST_C_1_pBSK_SK	353
			pOP-CBP00171_EST_C_1_pBSK_SK	230
			pOP-CBP00173_EST_C_1_pBSK_SK	590
			pOP-CBP00177_EST_C_1_pBSK_SK	389
			pOP-CBP00179_EST_C_1_pBSK_SK	215
			pOP-CBP00189_EST_C_1_pBSK_SK	523
			pOP-CBP00196_EST_C_1_pBSK_SK	273
			pOP-CBP00199_EST_C_1_pBSK_SK	427
			pOP-CBP00208_EST_C_1_pBSK_SK	233
			pOP-CBP00209_EST_C_1_pBSK_SK	482
			pOP-CBP00212_EST_C_1_pBSK_SK	345
			pOP-CBP00213_EST_C_1_pBSK_SK	377
			pOP-CBP00215_EST_C_1_pBSK_SK	195
			pOP-CBP00238_EST_C_1_pBSK_SK	523
			pOP-CBP00240_EST_C_1_pBSK_SK	624
			pOP-CBP00242_EST_C_1_pBSK_SK	548
			pOP-CBP00246_EST_C_1_pBSK_SK	186
			pOP-CBP00250_EST_C_1_pBSK_SK	529
			pOP-CBP00251_EST_C_1_pBSK_SK	455
			pOP-CBP00253_EST_C_1_pBSK_SK	411
			pOP-CBP00254_EST_C_1_pBSK_SK	506
			pOP-CBP00257_EST_C_1_pBSK_SK	217
			pOP-CBP00258_EST_C_1_pBSK_SK	596
			pOP-CBP00259_EST_C_1_pBSK_SK	399
			pOP-CBP00269_EST_C_1_pBSK_SK	465
			pOP-CCP00001_EST_C_1_pBSK_SK	385
			pOP-CCP00009_EST_C_1_pBSK_SK	343
			pOP-CCP00011_EST_C_1_pBSK_SK	191
			pOP-CEM00039_EST_C_1_pSK_SK	334
			pOP-CEM00044_EST_C_1_pSK_SK	528
			pOP-CEM00047_EST_C_1_pSK_SK	249
			pOP-CEM00056_EST_C_1_pSK_SK	436
			pOP-CEM00058_EST_C_1_pSK_SK	166
			pOP-CEM00060_EST_C_1_pSK_SK	108
			pOP-CEM00070_EST_C_1_pSK_SK	277
			pOP-CEM00076_EST_C_1_pSK_SK	252
			pOP-CEM00077_EST_C_1_pSK_SK	291
			pOP-CEM00079_EST_C_1_pSK_SK	504
			pOP-CEM00083_EST_C_1_pSK_SK	217
			pOP-CEM00084_EST_C_1_pSK_SK	201

			pOP-CEM00090_EST_C_1_pSK_SK	476
			pOP-CEM00095_EST_C_1_pSK_SK	315
			pOP-CEM00100_EST_C_1_pSK_SK	184
			pOP-CEM00102_EST_C_1_pSK_SK	487
			pOP-CEM00105_EST_C_1_pSK_SK	169
			pOP-CEM00106_EST_C_1_pSK_SK	446
			pOP-CEM00109_EST_C_1_pSK_SK	182
			pOP-CEM00113_EST_C_1_pSK_SK	220
			pOP-CEM00115_EST_C_1_pSK_SK	335
			pOP-CEM00118_EST_C_1_pSK_SK	722
			pOP-CEM00119_EST_C_1_pSK_SK	227
			pOP-CEM00124_EST_C_1_pSK_SK	227
			pOP-CEM00129_EST_C_1_pSK_SK	382
			pOP-CEM00137_EST_C_1_pSK_SK	109
			pOP-CEM00141_EST_C_1_pSK_SK	705
			pOP-CEM00145_EST_C_1_pSK_SK	595
			pOP-CEM00146_EST_C_1_pSK_SK	466
			pOP-CEM00152_EST_C_1_pSK_SK	149
			pOP-CEM00154_EST_C_1_pSK_SK	374
			pOP-CEM00155_EST_C_1_pSK_SK	308
			pOP-CEM00157_EST_C_1_pSK_SK	466
			pOP-CEM00158_EST_C_1_pSK_SK	136
			pOP-CEM00159_EST_C_1_pSK_SK	176
			pOP-CEM00160_EST_C_1_pSK_SK	272
			pOP-CEM00164_EST_C_1_pSK_SK	584
			pOP-CEM00165_EST_C_1_pSK_SK	175
			pOP-CEM00173_EST_C_1_pSK_SK	358
			pOP-CEM00176_EST_C_1_pSK_SK	293
			pOP-CEM00177_EST_C_1_pSK_SK	469
			pOP-CEM00178_EST_C_1_pSK_SK	206
			pOP-CEM00180_EST_C_1_pSK_SK	353
			pOP-CEM00182_EST_C_1_pSK_SK	186
			pOP-CEM00183_EST_C_1_pSK_SK	398
			pOP-CEM00184_EST_C_1_pSK_SK	395
			pOP-CEM00193_EST_C_1_pSK_SK	378
			pOP-CEM00195_EST_C_1_pSK_SK	296
			pOP-CEM00198_EST_C_1_pSK_SK	230
			pOP-CEM00203_EST_C_1_pSK_SK	481
			pOP-CEM00205_EST_C_1_pSK_SK	523
			pOP-CEM00207_EST_C_1_pSK_SK	166
			pOP-CEM00208_EST_C_1_pSK_SK	227
			pOP-CEM00217_EST_C_1_pSK_SK	167
			pOP-CEM00224_EST_C_1_pSK_SK	116
			pOP-CEM00227_EST_C_1_pSK_SK	744
			pOP-CEM00230_EST_C_1_pSK_SK	391
			pOP-CEM00236_EST_C_1_pSK_SK	271
			pOP-CEM00237_EST_C_1_pSK_SK	186
			pOP-CEM00240_EST_C_1_pSK_SK	206
			pOP-CEM00242_EST_C_1_pSK_SK	293
			pOP-CEM00243_EST_C_1_pSK_SK	254
			pOP-CEM00244_EST_C_1_pSK_SK	633
			pOP-CEM00247_EST_C_1_pSK_SK	510
			pOP-CEM00250_EST_C_1_pSK_SK	411
			pOP-CEM00253_EST_C_1_pSK_SK	203
			pOP-CEM00254_EST_C_1_pSK_SK	155
			pOP-CEM00256_EST_C_1_pSK_SK	179

			pOP-CEM00257_EST_C_1_pSK_SK	147
			pOP-CEMP00003_EST_C_1_pSK_SK	500
			pOP-CEMP00004_EST_C_1_pSK_SK	137
			pOP-CEMP00013_EST_C_1_pSK_SK	351
			pOP-CEMP00014_EST_C_1_pSK_SK	349
			pOP-CEMP00018_EST_C_1_pSK_SK	553
			pOP-CEMP00021_EST_C_1_pSK_SK	163
			pOP-CEMP00025_EST_C_1_pSK_SK	101
			pOP-CEO00505_EST_C_1_pSK_SK	227
			pOP-CEO00506_EST_C_1_pSK_SK	233
			pOP-CEO00508_EST_C_1_pSK_SK	214
			pOP-CEO00512_EST_C_1_pSK_SK	309
			pOP-CEO00516_EST_C_1_pSK_SK	146
			pOP-CEO00521_EST_C_1_pSK_SK	461
			pOP-CEO00528_EST_C_1_pSK_SK	408
			pOP-CEO00529_EST_C_1_pSK_SK	267
			pOP-CEO00530_EST_C_1_pSK_SK	161
			pOP-CEO00550_EST_C_1_pSK_SK	138
			pOP-CEO00554_EST_C_1_pSK_SK	141
			pOP-CEO00555_EST_C_1_pSK_SK	403
			pOP-CEO00556_EST_C_1_pSK_SK	323
			pOP-CEO00557_EST_C_1_pSK_SK	654
			pOP-CEO00558_EST_C_1_pSK_SK	265
			pOP-CEO00559_EST_C_1_pSK_SK	112
			pOP-CEO00560_EST_C_1_pSK_SK	223
			pOP-CEO00561_EST_C_1_pSK_SK	138
			pOP-CEO00562_EST_C_1_pSK_SK	132
			pOP-CEO00572_EST_C_1_pSK_SK	657
			pOP-CEO00574_EST_C_1_pSK_SK	125
			pOP-CEO00578_EST_C_1_pSK_SK	108
			pOP-CEO00579_EST_C_1_pSK_SK	591
			pOP-CEO00580_EST_C_1_pSK_SK	168
			pOP-CEO00581_EST_C_1_pSK_SK	138
			pOP-CEO00584_EST_C_1_pSK_SK	300
			pOP-CEO00585_EST_C_1_pSK_SK	588
			pOP-CEO00589_EST_C_1_pSK_SK	243
			pOP-CEO00593_EST_C_1_pSK_SK	448
			pOP-CEO00605_EST_C_1_pSK_SK	389
			pOP-CEO00641_EST_C_1_pSK_SK	324
			pOP-CEO00649_EST_C_1_pSK_SK	498
			pOP-CEO00661_EST_C_1_pSK_SK	122
			pOP-CEO00662_EST_C_1_pSK_SK	358
			pOP-CEO00663_EST_C_1_pSK_SK	298
			pOP-CEO00667_EST_C_1_pSK_SK	412
			pOP-CEO00668_EST_C_1_pSK_SK	364
			pOP-CEO00669_EST_C_1_pSK_SK	347
			pOP-CEO00676_EST_C_1_pSK_SK	280
			pOP-CEO00679_EST_C_1_pSK_SK	441
			pOP-CEO00680_EST_C_1_pSK_SK	551
			pOP-CEO00684_EST_C_1_pSK_SK	478
			pOP-CEO00687_EST_C_1_pSK_SK	435
			pOP-CEO00697_EST_C_1_pSK_SK	726
			pOP-CEO00699_EST_C_1_pSK_SK	156
			pOP-CEO00700_EST_C_1_pSK_SK	586
			pOP-CEO00702_EST_C_1_pSK_SK	528
			pOP-CEO00703_EST_C_1_pSK_SK	433

			pOP-CEO00707_EST_C_1_pSK_SK	494
			pOP-CEO00710_EST_C_1_pSK_SK	307
			pOP-CEO00715_EST_C_1_pSK_SK	288
			pOP-CEO00720_EST_C_1_pSK_SK	144
			pOP-CEO00724_EST_C_1_pSK_SK	486
			pOP-CEO00745_EST_C_1_pSK_SK	344
			pOP-CEO00761_EST_C_1_pSK_SK	374
			pOP-CEO00763_EST_C_1_pSK_SK	371
			pOP-CEO00764_EST_C_1_pSK_SK	373
			pOP-CEO00765_EST_C_1_pSK_SK	373
			pOP-CEO00772_EST_C_1_pSK_SK	194
			pOP-CEO00789_EST_C_1_pSK_SK	370
			pOP-CEO00815_EST_C_1_pSK_SK	522
			pOP-CEO00816_EST_C_1_pSK_SK	537
			pOP-CEO00820_EST_C_1_pSK_SK	303
			pOP-CEO00822_EST_C_1_pSK_SK	385
			pOP-CEO00824_EST_C_1_pSK_SK	317
			pOP-CEO00825_EST_C_1_pSK_SK	294
			pOP-CEO00830_EST_C_1_pSK_SK	114
			pOP-CEO00831_EST_C_1_pSK_SK	289
			pOP-CEO00834_EST_C_1_pSK_SK	221
			pOP-CEO00835_EST_C_1_pSK_SK	193
			pOP-CEO00843_EST_C_1_pSK_SK	591
			pOP-CEO00844_EST_C_1_pSK_SK	271
			pOP-CEO00846_EST_C_1_pSK_SK	107
			pOP-CEO00850_EST_C_1_pSK_SK	310
			pOP-CEO00852_EST_C_1_pSK_SK	221
			pOP-CEO00853_EST_C_1_pSK_SK	386
			pOP-CEO00854_EST_C_1_pSK_SK	350
			pOP-CEO00864_EST_C_1_pSK_SK	310
			pOP-CEO00868_EST_C_1_pSK_SK	388
			pOP-CEO00876_EST_C_1_pSK_SK	315
			pOP-CEO00879_EST_C_1_pSK_SK	449
			pOP-CEO00880_EST_C_1_pSK_SK	142
			pOP-CEO00883_EST_C_1_pSK_SK	328
			pOP-CEO00886_EST_C_1_pSK_SK	134
			pOP-CEO00889_EST_C_1_pSK_SK	287
			pOP-CEO00890_EST_C_1_pSK_SK	283
			pOP-CEO00893_EST_C_1_pSK_SK	320
			pOP-CEO00901_EST_C_1_pSK_SK	456
			pOP-CEO00902_EST_C_1_pSK_SK	153
			pOP-CEO00903_EST_C_1_pSK_SK	240
			pOP-CEO00906_EST_C_1_pSK_SK	100
			pOP-CEO00909_EST_C_1_pSK_SK	214
			pOP-CEO00924_EST_C_1_pSK_SK	472
			pOP-CEO00926_EST_C_1_pSK_SK	502
			pOP-CEO00930_EST_C_1_pSK_SK	245
			pOP-CEO00935_EST_C_1_pSK_SK	579
			pOP-CEO00946_EST_C_1_pSK_SK	589
			pOP-CEO00948_EST_C_1_pSK_SK	258
			pOP-CEO00955_EST_C_1_pSK_SK	184
			pOP-CEO00957_EST_C_1_pSK_SK	470
			pOP-CEO00970_EST_C_1_pSK_SK	113
			pOP-CEO00977_EST_C_1_pSK_SK	324
			pOP-CEO00978_EST_C_1_pSK_SK	369
			pOP-CEO00979_EST_C_1_pSK_SK	187

			pOP-CEO00980_EST_C_1_pSK_SK	123
			pOP-CEO00981_EST_C_1_pSK_SK	329
			pOP-CEO00984_EST_C_1_pSK_SK	256
			pOP-CEO01002_EST_C_1_pSK_SK	266
			pOP-CEO01007_EST_C_1_pSK_SK	181
			pOP-CEO01010_EST_C_1_pSK_SK	528
			pOP-CEO01012_EST_C_1_pSK_SK	132
			pOP-CEO01013_EST_C_1_pSK_SK	234
			pOP-CEO01018_EST_C_1_pSK_SK	223
			pOP-CEO01021_EST_C_1_pSK_SK	205
			pOP-CEO01024_EST_C_1_pSK_SK	504
			pOP-CEO01026_EST_C_1_pSK_SK	228
			pOP-CEO01029_EST_C_1_pSK_SK	253
			pOP-CEO01030_EST_C_1_pSK_SK	214
			pOP-CEO01036_EST_C_1_pSK_SK	200
			pOP-CEO01041_EST_C_1_pSK_SK	193
			pOP-CEO01043_EST_C_1_pSK_SK	288
			pOP-CEO01044_EST_C_1_pSK_SK	303
			pOP-CEO01047_EST_C_1_pSK_SK	417
			pOP-CEO01049_EST_C_1_pSK_SK	221
			pOP-CEO01050_EST_C_1_pSK_SK	226
			pOP-CEO01051_EST_C_1_pSK_SK	234
			pOP-CEO01063_EST_C_1_pSK_SK	388
			pOP-CEO01065_EST_C_1_pSK_SK	243
			pOP-CEO01071_EST_C_1_pSK_SK	371
			pOP-CEO01075_EST_C_1_pSK_SK	127
			pOP-CEO01078_EST_C_1_pSK_SK	130
			pOP-CEO01081_EST_C_1_pSK_SK	355
			pOP-CEO01082_EST_C_1_pSK_SK	668
			pOP-CEO01083_EST_C_1_pSK_SK	603
			pOP-CEO01090_EST_C_1_pSK_SK	460
			pOP-CEO01091_EST_C_1_pSK_SK	103
			pOP-CEO01092_EST_C_1_pSK_SK	186
			pOP-CEO01094_EST_C_1_pSK_SK	136
			pOP-CEO01095_EST_C_1_pSK_SK	355
			pOP-CEO01096_EST_C_1_pSK_SK	313
			pOP-CEO01097_EST_C_1_pSK_SK	379
			pOP-CEO01099_EST_C_1_pSK_SK	373
			pOP-CEO01100_EST_C_1_pSK_SK	322
			pOP-CEO01101_EST_C_1_pSK_SK	381
			pOP-CEO01103_EST_C_1_pSK_SK	360
			pOP-CEO01111_EST_C_1_pSK_SK	398
			pOP-CEO01115_EST_C_1_pSK_SK	443
			pOP-CEO01124_EST_C_1_pSK_SK	212
			pOP-CEO01125_EST_C_1_pSK_SK	370
			pOP-CEO01132_EST_C_1_pSK_SK	146
			pOP-CEO01134_EST_C_1_pSK_SK	145
			pOP-CEO01135_EST_C_1_pSK_SK	244
			pOP-CEO01141_EST_C_1_pSK_SK	309
			pOP-CEO01142_EST_C_1_pSK_SK	371
			pOP-CEO01151_EST_C_1_pSK_SK	114
			pOP-CEO01152_EST_C_1_pSK_SK	417
			pOP-CEO01156_EST_C_1_pSK_SK	472
			pOP-CEO01158_EST_C_1_pSK_SK	182
			pOP-CEO01162_EST_C_1_pSK_SK	749
			pOP-CEO01164_EST_C_1_pSK_SK	211

			pOP-CEO01171_EST_C_1_pSK_SK	187
			pOP-CEO01172_EST_C_1_pSK_SK	315
			pOP-CEO01177_EST_C_1_pSK_SK	198
			pOP-CEO01179_EST_C_1_pSK_SK	347
			pOP-CEO01180_EST_C_1_pSK_SK	401
			pOP-CEO01182_EST_C_1_pSK_SK	462
			pOP-CEO01183_EST_C_1_pSK_SK	401
			pOP-CEO01184_EST_C_1_pSK_SK	113
			pOP-CEO01188_EST_C_1_pSK_SK	349
			pOP-CEO01190_EST_C_1_pSK_SK	209
			pOP-CEO01194_EST_C_1_pSK_SK	507
			pOP-CEO01196_EST_C_1_pSK_SK	430
			pOP-CEO01199_EST_C_1_pSK_SK	320
			pOP-CEO01210_EST_C_1_pSK_SK	162
			pOP-CEO01218_EST_C_1_pSK_SK	173
			pOP-CEO01221_EST_C_1_pSK_SK	206
			pOP-CEO01223_EST_C_1_pSK_SK	478
			pOP-CEO01227_EST_C_1_pSK_SK	102
			pOP-CEO01230_EST_C_1_pSK_SK	179
			pOP-CEO01232_EST_C_1_pSK_SK	517
			pOP-CEO01233_EST_C_1_pSK_SK	206
			pOP-CEO01235_EST_C_1_pSK_SK	559
			pOP-CEO01238_EST_C_1_pSK_SK	305
			pOP-CEO01239_EST_C_1_pSK_SK	227
			pOP-CEO01243_EST_C_1_pSK_SK	512
			pOP-CEO01244_EST_C_1_pSK_SK	383
			pOP-CEO01245_EST_C_1_pSK_SK	273
			pOP-CEO01247_EST_C_1_pSK_SK	212
			pOP-CEO01251	196
			pOP-CEO01252	435
			pOP-CEO01254	234
			pOP-CEO01255	305
			pOP-CEO01256	243
			pOP-CEO01258	179
			pOP-CEO01259	145
			pOP-CEO01260	445
			pOP-CEO01261	180
			pOP-CEO01263	571
			pOP-CEO01266	317
			pOP-CEO01267	241
			pOP-CEO01269	289
			pOP-CEO01271	175
			pOP-CEO01272	450
			pOP-CEO01279	328
			pOP-CEO01291	497
			pOP-CEO01293	228
			pOP-CEO01294	408
			pOP-CEO01296	272
			pOP-CEO01297	399
			pOP-CEO01301	439
			pOP-CEO01304	109
			pOP-CEO01308	164
			pOP-CEO01310	153
			pOP-CEO01314	161
			pOP-CEO01319	222
			pOP-CEO01330	318

			pOP-CEO01332	333
			pOP-CEO01337	721
			pOP-CEO01340	470
			pOP-CEO01344	311
			pOP-CEO01345	119
			pOP-CEO01346	364
			pOP-CEO01355	104
			pOP-CEO01358	196
			pOP-CEO01359	199
			pOP-CEO01360	336
			pOP-CEO01361_EST_C_1_pSK_SK	274
			pOP-CEO01364_EST_C_1_pSK_SK	297
			pOP-CEO01366_EST_C_1_pSK_SK	534
			pOP-CEO01368_EST_C_1_pSK_SK	567
			pOP-CEO01370_EST_C_1_pSK_SK	353
			pOP-CEO01372_EST_C_1_pSK_SK	225
			pOP-CEO01375_EST_C_1_pSK_SK	468
			pOP-CEO01378_EST_C_1_pSK_SK	357
			pOP-CEO01381_EST_C_1_pSK_SK	463
			pOP-CEO01384_EST_C_1_pSK_SK	346
			pOP-CEO01385_EST_C_1_pSK_SK	316
			pOP-CEO01392_EST_C_1_pSK_SK	732
			pOP-CEO01394_EST_C_1_pSK_SK	275
			pOP-CEO01397_EST_C_1_pSK_SK	287
			pOP-CEO01404_EST_C_1_pSK_SK	105
			pOP-CEO01409_EST_C_1_pSK_SK	687
			pOP-CEO01410_EST_C_1_pSK_SK	146
			pOP-CEO01412_EST_C_1_pSK_SK	538
			pOP-CEO01413_EST_C_1_pSK_SK	176
			pOP-CEO01421_EST_C_1_pSK_SK	196
			pOP-CEO01425_EST_C_1_pSK_SK	422
			pOP-CEO01426_EST_C_1_pSK_SK	133
			pOP-CEO01428_EST_C_1_pSK_SK	328
			pOP-CEO01429_EST_C_1_pSK_SK	209
			pOP-CEO01431_EST_C_1_pSK_SK	325
			pOP-CEO01435_EST_C_1_pSK_SK	512
			pOP-CEO01436_EST_C_1_pSK_SK	364
			pOP-CEO01437_EST_C_1_pSK_SK	346
			pOP-CEO01439_EST_C_1_pSK_SK	216
			pOP-CEO01442_EST_C_1_pSK_SK	669
			pOP-CEO01443_EST_C_1_pSK_SK	232
			pOP-CEO01444_EST_C_1_pSK_SK	275
			pOP-CEO01447_EST_C_1_pSK_SK	320
			pOP-CEO01452_EST_C_1_pSK_SK	249
			pOP-CEO01455_EST_C_1_pSK_SK	363
			pOP-CEO01456_EST_C_1_pSK_SK	765
			pOP-CEO01458_EST_C_1_pSK_SK	528
			pOP-CEO01461_EST_C_1_pSK_SK	290
			pOP-CEO01466_EST_C_1_pSK_SK	437
			pOP-CEO01467_EST_C_1_pSK_SK	456
			pOP-CEO01468_EST_C_1_pSK_SK	246
			pOP-CEO01470_EST_C_1_pSK_SK	716
			pOP-CEO01471_EST_C_1_pSK_SK	212
			pOP-CEO01472_EST_C_1_pSK_SK	197
			pOP-CEO01473_EST_C_1_pSK_SK	177
			pOP-CEO01474_EST_C_1_pSK_SK	135

			pOP-CEO01478_EST_C_1_pSK_SK	124
			pOP-CEO01479_EST_C_1_pSK_SK	134
			pOP-CEO01480_EST_C_1_pSK_SK	210
			pOP-CEO01483_EST_C_1_pSK_SK	151
			pOP-CEO01484_EST_C_1_pSK_SK	282
			pOP-CEO01488_EST_C_1_pSK_SK	543
			pOP-CEO01490_EST_C_1_pSK_SK	190
			pOP-CEO01492_EST_C_1_pSK_SK	279
			pOP-CEO01496_EST_C_1_pSK_SK	226
			pOP-CEO01499_EST_C_1_pSK_SK	526
			pOP-CEO01504_EST_C_1_pSK_SK	255
			pOP-CEO01506_EST_C_1_pSK_SK	263
			pOP-CEO01508_EST_C_1_pSK_SK	548
			pOP-CEO01511_EST_C_1_pSK_SK	317
			pOP-CEO01512_EST_C_1_pSK_SK	348
			pOP-CEO01514_EST_C_1_pSK_SK	279
			pOP-CEO01516_EST_C_1_pSK_SK	267
			pOP-CEO01521_EST_C_1_pSK_SK	374
			pOP-CEO01524_EST_C_1_pSK_SK	265
			pOP-CEO01525_EST_C_1_pSK_SK	433
			pOP-CEO01526_EST_C_1_pSK_SK	698
			pOP-CEO01528_EST_C_1_pSK_SK	238
			pOP-CEO01539_EST_C_1_pSK_SK	347
			pOP-CEO01543_EST_C_1_pSK_SK	350
			pOP-CEO01555_EST_C_1_pSK_SK	227
			pOP-CEO01560_EST_C_1_pSK_SK	431
			pOP-CEO01562_EST_C_1_pSK_SK	260
			pOP-CEO01564_EST_C_1_pSK_SK	250
			pOP-CEO01566_EST_C_1_pSK_SK	232
			pOP-CEO01576_EST_C_1_pSK_SK	131
			pOP-CEO01577_EST_C_1_pSK_SK	252
			pOP-CEO01580_EST_C_1_pSK_SK	214
			pOP-CEO01582_EST_C_1_pSK_SK	266
			pOP-CEO01584_EST_C_1_pSK_SK	285
			pOP-CEO01591_EST_C_1_pSK_SK	180
			pOP-CEO01592_EST_C_1_pSK_SK	247
			pOP-CEO01596_EST_C_1_pSK_SK	545
			pOP-CEO01597_EST_C_1_pSK_SK	393
			pOP-CEO01600_EST_C_1_pSK_SK	365
			pOP-CEO01602_EST_C_1_pSK_SK	197
			pOP-CEO01603_EST_C_1_pSK_SK	119
			pOP-CEO01609_EST_C_1_pSK_SK	362
			pOP-CEO01611_EST_C_1_pSK_SK	439
			pOP-CEO01614_EST_C_1_pSK_SK	145
			pOP-CEO01616_EST_C_1_pSK_SK	484
			pOP-CEO01618_EST_C_1_pSK_SK	362
			pOP-CEO01619_EST_C_1_pSK_SK	164
			pOP-CEO01621_EST_C_1_pSK_SK	213
			pOP-CEO01625_EST_C_1_pSK_SK	142
			pOP-CEO01630_EST_C_1_pSK_SK	507
			pOP-CEO01631_EST_C_1_pSK_SK	182
			pOP-CEO01632_EST_C_1_pSK_SK	416
			pOP-CEO01638_EST_C_1_pSK_SK	311
			pOP-CEO01641_EST_C_1_pSK_SK	598
			pOP-CEO01642_EST_C_1_pSK_SK	346
			pOP-CEO01643_EST_C_1_pSK_SK	236

			pOP-CEO01648_EST_C_1_pSK_SK	229
			pOP-CEO01654_EST_C_1_pSK_SK	503
			pOP-CEO01655_EST_C_1_pSK_SK	677
			pOP-CEO01658_EST_C_1_pSK_SK	731
			pOP-CEO01663_EST_C_1_pSK_SK	270
			pOP-CEO01664_EST_C_1_pSK_SK	432
			pOP-CEO01671_EST_C_1_pSK_SK	518
			pOP-CEO01672_EST_C_1_pSK_SK	306
			pOP-CEO01674_EST_C_1_pSK_SK	493
			pOP-CEO01675_EST_C_1_pSK_SK	144
			pOP-CEO01676_EST_C_1_pSK_SK	224
			pOP-CEO01678_EST_C_1_pSK_SK	216
			pOP-CEO01681_EST_C_1_pSK_SK	153
			pOP-CEO01683_EST_C_1_pSK_SK	206
			pOP-CEO01684_EST_C_1_pSK_SK	487
			pOP-CEO01686_EST_C_1_pSK_SK	169
			pOP-CEO01693_EST_C_1_pSK_SK	694
			pOP-CEO01694_EST_C_1_pSK_SK	110
			pOP-CEO01696_EST_C_1_pSK_SK	100
			pOP-CEO01699_EST_C_1_pSK_SK	533
			pOP-CEO01701_EST_C_1_pSK_SK	638
			pOP-CEO01706_EST_C_1_pSK_SK	156
			pOP-CEO01710_EST_C_1_pSK_SK	303
			pOP-CEO01711_EST_C_1_pSK_SK	324
			pOP-CEO01715_EST_C_1_pSK_SK	436
			pOP-CEO01716_EST_C_1_pSK_SK	233
			pOP-CEO01717_EST_C_1_pSK_SK	472
			pOP-CEO01723_EST_C_1_pSK_SK	413
			pOP-CEO01727_EST_C_1_pSK_SK	210
			pOP-CEO01729_EST_C_1_pSK_SK	458
			pOP-CEO01730_EST_C_1_pSK_SK	172
			pOP-CEO01732_EST_C_1_pSK_SK	271
			pOP-CEO01742_EST_C_1_pSK_SK	350
			pOP-CEO01743_EST_C_1_pSK_SK	165
			pOP-CEO01745_EST_C_1_pSK_SK	347
			pOP-CEO01749_EST_C_1_pSK_SK	372
			pOP-CEO01755_EST_C_1_pSK_SK	163
			pOP-CEO01758_EST_C_1_pSK_SK	373
			pOP-CEO01760_EST_C_1_pSK_SK	371
			pOP-CEO01763_EST_C_1_pSK_SK	101
			pOP-CEO01766_EST_C_1_pSK_SK	206
			pOP-CEO01769_EST_C_1_pSK_SK	215
			pOP-CEO01770_EST_C_1_pSK_SK	372
			pOP-CEO01772_EST_C_1_pSK_SK	326
			pOP-CEO01773_EST_C_1_pSK_SK	374
			pOP-CEO01779_EST_C_1_pSK_SK	371
			pOP-CEO01782_EST_C_1_pSK_SK	313
			pOP-CEO01783_EST_C_1_pSK_SK	342
			pOP-CEO01784_EST_C_1_pSK_SK	360
			pOP-CEO01785_EST_C_1_pSK_SK	316
			pOP-CEO01787_EST_C_1_pSK_SK	360
			pOP-CEO01791_EST_C_1_pSK_SK	296
			pOP-CEO01793_EST_C_1_pSK_SK	211
			pOP-CEO01799_EST_C_1_pSK_SK	102
			pOP-CEO01802_EST_C_1_pSK_SK	360
			pOP-CEO01803_EST_C_1_pSK_SK	179

			pOP-CEO01804_EST_C_1_pSK_SK	326
			pOP-CEO01806_EST_C_1_pSK_SK	247
			pOP-CEO01807_EST_C_1_pSK_SK	256
			pOP-CEO01813_EST_C_1_pSK_SK	195
			pOP-CEO01817_EST_C_1_pSK_SK	374
			pOP-CEO01818_EST_C_1_pSK_SK	212
			pOP-CEO01820_EST_C_1_pSK_SK	182
			pOP-CEO01824_EST_C_1_pSK_SK	377
			pOP-CEO01830_EST_C_1_pSK_SK	213
			pOP-CEO01835_EST_C_1_pSK_SK	362
			pOP-CEO01837_EST_C_1_pSK_SK	325
			pOP-CEO01838_EST_C_1_pSK_SK	275
			pOP-CEO01840_EST_C_1_pSK_SK	368
			pOP-CEO01841_EST_C_1_pSK_SK	518
			pOP-CEO01842_EST_C_1_pSK_SK	489
			pOP-CEO01843_EST_C_1_pSK_SK	418
			pOP-CEO01850_EST_C_1_pSK_SK	199
			pOP-CEO01859_EST_C_1_pSK_SK	465
			pOP-CEO01860_EST_C_1_pSK_SK	280
			pOP-CEO01861_EST_C_1_pSK_SK	287
			pOP-CEO01864_EST_C_1_pSK_SK	149
			pOP-CEO01867_EST_C_1_pSK_SK	161
			pOP-CEO01870_EST_C_1_pSK_SK	223
			pOP-CEO01874_EST_C_1_pSK_SK	422
			pOP-CEO01876_EST_C_1_pSK_SK	216
			pOP-CEO01878_EST_C_1_pSK_SK	230
			pOP-CEO01881_EST_C_1_pSK_SK	137
			pOP-CEO01883_EST_C_1_pSK_SK	303
			pOP-CEO01891_EST_C_1_pSK_SK	320
			pOP-CEO01895_EST_C_1_pSK_SK	123
			pOP-CEO01898_EST_C_1_pSK_SK	408
			pOP-CEO01900_EST_C_1_pSK_SK	249
			pOP-CEO01904_EST_C_1_pSK_SK	456
			pOP-CEO01906_EST_C_1_pSK_SK	537
			pOP-CEO01910_EST_C_1_pSK_SK	437
			pOP-CEO01915_EST_C_1_pSK_SK	371
			pOP-CEO01916_EST_C_1_pSK_SK	374
			pOP-CEO01921_EST_C_1_pSK_SK	448
			pOP-CEO01923_EST_C_1_pSK_SK	445
			pOP-CEO01928_EST_C_1_pSK_SK	303
			pOP-CEO01934_EST_C_1_pSK_SK	166
			pOP-CEO01937_EST_C_1_pSK_SK	197
			pOP-CEO01942_EST_C_1_pSK_SK	297
			pOP-CEO01946_EST_C_1_pSK_SK	573
			pOP-CEO01949_EST_C_1_pSK_SK	117
			pOP-CEO01951_EST_C_1_pSK_SK	303
			pOP-CEO01957_EST_C_1_pSK_SK	234
			pOP-CEO01958_EST_C_1_pSK_SK	156
			pOP-CEO01959_EST_C_1_pSK_SK	225
			pOP-CEO01963_EST_C_1_pSK_SK	287
			pOP-CEO01964_EST_C_1_pSK_SK	554
			pOP-CEO01965_EST_C_1_pSK_SK	384
			pOP-CEO01968_EST_C_1_pSK_SK	125
			pOP-CEO01970_EST_C_1_pSK_SK	242
			pOP-CEO01972_EST_C_1_pSK_SK	111
			pOP-CEO01973_EST_C_1_pSK_SK	322

			pOP-CEO01974_EST_C_1_pSK_SK	325
			pOP-CEO01975_EST_C_1_pSK_SK	187
			pOP-CEO01978_EST_C_1_pSK_SK	559
			pOP-CEO01980_EST_C_1_pSK_SK	195
			pOP-CEO01981_EST_C_1_pSK_SK	199
			pOP-CEO01982_EST_C_1_pSK_SK	383
			pOP-CEO01987_EST_C_1_pSK_SK	371
			pOP-CEO01989_EST_C_1_pSK_SK	387
			pOP-CEO01992_EST_C_1_pSK_SK	278
			pOP-CEO01995_EST_C_1_pSK_SK	163
			pOP-CEO01997_EST_C_1_pSK_SK	319
			pOP-CEO01998_EST_C_1_pSK_SK	467
			pOP-CEO02002_EST_C_1_pSK_SK	155
			pOP-CEO02004_EST_C_1_pSK_SK	285
			pOP-CEO02005_EST_C_1_pSK_SK	376
			pOP-CEO02009_EST_C_1_pSK_SK	190
			pOP-CEO02011_EST_C_1_pSK_SK	379
			pOP-CEO02012_EST_C_1_pSK_SK	363
			pOP-CEO02013_EST_C_1_pSK_SK	378
			pOP-CEO02016_EST_C_1_pSK_SK	253
			pOP-CEO02026_EST_C_1_pSK_SK	378
			pOP-CEO02029_EST_C_1_pSK_SK	334
			pOP-CEO02030_EST_C_1_pSK_SK	322
			pOP-CEO02031_EST_C_1_pSK_SK	368
			pOP-CEO02033_EST_C_1_pSK_SK	236
			pOP-CEO02034_EST_C_1_pSK_SK	284
			pOP-CEO02035_EST_C_1_pSK_SK	269
			pOP-CEO02038_EST_C_1_pSK_SK	300
			pOP-CEO02040_EST_C_1_pSK_SK	144
			pOP-CEO02041_EST_C_1_pSK_SK	314
			pOP-CEO02046_EST_C_1_pSK_SK	342
			pOP-CEO02048_EST_C_1_pSK_SK	342
			pOP-CEO02050_EST_C_1_pSK_SK	294
			pOP-CEO02055_EST_C_1_pSK_SK	301
			pOP-CEO02058_EST_C_1_pSK_SK	125
			pOP-CEO02059_EST_C_1_pSK_SK	195
			pOP-CEO02062_EST_C_1_pSK_SK	287
			pOP-CEO02064_EST_C_1_pSK_SK	121
			pOP-CEO02065_EST_C_1_pSK_SK	341
			pOP-CEO02066_EST_C_1_pSK_SK	342
			pOP-CEO02068_EST_C_1_pSK_SK	279
			pOP-CEO02076_EST_C_1_pSK_SK	148
			pOP-CEO02079_EST_C_1_pSK_SK	405
			pOP-CEO02080_EST_C_1_pSK_SK	453
			pOP-CEO02084_EST_C_1_pSK_SK	478
			pOP-CEO02085_EST_C_1_pSK_SK	312
			pOP-CEO02086_EST_C_1_pSK_SK	325
			pOP-CEO02087_EST_C_1_pSK_SK	264
			pOP-CEO02090_EST_C_1_pSK_SK	278
			pOP-CEO02105_EST_C_1_pSK_SK	293
			pOP-CEO02107_EST_C_1_pSK_SK	185
			pOP-CEO02109_EST_C_1_pSK_SK	432
			pOP-CEO02124_EST_C_1_pSK_SK	400
			pOP-CEO02131_EST_C_1_pSK_SK	265
			pOP-CEO02135_EST_C_1_pSK_SK	207
			pOP-CEO02137_EST_C_1_pSK_SK	395

			pOP-CEO02139_EST_C_1_pSK_SK	352
			pOP-CEO02140_EST_C_1_pSK_SK	100
			pOP-CEO02142_EST_C_1_pSK_SK	389
			pOP-CEO02145_EST_C_1_pSK_SK	548
			pOP-CEO02147_EST_C_1_pSK_SK	335
			pOP-CEO02149_EST_C_1_pSK_SK	518
			pOP-CEO02152_EST_C_1_pSK_SK	263
			pOP-CEO02160_EST_C_1_pSK_SK	298
			pOP-CEO02168_EST_C_1_pSK_SK	259
			pOP-CEO02169_EST_C_1_pSK_SK	209
			pOP-CEO02173_EST_C_1_pSK_SK	556
			pOP-CEO02177_EST_C_1_pSK_SK	542
			pOP-CEO02178_EST_C_1_pSK_SK	244
			pOP-CEO02179_EST_C_1_pSK_SK	384
			pOP-CEO02188_EST_C_1_pSK_SK	232
			pOP-CEO02194_EST_C_1_pSK_SK	308
			pOP-CEO02195_EST_C_1_pSK_SK	211
			pOP-CEO02207_EST_C_1_pSK_SK	561
			pOP-CEO02211_EST_C_1_pSK_SK	415
			pOP-CEO02212_EST_C_1_pSK_SK	289
			pOP-CEO02215_EST_C_1_pSK_SK	344
			pOP-CEO02219_EST_C_1_pSK_SK	284
			pOP-CEO02225_EST_C_1_pSK_SK	650
			pOP-CEO02235_EST_C_1_pSK_SK	224
			pOP-CEO02236_EST_C_1_pSK_SK	241
			pOP-CEO02252_EST_C_1_pSK_SK	437
			pOP-CEO02261_EST_C_1_pSK_SK	374
			pOP-CEO02262_EST_C_1_pSK_SK	520
			pOP-CEO02265_EST_C_1_pSK_SK	204
			pOP-CEO02267_EST_C_1_pSK_SK	202
			pOP-CEO02270_EST_C_1_pSK_SK	173
			pOP-CEO02271_EST_C_1_pSK_SK	122
			pOP-CEO02274_EST_C_1_pSK_SK	310
			pOP-CEO02276_EST_C_1_pSK_SK	356
			pOP-CEO02279_EST_C_1_pSK_SK	390
			pOP-CEO02304_EST_C_1_pSK_SK	479
			pOP-CEO02309_EST_C_1_pSK_SK	564
			pOP-CEO02310_EST_C_1_pSK_SK	368
			pOP-CEO02319_EST_C_1_pSK_SK	436
			pOP-CEO02322_EST_C_1_pSK_SK	206
			pOP-CEO02323_EST_C_1_pSK_SK	412
			pOP-CEO02326_EST_C_1_pSK_SK	154
			pOP-CEO02328_EST_C_1_pSK_SK	560
			pOP-CEO02332_EST_C_1_pSK_SK	306
			pOP-CEO02333_EST_C_1_pSK_SK	272
			pOP-CEO02336_EST_C_1_pSK_SK	378
			pOP-CEO02337_EST_C_1_pSK_SK	200
			pOP-CEO02339_EST_C_1_pSK_SK	370
			pOP-CEO02342_EST_C_1_pSK_SK	265
			pOP-CEO02346_EST_C_1_pSK_SK	347
			pOP-CEO02347_EST_C_1_pSK_SK	464
			pOP-CEO02349_EST_C_1_pSK_SK	264
			pOP-CEO02357_EST_C_1_pSK_SK	457
			pOP-CEO02359_EST_C_1_pSK_SK	195
			pOP-CEO02361_EST_C_1_pSK_SK	275
			pOP-CEO02362_EST_C_1_pSK_SK	323

			pOP-CEO02364_EST_C_1_pSK_SK	216
			pOP-CEO02368_EST_C_1_pSK_SK	382
			pOP-CEO02370_EST_C_1_pSK_SK	237
			pOP-CEO02371_EST_C_1_pSK_SK	368
			pOP-CEO02379_EST_C_1_pSK_SK	304
			pOP-CEO02380_EST_C_1_pSK_SK	400
			pOP-CEO02384_EST_C_1_pSK_SK	320
			pOP-CEO02385_EST_C_1_pSK_SK	535
			pOP-CEO02388_EST_C_1_pSK_SK	482
			pOP-CEO02391_EST_C_1_pSK_SK	630
			pOP-CEO02392_EST_C_1_pSK_SK	477
			pOP-CEO02393_EST_C_1_pSK_SK	314
			pOP-CEO02394_EST_C_1_pSK_SK	504
			pOP-CEO02396_EST_C_1_pSK_SK	105
			pOP-CEO02401_EST_C_1_pSK_SK	593
			pOP-CEO02408_EST_C_1_pSK_SK	409
			pOP-CEO02417_EST_C_1_pSK_SK	521
			pOP-CEO02421_EST_C_1_pSK_SK	398
			pOP-CEO02428_EST_C_1_pSK_SK	296
			pOP-CEO02430_EST_C_1_pSK_SK	396
			pOP-CEO02433_EST_C_1_pSK_SK	263
			pOP-CEO02434_EST_C_1_pSK_SK	243
			pOP-CEO02438_EST_C_1_pSK_SK	144
			pOP-CEO02440_EST_C_1_pSK_SK	423
			pOP-CEO02441_EST_C_1_pSK_SK	253
			pOP-CEO02444_EST_C_1_pSK_SK	318
			pOP-CEO02449_EST_C_1_pSK_SK	248
			pOP-CEO02450_EST_C_1_pSK_SK	237
			pOP-CEO02456_EST_C_1_pSK_SK	224
			pOP-CEO02461_EST_C_1_pSK_SK	612
			pOP-CEO02462_EST_C_1_pSK_SK	196
			pOP-CEO02463_EST_C_1_pSK_SK	506
			pOP-CEO02472_EST_C_1_pSK_SK	354
			pOP-CEO02475_EST_C_1_pSK_SK	361
			pOP-CEO02479_EST_C_1_pSK_SK	511
			pOP-CEO02480_EST_C_1_pSK_SK	678
			pOP-CEO02485_EST_C_1_pSK_SK	170
			pOP-CEO02486_EST_C_1_pSK_SK	324
			pOP-CEO02489_EST_C_1_pSK_SK	432
			pOP-CEO02491_EST_C_1_pSK_SK	306
			pOP-CEO02494_EST_C_1_pSK_SK	496
			pOP-CEO02499_EST_C_1_pSK_SK	289
			pOP-CEO02501_EST_C_1_pSK_SK	498
			pOP-CEO02502_EST_C_1_pSK_SK	357
			pOP-CEO02510_EST_C_1_pSK_SK	661
			pOP-CEO02513_EST_C_1_pSK_SK	153
			pOP-CEO02515_EST_C_1_pSK_SK	141
			pOP-CEO02516_EST_C_1_pSK_SK	146
			pOP-CEO02521_EST_C_1_pSK_SK	197
			pOP-CEO02525_EST_C_1_pSK_SK	151
			pOP-CEO02526_EST_C_1_pSK_SK	149
			pOP-CEO02528_EST_C_1_pSK_SK	630
			pOP-CEO02532_EST_C_1_pSK_SK	373
			pOP-CEO02533_EST_C_1_pSK_SK	118
			pOP-CEO02539_EST_C_1_pSK_SK	176
			pOP-CEO02547_EST_C_1_pSK_SK	502

			pOP-CEO02548_EST_C_1_pSK_SK	531
			pOP-CEO02549_EST_C_1_pSK_SK	475
			pOP-CEO02552_EST_C_1_pSK_SK	536
			pOP-CEO02555_EST_C_1_pSK_SK	567
			pOP-CEO02558_EST_C_1_pSK_SK	375
			pOP-CEO02560_EST_C_1_pSK_SK	584
			pOP-CEO02564_EST_C_1_pSK_SK	487
			pOP-CEO02565_EST_C_1_pSK_SK	221
			pOP-CEO02566_EST_C_1_pSK_SK	451
			pOP-CEO02571_EST_C_1_pSK_SK	321
			pOP-CEO02573_EST_C_1_pSK_SK	384
			pOP-CEO02575_EST_C_1_pSK_SK	571
			pOP-CEO02578_EST_C_1_pSK_SK	284
			pOP-CEO02584_EST_C_1_pSK_SK	230
			pOP-CEO02585_EST_C_1_pSK_SK	269
			pOP-CEO02593_EST_C_1_pSK_SK	206
			pOP-CEO02598_EST_C_1_pSK_SK	483
			pOP-CEO02609_EST_C_1_pSK_SK	489
			pOP-CEO02610_EST_C_1_pSK_SK	467
			pOP-CEO02612_EST_C_1_pSK_SK	498
			pOP-CEO02616_EST_C_1_pSK_SK	245
			pOP-CEO02619_EST_C_1_pSK_SK	435
			pOP-CEO02620_EST_C_1_pSK_SK	239
			pOP-CEO02623_EST_C_1_pSK_SK	452
			pOP-CEO02624_EST_C_1_pSK_SK	551
			pOP-CEO02625_EST_C_1_pSK_SK	371
			pOP-CEO02631_EST_C_1_pSK_SK	340
			pOP-CEO02633_EST_C_1_pSK_SK	489
			pOP-CEO02634_EST_C_1_pSK_SK	226
			pOP-CEO02635_EST_C_1_pSK_SK	180
			pOP-CEO02636_EST_C_1_pSK_SK	210
			pOP-CEO02637_EST_C_1_pSK_SK	510
			pOP-CEO02638_EST_C_1_pSK_SK	285
			pOP-CEO02641_EST_C_1_pSK_SK	514
			pOP-CEO02643_EST_C_1_pSK_SK	694
			pOP-CEO02644_EST_C_1_pSK_SK	231
			pOP-CEO02648_EST_C_1_pSK_SK	395
			pOP-CEO02650_EST_C_1_pSK_SK	254
			pOP-CEO02652_EST_C_1_pSK_SK	563
			pOP-CEO02653_EST_C_1_pSK_SK	441
			pOP-CEO02654_EST_C_1_pSK_SK	456
			pOP-CEO02659_EST_C_1_pSK_SK	372
			pOP-CEO02667_EST_C_1_pSK_SK	542
			pOP-CEO02668_EST_C_1_pSK_SK	320
			pOP-CEO02670_EST_C_1_pSK_SK	337
			pOP-CEO02672_EST_C_1_pSK_SK	295
			pOP-CEO02675_EST_C_1_pSK_SK	402
			pOP-CEO02676_EST_C_1_pSK_SK	384
			pOP-CEO02677_EST_C_1_pSK_SK	377
			pOP-CEO02678_EST_C_1_pSK_SK	213
			pOP-CEO02679_EST_C_1_pSK_SK	299
			pOP-CEO02681_EST_C_1_pSK_SK	505
			pOP-CEO02682_EST_C_1_pSK_SK	294
			pOP-CEO02685_EST_C_1_pSK_SK	391
			pOP-CEO02686_EST_C_1_pSK_SK	511
			pOP-CEO02689_EST_C_1_pSK_SK	369

			pOP-CEO02690_EST_C_1_pSK_SK	350
			pOP-CEO02694_EST_C_1_pSK_SK	366
			pOP-CEO02697_EST_C_1_pSK_SK	340
			pOP-CEO02702_EST_C_1_pSK_SK	300
			pOP-CEO02722_EST_C_1_pSK_SK	546
			pOP-CEO02728_EST_C_1_pSK_SK	434
			pOP-CEO02733_EST_C_1_pSK_SK	509
			pOP-CEO02740_EST_C_1_pSK_SK	391
			pOP-CEO02741_EST_C_1_pSK_SK	351
			pOP-CEO02742_EST_C_1_pSK_SK	563
			pOP-CEO02746_EST_C_1_pSK_SK	556
			pOP-CEO02748_EST_C_1_pSK_SK	455
			pOP-CEO02753_EST_C_1_pSK_SK	455
			pOP-CEO02754_EST_C_1_pSK_SK	303
			pOP-CEO02757_EST_C_1_pSK_SK	465
			pOP-CEO02758_EST_C_1_pSK_SK	188
			pOP-CEO02761_EST_C_1_pSK_SK	343
			pOP-CEO02762_EST_C_1_pSK_SK	354
			pOP-CEO02765_EST_C_1_pSK_SK	361
			pOP-CEO02767_EST_C_1_pSK_SK	539
			pOP-CEO02776_EST_C_1_pSK_SK	140
			pOP-CEO02781_EST_C_1_pSK_SK	175
			pOP-CEO02787_EST_C_1_pSK_SK	379
			pOP-CEO02788_EST_C_1_pSK_SK	114
			pOP-CEO02789_EST_C_1_pSK_SK	421
			pOP-CEO02790_EST_C_1_pSK_SK	240
			pOP-CEO02792_EST_C_1_pSK_SK	394
			pOP-CEO02793_EST_C_1_pSK_SK	231
			pOP-CEO02794_EST_C_1_pSK_SK	168
			pOP-CEO02804_EST_C_1_pSK_SK	508
			pOP-CEO02805_EST_C_1_pSK_SK	494
			pOP-CEO02811_EST_C_1_pSK_SK	380
			pOP-CEO02813_EST_C_1_pSK_SK	293
			pOP-CEO02815_EST_C_1_pSK_SK	431
			pOP-CEO02816_EST_C_1_pSK_SK	397
			pOP-CEO02819_EST_C_1_pSK_SK	408
			pOP-CEO02820_EST_C_1_pSK_SK	308
			pOP-CEO02822_EST_C_1_pSK_SK	339
			pOP-CEO02825_EST_C_1_pSK_SK	156
			pOP-CEO02831_EST_C_1_pSK_SK	325
			pOP-CEO02832_EST_C_1_pSK_SK	120
			pOP-CEO02834_EST_C_1_pSK_SK	566
			pOP-CEO02835_EST_C_1_pSK_SK	210
			pOP-CEO02837_EST_C_1_pSK_SK	553
			pOP-CEO02838_EST_C_1_pSK_SK	439
			pOP-CEO02839_EST_C_1_pSK_SK	358
			pOP-CEO02842_EST_C_1_pSK_SK	256
			pOP-CEO02843_EST_C_1_pSK_SK	318
			pOP-CEO02844_EST_C_1_pSK_SK	312
			pOP-CEO02851_EST_C_1_pSK_SK	492
			pOP-CEO02854_EST_C_1_pSK_SK	408
			pOP-CEO02856_EST_C_1_pSK_SK	211
			pOP-CEO02859_EST_C_1_pSK_SK	581
			pOP-CEO02865_EST_C_1_pSK_SK	207
			pOP-CEO02867_EST_C_1_pSK_SK	718
			pOP-CEO02873_EST_C_1_pSK_SK	598

			pOP-CEO02874_EST_C_1_pSK_SK	148
			pOP-CEO02875_EST_C_1_pSK_SK	205
			pOP-CEO02881_EST_C_1_pSK_SK	474
			pOP-CEO02885_EST_C_1_pSK_SK	290
			pOP-CEO02886_EST_C_1_pSK_SK	340
			pOP-CEO02890_EST_C_1_pSK_SK	323
			pOP-CEO02892_EST_C_1_pSK_SK	129
			pOP-CEO02894_EST_C_1_pSK_SK	421
			pOP-CEO02898_EST_C_1_pSK_SK	443
			pOP-CEO02900_EST_C_1_pSK_SK	175
			pOP-CEO02901_EST_C_1_pSK_SK	248
			pOP-CEO02909_EST_C_1_pSK_SK	204
			pOP-CEO02911_EST_C_1_pSK_SK	135
			pOP-CEO02912_EST_C_1_pSK_SK	560
			pOP-CEO02916_EST_C_1_pSK_SK	278
			pOP-CEO02917_EST_C_1_pSK_SK	165
			pOP-CEO02921_EST_C_1_pSK_SK	101
			pOP-CEO02927_EST_C_1_pSK_SK	346
			pOP-CEO02931_EST_C_1_pSK_SK	344
			pOP-CEO02932_EST_C_1_pSK_SK	727
			pOP-CEO02936_EST_C_1_pSK_SK	293
			pOP-CEO02938_EST_C_1_pSK_SK	315
			pOP-CEO02940_EST_C_1_pSK_SK	222
			pOP-CEO02944_EST_C_1_pSK_SK	342
			pOP-CEO02945_EST_C_1_pSK_SK	218
			pOP-CEO02949_EST_C_1_pSK_SK	157
			pOP-CEO02952_EST_C_1_pSK_SK	309
			pOP-CEO02953_EST_C_1_pSK_SK	397
			pOP-CEO02954_EST_C_1_pSK_SK	302
			pOP-CEO02959_EST_C_1_pSK_SK	148
			pOP-CEO02960_EST_C_1_pSK_SK	477
			pOP-CEO02961_EST_C_1_pSK_SK	168
			pOP-CEO02962_EST_C_1_pSK_SK	551
			pOP-CEO02963_EST_C_1_pSK_SK	126
			pOP-CEO02966_EST_C_1_pSK_SK	208
			pOP-CEO02970_EST_C_1_pSK_SK	268
			pOP-CEO02974_EST_C_1_pSK_SK	588
			pOP-CEO02975_EST_C_1_pSK_SK	460
			pOP-CEO02978_EST_C_1_pSK_SK	202
			pOP-CEO02979_EST_C_1_pSK_SK	230
			pOP-CEO02981_EST_C_1_pSK_SK	498
			pOP-CEO02984_EST_C_1_pSK_SK	377
			pOP-CEO02985_EST_C_1_pSK_SK	252
			pOP-CEO02991_EST_C_1_pSK_SK	357
			pOP-CEO02996_EST_C_1_pSK_SK	252
			pOP-CEO02998_EST_C_1_pSK_SK	151
			pOP-CEO03004_EST_C_1_pSK_SK	336
			pOP-CEO03008_EST_C_1_pSK_SK	175
			pOP-CEO03019_EST_C_1_pSK_SK	116
			pOP-CEO03023_EST_C_1_pSK_SK	425
			pOP-CEO03028_EST_C_1_pSK_SK	408
			pOP-CEO03035_EST_C_1_pSK_SK	324
			pOP-CEO03039_EST_C_1_pSK_SK	425
			pOP-CEO03042_EST_C_1_pSK_SK	571
			pOP-CEO03044_EST_C_1_pSK_SK	152
			pOP-CEO03045_EST_C_1_pSK_SK	667

			pOP-CEO03046_EST_C_1_pSK_SK	402
			pOP-CEO03050_EST_C_1_pSK_SK	244
			pOP-CEO03051_EST_C_1_pSK_SK	340
			pOP-CEO03052_EST_C_1_pSK_SK	349
			pOP-CEO03054_EST_C_1_pSK_SK	212
			pOP-CEO03056_EST_C_1_pSK_SK	376
			pOP-CEO03058_EST_C_1_pSK_SK	117
			pOP-CEO03063_EST_C_1_pSK_SK	326
			pOP-CEO03065_EST_C_1_pSK_SK	340
			pOP-CEO03068_EST_C_1_pSK_SK	456
			pOP-CEO03069_EST_C_1_pSK_SK	449
			pOP-CEO03070_EST_C_1_pSK_SK	115
			pOP-CEO03077_EST_C_1_pSK_SK	218
			pOP-CEO03078_EST_C_1_pSK_SK	371
			pOP-CEO03079_EST_C_1_pSK_SK	260
			pOP-CEO03080_EST_C_1_pSK_SK	239
			pOP-CEO03082_EST_C_1_pSK_SK	750
			pOP-CEO03087_EST_C_1_pSK_SK	438
			pOP-CEO03089_EST_C_1_pSK_SK	703
			pOP-CEO03095_EST_C_1_pSK_SK	233
			pOP-CEO03101_EST_C_1_pSK_SK	180
			pOP-CEO03109_EST_C_1_pSK_SK	233
			pOP-CEO03116_EST_C_1_pSK_SK	182
			pOP-CEO03117_EST_C_1_pSK_SK	246
			pOP-CEO03120_EST_C_1_pSK_SK	156
			pOP-CEO03125_EST_C_1_pSK_SK	239
			pOP-CEO03126_EST_C_1_pSK_SK	132
			pOP-CEO03128_EST_C_1_pSK_SK	357
			pOP-CEO03129_EST_C_1_pSK_SK	619
			pOP-CEO03130_EST_C_1_pSK_SK	434
			pOP-CEO03132_EST_C_1_pSK_SK	372
			pOP-CEO03134_EST_C_1_pSK_SK	299
			pOP-CEO03135_EST_C_1_pSK_SK	223
			pOP-CEO03139_EST_C_1_pSK_SK	297
			pOP-CEO03140_EST_C_1_pSK_SK	278
			pOP-CEO03141_EST_C_1_pSK_SK	287
			pOP-CEO03143_EST_C_1_pSK_SK	348
			pOP-CEO03145_EST_C_1_pSK_SK	703
			pOP-CEO03146_EST_C_1_pSK_SK	170
			pOP-CEO03149_EST_C_1_pSK_SK	425
			pOP-CEO03154_EST_C_1_pSK_SK	245
			pOP-CEO03157_EST_C_1_pSK_SK	543
			pOP-CEO03162_EST_C_1_pSK_SK	455
			pOP-CEO03168_EST_C_1_pSK_SK	176
			pOP-CEO03170_EST_C_1_pSK_SK	166
			pOP-CEO03173_EST_C_1_pSK_SK	374
			pOP-CEO03176_EST_C_1_pSK_SK	220
			pOP-CEO03177_EST_C_1_pSK_SK	332
			pOP-CEO03178_EST_C_1_pSK_SK	456
			pOP-CEO03179_EST_C_1_pSK_SK	608
			pOP-CEO03180_EST_C_1_pSK_SK	401
			pOP-CEO03182_EST_C_1_pSK_SK	674
			pOP-CEO03185_EST_C_1_pSK_SK	366
			pOP-CEO03186_EST_C_1_pSK_SK	299
			pOP-CEO03192_EST_C_1_pSK_SK	208
			pOP-CEO03193_EST_C_1_pSK_SK	390

			pOP-CEO03195_EST_C_1_pSK_SK	464
			pOP-CEO03196_EST_C_1_pSK_SK	323
			pOP-CEO03198_EST_C_1_pSK_SK	419
			pOP-CEO03199_EST_C_1_pSK_SK	203
			pOP-CEO03200_EST_C_1_pSK_SK	528
			pOP-CEO03207_EST_C_1_pSK_SK	222
			pOP-CEO03212_EST_C_1_pSK_SK	706
			pOP-CEO03218_EST_C_1_pSK_SK	233
			pOP-CEO03221_EST_C_1_pSK_SK	274
			pOP-CEO03230_EST_C_1_pSK_SK	206
			pOP-CEO03232_EST_C_1_pSK_SK	183
			pOP-CEO03233_EST_C_1_pSK_SK	534
			pOP-CEO03235_EST_C_1_pSK_SK	321
			pOP-CEO03236_EST_C_1_pSK_SK	513
			pOP-CEO03237_EST_C_1_pSK_SK	441
			pOP-CEO03238_EST_C_1_pSK_SK	292
			pOP-CEO03242_EST_C_1_pSK_SK	630
			pOP-CEO03247_EST_C_1_pSK_SK	713
			pOP-CEO03253_EST_C_1_pSK_SK	537
			pOP-CEO03259_EST_C_1_pSK_SK	225
			pOP-CEO03260_EST_C_1_pSK_SK	457
			pOP-CEO03262_EST_C_1_pSK_SK	316
			pOP-CEO03263_EST_C_1_pSK_SK	504
			pOP-CEO03270_EST_C_1_pSK_SK	512
			pOP-CEO03272_EST_C_1_pSK_SK	162
			pOP-CEO03273_EST_C_1_pSK_SK	460
			pOP-CEO03276_EST_C_1_pSK_SK	149
			pOP-CEO03278_EST_C_1_pSK_SK	348
			pOP-CEO03279_EST_C_1_pSK_SK	248
			pOP-CEO03281_EST_C_1_pSK_SK	225
			pOP-CEO03283_EST_C_1_pSK_SK	253
			pOP-CEO03287_EST_C_1_pSK_SK	296
			pOP-CEO03290_EST_C_1_pSK_SK	252
			pOP-CEO03291_EST_C_1_pSK_SK	216
			pOP-CEO03294_EST_C_1_pSK_SK	489
			pOP-CEO03296_EST_C_1_pSK_SK	198
			pOP-CEO03297_EST_C_1_pSK_SK	124
			pOP-CEO03298_EST_C_1_pSK_SK	248
			pOP-CEO03300_EST_C_1_pSK_SK	334
			pOP-CEO03302_EST_C_1_pSK_SK	374
			pOP-CEO03304_EST_C_1_pSK_SK	423
			pOP-CEO03308_EST_C_1_pSK_SK	426
			pOP-CEO03309_EST_C_1_pSK_SK	663
			pOP-CEO03313_EST_C_1_pSK_SK	479
			pOP-CEO03318_EST_C_1_pSK_SK	569
			pOP-CEO03320_EST_C_1_pSK_SK	239
			pOP-CEO03322_EST_C_1_pSK_SK	298
			pOP-CEO03323_EST_C_1_pSK_SK	244
			pOP-CEO03326_EST_C_1_pSK_SK	102
			pOP-CEO03332_EST_C_1_pSK_SK	134
			pOP-CEO03335_EST_C_1_pSK_SK	230
			pOP-CEO03351_EST_C_1_pSK_SK	595
			pOP-CEO03358_EST_C_1_pSK_SK	265
			pOP-CEO03367_EST_C_1_pSK_SK	450
			pOP-CEO03370_EST_C_1_pSK_SK	309
			pOP-CEO03372_EST_C_1_pSK_SK	302

			pOP-CEO03375_EST_C_1_pSK_SK	607
			pOP-CEO03376_EST_C_1_pSK_SK	376
			pOP-CEO03381_EST_C_1_pSK_SK	323
			pOP-CEO03386_EST_C_1_pSK_SK	397
			pOP-CEO03387_EST_C_1_pSK_SK	469
			pOP-CEO03388_EST_C_1_pSK_SK	216
			pOP-CEO03390_EST_C_1_pSK_SK	384
			pOP-CEO03393_EST_C_1_pSK_SK	570
			pOP-CEO03395_EST_C_1_pSK_SK	147
			pOP-CEO03398_EST_C_1_pSK_SK	452
			pOP-CEO03399_EST_C_1_pSK_SK	600
			pOP-CEO03404_EST_C_1_pSK_SK	463
			pOP-CEO03406_EST_C_1_pSK_SK	150
			pOP-CEO03407_EST_C_1_pSK_SK	553
			pOP-CEO03420_EST_C_1_pSK_SK	307
			pOP-CEO03431_EST_C_1_pSK_SK	100
			pOP-CEO03433_EST_C_1_pSK_SK	598
			pOP-CEO03434_EST_C_1_pSK_SK	549
			pOP-CEO03436_EST_C_1_pSK_SK	383
			pOP-CEO03438_EST_C_1_pSK_SK	125
			pOP-CEO03439_EST_C_1_pSK_SK	505
			pOP-CEO03443_EST_C_1_pSK_SK	340
			pOP-CEO03445_EST_C_1_pSK_SK	226
			pOP-CEO03446_EST_C_1_pSK_SK	393
			pOP-CEO03447_EST_C_1_pSK_SK	251
			pOP-CEO03451_EST_C_1_pSK_SK	273
			pOP-CEO03455_EST_C_1_pSK_SK	294
			pOP-CEO03456_EST_C_1_pSK_SK	322
			pOP-CEO03457_EST_C_1_pSK_SK	465
			pOP-CEO03459_EST_C_1_pSK_SK	559
			pOP-CEO03464_EST_C_1_pSK_SK	146
			pOP-CEO03465_EST_C_1_pSK_SK	151
			pOP-CEO03469_EST_C_1_pSK_SK	231
			pOP-CEO03473_EST_C_1_pSK_SK	218
			pOP-CEO03480_EST_C_1_pSK_SK	491
			pOP-CEO03485_EST_C_1_pSK_SK	457
			pOP-CEO03489_EST_C_1_pSK_SK	276
			pOP-CEO03503_EST_C_1_pSK_SK	129
			pOP-CEO03505_EST_C_1_pSK_SK	620
			pOP-CEO03510_EST_C_1_pSK_SK	324
			pOP-CEO03515_EST_C_1_pSK_SK	184
			pOP-CEO03516_EST_C_1_pSK_SK	293
			pOP-CEO03520_EST_C_1_pSK_SK	298
			pOP-CEO03521_EST_C_1_pSK_SK	198
			pOP-CEO03525_EST_C_1_pSK_SK	365
			pOP-CEO03528_EST_C_1_pSK_SK	552
			pOP-CEO03532_EST_C_1_pSK_SK	351
			pOP-CEO03541_EST_C_1_pSK_SK	630
			pOP-CEO03546_EST_C_1_pSK_SK	223
			pOP-CEO03551_EST_C_1_pSK_SK	653
			pOP-CEO03554_EST_C_1_pSK_SK	253
			pOP-CEO03560_EST_C_1_pSK_SK	327
			pOP-CEO03562_EST_C_1_pSK_SK	352
			pOP-CEO03569_EST_C_1_pSK_SK	159
			pOP-CEO03572_EST_C_1_pSK_SK	227
			pOP-CEO03574_EST_C_1_pSK_SK	145

			pOP-CEO03580_EST_C_1_pSK_SK	469
			pOP-CEO03582_EST_C_1_pSK_SK	555
			pOP-CEO03583_EST_C_1_pSK_SK	380
			pOP-CEO03586_EST_C_1_pSK_SK	366
			pOP-CEO03590_EST_C_1_pSK_SK	451
			pOP-CEO03593_EST_C_1_pSK_SK	393
			pOP-CEO03598_EST_C_1_pSK_SK	599
			pOP-CEO03600_EST_C_1_pSK_SK	122
			pOP-CEO03603_EST_C_1_pSK_SK	472
			pOP-CEO03609_EST_C_1_pSK_SK	199
			pOP-CEO03612_EST_C_1_pSK_SK	204
			pOP-CEO03620_EST_C_1_pSK_SK	201
			pOP-CEO03622_EST_C_1_pSK_SK	364
			pOP-CEO03628_EST_C_1_pSK_SK	146
			pOP-CEO03631_EST_C_1_pSK_SK	545
			pOP-CEO03643_EST_C_1_pSK_SK	356
			pOP-CEO03644_EST_C_1_pSK_SK	407
			pOP-CEO03649_EST_C_1_pSK_SK	488
			pOP-CEO03650_EST_C_1_pSK_SK	388
			pOP-CEO03659_EST_C_1_pSK_SK	219
			pOP-CEO03661_EST_C_1_pSK_SK	320
			pOP-CEO03663_EST_C_1_pSK_SK	289
			pOP-CEO03665_EST_C_1_pSK_SK	270
			pOP-CEO03668_EST_C_1_pSK_SK	267
			pOP-CEO03672_EST_C_1_pSK_SK	193
			pOP-CEO03673_EST_C_1_pSK_SK	201
			pOP-CEO03674_EST_C_1_pSK_SK	334
			pOP-CEO03675_EST_C_1_pSK_SK	233
			pOP-CEO03677_EST_C_1_pSK_SK	189
			pOP-CEO03682_EST_C_1_pSK_SK	331
			pOP-CEO03684_EST_C_1_pSK_SK	284
			pOP-CEO03689_EST_C_1_pSK_SK	280
			pOP-CEO03692_EST_C_1_pSK_SK	370
			pOP-CEO03693_EST_C_1_pSK_SK	203
			pOP-CEO03694_EST_C_1_pSK_SK	259
			pOP-CEO03701_EST_C_1_pSK_SK	234
			pOP-CEO03706_EST_C_1_pSK_SK	241
			pOP-CEO03709_EST_C_1_pSK_SK	103
			pOP-CEO03715_EST_C_1_pSK_SK	522
			pOP-CEO03716_EST_C_1_pSK_SK	144
			pOP-CEO03717_EST_C_1_pSK_SK	463
			pOP-CEO03719_EST_C_1_pSK_SK	300
			pOP-CEO03720_EST_C_1_pSK_SK	487
			pOP-CEO03723_EST_C_1_pSK_SK	243
			pOP-CEO03724_EST_C_1_pSK_SK	377
			pOP-CEO03725_EST_C_1_pSK_SK	449
			pOP-CEO03729_EST_C_1_pSK_SK	191
			pOP-CEO03739_EST_C_1_pSK_SK	240
			pOP-CEO03742_EST_C_1_pSK_SK	490
			pOP-CEO03743_EST_C_1_pSK_SK	385
			pOP-CEO03745_EST_C_1_pSK_SK	272
			pOP-CEO03747_EST_C_1_pSK_SK	198
			pOP-CEO03748_EST_C_1_pSK_SK	431
			pOP-CEO03749_EST_C_1_pSK_SK	339
			pOP-CEO03752_EST_C_1_pSK_SK	510
			pOP-CEO03757_EST_C_1_pSK_SK	291

			pOP-CEO03759_EST_C_1_pSK_SK	322
			pOP-CEO03767_EST_C_1_pSK_SK	585
			pOP-CEO03770_EST_C_1_pSK_SK	241
			pOP-CEO03773_EST_C_1_pSK_SK	535
			pOP-CEO03774_EST_C_1_pSK_SK	136
			pOP-CEO03776_EST_C_1_pSK_SK	595
			pOP-CEO03777_EST_C_1_pSK_SK	282
			pOP-CEO03781_EST_C_1_pSK_SK	338
			pOP-CEO03783_EST_C_1_pSK_SK	163
			pOP-CEO03784_EST_C_1_pSK_SK	392
			pOP-CEO03787_EST_C_1_pSK_SK	360
			pOP-CEO03789_EST_C_1_pSK_SK	432
			pOP-CEO03792_EST_C_1_pSK_SK	400
			pOP-CEO03795_EST_C_1_pSK_SK	379
			pOP-CEOP00003_EST_C_1_pSK_SK	131
			pOP-CEOP00004_EST_C_1_pSK_SK	341
			pOP-CEOP00006_EST_C_1_pSK_SK	135
			pOP-CEOP00021_EST_C_1_pSK_SK	299
			pOP-CEOP00025_EST_C_1_pSK_SK	386
			pOP-CEOP00030_EST_C_1_pSK_SK	358
			pOP-CEOP00034_EST_C_1_pSK_SK	389
			pOP-CEOP00044_EST_C_1_pSK_SK	605
			pOP-CEOP00047_EST_C_1_pSK_SK	551
			pOP-CEOP00049_EST_C_1_pSK_SK	265
			pOP-CEOP00058_EST_C_1_pSK_SK	375
			pOP-CNH00548_EST_C_1_pSK_SK	599
			pOP-CNH00549_EST_C_1_pSK_SK	368
			pOP-CNH00556_EST_C_1_pSK_SK	618
			pOP-CNH00557_EST_C_1_pSK_SK	661
			pOP-CNH00558_EST_C_1_pSK_SK	444
			pOP-CNH00562_EST_C_1_pSK_SK	656
			pOP-CNH00567_EST_C_1_pSK_SK	650
			pOP-CNH00568_EST_C_1_pSK_SK	657
			pOP-CNH00569_EST_C_1_pSK_SK	665
			pOP-CNH00572_EST_C_1_pSK_SK	592
			pOP-CNH00576_EST_C_1_pSK_SK	663
			pOP-CNH00583_EST_C_1_pSK_SK	647
			pOP-CNH00586_EST_C_1_pSK_SK	662
			pOP-CNH00587_EST_C_1_pSK_SK	552
			pOP-CNH00588_EST_C_1_pSK_SK	580
			pOP-CNH00592_EST_C_1_pSK_SK	610
			pOP-CNH00603_EST_C_1_pSK_SK	605
			pOP-CNH00605_EST_C_1_pSK_SK	556
			pOP-CNH00606_EST_C_1_pSK_SK	608
			pOP-CNH00609_EST_C_1_pSK_SK	286
			pOP-CNH00617_EST_C_1_pSK_SK	530
			pOP-CNH00624_EST_C_1_pSK_SK	518
			pOP-CNH00625_EST_C_1_pSK_SK	721
			pOP-CNH00627_EST_C_1_pSK_SK	586
			pOP-CNH00628_EST_C_1_pSK_SK	691
			pOP-CNH00629_EST_C_1_pSK_SK	721
			pOP-CNH00631_EST_C_1_pSK_SK	717
			pOP-CNH00633_EST_C_1_pSK_SK	651
			pOP-CNH00635_EST_C_1_pSK_SK	517
			pOP-CNH00637_EST_C_1_pSK_SK	610
			pOP-CNH00638_EST_C_1_pSK_SK	618

			pOP-CNH00641_EST_C_1_pSK_SK	403
			pOP-CNH00642_EST_C_1_pSK_SK	508
			pOP-CNH00643_EST_C_1_pSK_SK	594
			pOP-CNH00644_EST_C_1_pSK_SK	543
			pOP-CNH00646_EST_C_1_pSK_SK	467
			pOP-CNH00648_EST_C_1_pSK_SK	520
			pOP-CNH00650_EST_C_1_pSK_SK	350
			pOP-CNH00656_EST_C_1_pSK_SK	592
			pOP-CNH00660_EST_C_1_pSK_SK	602
			pOP-CNH00661_EST_C_1_pSK_SK	634
			pOP-CNH00667_EST_C_1_pSK_SK	525
			pOP-CNH00673_EST_C_1_pSK_SK	594
			pOP-CNH00679_EST_C_1_pSK_SK	573
			pOP-CNH00680_EST_C_1_pSK_SK	428
			pOP-CNH00683_EST_C_1_pSK_SK	625
			pOP-CNH00684_EST_C_1_pSK_SK	294
			pOP-CNH00686_EST_C_1_pSK_SK	575
			pOP-CNH00695_EST_C_1_pSK_SK	592
			pOP-CNH00696_EST_C_1_pSK_SK	710
			pOP-CNH00712_EST_C_1_pSK_SK	570
			pOP-CNH00714_EST_C_1_pSK_SK	610
			pOP-CNH00722_EST_C_1_pSK_SK	302
			pOP-CNH00725_EST_C_1_pSK_SK	534
			pOP-CNH00727_EST_C_1_pSK_SK	680
			pOP-CNH00730_EST_C_1_pSK_SK	554
			pOP-CNH00735_EST_C_1_pSK_SK	529
			pOP-CNH00737_EST_C_1_pSK_SK	327
			pOP-CNH00738_EST_C_1_pSK_SK	418
			pOP-CNH00739_EST_C_1_pSK_SK	106
			pOP-CNH00741_EST_C_1_pSK_SK	546
			pOP-CNH00744_EST_C_1_pSK_SK	455
			pOP-CNH00748_EST_C_1_pSK_SK	472
			pOP-CNH00749_EST_C_1_pSK_SK	495
			pOP-CNH00750_EST_C_1_pSK_SK	601
			pOP-CNH00755_EST_C_1_pSK_SK	479
			pOP-CNH00764_EST_C_1_pSK_SK	485
			pOP-CNH00770_EST_C_1_pSK_SK	380
			pOP-CNH00773_EST_C_1_pSK_SK	494
			pOP-CNH00774_EST_C_1_pSK_SK	477
			pOP-CNH00778_EST_C_1_pSK_SK	412
			pOP-CNH00783_EST_C_1_pSK_SK	374
			pOP-CNH00785_EST_C_1_pSK_SK	449
			pOP-CNH00794_EST_C_1_pSK_SK	671
			pOP-CNH00796_EST_C_1_pSK_SK	525
			pOP-CNH00802_EST_C_1_pSK_SK	400
			pOP-CNH00804_EST_C_1_pSK_SK	590
			pOP-CNH00806_EST_C_1_pSK_SK	482
			pOP-CNH00810_EST_C_1_pSK_SK	377
			pOP-CNH00813_EST_C_1_pSK_SK	538
			pOP-CNH00818_EST_C_1_pSK_SK	365
			pOP-CNH00821_EST_C_1_pSK_SK	707
			pOP-CNH00823_EST_C_1_pSK_SK	608
			pOP-CNH00824_EST_C_1_pSK_SK	440
			pOP-CNH00830_EST_C_1_pSK_SK	568
			pOP-CNH00831_EST_C_1_pSK_SK	455
			pOP-CNH00838_EST_C_1_pSK_SK	579

			pOP-CNH00840_EST_C_1_pSK_SK	558
			pOP-CNH00850_EST_C_1_pSK_SK	506
			pOP-CNH00853_EST_C_1_pSK_SK	273
			pOP-CNH00855_EST_C_1_pSK_SK	546
			pOP-CNH00860_EST_C_1_pSK_SK	674
			pOP-CNH00864_EST_C_1_pSK_SK	621
			pOP-CNH00867_EST_C_1_pSK_SK	709
			pOP-CNH00868_EST_C_1_pSK_SK	607
			pOP-CNH00872_EST_C_1_pSK_SK	586
			pOP-CNH00876_EST_C_1_pSK_SK	606
			pOP-CNH00878_EST_C_1_pSK_SK	739
			pOP-CNH00879_EST_C_1_pSK_SK	541
			pOP-CNH00881_EST_C_1_pSK_SK	307
			pOP-CNH00883_EST_C_1_pSK_SK	279
			pOP-CNH00885_EST_C_1_pSK_SK	489
			pOP-CNH00891_EST_C_1_pSK_SK	507
			pOP-CNH00895_EST_C_1_pSK_SK	433
			pOP-CNH00897_EST_C_1_pSK_SK	521
			pOP-CNH00899_EST_C_1_pSK_SK	425
			pOP-CNH00901_EST_C_1_pSK_SK	445
			pOP-CNH00902_EST_C_1_pSK_SK	517
			pOP-CNH00904_EST_C_1_pSK_SK	427
			pOP-CNH00911_EST_C_1_pSK_SK	636
			pOP-CNH00914_EST_C_1_pSK_SK	521
			pOP-CNH00918_EST_C_1_pSK_SK	534
			pOP-CNH00919_EST_C_1_pSK_SK	551
			pOP-CNH00921_EST_C_1_pSK_SK	541
			pOP-CNH00927_EST_C_1_pSK_SK	425
			pOP-CNH00928_EST_C_1_pSK_SK	372
			pOP-CNH00932_EST_C_1_pSK_SK	374
			pOP-CNH00937_EST_C_1_pSK_SK	323
			pOP-CNH00940_EST_C_1_pSK_SK	439
			pOP-CNH00943_EST_C_1_pSK_SK	542
			pOP-CNH00944_EST_C_1_pSK_SK	590
			pOP-CNH00946_EST_C_1_pSK_SK	377
			pOP-CNH00958_EST_C_1_pSK_SK	620
			pOP-CNH00960_EST_C_1_pSK_SK	227
			pOP-CNH00965_EST_C_1_pSK_SK	758
			pOP-CNH00967_EST_C_1_pSK_SK	702
			pOP-CNH00968_EST_C_1_pSK_SK	437
			pOP-CNH00972_EST_C_1_pSK_SK	615
			pOP-CNH00981_EST_C_1_pSK_SK	526
			pOP-CNH00984_EST_C_1_pSK_SK	308
			pOP-CNH00986_EST_C_1_pSK_SK	207
			pOP-CNH00990_EST_C_1_pSK_SK	595
			pOP-CNH00991_EST_C_1_pSK_SK	585
			pOP-CNH00994_EST_C_1_pSK_SK	547
			pOP-CNH01000_EST_C_1_pSK_SK	335
			pOP-CNH01002_EST_C_1_pSK_SK	279
			pOP-CNH01005_EST_C_1_pSK_SK	225
			pOP-CNH01009_EST_C_1_pSK_SK	268
			pOP-CNH01011_EST_C_1_pSK_SK	332
			pOP-CNH01012_EST_C_1_pSK_SK	315
			pOP-CNH01013_EST_C_1_pSK_SK	255
			pOP-CNH01015_EST_C_1_pSK_SK	269
			pOP-CNH01017_EST_C_1_pSK_SK	297

			pOP-CNH01028_EST_C_1_pSK_SK	240
			pOP-CNH01034_EST_C_1_pSK_SK	168
			pOP-CNH01038_EST_C_1_pSK_SK	553
			pOP-CNH01039_EST_C_1_pSK_SK	327
			pOP-CNH01040_EST_C_1_pSK_SK	433
			pOP-CNH01053_EST_C_1_pSK_SK	673
			pOP-CNH01055_EST_C_1_pSK_SK	653
			pOP-CNH01057_EST_C_1_pSK_SK	618
			pOP-CNH01060_EST_C_1_pSK_SK	573
			pOP-CNH01062_EST_C_1_pSK_SK	395
			pOP-CNH01069_EST_C_1_pSK_SK	609
			pOP-CNH01072_EST_C_1_pSK_SK	343
			pOP-CNH01073_EST_C_1_pSK_SK	627
			pOP-CNH01075_EST_C_1_pSK_SK	648
			pOP-CNH01076_EST_C_1_pSK_SK	625
			pOP-CNH01080_EST_C_1_pSK_SK	617
			pOP-CNH01081_EST_C_1_pSK_SK	542
			pOP-CNH01082_EST_C_1_pSK_SK	681
			pOP-CNH01085_EST_C_1_pSK_SK	571
			pOP-CNH01089_EST_C_1_pSK_SK	596
			pOP-CNH01095_EST_C_1_pSK_SK	472
			pOP-CNH01097_EST_C_1_pSK_SK	428
			pOP-CNH01112_EST_C_1_pSK_SK	325
			pOP-CNH01117_EST_C_1_pSK_SK	246
			pOP-CNH01120_EST_C_1_pSK_SK	260
			pOP-CNH01123_EST_C_1_pSK_SK	372
			pOP-CNH01126_EST_C_1_pSK_SK	386
			pOP-CNH01129_EST_C_1_pSK_SK	437
			pOP-CNH01135_EST_C_1_pSK_SK	629
			pOP-CNH01146_EST_C_1_pSK_SK	512
			pOP-CNH01148_EST_C_1_pSK_SK	591
			pOP-CNH01149_EST_C_1_pSK_SK	384
			pOP-CNH01158_EST_C_1_pSK_SK	406
			pOP-CNH01160_EST_C_1_pSK_SK	388
			pOP-CNH01161_EST_C_1_pSK_SK	419
			pOP-CNH01163_EST_C_1_pSK_SK	443
			pOP-CNH01170_EST_C_1_pSK_SK	598
			pOP-CNH01173_EST_C_1_pSK_SK	338
			pOP-CNH01174_EST_C_1_pSK_SK	525
			pOP-CNH01179_EST_C_1_pSK_SK	599
			pOP-CNH01182_EST_C_1_pSK_SK	574
			pOP-CNH01184_EST_C_1_pSK_SK	621
			pOP-CNH01186_EST_C_1_pSK_SK	390
			pOP-CNH01190_EST_C_1_pSK_SK	551
			pOP-CNH01192_EST_C_1_pSK_SK	607
			pOP-CNH01201_EST_C_1_pSK_SK	596
			pOP-CNH01207_EST_C_1_pSK_SK	588
			pOP-CNH01209_EST_C_1_pSK_SK	258
			pOP-CNH01212_EST_C_1_pSK_SK	605
			pOP-CNH01213_EST_C_1_pSK_SK	563
			pOP-CNH01214_EST_C_1_pSK_SK	453
			pOP-CNH01216_EST_C_1_pSK_SK	551
			pOP-CNH01226_EST_C_1_pSK_SK	565
			pOP-CNH01229_EST_C_1_pSK_SK	597
			pOP-CNH01234_EST_C_1_pSK_SK	581
			pOP-CNH01240_EST_C_1_pSK_SK	637

			pOP-CNH01242_EST_C_1_pSK_SK	282
			pOP-CNH01243_EST_C_1_pSK_SK	532
			pOP-CNH01248_EST_C_1_pSK_SK	577
			pOP-CNH01250_EST_C_1_pSK_SK	635
			pOP-CNH01257_EST_C_1_pSK_SK	524
			pOP-CNH01267_EST_C_1_pSK_SK	237
			pOP-CNH01269_EST_C_1_pSK_SK	574
			pOP-CNH01272_EST_C_1_pSK_SK	642
			pOP-CNH01281_EST_C_1_pSK_SK	610
			pOP-CNH01286_EST_C_1_pSK_SK	430
			pOP-CNH01287_EST_C_1_pSK_SK	565
			pOP-CNH01289_EST_C_1_pSK_SK	480
			pOP-CNH01295_EST_C_1_pSK_SK	605
			pOP-CNH01301_EST_C_1_pSK_SK	676
			pOP-CNH01302_EST_C_1_pSK_SK	594
			pOP-CNH01307_EST_C_1_pSK_SK	572
			pOP-CNH01309_EST_C_1_pSK_SK	462
			pOP-CNH01318_EST_C_1_pSK_SK	680
			pOP-CNH01353_EST_C_1_pSK_SK	592
			pOP-CNH01357_EST_C_1_pSK_SK	355
			pOP-CNH01365_EST_C_1_pSK_SK	442
			pOP-CNH01372_EST_C_1_pSK_SK	627
			pOP-CNH01379_EST_C_1_pSK_SK	583
			pOP-CNH01380_EST_C_1_pSK_SK	783
			pOP-CNH01381_EST_C_1_pSK_SK	760
			pOP-CNH01382_EST_C_1_pSK_SK	665
			pOP-CNH01387_EST_C_1_pSK_SK	515
			pOP-CNH01396_EST_C_1_pSK_SK	517
			pOP-CNH01399_EST_C_1_pSK_SK	566
			pOP-CNH01400_EST_C_1_pSK_SK	449
			pOP-CNH01408_EST_C_1_pSK_SK	500
			pOP-CNH01421_EST_C_1_pSK_SK	613
			pOP-CNH01427_EST_C_1_pSK_SK	504
			pOP-CNH01430_EST_C_1_pSK_SK	520
			pOP-CNH01432_EST_C_1_pSK_SK	620
			pOP-CNH01443_EST_C_1_pSK_SK	645
			pOP-CNH01445_EST_C_1_pSK_SK	450
			pOP-CNH01452_EST_C_1_pSK_SK	566
			pOP-CNH01455_EST_C_1_pSK_SK	617
			pOP-CNH01458_EST_C_1_pSK_SK	642
			pOP-CNH01459_EST_C_1_pSK_SK	588
			pOP-CNH01461_EST_C_1_pSK_SK	459
			pOP-CNH01462_EST_C_1_pSK_SK	326
			pOP-CNH01472_EST_C_1_pSK_SK	375
			pOP-CNH01478_EST_C_1_pSK_SK	631
			pOP-CNH01482_EST_C_1_pSK_SK	531
			pOP-CNH01485_EST_C_1_pSK_SK	576
			pOP-CNH01489_EST_C_1_pSK_SK	443
			pOP-CNH01490_EST_C_1_pSK_SK	615
			pOP-CNH01491_EST_C_1_pSK_SK	760
			pOP-CNH01509_EST_C_1_pSK_SK	522
			pOP-CNH01511_EST_C_1_pSK_SK	546
			pOP-CNH01515_EST_C_1_pSK_SK	502
			pOP-CNH01521_EST_C_1_pSK_SK	506
			pOP-CNH01524_EST_C_1_pSK_SK	518
			pOP-CNH01528_EST_C_1_pSK_SK	496

			pOP-CNH01531_EST_C_1_pSK_SK	514
			pOP-CNH01533_EST_C_1_pSK_SK	600
			pOP-CNH01535_EST_C_1_pSK_SK	510
			pOP-CNH01537_EST_C_1_pSK_SK	572
			pOP-CNH01539_EST_C_1_pSK_SK	477
			pOP-CNH01540_EST_C_1_pSK_SK	220
			pOP-CNH01544_EST_C_1_pSK_SK	468
			pOP-CNH01548_EST_C_1_pSK_SK	129
			pOP-CNH01549_EST_C_1_pSK_SK	379
			pOP-CNH01557_EST_C_1_pSK_SK	697
			pOP-CNH01568_EST_C_1_pSK_SK	573
			pOP-CNH01569_EST_C_1_pSK_SK	751
			pOP-CNH01570_EST_C_1_pSK_SK	750
			pOP-CNH01577_EST_C_1_pSK_SK	827
			pOP-CNH01583_EST_C_1_pSK_SK	699
			pOP-CNH01596_EST_C_1_pSK_SK	472
			pOP-CNH01598_EST_C_1_pSK_SK	499
			pOP-CNH01599_EST_C_1_pSK_SK	488
			pOP-CNH01602_EST_C_1_pSK_SK	469
			pOP-CNH01603_EST_C_1_pSK_SK	565
			pOP-CNH01608_EST_C_1_pSK_SK	416
			pOP-CNH01619_EST_C_1_pSK_SK	618
			pOP-CNH01621_EST_C_1_pSK_SK	498
			pOP-CNH01623_EST_C_1_pSK_SK	292
			pOP-CNH01631_EST_C_1_pSK_SK	673
			pOP-CNH01633_EST_C_1_pSK_SK	675
			pOP-CNH01637_EST_C_1_pSK_SK	223
			pOP-CNH01642_EST_C_1_pSK_SK	615
			pOP-CNH01645_EST_C_1_pSK_SK	580
			pOP-CNH01659_EST_C_1_pSK_SK	523
			pOP-CNH01662_EST_C_1_pSK_SK	616
			pOP-CNH01663_EST_C_1_pSK_SK	599
			pOP-CNH01665_EST_C_1_pSK_SK	575
			pOP-CNH01667_EST_C_1_pSK_SK	709
			pOP-CNH01668_EST_C_1_pSK_SK	312
			pOP-CNH01669_EST_C_1_pSK_SK	652
			pOP-CNH01676_EST_C_1_pSK_SK	496
			pOP-CNH01685_EST_C_1_pSK_SK	570
			pOP-CNH01686_EST_C_1_pSK_SK	721
			pOP-CNH01689_EST_C_1_pSK_SK	475
			pOP-CNH01691_EST_C_1_pSK_SK	618
			pOP-CNH01692_EST_C_1_pSK_SK	427
			pOP-CNH01697_EST_C_1_pSK_SK	546
			pOP-CNH01698_EST_C_1_pSK_SK	529
			pOP-CNH01703_EST_C_1_pSK_SK	664
			pOP-CNH01707_EST_C_1_pSK_SK	614
			pOP-CNH01718_EST_C_1_pSK_SK	382
			pOP-CNH01723_EST_C_1_pSK_SK	479
			pOP-CNH01726_EST_C_1_pSK_SK	458
			pOP-CNH01727_EST_C_1_pSK_SK	554
			pOP-CNH01730_EST_C_1_pSK_SK	541
			pOP-CNH01734_EST_C_1_pSK_SK	552
			pOP-CNH01739_EST_C_1_pSK_SK	482
			pOP-CNH01745_EST_C_1_pSK_SK	570
			pOP-CNH01751_EST_C_1_pSK_SK	540
			pOP-CNH01753_EST_C_1_pSK_SK	386

			pOP-CNH01754_EST_C_1_pSK_SK	524
			pOP-CNH01755_EST_C_1_pSK_SK	542
			pOP-CNH01762_EST_C_1_pSK_SK	538
			pOP-CNH01769_EST_C_1_pSK_SK	511
			pOP-CNH01772_EST_C_1_pSK_SK	509
			pOP-CNH01781_EST_C_1_pSK_SK	575
			pOP-CNH01787_EST_C_1_pSK_SK	434
			pOP-CNH01790_EST_C_1_pSK_SK	659
			pOP-CNH01792_EST_C_1_pSK_SK	374
			pOP-CNH01794_EST_C_1_pSK_SK	568
			pOP-CNH01795_EST_C_1_pSK_SK	697
			pOP-CNH01800_EST_C_1_pSK_SK	515
			pOP-CNH01803_EST_C_1_pSK_SK	514
			pOP-CNH01804_EST_C_1_pSK_SK	500
			pOP-CNH01805_EST_C_1_pSK_SK	511
			pOP-CNH01807_EST_C_1_pSK_SK	483
			pOP-CNH01815_EST_C_1_pSK_SK	440
			pOP-CNH01827_EST_C_1_pSK_SK	486
			pOP-CNH01828_EST_C_1_pSK_SK	518
			pOP-CNH01835_EST_C_1_pSK_SK	451
			pOP-CNH01838_EST_C_1_pSK_SK	490
			pOP-CNH01839_EST_C_1_pSK_SK	487
			pOP-CNH01840_EST_C_1_pSK_SK	465
			pOP-CNH01846_EST_C_1_pSK_SK	538
			pOP-CNH01853_EST_C_1_pSK_SK	472
			pOP-CNH01856_EST_C_1_pSK_SK	483
			pOP-CNH01858_EST_C_1_pSK_SK	447
			pOP-CNH01866_EST_C_1_pSK_SK	471
			pOP-CNH01874_EST_C_1_pSK_SK	413
			pOP-CNH01879_EST_C_1_pSK_SK	422
			pOP-CNH01882_EST_C_1_pSK_SK	400
			pOP-CNH01887_EST_C_1_pSK_SK	507
			pOP-CNH01912_EST_C_1_pSK_SK	627
			pOP-CNH01919_EST_C_1_pSK_SK	744
			pOP-CNH01927_EST_C_1_pSK_SK	472
			pOP-CNH01930_EST_C_1_pSK_SK	181
			pOP-CNH01942_EST_C_1_pSK_SK	665
			pOP-CNH01944_EST_C_1_pSK_SK	591
			pOP-CNH01954_EST_C_1_pSK_SK	631
			pOP-CNH01955_EST_C_1_pSK_SK	771
			pOP-CNH01959_EST_C_1_pSK_SK	351
			pOP-CNH01960_EST_C_1_pSK_SK	672
			pOP-CNH01961_EST_C_1_pSK_SK	506
			pOP-CNH01962_EST_C_1_pSK_SK	509
			pOP-CNH01964_EST_C_1_pSK_SK	280
			pOP-CNH01965_EST_C_1_pSK_SK	719
			pOP-CNH01968_EST_C_1_pSK_SK	657
			pOP-CNH01970_EST_C_1_pSK_SK	559
			pOP-CNH01974_EST_C_1_pSK_SK	593
			pOP-CNH01990_EST_C_1_pSK_SK	568
			pOP-CNH01992_EST_C_1_pSK_SK	515
			pOP-CNH02002_EST_C_1_pSK_SK	548
			pOP-CNH02003_EST_C_1_pSK_SK	554
			pOP-CNH02004_EST_C_1_pSK_SK	385
			pOP-CNH02008_EST_C_1_pSK_SK	420
			pOP-CNH02012_EST_C_1_pSK_SK	464

			pOP-CNH02016_EST_C_1_pSK_SK	575
			pOP-CNH02018_EST_C_1_pSK_SK	584
			pOP-CNH02024_EST_C_1_pSK_SK	514
			pOP-CNH02029_EST_C_1_pSK_SK	410
			pOP-CNH02036_EST_C_1_pSK_SK	508
			pOP-CNH02043_EST_C_1_pSK_SK	512
			pOP-CNH02047_EST_C_1_pSK_SK	536
			pOP-CNH02057_EST_C_1_pSK_SK	403
			pOP-CNH02058_EST_C_1_pSK_SK	545
			pOP-CNH02065_EST_C_1_pSK_SK	534
			pOP-CNH02066_EST_C_1_pSK_SK	437
			pOP-CNH02067_EST_C_1_pSK_SK	524
			pOP-CNH02079_EST_C_1_pSK_SK	548
			pOP-CNH02092_EST_C_1_pSK_SK	503
			pOP-CNH02099_EST_C_1_pSK_SK	440
			pOP-CNH02102_EST_C_1_pSK_SK	505
			pOP-CNH02115_EST_C_1_pSK_SK	546
			pOP-CNH02124_EST_C_1_pSK_SK	448
			pOP-CNH02131_EST_C_1_pSK_SK	587
			pOP-CNH02138_EST_C_1_pSK_SK	561
			pOP-CNH02141_EST_C_1_pSK_SK	573
			pOP-CNH02143_EST_C_1_pSK_SK	237
			pOP-CNH02154_EST_C_1_pSK_SK	551
			pOP-CNH02158_EST_C_1_pSK_SK	601
			pOP-CNH02160_EST_C_1_pSK_SK	586
			pOP-CNH02162_EST_C_1_pSK_SK	468
			pOP-CNH02167_EST_C_1_pSK_SK	487
			pOP-CNH02169_EST_C_1_pSK_SK	565
			pOP-CNH02173_EST_C_1_pSK_SK	633
			pOP-CNH02174_EST_C_1_pSK_SK	578
			pOP-CNH02181_EST_C_1_pSK_SK	522
			pOP-CNH02189_EST_C_1_pSK_SK	695
			pOP-CNH02194_EST_C_1_pSK_SK	588
			pOP-CNH02199_EST_C_1_pSK_SK	471
			pOP-CNH02203_EST_C_1_pSK_SK	677
			pOP-CNH02210_EST_C_1_pSK_SK	567
			pOP-CNH02212_EST_C_1_pSK_SK	505
			pOP-CNH02219_EST_C_1_pSK_SK	701
			pOP-CNH02228_EST_C_1_pSK_SK	716
			pOP-CNH02230_EST_C_1_pSK_SK	772
			pOP-CNH02231_EST_C_1_pSK_SK	723
			pOP-CNH02233_EST_C_1_pSK_SK	668
			pOP-CNH02234_EST_C_1_pSK_SK	596
			pOP-CNH02235_EST_C_1_pSK_SK	640
			pOP-CNH02237_EST_C_1_pSK_SK	700
			pOP-CNH02238_EST_C_1_pSK_SK	724
			pOP-CNH02240_EST_C_1_pSK_SK	677
			pOP-CNH02244_EST_C_1_pSK_SK	686
			pOP-CNH02245_EST_C_1_pSK_SK	769
			pOP-CNH02247_EST_C_1_pSK_SK	304
			pOP-CNH02248_EST_C_1_pSK_SK	673
			pOP-CNH02250_EST_C_1_pSK_SK	689
			pOP-CNH02252_EST_C_1_pSK_SK	641
			pOP-CNH02253_EST_C_1_pSK_SK	665
			pOP-CNH02266_EST_C_1_pSK_SK	698
			pOP-CNH02273_EST_C_1_pSK_SK	655

			pOP-CNH02275_EST_C_1_pSK_SK	678
			pOP-CNH02277_EST_C_1_pSK_SK	596
			pOP-CNH02282_EST_C_1_pSK_SK	506
			pOP-CNH02284_EST_C_1_pSK_SK	475
			pOP-CNH02285_EST_C_1_pSK_SK	697
			pOP-CNH02292_EST_C_1_pSK_SK	599
			pOP-CNH02293_EST_C_1_pSK_SK	522
			pOP-CNH02294_EST_C_1_pSK_SK	676
			pOP-CNH02299_EST_C_1_pSK_SK	634
			pOP-CNH02308_EST_C_1_pSK_SK	648
			pOP-CNH02314_EST_C_1_pSK_SK	589
			pOP-CNH02320_EST_C_1_pSK_SK	585
			pOP-CNH02324_EST_C_1_pSK_SK	389
			pOP-CNH02329_EST_C_1_pSK_SK	468
			pOP-CNH02331_EST_C_1_pSK_SK	544
			pOP-CNH02335_EST_C_1_pSK_SK	584
			pOP-CNH02337_EST_C_1_pSK_SK	624
			pOP-CNH02339_EST_C_1_pSK_SK	565
			pOP-CNH02341_EST_C_1_pSK_SK	420
			pOP-CNH02345_EST_C_1_pSK_SK	395
			pOP-CNH02349_EST_C_1_pSK_SK	631
			pOP-CNH02350_EST_C_1_pSK_SK	610
			pOP-CNH02351_EST_C_1_pSK_SK	664
			pOP-CNH02353_EST_C_1_pSK_SK	631
			pOP-CNH02360_EST_C_1_pSK_SK	611
			pOP-CNH02363_EST_C_1_pSK_SK	525
			pOP-CNH02365_EST_C_1_pSK_SK	511
			pOP-CNH02379_EST_C_1_pSK_SK	513
			pOP-CNH02382_EST_C_1_pSK_SK	519
			pOP-CNH02387_EST_C_1_pSK_SK	528
			pOP-CNH02389_EST_C_1_pSK_SK	518
			pOP-CNH02391_EST_C_1_pSK_SK	386
			pOP-CNH02395_EST_C_1_pSK_SK	445
			pOP-CNH02398_EST_C_1_pSK_SK	511
			pOP-CNH02403_EST_C_1_pSK_SK	528
			pOP-CNH02406_EST_C_1_pSK_SK	522
			pOP-CNH02413_EST_C_1_pSK_SK	322
			pOP-CNH02414_EST_C_1_pSK_SK	411
			pOP-CNH02424_EST_C_1_pSK_SK	505
			pOP-CNH02426_EST_C_1_pSK_SK	171
			pOP-CNH02429_EST_C_1_pSK_SK	534
			pOP-CNH02431_EST_C_1_pSK_SK	506
			pOP-CNH02433_EST_C_1_pSK_SK	484
			pOP-CNH02435_EST_C_1_pSK_SK	189
			pOP-CNH02439_EST_C_1_pSK_SK	535
			pOP-CNH02440_EST_C_1_pSK_SK	230
			pOP-CNH02442_EST_C_1_pSK_SK	536
			pOP-CNH02443_EST_C_1_pSK_SK	372
			pOP-CNH02456_EST_C_1_pSK_SK	521
			pOP-CNH02458_EST_C_1_pSK_SK	428
			pOP-CNH02463_EST_C_1_pSK_SK	416
			pOP-CNH02471_EST_C_1_pSK_SK	383
			pOP-CNH02473_EST_C_1_pSK_SK	316
			pOP-CNH02477_EST_C_1_pSK_SK	532
			pOP-CNH02481_EST_C_1_pSK_SK	386
			pOP-CNH02482_EST_C_1_pSK_SK	340

			pOP-CNH02484_EST_C_1_pSK_SK	287
			pOP-CNH02491_EST_C_1_pSK_SK	234
			pOP-CNH02498_EST_C_1_pSK_SK	462
			pOP-CNH02499_EST_C_1_pSK_SK	444
			pOP-CNH02500_EST_C_1_pSK_SK	393
			pOP-CNH02518_EST_C_1_pSK_SK	480
			pOP-CNH02519_EST_C_1_pSK_SK	518
			pOP-CNH02520_EST_C_1_pSK_SK	521
			pOP-CNH02525_EST_C_1_pSK_SK	465
			pOP-CNH02529_EST_C_1_pSK_SK	491
			pOP-CNH02533_EST_C_1_pSK_SK	503
			pOP-CNH02536_EST_C_1_pSK_SK	400
			pOP-CNH02541_EST_C_1_pSK_SK	551
			pOP-CNH02542_EST_C_1_pSK_SK	541
			pOP-CNH02545_EST_C_1_pSK_SK	539
			pOP-CNH02546_EST_C_1_pSK_SK	545
			pOP-CNH02548_EST_C_1_pSK_SK	538
			pOP-CNH02549_EST_C_1_pSK_SK	549
			pOP-CNH02555_EST_C_1_pSK_SK	658
			pOP-CNH02556_EST_C_1_pSK_SK	547
			pOP-CNH02563_EST_C_1_pSK_SK	550
			pOP-CNH02564_EST_C_1_pSK_SK	600
			pOP-CNH02569_EST_C_1_pSK_SK	549
			pOP-CNH02579_EST_C_1_pSK_SK	545
			pOP-CNH02580_EST_C_1_pSK_SK	374
			pOP-CNH02591_EST_C_1_pSK_SK	467
			pOP-CNH02622_EST_C_1_pSK_SK	559
			pOP-CNH02623_EST_C_1_pSK_SK	489
			pOP-CNH02627_EST_C_1_pSK_SK	564
			pOP-CNH02630_EST_C_1_pSK_SK	536
			pOP-CNH02639_EST_C_1_pSK_SK	511
			pOP-CNH02660_EST_C_1_pSK_SK	522
			pOP-CNH02662_EST_C_1_pSK_SK	608
			pOP-CNH02665_EST_C_1_pSK_SK	638
			pOP-CNH02667_EST_C_1_pSK_SK	590
			pOP-CNH02668_EST_C_1_pSK_SK	640
			pOP-CNH02670_EST_C_1_pSK_SK	676
			pOP-CNH02678_EST_C_1_pSK_SK	647
			pOP-CNH02681_EST_C_1_pSK_SK	614
			pOP-CNH02684_EST_C_1_pSK_SK	333
			pOP-CNH02693_EST_C_1_pSK_SK	503
			pOP-CNH02696_EST_C_1_pSK_SK	571
			pOP-CNH02704_EST_C_1_pSK_SK	633
			pOP-CNH02714_EST_C_1_pSK_SK	528
			pOP-CNH02721_EST_C_1_pSK_SK	403
			pOP-CNH02742_EST_C_1_pSK_SK	672
			pOP-CNH02744_EST_C_1_pSK_SK	601
			pOP-CNH02766_EST_C_1_pSK_SK	644
			pOP-CNH02770_EST_C_1_pSK_SK	572
			pOP-CNH02772_EST_C_1_pSK_SK	589
			pOP-CNH02773_EST_C_1_pSK_SK	715
			pOP-CNH02784_EST_C_1_pSK_SK	622
			pOP-CNH02793_EST_C_1_pSK_SK	568
			pOP-CNH02795_EST_C_1_pSK_SK	581
			pOP-CNH02797_EST_C_1_pSK_SK	643
			pOP-CNH02808_EST_C_1_pSK_SK	432

			pOP-CNH02815_EST_C_1_pSK_SK	344
			pOP-CNH02816_EST_C_1_pSK_SK	398
			pOP-CNH02817_EST_C_1_pSK_SK	530
			pOP-CNH02819_EST_C_1_pSK_SK	532
			pOP-CNH02825_EST_C_1_pSK_SK	661
			pOP-CNH02827_EST_C_1_pSK_SK	585
			pOP-CNH02832_EST_C_1_pSK_SK	600
			pOP-CNH02833_EST_C_1_pSK_SK	594
			pOP-CNH02835_EST_C_1_pSK_SK	615
			pOP-CNH02838_EST_C_1_pSK_SK	777
			pOP-CNH02844_EST_C_1_pSK_SK	599
			pOP-CNH02846_EST_C_1_pSK_SK	567
			pOP-CNH02852_EST_C_1_pSK_SK	648
			pOP-CNH02865_EST_C_1_pSK_SK	638
			pOP-CNH02866_EST_C_1_pSK_SK	460
			pOP-CNH02867_EST_C_1_pSK_SK	582
			pOP-CNH02868_EST_C_1_pSK_SK	666
			pOP-CNH02870_EST_C_1_pSK_SK	464
			pOP-CNH02871_EST_C_1_pSK_SK	709
			pOP-CNH02872_EST_C_1_pSK_SK	555
			pOP-CNH02876_EST_C_1_pSK_SK	624
			pOP-CNH02880_EST_C_1_pSK_SK	567
			pOP-CNH02883_EST_C_1_pSK_SK	537
			pOP-CNH02893_EST_C_1_pSK_SK	633
			pOP-CNH02898_EST_C_1_pSK_SK	535
			pOP-CNH02902_EST_C_1_pSK_SK	326
			pOP-CNH02903_EST_C_1_pSK_SK	550
			pOP-CNH02904_EST_C_1_pSK_SK	613
			pOP-CNH02911_EST_C_1_pSK_SK	587
			pOP-CNH02922_EST_C_1_pSK_SK	594
			pOP-CNH02924_EST_C_1_pSK_SK	536
			pOP-CNH02927_EST_C_1_pSK_SK	575
			pOP-CNH02932_EST_C_1_pSK_SK	456
			pOP-CNH02938_EST_C_1_pSK_SK	655
			pOP-CNH02939_EST_C_1_pSK_SK	632
			pOP-CNH02956_EST_C_1_pSK_SK	586
			pOP-CNH02958_EST_C_1_pSK_SK	627
			pOP-CNH02963_EST_C_1_pSK_SK	539
			pOP-CNH02965_EST_C_1_pSK_SK	585
			pOP-CNH02970_EST_C_1_pSK_SK	570
			pOP-CNH02981_EST_C_1_pSK_SK	522
			pOP-CNH02983_EST_C_1_pSK_SK	552
			pOP-CNH02988_EST_C_1_pSK_SK	568
			pOP-CNH03013_EST_C_1_pSK_SK	302
			pOP-CNH03016_EST_C_1_pSK_SK	531
			pOP-CNH03018_EST_C_1_pSK_SK	656
			pOP-CNH03020_EST_C_1_pSK_SK	396
			pOP-CNH03021_EST_C_1_pSK_SK	515
			pOP-CNH03023_EST_C_1_pSK_SK	614
			pOP-CNH03026_EST_C_1_pSK_SK	531
			pOP-CNH03028_EST_C_1_pSK_SK	610
			pOP-CNH03030_EST_C_1_pSK_SK	541
			pOP-CNH03043_EST_C_1_pSK_SK	574
			pOP-CNH03063_EST_C_1_pSK_SK	598
			pOP-CNH03070_EST_C_1_pSK_SK	674
			pOP-CNH03074_EST_C_1_pSK_SK	666

			pOP-CNH03075_EST_C_1_pSK_SK	535
			pOP-CNH03081_EST_C_1_pSK_SK	680
			pOP-CNH03093_EST_C_1_pSK_SK	542
			pOP-CNH03098_EST_C_1_pSK_SK	367
			pOP-CNH03108_EST_C_1_pSK_SK	534
			pOP-CNH03113_EST_C_1_pSK_SK	601
			pOP-CNH03116_EST_C_1_pSK_SK	607
			pOP-CNH03127_EST_C_1_pSK_SK	542
			pOP-CNH03129_EST_C_1_pSK_SK	659
			pOP-CNH03133_EST_C_1_pSK_SK	435
			pOP-CNH03140_EST_C_1_pSK_SK	608
			pOP-CNH03149_EST_C_1_pSK_SK	700
			pOP-CNH03152_EST_C_1_pSK_SK	571
			pOP-CNH03159_EST_C_1_pSK_SK	472
			pOP-CNH03181_EST_C_1_pSK_SK	435
			pOP-CNH03228_EST_C_1_pSK_SK	486
			pOP-CNH03244_EST_C_1_pSK_SK	380
			pOP-CNH03245_EST_C_1_pSK_SK	516
			pOP-CNH03255_EST_C_1_pSK_SK	513
			pOP-CNH03257_EST_C_1_pSK_SK	633
			pOP-CNH03264_EST_C_1_pSK_SK	400
			pOP-CNH03266_EST_C_1_pSK_SK	562
			pOP-CNH03267_EST_C_1_pSK_SK	512
			pOP-CNH03275_EST_C_1_pSK_SK	549
			pOP-CNH03283_EST_C_1_pSK_SK	528
			pOP-CNH03284_EST_C_1_pSK_SK	337
			pOP-CNH03294_EST_C_1_pSK_SK	464
			pOP-CNH03300_EST_C_1_pSK_SK	743
			pOP-CNH03301_EST_C_1_pSK_SK	451
			pOP-CNH03319_EST_C_1_pSK_SK	671
			pOP-CNH03323_EST_C_1_pSK_SK	773
			pOP-CNH03325_EST_C_1_pSK_SK	749
			pOP-CNH03342_EST_C_1_pSK_SK	690
			pOP-CNH03352_EST_C_1_pSK_SK	569
			pOP-CNH03353_EST_C_1_pSK_SK	521
			pOP-CNH03360_EST_C_1_pSK_SK	575
			pOP-CNH03363_EST_C_1_pSK_SK	664
			pOP-CNH03369_EST_C_1_pSK_SK	687
			pOP-CNH03378_EST_C_1_pSK_SK	659
			pOP-CNH03384_EST_C_1_pSK_SK	625
			pOP-CNH03391_EST_C_1_pSK_SK	337
			pOP-CNH03399_EST_C_1_pSK_SK	614
			pOP-CNH03405_EST_C_1_pSK_SK	602
			pOP-CNH03406_EST_C_1_pSK_SK	545
			pOP-CNH03417_EST_C_1_pSK_SK	663
			pOP-CNH03431_EST_C_1_pSK_SK	619
			pOP-CNH03439_EST_C_1_pSK_SK	526
			pOP-CNH03442_EST_C_1_pSK_SK	563
			pOP-CNH03445_EST_C_1_pSK_SK	452
			pOP-CNH03446_EST_C_1_pSK_SK	510
			pOP-CNH03448_EST_C_1_pSK_SK	540
			pOP-CNH03454_EST_C_1_pSK_SK	545
			pOP-CNH03455_EST_C_1_pSK_SK	576
			pOP-CNH03468_EST_C_1_pSK_SK	443
			pOP-CNH03475_EST_C_1_pSK_SK	451
			pOP-CNH03487_EST_C_1_pSK_SK	512

			pOP-CNH03489_EST_C_1_pSK_SK	580
			pOP-CNH03493_EST_C_1_pSK_SK	532
			pOP-CNH03505_EST_C_1_pSK_SK	583
			pOP-CNH03519_EST_C_1_pSK_SK	270
			pOP-CNH03522_EST_C_1_pSK_SK	633
			pOP-CNH03533_EST_C_1_pSK_SK	587
			pOP-CNH03537_EST_C_1_pSK_SK	320
			pOP-CNH03538_EST_C_1_pSK_SK	560
			pOP-CNH03544_EST_C_1_pSK_SK	582
			pOP-CNH03551_EST_C_1_pSK_SK	563
			pOP-CNH03552_EST_C_1_pSK_SK	309
			pOP-CNH03563_EST_C_1_pSK_SK	235
			pOP-CNH03572_EST_C_1_pSK_SK	344
			pOP-CNH03587_EST_C_1_pSK_SK	356
			pOP-CNH03590_EST_C_1_pSK_SK	341
			pOP-CNH03593_EST_C_1_pSK_SK	347
			pOP-CNH03595_EST_C_1_pSK_SK	354
			pOP-CNH03626_EST_C_1_pSK_SK	594
			pOP-CNH03628_EST_C_1_pSK_SK	338
			pOP-CNH03634_EST_C_1_pSK_SK	666
			pOP-CNH03635_EST_C_1_pSK_SK	587
			pOP-CNH03639_EST_C_1_pSK_SK	619
			pOP-CNH03644_EST_C_1_pSK_SK	366
			pOP-CNH03645_EST_C_1_pSK_SK	637
			pOP-CNH03646_EST_C_1_pSK_SK	394
			pOP-CNH03647_EST_C_1_pSK_SK	432
			pOP-CNH03656_EST_C_1_pSK_SK	431
			pOP-CNH03657_EST_C_1_pSK_SK	430
			pOP-CNH03663_EST_C_1_pSK_SK	461
			pOP-CNH03670_EST_C_1_pSK_SK	451
			pOP-CNH03700_EST_C_1_pSK_SK	500
			pOP-CNH03703_EST_C_1_pSK_SK	463
			pOP-CNH03708_EST_C_1_pSK_SK	394
			pOP-CNH03714_EST_C_1_pSK_SK	363
			pOP-CNH03724_EST_C_1_pSK_SK	355
			pOP-CNH03725_EST_C_1_pSK_SK	408
			pOP-CNH03728_EST_C_1_pSK_SK	356
			pOP-CNH03730_EST_C_1_pSK_SK	456
			pOP-CNH03740_EST_C_1_pSK_SK	377
			pOP-CNH03743_EST_C_1_pSK_SK	589
			pOP-CNH03745_EST_C_1_pSK_SK	464
			pOP-CNH03752_EST_C_1_pSK_SK	397
			pOP-CNH03757_EST_C_1_pSK_SK	411
			pOP-CNH03758_EST_C_1_pSK_SK	562
			pOP-CNH03762_EST_C_1_pSK_SK	231
			pOP-CNH03763_EST_C_1_pSK_SK	609
			pOP-CNH03766_EST_C_1_pSK_SK	504
			pOP-CNH03769_EST_C_1_pSK_SK	588
			pOP-CNH03780_EST_C_1_pSK_SK	372
			pOP-CNH03787_EST_C_1_pSK_SK	393
			pOP-CNH03788_EST_C_1_pSK_SK	512
			pOP-CNH03799_EST_C_1_pSK_SK	381
			pOP-CNH03803_EST_C_1_pSK_SK	543
			pOP-CNH03806_EST_C_1_pSK_SK	452
			pOP-CNH03808_EST_C_1_pSK_SK	322
			pOP-CNH03809_EST_C_1_pSK_SK	660

			pOP-CNH04137	374
			pOP-CNH04144	612
			pOP-CNH04146	499
			pOP-CNH04150	461
			pOP-CNH04153	379
			pOP-CNH04155	559
			pOP-CNH04156	521
			pOP-CNH04158	549
			pOP-CNH04161	232
			pOP-CNH04163	441
			pOP-CNH04164	524
			pOP-CNH04169	463
			pOP-CNH04177	515
			pOP-CNH04179	443
			pOP-CNH04180	448
			pOP-CNH04183	508
			pOP-CNH04187	490
			pOP-CNH04189	521
			pOP-CNH04192	393
			pOP-CNH04193	335
			pOP-CNH04197	559
			pOP-CNH04198	527
			pOP-CNH04200	640
			pOP-CNH04206	455
			pOP-CNH04207	437
			pOP-CNH04209	301
			pOP-CNH04214	488
			pOP-CNH04220	275
			pOP-CNH04224	614
			pOP-CNH04228	352
			pOP-CNH04238	476
			pOP-CNH04247	370
			pOP-CNH04248	508
			pOP-CNH04252	528
			pOP-CNH04259	385
			pOP-CNH04264	529
			pOP-CNH04269	286
			pOP-CNH04271	410
			pOP-CNH04297	473
			pOP-CNH04300	581
			pOP-CNH04307	588
			pOP-CNH04311	566
			pOP-CNH04315	392
			pOP-CNH04319	526
			pOP-CNH04323	473
			pOP-CNH04326	771
			pOP-CNH04332	786
			pOP-CNH04334	800
			pOP-CNH04338	118
			pOP-CNH04341	808
			pOP-CNH04343	335
			pOP-CNH04346	416
			pOP-CNH04349	432
			pOP-CNH04371	753
			pOP-CNH04375	735
			pOP-CNH04386	871

			pOP-CNH04389	808
			pOP-CNH04396	805
			pOP-CNH04400	696
			pOP-CNH04401	708
			pOP-CNH04407	896
			pOP-CNH04408	381
			pOP-CNH04409	771
			pOP-CNH04410	848
			pOP-CNH04414	856
			pOP-CNH04431	653
			pOP-CNH04436	699
			pOP-CNH04439	573
			pOP-CNH04448	434
			pOP-CNH04452	733
			pOP-CNH04455	712
			pOP-CNH04459	222
			pOP-CNH04460	642
			pOP-CNH04465	782
			pOP-CNH04468	702
			pOP-CNH04470	640
			pOP-CNH04471	792
			pOP-CNH04473	749
			pOP-CNH04478	763
			pOP-CNH04479	781
			pOP-CNH04487	718
			pOP-CNH04488	688
			pOP-CNH04490	651
			pOP-CNH04502	725
			pOP-CNH04504	667
			pOP-CNH04508	712
			pOP-CNH04513	532
			pOP-CNH04517	756
			pOP-CNH04519	740
			pOP-CNH04520	505
			pOP-CNH04521	373
			pOP-CNH04527	766
			pOP-CNH04529	678
			pOP-CNH04534	703
			pOP-CNH04540	818
			pOP-CNH04557	656
			pOP-CNH04565	736
			pOP-CNH04571	643
			pOP-CNH04573	823
			pOP-CNH04579	681
			pOP-CNH04580	832
			pOP-CNH04583	108
			pOP-CNH04592	572
			pOP-CNH04600	198
			pOP-CNH04608	769
			pOP-CNH04620	760
			pOP-CNH04624	843
			pOP-CNH04648	811
			pOP-CNH04649	597
			pOP-CNH04654	854
			pOP-CNH04669	665
			pOP-CNH04670	772

			pOP-CNH04674	711	
			pOP-CNH04679	847	
			pOP-CNH04682	656	
			pOP-CNH04697	742	
			pOP-CNH04702	761	
			pOP-CNH04708	373	
			pOP-CNH04719_EST_C_1_pSK_SK	529	
			pOP-CNH04721_EST_C_1_pSK_SK	224	
			pOP-CNH04725_EST_C_1_pSK_SK	476	
			pOP-CNH04738_EST_C_1_pSK_SK	598	
			pOP-CNH04743_EST_C_1_pSK_SK	556	
			pOP-CNH04744_EST_C_1_pSK_SK	535	
			pOP-CNH04749_EST_C_1_pSK_SK	566	
			pOP-CNH04751_EST_C_1_pSK_SK	486	
			pOP-CNH04761_EST_C_1_pSK_SK	581	
			pOP-CNH04763_EST_C_1_pSK_SK	452	
			pOP-CNH04765_EST_C_1_pSK_SK	576	
			pOP-CNH04770_EST_C_1_pSK_SK	544	
			pOP-CNH04781_EST_C_1_pSK_SK	572	
			pOP-CNH04794_EST_C_1_pSK_SK	561	
			pOP-CNH04804_EST_C_1_pSK_SK	563	
			pOP-CNH04854_EST_C_1_pSK_SK	534	
			pOP-CNH04873_EST_C_1_pSK_SK	757	
			pOP-CNH04878_EST_C_1_pSK_SK	663	
			pOP-CNH04888_EST_C_1_pSK_SK	781	
			pOP-CNH04911_EST_C_1_pSK_SK	660	
			pOP-CNH04920_EST_C_1_pSK_SK	662	
			pOP-CNH04926_EST_C_1_pSK_SK	729	
			pOP-CNH04938_EST_C_1_pSK_SK	133	
			pOP-CNH04941_EST_C_1_pSK_SK	586	
			pOP-CNH04946_EST_C_1_pSK_SK	620	
			pOP-CNH04954_EST_C_1_pSK_SK	596	
			pOP-CNH04957_EST_C_1_pSK_SK	802	
			pOP-CNH04958_EST_C_1_pSK_SK	531	
			pOP-CNH04964_EST_C_1_pSK_SK	705	
			pOP-CNH04966_EST_C_1_pSK_SK	440	
			pOP-CNH04973_EST_C_1_pSK_SK	526	
			pOP-CNH04978_EST_C_1_pSK_SK	651	
			pOP-CNH04985_EST_C_1_pSK_SK	704	
			pOP-CNH04997_EST_C_1_pSK_SK	722	
			pOP-CNH04999_EST_C_1_pSK_SK	688	
			pOP-CNH05014_EST_C_1_pSK_SK	694	
			pOP-CNH05017_EST_C_1_pSK_SK	647	
			pOP-CNH05022_EST_C_1_pSK_SK	166	
			pOP-CNH05030_EST_C_1_pSK_SK	695	
			pOP-CNH05038_EST_C_1_pSK_SK	504	
			pOP-CNH05053_EST_C_1_pSK_SK	751	
			pOP-CNH05057_EST_C_1_pSK_SK	586	
			pOP-CNH05059_EST_C_1_pSK_SK	650	
			pOP-CNH05061_EST_C_1_pSK_SK	493	
			pOP-CNH05066_EST_C_1_pSK_SK	656	
			pOP-CNH05072_EST_C_1_pSK_SK	633	
			pOP-CNH05074_EST_C_1_pSK_SK	614	
			pOP-CNH05082_EST_C_1_pSK_SK	559	
			pOP-CNH05083_EST_C_1_pSK_SK	704	
			pOP-CNH05087_EST_C_1_pSK_SK	611	

			pOP-CNHP00021_EST_C_1_pSK_SK	557
			pOP-CNHP00032_EST_C_1_pSK_SK	513
			pOP-CNHP00037_EST_C_1_pSK_SK	682
			pOP-CNHP00039_EST_C_1_pSK_SK	610
			pOP-CNHP00043_EST_C_1_pSK_SK	631
			pOP-CNHP00051_EST_C_1_pSK_SK	579
			pOP-CNHP00052_EST_C_1_pSK_SK	570
			pOP-CNHP00056_EST_C_1_pSK_SK	596
			pOP-CNHP00057_EST_C_1_pSK_SK	536
			pOP-CNHP00060_EST_C_1_pSK_SK	611
			pOP-CNHP00062_EST_C_1_pSK_SK	584
			pOP-CNHP00065_EST_C_1_pSK_SK	603
			pOP-CNHP00074_EST_C_1_pSK_SK	618
			pOP-CNHP00075_EST_C_1_pSK_SK	640
			pOP-CNHP00077_EST_C_1_pSK_SK	667
			pOP-CNHP00083_EST_C_1_pSK_SK	605
			pOP-CNHP00084_EST_C_1_pSK_SK	543
			pOP-CNHP00085_EST_C_1_pSK_SK	645
			pOP-CNHP00088_EST_C_1_pSK_SK	594
			pOP-CNHP00090_EST_C_1_pSK_SK	641
			pOP-CNHP00091_EST_C_1_pSK_SK	162
			pOP-CNHP00108_EST_C_1_pSK_SK	603
			pOP-CNHP00110_EST_C_1_pSK_SK	604
			pOP-CNHP00111_EST_C_1_pSK_SK	606
			pOP-CNHP00113_EST_C_1_pSK_SK	618
			pOP-CNHP00114_EST_C_1_pSK_SK	599
			pOP-CNHP00115_EST_C_1_pSK_SK	574
			pOP-CNHP00119_EST_C_1_pSK_SK	493
			pOP-CNHP00123_EST_C_1_pSK_SK	577
			pOP-CNHP00124_EST_C_1_pSK_SK	618
			pOP-CNHP00126_EST_C_1_pSK_SK	561
			pOP-CNHP00127_EST_C_1_pSK_SK	610
			pOP-CNHP00131_EST_C_1_pSK_SK	383
			pOP-CNHP00133_EST_C_1_pSK_SK	629
			pOP-CNHP00134_EST_C_1_pSK_SK	673
			pOP-CNHP00139_EST_C_1_pSK_SK	597
			pOP-CNHP00142_EST_C_1_pSK_SK	421
			pOP-CNHP00143_EST_C_1_pSK_SK	511
			pOP-CNHP00144_EST_C_1_pSK_SK	528
			pOP-CNHP00147_EST_C_1_pSK_SK	609
			pOP-CNHP00148_EST_C_1_pSK_SK	409
			pOP-CNHP00149_EST_C_1_pSK_SK	421
			pOP-CNHP00154_EST_C_1_pSK_SK	227
			pOP-CNHP00156_EST_C_1_pSK_SK	493
			pOP-CNHP00164_EST_C_1_pSK_SK	417
			pOP-CNHP00167_EST_C_1_pSK_SK	332
			pOP-CNHP00168_EST_C_1_pSK_SK	252
			pOP-CNHP00169_EST_C_1_pSK_SK	311
			pOP-CNHP00177_EST_C_1_pSK_SK	410
			pOP-CNHP00180_EST_C_1_pSK_SK	526
			pOP-CNHP00189_EST_C_1_pSK_SK	620
			pOP-CNHP00191_EST_C_1_pSK_SK	602
			pOP-CNHP00193_EST_C_1_pSK_SK	628
			pOP-CNHP00195_EST_C_1_pSK_SK	582
			pOP-CNHP00196_EST_C_1_pSK_SK	640
			pOP-CNHP00197_EST_C_1_pSK_SK	667

			pOP-CNHP00201_EST_C_1_pSK_SK	441
			pOP-CNHP00222_EST_C_1_pSK_SK	453
			pOP-CNHP00226_EST_C_1_pSK_SK	626
			pOP-CNHP00240_EST_C_1_pSK_SK	521
			pOP-CNHP00246_EST_C_1_pSK_SK	566
			pOP-CNHP00248_EST_C_1_pSK_SK	548
			pOP-CNHP00250_EST_C_1_pSK_SK	487
			pOP-CNHP00255_EST_C_1_pSK_SK	301
			pOP-CNHP00260_EST_C_1_pSK_SK	558
			pOP-CNHP00261_EST_C_1_pSK_SK	563
			pOP-CNHP00263_EST_C_1_pSK_SK	522
			pOP-CNHP00265_EST_C_1_pSK_SK	424
			pOP-CNHP00269_EST_C_1_pSK_SK	351
			pOP-CNHP00278_EST_C_1_pSK_SK	269
			pOP-CNHP00286_EST_C_1_pSK_SK	553
			pOP-CNHP00289_EST_C_1_pSK_SK	435
			pOP-CNHP00291_EST_C_1_pSK_SK	465
			pOP-CNHP00295_EST_C_1_pSK_SK	435
			pOP-CNHP00301_EST_C_1_pSK_SK	568
			pOP-CNHP00302_EST_C_1_pSK_SK	659
			pOP-CNHP00304_EST_C_1_pSK_SK	503
			pOP-CNHP00306_EST_C_1_pSK_SK	492
			pOP-CNHP00308_EST_C_1_pSK_SK	409
			pOP-CNHP00315_EST_C_1_pSK_SK	644
			pOP-CNHP00317_EST_C_1_pSK_SK	555
			pOP-CNHP00334_EST_C_1_pSK_SK	651
			pOP-CNHP00336_EST_C_1_pSK_SK	661
			pOP-CNHP00343_EST_C_1_pSK_SK	634
			pOP-CNHP00344_EST_C_1_pSK_SK	618
			pOP-CNHP00347_EST_C_1_pSK_SK	221
			pOP-CNHP00348_EST_C_1_pSK_SK	668
			pOP-CNHP00352_EST_C_1_pSK_SK	289
			pOP-CNHP00355_EST_C_1_pSK_SK	241
			pOP-CNHP00358_EST_C_1_pSK_SK	602
			pOP-CNHP00367_EST_C_1_pSK_SK	626
			pOP-CNHP00379_EST_C_1_pSK_SK	517
			pOP-CNHP00391_EST_C_1_pSK_SK	622
			pOP-CNHP00399_EST_C_1_pSK_SK	648
			pOP-CNHP00400_EST_C_1_pSK_SK	663
			pOP-CNHP00418_EST_C_1_pSK_SK	577
			pOP-CNHP00420_EST_C_1_pSK_SK	689
			pOP-CNHP00423_EST_C_1_pSK_SK	675
			pOP-CNHP00426_EST_C_1_pSK_SK	724
			pOP-CNHP00430_EST_C_1_pSK_SK	704
			pOP-CNHP00431_EST_C_1_pSK_SK	722
			pOP-CNHP00436_EST_C_1_pSK_SK	637
			pOP-CNHP00437_EST_C_1_pSK_SK	640
			pOP-CNHP00438_EST_C_1_pSK_SK	741
			pOP-CNHP00440_EST_C_1_pSK_SK	600
			pOP-CNHP00442_EST_C_1_pSK_SK	670
			pOP-CNHP00443_EST_C_1_pSK_SK	682
			pOP-CNHP00444_EST_C_1_pSK_SK	550
			pOP-CNHP00448_EST_C_1_pSK_SK	741
			pOP-CNHP00449_EST_C_1_pSK_SK	676
			pOP-CNHP00453_EST_C_1_pSK_SK	756
			pOP-CNHP00454_EST_C_1_pSK_SK	719

			pOP-CNHP00459_EST_C_1_pSK_SK	721
			pOP-CNHP00471_EST_C_1_pSK_SK	487
			pOP-CNHP00475_EST_C_1_pSK_SK	619
			pOP-CNHP00486_EST_C_1_pSK_SK	611
			pOP-CNHP00489_EST_C_1_pSK_SK	585
			pOP-CNHP00490_EST_C_1_pSK_SK	543
			pOP-CNHP00507_EST_C_1_pSK_SK	429
			pOP-CNHP00511_EST_C_1_pSK_SK	585
			pOP-CNHP00512_EST_C_1_pSK_SK	626
			pOP-CNHP00520_EST_C_1_pSK_SK	453
			pOP-CNHP00524_EST_C_1_pSK_SK	623
			pOP-CNHP00528_EST_C_1_pSK_SK	614
			pOP-CNHP00531_EST_C_1_pSK_SK	591
			pOP-CNHP00538_EST_C_1_pSK_SK	588
			pOP-CNHP00541_EST_C_1_pSK_SK	451
			pOP-CNHP00543_EST_C_1_pSK_SK	593
			pOP-CNI01079_EST_C_1_pSK_SK	367
			pOP-CNI01087_EST_C_1_pSK_SK	478
			pOP-CNI01091_EST_C_1_pSK_SK	117
			pOP-CNI01093_EST_C_1_pSK_SK	331
			pOP-CNI01107_EST_C_1_pSK_SK	283
			pOP-CNI01109_EST_C_1_pSK_SK	402
			pOP-CNI01110_EST_C_1_pSK_SK	254
			pOP-CNI01114_EST_C_1_pSK_SK	338
			pOP-CNI01115_EST_C_1_pSK_SK	623
			pOP-CNI01120_EST_C_1_pSK_SK	419
			pOP-CNI01127_EST_C_1_pSK_SK	472
			pOP-CNI01137_EST_C_1_pSK_SK	362
			pOP-CNI01141_EST_C_1_pSK_SK	365
			pOP-CNI01147_EST_C_1_pSK_SK	390
			pOP-CNI01149_EST_C_1_pSK_SK	334
			pOP-CNI01152_EST_C_1_pSK_SK	331
			pOP-CNI01165_EST_C_1_pSK_SK	548
			pOP-CNI01168_EST_C_1_pSK_SK	531
			pOP-CNI01169_EST_C_1_pSK_SK	450
			pOP-CNI01171_EST_C_1_pSK_SK	352
			pOP-CNI01175_EST_C_1_pSK_SK	403
			pOP-CNI01181_EST_C_1_pSK_SK	420
			pOP-CNI01183_EST_C_1_pSK_SK	332
			pOP-CNI01187_EST_C_1_pSK_SK	682
			pOP-CNI01191_EST_C_1_pSK_SK	302
			pOP-CNI01193_EST_C_1_pSK_SK	331
			pOP-CNI01196_EST_C_1_pSK_SK	288
			pOP-CNI01199_EST_C_1_pSK_SK	591
			pOP-CNI01200_EST_C_1_pSK_SK	215
			pOP-CNI01201_EST_C_1_pSK_SK	147
			pOP-CNI01202_EST_C_1_pSK_SK	346
			pOP-CNI01205_EST_C_1_pSK_SK	254
			pOP-CNI01209_EST_C_1_pSK_SK	369
			pOP-CNI01215_EST_C_1_pSK_SK	383
			pOP-CNI01216_EST_C_1_pSK_SK	176
			pOP-CNI01225_EST_C_1_pSK_SK	189
			pOP-CNI01226_EST_C_1_pSK_SK	362
			pOP-CNI01227_EST_C_1_pSK_SK	602
			pOP-CNI01235_EST_C_1_pSK_SK	319
			pOP-CNI01239_EST_C_1_pSK_SK	391

			pOP-CNI01241_EST_C_1_pSK_SK	346
			pOP-CNI01243_EST_C_1_pSK_SK	329
			pOP-CNI01245_EST_C_1_pSK_SK	597
			pOP-CNI01246_EST_C_1_pSK_SK	429
			pOP-CNI01248_EST_C_1_pSK_SK	531
			pOP-CNI01251_EST_C_1_pSK_SK	481
			pOP-CNI01254_EST_C_1_pSK_SK	640
			pOP-CNI01255_EST_C_1_pSK_SK	494
			pOP-CNI01259_EST_C_1_pSK_SK	419
			pOP-CNI01260_EST_C_1_pSK_SK	582
			pOP-CNI01263_EST_C_1_pSK_SK	478
			pOP-CNI01264_EST_C_1_pSK_SK	256
			pOP-CNI01267_EST_C_1_pSK_SK	175
			pOP-CNI01274_EST_C_1_pSK_SK	138
			pOP-CNI01275_EST_C_1_pSK_SK	577
			pOP-CNI01276_EST_C_1_pSK_SK	374
			pOP-CNI01277_EST_C_1_pSK_SK	353
			pOP-CNI01278_EST_C_1_pSK_SK	273
			pOP-CNI01283_EST_C_1_pSK_SK	265
			pOP-CNI01284_EST_C_1_pSK_SK	333
			pOP-CNI01288_EST_C_1_pSK_SK	304
			pOP-CNI01291_EST_C_1_pSK_SK	578
			pOP-CNI01293_EST_C_1_pSK_SK	393
			pOP-CNI01294_EST_C_1_pSK_SK	165
			pOP-CNI01297_EST_C_1_pSK_SK	102
			pOP-CNI01299_EST_C_1_pSK_SK	193
			pOP-CNI01300_EST_C_1_pSK_SK	592
			pOP-CNI01303_EST_C_1_pSK_SK	247
			pOP-CNI01304_EST_C_1_pSK_SK	654
			pOP-CNI01305_EST_C_1_pSK_SK	633
			pOP-CNI01306_EST_C_1_pSK_SK	427
			pOP-CNI01307_EST_C_1_pSK_SK	159
			pOP-CNI01312_EST_C_1_pSK_SK	340
			pOP-CNI01317_EST_C_1_pSK_SK	264
			pOP-CNI01318_EST_C_1_pSK_SK	415
			pOP-CNI01320_EST_C_1_pSK_SK	407
			pOP-CNI01321_EST_C_1_pSK_SK	274
			pOP-CNI01323_EST_C_1_pSK_SK	328
			pOP-CNI01333_EST_C_1_pSK_SK	380
			pOP-CNI01336_EST_C_1_pSK_SK	370
			pOP-CNI01337_EST_C_1_pSK_SK	442
			pOP-CNI01345_EST_C_1_pSK_SK	532
			pOP-CNI01348_EST_C_1_pSK_SK	248
			pOP-CNI01350_EST_C_1_pSK_SK	410
			pOP-CNI01351_EST_C_1_pSK_SK	278
			pOP-CNI01353_EST_C_1_pSK_SK	231
			pOP-CNI01357_EST_C_1_pSK_SK	302
			pOP-CNI01358_EST_C_1_pSK_SK	470
			pOP-CNI01361_EST_C_1_pSK_SK	419
			pOP-CNI01363_EST_C_1_pSK_SK	485
			pOP-CNI01374_EST_C_1_pSK_SK	374
			pOP-CNI01375_EST_C_1_pSK_SK	361
			pOP-CNI01380_EST_C_1_pSK_SK	502
			pOP-CNI01381_EST_C_1_pSK_SK	141
			pOP-CNI01382_EST_C_1_pSK_SK	368
			pOP-CNI01383_EST_C_1_pSK_SK	502

			pOP-CNI01388_EST_C_1_pSK_SK	543
			pOP-CNI01389_EST_C_1_pSK_SK	568
			pOP-CNI01393_EST_C_1_pSK_SK	248
			pOP-CNI01396_EST_C_1_pSK_SK	680
			pOP-CNI01402_EST_C_1_pSK_SK	720
			pOP-CNI01404_EST_C_1_pSK_SK	366
			pOP-CNI01407_EST_C_1_pSK_SK	162
			pOP-CNI01409_EST_C_1_pSK_SK	308
			pOP-CNI01410_EST_C_1_pSK_SK	219
			pOP-CNI01411_EST_C_1_pSK_SK	316
			pOP-CNI01412_EST_C_1_pSK_SK	517
			pOP-CNI01416_EST_C_1_pSK_SK	609
			pOP-CNI01417_EST_C_1_pSK_SK	395
			pOP-CNI01419_EST_C_1_pSK_SK	298
			pOP-CNI01420_EST_C_1_pSK_SK	590
			pOP-CNI01425_EST_C_1_pSK_SK	200
			pOP-CNI01427_EST_C_1_pSK_SK	380
			pOP-CNI01432_EST_C_1_pSK_SK	411
			pOP-CNI01439_EST_C_1_pSK_SK	626
			pOP-CNI01442_EST_C_1_pSK_SK	139
			pOP-CNI01444_EST_C_1_pSK_SK	180
			pOP-CNI01448_EST_C_1_pSK_SK	601
			pOP-CNI01450_EST_C_1_pSK_SK	305
			pOP-CNI01452_EST_C_1_pSK_SK	292
			pOP-CNI01453_EST_C_1_pSK_SK	418
			pOP-CNI01454_EST_C_1_pSK_SK	316
			pOP-CNI01457_EST_C_1_pSK_SK	427
			pOP-CNI01458_EST_C_1_pSK_SK	443
			pOP-CNI01463_EST_C_1_pSK_SK	404
			pOP-CNI01464_EST_C_1_pSK_SK	483
			pOP-CNI01466_EST_C_1_pSK_SK	383
			pOP-CNI01471_EST_C_1_pSK_SK	306
			pOP-CNI01472_EST_C_1_pSK_SK	245
			pOP-CNI01476_EST_C_1_pSK_SK	246
			pOP-CNI01484_EST_C_1_pSK_SK	448
			pOP-CNI01493_EST_C_1_pSK_SK	300
			pOP-CNI01494_EST_C_1_pSK_SK	476
			pOP-CNI01499_EST_C_1_pSK_SK	610
			pOP-CNI01505_EST_C_1_pSK_SK	369
			pOP-CNI01508_EST_C_1_pSK_SK	497
			pOP-CNI01512_EST_C_1_pSK_SK	528
			pOP-CNI01516_EST_C_1_pSK_SK	475
			pOP-CNI01518_EST_C_1_pSK_SK	279
			pOP-CNI01521_EST_C_1_pSK_SK	317
			pOP-CNI01523_EST_C_1_pSK_SK	354
			pOP-CNI01524_EST_C_1_pSK_SK	381
			pOP-CNI01527_EST_C_1_pSK_SK	251
			pOP-CNI01528_EST_C_1_pSK_SK	123
			pOP-CNI01534_EST_C_1_pSK_SK	368
			pOP-CNI01541_EST_C_1_pSK_SK	250
			pOP-CNI01542_EST_C_1_pSK_SK	182
			pOP-CNI01546_EST_C_1_pSK_SK	198
			pOP-CNI01548_EST_C_1_pSK_SK	314
			pOP-CNI01550_EST_C_1_pSK_SK	332
			pOP-CNI01557_EST_C_1_pSK_SK	262
			pOP-CNI01560_EST_C_1_pSK_SK	360

			pOP-CNI01562_EST_C_1_pSK_SK	114
			pOP-CNI01569_EST_C_1_pSK_SK	637
			pOP-CNI01574_EST_C_1_pSK_SK	339
			pOP-CNI01575_EST_C_1_pSK_SK	363
			pOP-CNI01576_EST_C_1_pSK_SK	372
			pOP-CNI01577_EST_C_1_pSK_SK	345
			pOP-CNI01578_EST_C_1_pSK_SK	238
			pOP-CNI01580_EST_C_1_pSK_SK	294
			pOP-CNI01581_EST_C_1_pSK_SK	343
			pOP-CNI01583_EST_C_1_pSK_SK	285
			pOP-CNI01585_EST_C_1_pSK_SK	252
			pOP-CNI01590_EST_C_1_pSK_SK	382
			pOP-CNI01591_EST_C_1_pSK_SK	296
			pOP-CNI01592_EST_C_1_pSK_SK	526
			pOP-CNI01596_EST_C_1_pSK_SK	623
			pOP-CNI01610_EST_C_1_pSK_SK	391
			pOP-CNI01618_EST_C_1_pSK_SK	111
			pOP-CNI01619_EST_C_1_pSK_SK	338
			pOP-CNI01621_EST_C_1_pSK_SK	311
			pOP-CNI01632_EST_C_1_pSK_SK	397
			pOP-CNI01649_EST_C_1_pSK_SK	276
			pOP-CNI01656_EST_C_1_pSK_SK	363
			pOP-CNI01659_EST_C_1_pSK_SK	352
			pOP-CNI01660_EST_C_1_pSK_SK	600
			pOP-CNI01661_EST_C_1_pSK_SK	519
			pOP-CNI01663_EST_C_1_pSK_SK	242
			pOP-CNI01665_EST_C_1_pSK_SK	272
			pOP-CNI01671_EST_C_1_pSK_SK	406
			pOP-CNI01679_EST_C_1_pSK_SK	289
			pOP-CNI01682_EST_C_1_pSK_SK	452
			pOP-CNI01683_EST_C_1_pSK_SK	172
			pOP-CNI01684_EST_C_1_pSK_SK	692
			pOP-CNI01687_EST_C_1_pSK_SK	480
			pOP-CNI01695_EST_C_1_pSK_SK	265
			pOP-CNI01698_EST_C_1_pSK_SK	216
			pOP-CNI01706_EST_C_1_pSK_SK	262
			pOP-CNI01708_EST_C_1_pSK_SK	419
			pOP-CNI01709_EST_C_1_pSK_SK	285
			pOP-CNI01711_EST_C_1_pSK_SK	120
			pOP-CNI01722_EST_C_1_pSK_SK	453
			pOP-CNI01724_EST_C_1_pSK_SK	488
			pOP-CNI01730_EST_C_1_pSK_SK	305
			pOP-CNI01731_EST_C_1_pSK_SK	466
			pOP-CNI01740_EST_C_1_pSK_SK	429
			pOP-CNI01744_EST_C_1_pSK_SK	292
			pOP-CNI01746_EST_C_1_pSK_SK	464
			pOP-CNI01749_EST_C_1_pSK_SK	331
			pOP-CNI01752_EST_C_1_pSK_SK	549
			pOP-CNI01755_EST_C_1_pSK_SK	424
			pOP-CNI01764_EST_C_1_pSK_SK	351
			pOP-CNI01766_EST_C_1_pSK_SK	502
			pOP-CNI01769_EST_C_1_pSK_SK	274
			pOP-CNI01772_EST_C_1_pSK_SK	344
			pOP-CNI01773_EST_C_1_pSK_SK	569
			pOP-CNI01780_EST_C_1_pSK_SK	438
			pOP-CNI01782_EST_C_1_pSK_SK	230

			pOP-CNI01791_EST_C_1_pSK_SK	170	
			pOP-CNI01794_EST_C_1_pSK_SK	409	
			pOP-CNI01796_EST_C_1_pSK_SK	262	
			pOP-CNI01801_EST_C_1_pSK_SK	481	
			pOP-CNI01808_EST_C_1_pSK_SK	312	
			pOP-CNI01810_EST_C_1_pSK_SK	502	
			pOP-CNI01823_EST_C_1_pSK_SK	125	
			pOP-CNI01824_EST_C_1_pSK_SK	520	
			pOP-CNI01825_EST_C_1_pSK_SK	349	
			pOP-CNI01829_EST_C_1_pSK_SK	482	
			pOP-CNI01832_EST_C_1_pSK_SK	394	
			pOP-CNI01834_EST_C_1_pSK_SK	538	
			pOP-CNI01835_EST_C_1_pSK_SK	277	
			pOP-CNI01837_EST_C_1_pSK_SK	203	
			pOP-CNI01842_EST_C_1_pSK_SK	163	
			pOP-CNI01847_EST_C_1_pSK_SK	429	
			pOP-CNI01853_EST_C_1_pSK_SK	488	
			pOP-CNI01856_EST_C_1_pSK_SK	507	
			pOP-CNI01857_EST_C_1_pSK_SK	486	
			pOP-CNI01859_EST_C_1_pSK_SK	409	
			pOP-CNI01865_EST_C_1_pSK_SK	394	
			pOP-CNI01867_EST_C_1_pSK_SK	571	
			pOP-CNI01868_EST_C_1_pSK_SK	434	
			pOP-CNI01869_EST_C_1_pSK_SK	224	
			pOP-CNI01870_EST_C_1_pSK_SK	244	
			pOP-CNI01875_EST_C_1_pSK_SK	194	
			pOP-CNI01876_EST_C_1_pSK_SK	602	
			pOP-CNI01879_EST_C_1_pSK_SK	512	
			pOP-CNI01880_EST_C_1_pSK_SK	380	
			pOP-CNI01883_EST_C_1_pSK_SK	498	
			pOP-CNI01889_EST_C_1_pSK_SK	428	
			pOP-CNI01891_EST_C_1_pSK_SK	148	
			pOP-CNI01899_EST_C_1_pSK_SK	619	
			pOP-CNI01903_EST_C_1_pSK_SK	285	
			pOP-CNI01910_EST_C_1_pSK_SK	363	
			pOP-CNI01913_EST_C_1_pSK_SK	552	
			pOP-CNI01918_EST_C_1_pSK_SK	503	
			pOP-CNI01920_EST_C_1_pSK_SK	223	
			pOP-CNI01923_EST_C_1_pSK_SK	279	
			pOP-CNI01926_EST_C_1_pSK_SK	575	
			pOP-CNI01932_EST_C_1_pSK_SK	180	
			pOP-CNI01933_EST_C_1_pSK_SK	311	
			pOP-CNI01934_EST_C_1_pSK_SK	585	
			pOP-CNI01935_EST_C_1_pSK_SK	183	
			pOP-CNI01938_EST_C_1_pSK_SK	192	
			pOP-CNI01940_EST_C_1_pSK_SK	570	
			pOP-CNI01942_EST_C_1_pSK_SK	193	
			pOP-CNI01943_EST_C_1_pSK_SK	467	
			pOP-CNI01950_EST_C_1_pSK_SK	285	
			pOP-CNI01960_EST_C_1_pSK_SK	375	
			pOP-CNI01971_EST_C_1_pSK_SK	432	
			pOP-CNI01973_EST_C_1_pSK_SK	335	
			pOP-CNI01975_EST_C_1_pSK_SK	259	
			pOP-CNI01976_EST_C_1_pSK_SK	285	
			pOP-CNI01981_EST_C_1_pSK_SK	294	
			pOP-CNI01982_EST_C_1_pSK_SK	313	

			pOP-CNI01983_EST_C_1_pSK_SK	285
			pOP-CNI01984_EST_C_1_pSK_SK	374
			pOP-CNI01997_EST_C_1_pSK_SK	439
			pOP-CNI01998_EST_C_1_pSK_SK	256
			pOP-CNI02010_EST_C_1_pSK_SK	355
			pOP-CNI02012_EST_C_1_pSK_SK	246
			pOP-CNI02015_EST_C_1_pSK_SK	439
			pOP-CNI02017_EST_C_1_pSK_SK	209
			pOP-CNI02023_EST_C_1_pSK_SK	355
			pOP-CNI02025_EST_C_1_pSK_SK	125
			pOP-CNI02026_EST_C_1_pSK_SK	601
			pOP-CNI02032_EST_C_1_pSK_SK	489
			pOP-CNI02044_EST_C_1_pSK_SK	560
			pOP-CNI02045_EST_C_1_pSK_SK	525
			pOP-CNI02048_EST_C_1_pSK_SK	571
			pOP-CNI02050_EST_C_1_pSK_SK	117
			pOP-CNI02055_EST_C_1_pSK_SK	285
			pOP-CNI02057_EST_C_1_pSK_SK	339
			pOP-CNI02060_EST_C_1_pSK_SK	337
			pOP-CNI02061_EST_C_1_pSK_SK	334
			pOP-CNI02065_EST_C_1_pSK_SK	531
			pOP-CNI02068_EST_C_1_pSK_SK	331
			pOP-CNI02077_EST_C_1_pSK_SK	330
			pOP-CNI02079_EST_C_1_pSK_SK	363
			pOP-CNI02083_EST_C_1_pSK_SK	635
			pOP-CNI02095_EST_C_1_pSK_SK	657
			pOP-CNI02096_EST_C_1_pSK_SK	583
			pOP-CNI02097_EST_C_1_pSK_SK	255
			pOP-CNI02101_EST_C_1_pSK_SK	465
			pOP-CNI02103_EST_C_1_pSK_SK	419
			pOP-CNI02104_EST_C_1_pSK_SK	131
			pOP-CNI02109_EST_C_1_pSK_SK	128
			pOP-CNI02111_EST_C_1_pSK_SK	420
			pOP-CNI02115_EST_C_1_pSK_SK	191
			pOP-CNI02120_EST_C_1_pSK_SK	293
			pOP-CNI02123_EST_C_1_pSK_SK	418
			pOP-CNI02126_EST_C_1_pSK_SK	388
			pOP-CNI02127_EST_C_1_pSK_SK	327
			pOP-CNI02128_EST_C_1_pSK_SK	368
			pOP-CNI02129_EST_C_1_pSK_SK	256
			pOP-CNI02130_EST_C_1_pSK_SK	251
			pOP-CNI02132_EST_C_1_pSK_SK	472
			pOP-CNI02135_EST_C_1_pSK_SK	528
			pOP-CNI02136_EST_C_1_pSK_SK	534
			pOP-CNI02137_EST_C_1_pSK_SK	136
			pOP-CNI02141_EST_C_1_pSK_SK	355
			pOP-CNI02147_EST_C_1_pSK_SK	137
			pOP-CNI02149_EST_C_1_pSK_SK	227
			pOP-CNI02151_EST_C_1_pSK_SK	474
			pOP-CNI02155_EST_C_1_pSK_SK	589
			pOP-CNI02159_EST_C_1_pSK_SK	307
			pOP-CNI02160_EST_C_1_pSK_SK	395
			pOP-CNI02162_EST_C_1_pSK_SK	385
			pOP-CNI02163_EST_C_1_pSK_SK	273
			pOP-CNI02168_EST_C_1_pSK_SK	293
			pOP-CNI02172_EST_C_1_pSK_SK	436

			pOP-CNI02173_EST_C_1_pSK_SK	366
			pOP-CNI02183_EST_C_1_pSK_SK	126
			pOP-CNI02188_EST_C_1_pSK_SK	684
			pOP-CNI02189_EST_C_1_pSK_SK	344
			pOP-CNI02191_EST_C_1_pSK_SK	346
			pOP-CNI02194_EST_C_1_pSK_SK	302
			pOP-CNI02196_EST_C_1_pSK_SK	583
			pOP-CNI02200_EST_C_1_pSK_SK	747
			pOP-CNI02202_EST_C_1_pSK_SK	363
			pOP-CNI02203_EST_C_1_pSK_SK	276
			pOP-CNI02204_EST_C_1_pSK_SK	521
			pOP-CNI02208_EST_C_1_pSK_SK	732
			pOP-CNI02211_EST_C_1_pSK_SK	281
			pOP-CNI02215_EST_C_1_pSK_SK	186
			pOP-CNI02217_EST_C_1_pSK_SK	234
			pOP-CNI02218_EST_C_1_pSK_SK	167
			pOP-CNI02222_EST_C_1_pSK_SK	380
			pOP-CNI02229_EST_C_1_pSK_SK	138
			pOP-CNI02230_EST_C_1_pSK_SK	563
			pOP-CNI02245_EST_C_1_pSK_SK	342
			pOP-CNI02248_EST_C_1_pSK_SK	198
			pOP-CNI02249_EST_C_1_pSK_SK	325
			pOP-CNIP00002_EST_C_1_pSK_SK	405
			pOP-CNIP00005_EST_C_1_pSK_SK	322
			pOP-CNIP00018_EST_C_1_pSK_SK	517
			pOP-CNIP00019_EST_C_1_pSK_SK	685
			pOP-CNIP00022_EST_C_1_pSK_SK	406
			pOP-CNIP00024_EST_C_1_pSK_SK	678
			pOP-CNIP00025_EST_C_1_pSK_SK	379
			pOP-CNIP00026_EST_C_1_pSK_SK	371
			pOP-CNIP00033_EST_C_1_pSK_SK	251
			pOP-CNIP00037_EST_C_1_pSK_SK	393
			pOP-CNIP00043_EST_C_1_pSK_SK	119
			pOP-CNIP00045_EST_C_1_pSK_SK	329
			pOP-CNIP00049_EST_C_1_pSK_SK	412
			pOP-CNIP00050_EST_C_1_pSK_SK	586
			pOP-CNIP00052_EST_C_1_pSK_SK	246
			pOP-CNIP00054_EST_C_1_pSK_SK	628
			pOP-CNIP00059_EST_C_1_pSK_SK	603
			pOP-CNIP00060_EST_C_1_pSK_SK	643
			pOP-CNIP00062_EST_C_1_pSK_SK	331
			pOP-CNIP00064_EST_C_1_pSK_SK	617
			pOP-CNIP00066_EST_C_1_pSK_SK	464
			pOP-CNIP00075_EST_C_1_pSK_SK	259
			pOP-CNIP00079_EST_C_1_pSK_SK	576
			pOP-CNIP00081_EST_C_1_pSK_SK	533
			pOP-CNIP00085_EST_C_1_pSK_SK	194
			pOP-CNIP00086_EST_C_1_pSK_SK	547
			pOP-CNIP00089_EST_C_1_pSK_SK	304
			pOP-CNIP00090_EST_C_1_pSK_SK	304
			pOP-CNIP00093_EST_C_1_pSK_SK	454
			pOP-CNIP00101_EST_C_1_pSK_SK	237
			pOP-CNIP00102_EST_C_1_pSK_SK	459
			pOP-CNIP00103_EST_C_1_pSK_SK	282
			pOP-CNIP00105_EST_C_1_pSK_SK	422
			pOP-CNIP00106_EST_C_1_pSK_SK	414

			pOP-CNIP00115_EST_C_1_pSK_SK	212
			pOP-CNIP00120_EST_C_1_pSK_SK	423
			pOP-CNIP00126_EST_C_1_pSK_SK	392
			pOP-CNIP00130_EST_C_1_pSK_SK	249
			pOP-CNIP00137_EST_C_1_pSK_SK	302
			pOP-CNIP00143_EST_C_1_pSK_SK	281
			pOP-CNIP00144_EST_C_1_pSK_SK	365
			pOP-CNIP00149_EST_C_1_pSK_SK	291
			pOP-CNIP00153_EST_C_1_pSK_SK	425
			pOP-CNIP00157_EST_C_1_pSK_SK	266
			pOP-CNIP00167_EST_C_1_pSK_SK	243
			pOP-CNIP00174_EST_C_1_pSK_SK	371
			pOP-CNIP00183_EST_C_1_pSK_SK	438
			pOP-CNIP00184_EST_C_1_pSK_SK	131
			pOP-CNIP00188_EST_C_1_pSK_SK	438
			pOP-CNIP00192_EST_C_1_pSK_SK	309
			pOP-CNIP00196_EST_C_1_pSK_SK	328
			pOP-CNIP00200_EST_C_1_pSK_SK	502
			pOP-CNIP00206_EST_C_1_pSK_SK	144
			pOP-CNIP00208_EST_C_1_pSK_SK	324
			pOP-CNIP00217_EST_C_1_pSK_SK	521
			pOP-CNIP00219_EST_C_1_pSK_SK	488
			pOP-CNIP00222_EST_C_1_pSK_SK	215
			pOP-CNIP00224_EST_C_1_pSK_SK	304
			pOP-CNIP00226_EST_C_1_pSK_SK	516
			pOP-CNIP00227_EST_C_1_pSK_SK	403
			pOP-CNIP00231_EST_C_1_pSK_SK	271
			pOP-CNIP00234_EST_C_1_pSK_SK	109
			pOP-CNIP00242_EST_C_1_pSK_SK	372
			pOP-CNIP00243_EST_C_1_pSK_SK	505
			pOP-CNIP00248_EST_C_1_pSK_SK	276
			pOP-CNIP00249_EST_C_1_pSK_SK	264
			pOP-CNIP00251_EST_C_1_pSK_SK	410
			pOP-CNIP00253_EST_C_1_pSK_SK	362
			pOP-CNIP00255_EST_C_1_pSK_SK	156
			pOP-CNIP00256_EST_C_1_pSK_SK	356
			pOP-CNIP00261_EST_C_1_pSK_SK	452
			pOP-CNIP00262_EST_C_1_pSK_SK	197
			pOP-CNIP00265_EST_C_1_pSK_SK	203
			pOP-CNIP00266_EST_C_1_pSK_SK	308
			pOP-CNIP00267_EST_C_1_pSK_SK	355
			pOP-CNIP00269_EST_C_1_pSK_SK	192
			pOP-CNIP00270_EST_C_1_pSK_SK	253
			pOP-CNIP00274_EST_C_1_pSK_SK	268
			pOP-CNIP00276_EST_C_1_pSK_SK	407
			pOP-CNIP00285_EST_C_1_pSK_SK	277
			pOP-CNIP00286_EST_C_1_pSK_SK	323
			pOP-CNIP00287_EST_C_1_pSK_SK	146
			pOP-CNIP00291_EST_C_1_pSK_SK	192
			pOP-CNIP00293_EST_C_1_pSK_SK	217
			pOP-CNIP00295_EST_C_1_pSK_SK	491
			pOP-CNIP00297_EST_C_1_pSK_SK	386
			pOP-CNIP00298_EST_C_1_pSK_SK	541
			pOP-CNIP00299_EST_C_1_pSK_SK	380
			pOP-CNIP00301_EST_C_1_pSK_SK	192
			pOP-CNIP00307_EST_C_1_pSK_SK	234

			pOP-CNIP00309_EST_C_1_pSK_SK	478
			pOP-CNIP00311_EST_C_1_pSK_SK	345
			pOP-CNIP00314_EST_C_1_pSK_SK	298
			pOP-CNIP00320_EST_C_1_pSK_SK	263
			pOP-CNIP00322_EST_C_1_pSK_SK	164
			pOP-CNIP00327_EST_C_1_pSK_SK	497
			pOP-CNIP00328_EST_C_1_pSK_SK	528
			pOP-CNIP00331_EST_C_1_pSK_SK	430
			pOP-CNIP00334_EST_C_1_pSK_SK	239
			pOP-CNIP00335_EST_C_1_pSK_SK	329
			pOP-CNIP00339_EST_C_1_pSK_SK	337
			pOP-CNIP00347_EST_C_1_pSK_SK	398
			pOP-CNIP00350_EST_C_1_pSK_SK	457
			pOP-CNIP00352_EST_C_1_pSK_SK	295
			pOP-CNIP00353_EST_C_1_pSK_SK	297
			pOP-CNIP00354_EST_C_1_pSK_SK	126
			pOP-CNIP00356_EST_C_1_pSK_SK	111
			pOP-CNIP00363_EST_C_1_pSK_SK	263
			pOP-CNIP00367_EST_C_1_pSK_SK	224
			pOP-CNIP00368_EST_C_1_pSK_SK	423
			pOP-CNIP00369_EST_C_1_pSK_SK	250
			pOP-CNIP00373_EST_C_1_pSK_SK	512
			pOP-CNIP00375_EST_C_1_pSK_SK	479
			pOP-CNIP00379_EST_C_1_pSK_SK	295
			pOP-CNIP00380_EST_C_1_pSK_SK	535
			pOP-CNIP00382_EST_C_1_pSK_SK	232
			pOP-CNIP00385_EST_C_1_pSK_SK	189
			pOP-CNIP00386_EST_C_1_pSK_SK	450
			pOP-CNIP00387_EST_C_1_pSK_SK	282
			pOP-CNIP00392_EST_C_1_pSK_SK	201
			pOP-CNIP00394_EST_C_1_pSK_SK	153
			pOP-CNIP00395_EST_C_1_pSK_SK	350
			pOP-CNIP00400_EST_C_1_pSK_SK	516
			pOP-CNIP00403_EST_C_1_pSK_SK	513
			pOP-CNIP00404_EST_C_1_pSK_SK	368
			pOP-CNIP00406_EST_C_1_pSK_SK	256
			pOP-CNIP00408_EST_C_1_pSK_SK	175
			pOP-CNIP00414_EST_C_1_pSK_SK	215
			pOP-CNIP00416_EST_C_1_pSK_SK	542
			pOP-CNIP00423_EST_C_1_pSK_SK	602
			pOP-CNIP00426_EST_C_1_pSK_SK	246
			pOP-CNIP00429_EST_C_1_pSK_SK	348
			pOP-CNIP00433_EST_C_1_pSK_SK	374
			pOP-CNIP00441_EST_C_1_pSK_SK	378
			pOP-CNIP00442_EST_C_1_pSK_SK	465
			pOP-CNIP00446_EST_C_1_pSK_SK	265
			pOP-CNIP00447_EST_C_1_pSK_SK	447
			pOP-CNIP00448_EST_C_1_pSK_SK	394
			pOP-CNIP00450_EST_C_1_pSK_SK	539
			pOP-CNIP00452_EST_C_1_pSK_SK	571
			pOP-CNIP00453_EST_C_1_pSK_SK	467
			pOP-CNIP00456_EST_C_1_pSK_SK	197
			pOP-CNIP00458_EST_C_1_pSK_SK	229
			pOP-CNIP00464_EST_C_1_pSK_SK	451
			pOP-CNIP00465_EST_C_1_pSK_SK	452
			pOP-CNIP00467_EST_C_1_pSK_SK	274

			pOP-CNIP00474_EST_C_1_pSK_SK	504
			pOP-CNIP00479_EST_C_1_pSK_SK	365
			pOP-CNIP00489_EST_C_1_pSK_SK	550
			pOP-CNIP00496_EST_C_1_pSK_SK	391
			pOP-CNIP00497_EST_C_1_pSK_SK	431
			pOP-CNIP00498_EST_C_1_pSK_SK	615
			pOP-CNIP00500_EST_C_1_pSK_SK	303
			pOP-CNIP00501_EST_C_1_pSK_SK	277
			pOP-CNIP00503_EST_C_1_pSK_SK	431
			pOP-CNIP00504_EST_C_1_pSK_SK	138
			pOP-CNIP00505_EST_C_1_pSK_SK	497
			pOP-CNIP00506_EST_C_1_pSK_SK	554
			pOP-CNIP00508_EST_C_1_pSK_SK	409
			pOP-CNIP00512_EST_C_1_pSK_SK	614
			pOP-CNIP00514_EST_C_1_pSK_SK	124
			pOP-CNIP00518_EST_C_1_pSK_SK	611
			pOP-CNIP00532_EST_C_1_pSK_SK	418
			pOP-CNIP00537_EST_C_1_pSK_SK	434
			pOP-CNIP00559_EST_C_1_pSK_SK	255
			pOP-CNIP00562_EST_C_1_pSK_SK	557
			pOP-CNIP00565_EST_C_1_pSK_SK	451
			pOP-CNIP00573_EST_C_1_pSK_SK	571
			pOP-CNIP00574_EST_C_1_pSK_SK	534
			pOP-CNIP00576_EST_C_1_pSK_SK	651
			pOP-CNIP00578_EST_C_1_pSK_SK	431
			pOP-CNIP00584_EST_C_1_pSK_SK	616
			pOP-CNIP00586_EST_C_1_pSK_SK	257
			pOP-CNIP00598_EST_C_1_pSK_SK	383
			pOP-CNIP00600_EST_C_1_pSK_SK	497
			pOP-CNIP00602_EST_C_1_pSK_SK	592
			pOP-CNIP00603_EST_C_1_pSK_SK	546
			pOP-CNIP00606_EST_C_1_pSK_SK	191
			pOP-CNIP00611_EST_C_1_pSK_SK	495
			pOP-CNIP00618_EST_C_1_pSK_SK	149
			pOP-CNIP00619_EST_C_1_pSK_SK	228
			pOP-CNIP00622_EST_C_1_pSK_SK	301
			pOP-CNIP00623_EST_C_1_pSK_SK	257
			pOP-CNIP00626_EST_C_1_pSK_SK	255
			pOP-CNIP00629_EST_C_1_pSK_SK	466
			pOP-CNIP00641_EST_C_1_pSK_SK	447
			pOP-CNIP00643_EST_C_1_pSK_SK	432
			pOP-CNIP00644_EST_C_1_pSK_SK	322
			pOP-CNIP00645_EST_C_1_pSK_SK	138
			pOP-CNIP00646_EST_C_1_pSK_SK	164
			pOP-CNIP00648_EST_C_1_pSK_SK	441
			pOP-CNIP00649_EST_C_1_pSK_SK	302
			pOP-CNIP00651_EST_C_1_pSK_SK	417
			pOP-CNIP00652_EST_C_1_pSK_SK	395
			pOP-CNIP00654_EST_C_1_pSK_SK	285
			pOP-CNIP00655_EST_C_1_pSK_SK	523
			pOP-CNIP00659_EST_C_1_pSK_SK	640
			pOP-CNIP00660_EST_C_1_pSK_SK	225
			pOP-CNIP00669_EST_C_1_pSK_SK	352
			pOP-CNIP00670_EST_C_1_pSK_SK	379
			pOP-CNIP00676_EST_C_1_pSK_SK	357
			pOP-CNIP00677_EST_C_1_pSK_SK	288

			pOP-CNIP00685_EST_C_1_pSK_SK	707
			pOP-CNIP00690_EST_C_1_pSK_SK	574
			pOP-CNIP00694_EST_C_1_pSK_SK	582
			pOP-CNIP00699_EST_C_1_pSK_SK	109
			pOP-CNIP00704_EST_C_1_pSK_SK	567
			pOP-CNIP00712_EST_C_1_pSK_SK	237
			pOP-CNIP00723_EST_C_1_pSK_SK	676
			pOP-CNIP00727_EST_C_1_pSK_SK	595
			pOP-CNIP00730_EST_C_1_pSK_SK	247
			pOP-CNIP00733_EST_C_1_pSK_SK	187
			pOP-CNIP00736_EST_C_1_pSK_SK	342
			pOP-CNIP00738_EST_C_1_pSK_SK	200
			pOP-CNIP00741_EST_C_1_pSK_SK	264
			pOP-CNIP00744_EST_C_1_pSK_SK	371
			pOP-CNIP00753_EST_C_1_pSK_SK	743
			pOP-CNIP00761_EST_C_1_pSK_SK	673
			pOP-CNIP00767_EST_C_1_pSK_SK	671
			pOP-CNIP00768_EST_C_1_pSK_SK	654
			pOP-CNIP00769_EST_C_1_pSK_SK	410
			pOP-CNIP00774_EST_C_1_pSK_SK	427
			pOP-CNIP00778_EST_C_1_pSK_SK	322
			pOP-CNIP00781_EST_C_1_pSK_SK	237
			pOP-CNIP00786_EST_C_1_pSK_SK	495
			pOP-CNIP00788_EST_C_1_pSK_SK	538
			pOP-CNIP00794_EST_C_1_pSK_SK	636
			pOP-CNIP00798_EST_C_1_pSK_SK	208
			pOP-CNIP00804_EST_C_1_pSK_SK	338
			pOP-CNIP00805_EST_C_1_pSK_SK	383
			pOP-CNIP00807_EST_C_1_pSK_SK	704
			pOP-CNIP00808_EST_C_1_pSK_SK	531
			pOP-CNIP00810_EST_C_1_pSK_SK	301
			pOP-CNIP00818_EST_C_1_pSK_SK	292
			pOP-CNIP00821_EST_C_1_pSK_SK	222
			pOP-CNIP00822_EST_C_1_pSK_SK	239
			pOP-CNIP00832_EST_C_1_pSK_SK	254
			pOP-CNIP00833_EST_C_1_pSK_SK	248
			pOP-CNIP00834_EST_C_1_pSK_SK	251
			pOP-CNIP00840_EST_C_1_pSK_SK	227
			pOP-CNIP00841_EST_C_1_pSK_SK	266
			pOP-CNIP00845_EST_C_1_pSK_SK	452
			pOP-CNIP00849_EST_C_1_pSK_SK	332
			pOP-CNIP00851_EST_C_1_pSK_SK	618
			pOP-CNIP00852_EST_C_1_pSK_SK	671
			pOP-CNIP00862_EST_C_1_pSK_SK	255
			pOP-CNIP00864_EST_C_1_pSK_SK	180
			pOP-CNIP00866_EST_C_1_pSK_SK	369
			pOP-CNIP00867_EST_C_1_pSK_SK	590
			pOP-CNIP00869_EST_C_1_pSK_SK	499
			pOP-CNIP00870_EST_C_1_pSK_SK	517
			pOP-CNIP00874_EST_C_1_pSK_SK	540
			pOP-CNIP00875_EST_C_1_pSK_SK	391
			pOP-CNIP00876_EST_C_1_pSK_SK	289
			pOP-CNIP00877_EST_C_1_pSK_SK	677
			pOP-CNIP00883_EST_C_1_pSK_SK	424
			pOP-CNIP00886_EST_C_1_pSK_SK	457
			pOP-CNIP00888_EST_C_1_pSK_SK	394

			pOP-CNIP00890_EST_C_1_pSK_SK	258
			pOP-CNIP00891_EST_C_1_pSK_SK	201
			pOP-CNIP00892_EST_C_1_pSK_SK	251
			pOP-CNIP00893_EST_C_1_pSK_SK	171
			pOP-CNIP00894_EST_C_1_pSK_SK	110
			pOP-CNIP00907_EST_C_1_pSK_SK	154
			pOP-CNIP00908_EST_C_1_pSK_SK	569
			pOP-CNIP00909_EST_C_1_pSK_SK	368
			pOP-CNIP00915_EST_C_1_pSK_SK	465
			pOP-CNIP00936_EST_C_1_pSK_SK	648
			pOP-CNIP00951_EST_C_1_pSK_SK	287
			pOP-CNIP00953_EST_C_1_pSK_SK	629
			pOP-CNIP00960_EST_C_1_pSK_SK	253
			pOP-CNIP00964_EST_C_1_pSK_SK	481
			pOP-CNIP00973_EST_C_1_pSK_SK	388
			pOP-CNIP00976_EST_C_1_pSK_SK	266
			pOP-CNIP00977_EST_C_1_pSK_SK	473
			pOP-CNIP00983_EST_C_1_pSK_SK	371
			pOP-CNIP00984_EST_C_1_pSK_SK	403
			pOP-CNIP00985_EST_C_1_pSK_SK	708
			pOP-CNIP00989_EST_C_1_pSK_SK	355
			pOP-CNIP01001_EST_C_1_pSK_SK	218
			pOP-CNIP01002_EST_C_1_pSK_SK	237
			pOP-CNIP01009_EST_C_1_pSK_SK	586
			pOP-CNIP01012_EST_C_1_pSK_SK	365
			pOP-CNIP01018_EST_C_1_pSK_SK	325
			pOP-CNIP01021_EST_C_1_pSK_SK	368
			pOP-CNIP01024_EST_C_1_pSK_SK	290
			pOP-CNIP01028_EST_C_1_pSK_SK	685
			pOP-CNIP01032_EST_C_1_pSK_SK	522
			pOP-CNIP01035_EST_C_1_pSK_SK	617
			pOP-CNIP01037_EST_C_1_pSK_SK	354
			pOP-CNIP01043_EST_C_1_pSK_SK	233
			pOP-CNIP01045_EST_C_1_pSK_SK	735
			pOP-CNIP01050_EST_C_1_pSK_SK	571
			pOP-CNIP01058_EST_C_1_pSK_SK	642
			pOP-CNIP01062_EST_C_1_pSK_SK	270
			pOP-CNIP01063_EST_C_1_pSK_SK	296
			pOP-CNIP01064_EST_C_1_pSK_SK	300
			pOP-CNIP01067_EST_C_1_pSK_SK	273
			pOP-CNIP01069_EST_C_1_pSK_SK	634
			pOP-CNIP01072_EST_C_1_pSK_SK	731
			pOP-CNIP01075_EST_C_1_pSK_SK	167
			pOP-CNIP04005_EST_C_1_pSK_SK	528
			pOP-CNIP04006_EST_C_1_pSK_SK	431
			pOP-CNIP04012_EST_C_1_pSK_SK	405
			pOP-CNIP04015_EST_C_1_pSK_SK	231
			pOP-CNIP04018_EST_C_1_pSK_SK	472
			pOP-CNIP04019_EST_C_1_pSK_SK	595
			pOP-CNIP04020_EST_C_1_pSK_SK	264
			pOP-CNIP04021_EST_C_1_pSK_SK	172
			pOP-CNIP04023_EST_C_1_pSK_SK	371
			pOP-CNIP04025_EST_C_1_pSK_SK	190
			pOP-CNIP04029_EST_C_1_pSK_SK	445
			pOP-CNIP04032_EST_C_1_pSK_SK	382
			pOP-CNIP04038_EST_C_1_pSK_SK	163

			pOP-CNIP04039_EST_C_1_pSK_SK	542
			pOP-CNIP04042_EST_C_1_pSK_SK	273
			pOP-CNIP04043_EST_C_1_pSK_SK	317
			pOP-CNIP04053_EST_C_1_pSK_SK	263
			pOP-CNIP04054_EST_C_1_pSK_SK	186
			pOP-CNIP04059_EST_C_1_pSK_SK	559
			pOP-CNIP04060_EST_C_1_pSK_SK	661
			pOP-CNIP04064_EST_C_1_pSK_SK	388
			pOP-CNIP04065_EST_C_1_pSK_SK	463
			pOP-CNIP04067_EST_C_1_pSK_SK	651
			pOP-CNIP04076_EST_C_1_pSK_SK	279
			pOP-CNIP04080_EST_C_1_pSK_SK	300
			pOP-CNIP04082_EST_C_1_pSK_SK	312
			pOP-CNIP04085_EST_C_1_pSK_SK	489
			pOP-CNIP04087_EST_C_1_pSK_SK	388
			pOP-CNIP04091_EST_C_1_pSK_SK	509
			pOP-CNLP00002_EST_C_1_pSK_SK	402
			pOP-CNLP00008_EST_C_1_pSK_SK	381
			pOP-CNLP00011_EST_C_1_pSK_SK	593
			pOP-CNLP00012_EST_C_1_pSK_SK	657
			pOP-CNLP00020_EST_C_1_pSK_SK	543
			pOP-CNLP00021_EST_C_1_pSK_SK	355
			pOP-CNLP00028_EST_C_1_pSK_SK	448
			pOP-CNNP00006_EST_C_1_pBSK_SK	397
			pOP-CNNP00008_EST_C_1_pBSK_SK	551
			pOP-CNNP00009_EST_C_1_pBSK_SK	607
			pOP-CNNP00012_EST_C_1_pBSK_SK	296
			pOP-CNNP00014_EST_C_1_pBSK_SK	354
			pOP-CNNP00015_EST_C_1_pBSK_SK	432
			pOP-CNNP00017_EST_C_1_pBSK_SK	458
			pOP-CNNP00018_EST_C_1_pBSK_SK	519
			pOP-CNNP00019_EST_C_1_pBSK_SK	632
			pOP-CNNP00021_EST_C_1_pBSK_SK	350
			pOP-CNNP00023_EST_C_1_pBSK_SK	268
			pOP-CNNP00025_EST_C_1_pBSK_SK	412
			pOP-CNNP00030_EST_C_1_pBSK_SK	578
			pOP-EAP00056_EST_C_1_pBSK_SK	472
			pOP-EAP00074_EST_C_1_pBSK_SK	415
			pOP-EAP00075_EST_C_1_pBSK_SK	563
			pOP-EAP00092_EST_C_1_pBSK_SK	602
			pOP-EAP00154_EST_C_1_pBSK_SK	211
			pOP-EAP00168_EST_C_1_pBSK_SK	512
			pOP-EAP00177_EST_C_1_pBSK_SK	223
			pOP-EAP00199_EST_C_1_pBSK_SK	501
			pOP-EAP00226_EST_C_1_pBSK_SK	144
			pOP-EAP00243_EST_C_1_pBSK_SK	162
			pOP-EAP00244_EST_C_1_pBSK_SK	171
			pOP-EAP00246_EST_C_1_pBSK_SK	454
			pOP-EAP00247_EST_C_1_pBSK_SK	397
			pOP-EAP00248_EST_C_1_pBSK_SK	154
			pOP-EAP00253_EST_C_1_pBSK_SK	485
			pOP-EAP00262_EST_C_1_pBSK_SK	245
			pOP-EAP00269_EST_C_1_pBSK_SK	530
			pOP-EAP00271_EST_C_1_pBSK_SK	480
			pOP-EAP00272_EST_C_1_pBSK_SK	160
			pOP-EAP00278_EST_C_1_pBSK_SK	438

			pOP-EAP00279_EST_C_1_pBSK_SK	437
			pOP-EAP00281_EST_C_1_pBSK_SK	405
			pOP-EAP00282_EST_C_1_pBSK_SK	278
			pOP-EAP00286_EST_C_1_pBSK_SK	471
			pOP-EAP00299_EST_C_1_pBSK_SK	376
			pOP-EAP00311_EST_C_1_pBSK_SK	112
			pOP-EAP00312_EST_C_1_pBSK_SK	272
			pOP-EAP00315_EST_C_1_pBSK_SK	161
			pOP-EAP00318_EST_C_1_pBSK_SK	189
			pOP-EAP00329_EST_C_1_pBSK_SK	220
			pOP-EAP00330_EST_C_1_pBSK_SK	260
			pOP-EAP00335_EST_C_1_pBSK_SK	438
			pOP-EAP00337_EST_C_1_pBSK_SK	127
			pOP-EAP00339_EST_C_1_pBSK_SK	137
			pOP-EAP00340_EST_C_1_pBSK_SK	302
			pOP-EAP00341_EST_C_1_pBSK_SK	258
			pOP-EAP00389_EST_C_1_pBSK_SK	259
			pOP-EAP00391_EST_C_1_pBSK_SK	132
			pOP-EAP00395_EST_C_1_pBSK_SK	178
			pOP-EAP00409_EST_C_1_pBSK_SK	487
			pOP-EAP00410_EST_C_1_pBSK_SK	192
			pOP-EAP00412_EST_C_1_pBSK_SK	311
			pOP-EAP00415_EST_C_1_pBSK_SK	318
			pOP-EAP00420_EST_C_1_pBSK_SK	194
			pOP-EAP00422_EST_C_1_pBSK_SK	280
			pOP-EAP00423_EST_C_1_pBSK_SK	285
			pOP-EAP00428_EST_C_1_pBSK_SK	418
			pOP-EAP00429_EST_C_1_pBSK_SK	131
			pOP-EAP00430_EST_C_1_pBSK_SK	472
			pOP-EAP00432_EST_C_1_pBSK_SK	497
			pOP-EAP00483_EST_C_1_pBSK_SK	180
			pOP-EAP00491_EST_C_1_pBSK_SK	138
			pOP-EAP00492_EST_C_1_pBSK_SK	623
			pOP-EAP00493_EST_C_1_pBSK_SK	513
			pOP-EAP00494_EST_C_1_pBSK_SK	371
			pOP-EAP00497_EST_C_1_pBSK_SK	600
			pOP-EAP00499_EST_C_1_pBSK_SK	481
			pOP-EAP00502_EST_C_1_pBSK_SK	635
			pOP-EAP00503_EST_C_1_pBSK_SK	463
			pOP-EAP00504_EST_C_1_pBSK_SK	642
			pOP-EAP00508_EST_C_1_pBSK_SK	621
			pOP-EAP00510_EST_C_1_pBSK_SK	412
			pOP-EAP00512_EST_C_1_pBSK_SK	619
			pOP-EAP00520_EST_C_1_pBSK_SK	222
			pOP-EAP00522_EST_C_1_pBSK_SK	636
			pOP-EAP00524_EST_C_1_pBSK_SK	105
			pOP-EAP00528_EST_C_1_pBSK_SK	627
			pOP-EAP00529_EST_C_1_pBSK_SK	636
			pOP-EAP00530_EST_C_1_pBSK_SK	371
			pOP-EAP00532_EST_C_1_pBSK_SK	641
			pOP-EAP00533_EST_C_1_pBSK_SK	445
			pOP-EAP00543_EST_C_1_pBSK_SK	579
			pOP-EAP00544_EST_C_1_pBSK_SK	290
			pOP-EAP00545_EST_C_1_pBSK_SK	646
			pOP-EAP00548_EST_C_1_pBSK_SK	610
			pOP-EAP00550_EST_C_1_pBSK_SK	512

			pOP-EAP00555_EST_C_1_pBSK_SK	582	
			pOP-EAP00556_EST_C_1_pBSK_SK	625	
			pOP-EAP00565_EST_C_1_pBSK_SK	233	
			pOP-EAP00578_EST_C_1_pBSK_SK	323	
			pOP-EAP00599_EST_C_1_pBSK_SK	267	
			pOP-EAP00600_EST_C_1_pBSK_SK	464	
			pOP-EAP00601_EST_C_1_pBSK_SK	610	
			pOP-EAP00603_EST_C_1_pBSK_SK	634	
			pOP-EAP00604_EST_C_1_pBSK_SK	580	
			pOP-EAP00608_EST_C_1_pBSK_SK	357	
			pOP-EAP00614_EST_C_1_pBSK_SK	614	
			pOP-EAP00621_EST_C_1_pBSK_SK	596	
			pOP-EAP00624_EST_C_1_pBSK_SK	658	
			pOP-EAP00628_EST_C_1_pBSK_SK	384	
			pOP-EAP00630_EST_C_1_pBSK_SK	390	
			pOP-EAP00634_EST_C_1_pBSK_SK	447	
			pOP-EAP00647_EST_C_1_pBSK_SK	278	
			pOP-EAP00651_EST_C_1_pBSK_SK	588	
			pOP-EAP00652_EST_C_1_pBSK_SK	625	
			pOP-EAP00659_EST_C_1_pBSK_SK	627	
			pOP-EAP00667_EST_C_1_pBSK_SK	633	
			pOP-EAP00670_EST_C_1_pBSK_SK	607	
			pOP-EAP00673_EST_C_1_pBSK_SK	630	
			pOP-EAP00674_EST_C_1_pBSK_SK	247	
			pOP-EAP00679_EST_C_1_pBSK_SK	494	
			pOP-EAP00683_EST_C_1_pBSK_SK	611	
			pOP-EAP00684_EST_C_1_pBSK_SK	375	
			pOP-EAP00687_EST_C_1_pBSK_SK	178	
			pOP-EAP00695_EST_C_1_pBSK_SK	620	
			pOP-EAP00697_EST_C_1_pBSK_SK	298	
			pOP-EAP00698_EST_C_1_pBSK_SK	623	
			pOP-EAP00701_EST_C_1_pBSK_SK	539	
			pOP-EAP00706_EST_C_1_pBSK_SK	551	
			pOP-EAP00707_EST_C_1_pBSK_SK	230	
			pOP-EAP00711_EST_C_1_pBSK_SK	516	
			pOP-EAP00712_EST_C_1_pBSK_SK	419	
			pOP-EAP00715_EST_C_1_pBSK_SK	598	
			pOP-EAP00718_EST_C_1_pBSK_SK	550	
			pOP-EAP00719_EST_C_1_pBSK_SK	308	
			pOP-EAP00720_EST_C_1_pBSK_SK	126	
			pOP-EAP00730_EST_C_1_pBSK_SK	529	
			pOP-EAP00735_EST_C_1_pBSK_SK	363	
			pOP-EAP00737_EST_C_1_pBSK_SK	408	
			pOP-EAP00739_EST_C_1_pBSK_SK	541	
			pOP-EAP00741_EST_C_1_pBSK_SK	397	
			pOP-EAP00746_EST_C_1_pBSK_SK	608	
			pOP-EAP00747_EST_C_1_pBSK_SK	503	
			pOP-EAP00752_EST_C_1_pBSK_SK	524	
			pOP-EAP00763_EST_C_1_pBSK_SK	465	
			pOP-EAP00768_EST_C_1_pBSK_SK	438	
			pOP-EAP00772_EST_C_1_pBSK_SK	442	
			pOP-EAP00775_EST_C_1_pBSK_SK	538	
			pOP-EAP00777_EST_C_1_pBSK_SK	607	
			pOP-EAP00779_EST_C_1_pBSK_SK	496	
			pOP-EAP00784_EST_C_1_pBSK_SK	402	
			pOP-EAP00785_EST_C_1_pBSK_SK	527	

			pOP-EAP00789_EST_C_1_pBSK_SK	384
			pOP-EAP00790_EST_C_1_pBSK_SK	231
			pOP-EAP00792_EST_C_1_pBSK_SK	411
			pOP-EAP00798_EST_C_1_pBSK_SK	409
			pOP-EAP00801_EST_C_1_pBSK_SK	408
			pOP-EAP00802_EST_C_1_pBSK_SK	395
			pOP-EAP00803_EST_C_1_pBSK_SK	448
			pOP-EAP00809_EST_C_1_pBSK_SK	320
			pOP-EAP00811_EST_C_1_pBSK_SK	261
			pOP-EAP00813_EST_C_1_pBSK_SK	399
			pOP-EAP00815_EST_C_1_pBSK_SK	336
			pOP-EAP00818_EST_C_1_pBSK_SK	274
			pOP-EAP00822_EST_C_1_pBSK_SK	410
			pOP-EAP00827_EST_C_1_pBSK_SK	264
			pOP-EAP00830_EST_C_1_pBSK_SK	425
			pOP-EAP00832_EST_C_1_pBSK_SK	552
			pOP-EAP00833_EST_C_1_pBSK_SK	538
			pOP-EAP00841_EST_C_1_pBSK_SK	562
			pOP-EAP00844_EST_C_1_pBSK_SK	576
			pOP-EAP00847_EST_C_1_pBSK_SK	462
			pOP-EAP00848_EST_C_1_pBSK_SK	338
			pOP-EAP00849_EST_C_1_pBSK_SK	329
			pOP-EAP00851_EST_C_1_pBSK_SK	316
			pOP-EAP00852_EST_C_1_pBSK_SK	250
			pOP-EAP00868_EST_C_1_pBSK_SK	370
			pOP-EAP00871_EST_C_1_pBSK_SK	350
			pOP-EAP00875_EST_C_1_pBSK_SK	360
			pOP-EAP00876_EST_C_1_pBSK_SK	196
			pOP-EAP00879_EST_C_1_pBSK_SK	350
			pOP-EAP00881_EST_C_1_pBSK_SK	273
			pOP-EAP00883_EST_C_1_pBSK_SK	247
			pOP-EAP00884_EST_C_1_pBSK_SK	515
			pOP-EAP00886_EST_C_1_pBSK_SK	109
			pOP-EAP00887_EST_C_1_pBSK_SK	160
			pOP-EAP00889_EST_C_1_pBSK_SK	332
			pOP-EAP00890_EST_C_1_pBSK_SK	321
			pOP-EAP00893_EST_C_1_pBSK_SK	434
			pOP-EAP00897_EST_C_1_pBSK_SK	244
			pOP-EAP00898_EST_C_1_pBSK_SK	254
			pOP-EAP00900_EST_C_1_pBSK_SK	171
			pOP-EAP00908_EST_C_1_pBSK_SK	317
			pOP-EAP00909_EST_C_1_pBSK_SK	148
			pOP-EAP00911_EST_C_1_pBSK_SK	126
			pOP-EAP00912_EST_C_1_pBSK_SK	263
			pOP-EAP00914_EST_C_1_pBSK_SK	178
			pOP-EAP00916_EST_C_1_pBSK_SK	225
			pOP-EAP00918_EST_C_1_pBSK_SK	596
			pOP-EAP00922_EST_C_1_pBSK_SK	334
			pOP-EAP00924_EST_C_1_pBSK_SK	111
			pOP-EAP00928_EST_C_1_pBSK_SK	327
			pOP-EAP00929_EST_C_1_pBSK_SK	606
			pOP-EAP00930_EST_C_1_pBSK_SK	210
			pOP-EAP00931_EST_C_1_pBSK_SK	433
			pOP-EAP00932_EST_C_1_pBSK_SK	148
			pOP-EAP00934_EST_C_1_pBSK_SK	292
			pOP-EAP00936_EST_C_1_pBSK_SK	156

			pOP-EAP00938_EST_C_1_pBSK_SK	361
			pOP-EAP00941_EST_C_1_pBSK_SK	140
			pOP-EAP00947_EST_C_1_pBSK_SK	440
			pOP-EAP00950_EST_C_1_pBSK_SK	414
			pOP-EAP00952_EST_C_1_pBSK_SK	266
			pOP-EAP00954_EST_C_1_pBSK_SK	174
			pOP-EAP00955_EST_C_1_pBSK_SK	473
			pOP-EAP00958_EST_C_1_pBSK_SK	147
			pOP-EAP00960_EST_C_1_pBSK_SK	236
			pOP-EAP00966_EST_C_1_pBSK_SK	225
			pOP-EAP00990_EST_C_1_pBSK_SK	250
			pOP-EAP00994_EST_C_1_pBSK_SK	257
			pOP-EAP00995_EST_C_1_pBSK_SK	116
			pOP-EAP00996_EST_C_1_pBSK_SK	364
			pOP-EAP00997_EST_C_1_pBSK_SK	483
			pOP-EAP01002_EST_C_1_pBSK_SK	425
			pOP-EAP01006_EST_C_1_pBSK_SK	298
			pOP-EAP01008_EST_C_1_pBSK_SK	519
			pOP-EAP01009_EST_C_1_pBSK_SK	100
			pOP-EAP01010_EST_C_1_pBSK_SK	403
			pOP-EAP01011_EST_C_1_pBSK_SK	170
			pOP-EAP01012_EST_C_1_pBSK_SK	412
			pOP-EAP01018_EST_C_1_pBSK_SK	185
			pOP-EAP01019_EST_C_1_pBSK_SK	328
			pOP-EAP01021_EST_C_1_pBSK_SK	184
			pOP-EAP01022_EST_C_1_pBSK_SK	285
			pOP-EAP01024_EST_C_1_pBSK_SK	601
			pOP-EAP01044_EST_C_1_pBSK_SK	356
			pOP-EAP01052_EST_C_1_pBSK_SK	134
			pOP-EAP01054_EST_C_1_pBSK_SK	368
			pOP-EAP01064_EST_C_1_pBSK_SK	416
			pOP-EAP01074_EST_C_1_pBSK_SK	176
			pOP-EAP01081_EST_C_1_pBSK_SK	526
			pOP-EAP01082_EST_C_1_pBSK_SK	192
			pOP-EAP01091_EST_C_1_pBSK_SK	104
			pOP-EAP01094_EST_C_1_pBSK_SK	146
			pOP-EAP01104_EST_C_1_pBSK_SK	121
			pOP-EAP01108_EST_C_1_pBSK_SK	147
			pOP-EAP01113_EST_C_1_pBSK_SK	567
			pOP-EAP01116_EST_C_1_pBSK_SK	380
			pOP-EAP01119_EST_C_1_pBSK_SK	539
			pOP-EAP01120_EST_C_1_pBSK_SK	133
			pOP-EAP01124_EST_C_1_pBSK_SK	107
			pOP-EAP01128_EST_C_1_pBSK_SK	350
			pOP-EAP01132_EST_C_1_pBSK_SK	183
			pOP-EAP01135_EST_C_1_pBSK_SK	150
			pOP-EAP01151_EST_C_1_pBSK_SK	191
			pOP-EAP01167_EST_C_1_pBSK_SK	332
			pOP-EAP01168_EST_C_1_pBSK_SK	334
			pOP-EAP01169_EST_C_1_pBSK_SK	595
			pOP-EAP01176_EST_C_1_pBSK_SK	140
			pOP-EAP01191_EST_C_1_pBSK_SK	176
			pOP-EAP01192_EST_C_1_pBSK_SK	252
			pOP-EAP01195_EST_C_1_pBSK_SK	644
			pOP-EAP01199_EST_C_1_pBSK_SK	643
			pOP-EAP01200_EST_C_1_pBSK_SK	438

			pOP-EAP01202_EST_C_1_pBSK_SK	372
			pOP-EAP01206_EST_C_1_pBSK_SK	513
			pOP-EAP01207_EST_C_1_pBSK_SK	603
			pOP-EAP01211_EST_C_1_pBSK_SK	333
			pOP-EAP01215_EST_C_1_pBSK_SK	313
			pOP-EAP01221_EST_C_1_pBSK_SK	214
			pOP-EAP01222_EST_C_1_pBSK_SK	229
			pOP-EAP01225_EST_C_1_pBSK_SK	537
			pOP-EAP01229_EST_C_1_pBSK_SK	187
			pOP-EAP01230_EST_C_1_pBSK_SK	148
			pOP-EAP01233_EST_C_1_pBSK_SK	347
			pOP-EAP01241_EST_C_1_pBSK_SK	306
			pOP-EAP01248_EST_C_1_pBSK_SK	116
			pOP-EAP01249_EST_C_1_pBSK_SK	305
			pOP-EAP01250_EST_C_1_pBSK_SK	188
			pOP-EAP01254_EST_C_1_pBSK_SK	288
			pOP-EAP01263_EST_C_1_pBSK_SK	305
			pOP-EAP01266_EST_C_1_pBSK_SK	519
			pOP-EAP01267_EST_C_1_pBSK_SK	103
			pOP-EAP01271_EST_C_1_pBSK_SK	257
			pOP-EAP01279_EST_C_1_pBSK_SK	349
			pOP-EAP01288_EST_C_1_pBSK_SK	600
			pOP-EAP01293_EST_C_1_pBSK_SK	482
			pOP-EAP01294_EST_C_1_pBSK_SK	323
			pOP-EAP01302_EST_C_1_pBSK_SK	595
			pOP-EAP01304_EST_C_1_pBSK_SK	205
			pOP-EAP01306_EST_C_1_pBSK_SK	194
			pOP-EAP01307_EST_C_1_pBSK_SK	172
			pOP-EAP01313_EST_C_1_pBSK_SK	380
			pOP-EAP01317_EST_C_1_pBSK_SK	604
			pOP-EAP01320_EST_C_1_pBSK_SK	482
			pOP-EAP01325_EST_C_1_pBSK_SK	444
			pOP-EAP01332_EST_C_1_pBSK_SK	618
			pOP-EAP01333_EST_C_1_pBSK_SK	306
			pOP-EAP01339_EST_C_1_pBSK_SK	355
			pOP-EAP01341_EST_C_1_pBSK_SK	437
			pOP-EAP01344_EST_C_1_pBSK_SK	232
			pOP-EAP01345_EST_C_1_pBSK_SK	607
			pOP-EAP01347_EST_C_1_pBSK_SK	422
			pOP-EAP01348_EST_C_1_pBSK_SK	307
			pOP-EAP01351_EST_C_1_pBSK_SK	526
			pOP-EAP01353_EST_C_1_pBSK_SK	206
			pOP-EAP01354_EST_C_1_pBSK_SK	241
			pOP-EAP01356_EST_C_1_pBSK_SK	101
			pOP-EAP01358_EST_C_1_pBSK_SK	178
			pOP-EAP01366_EST_C_1_pBSK_SK	263
			pOP-EAP01367_EST_C_1_pBSK_SK	452
			pOP-EAP01369_EST_C_1_pBSK_SK	249
			pOP-EAP01377_EST_C_1_pBSK_SK	386
			pOP-EAP01379_EST_C_1_pBSK_SK	278
			pOP-EAP01380_EST_C_1_pBSK_SK	165
			pOP-EAP01381_EST_C_1_pBSK_SK	287
			pOP-EAP01382_EST_C_1_pBSK_SK	366
			pOP-EAP01384_EST_C_1_pBSK_SK	256
			pOP-EAP01385_EST_C_1_pBSK_SK	308
			pOP-EAP01386_EST_C_1_pBSK_SK	244

			pOP-EAP01390_EST_C_1_pBSK_SK	439
			pOP-EAP01391_EST_C_1_pBSK_SK	214
			pOP-EAP01401_EST_C_1_pBSK_SK	325
			pOP-EAP01404_EST_C_1_pBSK_SK	348
			pOP-EAP01415_EST_C_1_pBSK_SK	184
			pOP-EAP01420_EST_C_1_pBSK_SK	233
			pOP-EAP01423_EST_C_1_pBSK_SK	111
			pOP-EAP01425_EST_C_1_pBSK_SK	499
			pOP-EAP01428_EST_C_1_pBSK_SK	445
			pOP-EAP01430_EST_C_1_pBSK_SK	220
			pOP-EAP01431_EST_C_1_pBSK_SK	421
			pOP-EAP01435_EST_C_1_pBSK_SK	268
			pOP-EAP01437_EST_C_1_pBSK_SK	396
			pOP-EAP01457_EST_C_1_pBSK_SK	425
			pOP-EAP01459_EST_C_1_pBSK_SK	479
			pOP-EAP01460_EST_C_1_pBSK_SK	647
			pOP-EAP01461_EST_C_1_pBSK_SK	654
			pOP-EAP01466_EST_C_1_pBSK_SK	593
			pOP-EAP01469_EST_C_1_pBSK_SK	653
			pOP-EAP01473_EST_C_1_pBSK_SK	387
			pOP-EAP01474_EST_C_1_pBSK_SK	654
			pOP-EAP01477_EST_C_1_pBSK_SK	454
			pOP-EAP01485_EST_C_1_pBSK_SK	552
			pOP-EAP01486_EST_C_1_pBSK_SK	487
			pOP-EAP01489_EST_C_1_pBSK_SK	602
			pOP-EAP01490_EST_C_1_pBSK_SK	564
			pOP-EAP01496_EST_C_1_pBSK_SK	547
			pOP-EAP01499_EST_C_1_pBSK_SK	407
			pOP-EAP01506_EST_C_1_pBSK_SK	313
			pOP-EAP01512_EST_C_1_pBSK_SK	283
			pOP-EAP01520_EST_C_1_pBSK_SK	547
			pOP-EAP01523_EST_C_1_pBSK_SK	382
			pOP-EAP01527_EST_C_1_pBSK_SK	490
			pOP-EAP01530_EST_C_1_pBSK_SK	623
			pOP-EAP01531_EST_C_1_pBSK_SK	521
			pOP-EAP01534_EST_C_1_pBSK_SK	410
			pOP-EAP01537_EST_C_1_pBSK_SK	529
			pOP-EAP01538_EST_C_1_pBSK_SK	315
			pOP-EAP01553_EST_C_1_pBSK_SK	404
			pOP-EAP01556_EST_C_1_pBSK_SK	566
			pOP-EAP01559_EST_C_1_pBSK_SK	271
			pOP-EAP01560_EST_C_1_pBSK_SK	517
			pOP-EAP01561_EST_C_1_pBSK_SK	329
			pOP-EAP01565_EST_C_1_pBSK_SK	568
			pOP-EAP01573_EST_C_1_pBSK_SK	429
			pOP-EAP01574_EST_C_1_pBSK_SK	454
			pOP-EAP01575_EST_C_1_pBSK_SK	455
			pOP-EAP01580_EST_C_1_pBSK_SK	192
			pOP-EAP01581_EST_C_1_pBSK_SK	563
			pOP-EAP01584_EST_C_1_pBSK_SK	393
			pOP-EAP01585_EST_C_1_pBSK_SK	460
			pOP-EAP01586_EST_C_1_pBSK_SK	480
			pOP-EAP01587_EST_C_1_pBSK_SK	438
			pOP-EAP01588_EST_C_1_pBSK_SK	258
			pOP-EAP01590_EST_C_1_pBSK_SK	293
			pOP-EAP01597_EST_C_1_pBSK_SK	458

			pOP-EAP01602_EST_C_1_pBSK_SK	413
			pOP-EAP01608_EST_C_1_pBSK_SK	648
			pOP-EAP01611_EST_C_1_pBSK_SK	325
			pOP-EAP01613_EST_C_1_pBSK_SK	493
			pOP-EAP01621_EST_C_1_pBSK_SK	351
			pOP-EAP01622_EST_C_1_pBSK_SK	472
			pOP-EAP01628_EST_C_1_pBSK_SK	656
			pOP-EAP01632_EST_C_1_pBSK_SK	470
			pOP-EAP01635_EST_C_1_pBSK_SK	668
			pOP-EAP01636_EST_C_1_pBSK_SK	257
			pOP-EAP01640_EST_C_1_pBSK_SK	412
			pOP-EAP01641_EST_C_1_pBSK_SK	619
			pOP-EAP01643_EST_C_1_pBSK_SK	371
			pOP-EAP01646_EST_C_1_pBSK_SK	572
			pOP-EAP01647_EST_C_1_pBSK_SK	398
			pOP-EAP01649_EST_C_1_pBSK_SK	627
			pOP-EAP01651_EST_C_1_pBSK_SK	368
			pOP-EAP01653_EST_C_1_pBSK_SK	396
			pOP-EAP01655_EST_C_1_pBSK_SK	365
			pOP-EAP01664_EST_C_1_pBSK_SK	457
			pOP-EAP01667_EST_C_1_pBSK_SK	642
			pOP-EAP01668_EST_C_1_pBSK_SK	648
			pOP-EAP01669_EST_C_1_pBSK_SK	557
			pOP-EAP01670_EST_C_1_pBSK_SK	672
			pOP-EAP01672_EST_C_1_pBSK_SK	596
			pOP-EAP01673_EST_C_1_pBSK_SK	402
			pOP-EAP01674_EST_C_1_pBSK_SK	305
			pOP-EAP01675_EST_C_1_pBSK_SK	445
			pOP-EAP01682_EST_C_1_pBSK_SK	457
			pOP-EAP01683_EST_C_1_pBSK_SK	485
			pOP-EAP01685_EST_C_1_pBSK_SK	639
			pOP-EAP01692_EST_C_1_pBSK_SK	672
			pOP-EAP01695_EST_C_1_pBSK_SK	164
			pOP-EAP01697_EST_C_1_pBSK_SK	636
			pOP-EAP01700_EST_C_1_pBSK_SK	465
			pOP-EAP01701_EST_C_1_pBSK_SK	317
			pOP-EAP01703_EST_C_1_pBSK_SK	521
			pOP-EAP01707_EST_C_1_pBSK_SK	261
			pOP-EAP01708_EST_C_1_pBSK_SK	596
			pOP-EAP01711_EST_C_1_pBSK_SK	279
			pOP-EAP01719_EST_C_1_pBSK_SK	437
			pOP-EAP01726_EST_C_1_pBSK_SK	724
			pOP-EAP01729_EST_C_1_pBSK_SK	600
			pOP-EAP01733_EST_C_1_pBSK_SK	411
			pOP-EAP01735_EST_C_1_pBSK_SK	679
			pOP-EAP01738_EST_C_1_pBSK_SK	456
			pOP-EAP01744_EST_C_1_pBSK_SK	476
			pOP-EAP01745_EST_C_1_pBSK_SK	263
			pOP-EAP01748_EST_C_1_pBSK_SK	480
			pOP-EAP01750_EST_C_1_pBSK_SK	717
			pOP-EAP01753_EST_C_1_pBSK_SK	253
			pOP-EAP01754_EST_C_1_pBSK_SK	547
			pOP-EAP01784_EST_C_1_pBSK_SK	299
			pOP-EAP01785_EST_C_1_pBSK_SK	352
			pOP-EAP01788_EST_C_1_pBSK_SK	752
			pOP-EAP01789_EST_C_1_pBSK_SK	654

			pOP-EAP01791_EST_C_1_pBSK_SK	259
			pOP-EAP01796_EST_C_1_pBSK_SK	678
			pOP-EAP01802_EST_C_1_pBSK_SK	577
			pOP-EAP01806_EST_C_1_pBSK_SK	732
			pOP-EAP01807_EST_C_1_pBSK_SK	681
			pOP-EAP01810_EST_C_1_pBSK_SK	575
			pOP-EAP01816_EST_C_1_pBSK_SK	447
			pOP-EAP01818_EST_C_1_pBSK_SK	693
			pOP-EAP01823_EST_C_1_pBSK_SK	720
			pOP-EAP01834_EST_C_1_pBSK_SK	538
			pOP-EAP01836_EST_C_1_pBSK_SK	668
			pOP-EAP01840_EST_C_1_pBSK_SK	284
			pOP-EAP01844_EST_C_1_pBSK_SK	531
			pOP-EAP01845_EST_C_1_pBSK_SK	724
			pOP-EAP01846_EST_C_1_pBSK_SK	462
			pOP-EAP01847_EST_C_1_pBSK_SK	598
			pOP-EAP01853_EST_C_1_pBSK_SK	346
			pOP-EAP01860_EST_C_1_pBSK_SK	668
			pOP-EAP01862_EST_C_1_pBSK_SK	712
			pOP-EAP01863_EST_C_1_pBSK_SK	712
			pOP-EAP01869_EST_C_1_pBSK_SK	528
			pOP-EAP01874_EST_C_1_pBSK_SK	627
			pOP-EAP01878_EST_C_1_pBSK_SK	188
			pOP-EAP01879_EST_C_1_pBSK_SK	341
			pOP-EAP01880_EST_C_1_pBSK_SK	477
			pOP-EAP01886_EST_C_1_pBSK_SK	606
			pOP-EAP01892_EST_C_1_pBSK_SK	544
			pOP-EAP01901_EST_C_1_pBSK_SK	132
			pOP-EAP01903_EST_C_1_pBSK_SK	602
			pOP-EAP01904_EST_C_1_pBSK_SK	228
			pOP-EAP01906_EST_C_1_pBSK_SK	496
			pOP-EAP01911_EST_C_1_pBSK_SK	468
			pOP-EAP01915_EST_C_1_pBSK_SK	494
			pOP-EAP01920_EST_C_1_pBSK_SK	390
			pOP-EAP01921_EST_C_1_pBSK_SK	227
			pOP-EAP01927_EST_C_1_pBSK_SK	237
			pOP-EAP01935_EST_C_1_pBSK_SK	306
			pOP-EAP01937_EST_C_1_pBSK_SK	345
			pOP-EAP01938_EST_C_1_pBSK_SK	390
			pOP-EAP01940_EST_C_1_pBSK_SK	268
			pOP-EAP01947_EST_C_1_pBSK_SK	368
			pOP-EAP01950_EST_C_1_pBSK_SK	264
			pOP-EAP01955_EST_C_1_pBSK_SK	415
			pOP-EAP01957_EST_C_1_pBSK_SK	532
			pOP-EAP01961_EST_C_1_pBSK_SK	227
			pOP-EAP01962_EST_C_1_pBSK_SK	322
			pOP-EAP01965_EST_C_1_pBSK_SK	451
			pOP-EAP01967_EST_C_1_pBSK_SK	362
			pOP-EAP01969_EST_C_1_pBSK_SK	234
			pOP-EAP01973_EST_C_1_pBSK_SK	402
			pOP-EAP01977_EST_C_1_pBSK_SK	238
			pOP-EAP01981_EST_C_1_pBSK_SK	428
			pOP-EAP01988_EST_C_1_pBSK_SK	321
			pOP-EAP01996_EST_C_1_pBSK_SK	307
			pOP-EAP01997_EST_C_1_pBSK_SK	249
			pOP-EAP01998_EST_C_1_pBSK_SK	285

			pOP-EAP01999_EST_C_1_pBSK_SK	213
			pOP-EAP02001_EST_C_1_pBSK_SK	307
			pOP-EAP02002_EST_C_1_pBSK_SK	205
			pOP-EAP02003_EST_C_1_pBSK_SK	247
			pOP-EAP02004_EST_C_1_pBSK_SK	260
			pOP-EAP02005_EST_C_1_pBSK_SK	121
			pOP-EAP02006_EST_C_1_pBSK_SK	263
			pOP-EAP02008_EST_C_1_pBSK_SK	311
			pOP-EAP02011_EST_C_1_pBSK_SK	301
			pOP-EAP02012_EST_C_1_pBSK_SK	368
			pOP-EAP02013_EST_C_1_pBSK_SK	427
			pOP-EAP02015_EST_C_1_pBSK_SK	273
			pOP-EAP02016_EST_C_1_pBSK_SK	151
			pOP-EAP02019_EST_C_1_pBSK_SK	177
			pOP-EAP02020_EST_C_1_pBSK_SK	255
			pOP-EAP02021_EST_C_1_pBSK_SK	127
			pOP-EAP02023_EST_C_1_pBSK_SK	361
			pOP-EAP02058_EST_C_1_pBSK_SK	489
			pOP-EAP02063_EST_C_1_pBSK_SK	265
			pOP-EAP02072_EST_C_1_pBSK_SK	238
			pOP-EAP02075_EST_C_1_pBSK_SK	405
			pOP-EAP02076_EST_C_1_pBSK_SK	402
			pOP-EAP02079_EST_C_1_pBSK_SK	334
			pOP-EAP02084_EST_C_1_pBSK_SK	201
			pOP-EAP02089_EST_C_1_pBSK_SK	186
			pOP-EAP02090_EST_C_1_pBSK_SK	384
			pOP-EAP02092_EST_C_1_pBSK_SK	158
			pOP-EAP02102_EST_C_1_pBSK_SK	108
			pOP-EAP02107_EST_C_1_pBSK_SK	172
			pOP-EAP02108_EST_C_1_pBSK_SK	295
			pOP-EAP02110_EST_C_1_pBSK_SK	385
			pOP-EAP02113_EST_C_1_pBSK_SK	339
			pOP-EAP02115_EST_C_1_pBSK_SK	331
			pOP-EAP02117_EST_C_1_pBSK_SK	242
			pOP-EAP02125_EST_C_1_pBSK_SK	393
			pOP-EAP02130_EST_C_1_pBSK_SK	215
			pOP-EAP02132_EST_C_1_pBSK_SK	320
			pOP-EAP02134_EST_C_1_pBSK_SK	111
			pOP-EAP02135_EST_C_1_pBSK_SK	265
			pOP-EAP02136_EST_C_1_pBSK_SK	272
			pOP-EAP02139_EST_C_1_pBSK_SK	109
			pOP-EAP02141_EST_C_1_pBSK_SK	368
			pOP-EAP02145_EST_C_1_pBSK_SK	188
			pOP-EAP02146_EST_C_1_pBSK_SK	110
			pOP-EAP02152_EST_C_1_pBSK_SK	649
			pOP-EAP02159_EST_C_1_pBSK_SK	315
			pOP-EAP02160_EST_C_1_pBSK_SK	438
			pOP-EAP02162_EST_C_1_pBSK_SK	272
			pOP-EAP02165_EST_C_1_pBSK_SK	403
			pOP-EAP02167_EST_C_1_pBSK_SK	183
			pOP-EAP02170_EST_C_1_pBSK_SK	300
			pOP-EAP02172_EST_C_1_pBSK_SK	301
			pOP-EAP02173_EST_C_1_pBSK_SK	679
			pOP-EAP02181_EST_C_1_pBSK_SK	528
			pOP-EAP02182_EST_C_1_pBSK_SK	552
			pOP-EAP02186_EST_C_1_pBSK_SK	288

			pOP-EAP02188_EST_C_1_pBSK_SK	233
			pOP-EAP02195_EST_C_1_pBSK_SK	167
			pOP-EAP02198_EST_C_1_pBSK_SK	135
			pOP-EAP02199_EST_C_1_pBSK_SK	220
			pOP-EAP02204_EST_C_1_pBSK_SK	291
			pOP-EAP02205_EST_C_1_pBSK_SK	577
			pOP-EAP02206_EST_C_1_pBSK_SK	439
			pOP-EAP02207_EST_C_1_pBSK_SK	135
			pOP-EAP02208_EST_C_1_pBSK_SK	548
			pOP-EAP02212_EST_C_1_pBSK_SK	290
			pOP-EAP02215_EST_C_1_pBSK_SK	285
			pOP-EAP02216_EST_C_1_pBSK_SK	304
			pOP-EAP02226_EST_C_1_pBSK_SK	429
			pOP-EAP02227_EST_C_1_pBSK_SK	348
			pOP-EAP02228_EST_C_1_pBSK_SK	218
			pOP-EAP02231_EST_C_1_pBSK_SK	624
			pOP-EAP02236_EST_C_1_pBSK_SK	337
			pOP-EAP02250_EST_C_1_pBSK_SK	320
			pOP-EAP02252_EST_C_1_pBSK_SK	198
			pOP-EAP02253_EST_C_1_pBSK_SK	331
			pOP-EAP02260_EST_C_1_pBSK_SK	644
			pOP-EAP02263_EST_C_1_pBSK_SK	470
			pOP-EAP02264_EST_C_1_pBSK_SK	414
			pOP-EAP02268_EST_C_1_pBSK_SK	131
			pOP-EAP02271_EST_C_1_pBSK_SK	561
			pOP-EAP02276_EST_C_1_pBSK_SK	240
			pOP-EAP02277_EST_C_1_pBSK_SK	108
			pOP-EAP02283_EST_C_1_pBSK_SK	237
			pOP-EAP02291_EST_C_1_pBSK_SK	699
			pOP-EAP02293_EST_C_1_pBSK_SK	389
			pOP-EAP02294_EST_C_1_pBSK_SK	336
			pOP-EAP02299_EST_C_1_pBSK_SK	654
			pOP-EAP02300_EST_C_1_pBSK_SK	719
			pOP-EAP02302_EST_C_1_pBSK_SK	518
			pOP-EAP02305_EST_C_1_pBSK_SK	363
			pOP-EAP02308_EST_C_1_pBSK_SK	589
			pOP-EAP02309_EST_C_1_pBSK_SK	572
			pOP-EAP02310_EST_C_1_pBSK_SK	699
			pOP-EAP02311_EST_C_1_pBSK_SK	580
			pOP-EAP02313_EST_C_1_pBSK_SK	466
			pOP-EAP02316_EST_C_1_pBSK_SK	667
			pOP-EAP02322_EST_C_1_pBSK_SK	463
			pOP-EAP02324_EST_C_1_pBSK_SK	337
			pOP-EAP02331_EST_C_1_pBSK_SK	774
			pOP-EAP02333_EST_C_1_pBSK_SK	713
			pOP-EAP02336_EST_C_1_pBSK_SK	480
			pOP-EAP02339_EST_C_1_pBSK_SK	400
			pOP-EAP02342_EST_C_1_pBSK_SK	700
			pOP-EAP02352_EST_C_1_pBSK_SK	764
			pOP-EAP02359_EST_C_1_pBSK_SK	544
			pOP-EAP02361_EST_C_1_pBSK_SK	679
			pOP-EAP02363_EST_C_1_pBSK_SK	596
			pOP-EAP02365_EST_C_1_pBSK_SK	606
			pOP-EAP02366_EST_C_1_pBSK_SK	748
			pOP-EAP02384_EST_C_1_pBSK_SK	576
			pOP-EAP02390_EST_C_1_pBSK_SK	460

			pOP-EAP02392_EST_C_1_pBSK_SK	620
			pOP-EAP02393_EST_C_1_pBSK_SK	736
			pOP-EAP02395_EST_C_1_pBSK_SK	650
			pOP-EAP02700_EST_C_1_pBSK_SK	646
			pOP-EAP02703_EST_C_1_pBSK_SK	277
			pOP-EAP02704_EST_C_1_pBSK_SK	575
			pOP-EAP02705_EST_C_1_pBSK_SK	323
			pOP-EAP02711_EST_C_1_pBSK_SK	573
			pOP-EAP02713_EST_C_1_pBSK_SK	625
			pOP-EAP02718_EST_C_1_pBSK_SK	724
			pOP-EAP02721_EST_C_1_pBSK_SK	459
			pOP-EAP02723_EST_C_1_pBSK_SK	441
			pOP-EAP02724_EST_C_1_pBSK_SK	381
			pOP-EAP02728_EST_C_1_pBSK_SK	632
			pOP-EAP02733_EST_C_1_pBSK_SK	569
			pOP-EAP02735_EST_C_1_pBSK_SK	605
			pOP-EAP02737_EST_C_1_pBSK_SK	242
			pOP-EAP02741_EST_C_1_pBSK_SK	616
			pOP-EAP02743_EST_C_1_pBSK_SK	662
			pOP-EAP02745_EST_C_1_pBSK_SK	692
			pOP-EAP02748_EST_C_1_pBSK_SK	706
			pOP-EAP02750_EST_C_1_pBSK_SK	410
			pOP-EAP02751_EST_C_1_pBSK_SK	613
			pOP-EAP02754_EST_C_1_pBSK_SK	600
			pOP-EAP02757_EST_C_1_pBSK_SK	502
			pOP-EAP02758_EST_C_1_pBSK_SK	611
			pOP-EAP02761_EST_C_1_pBSK_SK	401
			pOP-EAP02765_EST_C_1_pBSK_SK	542
			pOP-EAP02766_EST_C_1_pBSK_SK	778
			pOP-EAP02769_EST_C_1_pBSK_SK	197
			pOP-EAP02771_EST_C_1_pBSK_SK	315
			pOP-EAP02773_EST_C_1_pBSK_SK	380
			pOP-EAP02774_EST_C_1_pBSK_SK	416
			pOP-EAP02777_EST_C_1_pBSK_SK	635
			pOP-EAP02779_EST_C_1_pBSK_SK	638
			pOP-EAP02780_EST_C_1_pBSK_SK	594
			pOP-EAP02782_EST_C_1_pBSK_SK	461
			pOP-EAP02784_EST_C_1_pBSK_SK	310
			pOP-EAP02785_EST_C_1_pBSK_SK	432
			pOP-EAP02786_EST_C_1_pBSK_SK	613
			pOP-EAP02792_EST_C_1_pBSK_SK	569
			pOP-EAP02793_EST_C_1_pBSK_SK	356
			pOP-EAP02794_EST_C_1_pBSK_SK	537
			pOP-EAP02795_EST_C_1_pBSK_SK	557
			pOP-EAP02800_EST_C_1_pBSK_SK	380
			pOP-EAP02806_EST_C_1_pBSK_SK	591
			pOP-EAP02807_EST_C_1_pBSK_SK	496
			pOP-EAP02808_EST_C_1_pBSK_SK	279
			pOP-EAP02809_EST_C_1_pBSK_SK	392
			pOP-EAP02815_EST_C_1_pBSK_SK	564
			pOP-EAP02817_EST_C_1_pBSK_SK	600
			pOP-EAP02819_EST_C_1_pBSK_SK	329
			pOP-EAP02820_EST_C_1_pBSK_SK	356
			pOP-EAP02824_EST_C_1_pBSK_SK	643
			pOP-EAP02826_EST_C_1_pBSK_SK	659
			pOP-EAP02827_EST_C_1_pBSK_SK	526

			pOP-EAP02828_EST_C_1_pBSK_SK	679
			pOP-EAP02829_EST_C_1_pBSK_SK	701
			pOP-EAP02832_EST_C_1_pBSK_SK	722
			pOP-EAP02842_EST_C_1_pBSK_SK	426
			pOP-EAP02846_EST_C_1_pBSK_SK	534
			pOP-EAP02849_EST_C_1_pBSK_SK	720
			pOP-EAP02850_EST_C_1_pBSK_SK	643
			pOP-EAP02852_EST_C_1_pBSK_SK	664
			pOP-EAP02853_EST_C_1_pBSK_SK	434
			pOP-EAP02854_EST_C_1_pBSK_SK	671
			pOP-EAP02855_EST_C_1_pBSK_SK	556
			pOP-EAP02857_EST_C_1_pBSK_SK	623
			pOP-EAP02858_EST_C_1_pBSK_SK	652
			pOP-EAP02862_EST_C_1_pBSK_SK	223
			pOP-EAP02863_EST_C_1_pBSK_SK	219
			pOP-EAP02867_EST_C_1_pBSK_SK	523
			pOP-EAP02882_EST_C_1_pBSK_SK	301
			pOP-EAP02905_EST_C_1_pBSK_SK	712
			pOP-EAP02906_EST_C_1_pBSK_SK	692
			pOP-EAP02916_EST_C_1_pBSK_SK	692
			pOP-EAP02928_EST_C_1_pBSK_SK	539
			pOP-EAP02931_EST_C_1_pBSK_SK	464
			pOP-EAP02932_EST_C_1_pBSK_SK	358
			pOP-EAP02934_EST_C_1_pBSK_SK	561
			pOP-EAP02940_EST_C_1_pBSK_SK	693
			pOP-EAP02942_EST_C_1_pBSK_SK	545
			pOP-EAP02948_EST_C_1_pBSK_SK	379
			pOP-EAP02953_EST_C_1_pBSK_SK	761
			pOP-EAP02955_EST_C_1_pBSK_SK	442
			pOP-EAP02958_EST_C_1_pBSK_SK	385
			pOP-EAP02959_EST_C_1_pBSK_SK	240
			pOP-EAP02960_EST_C_1_pBSK_SK	329
			pOP-EAP02961_EST_C_1_pBSK_SK	393
			pOP-EAP02963_EST_C_1_pBSK_SK	313
			pOP-EAP02964_EST_C_1_pBSK_SK	307
			pOP-EAP02968_EST_C_1_pBSK_SK	294
			pOP-EAP02969_EST_C_1_pBSK_SK	535
			pOP-EAP02971_EST_C_1_pBSK_SK	472
			pOP-EAP02975_EST_C_1_pBSK_SK	375
			pOP-EAP02980_EST_C_1_pBSK_SK	386
			pOP-EAP02983_EST_C_1_pBSK_SK	425
			pOP-EAP02987_EST_C_1_pBSK_SK	384
			pOP-EAP02988_EST_C_1_pBSK_SK	407
			pOP-EAP02989_EST_C_1_pBSK_SK	311
			pOP-EAP02996_EST_C_1_pBSK_SK	291
			pOP-EAP02997_EST_C_1_pBSK_SK	329
			pOP-EAP02999_EST_C_1_pBSK_SK	561
			pOP-EAP03116_EST_C_1_pBSK_SK	484
			pOP-EAP03118_EST_C_1_pBSK_SK	487
			pOP-EAP03126_EST_C_1_pBSK_SK	649
			pOP-EAP03127_EST_C_1_pBSK_SK	443
			pOP-EAP03130_EST_C_1_pBSK_SK	702
			pOP-EAP03131_EST_C_1_pBSK_SK	443
			pOP-EAP03134_EST_C_1_pBSK_SK	696
			pOP-EAP03135_EST_C_1_pBSK_SK	389
			pOP-EAP03138_EST_C_1_pBSK_SK	686

			pOP-EAP03141_EST_C_1_pBSK_SK	684
			pOP-EAP03143_EST_C_1_pBSK_SK	702
			pOP-EAP03152_EST_C_1_pBSK_SK	247
			pOP-EAP03153_EST_C_1_pBSK_SK	484
			pOP-EAP03161_EST_C_1_pBSK_SK	271
			pOP-EAP03168_EST_C_1_pBSK_SK	537
			pOP-EAP03170_EST_C_1_pBSK_SK	609
			pOP-EAP03175_EST_C_1_pBSK_SK	483
			pOP-EAP03176_EST_C_1_pBSK_SK	433
			pOP-EAP03180_EST_C_1_pBSK_SK	436
			pOP-EAP03184_EST_C_1_pBSK_SK	652
			pOP-EAP03185_EST_C_1_pBSK_SK	625
			pOP-EAP03191_EST_C_1_pBSK_SK	408
			pOP-EAP03193_EST_C_1_pBSK_SK	569
			pOP-EAP03198_EST_C_1_pBSK_SK	674
			pOP-EAP03199_EST_C_1_pBSK_SK	553
			pOP-EAP03205_EST_C_1_pBSK_SK	632
			pOP-EAP03206_EST_C_1_pBSK_SK	369
			pOP-EAP03207_EST_C_1_pBSK_SK	625
			pOP-EAP03208_EST_C_1_pBSK_SK	402
			pOP-EAP03209_EST_C_1_pBSK_SK	656
			pOP-EAP03210_EST_C_1_pBSK_SK	625
			pOP-EAP03217_EST_C_1_pBSK_SK	255
			pOP-EAP03222_EST_C_1_pBSK_SK	245
			pOP-EAP03223_EST_C_1_pBSK_SK	569
			pOP-EAP03226_EST_C_1_pBSK_SK	690
			pOP-EAP03230_EST_C_1_pBSK_SK	614
			pOP-EAP03245_EST_C_1_pBSK_SK	351
			pOP-EAP03250_EST_C_1_pBSK_SK	666
			pOP-EAP03252_EST_C_1_pBSK_SK	315
			pOP-EAP03253_EST_C_1_pBSK_SK	172
			pOP-EAP03271_EST_C_1_pBSK_SK	464
			pOP-EAP03275_EST_C_1_pBSK_SK	349
			pOP-EAP03279_EST_C_1_pBSK_SK	742
			pOP-EAP03285_EST_C_1_pBSK_SK	421
			pOP-EAP03286_EST_C_1_pBSK_SK	513
			pOP-EAP03287_EST_C_1_pBSK_SK	491
			pOP-EAP03289_EST_C_1_pBSK_SK	471
			pOP-EAP03296_EST_C_1_pBSK_SK	659
			pOP-EAP03301_EST_C_1_pBSK_SK	404
			pOP-EAP03313_EST_C_1_pBSK_SK	564
			pOP-EAP03314_EST_C_1_pBSK_SK	345
			pOP-EAP03318_EST_C_1_pBSK_SK	625
			pOP-EAP03319_EST_C_1_pBSK_SK	493
			pOP-EAP03322_EST_C_1_pBSK_SK	396
			pOP-EAP03325_EST_C_1_pBSK_SK	549
			pOP-EAP03331_EST_C_1_pBSK_SK	489
			pOP-EAP03334_EST_C_1_pBSK_SK	441
			pOP-EAP03337_EST_C_1_pBSK_SK	539
			pOP-EAP03338_EST_C_1_pBSK_SK	410
			pOP-EAP03342_EST_C_1_pBSK_SK	414
			pOP-EAP03353_EST_C_1_pBSK_SK	255
			pOP-EAP03354_EST_C_1_pBSK_SK	377
			pOP-EAP03356_EST_C_1_pBSK_SK	674
			pOP-EAP03362_EST_C_1_pBSK_SK	583
			pOP-EAP03367_EST_C_1_pBSK_SK	389

			pOP-EAP03370_EST_C_1_pBSK_SK	580
			pOP-EAP03371_EST_C_1_pBSK_SK	339
			pOP-EAP03373_EST_C_1_pBSK_SK	435
			pOP-EAP03374_EST_C_1_pBSK_SK	303
			pOP-EAP03378_EST_C_1_pBSK_SK	304
			pOP-EAP03387_EST_C_1_pBSK_SK	569
			pOP-EAP03392_EST_C_1_pBSK_SK	327
			pOP-EAP03395_EST_C_1_pBSK_SK	290
			pOP-EAP03412_EST_C_1_pBSK_SK	552
			pOP-EAP03413_EST_C_1_pBSK_SK	374
			pOP-EAP03414_EST_C_1_pBSK_SK	391
			pOP-EAP03417_EST_C_1_pBSK_SK	360
			pOP-EAP03430_EST_C_1_pBSK_SK	225
			pOP-EAP03432_EST_C_1_pBSK_SK	223
			pOP-EAP03433_EST_C_1_pBSK_SK	313
			pOP-EAP03437_EST_C_1_pBSK_SK	351
			pOP-EAP03438_EST_C_1_pBSK_SK	389
			pOP-EAP03439_EST_C_1_pBSK_SK	334
			pOP-EAP03444_EST_C_1_pBSK_SK	401
			pOP-EAP03445_EST_C_1_pBSK_SK	318
			pOP-EAP03446_EST_C_1_pBSK_SK	357
			pOP-EAP03449_EST_C_1_pBSK_SK	177
			pOP-EAP03450_EST_C_1_pBSK_SK	180
			pOP-EAP03451_EST_C_1_pBSK_SK	606
			pOP-EAP03452_EST_C_1_pBSK_SK	168
			pOP-EAP03457_EST_C_1_pBSK_SK	282
			pOP-EAP03468_EST_C_1_pBSK_SK	529
			pOP-EAP03471_EST_C_1_pBSK_SK	361
			pOP-EAP03477_EST_C_1_pBSK_SK	501
			pOP-EAP03478_EST_C_1_pBSK_SK	462
			pOP-EAP03483_EST_C_1_pBSK_SK	224
			pOP-EAP03485_EST_C_1_pBSK_SK	250
			pOP-EAP03558_EST_C_1_pBSK_SK	236
			pOP-EAP03568_EST_C_1_pBSK_SK	286
			pOP-EAP03571_EST_C_1_pBSK_SK	543
			pOP-EAP03574_EST_C_1_pBSK_SK	261
			pOP-EAP03576_EST_C_1_pBSK_SK	199
			pOP-EAP03579_EST_C_1_pBSK_SK	352
			pOP-EAP03583_EST_C_1_pBSK_SK	300
			pOP-EAP03584_EST_C_1_pBSK_SK	246
			pOP-EAP03586_EST_C_1_pBSK_SK	308
			pOP-EAP03589_EST_C_1_pBSK_SK	342
			pOP-EAP03590_EST_C_1_pBSK_SK	320
			pOP-EAP03592_EST_C_1_pBSK_SK	541
			pOP-EAP03594_EST_C_1_pBSK_SK	318
			pOP-EAP03596_EST_C_1_pBSK_SK	397
			pOP-EAP03598_EST_C_1_pBSK_SK	197
			pOP-EAP03599_EST_C_1_pBSK_SK	331
			pOP-EAP03600_EST_C_1_pBSK_SK	635
			pOP-EAP03601_EST_C_1_pBSK_SK	525
			pOP-EAP03605_EST_C_1_pBSK_SK	245
			pOP-EAP03607_EST_C_1_pBSK_SK	390
			pOP-EAP03617_EST_C_1_pBSK_SK	348
			pOP-EAP03618_EST_C_1_pBSK_SK	163
			pOP-EAP03621_EST_C_1_pBSK_SK	353
			pOP-EAP03625_EST_C_1_pBSK_SK	239

			pOP-EAP03626_EST_C_1_pBSK_SK	194
			pOP-EAP03629_EST_C_1_pBSK_SK	360
			pOP-EAP03630_EST_C_1_pBSK_SK	403
			pOP-EAP03638_EST_C_1_pBSK_SK	297
			pOP-EAP03651_EST_C_1_pBSK_SK	367
			pOP-EAP03655_EST_C_1_pBSK_SK	444
			pOP-EAP03662_EST_C_1_pBSK_SK	479
			pOP-EAP03666_EST_C_1_pBSK_SK	529
			pOP-EAP03682_EST_C_1_pBSK_SK	490
			pOP-EAP03688_EST_C_1_pBSK_SK	491
			pOP-EAP03693_EST_C_1_pBSK_SK	605
			pOP-EAP03694_EST_C_1_pBSK_SK	337
			pOP-EAP03697_EST_C_1_pBSK_SK	272
			pOP-EAP03699_EST_C_1_pBSK_SK	562
			pOP-EAP03700_EST_C_1_pBSK_SK	292
			pOP-EAP03701_EST_C_1_pBSK_SK	320
			pOP-EAP03709_EST_C_1_pBSK_SK	315
			pOP-EAP03710_EST_C_1_pBSK_SK	200
			pOP-EAP03718_EST_C_1_pBSK_SK	183
			pOP-EAP03723_EST_C_1_pBSK_SK	325
			pOP-EAP03725_EST_C_1_pBSK_SK	406
			pOP-EAP03728_EST_C_1_pBSK_SK	385
			pOP-EAP03730_EST_C_1_pBSK_SK	501
			pOP-EAP03739_EST_C_1_pBSK_SK	438
			pOP-EAP03740_EST_C_1_pBSK_SK	391
			pOP-EAP03741_EST_C_1_pBSK_SK	490
			pOP-EAP03742_EST_C_1_pBSK_SK	410
			pOP-EAP03744_EST_C_1_pBSK_SK	493
			pOP-EAP03746_EST_C_1_pBSK_SK	418
			pOP-EAP03751_EST_C_1_pBSK_SK	493
			pOP-EAP03752_EST_C_1_pBSK_SK	405
			pOP-EAP03755_EST_C_1_pBSK_SK	509
			pOP-EAP03757_EST_C_1_pBSK_SK	398
			pOP-EAP03758_EST_C_1_pBSK_SK	341
			pOP-EAP03759_EST_C_1_pBSK_SK	355
			pOP-EAP03762_EST_C_1_pBSK_SK	349
			pOP-EAP03763_EST_C_1_pBSK_SK	472
			pOP-EAP03765_EST_C_1_pBSK_SK	500
			pOP-EAP03767_EST_C_1_pBSK_SK	420
			pOP-EAP03768_EST_C_1_pBSK_SK	387
			pOP-EAP03770_EST_C_1_pBSK_SK	536
			pOP-EAP03772_EST_C_1_pBSK_SK	637
			pOP-EAP03775_EST_C_1_pBSK_SK	362
			pOP-EAP03780_EST_C_1_pBSK_SK	276
			pOP-EAP03783_EST_C_1_pBSK_SK	470
			pOP-EAP03784_EST_C_1_pBSK_SK	505
			pOP-EAP03787_EST_C_1_pBSK_SK	409
			pOP-EAP03789_EST_C_1_pBSK_SK	610
			pOP-EAP03791_EST_C_1_pBSK_SK	314
			pOP-EAP03792_EST_C_1_pBSK_SK	263
			pOP-EAP03793_EST_C_1_pBSK_SK	448
			pOP-EAP03794_EST_C_1_pBSK_SK	462
			pOP-EAP03795_EST_C_1_pBSK_SK	301
			pOP-EAP03796_EST_C_1_pBSK_SK	587
			pOP-EAP03797_EST_C_1_pBSK_SK	231
			pOP-EAP03800_EST_C_1_pBSK_SK	438

			pOP-EAP03801_EST_C_1_pBSK_SK	499
			pOP-EAP03804_EST_C_1_pBSK_SK	647
			pOP-EAP03809_EST_C_1_pBSK_SK	251
			pOP-EAP03813_EST_C_1_pBSK_SK	531
			pOP-EAP03814_EST_C_1_pBSK_SK	639
			pOP-EAP03815_EST_C_1_pBSK_SK	676
			pOP-EAP03817_EST_C_1_pBSK_SK	493
			pOP-EAP03819_EST_C_1_pBSK_SK	427
			pOP-EAP03827_EST_C_1_pBSK_SK	254
			pOP-EAP03836_EST_C_1_pBSK_SK	277
			pOP-EAP03837_EST_C_1_pBSK_SK	544
			pOP-EAP03840_EST_C_1_pBSK_SK	614
			pOP-EAP03845_EST_C_1_pBSK_SK	400
			pOP-EAP03846_EST_C_1_pBSK_SK	609
			pOP-EAP03851_EST_C_1_pBSK_SK	671
			pOP-EAP03854_EST_C_1_pBSK_SK	418
			pOP-EAP03856_EST_C_1_pBSK_SK	397
			pOP-EAP03859_EST_C_1_pBSK_SK	376
			pOP-EAP03864_EST_C_1_pBSK_SK	327
			pOP-EAP03865_EST_C_1_pBSK_SK	462
			pOP-EAP03866_EST_C_1_pBSK_SK	707
			pOP-EAP03867_EST_C_1_pBSK_SK	656
			pOP-EAP03870_EST_C_1_pBSK_SK	664
			pOP-EAP03871_EST_C_1_pBSK_SK	514
			pOP-EAP03874_EST_C_1_pBSK_SK	419
			pOP-EAP03875_EST_C_1_pBSK_SK	313
			pOP-EAP05001_EST_C_1_pBSK_SK	410
			pOP-EAP05002_EST_C_1_pBSK_SK	500
			pOP-EAP05003_EST_C_1_pBSK_SK	122
			pOP-EAP05004_EST_C_1_pBSK_SK	362
			pOP-EAP05006_EST_C_1_pBSK_SK	356
			pOP-EAP05008_EST_C_1_pBSK_SK	622
			pOP-EAP05010_EST_C_1_pBSK_SK	344
			pOP-EAP05021_EST_C_1_pBSK_SK	549
			pOP-EAP05022_EST_C_1_pBSK_SK	492
			pOP-EAP05023_EST_C_1_pBSK_SK	625
			pOP-EAP05027_EST_C_1_pBSK_SK	634
			pOP-EAP05028_EST_C_1_pBSK_SK	565
			pOP-EAP05032_EST_C_1_pBSK_SK	654
			pOP-EAP05036_EST_C_1_pBSK_SK	578
			pOP-EAP05038_EST_C_1_pBSK_SK	679
			pOP-EAP05041_EST_C_1_pBSK_SK	618
			pOP-EAP05042_EST_C_1_pBSK_SK	376
			pOP-EAP05043_EST_C_1_pBSK_SK	642
			pOP-EAP05045_EST_C_1_pBSK_SK	606
			pOP-EAP05049_EST_C_1_pBSK_SK	322
			pOP-EAP05052_EST_C_1_pBSK_SK	430
			pOP-EBP03083_EST_C_1_pBSK_M13F	191
			pOP-EBP03086_EST_C_1_pBSK_M13F	186
			pOP-EBP03090_EST_C_1_pBSK_M13F	243
			pOP-EBP03093_EST_C_1_pBSK_M13F	149
			pOP-EBP03104_EST_C_1_pBSK_M13F	219
			pOP-EBP03117_EST_C_1_pBSK_M13F	166
			pOP-EBP03123_EST_C_1_pBSK_M13F	117
			pOP-EN00102_EST_C_1_pSK_SK	510
			pOP-EN00103_EST_C_1_pSK_SK	516

			pOP-EN00104_EST_C_1_pSK_SK	579
			pOP-EN00106_EST_C_1_pSK_SK	500
			pOP-EN00107_EST_C_1_pSK_SK	516
			pOP-EN00109_EST_C_1_pSK_SK	404
			pOP-EN00110_EST_C_1_pSK_SK	533
			pOP-EN00112_EST_C_1_pSK_SK	671
			pOP-EN00113_EST_C_1_pSK_SK	510
			pOP-EN00117_EST_C_1_pSK_SK	570
			pOP-EN00118_EST_C_1_pSK_SK	515
			pOP-EN00119_EST_C_1_pSK_SK	473
			pOP-EN00121_EST_C_1_pSK_SK	541
			pOP-EN00123_EST_C_1_pSK_SK	505
			pOP-EN00125_EST_C_1_pSK_SK	594
			pOP-EN00129_EST_C_1_pSK_SK	595
			pOP-EN00130_EST_C_1_pSK_SK	518
			pOP-EN00134_EST_C_1_pSK_SK	162
			pOP-EN00135_EST_C_1_pSK_SK	586
			pOP-EN00136_EST_C_1_pSK_SK	518
			pOP-EN00138_EST_C_1_pSK_SK	499
			pOP-EN00140_EST_C_1_pSK_SK	516
			pOP-EN00141_EST_C_1_pSK_SK	510
			pOP-EN00143_EST_C_1_pSK_SK	567
			pOP-EN00144_EST_C_1_pSK_SK	623
			pOP-EN00146_EST_C_1_pSK_SK	506
			pOP-EN00147_EST_C_1_pSK_SK	523
			pOP-EN00148_EST_C_1_pSK_SK	516
			pOP-EN00149_EST_C_1_pSK_SK	595
			pOP-EN00151_EST_C_1_pSK_SK	624
			pOP-EN00152_EST_C_1_pSK_SK	578
			pOP-EN00154_EST_C_1_pSK_SK	399
			pOP-EN00155_EST_C_1_pSK_SK	505
			pOP-EN00156_EST_C_1_pSK_SK	581
			pOP-EN00157_EST_C_1_pSK_SK	510
			pOP-EN00159_EST_C_1_pSK_SK	362
			pOP-EN00162_EST_C_1_pSK_SK	497
			pOP-EN00164_EST_C_1_pSK_SK	433
			pOP-EN00165_EST_C_1_pSK_SK	511
			pOP-EN00166_EST_C_1_pSK_SK	502
			pOP-EN00169_EST_C_1_pSK_SK	500
			pOP-EN00170_EST_C_1_pSK_SK	369
			pOP-EN00171_EST_C_1_pSK_SK	390
			pOP-EN00173_EST_C_1_pSK_SK	433
			pOP-EN00174_EST_C_1_pSK_SK	449
			pOP-EN00177_EST_C_1_pSK_SK	318
			pOP-EN00178_EST_C_1_pSK_SK	447
			pOP-EN00182_EST_C_1_pSK_SK	455
			pOP-EN00185_EST_C_1_pSK_SK	443
			pOP-EN00186_EST_C_1_pSK_SK	245
			pOP-EN00187_EST_C_1_pSK_SK	462
			pOP-EN00189_EST_C_1_pSK_SK	519
			pOP-EN00191_EST_C_1_pSK_SK	402
			pOP-EN00193_EST_C_1_pSK_SK	460
			pOP-EN00194_EST_C_1_pSK_SK	398
			pOP-EN00196_EST_C_1_pSK_SK	454
			pOP-EN00198_EST_C_1_pSK_SK	393
			pOP-EN00199_EST_C_1_pSK_SK	384

			pOP-EN00200_EST_C_1_pSK_SK	394	
			pOP-EN00202_EST_C_1_pSK_SK	421	
			pOP-EN00203_EST_C_1_pSK_SK	379	
			pOP-EN00204_EST_C_1_pSK_SK	521	
			pOP-EN00205_EST_C_1_pSK_SK	456	
			pOP-EN00206_EST_C_1_pSK_SK	454	
			pOP-EN00207_EST_C_1_pSK_SK	483	
			pOP-EN00208_EST_C_1_pSK_SK	432	
			pOP-EN00209_EST_C_1_pSK_SK	452	
			pOP-EN00212_EST_C_1_pSK_SK	440	
			pOP-EN00213_EST_C_1_pSK_SK	457	
			pOP-EN00214_EST_C_1_pSK_SK	450	
			pOP-EN00217_EST_C_1_pSK_SK	521	
			pOP-EN00219_EST_C_1_pSK_SK	294	
			pOP-EN00220_EST_C_1_pSK_SK	518	
			pOP-EN00221_EST_C_1_pSK_SK	509	
			pOP-EN00222_EST_C_1_pSK_SK	514	
			pOP-EN00225_EST_C_1_pSK_SK	438	
			pOP-EN00229_EST_C_1_pSK_SK	506	
			pOP-EN00230_EST_C_1_pSK_SK	516	
			pOP-EN00232_EST_C_1_pSK_SK	442	
			pOP-EN00233_EST_C_1_pSK_SK	517	
			pOP-EN00234_EST_C_1_pSK_SK	414	
			pOP-EN00235_EST_C_1_pSK_SK	444	
			pOP-EN00238_EST_C_1_pSK_SK	411	
			pOP-EN00240_EST_C_1_pSK_SK	560	
			pOP-EN00243_EST_C_1_pSK_SK	562	
			pOP-EN00245_EST_C_1_pSK_SK	567	
			pOP-EN00246_EST_C_1_pSK_SK	563	
			pOP-EN00248_EST_C_1_pSK_SK	495	
			pOP-EN00253_EST_C_1_pSK_SK	537	
			pOP-EN00256_EST_C_1_pSK_SK	476	
			pOP-EN00258_EST_C_1_pSK_SK	382	
			pOP-EN00259_EST_C_1_pSK_SK	568	
			pOP-EN00260_EST_C_1_pSK_SK	566	
			pOP-EN00262_EST_C_1_pSK_SK	558	
			pOP-EN00263_EST_C_1_pSK_SK	547	
			pOP-EN00268_EST_C_1_pSK_SK	542	
			pOP-EN00269_EST_C_1_pSK_SK	561	
			pOP-EN00273_EST_C_1_pSK_SK	565	
			pOP-EN00274_EST_C_1_pSK_SK	563	
			pOP-EN00275_EST_C_1_pSK_SK	557	
			pOP-EN00278_EST_C_1_pSK_SK	513	
			pOP-EN00281_EST_C_1_pSK_SK	567	
			pOP-EN00283_EST_C_1_pSK_SK	543	
			pOP-EN00288_EST_C_1_pSK_SK	567	
			pOP-EN00292_EST_C_1_pSK_SK	462	
			pOP-EN00293_EST_C_1_pSK_SK	502	
			pOP-EN00295_EST_C_1_pSK_SK	560	
			pOP-EN00296_EST_C_1_pSK_SK	556	
			pOP-EN00302_EST_C_1_pSK_SK	546	
			pOP-EN00303_EST_C_1_pSK_SK	567	
			pOP-EN00305_EST_C_1_pSK_SK	566	
			pOP-EN00307_EST_C_1_pSK_SK	523	
			pOP-EN00309_EST_C_1_pSK_SK	564	
			pOP-EN00313_EST_C_1_pSK_SK	539	

			pOP-EN00318_EST_C_1_pSK_SK	559
			pOP-EN00320_EST_C_1_pSK_SK	553
			pOP-EN00323_EST_C_1_pSK_SK	550
			pOP-EN00325_EST_C_1_pSK_SK	400
			pOP-EN00326_EST_C_1_pSK_SK	473
			pOP-EN00330_EST_C_1_pSK_SK	458
			pOP-EN00331_EST_C_1_pSK_SK	490
			pOP-EN00336_EST_C_1_pSK_SK	546
			pOP-EN00337_EST_C_1_pSK_SK	546
			pOP-EN00340_EST_C_1_pSK_SK	547
			pOP-EN00346_EST_C_1_pSK_SK	536
			pOP-EN00347_EST_C_1_pSK_SK	535
			pOP-EN00349_EST_C_1_pSK_SK	554
			pOP-EN00352_EST_C_1_pSK_SK	560
			pOP-EN00353_EST_C_1_pSK_SK	477
			pOP-EN00354_EST_C_1_pSK_SK	534
			pOP-EN00359_EST_C_1_pSK_SK	511
			pOP-EN00360_EST_C_1_pSK_SK	554
			pOP-EN00363_EST_C_1_pSK_SK	539
			pOP-EN00364_EST_C_1_pSK_SK	546
			pOP-EN00366_EST_C_1_pSK_SK	550
			pOP-EN00371_EST_C_1_pSK_SK	524
			pOP-EN00373_EST_C_1_pSK_SK	550
			pOP-EN00374_EST_C_1_pSK_SK	534
			pOP-EN00376_EST_C_1_pSK_SK	557
			pOP-EN00378_EST_C_1_pSK_SK	473
			pOP-EN00380_EST_C_1_pSK_SK	557
			pOP-EN00381_EST_C_1_pSK_SK	505
			pOP-EN00382_EST_C_1_pSK_SK	539
			pOP-EN00386_EST_C_1_pSK_SK	557
			pOP-EN00395_EST_C_1_pSK_SK	529
			pOP-EN00396_EST_C_1_pSK_SK	547
			pOP-EN00398_EST_C_1_pSK_SK	563
			pOP-EN00400_EST_C_1_pSK_SK	534
			pOP-EN00402_EST_C_1_pSK_SK	556
			pOP-EN00403_EST_C_1_pSK_SK	529
			pOP-EN00405_EST_C_1_pSK_SK	539
			pOP-EN00406_EST_C_1_pSK_SK	433
			pOP-EN00409_EST_C_1_pSK_SK	534
			pOP-EN00419_EST_C_1_pSK_SK	384
			pOP-EN00424_EST_C_1_pSK_SK	547
			pOP-EN00428_EST_C_1_pSK_SK	431
			pOP-EN00432_EST_C_1_pSK_SK	512
			pOP-EN00438_EST_C_1_pSK_SK	515
			pOP-EN00439_EST_C_1_pSK_SK	511
			pOP-EN00440_EST_C_1_pSK_SK	510
			pOP-EN00445_EST_C_1_pSK_SK	501
			pOP-EN00448_EST_C_1_pSK_SK	346
			pOP-EN00449_EST_C_1_pSK_SK	507
			pOP-EN00450_EST_C_1_pSK_SK	517
			pOP-EN00453_EST_C_1_pSK_SK	516
			pOP-EN00454_EST_C_1_pSK_SK	439
			pOP-EN00456_EST_C_1_pSK_SK	512
			pOP-EN00457_EST_C_1_pSK_SK	515
			pOP-EN00458_EST_C_1_pSK_SK	515
			pOP-EN00459_EST_C_1_pSK_SK	513

			pOP-EN00460_EST_C_1_pSK_SK	517
			pOP-EN00461_EST_C_1_pSK_SK	494
			pOP-EN00462_EST_C_1_pSK_SK	501
			pOP-EN00463_EST_C_1_pSK_SK	511
			pOP-EN00469_EST_C_1_pSK_SK	325
			pOP-EN00470_EST_C_1_pSK_SK	465
			pOP-EN00471_EST_C_1_pSK_SK	515
			pOP-EN00472_EST_C_1_pSK_SK	510
			pOP-EN00475_EST_C_1_pSK_SK	509
			pOP-EN00477_EST_C_1_pSK_SK	515
			pOP-EN00478_EST_C_1_pSK_SK	514
			pOP-EN00479_EST_C_1_pSK_SK	510
			pOP-EN00483_EST_C_1_pSK_SK	510
			pOP-EN00484_EST_C_1_pSK_SK	513
			pOP-EN00485_EST_C_1_pSK_SK	462
			pOP-EN00486_EST_C_1_pSK_SK	463
			pOP-EN00487_EST_C_1_pSK_SK	521
			pOP-EN00490_EST_C_1_pSK_SK	511
			pOP-EN00491_EST_C_1_pSK_SK	519
			pOP-EN00492_EST_C_1_pSK_SK	515
			pOP-EN00496_EST_C_1_pSK_SK	482
			pOP-EN00498_EST_C_1_pSK_SK	517
			pOP-EN00500_EST_C_1_pSK_SK	430
			pOP-EN00501_EST_C_1_pSK_SK	509
			pOP-EN00504_EST_C_1_pSK_SK	514
			pOP-EN00507_EST_C_1_pSK_SK	494
			pOP-EN00510_EST_C_1_pSK_SK	510
			pOP-EN00511_EST_C_1_pSK_SK	498
			pOP-EN00512_EST_C_1_pSK_SK	508
			pOP-EN00513_EST_C_1_pSK_SK	489
			pOP-EN00514_EST_C_1_pSK_SK	509
			pOP-EN00515_EST_C_1_pSK_SK	495
			pOP-EN00520_EST_C_1_pSK_SK	463
			pOP-EN00522_EST_C_1_pSK_SK	415
			pOP-EN00523_EST_C_1_pSK_SK	510
			pOP-EN00527_EST_C_1_pSK_SK	420
			pOP-EN00534_EST_C_1_pSK_SK	456
			pOP-EN00537_EST_C_1_pSK_SK	459
			pOP-EN00538_EST_C_1_pSK_SK	624
			pOP-EN00542_EST_C_1_pSK_SK	611
			pOP-EN00544_EST_C_1_pSK_SK	476
			pOP-EN00545_EST_C_1_pSK_SK	543
			pOP-EN00546_EST_C_1_pSK_SK	594
			pOP-EN00561_EST_C_1_pSK_SK	537
			pOP-EN00563_EST_C_1_pSK_SK	370
			pOP-EN00565_EST_C_1_pSK_SK	410
			pOP-EN00566_EST_C_1_pSK_SK	534
			pOP-EN00567_EST_C_1_pSK_SK	623
			pOP-EN00572_EST_C_1_pSK_SK	518
			pOP-EN00573_EST_C_1_pSK_SK	338
			pOP-EN00574_EST_C_1_pSK_SK	462
			pOP-EN00577_EST_C_1_pSK_SK	560
			pOP-EN00579_EST_C_1_pSK_SK	472
			pOP-EN00580_EST_C_1_pSK_SK	234
			pOP-EN00582_EST_C_1_pSK_SK	616
			pOP-EN00586_EST_C_1_pSK_SK	622

			pOP-EN00587_EST_C_1_pSK_SK	620
			pOP-EN00589_EST_C_1_pSK_SK	529
			pOP-EN00596_EST_C_1_pSK_SK	490
			pOP-EN00598_EST_C_1_pSK_SK	509
			pOP-EN00604_EST_C_1_pSK_SK	440
			pOP-EN00605_EST_C_1_pSK_SK	561
			pOP-EN00606_EST_C_1_pSK_SK	416
			pOP-EN00610_EST_C_1_pSK_SK	481
			pOP-EN00617_EST_C_1_pSK_SK	602
			pOP-EN00618_EST_C_1_pSK_SK	484
			pOP-EN00619_EST_C_1_pSK_SK	453
			pOP-EN00620_EST_C_1_pSK_SK	420
			pOP-EN00621_EST_C_1_pSK_SK	474
			pOP-EN00623_EST_C_1_pSK_SK	452
			pOP-EN00626_EST_C_1_pSK_SK	419
			pOP-EN00627_EST_C_1_pSK_SK	407
			pOP-EN00629_EST_C_1_pSK_SK	395
			pOP-EN00630_EST_C_1_pSK_SK	406
			pOP-EN00632_EST_C_1_pSK_SK	413
			pOP-EN00633_EST_C_1_pSK_SK	467
			pOP-EN00638_EST_C_1_pSK_SK	543
			pOP-EN00639_EST_C_1_pSK_SK	421
			pOP-EN00641_EST_C_1_pSK_SK	406
			pOP-EN00652_EST_C_1_pSK_SK	544
			pOP-EN00655_EST_C_1_pSK_SK	527
			pOP-EN00659_EST_C_1_pSK_SK	553
			pOP-EN00665_EST_C_1_pSK_SK	548
			pOP-EN00672_EST_C_1_pSK_SK	406
			pOP-EN00673_EST_C_1_pSK_SK	365
			pOP-EN00675_EST_C_1_pSK_SK	432
			pOP-EN00676_EST_C_1_pSK_SK	509
			pOP-EN00678_EST_C_1_pSK_SK	514
			pOP-EN00679_EST_C_1_pSK_SK	501
			pOP-EN00682_EST_C_1_pSK_SK	444
			pOP-EN00684_EST_C_1_pSK_SK	533
			pOP-EN00685_EST_C_1_pSK_SK	505
			pOP-EN00688_EST_C_1_pSK_SK	519
			pOP-EN00694_EST_C_1_pSK_SK	534
			pOP-EN00695_EST_C_1_pSK_SK	501
			pOP-EN00696_EST_C_1_pSK_SK	450
			pOP-EN00698_EST_C_1_pSK_SK	482
			pOP-EN00700_EST_C_1_pSK_SK	446
			pOP-EN00701_EST_C_1_pSK_SK	533
			pOP-EN00702_EST_C_1_pSK_SK	414
			pOP-EN00703_EST_C_1_pSK_SK	420
			pOP-EN00706_EST_C_1_pSK_SK	529
			pOP-EN00707_EST_C_1_pSK_SK	463
			pOP-EN00709_EST_C_1_pSK_SK	497
			pOP-EN00712_EST_C_1_pSK_SK	490
			pOP-EN00714_EST_C_1_pSK_SK	476
			pOP-EN00715_EST_C_1_pSK_SK	286
			pOP-EN00718_EST_C_1_pSK_SK	534
			pOP-EN00720_EST_C_1_pSK_SK	406
			pOP-EN00721_EST_C_1_pSK_SK	403
			pOP-EN00735_EST_C_1_pSK_SK	482
			pOP-EN00736_EST_C_1_pSK_SK	542

			pOP-EN00753_EST_C_1_pSK_SK	575
			pOP-EN00759_EST_C_1_pSK_SK	530
			pOP-EN00763_EST_C_1_pSK_SK	487
			pOP-EN00767_EST_C_1_pSK_SK	433
			pOP-EN00770_EST_C_1_pSK_SK	563
			pOP-EN00781_EST_C_1_pSK_SK	572
			pOP-EN00783_EST_C_1_pSK_SK	573
			pOP-EN00790_EST_C_1_pSK_SK	557
			pOP-EN00796_EST_C_1_pSK_SK	335
			pOP-EN00797_EST_C_1_pSK_SK	266
			pOP-EN00798_EST_C_1_pSK_SK	603
			pOP-EN00800_EST_C_1_pSK_SK	442
			pOP-EN00801_EST_C_1_pSK_SK	605
			pOP-EN00802_EST_C_1_pSK_SK	391
			pOP-EN00803_EST_C_1_pSK_SK	440
			pOP-EN00805_EST_C_1_pSK_SK	585
			pOP-EN00806_EST_C_1_pSK_SK	526
			pOP-EN00809_EST_C_1_pSK_SK	519
			pOP-EN00810_EST_C_1_pSK_SK	517
			pOP-EN00811_EST_C_1_pSK_SK	469
			pOP-EN00815_EST_C_1_pSK_SK	530
			pOP-EN00820_EST_C_1_pSK_SK	508
			pOP-EN00829_EST_C_1_pSK_SK	526
			pOP-EN00831_EST_C_1_pSK_SK	527
			pOP-EN00840_EST_C_1_pSK_SK	468
			pOP-EN00841_EST_C_1_pSK_SK	396
			pOP-EN00842_EST_C_1_pSK_SK	490
			pOP-EN00847_EST_C_1_pSK_SK	540
			pOP-EN00849_EST_C_1_pSK_SK	544
			pOP-EN00850_EST_C_1_pSK_SK	527
			pOP-EN00851_EST_C_1_pSK_SK	199
			pOP-EN00852_EST_C_1_pSK_SK	543
			pOP-EN00853_EST_C_1_pSK_SK	540
			pOP-EN00854_EST_C_1_pSK_SK	488
			pOP-EN00855_EST_C_1_pSK_SK	536
			pOP-EN00856_EST_C_1_pSK_SK	523
			pOP-EN00857_EST_C_1_pSK_SK	542
			pOP-EN00865_EST_C_1_pSK_SK	534
			pOP-EN00866_EST_C_1_pSK_SK	530
			pOP-EN00867_EST_C_1_pSK_SK	385
			pOP-EN00871_EST_C_1_pSK_SK	543
			pOP-EN00872_EST_C_1_pSK_SK	447
			pOP-EN00877_EST_C_1_pSK_SK	538
			pOP-EN00886_EST_C_1_pSK_SK	377
			pOP-EN00887_EST_C_1_pSK_SK	541
			pOP-EN00889_EST_C_1_pSK_SK	536
			pOP-EN00890_EST_C_1_pSK_SK	521
			pOP-EN00892_EST_C_1_pSK_SK	536
			pOP-EN00893_EST_C_1_pSK_SK	528
			pOP-EN00903_EST_C_1_pSK_SK	499
			pOP-EN00909_EST_C_1_pSK_SK	529
			pOP-ENP00001_EST_C_1_pSK_SK	650
			pOP-ENP00002_EST_C_1_pSK_SK	643
			pOP-ENP00007_EST_C_1_pSK_SK	452
			pOP-ENP00008_EST_C_1_pSK_SK	307
			pOP-ENP00010_EST_C_1_pSK_SK	208

			pOP-ENP00011_EST_C_1_pSK_SK	383
			pOP-ENP00013_EST_C_1_pSK_SK	211
			pOP-ENP00015_EST_C_1_pSK_SK	225
			pOP-ENP00017_EST_C_1_pSK_SK	341
			pOP-ENP00018_EST_C_1_pSK_SK	250
			pOP-ENP00019_EST_C_1_pSK_SK	233
			pOP-ENP00020_EST_C_1_pSK_SK	521
			pOP-EO02002_EST_C_1_pSK_SK	252
			pOP-EO02007_EST_C_1_pSK_SK	501
			pOP-EO02014_EST_C_1_pSK_SK	391
			pOP-EO02016_EST_C_1_pSK_SK	589
			pOP-EO02019_EST_C_1_pSK_SK	363
			pOP-EO02022_EST_C_1_pSK_SK	357
			pOP-EO02023_EST_C_1_pSK_SK	598
			pOP-EO02025_EST_C_1_pSK_SK	555
			pOP-EO02032_EST_C_1_pSK_SK	311
			pOP-EO02033_EST_C_1_pSK_SK	416
			pOP-EO02034_EST_C_1_pSK_SK	666
			pOP-EO02041_EST_C_1_pSK_SK	619
			pOP-EO02049_EST_C_1_pSK_SK	643
			pOP-EO02051_EST_C_1_pSK_SK	497
			pOP-EO02052_EST_C_1_pSK_SK	485
			pOP-EO02053_EST_C_1_pSK_SK	601
			pOP-EO02054_EST_C_1_pSK_SK	696
			pOP-EO02061_EST_C_1_pSK_SK	483
			pOP-EO02063_EST_C_1_pSK_SK	433
			pOP-EO02065_EST_C_1_pSK_SK	651
			pOP-EO02066_EST_C_1_pSK_SK	618
			pOP-EO02070_EST_C_1_pSK_SK	605
			pOP-EO02072_EST_C_1_pSK_SK	440
			pOP-EO02074_EST_C_1_pSK_SK	509
			pOP-EO02075_EST_C_1_pSK_SK	566
			pOP-EO02080_EST_C_1_pSK_SK	707
			pOP-EO02081_EST_C_1_pSK_SK	382
			pOP-EO02084_EST_C_1_pSK_SK	560
			pOP-EO02086_EST_C_1_pSK_SK	397
			pOP-EO02093_EST_C_1_pSK_SK	594
			pOP-EO02095_EST_C_1_pSK_SK	532
			pOP-EO02098_EST_C_1_pSK_SK	360
			pOP-EO02100_EST_C_1_pSK_SK	604
			pOP-EO02102_EST_C_1_pSK_SK	566
			pOP-EO02109_EST_C_1_pSK_SK	232
			pOP-EO02118_EST_C_1_pSK_SK	418
			pOP-EO02122_EST_C_1_pSK_SK	321
			pOP-EO02127_EST_C_1_pSK_SK	670
			pOP-EO02133_EST_C_1_pSK_SK	605
			pOP-EO02138_EST_C_1_pSK_SK	147
			pOP-EO02139_EST_C_1_pSK_SK	501
			pOP-EO02140_EST_C_1_pSK_SK	417
			pOP-EO02147_EST_C_1_pSK_SK	633
			pOP-EO02148_EST_C_1_pSK_SK	325
			pOP-EO02149_EST_C_1_pSK_SK	661
			pOP-EO02150_EST_C_1_pSK_SK	512
			pOP-EO02152_EST_C_1_pSK_SK	488
			pOP-EO02154_EST_C_1_pSK_SK	383
			pOP-EO02156_EST_C_1_pSK_SK	404

			pOP-EO02159_EST_C_1_pSK_SK	382
			pOP-EO02161_EST_C_1_pSK_SK	628
			pOP-EO02163_EST_C_1_pSK_SK	741
			pOP-EO02167_EST_C_1_pSK_SK	713
			pOP-EO02172_EST_C_1_pSK_SK	503
			pOP-EO02173_EST_C_1_pSK_SK	451
			pOP-EO02175_EST_C_1_pSK_SK	442
			pOP-EO02176_EST_C_1_pSK_SK	441
			pOP-EO02179_EST_C_1_pSK_SK	440
			pOP-EO02188_EST_C_1_pSK_SK	592
			pOP-EO02189_EST_C_1_pSK_SK	339
			pOP-EO02190_EST_C_1_pSK_SK	563
			pOP-EO02192_EST_C_1_pSK_SK	317
			pOP-EO02195_EST_C_1_pSK_SK	486
			pOP-EO02199_EST_C_1_pSK_SK	624
			pOP-EO02200_EST_C_1_pSK_SK	566
			pOP-EO02209_EST_C_1_pSK_SK	576
			pOP-EO02213_EST_C_1_pSK_SK	496
			pOP-EO02215_EST_C_1_pSK_SK	557
			pOP-EO02218_EST_C_1_pSK_SK	436
			pOP-EO02219_EST_C_1_pSK_SK	370
			pOP-EO02226_EST_C_1_pSK_SK	463
			pOP-EO02229_EST_C_1_pSK_SK	354
			pOP-EO02236_EST_C_1_pSK_SK	642
			pOP-EO02239_EST_C_1_pSK_SK	339
			pOP-EO02240_EST_C_1_pSK_SK	585
			pOP-EO02248_EST_C_1_pSK_SK	661
			pOP-EO02257_EST_C_1_pSK_SK	685
			pOP-EO02258_EST_C_1_pSK_SK	658
			pOP-EO02266_EST_C_1_pSK_SK	631
			pOP-EO02268_EST_C_1_pSK_SK	492
			pOP-EO02269_EST_C_1_pSK_SK	336
			pOP-EO02270_EST_C_1_pSK_SK	566
			pOP-EO02271_EST_C_1_pSK_SK	615
			pOP-EO02275_EST_C_1_pSK_SK	564
			pOP-EO02287_EST_C_1_pSK_SK	617
			pOP-EO02291_EST_C_1_pSK_SK	574
			pOP-EO02293_EST_C_1_pSK_SK	631
			pOP-EO02296_EST_C_1_pSK_SK	622
			pOP-EO02298_EST_C_1_pSK_SK	601
			pOP-EO02300_EST_C_1_pSK_SK	610
			pOP-EO02301_EST_C_1_pSK_SK	534
			pOP-EO02307_EST_C_1_pSK_SK	527
			pOP-EO02309_EST_C_1_pSK_SK	539
			pOP-EO02314_EST_C_1_pSK_SK	540
			pOP-EO02323_EST_C_1_pSK_SK	689
			pOP-EO02327_EST_C_1_pSK_SK	698
			pOP-EO02331_EST_C_1_pSK_SK	418
			pOP-EO02332_EST_C_1_pSK_SK	669
			pOP-EO02333_EST_C_1_pSK_SK	366
			pOP-EO02335_EST_C_1_pSK_SK	530
			pOP-EO02336_EST_C_1_pSK_SK	585
			pOP-EO02339_EST_C_1_pSK_SK	604
			pOP-EO02342_EST_C_1_pSK_SK	722
			pOP-EO02343_EST_C_1_pSK_SK	572
			pOP-EO02344_EST_C_1_pSK_SK	674

			pOP-EO02349_EST_C_1_pSK_SK	521
			pOP-EO02353_EST_C_1_pSK_SK	639
			pOP-EO02354_EST_C_1_pSK_SK	372
			pOP-EO02364_EST_C_1_pSK_SK	684
			pOP-EO02365_EST_C_1_pSK_SK	363
			pOP-EO02368_EST_C_1_pSK_SK	711
			pOP-EO02369_EST_C_1_pSK_SK	691
			pOP-EO02372_EST_C_1_pSK_SK	364
			pOP-EO02378_EST_C_1_pSK_SK	547
			pOP-EO02393_EST_C_1_pSK_SK	568
			pOP-EO02394_EST_C_1_pSK_SK	337
			pOP-EO02395_EST_C_1_pSK_SK	594
			pOP-EO02397_EST_C_1_pSK_SK	613
			pOP-EO02399_EST_C_1_pSK_SK	682
			pOP-EO02406_EST_C_1_pSK_SK	566
			pOP-EO02409_EST_C_1_pSK_SK	463
			pOP-EO02410_EST_C_1_pSK_SK	318
			pOP-EO02416_EST_C_1_pSK_SK	460
			pOP-EO02420_EST_C_1_pSK_SK	465
			pOP-EO02429_EST_C_1_pSK_SK	463
			pOP-EO02432_EST_C_1_pSK_SK	464
			pOP-EO02437_EST_C_1_pSK_SK	455
			pOP-EO02438_EST_C_1_pSK_SK	466
			pOP-EO02443_EST_C_1_pSK_SK	462
			pOP-EO02448_EST_C_1_pSK_SK	403
			pOP-EO02451_EST_C_1_pSK_SK	461
			pOP-EO02458_EST_C_1_pSK_SK	460
			pOP-EO02461_EST_C_1_pSK_SK	458
			pOP-EO02463_EST_C_1_pSK_SK	188
			pOP-EO02465_EST_C_1_pSK_SK	461
			pOP-EO02469_EST_C_1_pSK_SK	449
			pOP-EO02471_EST_C_1_pSK_SK	470
			pOP-EO02475_EST_C_1_pSK_SK	472
			pOP-EO02476_EST_C_1_pSK_SK	471
			pOP-EO02477_EST_C_1_pSK_SK	471
			pOP-EO02493_EST_C_1_pSK_SK	528
			pOP-EO02494_EST_C_1_pSK_SK	528
			pOP-EO02499_EST_C_1_pSK_SK	528
			pOP-EO02504_EST_C_1_pSK_SK	527
			pOP-EO02515_EST_C_1_pSK_SK	527
			pOP-EO02526_EST_C_1_pSK_SK	529
			pOP-EO02530_EST_C_1_pSK_SK	235
			pOP-EO02534_EST_C_1_pSK_SK	528
			pOP-EO02539_EST_C_1_pSK_SK	424
			pOP-EO02540_EST_C_1_pSK_SK	521
			pOP-EO02544_EST_C_1_pSK_SK	528
			pOP-EO02546_EST_C_1_pSK_SK	526
			pOP-EO02548_EST_C_1_pSK_SK	379
			pOP-EO02553_EST_C_1_pSK_SK	479
			pOP-EO02554_EST_C_1_pSK_SK	436
			pOP-EO02556_EST_C_1_pSK_SK	441
			pOP-EO02557_EST_C_1_pSK_SK	478
			pOP-EO02559_EST_C_1_pSK_SK	419
			pOP-EO02560_EST_C_1_pSK_SK	466
			pOP-EO02562_EST_C_1_pSK_SK	463
			pOP-EO02563_EST_C_1_pSK_SK	478

			pOP-EO02564_EST_C_1_pSK_SK	480
			pOP-EO02565_EST_C_1_pSK_SK	478
			pOP-EO02566_EST_C_1_pSK_SK	423
			pOP-EO02569_EST_C_1_pSK_SK	469
			pOP-EO02575_EST_C_1_pSK_SK	346
			pOP-EO02582_EST_C_1_pSK_SK	415
			pOP-EO02584_EST_C_1_pSK_SK	470
			pOP-EO02586_EST_C_1_pSK_SK	472
			pOP-EO02591_EST_C_1_pSK_SK	466
			pOP-EO02592_EST_C_1_pSK_SK	473
			pOP-EO02593_EST_C_1_pSK_SK	374
			pOP-EO02597_EST_C_1_pSK_SK	372
			pOP-EO02598_EST_C_1_pSK_SK	373
			pOP-EO02603_EST_C_1_pSK_SK	372
			pOP-EO02607_EST_C_1_pSK_SK	387
			pOP-EO02612_EST_C_1_pSK_SK	375
			pOP-EO02613_EST_C_1_pSK_SK	375
			pOP-EO02620_EST_C_1_pSK_SK	373
			pOP-EO02621_EST_C_1_pSK_SK	375
			pOP-EO02622_EST_C_1_pSK_SK	372
			pOP-EO02623_EST_C_1_pSK_SK	377
			pOP-EO02624_EST_C_1_pSK_SK	374
			pOP-EO02625_EST_C_1_pSK_SK	346
			pOP-EO02627_EST_C_1_pSK_SK	386
			pOP-EO02631_EST_C_1_pSK_SK	457
			pOP-EO02637_EST_C_1_pSK_SK	463
			pOP-EO02644_EST_C_1_pSK_SK	333
			pOP-EO02645_EST_C_1_pSK_SK	453
			pOP-EO02646_EST_C_1_pSK_SK	458
			pOP-EO02649_EST_C_1_pSK_SK	461
			pOP-EO02650_EST_C_1_pSK_SK	461
			pOP-EO02651_EST_C_1_pSK_SK	452
			pOP-EO02652_EST_C_1_pSK_SK	460
			pOP-EO02653_EST_C_1_pSK_SK	473
			pOP-EO02657_EST_C_1_pSK_SK	460
			pOP-EO02658_EST_C_1_pSK_SK	362
			pOP-EO02665_EST_C_1_pSK_SK	455
			pOP-EO02667_EST_C_1_pSK_SK	276
			pOP-EO02670_EST_C_1_pSK_SK	472
			pOP-EO02673_EST_C_1_pSK_SK	369
			pOP-EO02680_EST_C_1_pSK_SK	445
			pOP-EO02681_EST_C_1_pSK_SK	387
			pOP-EO02682_EST_C_1_pSK_SK	435
			pOP-EO02683_EST_C_1_pSK_SK	349
			pOP-EO02686_EST_C_1_pSK_SK	445
			pOP-EO02688_EST_C_1_pSK_SK	401
			pOP-EO02690_EST_C_1_pSK_SK	386
			pOP-EO02691_EST_C_1_pSK_SK	420
			pOP-EO02694_EST_C_1_pSK_SK	449
			pOP-EO02696_EST_C_1_pSK_SK	443
			pOP-EO02703_EST_C_1_pSK_SK	463
			pOP-EO02708_EST_C_1_pSK_SK	397
			pOP-EO02709_EST_C_1_pSK_SK	448
			pOP-EO02712_EST_C_1_pSK_SK	446
			pOP-EO02716_EST_C_1_pSK_SK	469
			pOP-EO02717_EST_C_1_pSK_SK	433

			pOP-EO02718_EST_C_1_pSK_SK	444
			pOP-EO02719_EST_C_1_pSK_SK	448
			pOP-EO02723_EST_C_1_pSK_SK	424
			pOP-EO02729_EST_C_1_pSK_SK	195
			pOP-EO02730_EST_C_1_pSK_SK	183
			pOP-EO02731_EST_C_1_pSK_SK	451
			pOP-EO02737_EST_C_1_pSK_SK	450
			pOP-EO02743_EST_C_1_pSK_SK	447
			pOP-EO02745_EST_C_1_pSK_SK	293
			pOP-EO02747_EST_C_1_pSK_SK	460
			pOP-EO02748_EST_C_1_pSK_SK	414
			pOP-EO02751_EST_C_1_pSK_SK	444
			pOP-EO02754_EST_C_1_pSK_SK	445
			pOP-EO02759_EST_C_1_pSK_SK	436
			pOP-EO02763_EST_C_1_pSK_SK	410
			pOP-EO02767_EST_C_1_pSK_SK	196
			pOP-EO02768_EST_C_1_pSK_SK	437
			pOP-EO02771_EST_C_1_pSK_SK	446
			pOP-EO02779_EST_C_1_pSK_SK	125
			pOP-EO02783_EST_C_1_pSK_SK	422
			pOP-EO02784_EST_C_1_pSK_SK	450
			pOP-EO02787_EST_C_1_pSK_SK	420
			pOP-EO02791_EST_C_1_pSK_SK	465
			pOP-EO02793_EST_C_1_pSK_SK	133
			pOP-EO02795_EST_C_1_pSK_SK	361
			pOP-EO02799_EST_C_1_pSK_SK	405
			pOP-EO02801_EST_C_1_pSK_SK	414
			pOP-EO02814_EST_C_1_pSK_SK	329
			pOP-EO02815_EST_C_1_pSK_SK	436
			pOP-EO02816_EST_C_1_pSK_SK	391
			pOP-EO02818_EST_C_1_pSK_SK	418
			pOP-EO02819_EST_C_1_pSK_SK	330
			pOP-EO02821_EST_C_1_pSK_SK	338
			pOP-EO02823_EST_C_1_pSK_SK	441
			pOP-EO02827_EST_C_1_pSK_SK	318
			pOP-EO02833_EST_C_1_pSK_SK	347
			pOP-EO02834_EST_C_1_pSK_SK	454
			pOP-EO02835_EST_C_1_pSK_SK	338
			pOP-EO02837_EST_C_1_pSK_SK	458
			pOP-EO02839_EST_C_1_pSK_SK	467
			pOP-EO02840_EST_C_1_pSK_SK	308
			pOP-EO02843_EST_C_1_pSK_SK	405
			pOP-EO02846_EST_C_1_pSK_SK	235
			pOP-EO02848_EST_C_1_pSK_SK	455
			pOP-EO02850_EST_C_1_pSK_SK	345
			pOP-EO02853_EST_C_1_pSK_SK	319
			pOP-EO02858_EST_C_1_pSK_SK	347
			pOP-EO02859_EST_C_1_pSK_SK	177
			pOP-EO02860_EST_C_1_pSK_SK	391
			pOP-EO02861_EST_C_1_pSK_SK	308
			pOP-EO02863_EST_C_1_pSK_SK	308
			pOP-EO02880_EST_C_1_pSK_SK	415
			pOP-EO02883_EST_C_1_pSK_SK	344
			pOP-EO02888_EST_C_1_pSK_SK	440
			pOP-EO02895_EST_C_1_pSK_SK	256
			pOP-EO02898_EST_C_1_pSK_SK	456

			pOP-EO02901_EST_C_1_pSK_SK	361
			pOP-EO02904_EST_C_1_pSK_SK	442
			pOP-EO02910_EST_C_1_pSK_SK	263
			pOP-EO02911_EST_C_1_pSK_SK	447
			pOP-EO02912_EST_C_1_pSK_SK	330
			pOP-EO02914_EST_C_1_pSK_SK	415
			pOP-EO02921_EST_C_1_pSK_SK	188
			pOP-EO02922_EST_C_1_pSK_SK	448
			pOP-EO02924_EST_C_1_pSK_SK	263
			pOP-EO02926_EST_C_1_pSK_SK	439
			pOP-EO02929_EST_C_1_pSK_SK	447
			pOP-EO02930_EST_C_1_pSK_SK	448
			pOP-EO02939_EST_C_1_pSK_SK	456
			pOP-EO02942_EST_C_1_pSK_SK	436
			pOP-EO02943_EST_C_1_pSK_SK	441
			pOP-EO02946_EST_C_1_pSK_SK	258
			pOP-EO02949_EST_C_1_pSK_SK	426
			pOP-EO02952_EST_C_1_pSK_SK	216
			pOP-EO02953_EST_C_1_pSK_SK	302
			pOP-EO02954_EST_C_1_pSK_SK	428
			pOP-EO02955_EST_C_1_pSK_SK	445
			pOP-EO02960_EST_C_1_pSK_SK	304
			pOP-EO02961_EST_C_1_pSK_SK	452
			pOP-EO02963_EST_C_1_pSK_SK	338
			pOP-EO02967_EST_C_1_pSK_SK	275
			pOP-EO02969_EST_C_1_pSK_SK	187
			pOP-EO02970_EST_C_1_pSK_SK	314
			pOP-EO02976_EST_C_1_pSK_SK	423
			pOP-EO02978_EST_C_1_pSK_SK	383
			pOP-EO02987_EST_C_1_pSK_SK	448
			pOP-EO02988_EST_C_1_pSK_SK	335
			pOP-EO02993_EST_C_1_pSK_SK	215
			pOP-EO02995_EST_C_1_pSK_SK	450
			pOP-EO02997_EST_C_1_pSK_SK	428
			pOP-EO03002_EST_C_1_pSK_SK	441
			pOP-EO03003_EST_C_1_pSK_SK	463
			pOP-EO03005_EST_C_1_pSK_SK	270
			pOP-EO03008_EST_C_1_pSK_SK	381
			pOP-EO03012_EST_C_1_pSK_SK	408
			pOP-EO03013_EST_C_1_pSK_SK	443
			pOP-EO03014_EST_C_1_pSK_SK	444
			pOP-EO03016_EST_C_1_pSK_SK	434
			pOP-EO03019_EST_C_1_pSK_SK	354
			pOP-EO03020_EST_C_1_pSK_SK	496
			pOP-EO03023_EST_C_1_pSK_SK	381
			pOP-EO03028_EST_C_1_pSK_SK	493
			pOP-EO03030_EST_C_1_pSK_SK	398
			pOP-EO03032_EST_C_1_pSK_SK	459
			pOP-EO03034_EST_C_1_pSK_SK	155
			pOP-EO03035_EST_C_1_pSK_SK	355
			pOP-EO03038_EST_C_1_pSK_SK	448
			pOP-EO03040_EST_C_1_pSK_SK	449
			pOP-EO03041_EST_C_1_pSK_SK	143
			pOP-EO03042_EST_C_1_pSK_SK	413
			pOP-EO03044_EST_C_1_pSK_SK	287
			pOP-EO03048_EST_C_1_pSK_SK	479

			pOP-EO03050_EST_C_1_pSK_SK	412
			pOP-EO03052_EST_C_1_pSK_SK	354
			pOP-EO03055_EST_C_1_pSK_SK	396
			pOP-EO03058_EST_C_1_pSK_SK	434
			pOP-EO03063_EST_C_1_pSK_SK	194
			pOP-EO03068_EST_C_1_pSK_SK	482
			pOP-EO03070_EST_C_1_pSK_SK	378
			pOP-EO03072_EST_C_1_pSK_SK	453
			pOP-EO03073_EST_C_1_pSK_SK	443
			pOP-EO03074_EST_C_1_pSK_SK	430
			pOP-EO03075_EST_C_1_pSK_SK	261
			pOP-EO03078_EST_C_1_pSK_SK	417
			pOP-EO03081_EST_C_1_pSK_SK	427
			pOP-EO03088_EST_C_1_pSK_SK	494
			pOP-EO03092_EST_C_1_pSK_SK	369
			pOP-EO03099_EST_C_1_pSK_SK	453
			pOP-EO03100_EST_C_1_pSK_SK	362
			pOP-EO03102_EST_C_1_pSK_SK	336
			pOP-EO03104_EST_C_1_pSK_SK	481
			pOP-EO03107_EST_C_1_pSK_SK	438
			pOP-EO03108_EST_C_1_pSK_SK	345
			pOP-EO03109_EST_C_1_pSK_SK	478
			pOP-EO03110_EST_C_1_pSK_SK	438
			pOP-EO03111_EST_C_1_pSK_SK	481
			pOP-EO03113_EST_C_1_pSK_SK	386
			pOP-EO03114_EST_C_1_pSK_SK	476
			pOP-EO03116_EST_C_1_pSK_SK	392
			pOP-EO03117_EST_C_1_pSK_SK	295
			pOP-EO03123_EST_C_1_pSK_SK	470
			pOP-EO03126_EST_C_1_pSK_SK	381
			pOP-EO03129_EST_C_1_pSK_SK	302
			pOP-EO03132_EST_C_1_pSK_SK	414
			pOP-EO03135_EST_C_1_pSK_SK	421
			pOP-EO03137_EST_C_1_pSK_SK	278
			pOP-EO03140_EST_C_1_pSK_SK	465
			pOP-EO03149_EST_C_1_pSK_SK	473
			pOP-EO03150_EST_C_1_pSK_SK	368
			pOP-EO03153_EST_C_1_pSK_SK	486
			pOP-EO03156_EST_C_1_pSK_SK	480
			pOP-EO03157_EST_C_1_pSK_SK	470
			pOP-EO03159_EST_C_1_pSK_SK	474
			pOP-EO03161_EST_C_1_pSK_SK	431
			pOP-EO03163_EST_C_1_pSK_SK	469
			pOP-EO03165_EST_C_1_pSK_SK	255
			pOP-EO03166_EST_C_1_pSK_SK	411
			pOP-EO03167_EST_C_1_pSK_SK	474
			pOP-EO03168_EST_C_1_pSK_SK	422
			pOP-EO03178_EST_C_1_pSK_SK	377
			pOP-EO03181_EST_C_1_pSK_SK	376
			pOP-EO03182_EST_C_1_pSK_SK	465
			pOP-EO03198_EST_C_1_pSK_SK	450
			pOP-EO03200_EST_C_1_pSK_SK	466
			pOP-EO03203_EST_C_1_pSK_SK	401
			pOP-EO03205_EST_C_1_pSK_SK	437
			pOP-EO03206_EST_C_1_pSK_SK	435
			pOP-EO03208_EST_C_1_pSK_SK	473

			pOP-EO03210_EST_C_1_pSK_SK	472
			pOP-EO03212_EST_C_1_pSK_SK	705
			pOP-EO03215_EST_C_1_pSK_SK	701
			pOP-EO03216_EST_C_1_pSK_SK	691
			pOP-EO03218_EST_C_1_pSK_SK	456
			pOP-EO03219_EST_C_1_pSK_SK	464
			pOP-EO03220_EST_C_1_pSK_SK	476
			pOP-EO03221_EST_C_1_pSK_SK	474
			pOP-EO03223_EST_C_1_pSK_SK	432
			pOP-EO03229_EST_C_1_pSK_SK	472
			pOP-EO03232_EST_C_1_pSK_SK	461
			pOP-EO03239_EST_C_1_pSK_SK	461
			pOP-EO03240_EST_C_1_pSK_SK	472
			pOP-EO03244_EST_C_1_pSK_SK	473
			pOP-EO03252_EST_C_1_pSK_SK	459
			pOP-EO03258_EST_C_1_pSK_SK	474
			pOP-EO03270_EST_C_1_pSK_SK	267
			pOP-EO03271_EST_C_1_pSK_SK	427
			pOP-EO03273_EST_C_1_pSK_SK	436
			pOP-EO03275_EST_C_1_pSK_SK	473
			pOP-EO03278_EST_C_1_pSK_SK	441
			pOP-EO03282_EST_C_1_pSK_SK	395
			pOP-EO03288_EST_C_1_pSK_SK	454
			pOP-EO03291_EST_C_1_pSK_SK	396
			pOP-EO03292_EST_C_1_pSK_SK	350
			pOP-EO03293_EST_C_1_pSK_SK	403
			pOP-EO03294_EST_C_1_pSK_SK	368
			pOP-EO03295_EST_C_1_pSK_SK	457
			pOP-EO03296_EST_C_1_pSK_SK	343
			pOP-EO03298_EST_C_1_pSK_SK	314
			pOP-EO03300_EST_C_1_pSK_SK	321
			pOP-EO03301_EST_C_1_pSK_SK	326
			pOP-EO03302_EST_C_1_pSK_SK	278
			pOP-EO03303_EST_C_1_pSK_SK	321
			pOP-EO03315_EST_C_1_pSK_SK	402
			pOP-EO03316_EST_C_1_pSK_SK	461
			pOP-EO03317_EST_C_1_pSK_SK	503
			pOP-EO03318_EST_C_1_pSK_SK	461
			pOP-EO03319_EST_C_1_pSK_SK	450
			pOP-EO03320_EST_C_1_pSK_SK	393
			pOP-EO03323_EST_C_1_pSK_SK	376
			pOP-EO03324_EST_C_1_pSK_SK	463
			pOP-EO03327_EST_C_1_pSK_SK	463
			pOP-EO03328_EST_C_1_pSK_SK	463
			pOP-EO03330_EST_C_1_pSK_SK	488
			pOP-EO03331_EST_C_1_pSK_SK	463
			pOP-EO03332_EST_C_1_pSK_SK	350
			pOP-EO03336_EST_C_1_pSK_SK	464
			pOP-EO03337_EST_C_1_pSK_SK	368
			pOP-EO03339_EST_C_1_pSK_SK	283
			pOP-EO03340_EST_C_1_pSK_SK	464
			pOP-EO03342_EST_C_1_pSK_SK	402
			pOP-EO03344_EST_C_1_pSK_SK	420
			pOP-EO03346_EST_C_1_pSK_SK	463
			pOP-EO03349_EST_C_1_pSK_SK	498
			pOP-EO03350_EST_C_1_pSK_SK	359

			pOP-EO03352_EST_C_1_pSK_SK	316
			pOP-EO03357_EST_C_1_pSK_SK	466
			pOP-EO03358_EST_C_1_pSK_SK	498
			pOP-EO03359_EST_C_1_pSK_SK	468
			pOP-EO03364_EST_C_1_pSK_SK	465
			pOP-EO03368_EST_C_1_pSK_SK	422
			pOP-EO03369_EST_C_1_pSK_SK	463
			pOP-EO03370_EST_C_1_pSK_SK	466
			pOP-EO03371_EST_C_1_pSK_SK	463
			pOP-EO03374_EST_C_1_pSK_SK	373
			pOP-EO03377_EST_C_1_pSK_SK	392
			pOP-EO03378_EST_C_1_pSK_SK	460
			pOP-EO03379_EST_C_1_pSK_SK	476
			pOP-EO03381_EST_C_1_pSK_SK	460
			pOP-EO03383_EST_C_1_pSK_SK	465
			pOP-EO03389_EST_C_1_pSK_SK	412
			pOP-EO03390_EST_C_1_pSK_SK	370
			pOP-EO03391_EST_C_1_pSK_SK	463
			pOP-EO03395_EST_C_1_pSK_SK	416
			pOP-EO03397_EST_C_1_pSK_SK	466
			pOP-EO03401_EST_C_1_pSK_SK	451
			pOP-EO03408_EST_C_1_pSK_SK	362
			pOP-EO03409_EST_C_1_pSK_SK	270
			pOP-EO03412_EST_C_1_pSK_SK	496
			pOP-EO03416_EST_C_1_pSK_SK	444
			pOP-EO03418_EST_C_1_pSK_SK	487
			pOP-EO03419_EST_C_1_pSK_SK	467
			pOP-EO03420_EST_C_1_pSK_SK	459
			pOP-EO03423_EST_C_1_pSK_SK	280
			pOP-EO03425_EST_C_1_pSK_SK	354
			pOP-EO03431_EST_C_1_pSK_SK	545
			pOP-EO03432_EST_C_1_pSK_SK	519
			pOP-EO03435_EST_C_1_pSK_SK	537
			pOP-EO03437_EST_C_1_pSK_SK	538
			pOP-EO03439_EST_C_1_pSK_SK	536
			pOP-EO03440_EST_C_1_pSK_SK	520
			pOP-EO03442_EST_C_1_pSK_SK	530
			pOP-EO03444_EST_C_1_pSK_SK	534
			pOP-EO03445_EST_C_1_pSK_SK	526
			pOP-EO03450_EST_C_1_pSK_SK	492
			pOP-EO03453_EST_C_1_pSK_SK	504
			pOP-EO03455_EST_C_1_pSK_SK	480
			pOP-EO03459_EST_C_1_pSK_SK	534
			pOP-EO03462_EST_C_1_pSK_SK	540
			pOP-EO03465_EST_C_1_pSK_SK	503
			pOP-EO03466_EST_C_1_pSK_SK	534
			pOP-EO03468_EST_C_1_pSK_SK	539
			pOP-EO03469_EST_C_1_pSK_SK	485
			pOP-EO03470_EST_C_1_pSK_SK	528
			pOP-EO03472_EST_C_1_pSK_SK	540
			pOP-EO03473_EST_C_1_pSK_SK	494
			pOP-EO03474_EST_C_1_pSK_SK	459
			pOP-EO03475_EST_C_1_pSK_SK	385
			pOP-EO03477_EST_C_1_pSK_SK	544
			pOP-EO03481_EST_C_1_pSK_SK	320
			pOP-EO03482_EST_C_1_pSK_SK	542

			pOP-EO03488_EST_C_1_pSK_SK	540
			pOP-EO03490_EST_C_1_pSK_SK	529
			pOP-EO03492_EST_C_1_pSK_SK	270
			pOP-EO03493_EST_C_1_pSK_SK	536
			pOP-EO03495_EST_C_1_pSK_SK	548
			pOP-EO03497_EST_C_1_pSK_SK	540
			pOP-EO03498_EST_C_1_pSK_SK	523
			pOP-EO03505_EST_C_1_pSK_SK	531
			pOP-EO03509_EST_C_1_pSK_SK	455
			pOP-EO03510_EST_C_1_pSK_SK	428
			pOP-EO03511_EST_C_1_pSK_SK	427
			pOP-EO03512_EST_C_1_pSK_SK	462
			pOP-EO03513_EST_C_1_pSK_SK	533
			pOP-EO03515_EST_C_1_pSK_SK	434
			pOP-EO03517_EST_C_1_pSK_SK	523
			pOP-EO03518_EST_C_1_pSK_SK	372
			pOP-EO03521_EST_C_1_pSK_SK	253
			pOP-EO03522_EST_C_1_pSK_SK	384
			pOP-EO03525_EST_C_1_pSK_SK	492
			pOP-EO03528_EST_C_1_pSK_SK	537
			pOP-EO03529_EST_C_1_pSK_SK	511
			pOP-EO03531_EST_C_1_pSK_SK	489
			pOP-EO03534_EST_C_1_pSK_SK	459
			pOP-EO03535_EST_C_1_pSK_SK	236
			pOP-EO03536_EST_C_1_pSK_SK	528
			pOP-EO03537_EST_C_1_pSK_SK	405
			pOP-EO03539_EST_C_1_pSK_SK	301
			pOP-EO03540_EST_C_1_pSK_SK	341
			pOP-EO03543_EST_C_1_pSK_SK	502
			pOP-EO03544_EST_C_1_pSK_SK	256
			pOP-EO03546_EST_C_1_pSK_SK	433
			pOP-EO03551_EST_C_1_pSK_SK	467
			pOP-EO03552_EST_C_1_pSK_SK	542
			pOP-EO03557_EST_C_1_pSK_SK	310
			pOP-EO03559_EST_C_1_pSK_SK	503
			pOP-EO03561_EST_C_1_pSK_SK	401
			pOP-EO03562_EST_C_1_pSK_SK	503
			pOP-EO03569_EST_C_1_pSK_SK	508
			pOP-EO03575_EST_C_1_pSK_SK	500
			pOP-EO03577_EST_C_1_pSK_SK	548
			pOP-EO03582_EST_C_1_pSK_SK	495
			pOP-EO03583_EST_C_1_pSK_SK	543
			pOP-EO03588_EST_C_1_pSK_SK	557
			pOP-EO03590_EST_C_1_pSK_SK	332
			pOP-EO03593_EST_C_1_pSK_SK	318
			pOP-EO03595_EST_C_1_pSK_SK	418
			pOP-EO03596_EST_C_1_pSK_SK	436
			pOP-EO03599_EST_C_1_pSK_SK	475
			pOP-EO03600_EST_C_1_pSK_SK	402
			pOP-EO03601_EST_C_1_pSK_SK	480
			pOP-EO03604_EST_C_1_pSK_SK	423
			pOP-EO03607_EST_C_1_pSK_SK	476
			pOP-EO03608_EST_C_1_pSK_SK	478
			pOP-EO03611_EST_C_1_pSK_SK	280
			pOP-EO03615_EST_C_1_pSK_SK	474
			pOP-EO03617_EST_C_1_pSK_SK	480

			pOP-EO03620_EST_C_1_pSK_SK	189
			pOP-EO03622_EST_C_1_pSK_SK	258
			pOP-EO03624_EST_C_1_pSK_SK	475
			pOP-EO03625_EST_C_1_pSK_SK	281
			pOP-EO03626_EST_C_1_pSK_SK	467
			pOP-EO03627_EST_C_1_pSK_SK	340
			pOP-EO03630_EST_C_1_pSK_SK	307
			pOP-EO03634_EST_C_1_pSK_SK	393
			pOP-EO03638_EST_C_1_pSK_SK	326
			pOP-EO03640_EST_C_1_pSK_SK	471
			pOP-EO03642_EST_C_1_pSK_SK	348
			pOP-EO03643_EST_C_1_pSK_SK	295
			pOP-EO03644_EST_C_1_pSK_SK	472
			pOP-EO03649_EST_C_1_pSK_SK	442
			pOP-EO03650_EST_C_1_pSK_SK	318
			pOP-EO03651_EST_C_1_pSK_SK	474
			pOP-EO03652_EST_C_1_pSK_SK	472
			pOP-EO03654_EST_C_1_pSK_SK	304
			pOP-EO03659_EST_C_1_pSK_SK	260
			pOP-EO03660_EST_C_1_pSK_SK	332
			pOP-EO03661_EST_C_1_pSK_SK	453
			pOP-EO03664_EST_C_1_pSK_SK	480
			pOP-EO03665_EST_C_1_pSK_SK	321
			pOP-EO03666_EST_C_1_pSK_SK	463
			pOP-EO03668_EST_C_1_pSK_SK	232
			pOP-EO03671_EST_C_1_pSK_SK	335
			pOP-EO03672_EST_C_1_pSK_SK	476
			pOP-EO03674_EST_C_1_pSK_SK	335
			pOP-EO03675_EST_C_1_pSK_SK	266
			pOP-EO03676_EST_C_1_pSK_SK	447
			pOP-EO03677_EST_C_1_pSK_SK	459
			pOP-EO03678_EST_C_1_pSK_SK	444
			pOP-EO03680_EST_C_1_pSK_SK	474
			pOP-EO03685_EST_C_1_pSK_SK	361
			pOP-EO03687_EST_C_1_pSK_SK	360
			pOP-EO03689_EST_C_1_pSK_SK	319
			pOP-EO03690_EST_C_1_pSK_SK	359
			pOP-EO03693_EST_C_1_pSK_SK	361
			pOP-EO03695_EST_C_1_pSK_SK	362
			pOP-EO03696_EST_C_1_pSK_SK	357
			pOP-EO03697_EST_C_1_pSK_SK	350
			pOP-EO03698_EST_C_1_pSK_SK	360
			pOP-EO03701_EST_C_1_pSK_SK	361
			pOP-EO03703_EST_C_1_pSK_SK	312
			pOP-EO03705_EST_C_1_pSK_SK	360
			pOP-EO03706_EST_C_1_pSK_SK	359
			pOP-EO03708_EST_C_1_pSK_SK	359
			pOP-EO03713_EST_C_1_pSK_SK	362
			pOP-EO03714_EST_C_1_pSK_SK	359
			pOP-EO03716_EST_C_1_pSK_SK	300
			pOP-EO03717_EST_C_1_pSK_SK	357
			pOP-EO03719_EST_C_1_pSK_SK	356
			pOP-EO03724_EST_C_1_pSK_SK	433
			pOP-EO03729_EST_C_1_pSK_SK	379
			pOP-EO03730_EST_C_1_pSK_SK	425
			pOP-EO03732_EST_C_1_pSK_SK	535

			pOP-EO03744_EST_C_1_pSK_SK	521
			pOP-EO03747_EST_C_1_pSK_SK	522
			pOP-EO03748_EST_C_1_pSK_SK	496
			pOP-EO03754_EST_C_1_pSK_SK	535
			pOP-EO03763_EST_C_1_pSK_SK	493
			pOP-EO03766_EST_C_1_pSK_SK	534
			pOP-EO03768_EST_C_1_pSK_SK	341
			pOP-EO03773_EST_C_1_pSK_SK	534
			pOP-EO03778_EST_C_1_pSK_SK	531
			pOP-EO03783_EST_C_1_pSK_SK	516
			pOP-EO03793_EST_C_1_pSK_SK	420
			pOP-EO03797_EST_C_1_pSK_SK	497
			pOP-EO03798_EST_C_1_pSK_SK	484
			pOP-EO03801_EST_C_1_pSK_SK	503
			pOP-EO03809_EST_C_1_pSK_SK	410
			pOP-EO03812_EST_C_1_pSK_SK	477
			pOP-EO03816_EST_C_1_pSK_SK	464
			pOP-EO03817_EST_C_1_pSK_SK	481
			pOP-EO03818_EST_C_1_pSK_SK	526
			pOP-EO03822_EST_C_1_pSK_SK	102
			pOP-EO03824_EST_C_1_pSK_SK	510
			pOP-EO03834_EST_C_1_pSK_SK	432
			pOP-EO03835_EST_C_1_pSK_SK	431
			pOP-EO03836_EST_C_1_pSK_SK	491
			pOP-EO03840_EST_C_1_pSK_SK	524
			pOP-EO03844_EST_C_1_pSK_SK	217
			pOP-EO03848_EST_C_1_pSK_SK	516
			pOP-EO03859_EST_C_1_pSK_SK	518
			pOP-EO03861_EST_C_1_pSK_SK	518
			pOP-EO03862_EST_C_1_pSK_SK	520
			pOP-EO03863_EST_C_1_pSK_SK	474
			pOP-EO03870_EST_C_1_pSK_SK	517
			pOP-EO03872_EST_C_1_pSK_SK	323
			pOP-EO03873_EST_C_1_pSK_SK	471
			pOP-EO03876_EST_C_1_pSK_SK	568
			pOP-EO03879_EST_C_1_pSK_SK	574
			pOP-EO03880_EST_C_1_pSK_SK	612
			pOP-EO03885_EST_C_1_pSK_SK	566
			pOP-EO03889_EST_C_1_pSK_SK	609
			pOP-EO03893_EST_C_1_pSK_SK	455
			pOP-EO03894_EST_C_1_pSK_SK	526
			pOP-EO03896_EST_C_1_pSK_SK	503
			pOP-EO03898_EST_C_1_pSK_SK	597
			pOP-EO03904_EST_C_1_pSK_SK	517
			pOP-EO03911_EST_C_1_pSK_SK	530
			pOP-EO03915_EST_C_1_pSK_SK	171
			pOP-EO03917_EST_C_1_pSK_SK	334
			pOP-EO03918_EST_C_1_pSK_SK	553
			pOP-EO03919_EST_C_1_pSK_SK	532
			pOP-EO03920_EST_C_1_pSK_SK	532
			pOP-EO03921_EST_C_1_pSK_SK	537
			pOP-EO03931_EST_C_1_pSK_SK	520
			pOP-EO03935_EST_C_1_pSK_SK	530
			pOP-EO03947_EST_C_1_pSK_SK	486
			pOP-EO03951_EST_C_1_pSK_SK	488
			pOP-EO03952_EST_C_1_pSK_SK	489

			pOP-EO03954_EST_C_1_pSK_SK	492
			pOP-EO03955_EST_C_1_pSK_SK	490
			pOP-EO03957_EST_C_1_pSK_SK	357
			pOP-EO03962_EST_C_1_pSK_SK	435
			pOP-EO03967_EST_C_1_pSK_SK	491
			pOP-EO03985_EST_C_1_pSK_SK	414
			pOP-EO03988_EST_C_1_pSK_SK	586
			pOP-EO03989_EST_C_1_pSK_SK	559
			pOP-EO03999_EST_C_1_pSK_SK	539
			pOP-EO04000_EST_C_1_pSK_SK	542
			pOP-EO04009_EST_C_1_pSK_SK	495
			pOP-EO04014_EST_C_1_pSK_SK	424
			pOP-EO04017_EST_C_1_pSK_SK	518
			pOP-EO04034_EST_C_1_pSK_SK	512
			pOP-EO04044_EST_C_1_pSK_SK	643
			pOP-EO04045_EST_C_1_pSK_SK	590
			pOP-EO04057_EST_C_1_pSK_SK	650
			pOP-EO04061_EST_C_1_pSK_SK	436
			pOP-EO04064_EST_C_1_pSK_SK	423
			pOP-EO04070_EST_C_1_pSK_SK	541
			pOP-EO04071_EST_C_1_pSK_SK	555
			pOP-EO04076_EST_C_1_pSK_SK	449
			pOP-EO04077_EST_C_1_pSK_SK	516
			pOP-EO04080_EST_C_1_pSK_SK	549
			pOP-EO04095_EST_C_1_pSK_SK	437
			pOP-EO04102_EST_C_1_pSK_SK	535
			pOP-EO04109_EST_C_1_pSK_SK	557
			pOP-EO04125_EST_C_1_pSK_SK	431
			pOP-EO04227_EST_C_1_pSK_SK	246
			pOP-EO04233_EST_C_1_pSK_SK	469
			pOP-EO04237_EST_C_1_pSK_SK	336
			pOP-EO04238_EST_C_1_pSK_SK	320
			pOP-EO04240_EST_C_1_pSK_SK	378
			pOP-EO04241_EST_C_1_pSK_SK	500
			pOP-EO04242_EST_C_1_pSK_SK	201
			pOP-EO04243_EST_C_1_pSK_SK	388
			pOP-EO04244_EST_C_1_pSK_SK	324
			pOP-EO04245_EST_C_1_pSK_SK	389
			pOP-EO04249_EST_C_1_pSK_SK	289
			pOP-EO04250_EST_C_1_pSK_SK	217
			pOP-EO04256_EST_C_1_pSK_SK	365
			pOP-EO04260_EST_C_1_pSK_SK	365
			pOP-EO04264_EST_C_1_pSK_SK	267
			pOP-EO04265_EST_C_1_pSK_SK	304
			pOP-EO04268_EST_C_1_pSK_SK	435
			pOP-EO04270_EST_C_1_pSK_SK	485
			pOP-EO04274_EST_C_1_pSK_SK	377
			pOP-EO04276_EST_C_1_pSK_SK	409
			pOP-EO04283_EST_C_1_pSK_SK	459
			pOP-EO04285_EST_C_1_pSK_SK	377
			pOP-EO04286_EST_C_1_pSK_SK	197
			pOP-EO04289_EST_C_1_pSK_SK	524
			pOP-EO04290_EST_C_1_pSK_SK	353
			pOP-EO04293_EST_C_1_pSK_SK	339
			pOP-EO04301_EST_C_1_pSK_SK	297
			pOP-EO04306_EST_C_1_pSK_SK	283

			pOP-EO04318_EST_C_1_pSK_SK	356
			pOP-EO04319_EST_C_1_pSK_SK	357
			pOP-EO04321_EST_C_1_pSK_SK	290
			pOP-EO04322_EST_C_1_pSK_SK	349
			pOP-EO04324_EST_C_1_pSK_SK	344
			pOP-EO04328_EST_C_1_pSK_SK	331
			pOP-EO04329_EST_C_1_pSK_SK	409
			pOP-EO04330_EST_C_1_pSK_SK	482
			pOP-EO04331_EST_C_1_pSK_SK	363
			pOP-EO04334_EST_C_1_pSK_SK	190
			pOP-EO04335_EST_C_1_pSK_SK	382
			pOP-EO04340_EST_C_1_pSK_SK	515
			pOP-EO04341_EST_C_1_pSK_SK	524
			pOP-EO04350_EST_C_1_pSK_SK	534
			pOP-EO04355_EST_C_1_pSK_SK	374
			pOP-EO04357_EST_C_1_pSK_SK	484
			pOP-EO04358_EST_C_1_pSK_SK	380
			pOP-EO04360_EST_C_1_pSK_SK	402
			pOP-EO04361_EST_C_1_pSK_SK	449
			pOP-EO04364_EST_C_1_pSK_SK	432
			pOP-EO04370_EST_C_1_pSK_SK	530
			pOP-EO04375_EST_C_1_pSK_SK	518
			pOP-EO04379_EST_C_1_pSK_SK	362
			pOP-EO04383_EST_C_1_pSK_SK	519
			pOP-EO04384_EST_C_1_pSK_SK	454
			pOP-EO04392_EST_C_1_pSK_SK	476
			pOP-EO04393_EST_C_1_pSK_SK	356
			pOP-EO04395_EST_C_1_pSK_SK	471
			pOP-EO04399_EST_C_1_pSK_SK	428
			pOP-EO04404_EST_C_1_pSK_SK	529
			pOP-EO04405_EST_C_1_pSK_SK	505
			pOP-EO04407_EST_C_1_pSK_SK	482
			pOP-EO04412_EST_C_1_pSK_SK	480
			pOP-EO04413_EST_C_1_pSK_SK	501
			pOP-EO04419_EST_C_1_pSK_SK	531
			pOP-EO04423_EST_C_1_pSK_SK	497
			pOP-EO04426_EST_C_1_pSK_SK	534
			pOP-EO04429_EST_C_1_pSK_SK	482
			pOP-EO04431_EST_C_1_pSK_SK	379
			pOP-EO04432_EST_C_1_pSK_SK	480
			pOP-EO04436_EST_C_1_pSK_SK	509
			pOP-EO04438_EST_C_1_pSK_SK	180
			pOP-EO04440_EST_C_1_pSK_SK	372
			pOP-EO04443_EST_C_1_pSK_SK	256
			pOP-EO04446_EST_C_1_pSK_SK	532
			pOP-EO04448_EST_C_1_pSK_SK	483
			pOP-EO04454_EST_C_1_pSK_SK	501
			pOP-EO04458_EST_C_1_pSK_SK	481
			pOP-EO04462_EST_C_1_pSK_SK	438
			pOP-EO04464_EST_C_1_pSK_SK	471
			pOP-EO04466_EST_C_1_pSK_SK	445
			pOP-EO04470_EST_C_1_pSK_SK	247
			pOP-EO04471_EST_C_1_pSK_SK	279
			pOP-EO04473_EST_C_1_pSK_SK	342
			pOP-EO04474_EST_C_1_pSK_SK	478
			pOP-EO04482_EST_C_1_pSK_SK	435

			pOP-EO04483_EST_C_1_pSK_SK	382
			pOP-EO04484_EST_C_1_pSK_SK	499
			pOP-EO04488_EST_C_1_pSK_SK	483
			pOP-EO04489_EST_C_1_pSK_SK	324
			pOP-EO04490_EST_C_1_pSK_SK	350
			pOP-EO04491_EST_C_1_pSK_SK	529
			pOP-EO04496_EST_C_1_pSK_SK	537
			pOP-EO04497_EST_C_1_pSK_SK	441
			pOP-EO04499_EST_C_1_pSK_SK	536
			pOP-EO04509_EST_C_1_pSK_SK	441
			pOP-EO04510_EST_C_1_pSK_SK	536
			pOP-EO04514_EST_C_1_pSK_SK	517
			pOP-EO04516_EST_C_1_pSK_SK	540
			pOP-EO04519_EST_C_1_pSK_SK	529
			pOP-EO04523_EST_C_1_pSK_SK	536
			pOP-EO04525_EST_C_1_pSK_SK	379
			pOP-EO04527_EST_C_1_pSK_SK	507
			pOP-EO04530_EST_C_1_pSK_SK	532
			pOP-EO04533_EST_C_1_pSK_SK	409
			pOP-EO04534_EST_C_1_pSK_SK	544
			pOP-EO04538_EST_C_1_pSK_SK	333
			pOP-EO04540_EST_C_1_pSK_SK	519
			pOP-EO04544_EST_C_1_pSK_SK	431
			pOP-EO04545_EST_C_1_pSK_SK	513
			pOP-EO04546_EST_C_1_pSK_SK	524
			pOP-EO04547_EST_C_1_pSK_SK	412
			pOP-EO04549_EST_C_1_pSK_SK	429
			pOP-EO04551_EST_C_1_pSK_SK	357
			pOP-EO04552_EST_C_1_pSK_SK	541
			pOP-EO04554_EST_C_1_pSK_SK	546
			pOP-EO04558_EST_C_1_pSK_SK	548
			pOP-EO04559_EST_C_1_pSK_SK	544
			pOP-EO04564_EST_C_1_pSK_SK	546
			pOP-EO04565_EST_C_1_pSK_SK	530
			pOP-EO04572_EST_C_1_pSK_SK	491
			pOP-EO04573_EST_C_1_pSK_SK	548
			pOP-EO04575_EST_C_1_pSK_SK	493
			pOP-EO04577_EST_C_1_pSK_SK	549
			pOP-EO04583_EST_C_1_pSK_SK	551
			pOP-EO04587_EST_C_1_pSK_SK	547
			pOP-EO04588_EST_C_1_pSK_SK	499
			pOP-EO04589_EST_C_1_pSK_SK	550
			pOP-EO04594_EST_C_1_pSK_SK	511
			pOP-EO04597_EST_C_1_pSK_SK	539
			pOP-EO04602_EST_C_1_pSK_SK	542
			pOP-EO04606_EST_C_1_pSK_SK	530
			pOP-EO04611_EST_C_1_pSK_SK	555
			pOP-EO04612_EST_C_1_pSK_SK	503
			pOP-EO04614_EST_C_1_pSK_SK	532
			pOP-EO04619_EST_C_1_pSK_SK	541
			pOP-EO04620_EST_C_1_pSK_SK	329
			pOP-EO04625_EST_C_1_pSK_SK	518
			pOP-EO04626_EST_C_1_pSK_SK	408
			pOP-EO04634_EST_C_1_pSK_SK	530
			pOP-EO04635_EST_C_1_pSK_SK	494
			pOP-EO04637_EST_C_1_pSK_SK	533

			pOP-EO04641_EST_C_1_pSK_SK	367
			pOP-EO04643_EST_C_1_pSK_SK	527
			pOP-EO04646_EST_C_1_pSK_SK	510
			pOP-EO04653_EST_C_1_pSK_SK	506
			pOP-EO04654_EST_C_1_pSK_SK	411
			pOP-EO04657_EST_C_1_pSK_SK	530
			pOP-EO04660_EST_C_1_pSK_SK	522
			pOP-EO04662_EST_C_1_pSK_SK	522
			pOP-EO04667_EST_C_1_pSK_SK	518
			pOP-EO04669_EST_C_1_pSK_SK	535
			pOP-EO04672_EST_C_1_pSK_SK	538
			pOP-EO04675_EST_C_1_pSK_SK	509
			pOP-EO04676_EST_C_1_pSK_SK	528
			pOP-EO04679_EST_C_1_pSK_SK	537
			pOP-EO04681_EST_C_1_pSK_SK	534
			pOP-EO04682_EST_C_1_pSK_SK	538
			pOP-EO04683_EST_C_1_pSK_SK	532
			pOP-EO04684_EST_C_1_pSK_SK	614
			pOP-EO04687_EST_C_1_pSK_SK	532
			pOP-EO04690_EST_C_1_pSK_SK	533
			pOP-EO04693_EST_C_1_pSK_SK	527
			pOP-EO04695_EST_C_1_pSK_SK	532
			pOP-EO04698_EST_C_1_pSK_SK	522
			pOP-EO04708_EST_C_1_pSK_SK	532
			pOP-EO04709_EST_C_1_pSK_SK	524
			pOP-EO04711_EST_C_1_pSK_SK	528
			pOP-EO04712_EST_C_1_pSK_SK	524
			pOP-EO04718_EST_C_1_pSK_SK	517
			pOP-EO04726_EST_C_1_pSK_SK	517
			pOP-EO04727_EST_C_1_pSK_SK	531
			pOP-EO04728_EST_C_1_pSK_SK	501
			pOP-EO04735_EST_C_1_pSK_SK	533
			pOP-EO04737_EST_C_1_pSK_SK	423
			pOP-EO04740_EST_C_1_pSK_SK	526
			pOP-EO04743_EST_C_1_pSK_SK	418
			pOP-EO04746_EST_C_1_pSK_SK	538
			pOP-EO04748_EST_C_1_pSK_SK	465
			pOP-EO04749_EST_C_1_pSK_SK	516
			pOP-EO04752_EST_C_1_pSK_SK	495
			pOP-EO04754_EST_C_1_pSK_SK	526
			pOP-EO04756_EST_C_1_pSK_SK	525
			pOP-EO04758_EST_C_1_pSK_SK	519
			pOP-EO04760_EST_C_1_pSK_SK	526
			pOP-EO04767_EST_C_1_pSK_SK	523
			pOP-EO04770_EST_C_1_pSK_SK	528
			pOP-EO04771_EST_C_1_pSK_SK	515
			pOP-EO04796_EST_C_1_pSK_SK	575
			pOP-EO04799_EST_C_1_pSK_SK	546
			pOP-EO04807_EST_C_1_pSK_SK	336
			pOP-EO04809_EST_C_1_pSK_SK	475
			pOP-EO04811_EST_C_1_pSK_SK	519
			pOP-EO04812_EST_C_1_pSK_SK	506
			pOP-EO04814_EST_C_1_pSK_SK	416
			pOP-EO04825_EST_C_1_pSK_SK	497
			pOP-EO04826_EST_C_1_pSK_SK	561
			pOP-EO04834_EST_C_1_pSK_SK	578

			pOP-EO04838_EST_C_1_pSK_SK	318
			pOP-EO04840_EST_C_1_pSK_SK	508
			pOP-EO04846_EST_C_1_pSK_SK	533
			pOP-EO04848_EST_C_1_pSK_SK	573
			pOP-EO04856_EST_C_1_pSK_SK	451
			pOP-EO04860_EST_C_1_pSK_SK	509
			pOP-EO04861_EST_C_1_pSK_SK	511
			pOP-EO04866_EST_C_1_pSK_SK	515
			pOP-EO04868_EST_C_1_pSK_SK	504
			pOP-EO04869_EST_C_1_pSK_SK	511
			pOP-EO04872_EST_C_1_pSK_SK	501
			pOP-EO04874_EST_C_1_pSK_SK	393
			pOP-EO04875_EST_C_1_pSK_SK	433
			pOP-EO04878_EST_C_1_pSK_SK	447
			pOP-EO04882_EST_C_1_pSK_SK	514
			pOP-EO04885_EST_C_1_pSK_SK	514
			pOP-EO04894_EST_C_1_pSK_SK	505
			pOP-EO04895_EST_C_1_pSK_SK	498
			pOP-EO04898_EST_C_1_pSK_SK	523
			pOP-EO04899_EST_C_1_pSK_SK	522
			pOP-EO04900_EST_C_1_pSK_SK	513
			pOP-EO04901_EST_C_1_pSK_SK	376
			pOP-EO04909_EST_C_1_pSK_SK	518
			pOP-EO04910_EST_C_1_pSK_SK	474
			pOP-EO04911_EST_C_1_pSK_SK	524
			pOP-EO04914_EST_C_1_pSK_SK	471
			pOP-EO04917_EST_C_1_pSK_SK	515
			pOP-EO04918_EST_C_1_pSK_SK	474
			pOP-EO04925_EST_C_1_pSK_SK	519
			pOP-EO04927_EST_C_1_pSK_SK	372
			pOP-EO04930_EST_C_1_pSK_SK	523
			pOP-EO04931_EST_C_1_pSK_SK	449
			pOP-EO04933_EST_C_1_pSK_SK	511
			pOP-EO04937_EST_C_1_pSK_SK	513
			pOP-EO04942_EST_C_1_pSK_SK	511
			pOP-EO04946_EST_C_1_pSK_SK	493
			pOP-EO04950_EST_C_1_pSK_SK	518
			pOP-EO04952_EST_C_1_pSK_SK	462
			pOP-EO04954_EST_C_1_pSK_SK	467
			pOP-EO04965_EST_C_1_pSK_SK	512
			pOP-EO04966_EST_C_1_pSK_SK	518
			pOP-EO04970_EST_C_1_pSK_SK	524
			pOP-EO04972_EST_C_1_pSK_SK	511
			pOP-EO04974_EST_C_1_pSK_SK	502
			pOP-EO04976_EST_C_1_pSK_SK	515
			pOP-EO04977_EST_C_1_pSK_SK	516
			pOP-EO04978_EST_C_1_pSK_SK	512
			pOP-EO04982_EST_C_1_pSK_SK	360
			pOP-EO04984_EST_C_1_pSK_SK	428
			pOP-EO04985_EST_C_1_pSK_SK	464
			pOP-EO04986_EST_C_1_pSK_SK	507
			pOP-EO04991_EST_C_1_pSK_SK	499
			pOP-EO04993_EST_C_1_pSK_SK	505
			pOP-EO04998_EST_C_1_pSK_SK	502
			pOP-EO05000_EST_C_1_pSK_SK	477
			pOP-EO05001_EST_C_1_pSK_SK	489

			pOP-EO05003_EST_C_1_pSK_SK	516
			pOP-EO05007_EST_C_1_pSK_SK	512
			pOP-EO05010_EST_C_1_pSK_SK	411
			pOP-EO05014_EST_C_1_pSK_SK	533
			pOP-EO05016_EST_C_1_pSK_SK	467
			pOP-EO05017_EST_C_1_pSK_SK	526
			pOP-EO05018_EST_C_1_pSK_SK	418
			pOP-EO05020_EST_C_1_pSK_SK	508
			pOP-EO05023_EST_C_1_pSK_SK	510
			pOP-EO05024_EST_C_1_pSK_SK	524
			pOP-EO05026_EST_C_1_pSK_SK	537
			pOP-EO05031_EST_C_1_pSK_SK	512
			pOP-EO05039_EST_C_1_pSK_SK	538
			pOP-EO05041_EST_C_1_pSK_SK	532
			pOP-EO05045_EST_C_1_pSK_SK	527
			pOP-EO05046_EST_C_1_pSK_SK	516
			pOP-EO05050_EST_C_1_pSK_SK	528
			pOP-EO05055_EST_C_1_pSK_SK	422
			pOP-EO05057_EST_C_1_pSK_SK	507
			pOP-EO05058_EST_C_1_pSK_SK	518
			pOP-EO05065_EST_C_1_pSK_SK	512
			pOP-EO05066_EST_C_1_pSK_SK	519
			pOP-EO05069_EST_C_1_pSK_SK	529
			pOP-EO05070_EST_C_1_pSK_SK	482
			pOP-EO05073_EST_C_1_pSK_SK	531
			pOP-EO05075_EST_C_1_pSK_SK	517
			pOP-EO05076_EST_C_1_pSK_SK	496
			pOP-EO05078_EST_C_1_pSK_SK	469
			pOP-EO05081_EST_C_1_pSK_SK	533
			pOP-EO05083_EST_C_1_pSK_SK	522
			pOP-EO05087_EST_C_1_pSK_SK	533
			pOP-EO05090_EST_C_1_pSK_SK	524
			pOP-EO05092_EST_C_1_pSK_SK	522
			pOP-EO05101_EST_C_1_pSK_SK	498
			pOP-EO05105_EST_C_1_pSK_SK	523
			pOP-EO05110_EST_C_1_pSK_SK	247
			pOP-EO05113_EST_C_1_pSK_SK	457
			pOP-EO05115_EST_C_1_pSK_SK	494
			pOP-EO05117_EST_C_1_pSK_SK	537
			pOP-EO05126_EST_C_1_pSK_SK	524
			pOP-EO05128_EST_C_1_pSK_SK	532
			pOP-EO05129_EST_C_1_pSK_SK	376
			pOP-EO05131_EST_C_1_pSK_SK	499
			pOP-EO05132_EST_C_1_pSK_SK	500
			pOP-EO05134_EST_C_1_pSK_SK	537
			pOP-EO05135_EST_C_1_pSK_SK	504
			pOP-EO05136_EST_C_1_pSK_SK	532
			pOP-EO05138_EST_C_1_pSK_SK	406
			pOP-EO05142_EST_C_1_pSK_SK	417
			pOP-EO05149_EST_C_1_pSK_SK	526
			pOP-EO05152_EST_C_1_pSK_SK	532
			pOP-EO05153_EST_C_1_pSK_SK	459
			pOP-EO05157_EST_C_1_pSK_SK	535
			pOP-EO05158_EST_C_1_pSK_SK	499
			pOP-EO05159_EST_C_1_pSK_SK	469
			pOP-EO05164_EST_C_1_pSK_SK	255

			pOP-EO05168_EST_C_1_pSK_SK	464
			pOP-EO05178_EST_C_1_pSK_SK	539
			pOP-EO05179_EST_C_1_pSK_SK	491
			pOP-EO05180_EST_C_1_pSK_SK	404
			pOP-EO05182_EST_C_1_pSK_SK	501
			pOP-EO05187_EST_C_1_pSK_SK	427
			pOP-EO05195_EST_C_1_pSK_SK	525
			pOP-EO05196_EST_C_1_pSK_SK	495
			pOP-EO05201_EST_C_1_pSK_SK	533
			pOP-EO05202_EST_C_1_pSK_SK	528
			pOP-EO05213_EST_C_1_pSK_SK	530
			pOP-EO05233_EST_C_1_pSK_SK	386
			pOP-EO05241_EST_C_1_pSK_SK	407
			pOP-EO05243_EST_C_1_pSK_SK	480
			pOP-EO05251_EST_C_1_pSK_SK	504
			pOP-EO05258_EST_C_1_pSK_SK	510
			pOP-EO05262_EST_C_1_pSK_SK	453
			pOP-EO05263_EST_C_1_pSK_SK	436
			pOP-EO05267_EST_C_1_pSK_SK	533
			pOP-EO05269_EST_C_1_pSK_SK	464
			pOP-EO05274_EST_C_1_pSK_SK	556
			pOP-EO05276_EST_C_1_pSK_SK	547
			pOP-EO05279_EST_C_1_pSK_SK	583
			pOP-EO05284_EST_C_1_pSK_SK	526
			pOP-EO05292_EST_C_1_pSK_SK	539
			pOP-EO05299_EST_C_1_pSK_SK	567
			pOP-EO05305_EST_C_1_pSK_SK	574
			pOP-EO05308_EST_C_1_pSK_SK	564
			pOP-EO05312_EST_C_1_pSK_SK	483
			pOP-EO05315_EST_C_1_pSK_SK	445
			pOP-EO05317_EST_C_1_pSK_SK	493
			pOP-EO05318_EST_C_1_pSK_SK	473
			pOP-EO05323_EST_C_1_pSK_SK	578
			pOP-EO05327_EST_C_1_pSK_SK	537
			pOP-EO05330_EST_C_1_pSK_SK	528
			pOP-EO05332_EST_C_1_pSK_SK	595
			pOP-EO05343_EST_C_1_pSK_SK	550
			pOP-EO05344_EST_C_1_pSK_SK	506
			pOP-EO05350_EST_C_1_pSK_SK	547
			pOP-EO05351_EST_C_1_pSK_SK	556
			pOP-EO05353_EST_C_1_pSK_SK	513
			pOP-EO05354_EST_C_1_pSK_SK	558
			pOP-EO05357_EST_C_1_pSK_SK	527
			pOP-EO05359_EST_C_1_pSK_SK	554
			pOP-EO05364_EST_C_1_pSK_SK	540
			pOP-EO05378_EST_C_1_pSK_SK	295
			pOP-EO05380_EST_C_1_pSK_SK	513
			pOP-EO05401_EST_C_1_pSK_SK	548
			pOP-EO05407_EST_C_1_pSK_SK	542
			pOP-EO05414_EST_C_1_pSK_SK	525
			pOP-EO05415_EST_C_1_pSK_SK	536
			pOP-EO05416_EST_C_1_pSK_SK	522
			pOP-EO05419_EST_C_1_pSK_SK	533
			pOP-EO05422_EST_C_1_pSK_SK	543
			pOP-EO05432_EST_C_1_pSK_SK	249
			pOP-EO05440_EST_C_1_pSK_SK	502

			pOP-EO05444_EST_C_1_pSK_SK	518
			pOP-EO05446_EST_C_1_pSK_SK	464
			pOP-EO05448_EST_C_1_pSK_SK	515
			pOP-EO05452_EST_C_1_pSK_SK	526
			pOP-EO05453_EST_C_1_pSK_SK	405
			pOP-EO05454_EST_C_1_pSK_SK	525
			pOP-EO05456_EST_C_1_pSK_SK	509
			pOP-EO05460_EST_C_1_pSK_SK	514
			pOP-EO05465_EST_C_1_pSK_SK	245
			pOP-EO05466_EST_C_1_pSK_SK	487
			pOP-EO05469_EST_C_1_pSK_SK	451
			pOP-EO05470_EST_C_1_pSK_SK	507
			pOP-EO05473_EST_C_1_pSK_SK	245
			pOP-EO05475_EST_C_1_pSK_SK	522
			pOP-EO05478_EST_C_1_pSK_SK	515
			pOP-EO05485_EST_C_1_pSK_SK	518
			pOP-EO05487_EST_C_1_pSK_SK	435
			pOP-EO05501_EST_C_1_pSK_SK	490
			pOP-EO05502_EST_C_1_pSK_SK	468
			pOP-EO05504_EST_C_1_pSK_SK	512
			pOP-EO05506_EST_C_1_pSK_SK	456
			pOP-EO05507_EST_C_1_pSK_SK	452
			pOP-EO05515_EST_C_1_pSK_SK	474
			pOP-EO05518_EST_C_1_pSK_SK	514
			pOP-EO05520_EST_C_1_pSK_SK	388
			pOP-EO05523_EST_C_1_pSK_SK	327
			pOP-EO05524_EST_C_1_pSK_SK	186
			pOP-EO05527_EST_C_1_pSK_SK	484
			pOP-EO05538_EST_C_1_pSK_SK	489
			pOP-EO05540_EST_C_1_pSK_SK	491
			pOP-EO05544_EST_C_1_pSK_SK	486
			pOP-EO05545_EST_C_1_pSK_SK	319
			pOP-EO05548_EST_C_1_pSK_SK	501
			pOP-EO05550_EST_C_1_pSK_SK	479
			pOP-EO05553_EST_C_1_pSK_SK	490
			pOP-EO05554_EST_C_1_pSK_SK	472
			pOP-EO05555_EST_C_1_pSK_SK	421
			pOP-EO05556_EST_C_1_pSK_SK	436
			pOP-EO05557_EST_C_1_pSK_SK	462
			pOP-EO05558_EST_C_1_pSK_SK	434
			pOP-EO05564_EST_C_1_pSK_SK	479
			pOP-EO05569_EST_C_1_pSK_SK	489
			pOP-EO05571_EST_C_1_pSK_SK	504
			pOP-EO05572_EST_C_1_pSK_SK	501
			pOP-EO05573_EST_C_1_pSK_SK	497
			pOP-EO05576_EST_C_1_pSK_SK	501
			pOP-EO05577_EST_C_1_pSK_SK	454
			pOP-EO05578_EST_C_1_pSK_SK	472
			pOP-EO05579_EST_C_1_pSK_SK	504
			pOP-EO05580_EST_C_1_pSK_SK	483
			pOP-EO05583_EST_C_1_pSK_SK	484
			pOP-EO05585_EST_C_1_pSK_SK	497
			pOP-EO05586_EST_C_1_pSK_SK	497
			pOP-EO05588_EST_C_1_pSK_SK	493
			pOP-EO05590_EST_C_1_pSK_SK	486
			pOP-EO05591_EST_C_1_pSK_SK	481

			pOP-EO05595_EST_C_1_pSK_SK	403	
			pOP-EO05597_EST_C_1_pSK_SK	495	
			pOP-EO05598_EST_C_1_pSK_SK	397	
			pOP-EO05600_EST_C_1_pSK_SK	448	
			pOP-EO05601_EST_C_1_pSK_SK	493	
			pOP-EO05602_EST_C_1_pSK_SK	497	
			pOP-EO05603_EST_C_1_pSK_SK	497	
			pOP-EO05604_EST_C_1_pSK_SK	498	
			pOP-EO05605_EST_C_1_pSK_SK	500	
			pOP-EO05607_EST_C_1_pSK_SK	480	
			pOP-EO05608_EST_C_1_pSK_SK	485	
			pOP-EO05609_EST_C_1_pSK_SK	495	
			pOP-EO05610_EST_C_1_pSK_SK	440	
			pOP-EO05611_EST_C_1_pSK_SK	471	
			pOP-EO05612_EST_C_1_pSK_SK	497	
			pOP-EO05618_EST_C_1_pSK_SK	491	
			pOP-EO05624_EST_C_1_pSK_SK	479	
			pOP-EO05625_EST_C_1_pSK_SK	490	
			pOP-EO05626_EST_C_1_pSK_SK	470	
			pOP-EO05627_EST_C_1_pSK_SK	455	
			pOP-EO05630_EST_C_1_pSK_SK	450	
			pOP-EO05631_EST_C_1_pSK_SK	158	
			pOP-EO05636_EST_C_1_pSK_SK	563	
			pOP-EO05638_EST_C_1_pSK_SK	483	
			pOP-EO05639_EST_C_1_pSK_SK	493	
			pOP-EO05640_EST_C_1_pSK_SK	498	
			pOP-EO05642_EST_C_1_pSK_SK	491	
			pOP-EO05643_EST_C_1_pSK_SK	403	
			pOP-EO05645_EST_C_1_pSK_SK	328	
			pOP-EO05648_EST_C_1_pSK_SK	388	
			pOP-EO05649_EST_C_1_pSK_SK	452	
			pOP-EO05652_EST_C_1_pSK_SK	495	
			pOP-EO05653_EST_C_1_pSK_SK	500	
			pOP-EO05656_EST_C_1_pSK_SK	452	
			pOP-EO05657_EST_C_1_pSK_SK	341	
			pOP-EO05659_EST_C_1_pSK_SK	421	
			pOP-EO05660_EST_C_1_pSK_SK	531	
			pOP-EO05663_EST_C_1_pSK_SK	390	
			pOP-EO05667_EST_C_1_pSK_SK	429	
			pOP-EO05669_EST_C_1_pSK_SK	517	
			pOP-EO05670_EST_C_1_pSK_SK	461	
			pOP-EO05672_EST_C_1_pSK_SK	423	
			pOP-EO05675_EST_C_1_pSK_SK	397	
			pOP-EO05679_EST_C_1_pSK_SK	439	
			pOP-EO05681_EST_C_1_pSK_SK	411	
			pOP-EO05682_EST_C_1_pSK_SK	352	
			pOP-EO05683_EST_C_1_pSK_SK	348	
			pOP-EO05685_EST_C_1_pSK_SK	472	
			pOP-EO05692_EST_C_1_pSK_SK	468	
			pOP-EO05695_EST_C_1_pSK_SK	424	
			pOP-EO05697_EST_C_1_pSK_SK	514	
			pOP-EO05699_EST_C_1_pSK_SK	511	
			pOP-EO05701_EST_C_1_pSK_SK	301	
			pOP-EO05703_EST_C_1_pSK_SK	487	
			pOP-EO05705_EST_C_1_pSK_SK	443	
			pOP-EO05706_EST_C_1_pSK_SK	480	

			pOP-EO05712_EST_C_1_pSK_SK	452
			pOP-EO05714_EST_C_1_pSK_SK	523
			pOP-EO05717_EST_C_1_pSK_SK	425
			pOP-EO05719_EST_C_1_pSK_SK	456
			pOP-EO05720_EST_C_1_pSK_SK	391
			pOP-EO05721_EST_C_1_pSK_SK	418
			pOP-EO05723_EST_C_1_pSK_SK	423
			pOP-EO05728_EST_C_1_pSK_SK	562
			pOP-EO05729_EST_C_1_pSK_SK	290
			pOP-EO05730_EST_C_1_pSK_SK	564
			pOP-EO05734_EST_C_1_pSK_SK	394
			pOP-EO05740_EST_C_1_pSK_SK	567
			pOP-EO05741_EST_C_1_pSK_SK	567
			pOP-EO05742_EST_C_1_pSK_SK	512
			pOP-EO05743_EST_C_1_pSK_SK	554
			pOP-EO05744_EST_C_1_pSK_SK	566
			pOP-EO05745_EST_C_1_pSK_SK	567
			pOP-EO05746_EST_C_1_pSK_SK	515
			pOP-EO05752_EST_C_1_pSK_SK	655
			pOP-EO05757_EST_C_1_pSK_SK	595
			pOP-EO05763_EST_C_1_pSK_SK	651
			pOP-EO05764_EST_C_1_pSK_SK	558
			pOP-EO05768_EST_C_1_pSK_SK	570
			pOP-EO05769_EST_C_1_pSK_SK	681
			pOP-EO05771_EST_C_1_pSK_SK	572
			pOP-EO05776_EST_C_1_pSK_SK	441
			pOP-EO05782_EST_C_1_pSK_SK	712
			pOP-EO05788_EST_C_1_pSK_SK	111
			pOP-EO05790_EST_C_1_pSK_SK	560
			pOP-EO05792_EST_C_1_pSK_SK	654
			pOP-EO05795_EST_C_1_pSK_SK	656
			pOP-EO05801_EST_C_1_pSK_SK	487
			pOP-EO05802_EST_C_1_pSK_SK	639
			pOP-EO05803_EST_C_1_pSK_SK	617
			pOP-EO05804_EST_C_1_pSK_SK	732
			pOP-EO05806_EST_C_1_pSK_SK	506
			pOP-EO05809_EST_C_1_pSK_SK	647
			pOP-EO05811_EST_C_1_pSK_SK	562
			pOP-EO05812_EST_C_1_pSK_SK	567
			pOP-EO05815_EST_C_1_pSK_SK	585
			pOP-EO05817_EST_C_1_pSK_SK	621
			pOP-EO05820_EST_C_1_pSK_SK	521
			pOP-EO05821_EST_C_1_pSK_SK	679
			pOP-EO05822_EST_C_1_pSK_SK	712
			pOP-EO05827_EST_C_1_pSK_SK	516
			pOP-EO05828_EST_C_1_pSK_SK	625
			pOP-EO05830_EST_C_1_pSK_SK	703
			pOP-EO05832_EST_C_1_pSK_SK	614
			pOP-EO05833_EST_C_1_pSK_SK	224
			pOP-EO05839_EST_C_1_pSK_SK	643
			pOP-EO05843_EST_C_1_pSK_SK	355
			pOP-EO05848_EST_C_1_pSK_SK	668
			pOP-EO05853_EST_C_1_pSK_SK	583
			pOP-EO05862_EST_C_1_pSK_SK	553
			pOP-EO05865_EST_C_1_pSK_SK	383
			pOP-EO05866_EST_C_1_pSK_SK	638

			pOP-EO05868_EST_C_1_pSK_SK	631
			pOP-EO05875_EST_C_1_pSK_SK	595
			pOP-EO05879_EST_C_1_pSK_SK	476
			pOP-EO05881_EST_C_1_pSK_SK	616
			pOP-EO05883_EST_C_1_pSK_SK	507
			pOP-EO05884_EST_C_1_pSK_SK	219
			pOP-EO05886_EST_C_1_pSK_SK	528
			pOP-EO05887_EST_C_1_pSK_SK	604
			pOP-EO05895_EST_C_1_pSK_SK	347
			pOP-EO05898_EST_C_1_pSK_SK	258
			pOP-EO05903_EST_C_1_pSK_SK	661
			pOP-EO05904_EST_C_1_pSK_SK	493
			pOP-EO05906_EST_C_1_pSK_SK	377
			pOP-EO05907_EST_C_1_pSK_SK	609
			pOP-EO05910_EST_C_1_pSK_SK	636
			pOP-EO05913_EST_C_1_pSK_SK	552
			pOP-EO05916_EST_C_1_pSK_SK	292
			pOP-EO05921_EST_C_1_pSK_SK	656
			pOP-EO05940_EST_C_1_pSK_SK	286
			pOP-EO05941_EST_C_1_pSK_SK	106
			pOP-EO05942_EST_C_1_pSK_SK	663
			pOP-EO05943_EST_C_1_pSK_SK	576
			pOP-EO05944_EST_C_1_pSK_SK	596
			pOP-EO05947_EST_C_1_pSK_SK	502
			pOP-EO05951_EST_C_1_pSK_SK	482
			pOP-EO05952_EST_C_1_pSK_SK	642
			pOP-EO05954_EST_C_1_pSK_SK	649
			pOP-EO05955_EST_C_1_pSK_SK	537
			pOP-EO05961_EST_C_1_pSK_SK	530
			pOP-EO05964_EST_C_1_pSK_SK	493
			pOP-EO05965_EST_C_1_pSK_SK	618
			pOP-EO05966_EST_C_1_pSK_SK	391
			pOP-EO05967_EST_C_1_pSK_SK	620
			pOP-EO05968_EST_C_1_pSK_SK	667
			pOP-EO05974_EST_C_1_pSK_SK	535
			pOP-EO05975_EST_C_1_pSK_SK	406
			pOP-EO05976_EST_C_1_pSK_SK	526
			pOP-EO05977_EST_C_1_pSK_SK	541
			pOP-EO05979_EST_C_1_pSK_SK	563
			pOP-EO05983_EST_C_1_pSK_SK	293
			pOP-EO05984_EST_C_1_pSK_SK	471
			pOP-EO05985_EST_C_1_pSK_SK	562
			pOP-EO05987_EST_C_1_pSK_SK	621
			pOP-EO05988_EST_C_1_pSK_SK	628
			pOP-EO05989_EST_C_1_pSK_SK	499
			pOP-EO05990_EST_C_1_pSK_SK	540
			pOP-EO05992_EST_C_1_pSK_SK	307
			pOP-EO05993_EST_C_1_pSK_SK	541
			pOP-EO05998_EST_C_1_pSK_SK	510
			pOP-EO05999_EST_C_1_pSK_SK	614
			pOP-EO06000_EST_C_1_pSK_SK	277
			pOP-EO06002_EST_C_1_pSK_SK	474
			pOP-EO06003_EST_C_1_pSK_SK	565
			pOP-EO06012_EST_C_1_pSK_SK	517
			pOP-EO06014_EST_C_1_pSK_SK	472
			pOP-EO06015_EST_C_1_pSK_SK	606

			pOP-EO06017_EST_C_1_pSK_SK	561
			pOP-EO06018_EST_C_1_pSK_SK	538
			pOP-EO06020_EST_C_1_pSK_SK	601
			pOP-EO06021_EST_C_1_pSK_SK	502
			pOP-EO06027_EST_C_1_pSK_SK	559
			pOP-EO06031_EST_C_1_pSK_SK	115
			pOP-EO06040_EST_C_1_pSK_SK	489
			pOP-EO06042_EST_C_1_pSK_SK	508
			pOP-EO06043_EST_C_1_pSK_SK	566
			pOP-EO06045_EST_C_1_pSK_SK	694
			pOP-EO06046_EST_C_1_pSK_SK	666
			pOP-EO06047_EST_C_1_pSK_SK	545
			pOP-EO06050_EST_C_1_pSK_SK	581
			pOP-EO06051_EST_C_1_pSK_SK	683
			pOP-EO06055_EST_C_1_pSK_SK	671
			pOP-EO06056_EST_C_1_pSK_SK	465
			pOP-EO06061_EST_C_1_pSK_SK	669
			pOP-EO06062_EST_C_1_pSK_SK	607
			pOP-EO06068_EST_C_1_pSK_SK	444
			pOP-EO06069_EST_C_1_pSK_SK	695
			pOP-EO06070_EST_C_1_pSK_SK	619
			pOP-EO06071_EST_C_1_pSK_SK	569
			pOP-EO06082_EST_C_1_pSK_SK	445
			pOP-EO06083_EST_C_1_pSK_SK	387
			pOP-EO06085_EST_C_1_pSK_SK	540
			pOP-EO06086_EST_C_1_pSK_SK	201
			pOP-EO06088_EST_C_1_pSK_SK	685
			pOP-EO06089_EST_C_1_pSK_SK	460
			pOP-EO06091_EST_C_1_pSK_SK	658
			pOP-EO06097_EST_C_1_pSK_SK	597
			pOP-EO06103_EST_C_1_pSK_SK	620
			pOP-EO06106_EST_C_1_pSK_SK	634
			pOP-EO06111_EST_C_1_pSK_SK	582
			pOP-EO06112_EST_C_1_pSK_SK	442
			pOP-EO06118_EST_C_1_pSK_SK	411
			pOP-EO06120_EST_C_1_pSK_SK	544
			pOP-EO06121_EST_C_1_pSK_SK	631
			pOP-EO06122_EST_C_1_pSK_SK	615
			pOP-EO06123_EST_C_1_pSK_SK	634
			pOP-EO06127_EST_C_1_pSK_SK	544
			pOP-EO06138_EST_C_1_pSK_SK	603
			pOP-EO06139_EST_C_1_pSK_SK	600
			pOP-EO06140_EST_C_1_pSK_SK	288
			pOP-EO06143_EST_C_1_pSK_SK	618
			pOP-EO06146_EST_C_1_pSK_SK	548
			pOP-EO06148_EST_C_1_pSK_SK	509
			pOP-EO06149_EST_C_1_pSK_SK	532
			pOP-EO06153_EST_C_1_pSK_SK	262
			pOP-EO06154_EST_C_1_pSK_SK	469
			pOP-EO06155_EST_C_1_pSK_SK	441
			pOP-EO06158_EST_C_1_pSK_SK	450
			pOP-EO06162_EST_C_1_pSK_SK	530
			pOP-EO06163_EST_C_1_pSK_SK	532
			pOP-EO06164_EST_C_1_pSK_SK	244
			pOP-EO06167_EST_C_1_pSK_SK	413
			pOP-EO06171_EST_C_1_pSK_SK	483

			pOP-EO06173_EST_C_1_pSK_SK	599
			pOP-EO06174_EST_C_1_pSK_SK	575
			pOP-EO06176_EST_C_1_pSK_SK	297
			pOP-EO06178_EST_C_1_pSK_SK	563
			pOP-EO06185_EST_C_1_pSK_SK	565
			pOP-EO06186_EST_C_1_pSK_SK	432
			pOP-EO06189_EST_C_1_pSK_SK	321
			pOP-EO06190_EST_C_1_pSK_SK	574
			pOP-EO06193_EST_C_1_pSK_SK	444
			pOP-EO06194_EST_C_1_pSK_SK	312
			pOP-EO06196_EST_C_1_pSK_SK	670
			pOP-EO06199_EST_C_1_pSK_SK	567
			pOP-EO06200_EST_C_1_pSK_SK	113
			pOP-EO06202_EST_C_1_pSK_SK	535
			pOP-EO06203_EST_C_1_pSK_SK	582
			pOP-EO06204_EST_C_1_pSK_SK	400
			pOP-EO06210_EST_C_1_pSK_SK	596
			pOP-EO06215_EST_C_1_pSK_SK	570
			pOP-EO06219_EST_C_1_pSK_SK	620
			pOP-EO06223_EST_C_1_pSK_SK	436
			pOP-EO06224_EST_C_1_pSK_SK	310
			pOP-EO06228_EST_C_1_pSK_SK	661
			pOP-EO06231_EST_C_1_pSK_SK	606
			pOP-EO06232_EST_C_1_pSK_SK	620
			pOP-EO06233_EST_C_1_pSK_SK	626
			pOP-EO06235_EST_C_1_pSK_SK	682
			pOP-EO06238_EST_C_1_pSK_SK	714
			pOP-EO06243_EST_C_1_pSK_SK	598
			pOP-EO06244_EST_C_1_pSK_SK	596
			pOP-EO06245_EST_C_1_pSK_SK	678
			pOP-EO06247_EST_C_1_pSK_SK	668
			pOP-EO06248_EST_C_1_pSK_SK	655
			pOP-EO06249_EST_C_1_pSK_SK	643
			pOP-EO06255_EST_C_1_pSK_SK	704
			pOP-EO06256_EST_C_1_pSK_SK	674
			pOP-EO06257_EST_C_1_pSK_SK	102
			pOP-EO06259_EST_C_1_pSK_SK	243
			pOP-EO06260_EST_C_1_pSK_SK	606
			pOP-EO06263_EST_C_1_pSK_SK	670
			pOP-EO06267_EST_C_1_pSK_SK	697
			pOP-EO06269_EST_C_1_pSK_SK	616
			pOP-EO06270_EST_C_1_pSK_SK	617
			pOP-EO06271_EST_C_1_pSK_SK	599
			pOP-EO06273_EST_C_1_pSK_SK	533
			pOP-EO06276_EST_C_1_pSK_SK	608
			pOP-EO06278_EST_C_1_pSK_SK	645
			pOP-EO06281_EST_C_1_pSK_SK	713
			pOP-EO06286_EST_C_1_pSK_SK	576
			pOP-EO06289_EST_C_1_pSK_SK	632
			pOP-EO06291_EST_C_1_pSK_SK	485
			pOP-EO06292_EST_C_1_pSK_SK	385
			pOP-EO06302_EST_C_1_pSK_SK	646
			pOP-EO06304_EST_C_1_pSK_SK	613
			pOP-EO06305_EST_C_1_pSK_SK	626
			pOP-EO06306_EST_C_1_pSK_SK	278
			pOP-EO06310_EST_C_1_pSK_SK	531

			pOP-EO06312_EST_C_1_pSK_SK	624
			pOP-EO06313_EST_C_1_pSK_SK	683
			pOP-EO06315_EST_C_1_pSK_SK	559
			pOP-EO06318_EST_C_1_pSK_SK	107
			pOP-EO06322_EST_C_1_pSK_SK	654
			pOP-EO06324_EST_C_1_pSK_SK	657
			pOP-EO06326_EST_C_1_pSK_SK	353
			pOP-EO06330_EST_C_1_pSK_SK	602
			pOP-EO06333_EST_C_1_pSK_SK	724
			pOP-EO06334_EST_C_1_pSK_SK	458
			pOP-EO06335_EST_C_1_pSK_SK	627
			pOP-EO06338_EST_C_1_pSK_SK	540
			pOP-EO06339_EST_C_1_pSK_SK	717
			pOP-EO06341_EST_C_1_pSK_SK	711
			pOP-EO06342_EST_C_1_pSK_SK	770
			pOP-EO06343_EST_C_1_pSK_SK	678
			pOP-EO06344_EST_C_1_pSK_SK	743
			pOP-EO06347_EST_C_1_pSK_SK	372
			pOP-EO06349_EST_C_1_pSK_SK	676
			pOP-EO06350_EST_C_1_pSK_SK	496
			pOP-EO06351_EST_C_1_pSK_SK	667
			pOP-EO06353_EST_C_1_pSK_SK	472
			pOP-EO06355_EST_C_1_pSK_SK	692
			pOP-EO06360_EST_C_1_pSK_SK	705
			pOP-EO06363_EST_C_1_pSK_SK	645
			pOP-EO06364_EST_C_1_pSK_SK	737
			pOP-EO06369_EST_C_1_pSK_SK	490
			pOP-EO06370_EST_C_1_pSK_SK	741
			pOP-EO06371_EST_C_1_pSK_SK	595
			pOP-EO06374_EST_C_1_pSK_SK	100
			pOP-EO06375_EST_C_1_pSK_SK	559
			pOP-EO06382_EST_C_1_pSK_SK	729
			pOP-EO06385_EST_C_1_pSK_SK	737
			pOP-EO06386_EST_C_1_pSK_SK	420
			pOP-EO06387_EST_C_1_pSK_SK	803
			pOP-EO06388_EST_C_1_pSK_SK	576
			pOP-EO06389_EST_C_1_pSK_SK	691
			pOP-EO06391_EST_C_1_pSK_SK	603
			pOP-EO06393_EST_C_1_pSK_SK	768
			pOP-EO06397_EST_C_1_pSK_SK	786
			pOP-EO06398_EST_C_1_pSK_SK	789
			pOP-EO06399_EST_C_1_pSK_SK	763
			pOP-EO06400_EST_C_1_pSK_SK	706
			pOP-EO06410_EST_C_1_pSK_SK	823
			pOP-EO06413_EST_C_1_pSK_SK	786
			pOP-EO06416_EST_C_1_pSK_SK	693
			pOP-EO06420_EST_C_1_pSK_SK	729
			pOP-EO06422_EST_C_1_pSK_SK	769
			pOP-EO06424_EST_C_1_pSK_SK	719
			pOP-EO06435_EST_C_1_pSK_SK	734
			pOP-EO06436_EST_C_1_pSK_SK	694
			pOP-EO06439_EST_C_1_pSK_SK	763
			pOP-EO06440_EST_C_1_pSK_SK	735
			pOP-EO06443_EST_C_1_pSK_SK	727
			pOP-EO06449_EST_C_1_pSK_SK	757
			pOP-EO06451_EST_C_1_pSK_SK	775

			pOP-EO06453_EST_C_1_pSK_SK	710
			pOP-EO06454_EST_C_1_pSK_SK	225
			pOP-EO06455_EST_C_1_pSK_SK	713
			pOP-EO06456_EST_C_1_pSK_SK	535
			pOP-EO06458_EST_C_1_pSK_SK	380
			pOP-EO06460_EST_C_1_pSK_SK	553
			pOP-EO06463_EST_C_1_pSK_SK	717
			pOP-EO06467_EST_C_1_pSK_SK	781
			pOP-EO06470_EST_C_1_pSK_SK	812
			pOP-EO06471_EST_C_1_pSK_SK	752
			pOP-EO06472_EST_C_1_pSK_SK	640
			pOP-EO06477_EST_C_1_pSK_SK	656
			pOP-EO06484_EST_C_1_pSK_SK	472
			pOP-EO06487_EST_C_1_pSK_SK	825
			pOP-EO06488_EST_C_1_pSK_SK	809
			pOP-EO06489_EST_C_1_pSK_SK	871
			pOP-EO06493_EST_C_1_pSK_SK	253
			pOP-EO06496_EST_C_1_pSK_SK	447
			pOP-EO06498_EST_C_1_pSK_SK	803
			pOP-EO06501_EST_C_1_pSK_SK	747
			pOP-EO06503_EST_C_1_pSK_SK	607
			pOP-EO06510_EST_C_1_pSK_SK	530
			pOP-EO06515_EST_C_1_pSK_SK	791
			pOP-EO06516_EST_C_1_pSK_SK	757
			pOP-EO06518_EST_C_1_pSK_SK	794
			pOP-EO06520_EST_C_1_pSK_SK	397
			pOP-EO06522_EST_C_1_pSK_SK	799
			pOP-EO06523_EST_C_1_pSK_SK	222
			pOP-EO06527_EST_C_1_pSK_SK	821
			pOP-EO06537_EST_C_1_pSK_SK	763
			pOP-EO06538_EST_C_1_pSK_SK	758
			pOP-EO06541_EST_C_1_pSK_SK	485
			pOP-EO06550_EST_C_1_pSK_SK	780
			pOP-EO06551_EST_C_1_pSK_SK	598
			pOP-EO06563_EST_C_1_pSK_SK	514
			pOP-EO06566_EST_C_1_pSK_SK	631
			pOP-EO06568_EST_C_1_pSK_SK	584
			pOP-EO06572_EST_C_1_pSK_SK	838
			pOP-EO06574_EST_C_1_pSK_SK	511
			pOP-EO06580_EST_C_1_pSK_SK	843
			pOP-EO06582_EST_C_1_pSK_SK	550
			pOP-EO06583_EST_C_1_pSK_SK	726
			pOP-EO06584_EST_C_1_pSK_SK	802
			pOP-EO06586_EST_C_1_pSK_SK	102
			pOP-EO06588_EST_C_1_pSK_SK	831
			pOP-EO06591_EST_C_1_pSK_SK	684
			pOP-EO06593_EST_C_1_pSK_SK	849
			pOP-EO06596_EST_C_1_pSK_SK	837
			pOP-EO06597_EST_C_1_pSK_SK	785
			pOP-EO06598_EST_C_1_pSK_SK	791
			pOP-EO06601_EST_C_1_pSK_SK	935
			pOP-EO06604_EST_C_1_pSK_SK	621
			pOP-EO06605_EST_C_1_pSK_SK	827
			pOP-EO06657_EST_C_1_pSK_SK	297
			pOP-EO06709_EST_C_1_pSK_SK	172
			pOP-EO06766_EST_C_1_pSK_SK	442

			pOP-EO06810_EST_C_1_pSK_SK	578
			pOP-EO06813_EST_C_1_pSK_SK	671
			pOP-EO06818_EST_C_1_pSK_SK	542
			pOP-EO06819_EST_C_1_pSK_SK	637
			pOP-EO06824_EST_C_1_pSK_SK	348
			pOP-EO06825_EST_C_1_pSK_SK	761
			pOP-EO06827_EST_C_1_pSK_SK	741
			pOP-EO06828_EST_C_1_pSK_SK	491
			pOP-EO06830_EST_C_1_pSK_SK	715
			pOP-EO06832_EST_C_1_pSK_SK	764
			pOP-EO06833_EST_C_1_pSK_SK	747
			pOP-EO06840_EST_C_1_pSK_SK	735
			pOP-EO06841_EST_C_1_pSK_SK	742
			pOP-EO06846_EST_C_1_pSK_SK	254
			pOP-EO06848_EST_C_1_pSK_SK	492
			pOP-EO06858_EST_C_1_pSK_SK	576
			pOP-EO06859_EST_C_1_pSK_SK	850
			pOP-EO06860_EST_C_1_pSK_SK	780
			pOP-EO06866_EST_C_1_pSK_SK	906
			pOP-EO06867_EST_C_1_pSK_SK	796
			pOP-EO06871_EST_C_1_pSK_SK	793
			pOP-EO06874_EST_C_1_pSK_SK	709
			pOP-EO06876_EST_C_1_pSK_SK	457
			pOP-EO06878_EST_C_1_pSK_SK	805
			pOP-EO06879_EST_C_1_pSK_SK	488
			pOP-EO06880_EST_C_1_pSK_SK	936
			pOP-EO06881_EST_C_1_pSK_SK	847
			pOP-EO06887_EST_C_1_pSK_SK	637
			pOP-EO06891_EST_C_1_pSK_SK	841
			pOP-EO06893_EST_C_1_pSK_SK	601
			pOP-EO06894_EST_C_1_pSK_SK	122
			pOP-EO06895_EST_C_1_pSK_SK	792
			pOP-EO06898_EST_C_1_pSK_SK	756
			pOP-EO06901_EST_C_1_pSK_SK	330
			pOP-EO06903_EST_C_1_pSK_SK	747
			pOP-EO06904_EST_C_1_pSK_SK	642
			pOP-EO06906_EST_C_1_pSK_SK	681
			pOP-EO06914_EST_C_1_pSK_SK	688
			pOP-EO06915_EST_C_1_pSK_SK	737
			pOP-EO06916_EST_C_1_pSK_SK	775
			pOP-EO06919_EST_C_1_pSK_SK	617
			pOP-EO06920_EST_C_1_pSK_SK	287
			pOP-EO06922_EST_C_1_pSK_SK	247
			pOP-EO06925_EST_C_1_pSK_SK	308
			pOP-EO06928_EST_C_1_pSK_SK	703
			pOP-EO06930_EST_C_1_pSK_SK	532
			pOP-EO06931_EST_C_1_pSK_SK	246
			pOP-EO06934_EST_C_1_pSK_SK	696
			pOP-EO06935_EST_C_1_pSK_SK	681
			pOP-EO06936_EST_C_1_pSK_SK	667
			pOP-EO06937_EST_C_1_pSK_SK	777
			pOP-EO06938_EST_C_1_pSK_SK	761
			pOP-EO06939_EST_C_1_pSK_SK	728
			pOP-EO06940_EST_C_1_pSK_SK	378
			pOP-EO06942_EST_C_1_pSK_SK	735
			pOP-EO06945_EST_C_1_pSK_SK	714

			pOP-EO06946_EST_C_1_pSK_SK	714
			pOP-EO06948_EST_C_1_pSK_SK	735
			pOP-EO06949_EST_C_1_pSK_SK	671
			pOP-EO06955_EST_C_1_pSK_SK	804
			pOP-EO06958_EST_C_1_pSK_SK	301
			pOP-EO06959_EST_C_1_pSK_SK	721
			pOP-EO06961_EST_C_1_pSK_SK	783
			pOP-EO06963_EST_C_1_pSK_SK	734
			pOP-EO06965_EST_C_1_pSK_SK	683
			pOP-EO06966_EST_C_1_pSK_SK	737
			pOP-EO06970_EST_C_1_pSK_SK	538
			pOP-EO06971_EST_C_1_pSK_SK	785
			pOP-EO06972_EST_C_1_pSK_SK	720
			pOP-EO06973_EST_C_1_pSK_SK	826
			pOP-EO06975_EST_C_1_pSK_SK	764
			pOP-EO06978_EST_C_1_pSK_SK	747
			pOP-EO06981_EST_C_1_pSK_SK	640
			pOP-EO06987_EST_C_1_pSK_SK	517
			pOP-EO06991_EST_C_1_pSK_SK	584
			pOP-EO06994_EST_C_1_pSK_SK	753
			pOP-EO06997_EST_C_1_pSK_SK	295
			pOP-EO06999_EST_C_1_pSK_SK	585
			pOP-EO07004_EST_C_1_pSK_SK	661
			pOP-EO07010_EST_C_1_pSK_SK	333
			pOP-EO07013_EST_C_1_pSK_SK	650
			pOP-EO07017_EST_C_1_pSK_SK	573
			pOP-EO07018_EST_C_1_pSK_SK	335
			pOP-EO07020_EST_C_1_pSK_SK	372
			pOP-EO07034_EST_C_1_pSK_SK	639
			pOP-EO07035_EST_C_1_pSK_SK	646
			pOP-EO07036_EST_C_1_pSK_SK	679
			pOP-EO07039_EST_C_1_pSK_SK	598
			pOP-EO07042_EST_C_1_pSK_SK	679
			pOP-EO07048_EST_C_1_pSK_SK	587
			pOP-EO07050_EST_C_1_pSK_SK	633
			pOP-EO07051_EST_C_1_pSK_SK	600
			pOP-EO07052_EST_C_1_pSK_SK	596
			pOP-EO07054_EST_C_1_pSK_SK	615
			pOP-EO07059_EST_C_1_pSK_SK	560
			pOP-EO07060_EST_C_1_pSK_SK	590
			pOP-EO07062_EST_C_1_pSK_SK	523
			pOP-EO07064_EST_C_1_pSK_SK	629
			pOP-EO07068_EST_C_1_pSK_SK	662
			pOP-EO07069_EST_C_1_pSK_SK	563
			pOP-EO07070_EST_C_1_pSK_SK	592
			pOP-EO07073_EST_C_1_pSK_SK	605
			pOP-EO07080_EST_C_1_pSK_SK	298
			pOP-EO07083_EST_C_1_pSK_SK	623
			pOP-EO07085_EST_C_1_pSK_SK	471
			pOP-EO07086_EST_C_1_pSK_SK	659
			pOP-EO07089_EST_C_1_pSK_SK	607
			pOP-EO07091_EST_C_1_pSK_SK	557
			pOP-EO07097_EST_C_1_pSK_SK	578
			pOP-EO07101_EST_C_1_pSK_SK	432
			pOP-EO07102_EST_C_1_pSK_SK	596
			pOP-EO07103_EST_C_1_pSK_SK	507

			pOP-EO07105_EST_C_1_pSK_SK	607
			pOP-EO07106_EST_C_1_pSK_SK	390
			pOP-EO07109_EST_C_1_pSK_SK	326
			pOP-EO07120_EST_C_1_pSK_SK	485
			pOP-EO07121_EST_C_1_pSK_SK	555
			pOP-EO07123_EST_C_1_pSK_SK	572
			pOP-EO07130_EST_C_1_pSK_SK	630
			pOP-EO07133_EST_C_1_pSK_SK	654
			pOP-EO07137_EST_C_1_pSK_SK	241
			pOP-EO07139_EST_C_1_pSK_SK	612
			pOP-EO07145_EST_C_1_pSK_SK	479
			pOP-EO07150_EST_C_1_pSK_SK	580
			pOP-EO07153_EST_C_1_pSK_SK	643
			pOP-EO07154_EST_C_1_pSK_SK	621
			pOP-EO07156_EST_C_1_pSK_SK	687
			pOP-EO07158_EST_C_1_pSK_SK	319
			pOP-EO07166_EST_C_1_pSK_SK	445
			pOP-EO07167_EST_C_1_pSK_SK	636
			pOP-EO07171_EST_C_1_pSK_SK	573
			pOP-EO07173_EST_C_1_pSK_SK	554
			pOP-EO07176_EST_C_1_pSK_SK	665
			pOP-EO07179_EST_C_1_pSK_SK	637
			pOP-EO07180_EST_C_1_pSK_SK	729
			pOP-EO07181_EST_C_1_pSK_SK	614
			pOP-EO07182_EST_C_1_pSK_SK	549
			pOP-EO07185_EST_C_1_pSK_SK	422
			pOP-EO07186_EST_C_1_pSK_SK	653
			pOP-EO07188_EST_C_1_pSK_SK	532
			pOP-EO07189_EST_C_1_pSK_SK	428
			pOP-EO07196_EST_C_1_pSK_SK	775
			pOP-EO07199_EST_C_1_pSK_SK	723
			pOP-EO07206_EST_C_1_pSK_SK	674
			pOP-EO07211_EST_C_1_pSK_SK	794
			pOP-EO07219_EST_C_1_pSK_SK	708
			pOP-EO07220_EST_C_1_pSK_SK	596
			pOP-EO07223_EST_C_1_pSK_SK	719
			pOP-EO07226_EST_C_1_pSK_SK	204
			pOP-EO07231_EST_C_1_pSK_SK	701
			pOP-EO07234_EST_C_1_pSK_SK	342
			pOP-EO07238_EST_C_1_pSK_SK	372
			pOP-EO07240_EST_C_1_pSK_SK	771
			pOP-EO07241_EST_C_1_pSK_SK	457
			pOP-EO07242_EST_C_1_pSK_SK	764
			pOP-EO07243_EST_C_1_pSK_SK	750
			pOP-EO07244_EST_C_1_pSK_SK	722
			pOP-EO07245_EST_C_1_pSK_SK	704
			pOP-EO07253_EST_C_1_pSK_SK	654
			pOP-EO07254_EST_C_1_pSK_SK	427
			pOP-EO07259_EST_C_1_pSK_SK	721
			pOP-EO07260_EST_C_1_pSK_SK	720
			pOP-EO07261_EST_C_1_pSK_SK	684
			pOP-EO07267_EST_C_1_pSK_SK	761
			pOP-EO07278_EST_C_1_pSK_SK	684
			pOP-EO07285_EST_C_1_pSK_SK	732
			pOP-EO07286_EST_C_1_pSK_SK	750
			pOP-EO07289_EST_C_1_pSK_SK	804

			pOP-EO07294_EST_C_1_pSK_SK	803
			pOP-EO07295_EST_C_1_pSK_SK	805
			pOP-EO07301_EST_C_1_pSK_SK	503
			pOP-EO07302_EST_C_1_pSK_SK	774
			pOP-EO07303_EST_C_1_pSK_SK	790
			pOP-EO07305_EST_C_1_pSK_SK	199
			pOP-EO07312_EST_C_1_pSK_SK	713
			pOP-EO07313_EST_C_1_pSK_SK	801
			pOP-EO07318_EST_C_1_pSK_SK	662
			pOP-EO07319_EST_C_1_pSK_SK	778
			pOP-EO07323_EST_C_1_pSK_SK	809
			pOP-EO07325_EST_C_1_pSK_SK	732
			pOP-EO07327_EST_C_1_pSK_SK	611
			pOP-EO07340_EST_C_1_pSK_SK	747
			pOP-EO07341_EST_C_1_pSK_SK	523
			pOP-EO07344_EST_C_1_pSK_SK	818
			pOP-EO07345_EST_C_1_pSK_SK	383
			pOP-EO07350_EST_C_1_pSK_SK	260
			pOP-EO07352_EST_C_1_pSK_SK	855
			pOP-EO07357_EST_C_1_pSK_SK	627
			pOP-EO07359_EST_C_1_pSK_SK	643
			pOP-EO07362_EST_C_1_pSK_SK	606
			pOP-EO07366_EST_C_1_pSK_SK	802
			pOP-EO07369_EST_C_1_pSK_SK	312
			pOP-EO07370_EST_C_1_pSK_SK	820
			pOP-EO07378_EST_C_1_pSK_SK	685
			pOP-EO07383_EST_C_1_pSK_SK	840
			pOP-EO07387_EST_C_1_pSK_SK	799
			pOP-EO07388_EST_C_1_pSK_SK	774
			pOP-EO07390_EST_C_1_pSK_SK	710
			pOP-EO07396_EST_C_1_pSK_SK	784
			pOP-EO07399_EST_C_1_pSK_SK	641
			pOP-EO07401_EST_C_1_pSK_SK	470
			pOP-EO07406_EST_C_1_pSK_SK	735
			pOP-EO07408_EST_C_1_pSK_SK	769
			pOP-EO07409_EST_C_1_pSK_SK	658
			pOP-EO07423_EST_C_1_pSK_SK	801
			pOP-EO07431_EST_C_1_pSK_SK	740
			pOP-EO07435_EST_C_1_pSK_SK	727
			pOP-EO07436_EST_C_1_pSK_SK	765
			pOP-EO07444_EST_C_1_pSK_SK	742
			pOP-EO07445_EST_C_1_pSK_SK	837
			pOP-EO07447_EST_C_1_pSK_SK	777
			pOP-EO07448_EST_C_1_pSK_SK	839
			pOP-EO07449_EST_C_1_pSK_SK	523
			pOP-EO07455_EST_C_1_pSK_SK	802
			pOP-EO07458_EST_C_1_pSK_SK	662
			pOP-EO07459_EST_C_1_pSK_SK	783
			pOP-EO07460_EST_C_1_pSK_SK	374
			pOP-EO07461_EST_C_1_pSK_SK	873
			pOP-EO07466_EST_C_1_pSK_SK	682
			pOP-EO07471_EST_C_1_pSK_SK	413
			pOP-EO07474_EST_C_1_pSK_SK	815
			pOP-EO07478_EST_C_1_pSK_SK	780
			pOP-EO07483_EST_C_1_pSK_SK	790
			pOP-EO07484_EST_C_1_pSK_SK	756

			pOP-EO07486_EST_C_1_pSK_SK	735
			pOP-EO07491_EST_C_1_pSK_SK	770
			pOP-EO07495_EST_C_1_pSK_SK	710
			pOP-EO07498_EST_C_1_pSK_SK	481
			pOP-EO07500_EST_C_1_pSK_SK	733
			pOP-EO07511_EST_C_1_pSK_SK	223
			pOP-EO07518_EST_C_1_pSK_SK	674
			pOP-EO07519_EST_C_1_pSK_SK	748
			pOP-EO07528_EST_C_1_pSK_SK	720
			pOP-EO07530_EST_C_1_pSK_SK	631
			pOP-EO07531_EST_C_1_pSK_SK	692
			pOP-EO07532_EST_C_1_pSK_SK	798
			pOP-EO07537_EST_C_1_pSK_SK	590
			pOP-EO07539_EST_C_1_pSK_SK	545
			pOP-EO07548_EST_C_1_pSK_SK	575
			pOP-EO07553_EST_C_1_pSK_SK	755
			pOP-EO07572_EST_C_1_pSK_SK	743
			pOP-EO07584_EST_C_1_pSK_SK	499
			pOP-EO07587_EST_C_1_pSK_SK	550
			pOP-EO07590_EST_C_1_pSK_SK	735
			pOP-EO07591_EST_C_1_pSK_SK	625
			pOP-EO07595_EST_C_1_pSK_SK	659
			pOP-EO07604_EST_C_1_pSK_SK	677
			pOP-EO07611_EST_C_1_pSK_SK	737
			pOP-EO07620_EST_C_1_pSK_SK	726
			pOP-EO07626_EST_C_1_pSK_SK	600
			pOP-EO07630_EST_C_1_pSK_SK	450
			pOP-EO07643_EST_C_1_pSK_SK	597
			pOP-EO07644_EST_C_1_pSK_SK	757
			pOP-EO07654_EST_C_1_pSK_SK	722
			pOP-EO07665_EST_C_1_pSK_SK	330
			pOP-EO07668_EST_C_1_pSK_SK	734
			pOP-EO07670_EST_C_1_pSK_SK	732
			pOP-EO07678_EST_C_1_pSK_SK	657
			pOP-EO07679_EST_C_1_pSK_SK	533
			pOP-EO07688_EST_C_1_pSK_SK	500
			pOP-EO07690_EST_C_1_pSK_SK	712
			pOP-EO07692_EST_C_1_pSK_SK	452
			pOP-EO07697_EST_C_1_pSK_SK	628
			pOP-EO07699_EST_C_1_pSK_SK	716
			pOP-EO07702_EST_C_1_pSK_SK	385
			pOP-EO07703_EST_C_1_pSK_SK	723
			pOP-EO07714_EST_C_1_pSK_SK	680
			pOP-EO07715_EST_C_1_pSK_SK	745
			pOP-EO07716_EST_C_1_pSK_SK	278
			pOP-EO07722_EST_C_1_pSK_SK	537
			pOP-EO07727_EST_C_1_pSK_SK	598
			pOP-EO07729_EST_C_1_pSK_SK	415
			pOP-EO07730_EST_C_1_pSK_SK	746
			pOP-EO07732_EST_C_1_pSK_SK	672
			pOP-EO07733_EST_C_1_pSK_SK	697
			pOP-EO07740_EST_C_1_pSK_SK	749
			pOP-EO07748_EST_C_1_pSK_SK	668
			pOP-EO07750_EST_C_1_pSK_SK	490
			pOP-EO07757_EST_C_1_pSK_SK	732
			pOP-EO07764_EST_C_1_pSK_SK	753

			pOP-EO07774_EST_C_1_pSK_SK	692
			pOP-EO07783_EST_C_1_pSK_SK	833
			pOP-EO07785_EST_C_1_pSK_SK	744
			pOP-EO07799_EST_C_1_pSK_SK	242
			pOP-EO07802_EST_C_1_pSK_SK	776
			pOP-EO07803_EST_C_1_pSK_SK	768
			pOP-EO07804_EST_C_1_pSK_SK	500
			pOP-EO07820_EST_C_1_pSK_SK	795
			pOP-EO07825_EST_C_1_pSK_SK	289
			pOP-EO07826_EST_C_1_pSK_SK	832
			pOP-EO07830_EST_C_1_pSK_SK	806
			pOP-EO07831_EST_C_1_pSK_SK	833
			pOP-EO07834_EST_C_1_pSK_SK	770
			pOP-EO07842_EST_C_1_pSK_SK	816
			pOP-EO07855_EST_C_1_pSK_SK	683
			pOP-EO07860_EST_C_1_pSK_SK	560
			pOP-EO07861_EST_C_1_pSK_SK	879
			pOP-EO07865_EST_C_1_pSK_SK	532
			pOP-EO07866_EST_C_1_pSK_SK	435
			pOP-EO07874_EST_C_1_pSK_SK	620
			pOP-EO07875_EST_C_1_pSK_SK	484
			pOP-EO07876_EST_C_1_pSK_SK	577
			pOP-EO07878_EST_C_1_pSK_SK	551
			pOP-EO07879_EST_C_1_pSK_SK	410
			pOP-EO07880_EST_C_1_pSK_SK	654
			pOP-EO07882_EST_C_1_pSK_SK	127
			pOP-EO07884_EST_C_1_pSK_SK	189
			pOP-EO07885_EST_C_1_pSK_SK	431
			pOP-EO07887_EST_C_1_pSK_SK	586
			pOP-EO07890_EST_C_1_pSK_SK	227
			pOP-EO07892_EST_C_1_pSK_SK	594
			pOP-EO07895_EST_C_1_pSK_SK	396
			pOP-EO07909_EST_C_1_pSK_SK	488
			pOP-EO07912_EST_C_1_pSK_SK	486
			pOP-EO07914_EST_C_1_pSK_SK	344
			pOP-EO07919_EST_C_1_pSK_SK	576
			pOP-EO07922_EST_C_1_pSK_SK	322
			pOP-EO07927_EST_C_1_pSK_SK	365
			pOP-EO07930_EST_C_1_pSK_SK	741
			pOP-EO07934_EST_C_1_pSK_SK	487
			pOP-EO07935_EST_C_1_pSK_SK	662
			pOP-EO07938_EST_C_1_pSK_SK	481
			pOP-EO07939_EST_C_1_pSK_SK	749
			pOP-EO07942_EST_C_1_pSK_SK	655
			pOP-EO07946_EST_C_1_pSK_SK	749
			pOP-EO07947_EST_C_1_pSK_SK	636
			pOP-EO07949_EST_C_1_pSK_SK	653
			pOP-EO07950_EST_C_1_pSK_SK	539
			pOP-EO07951_EST_C_1_pSK_SK	647
			pOP-EO07952_EST_C_1_pSK_SK	471
			pOP-EO07955_EST_C_1_pSK_SK	763
			pOP-EO07959_EST_C_1_pSK_SK	608
			pOP-EO07963_EST_C_1_pSK_SK	412
			pOP-EO07964_EST_C_1_pSK_SK	339
			pOP-EO07966_EST_C_1_pSK_SK	534
			pOP-EO07969_EST_C_1_pSK_SK	470

			pOP-EO07970_EST_C_1_pSK_SK	319
			pOP-EO07971_EST_C_1_pSK_SK	512
			pOP-EO07972_EST_C_1_pSK_SK	433
			pOP-EO07974_EST_C_1_pSK_SK	324
			pOP-EO07976_EST_C_1_pSK_SK	357
			pOP-EO07977_EST_C_1_pSK_SK	476
			pOP-EO07978_EST_C_1_pSK_SK	446
			pOP-EO07982_EST_C_1_pSK_SK	426
			pOP-EO07987_EST_C_1_pSK_SK	367
			pOP-EO07990_EST_C_1_pSK_SK	275
			pOP-EO07991_EST_C_1_pSK_SK	360
			pOP-EO07994_EST_C_1_pSK_SK	107
			pOP-EO07996_EST_C_1_pSK_SK	198
			pOP-EO07997_EST_C_1_pSK_SK	306
			pOP-EO07999_EST_C_1_pSK_SK	571
			pOP-EO08001_EST_C_1_pSK_SK	451
			pOP-EO08005_EST_C_1_pSK_SK	239
			pOP-EO08007_EST_C_1_pSK_SK	425
			pOP-EO08009_EST_C_1_pSK_SK	381
			pOP-EO08020_EST_C_1_pSK_SK	453
			pOP-EO08023_EST_C_1_pSK_SK	322
			pOP-EO08027_EST_C_1_pSK_SK	279
			pOP-EO08033_EST_C_1_pSK_SK	393
			pOP-EO08035_EST_C_1_pSK_SK	438
			pOP-EO08036_EST_C_1_pSK_SK	332
			pOP-EO08037_EST_C_1_pSK_SK	132
			pOP-EO08039_EST_C_1_pSK_SK	322
			pOP-EO08043_EST_C_1_pSK_SK	230
			pOP-EO08044_EST_C_1_pSK_SK	389
			pOP-EO08046_EST_C_1_pSK_SK	395
			pOP-EO08049_EST_C_1_pSK_SK	383
			pOP-EO08050_EST_C_1_pSK_SK	403
			pOP-EO08052_EST_C_1_pSK_SK	428
			pOP-EO08057_EST_C_1_pSK_SK	552
			pOP-EO08058_EST_C_1_pSK_SK	500
			pOP-EO08062_EST_C_1_pSK_SK	419
			pOP-EO08065_EST_C_1_pSK_SK	530
			pOP-EO08070_EST_C_1_pSK_SK	627
			pOP-EO08073_EST_C_1_pSK_SK	542
			pOP-EO08074_EST_C_1_pSK_SK	506
			pOP-EO08075_EST_C_1_pSK_SK	462
			pOP-EO08078_EST_C_1_pSK_SK	468
			pOP-EO08084_EST_C_1_pSK_SK	540
			pOP-EO08085_EST_C_1_pSK_SK	420
			pOP-EO08086_EST_C_1_pSK_SK	103
			pOP-EO08088_EST_C_1_pSK_SK	540
			pOP-EO08094_EST_C_1_pSK_SK	305
			pOP-EO08097_EST_C_1_pSK_SK	510
			pOP-EO08100_EST_C_1_pSK_SK	631
			pOP-EO08107_EST_C_1_pSK_SK	608
			pOP-EO08109_EST_C_1_pSK_SK	668
			pOP-EO08111_EST_C_1_pSK_SK	608
			pOP-EO08114_EST_C_1_pSK_SK	156
			pOP-EO08116_EST_C_1_pSK_SK	210
			pOP-EO08123_EST_C_1_pSK_SK	608
			pOP-EO08124_EST_C_1_pSK_SK	506

			pOP-EO08127_EST_C_1_pSK_SK	507	
			pOP-EO08128_EST_C_1_pSK_SK	145	
			pOP-EO08132_EST_C_1_pSK_SK	543	
			pOP-EO08134_EST_C_1_pSK_SK	670	
			pOP-EO08136_EST_C_1_pSK_SK	445	
			pOP-EO08144_EST_C_1_pSK_SK	222	
			pOP-EO08147_EST_C_1_pSK_SK	580	
			pOP-EO08148_EST_C_1_pSK_SK	600	
			pOP-EO08149_EST_C_1_pSK_SK	568	
			pOP-EO08150_EST_C_1_pSK_SK	553	
			pOP-EO08154_EST_C_1_pSK_SK	594	
			pOP-EO08158_EST_C_1_pSK_SK	494	
			pOP-EO08160_EST_C_1_pSK_SK	491	
			pOP-EO08161_EST_C_1_pSK_SK	488	
			pOP-EO08162_EST_C_1_pSK_SK	520	
			pOP-EO08163_EST_C_1_pSK_SK	555	
			pOP-EO08164_EST_C_1_pSK_SK	481	
			pOP-EO08166_EST_C_1_pSK_SK	585	
			pOP-EO08173_EST_C_1_pSK_SK	497	
			pOP-EO08174_EST_C_1_pSK_SK	156	
			pOP-EO08176_EST_C_1_pSK_SK	465	
			pOP-EO08180_EST_C_1_pSK_SK	464	
			pOP-EO08182_EST_C_1_pSK_SK	408	
			pOP-EO08195_EST_C_1_pSK_SK	649	
			pOP-EO08196_EST_C_1_pSK_SK	647	
			pOP-EO08197_EST_C_1_pSK_SK	657	
			pOP-EO08199_EST_C_1_pSK_SK	524	
			pOP-EO08205_EST_C_1_pSK_SK	603	
			pOP-EO08206_EST_C_1_pSK_SK	531	
			pOP-EO08208_EST_C_1_pSK_SK	627	
			pOP-EO08215_EST_C_1_pSK_SK	308	
			pOP-EO08216_EST_C_1_pSK_SK	456	
			pOP-EO08217_EST_C_1_pSK_SK	321	
			pOP-EO08219_EST_C_1_pSK_SK	417	
			pOP-EO08222_EST_C_1_pSK_SK	423	
			pOP-EO08224_EST_C_1_pSK_SK	608	
			pOP-EO08225_EST_C_1_pSK_SK	533	
			pOP-EO08227_EST_C_1_pSK_SK	624	
			pOP-EO08228_EST_C_1_pSK_SK	594	
			pOP-EO08229_EST_C_1_pSK_SK	333	
			pOP-EO08230_EST_C_1_pSK_SK	338	
			pOP-EO08231_EST_C_1_pSK_SK	528	
			pOP-EO08234_EST_C_1_pSK_SK	533	
			pOP-EO08238_EST_C_1_pSK_SK	533	
			pOP-EO08243_EST_C_1_pSK_SK	511	
			pOP-EO08246_EST_C_1_pSK_SK	435	
			pOP-EO08248_EST_C_1_pSK_SK	441	
			pOP-EO08253_EST_C_1_pSK_SK	441	
			pOP-EO08256_EST_C_1_pSK_SK	423	
			pOP-EO08257_EST_C_1_pSK_SK	544	
			pOP-EO08260_EST_C_1_pSK_SK	508	
			pOP-EO08261_EST_C_1_pSK_SK	376	
			pOP-EO08263_EST_C_1_pSK_SK	321	
			pOP-EO08267_EST_C_1_pSK_SK	508	
			pOP-EO08268_EST_C_1_pSK_SK	436	
			pOP-EO08273_EST_C_1_pSK_SK	538	

			pOP-EO08277_EST_C_1_pSK_SK	359
			pOP-EO08278_EST_C_1_pSK_SK	353
			pOP-EO08279_EST_C_1_pSK_SK	425
			pOP-EO08283_EST_C_1_pSK_SK	390
			pOP-EO08285_EST_C_1_pSK_SK	361
			pOP-EO08287_EST_C_1_pSK_SK	420
			pOP-EO08288_EST_C_1_pSK_SK	510
			pOP-EO08290_EST_C_1_pSK_SK	237
			pOP-EO08291_EST_C_1_pSK_SK	429
			pOP-EO08292_EST_C_1_pSK_SK	445
			pOP-EO08293_EST_C_1_pSK_SK	324
			pOP-EO08298_EST_C_1_pSK_SK	564
			pOP-EO08303_EST_C_1_pSK_SK	200
			pOP-EO08304_EST_C_1_pSK_SK	643
			pOP-EO08305_EST_C_1_pSK_SK	415
			pOP-EO08306_EST_C_1_pSK_SK	405
			pOP-EO08307_EST_C_1_pSK_SK	474
			pOP-EO08309_EST_C_1_pSK_SK	310
			pOP-EO08311_EST_C_1_pSK_SK	614
			pOP-EO08314_EST_C_1_pSK_SK	444
			pOP-EO08317_EST_C_1_pSK_SK	476
			pOP-EO08323_EST_C_1_pSK_SK	462
			pOP-EO08326_EST_C_1_pSK_SK	328
			pOP-EO08331_EST_C_1_pSK_SK	603
			pOP-EO08332_EST_C_1_pSK_SK	573
			pOP-EO08333_EST_C_1_pSK_SK	607
			pOP-EO08335_EST_C_1_pSK_SK	550
			pOP-EO08340_EST_C_1_pSK_SK	281
			pOP-EO08341_EST_C_1_pSK_SK	581
			pOP-EO08342_EST_C_1_pSK_SK	528
			pOP-EO08344_EST_C_1_pSK_SK	482
			pOP-EO08349_EST_C_1_pSK_SK	448
			pOP-EO08351_EST_C_1_pSK_SK	458
			pOP-EO08352_EST_C_1_pSK_SK	608
			pOP-EO08358_EST_C_1_pSK_SK	313
			pOP-EO08359_EST_C_1_pSK_SK	414
			pOP-EO08361_EST_C_1_pSK_SK	150
			pOP-EO08362_EST_C_1_pSK_SK	480
			pOP-EO08363_EST_C_1_pSK_SK	254
			pOP-EO08366_EST_C_1_pSK_SK	557
			pOP-EO08367_EST_C_1_pSK_SK	488
			pOP-EO08368_EST_C_1_pSK_SK	386
			pOP-EO08371_EST_C_1_pSK_SK	297
			pOP-EO08374_EST_C_1_pSK_SK	185
			pOP-EO08377_EST_C_1_pSK_SK	458
			pOP-EO08378_EST_C_1_pSK_SK	259
			pOP-EO08379_EST_C_1_pSK_SK	373
			pOP-EO08381_EST_C_1_pSK_SK	185
			pOP-EO08384_EST_C_1_pSK_SK	490
			pOP-EO08385_EST_C_1_pSK_SK	330
			pOP-EO08387_EST_C_1_pSK_SK	465
			pOP-EO08388_EST_C_1_pSK_SK	366
			pOP-EO08393_EST_C_1_pSK_SK	296
			pOP-EO08395_EST_C_1_pSK_SK	173
			pOP-EO08397_EST_C_1_pSK_SK	206
			pOP-EO08399_EST_C_1_pSK_SK	285

			pOP-EO08401_EST_C_1_pSK_SK	426
			pOP-EO08404_EST_C_1_pSK_SK	361
			pOP-EO08405_EST_C_1_pSK_SK	376
			pOP-EO08406_EST_C_1_pSK_SK	438
			pOP-EO08407_EST_C_1_pSK_SK	354
			pOP-EO08409_EST_C_1_pSK_SK	417
			pOP-EO08411_EST_C_1_pSK_SK	414
			pOP-EO08412_EST_C_1_pSK_SK	328
			pOP-EO08414_EST_C_1_pSK_SK	384
			pOP-EO08415_EST_C_1_pSK_SK	457
			pOP-EO08416_EST_C_1_pSK_SK	422
			pOP-EO08418_EST_C_1_pSK_SK	470
			pOP-EO08419_EST_C_1_pSK_SK	489
			pOP-EO08421_EST_C_1_pSK_SK	452
			pOP-EO08422_EST_C_1_pSK_SK	594
			pOP-EO08423_EST_C_1_pSK_SK	352
			pOP-EO08424_EST_C_1_pSK_SK	483
			pOP-EO08426_EST_C_1_pSK_SK	435
			pOP-EO08427_EST_C_1_pSK_SK	399
			pOP-EO08428_EST_C_1_pSK_SK	204
			pOP-EO08429_EST_C_1_pSK_SK	403
			pOP-EO08431_EST_C_1_pSK_SK	519
			pOP-EO08435_EST_C_1_pSK_SK	422
			pOP-EO08437_EST_C_1_pSK_SK	391
			pOP-EO08438_EST_C_1_pSK_SK	403
			pOP-EO08439_EST_C_1_pSK_SK	111
			pOP-EO08440_EST_C_1_pSK_SK	174
			pOP-EO08442_EST_C_1_pSK_SK	194
			pOP-EO08444_EST_C_1_pSK_SK	339
			pOP-EO08449_EST_C_1_pSK_SK	334
			pOP-EO08450_EST_C_1_pSK_SK	324
			pOP-EO08451_EST_C_1_pSK_SK	126
			pOP-EO08456_EST_C_1_pSK_SK	250
			pOP-EO08459_EST_C_1_pSK_SK	155
			pOP-EO08461_EST_C_1_pSK_SK	167
			pOP-EO08463_EST_C_1_pSK_SK	368
			pOP-EO08465_EST_C_1_pSK_SK	113
			pOP-EO08466_EST_C_1_pSK_SK	217
			pOP-EO08468_EST_C_1_pSK_SK	260
			pOP-EO08470_EST_C_1_pSK_SK	290
			pOP-EO08473_EST_C_1_pSK_SK	122
			pOP-EO08474_EST_C_1_pSK_SK	226
			pOP-EO08475_EST_C_1_pSK_SK	132
			pOP-EO08479_EST_C_1_pSK_SK	298
			pOP-EO08480_EST_C_1_pSK_SK	272
			pOP-EO08481_EST_C_1_pSK_SK	118
			pOP-EO08482_EST_C_1_pSK_SK	105
			pOP-EO08484_EST_C_1_pSK_SK	215
			pOP-EO08485_EST_C_1_pSK_SK	249
			pOP-EO08486_EST_C_1_pSK_SK	262
			pOP-EO08488_EST_C_1_pSK_SK	181
			pOP-EO08492_EST_C_1_pSK_SK	266
			pOP-EO08493_EST_C_1_pSK_SK	112
			pOP-EO08494_EST_C_1_pSK_SK	255
			pOP-EO08497_EST_C_1_pSK_SK	108
			pOP-EO08499_EST_C_1_pSK_SK	212

			pOP-EO08501_EST_C_1_pSK_SK	104
			pOP-EO08505_EST_C_1_pSK_SK	202
			pOP-EO08506_EST_C_1_pSK_SK	247
			pOP-EO08507_EST_C_1_pSK_SK	214
			pOP-EO08508_EST_C_1_pSK_SK	204
			pOP-EO08515_EST_C_1_pSK_SK	339
			pOP-EO08517_EST_C_1_pSK_SK	383
			pOP-EO08525_EST_C_1_pSK_SK	255
			pOP-EO08526_EST_C_1_pSK_SK	344
			pOP-EO08527_EST_C_1_pSK_SK	416
			pOP-EO08528_EST_C_1_pSK_SK	330
			pOP-EO08530_EST_C_1_pSK_SK	334
			pOP-EO08531_EST_C_1_pSK_SK	312
			pOP-EOP00005_EST_C_1_pSK_SK	603
			pOP-EOP00006_EST_C_1_pSK_SK	436
			pOP-EOP00009_EST_C_1_pSK_SK	585
			pOP-EOP00017_EST_C_1_pSK_SK	592
			pOP-EOP00019_EST_C_1_pSK_SK	630
			pOP-EOP00023_EST_C_1_pSK_SK	177
